# Oral abstracts of the 21st International AIDS Conference 18–22 July 2016, Durban, South Africa

**DOI:** 10.7448/IAS.19.6.21264

**Published:** 2016-07-22

**Figure 1:**
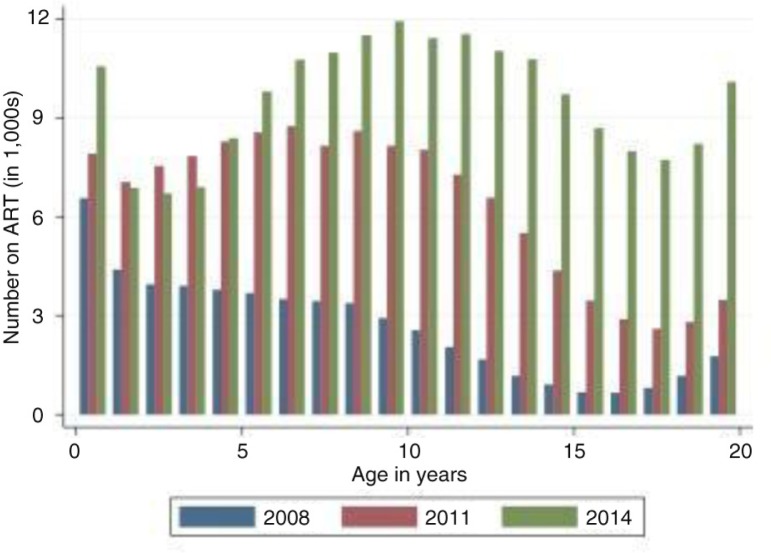
Distribution of individual viral load test results by age and period.

**Abstract TUAB0102–Table 1:** Distribution of viral load test results by age category and calendar year

	0–1 years	1–4 years	5–9 years	10–14 years	15–19 years
2004–2007	11,593 (15%)	27,157 (35%)	24,921 (32%)	8854 (11%)	5904 (8%)
2008–2011	29,983 (9%)	88,391 (26%)	110,737 (33%)	72,774 (22%)	34,981 (10%)
2012–2014	31,299 (6%)	89,530 (17%)	155,163 (30%)	141,945 (28%)	96,042 (19%)

