# Supplementary material for: Oral abstracts of the 21st International AIDS Conference 18–22 July 2016, Durban, South Africa
Source: J Int AIDS Soc. 2016 Jul 22;19(6 Suppl 5):21264. doi: 10.7448/IAS.19.6.21264 (PMC4960637; doi:10.7448/IAS.19.6.21264)
Supplement: Related texts

# Sub article: TUAA0101 Microbial translocation during hyperacute SIV infection

**Published:** 2016-07-22

**Authors:** A Ericsen, M Lauck, M Mohns, S Dinapoli, J Mutschler, J Greene, J Weinfurter, G Lehrer-Brey, K Crosno, E Peterson, M Reynolds, R Wiseman, B Burwitz, J Sacha, T Friedrich, J Brenchley, D O'Connor

**Affiliations:** 1 Department of Pathology and Laboratory Medicine, University of Wisconsin-Madison, Madison, WI, United States; 2 Virology Training Program, University of Wisconsin-Madison, Madison, WI, United States; 3 University of Wisconsin-Madison, Madison, WI, United States; 4 Laboratory of Molecular Microbiology, National Institute of Allergy and Infectious Disease, Bethesda, MD, United States; 5 Wisconsin National Primate Research Center, Madison, WI, United States; 6 Vaccine & Gene Therapy Institute, Oregon Health & Sciences University, Beaverton, OR, United States; 7 National Primate Research Center, Oregon Health & Sciences University, Beaverton, OR, United States; 8 Department of Pathobiological Sciences, University of Wisconsin-Madison, Madison, WI, United States

## Abstract

**Introduction:**

Within the first weeks of human immunodeficiency virus (HIV) infection, virus replication reaches systemic circulation. Despite the critical, causal role of virus replication in determining transmissibility and kinetics of disease progression, there is limited understanding of the conditions required to transform a small localized transmitted founder population into a large and heterogeneous systemic infection.

**Methods:**

Cynomolgus and rhesus macaques were infected with simian immunodeficiency virus (SIV) and followed longitudinally. Plasma levels of SIV were monitored using qRT-PCR. Bacterial genomic DNA in plasma was characterized and quantified longitudinally using 16S ribosomal deep sequencing and qPCR. ELISA-based assays were used to monitor intestinal permeability (IFABP) and perturbation of bacteria-specific host factors (sCD14 and EndoCab). Flow cytometry was used to track peripheral blood lymphocyte populations. In vitro assays were performed by exposing freshly isolated peripheral blood mononuclear cells to bacterial lysate prepared from major translocators. Effects of bacterial lysate on CD4+ T cell activation and CD8+ T cell cytotoxicity were measured using flow cytometry. Statistical significance was calculated using ANOVA or Wilcoxon signed-rank testing.

**Results:**

Prior to the peak of viremia, we observed a transient high-level influx of microbial genomic DNA into peripheral blood. This microbial translocation was accompanied by perturbation of bacteria-specific host factors in plasma, as well as expansion of the CD4+CCR5+ T cell compartment. Exposure of freshly isolated peripheral blood mononuclear cells to lysate prepared from major translocating taxa revealed differential taxa-specific effects on the CD4+CCR5+ T cell compartment and cytotoxic granule expression within CD8+ T cells.

**Conclusions:**

Altogether, our data identify the influx of microbial products into blood during hyperacute SIV infection as a candidate modifier of early interactions between the antiviral host response and nascent HIV infection. Over the next few months, we will explore the effect of inducing microbial translocation during SIV infection, with particular interest on microbial reactivity within the CD4+CCR5+ target cell compartment.

# Sub article: TUAA0102 Impact of a fat-rich diet on the pathogenesis of SIV infection in the African green monkey host

**Published:** 2016-07-22

**Authors:** C Xu, T He, G Haret-Richter, D Franck, B Policicchio, E Brocca-Cofano, D Ma, J Stock, R Tracy, A Landay, C Wilson, C Apetrei, I Pandrea

**Affiliations:** 1 Center for Vaccine Research, University of Pittsburgh, Pittsburgh, PA, United States; 2 University of Colorado, Denver, CO, United States; 3 University of Vermont, Burlington, VT, United States; 4 Rush University, Chicago, IL, United States

## Abstract

**Introduction:**

High dietary fats were reported to induce intestinal dysbiosis, drive gut inflammation and breakdown the intestinal epithelial barrier, granting intestinal flora access to the bloodstream. As microbial translocation is a major determinant of the chronic immune activation and HIV/SIV disease progression, we investigated whether fat diet impacts HIV/SIV pathogenesis.

**Methods:**

The non-progressive African green monkey (AGM) model of SIV is an ideal system to assess the role of fat diet on disease progression, because they do not develop SIV-related intestinal dysfunction. We included four AGMs that received a fat diet prior and after SIVsab infection, and five controls in which the impact on key parameters of SIV infection such as: viral loads, CD4^+^ T cell counts, microbial translocation, immune activation and inflammation were compared and contrasted.

**Results:**

LPS levels increased in the AGMs receiving fat diet prior and after SIV infection. Fat-rich diet also resulted in increases of immune activation (HLA-DR CD38, CD69 and Ki-67) and inflammation (inflammatory cytokines-IL-6, IL-17 and C reactive protein), leading to a prolonged depletion of CD4^+^ T cells compared to controls. However, these significant alterations of key parameters that are associated with the lack of disease progression in natural hosts of SIVs did not reach the levels described during progressive HIV/SIV infection. Furthermore, these changes did not result in significant increases in the levels of viral replication in the AGMs receiving a fat diet.

**Conclusions:**

Administration of fat-rich diet resulted in alterations of markers of pathogenicity in the non-progressive SIV infection of AGMs. Although not major, these changes were significant, suggesting that a diet very rich in fats may negatively impact HIV pathogenesis, especially if combined with other behavioural risk factors reported to impact gut integrity or systemic inflammation, such as alcohol consumption, drug usage and smoking. Detailed studies on the correlations between fat diet, alterations in the intestinal microbiota, metabolic markers, liver function and SIV progression to AIDS are in progress.

# Sub article: TUAA0103 HIV infection is associated with preservation of MAIT cells in the lungs but alteration of their phenotype and T cell receptor repertoire

**Published:** 2016-07-22

**Authors:** EB Wong, B Xulu, S Prakadan, AK Shalek, U Lalloo, P Baijnath, M Suleman, V Moodley, M Mitha, P Maharaj, C Costiniuk, M Nielsen, Z Mhlane, F Karim, D Lewinsohn, T Ndung'u

**Affiliations:** 1 KwaZulu Natal Research Institute for Tuberculosis and HIV, Durban, South Africa; 2 Division of Infectious Diseases, Massachusetts General Hospital, Boston, MA, United States; 3 Department of Medicine, Harvard Medical School, Boston, MA, United States; 4 Institute for Medical Engineering & Science, Massachusetts Institute of Technology, Cambridge, MA, United States; 5 Department of Chemistry, Massachusetts Institute of Technology, Cambridge, MA, United States; 6 Ragon Institute of Harvard, MGH and MIT, Cambridge, MA, United States; 7 Broad Institute, Cambridge, MA, United States; 8 Durban University of Technology, Durban, South Africa; 9 Department of Pulmonology and Critical Care, University of KwaZulu Natal, Durban, South Africa; 10 Department of Pulmonology and Critical Care, Inkosi Albert Luthuli Hospital, Durban, South Africa; 11 Division of Infectious Diseases, McGill University Health Center, Montreal, QC, Canada; 12 Department of Systems Biology, Technical University of Denmark, Lyngby, Denmark; 13 Division of Pulmonary Medicine and Critical Care, Oregon Health and Sciences University, Portland, OR, United States; 14 HIV Pathogenesis Programme, University of KwaZulu Natal, Durban, South Africa

## Abstract

**Introduction:**

Tuberculosis remains the leading cause of death in HIV-positive people. A better understanding of the impact of HIV on lung immunity may lead to novel immunotherapeutic interventions. MAIT cells are tissue-homing donor-unrestricted T cells with broad anti-microbial activity. HIV infection causes early and irreversible depletion of MAIT cells in the peripheral circulation, but the effect of HIV on MAIT cells in the lungs is unknown.

**Methods:**

We FACS-sorted MAIT cells from bronchoalveolar lavage (BAL) fluid and peripheral blood of HIV-infected and HIV-negative patients from Durban, South Africa. MR1-5OPRU tetramer staining was used to identify and phenotype MAIT cells based on expression of CD3, CD4, CD8, TRAV1-2, CD161 and CD26. High throughput bias-controlled TCR sequencing (ImmunoSEQ) of sorted populations enabled detailed analysis of TCRA CDR3a usage.

**Results:**

HIV infection was associated with depletion of MAIT cells in the peripheral circulation (median %5OPRU+of CD3+CD4- lymphocytes was 1.09% in HIV-negatives, 0.34% in HIV-positives, p=0.027). In contrast, MAIT cells were not depleted in the BAL compartment during HIV infection (0.68% in HIV-negatives, 0.89% in HIV-positives, p=non-significant). In HIV-negative individuals, 77.1% of circulating MAIT cells expressed the expected CD161++CD26++ phenotype, but only 43.8% of BAL MAITs expressed this phenotype (p≤0.0001). In HIV infected lungs, the frequency of MAITs with the CD161++CD26++ phenotype was significantly higher (57.6%) than in HIV-negative lungs (p=0.021). MAIT cells with canonical MAIT TCRA CDR3a rearrangements were highly shared between donors and clonally expanded in the BALs. MAIT cells with non-canonical TCRs were unique to individuals and more frequent in HIV-infection.

**Conclusions:**

We report for the first time that MAIT cells in the lungs are numerically preserved but phenotypically and clonotypically altered by HIV infection. We confirm previous reports that circulating MAIT cells are depleted in HIV. Our results suggest that peripheral MAIT cell depletions observed in HIV infection may be due to compartment-specific microbial alterations and/or tissue redistribution. Further study is needed to determine the mechanisms underlying the altered phenotypes of lung-resident MAITs and whether these can be targeted to improve anti-microbial lung immunity in people living with HIV.

# Sub article: TUAA0104 Cell-associated HIV-1 unspliced RNA level predicts both time to virological suppression and duration of post-treatment virological control in patients treated with temporary early ART

**Published:** 2016-07-22

**Authors:** A Pasternak, J Prins, B Berkhout

**Affiliations:** 1 Medical Microbiology, Academic Medical Center, University of Amsterdam, Amsterdam, The Netherlands; 2 Internal Medicine, Academic Medical Center, University of Amsterdam, Amsterdam, The Netherlands

## Abstract

**Introduction:**

For the improved design of strategies towards HIV-1 functional cure, it is important to identify biomarkers that could predict the duration of post-treatment virological control.

**Methods:**

We studied 46 patients that received 24 or 60 weeks of temporary ART initiated at primary HIV infection (PHI). Patients were treated with a quadruple triple-class ART regimen. Cell-associated HIV-1 nucleic acids were quantified by seminested real-time PCR.

**Results:**

All patients achieved virological suppression (VS) (plasma HIV-1 viremia <50 copies/ml) with a median of 21 weeks. We first assessed the predictive power of plasma viremia, total HIV-1 DNA, unspliced (US) cell-associated HIV-1 RNA, CD4^+^ T-cell count, and CD4:CD8 ratio, measured at PHI, for the time to VS. In the univariate analysis, both plasma viremia and US RNA were predictive for time to VS (p=0.016 and p=0.0033, respectively, log-rank test). In the multivariate Cox regression, US RNA at PHI was the only significant predictor of the time to VS (HR=0.65 per 1 log_10_ increase in US RNA, 95% confidence interval (CI): 0.48–0.87, p=0.0043). Subsequently, the same biomarkers were longitudinally quantified every 12 weeks during ART. All 45 patients who discontinued ART experienced virological rebound (VR) (plasma viremia >50 copies/ml) within 9 months after therapy interruption. We assessed the predictive power of the last measurements of the biomarkers on ART before the therapy interruption, as well as of the duration of temporary ART, for the time to VR (the duration of post-treatment virological control). Again, US RNA was the only significant predictor of the time to VR (HR=0.29 for patients with US RNA levels below vs. above the median, 95% CI: 0.10–0.83, p=0.021, log-rank test).

**Conclusions:**

In summary, in this cohort of patients treated at PHI, cell-associated HIV-1 US RNA level was the sole independent predictor of both virological suppression on ART and post-treatment virological control after ART discontinuation. Further exploration of the potential of this biomarker as a predictor of post-treatment control in large-scale clinical trials aimed at HIV functional cure is warranted.

# Sub article: TUAA0105 HIV-infected patients with exceptional TCD4+ recovery during effective HAART present a distinct T CD4+ differentiation pattern, higher CD31neg naïve cells and a smaller HIV reservoir

**Published:** 2016-07-22

**Authors:** L Leon-Fuentes, M Viveros-Rogel, M Vergara-Mendoza, M Rodriguez-Castañón, A Cardenas-Ochoa, A Tello-Mercado, C Vega, J Sierra-Madero, L Soto-Ramirez, S Perez-Patrigeon

**Affiliations:** Instituto Nacional de Ciencias Médicas y Nutrición Salvador Zubirán (INCMNSZ) Infectious Diseases, Mexico, Mexico

## Abstract

**Introduction:**

Clinical outcome of HIV infected patients relies on the recovery of CD4+ T cells after HAART. However, this immune recovery is variable and difficult to predict. Here, we present a cohort of patients with undetectable viral load and a follow up of 48 months of HAART who reached CD4+ T cell counts >1000 cells/mm^3^ (Hypers) and compare them to those who reached between 350 and 999 CD4+ T cells/mm^3^ (concordants). Their demographic data, immune recovery kinetics and T CD4+ subsets phenotype as well as their integrated HIV DNA were analyzed.

**Methods:**

Retrospective data were obtained from the charts of 447 undetectable patients on their first ARV regimen and a follow up of 48 months at the INCMSZ HIV cohort. For immune phenotype and reservoir analysis, 20 Hypers and 19 Concordants matched by sex, age and T CD4+ nadir were available. The following subsets were analyzed by Flow cytometry on whole blood: naïve T-cells, central memory T-cells, effector memory and terminally differentiated. A two-step quantitative real-time PCR (qPCR) method to detect HIV-1 integrated DNA was used, with a DNA pre-amplification using *Alu* and LTR-specific primers. Proviral DNA levels were determined by a second round SYBR Green-based qPCR assay in reference to a standard curve.

**Results:**

In total, 28 Hypers (6%) and 354 concordants (79%) were identified. Hypers had a higher proportion of CD4+ naïve T-cells (37.6 vs. 24.8, p<0.05), and a low proportion of CD4+ EM T Cells (27.9 vs. 39.4, p<0.05), with similar results found in CD8+ T Cells. Hypers presented a higher percentage of CD4+CD45RA+CD31neg cells. There was no difference in total integrated HIV DNA copies per 10^6^ PBMC (1729 vs. 3062, p=0.19), however the DNA/CD4 ratio of Hypers was significantly lower (1.2 vs. 2.89, p<0.05).

**Conclusions:**

T cell recovery of Hypers occurs very early suggesting cell redistribution, however on the long term, their T CD4+ level is driven by non-thymic-central-naïve cells that are less likely to be HIV infected, thus diluting HIV reservoir. Understanding better immune recovery after HAART and its impact on viral reservoir could contribute to design more effective therapeutic strategies.

# Sub article: TUAA0106LB Dysbiotic bacteria drive suppressive neutrophil phenotypes and prolonged lifespan in mucosal tissues of HIV-infected individuals

**Published:** 2016-07-22

**Authors:** T Hensley-Mcbain, R Cheu, J Manuzak, A Zevin, C Miller, E Lee, C Wilson, A Burgener, N Klatt

**Affiliations:** 1 Department of Pharmaceutics, University of Washington, Seattle, WA, United States; 2 Divisions of Infectious Disease and Clinical Immunology, University of Colorado, Aurora, CO, United States; 3 NLHI, Public Health Agency of Canada, Department of Medical Microbiology and Infectious Diseases, University of Manitoba, Winnipeg, Canada

## Abstract

**Introduction:**

Neutrophils infiltrate the gastrointestinal (GI) tract during HIV infection, yet their contribution to the pathology of mucosal dysfunction is unknown. In chronic HIV, blood neutrophils expressing high levels of PD-L1 suppress T cell function and correlate with T cell expression of PD-1, an exhaustion marker predictive of HIV disease progression.

**Methods:**

Our study aimed to investigate whether suppressive neutrophils are also present in the colon in HIV and examined whether bacterial dysbiosis contributed to their induction. Whole blood and isolated colon biopsy leukocytes from 10 HIV-infected individuals were phenotyped by flow cytometry. To examine the effects of bacterial dysbiosis, whole blood was stimulated for 20 hours with HIV-altered mucosal bacteria prior to phenotyping, including *Prevotella copri*, *Prevotella stercorea*, *Ruminicoccus bromii* and *Lactobacillus plantarum*.

**Results:**

We found a higher frequency of PD-L1 high neutrophils in the colon compared to blood in HIV-infected individuals (p=0.0028). In addition, colon PD-L1 high neutrophils correlated with colon PD-1+ CD4+ T cells (p=0.0207). Incubation of cells with GI bacteria increased in HIV (*Prevotella* spp.), induced this PD-L1 high phenotype in neutrophils. Conversely, the beneficial GI bacteria decreased in HIV, *R. bromii* and *Lactobacillus* did not affect PD-L1 expression. Neutrophil PD-L1 expression correlated with PD-1 expression on CD4+ T cells after bacterial stimulation (p=0.0065). Finally, stimulation with *Prevotella* species reduced neutrophil apoptosis compared to the media control.

**Conclusions:**

These data suggest a role for dysbiotic bacteria in reducing neutrophil homeostatic cell death and clearance and contributing to gut neutrophil infiltration in HIV. Together, these suggests that suppressive colon neutrophils may play a role in T cell exhaustion and mucosal dysfunction associated with bacterial dysbiosis and translocation in HIV, and the continual presence of neutrophils in GI tissues may be a consequence of reduced homeostatic apoptosis upon interaction with these bacteria.

# Sub article: TUAB0101 Resilience in perinatally HIV-infected and perinatally HIV-exposed adolescents and young adults growing up in high-risk environments

**Published:** 2016-07-22

**Authors:** CA Mellins, EJ Abrams, C Dolezal, P Warne, K Elkington, A Bucek, CS Leu

**Affiliations:** 1 New York State Psychiatric Institute, Columbia University, New York, NY, United States; 2 ICAP, Mailman School of Public Health, Columbia University, New York, NY, United States; 3 College of Physicians & Surgeons, Columbia University, New York, NY, United States

## Abstract

**Introduction:**

Globally, paediatric HIV is increasingly an adolescent and young adult (AYA) epidemic. Research with perinatally HIV-infected (PHIV+) AYA has prioritized identification of poor health and behavioural risk outcomes, but understanding positive outcomes in spite of adversity is critical to informing evidence-based programmes. Using data from a New York City longitudinal cohort study (CASAH) of PHIV+ and perinatally HIV-exposed, uninfected (PHIV−) youth, we examined psychosocial and health outcomes pertinent to understanding resilience.

**Methods:**

Data are from the most recent CASAH follow-up interview (2014–2015) with 135 PHIV+ and 86 PHIV− AYA to date. Participants were recruited when aged 9–16 years (2003–2008). Psychosocial batteries are administered every 12–18 months; PHIV+ youth viral load (VL) and CD4 are abstracted from medical records. Data on psychiatric disorders, sexual behaviour, substance use disorders (SUD) and young adult milestones were compared across HIV status and age groups. Descriptive statistics, and chi-square and t-tests for comparing groups were used.

**Results:**

Most participants were female (55%), African-American (67%), living in impoverished communities (100%); mean age was 22 years (range 15–28). There were no HIV-status differences in rates of psychiatric disorder (28%), SUD (27%), or past 3-month condomless sex (36%). At this wave, only 29% of PHIV+AYA had a psychiatric disorder and 25% SUD. Most PHIV+ AYA aged ≥19 years had achieved young adult milestones: 78% had graduated high school, 29% taken college classes; 53% were currently working or in school; 86% had ever had sex; and 41% were in romantic relationships. Achieving milestones did not differ by HIV status. Among all PHIV+ AYA, most had positive health outcomes: CD4≥250 cells/mm^3^ (79%); CD4≥500 cells/mm^3^ (44%) and VL≤1000 copies/ml (70%); 46% had VL<50 copies/ml. Older age was associated with CD4 <250 cells/mm^3^ (*X* ^2^=7.01, df=2, p=0.030) and having a psychiatric disorder was associated with VL>1000 copies/ml (*X* ^2^=4.29, df=1, p=0.038).

**Conclusions:**

In one of the few ongoing US-based studies with this population, we found, despite significant biopsychosocial risks, many PHIV+ AYA have positive health and mental health outcomes and achieve AYA milestones comparable to PHIV- and other vulnerable AYA. Identification of protective factors conferring resilience can inform evidence-based practice for millions of PHIV+ youth world-wide.

# Sub article: TUAB0102 The youth treatment bulge in South Africa: increasing numbers, inferior outcomes among adolescents on ART

**Published:** 2016-07-22

**Authors:** M Maskew, J Bor, W MacLeod, S Carmona, G Sherman, MP Fox

**Affiliations:** 1 Department of Internal Medicine, School of Clinical Medicine, Faculty of Health Sciences, University of the Witwatersrand, Johannesburg, South Africa; 2 Department of Global Health, Boston University School of Public Health, Boston, MA, United States; 3 National Health Laboratory Service, Johannesburg, South Africa; 4 National Institute for Communicable Disease, Johannesburg, South Africa; 5 Department of Paediatrics and Child Health, School of Medicine, University of the Witwatersrand, Johannesburg, South Africa; 6 Department of Epidemiology, Boston University School of Public Health, Boston, MA, United States

## Abstract

**Introduction:**

Children perinatally infected with HIV surviving due to paediatric ART are now ageing into adolescence. Yet monitoring adolescent treatment programmes remains difficult as large, well-defined cohorts are rare. We quantify the size adolescent ART population and proportion virologic suppressed using a national patient cohort developed from South Africa's National Health Laboratory Service (NHLS) database.

**Methods:**

Using NHLS data on all public sector viral load tests nationally since 2004, we analyzed information on all patients aged <20 years at test date. We estimated the total number of patients accessing ART care in a given year as the number of individual patients with viral load results. Data were stratified by age and year (2004–2014) to assess shifts in age distribution on ART over time. We also assessed proportions virally suppressed in 2014, by age.

**Results:**

A total of 929,274 person-years were analyzed. There was a steady increase in number of children on ART under 5 years from 2004 to 2011, after which numbers stabilized, likely due to prevention of mother-to-child transmission (PMTCT) successes. There were large increases in numbers of adolescents on ART, rising 10- to 20-fold from 2004–2007 to 2012–2014 (Table 1). Further increases are expected in 15- to 19-year-olds for the next decade, after which the younger cohort ageing into adolescence will decline. In 2014, the proportion virally suppressed was 71% among 5–9 years (95% confidence interval (CI): 71–72%), 65% among 10–14 years (95% CI: 65–66%) and 61% among 15–19 years (95% CI: 60–61%).

**Conclusions:**

The rollout of PMTCT and paediatric ART led to a demographic bulge of HIV-infected adolescents and subsequently large numbers of adolescents receiving ART. Declining viral suppression among older adolescence suggests an urgent need to improve care for this vulnerable and growing population. Laboratory datasets represent an important tool for national and local resource planning and allocation.

# Sub article: TUAB0103 Long-term trends in mortality and AIDS-defining events among perinatally HIV-infected children across Europe and Thailand

**Published:** 2016-07-22

**Authors:** A Judd, E Chappell, K Doerholt, L Galli, C Giaquinto, D Gibb, T Goetghebuer, S Le Coeur, A Noguera Julian, A Turkova, R Goodall, European Pregnancy and Paediatric Hiv Cohort Collaboration (EPPICC)

**Affiliations:** 1 MRC Clinical Trials Unit, University College London (UCL), London, United Kingdom; 2 St George's Hospital NHS Trust, London, United Kingdom; 3 University of Florence, Florence, Italy; 4 PENTA Foundation, Padova, Italy; 5 Hopital St Pierre, Brussels, Belgium; 6 IRD 174 - PHPT, Chiang Mai, Thailand; 7 Hospital Sant Joan de Deu Barcelona, Barcelona, Spain

## Abstract

**Introduction:**

There are limited data on the prognostic effects of time-updated covariates on long-term mortality rates of perinatally HIV-infected children after starting ART. We analyzed individual patient data from 19 cohorts in 16 European countries and Thailand in EPPICC.

**Methods:**

Perinatally HIV-infected children aged <18 starting cART were followed until death, loss to follow-up (LTFU), transfer to adult care, their 21st birthday or last visit to 31/12/2013. Crude rates of death and first AIDS-defining events were calculated. Baseline and time-updated risk factors for death ≤/>6 months of cART and progression to AIDS were assessed using inverse-probability-censoring-weighted Cox models to account for informative censoring of LTFU.

**Results:**

Of 3527 children, 32, 20, 18 and 30% were from the UK/Ireland, Thailand, Russia/Ukraine and the rest of Europe, respectively. At cART initiation, median (IQR) age was 5.2 (1.4–9.3) years, and 42% had severe WHO immunological stage. Median follow-up was 5.6 (2.9–8.7) years. There were 94 deaths and 174 first AIDS-defining events, of which 43 (46%) and 79 (45%) occurred within 6 months of cART initiation. The crude mortality rate was 2.50 (95% confidence interval (CI): 1.86–3.38)/100 person-years (PY) in the ≤6 month period, and 0.27 (0.21–0.36) thereafter. In total, 59 (63%) {31 ≤6 months} deaths were from HIV-related infections, 19 (20%) {9} were HIV-related non-infectious conditions, 12 (13%) {1} were HIV-unrelated and 4 (4%) {2} were unknown. The rate of first AIDS-defining event was 0.88 (0.76–1.02)/100PY, including 31 (18%) HIV encephalopathy, 29 (17%) tuberculosis and 25 (14%) HIV wasting syndrome. The Table shows multivariable predictors of increased risk of death >6 months of cART. Predictors for death ≤6 months (baseline only) and progression to AIDS (baseline and time-updated) were broadly similar.

**Conclusions:**

Almost half of deaths occurred ≤6 months of cART, after which current severe WHO immune stage, low BMI-for-age z-score and fewer VL copy-years suppressed were the strongest predictors for mortality. The raised mortality risk in those aged ≥14 and in middle-income countries raises concern.

**Abstract TUAB0103–Table 1:** Predictors of death >6 months of cART

Variable		Adjusted HR (95% CI)	P
Country type	Middle-income (Russia, Ukraine, Thailand)	ref	0.028
	High-income	0.5 (0.2–0.9)	
Calendar year at cART start	1997–<2004	ref	0.035
	2004–<2008	0.4 (0.2–0.8)	
	≥2008	0.5 (0.1–1.5)	
BMI-for-age z-score at cART start	>0	0.2 (0.1–0.6)	0.045
	−3 to 0	ref	
	<−3	0.5 (0.2–1.6)	
VL copy-years suppressed (≤400 c/ml) since cART Initiation (per year increase)		0.7 (0.6–0.9)	0.001
Current (time updated) age (years)	<2	4.2 (1.4–12.7)	0.002
	2–<5	0.2 (0.1–1.8)	
	5–<l4	ref	
	≥14	2.1 (1.0–4.2)	
Current (time updated) WHO immune stage severe	No	0.1 (0.1–0.2)	<0.001
	Yes	ref	
Current (time updated) BMl-for-age z-score	>0	1.1 (0.4–2.8)	<0.001
	−3 to 0	ref	
	<−3	19.5 (7.2–52.8)	

# Sub article: TUAB0104 What does adolescent transition mean in sub-Saharan Africa? Predictors of transfer in Southern African perinatally HIV-infected adolescents

**Published:** 2016-07-22

**Authors:** M-A Davies, S Sawry, S Phiri, H Rabie, B Eley, G Fatti, K-G Technau, F Tanser, R Wood, J Giddy, C Bolton-Moore, C Chimbetete, R Hazra, O Keiser, K Stinson

**Affiliations:** 1 School of Public Health and Family Medicine, University of Cape Town, Cape Town, South Africa; 2 Wits Reproductive Health and HIV Institute, University of Witwatersrand, Johannesburg, South Africa; 3 Harriet Shezi Children's Clinic, Johannesburg, South Africa; 4 Lighthouse Trust Clinic, Lilongwe, Malawi; 5 Tygerberg Academic Hospital, University of Stellenbosch, Stellenbosch, South Africa; 6 Red Cross Children's Hospital, University of Cape Town, Cape Town, South Africa; 7 Department of Paediatrics, University of Cape Town, Cape Town, South Africa; 8 Kheth'Impilo, Cape Town, South Africa; 9 Empilweni Services and Research Unit, Rahima Moosa Mother and Child Hospital, University of Witwatersrand, Johannesburg, South Africa; 10 Hlabisa ART Programme, Africa Centre for Health and Population Studies, University of KwaZulu Natal, Somkhele, South Africa; 11 Gugulethu Community Health Centre, University of Cape Town, Cape Town, South Africa; 12 Desmond Tutu HIV Centre, Institute of Infectious Diseases and Molecular Medicine, University of Cape Town, Cape Town, South Africa; 13 McCord Hospital, Durban, South Africa; 14 Centre for Infectious Disease Research in Zambia, Lusaka, Zambia; 15 Newlands Clinic, Harare, Zimbabwe; 16 Maternal and Pediatric Infectious Disease Branch, Eunice Kennedy Shriver National Institute of Child Health and Human Development (NICHD), Bethesda, MD, United States; 17 Institute of Social & Preventive Medicine (ISPM), University of Bern, Bern, Switzerland; 18 Khayelitsha ART Programme, Médecins Sans Frontières, Khayelitsha, South Africa

## Abstract

**Introduction:**

In wealthy countries, many perinatally HIV-infected adolescents (PHA) transition from specialist paediatric clinics to adolescent/adult clinics during late adolescence. Transition may differ in sub-Saharan Africa, where paediatric HIV care is mostly provided in decentralized non-specialist primary care clinics either from antiretroviral therapy (ART) start or soon thereafter once children are stable on treatment. We examined transfer patterns in PHA in Southern Africa.

**Methods:**

We included presumed PHA (ART initiation at <9.5 years old without documented non-perinatal infection) with follow-up after 10 years of age at 12 IeDEA-SA cohorts providing paediatric ART care from Malawi, South Africa, Zambia and Zimbabwe from 2000 to 2014. We described characteristics at ART initiation and at transfer or last visit in those remaining in care (RIC) at their original site. We used Cox proportional hazards models to identify predictors of transfer.

**Results:**

We excluded 1660 PHA from two cohorts, where no children transferred. Among 3820 children included, estimated probability of transfer by age 13 years varied widely between sites from 5.1 to 54.3%. Transfer was higher from specialist paediatric facilities compared to primary care facilities. At transfer, the median age was 11.4 years; 82% of children had CD4 >500 cells/µl and 89% had HIV-RNA <400 copies/ml (Table 1). After adjusting for site, PHA with the following characteristics were more likely to transfer: longer ART duration at 10 years (adjusted hazard ratio (aHR): 1.29, 95% confidence interval (CI): 1.22–1.35), not severely immunodeficient at ART start (aHR: 1.25; 95% CI: 1.03–1.52), CD4 >500 cells/µl at age 10 (aHR: 1.30; 95% CI: 1.01–1.6) and HIV-RNA <400 copies/ml at age 10 (aHR: 1.38; 95% CI: 1.05–1.82).

**Conclusions:**

Transfer patterns differ considerably between cohorts with many children transferring during early adolescence. PHA were relatively well at transfer; more than 80% had CD4 >500 cells/µl and virologic control. Understanding transfer patterns and tracking outcomes after transfer are important to comprehensively evaluate PHA outcomes.

**Abstract TUAB0104–Table 1:** Characteristics of children with presumed perinatal HIV infection who remain in care at the original site (RIC) or are transferred out (TFO)

	RIC (n=2650) (excludes 253 children deceased or LTFU)	TFO (n=917)	p
Female (n/N; %)	1260/2650; 48%	439/917; 48%	0.865
Median (IQR) age in years at ART start	7.2 (5.6–8.4)	7.1 (5.4–8.3)	0.195
WHO-defined severe immunosuppression at ART start (n/N; %)	993/1461; 68%	433/627; 69%	0.623
Median (IQR) age (years) at TFO or last visit if RIC	12.1 (10.9–13.8)	11.4 (10.6–12.7)	<0.001
Median (IQR) CD4 (cells/µl) at TFO or last visit if RIC	725 (518–950)	779 (569–1032)	<0.001
CD4 >500 cells/µl at TFO or last visit if RIC (n/N; %)	1566/2036; 77%	656/801; 82%	0.004
Height-for-age z-score <–2 at TFO or last visit if RIC (n/N; %)	355/906; 39%	221/543; 41%	0.568
HIV-RNA <400 copies/ml (n/N; %)	1543/2117; 73%	694/781; 89%	<0.001

# Sub article: TUAB0105LB Switching to second-line antiretroviral therapy in HIV-infected children: a CIPHER cohort collaboration global analysis

**Published:** 2016-07-22

**Authors:** IJ Collins, Collaborative Initiative for Paediatric HIV Education and Research (CIPHER) Cohort Collaboration Duration of First-line Team

**Affiliations:** MRC Clinical Trials Unit at UCL, Institute of Clinical Trials & Methodology, London, United Kingdom

## Abstract

**Introduction:**

There are conflicting data on time to switch from first-line to second-line antiretroviral therapy (ART) in children. Here we present the first global estimates.

**Methods:**

Individual-level data were pooled from 12 cohort networks within CIPHER. Children aged <18-years initiating combination ART (≥2 nucleoside reverse-transcriptase inhibitors (NRTI) plus non-NRTI (NNRTI) or boosted protease inhibitor (PI)) were included. Switch to second-line was defined as: (i) change of ≥1 NRTI plus either change in drug class (NNRTI to PI or vice versa) or PI change; (ii) change from single to dual PI; or (iii) addition of new drug class. Cumulative incidence curves assessed time to switch, with death and loss to follow-up (LTFU) as competing risks.

**Results:**

Of 95,194 children included, 18% were from South Africa and 72% from rest of sub-Saharan Africa (SSA). At ART start, median [IQR] age was 3.7 [1.6–6.8] years, CD4% 15% [9–21%], 42% had AIDS, 89 and 11% initiated NNRTI- and PI-based ART, respectively. Median duration of follow-up from ART initiation was 26 [9–51] months; 1% died, 26% were LTFU and 20% transferred out. Overall 4266 (4.5%) switched to second-line at median of 33.8 [18.5, 55.1] months. The proportion switching at 3 years after ART start varied significantly across regions from 1.6% (95% CI: 1.5, 1.7) in SSA to 26.8% (20.6, 33.3) in North America (Figure). A higher incidence of switch was seen in children aged ≥10 years at ART start compared to younger children in all regions except North America, in settings with routine viral load (VL) monitoring, and in children initiating NNRTI-based ART compared to PI-based ART in all regions except SSA.

**Conclusions:**

We found wide regional variations in the cumulative incidence of switch to second-line, with higher incidence among children initiating ART aged ≥10 years and those in settings with routine VL monitoring. High rates of transfer and LTFU mean these estimates maybe the lower bound of the true switch rates.

**Abstract TUAB0105LB–Figure 1:** Cumulative incidence of switch at 3 years of ART by age at start of ART, initial regimen and monitoring strategy by region.

# Sub article: TUAB0201 Cash, care and HIV community: social protection improves adolescent ART adherence in South Africa

**Published:** 2016-07-22

**Authors:** L Cluver, E Toska, M Orkin, A Yakubovich, R Hodes, L Sherr

**Affiliations:** 1 University of Cape Town, Cape Town, South Africa; 2 Department of Social Policy and Intervention, University of Oxford, Oxford, United Kingdom; 3 DST-NRF Centre of Excellence in Human Development, University of the Witwatersrand, Johannesburg, South Africa; 4 University College London, London, United Kingdom

## Abstract

**Introduction:**

Low antiretroviral therapy (ART) adherence amongst adolescents causes morbidity, mortality and onwards HIV transmission. Reviews find no effective adherence-promoting interventions. This study examines associations of seven potential social protection factors with adherence, in the world's largest community sample of HIV-positive adolescents.

**Methods:**

N=1059 adolescents: all 10- to 19-year-olds ever ART-initiated in 53 government healthcare facilities in a health district of South Africa's Eastern Cape were traced and interviewed in 2014–15. 90.1% of the eligible sample was included (4.1% adolescent or caregiver refused, 0.9% had severe cognitive disability, 1.2% excluded and 3.7% unable to trace). Potential social protection predictors were “cash”: food security, school fees/materials, clothing; and “care”: HIV support group, sports group, positive parenting and high parental supervision. Analyses used multivariate regression with all potential predictors entered simultaneously, and interaction and marginal effects models in SPSS and STATA.

**Results:**

Past-week self-reported ART non-adherence was 36%, associated with increased opportunistic infections (p<0.002, SD=0.09). Postnatally infected and rural adolescents were at highest risk; age and gender did not predict adherence. Analyses controlled for covariates: age, gender, location, perinatal/postnatal infection, treatment duration, ethnicity, maternal/paternal death, distance to clinic and general health. Independent of these, three cash and care social protection factors were associated with reduced non-adherence: food security (2 meals/day) (OR=0.60, CI: 0.44–0.81, p<0.001); high parental/caregiver supervision (i.e. monitoring of adolescent activities) (OR=0.62, CI: 0.47–0.82, p<0.001); and attending an HIV support group (OR=0.54, CI: 0.36–0.83, p<0.004). Effects of combination social protection were not multiplicative but were additive in predicted probabilities controlling for co-factors. With no protection factors, non-adherence was 52%, with any one protection it was 37–40%, and with all three social protection, it was 18%.

**Conclusions:**

Combination social protection, “cash plus care,” improves adolescent ART adherence. Specifically, food security, parenting support programmes and expanded provision of HIV support groups have potential to improve adherence, and subsequently adolescent HIV survival and HIV prevention.

**Abstract TUAB0201–Figure 1:** Distribution of individual viral load test results by age and period.

# Sub article: TUAB0202 The effect of community ART groups on retention-in-care among patients on ART in Tete Province, Mozambique

**Published:** 2016-07-22

**Authors:** T Decroo, B Telfer, C Das Dores, B Candrinho, N Dos Santos, A Mkwamba, S Dezembro, M Jofrisse, T Ellman, C Metcalf

**Affiliations:** 1 Médecins Sans Frontières, Tete, Mozambique; 2 Direcção Provincial de Saúde, Tete, Mozambique; 3 Southern Africa Medical Unit, Médecins Sans Frontières, Cape Town, South Africa

## Abstract

**Introduction:**

Antiretroviral therapy (ART) programmes in many African countries have high attrition rates (death or loss-to-follow-up (LTFU) combined). In 2008, patients on ART in Tete Province, Mozambique, began forming community ART groups (CAGs) to overcome barriers to retention-in-care (RIC). CAGs are peer groups in which members take turns to collect ART at the health facility. Patients on ART can either join a CAG or remain in clinic-based care. We conducted a retrospective cohort study among adult patients on ART to quantify the effect of CAG versus individual care on RIC.

**Methods:**

Information until May 2012 was collected from patient records at eight health facilities. Patients who started ART ≥6 months before CAGs started at a health facility, or aged <15 years at ART initiation, were excluded. Furthermore, patients had to remain in care for at least 6 months after starting ART to be included in the analysis. Survival analysis was used to compare RIC among patients in CAGs and patients in individual care, with time to joining a CAG treated as an irreversible time-dependent covariate. Cox regression was used to determine hazard ratios (aHR) for attrition, adjusted for age, gender and health facility.

**Results:**

Of the 2683 patients in the analysis, 62.6% were female. The median age was 32 years. 12- and 24-month RIC from point of eligibility were, respectively, 99.3% (95% CI: 97.8%–99.8%) and 96.3% (95% CI: 94.4%–97.6%) among patients in CAGs, and 89.0% (95% CI: 87.3%–90.2%) and 81.3% (95% CI: 78.8%–83.4%) among those in individual care (p<0.001). CAG patients were more than four times less at risk to die or to be LTFU (aHR=0.22; 95% CI: 0.15–0.32; p<0.001).

**Conclusions:**

RIC was substantially better among patients on ART in CAGs than those in individual care. While exclusion of the first 6 months on ART reduced the potential impact of survivor bias, residual confounders may contribute to the differences observed. Nevertheless, this study confirms that patient-led ART distribution through CAG results in high RIC and supports the Mozambique Ministry of Health decision to implement CAG nationally.

# Sub article: TUAB0203 One year retention in community versus clinic-based adherence clubs for stable ART patients in South Africa: findings from a randomized controlled trial

**Published:** 2016-07-22

**Authors:** C Hanrahan, V Keyser, S Schwartz, P Soyizwaphi, N West, L Mutunga, J Steingo, J Bassett, A Van Rie

**Affiliations:** 1 Department of Epidemiology, Johns Hopkins Bloomberg School of Public Health, Baltimore, MD, United States; 2 Witkoppen Health and Welfare Centre, Johannesburg, South Africa; 3 University of Antwerp, Antwerp, Belgium; 4 School of Global Public Health, University of North Carolina Gillings, Chapel Hill, NC, United States

## Abstract

**Introduction:**

Adherence clubs, where groups of 25–30 patients stable on antiretroviral therapy (ART) meet for counselling and medication pick-up, are an innovative model to retain patients in care and facilitate task-shifting. Adherence clubs can be organized at a clinic or community venue. We performed a randomized controlled trial to compare club retention between community and clinic-based adherence clubs.

**Methods:**

Stable patients with undetectable viral load at Witkoppen Health and Welfare Centre, in Johannesburg, South Africa, were randomized to either a clinic- or community-based adherence club. Clubs were held every other month and were run by an HIV counsellor. All club participants received annual viral load monitoring and annual medical exam by a clinician at the clinic. Patients became ineligible for club participation and were referred back to routine care if they missed a club visit without ART pickup within 5 days, had two consecutive late ART pickups, developed a comorbidity requiring closer monitoring or had viral rebound. We compared the proportion referred back to routine care between clinic and community-based clubs in the first 12 months of club participation.

**Results:**

From February 2014–May 2015, we randomized 770 adults into 12 pairs of clubs – 378 (49%) clinic-based and 392 (51%) community-based. Characteristics were similar by arm: 66% female, 88% on fixed-dose combination ART and median CD4 count of 502 cells/mm^3^. The proportion referred back to routine care was greater among community-based clubs (26%, n=102) compared to clinic-based clubs (19%, n=70, p=0.012) (Figure 1). Viral rebound was uncommon and comparable by club type (3% in clinic and 2% in community, p=0.594). Among those referred back to routine care, missing a club visit was the most common reason in both club types (61%).

**Conclusions:**

Within the first year of adherence club participation, drop out was higher among community-based compared to clinic-based clubs.

**Figure 1:** Retention in adherence club care – community vs clinic-based clubs.

# Sub article: TUAB0204 Improved adherence to antiretroviral treatment observed among children whose caregivers had positive beliefs in medicine

**Published:** 2016-07-22

**Authors:** G Abongomera, A Cook, M Lamorde, C Chabala, V Musiime, M Thomason, V Mulenga, R Colebunders, C Kityo, S Walker, DM Gibb, on behalf of the CHAPAS-3 Trial Team

**Affiliations:** 1 Joint Clinical Research Centre, Kampala, Uganda; 2 University of Antwerp, Antwerp, Belgium; 3 Clinical Trials Unit, Medical Research Council, London, United Kingdom; 4 Infectious Diseases Institute, Kampala, Uganda; 5 University Teaching Hospital, Lusaka, Zambia

## Abstract

**Introduction:**

CHAPAS-3 trial investigated how the views of the child's caregiver towards medicine affected the adherence of their child to fixed-dose combination antiretroviral therapy (ART).

**Methods:**

A total 478 HIV-infected children aged 1 month to 13 years were randomized to one of three first-line ART regimens in Uganda and Zambia. Children were ART naïve (n=365) or ART experienced (n=113) at enrolment. We measured adherence to ART using medication event monitoring systems (MEMS) caps and caregivers' views towards all medicines and medicines currently prescribed using the Beliefs in Medicine Questionnaire (BMQ). MEMS caps data were collected during weeks 0–18 and 54–72. The BMQ was completed by caregivers at weeks 0, 6, 24, 48, 72 and 96. We used repeated measures linear regression models to investigate associations between MEMS adherence in weeks 0–18 and BMQ at weeks 0 and 6 (period 1), and MEMS adherence in weeks 54–72 and BMQ in week 48 (period 2).

**Results:**

MEMS adherence and BMQ data were available from 271/365 (74%) ART-naïve and 97/113 (86%) ART-experienced children in period 1, and 235/335 (70%) naïve and 98/112 (88%) experienced children in period 2. We present results from the ART-naïve group in period 1, similar results were observed in period 2, and also among ART-experienced children. Caregivers belief in the necessity of ART was stronger on average than their concern; median (IQR) scores were 20.0 (19.3,21.7) and 12.0 (10.7,14.7) for necessity and concern, respectively. The median (IQR) necessity-concern differential was 8.3 (6.7,9.7). Adherence was good, as measured by MEMS, with median (IQR) 92% (84%, 96%) doses taken. A significant positive association was observed between high necessity-concern score and high MEMS adherence, p=0.028 (β=0.236). A significant association was also seen among naïve children in period 2 (p<0.001) but not among ART-experienced children.

**Conclusions:**

Caregivers of HIV-infected children had a strong belief in the necessity of ART, outweighing their concerns about treatment. High levels of adherence to ART were associated with positive overall beliefs towards medicine. There is a need of emphasizing the necessity of treatment to caregivers, while addressing any concerns they may have about ART.

# Sub article: TUAB0205 Is retention on ART underestimated due to patient transfers? Estimating system-wide retention using a national labs database in South Africa

**Published:** 2016-07-22

**Authors:** M Fox, J Bor, W MacLeod, M Maskew, A Brennan, W Stevens, S Carmona

**Affiliations:** 1 Departments of Epidemiology and Global Health, School of Public Health, Boston University, Boston, MA, United States; 2 Health Economics and Epidemiology Research Office, University of the Witwatersrand, Johannesburg, South Africa; 3 Department of Global Health, School of Public Health, Boston University, Boston, MA, United States; 4 National Health Laboratory Service, Johannesburg, South Africa

## Abstract

**Introduction:**

Systematic reviews have described high rates of attrition in patients receiving antiretroviral therapy (ART). However, migration and clinical transfer may lead to overestimation of attrition. Using a newly linked national laboratory database in South Africa, we assessed system-wide retention in care.

**Methods:**

South Africa's National Health Laboratory Service maintains a database of all public sector CD4 count and viral load (VL) test results since 2004. We developed an algorithm to link individual lab results using probabilistic matching techniques, creating a national cohort of HIV patients. We analyzed data on all patients initiating ART in 2004 and 2005 (during which time VL were collected at ART initiation) and who had at least one subsequent lab result. We assessed retention in care as *time to a patient's most recent lab result* (CD4 or VL), following patients through March 2015. Patients were identified as still in care if their last lab test occurred April 2013–March 2015. We assessed two retention concepts: (a) *system-wide retention* including all lab results regardless of testing facility and (b) *retention at the initiating clinic*, in which lab tests at other facilities were ignored. These two concepts mirror the information available on patient histories from clinic-based and health system-wide perspectives.

**Results:**

We followed 53,880 patients who initiated ART in 2004 and 2005. Eight-year retention at the initiating clinic was 13.1% (95% CI: 12.9–13.4). After allowing for transfers, system-wide eight-year retention increased to 47.3% (95% CI: 46.9–47.7) (Figure 1).

**Conclusions:**

Patient migration and transfer are common throughout sub-Saharan Africa. Although prior cohort studies have tracked patients through resource-intensive follow-up, we show the utility of a national laboratory database for passive tracking of patients regardless of where they seek care. These findings have implications not just for measurement but also potential to improve continuity of patient care in migration populations.

**Figure 1:** Effect of patient transfer on retention estimates.

# Sub article: TUAC0101 Oral administration of maraviroc, in infant rhesus macaques, fails to prevent SIVmac oral transmission

**Published:** 2016-07-22

**Authors:** E Brocca-Cofano, C Xu, D Ma, BB Policicchio, KD Raehtz, T Dunsmore, GS Haret-Richter, C Sykes, BF Keele, ADM Kashuba, I Pandrea, C Apetrei

**Affiliations:** 1 Center for Vaccine Research, University of Pittsburgh, Pittsburgh, PA, United States; 2 Department of Pathology, University of Pittsburgh, Pittsburgh, PA, United States; 3 Department of Microbiology and Molecular Genetics, University of Pittsburgh, Pittsburgh, PA, United States; 4 Microbiology and Infectious Diseases, Graduate School of Public Health, University of Pittsburgh, Pittsburgh, PA, United States; 5 Eshelman School of Pharmacy, Chapel Hill, NC, United States; 6 National Cancer Institute, Frederick, MD, United States; 7 School of Medicine, University of North Carolina, Chapel Hill, NC, United States

## Abstract

**Introduction:**

HIV maternal-to-infant-transmission (MTIT) accounts for >300,000 cases annually. New strategies of prevention are needed. SIV target cell availability at mucosal sites was reported to drive virus transmission. We investigated if systemic CCR5 blockade with Maraviroc (MVC) impacts oral SIV transmission to infant rhesus macaques (RMs).

**Methods:**

Nine infant RMs aged 6 months were included. Four RMs were untreated controls and five RMs received MVC (150 mg/kg/bid/orally) for up to 6 months. Co-receptor occupancy was closely monitored, and RMs were orally exposed to 10,000 TCID50 of SIVmac766XII every 2 weeks, up to 6 times. The concentration of MVC in plasma was measured by validated LC-MS/MS, plasma viral loads (VLs) and changes of immune cells were monitored respectively by RT-PCR and flow cytometry.

**Results:**

MVC was well tolerated by RMs, with no adverse reactions and significantly blocked CCR5 co-receptor compared to control group (I challenge p=0.0159, II challenge p=0.0317 and III challenge p=0.0286). All RMs in the control group and 60% of those receiving MVC became infected (p=0.1515). No difference in the number of exposures needed to infect RMs in the two groups was observed. At the time of viral exposure, MVC plasma concentrations were of 538.36±422.56 ng/ml, within the range seen in humans receiving MVC. All treated and control RMs were infected with one viral variant, suggesting that the animals were not overexposed to virus, which might have offset MVC protective effect. Ramp-up viremia was significantly delayed (p=0.05) in the MVC-treated RMs. Peak (MVC-treated 7.18 log; control 6.71 log) and post-peak (MVC-treated 5.49 log; control 5.29 logs) VLs were similar in both groups. No significant differences in CD4^+^ T cell depletion or in the levels of immune activation were observed between the two groups.

**Conclusions:**

MVC effectively blocked CCR5 and was well tolerated in infant RMs. Yet, CCR5 blockade with MVC did not significantly impact SIV oral transmission. Since SIVmac is more promiscuous than HIV-1 with regard to co-receptor usage (i.e. being able to use alternative co-receptors, such as BOB/GPR15 and Bonzo/STRL33), CCR5 blockade in humans might be more effective in preventing MTIT alone or in combination with other antiretroviral drugs.

# Sub article: TUAC0102 HPTN 069/ACTG A5305: phase II study of maraviroc-containing regimens for HIV PrEP in US women

**Published:** 2016-07-22

**Authors:** R Gulick, T Wilkin, Y Chen, R Landovitz, R Amico, A Young, P Richardson, M Marzinke, C Hendrix, S Eshleman, I McGowan, A Andrade, S Hodder, K Klingman, W Chege, A Rinehart, J Rooney, P Andrew, M McCauley, K Mayer, HPTN 069/ACTG 5305 Study Team

**Affiliations:** 1 Weill Cornell Medicine, New York, NY, United States; 2 Fred Hutchinson Cancer Research Center, Seattle, WA, United States; 3 University of California, Los Angeles, CA, United States; 4 School of Public Health, University of Michigan, Ann Arbor, MI, United States; 5 School of Medicine, Johns Hopkins University, Baltimore, MD, United States; 6 Hepatology & Nutrition, School of Medicine, University of Pittsburgh, Pittsburgh, PA, United States; 7 West Virginia University School of Medicine, Morgantown, WV, United States; 8 National Institute of Allergy and Infectious Diseases, Bethesda, MD, United States; 9 ViiV Healthcare, Durham, NC, United States; 10 Gilead Sciences, Foster City, CA, United States; 11 FHI 360, Durham, NC, United States; 12 FHI 360, Washington, DC, United States; 13 The Fenway Institute, Boston, MA, United States

## Abstract

**Introduction:**

Maraviroc (MVC) is an HIV entry inhibitor that concentrates in the genital tract/rectum, making it a potential pre-exposure prophylaxis (PrEP) agent.

**Methods:**

Prospective, randomized, double-blinded, multisite, safety/tolerability study of 4 regimens for HIV PrEP: (1) MVC alone; (2) MVC+ emtricitabine (FTC); (3) MVC+ tenofovir (TDF); (4) TDF+FTC. Study regimens consisted of three pills once-daily – MVC 300 mg, FTC 200 mg, TDF 300 mg, with matching placebos. Eligible participants were adult HIV-uninfected women who reported a history of condomless vaginal or anal intercourse with ≥1 HIV-infected or unknown-serostatus man within 90 days of screening, and had adequate safety laboratory parameters including calculated creatinine clearance ≥70 ml/min. Participants were randomized to study regimens for 48 weeks with follow-up visits at weeks 2, 4, 8, and then every 8 weeks. At each visit, history, physical exam, safety laboratories, blood plasma for drug concentrations, adherence counselling and HIV testing, were conducted. All analyzes were intent-to-treat.

**Results:**

12 HPTN and ACTG sites enrolled 188 women with a median age of 35 (range 18–61), including 65% black, 27% white, and 17% Latina participants. 153 (81%) completed study follow-up; 15 (8%) were lost to follow-up. 37 (20%) permanently discontinued the study regimen early, including 16 (8%) for participant request and 10 (5%) for pregnancy; rates and times to study drug discontinuation did not differ among the study arms (both p>0.2). MVC-alone was associated with fewer grade 2–4 adverse events than either TDF-containing regimen (p<0.01); MVC+FTC was associated with fewer events than MVC+TDF (p=0.02). In a random subset of participants (n=125) at random study time points, 66% had detectable study drug plasma concentrations. Four women had sexually transmitted infections while on study (3 chlamydia, 1 gonorrhoea). No HIV infections were identified during the study; the annual HIV incidence in women on this study was 0% (95% CI: 0%, 2.5%).

**Conclusions:**

In this study of HIV PrEP in women, MVC-containing regimens were safe and well-tolerated compared to the control regimen of TDF+FTC. Only 2/3 had detectable study drug concentrations, but no HIV infections were identified. MVC-containing regimens should be explored further as oral PrEP for women.

# Sub article: TUAC0103 Persistence of rilpivirine following single dose of long-acting injection

**Published:** 2016-07-22

**Authors:** I McGowan, A Siegel, J Engstrom, A Nikiforov, K Duffill, S Edick, C Mitchell, D Back, L Else, D Egan, S Khoo, P Williams, RM Brand, RD Cranston

**Affiliations:** 1 University of Pittsburgh, School of Medicine, Pittsburgh, PA, United States; 2 Magee-Womens Research Institute, Pittsburgh, PA, United States; 3 University of Liverpool, Liverpool, United Kingdom; 4 Janssen Pharmaceuticals, Beerse, Belgium

## Abstract

**Introduction:**

Long-acting (LA) injectable formulations of rilpivirine (RPV) and cabotegravir (CAB) are currently being evaluated for the treatment and prevention of HIV infection. It is possible that, following completion of the pre-exposure prophylaxis (PrEP) dosing regimen with an LA agent, participants may experience an extended period of exposure to declining antiretroviral concentrations. Individuals who acquire HIV infection during this period may be at risk of developing resistance to the agent. To mitigate this risk, the HPTN-083 Phase 2B/3 study of CAB LA PrEP is proposing to provide study participants with 12 months of oral PrEP to cover the LA pharmacokinetics (PK) tail. However, there is an urgent need to better define the PK tail for LA PrEP agents.

**Methods:**

The MWRI-01 study was undertaken to characterize the safety, acceptability, PK and pharmacodynamic profile of RPV LA. Participants from the single dose (SD) phase of evaluation of RPV (600 and 1200 mg) were able to enrol in a multiple dose (MD) phase evaluation of RPV (1200 mg). This study design provided an opportunity to characterize the persistence of RPV in baseline plasma samples obtained from participants enrolled in the MD phase of the MWRI-01 study. The Lower Limit of Quantification for RPV was 0.5 ng/ml. Multiple blanks were included in the PK assays to exclude the possibility of carryover contamination.

**Results:**

Eight women and four men were enrolled in the MD phase of the study of whom 9/12 (75%) had participated in the SD phase of the study (Table 1). RPV was detected in baseline plasma samples of all 5 female participants (Mean RPV concentration 4.8±2.9 ng/ml) and 3/4 of the male participants (Mean RPV concentration 2.9±1.6 ng/ml). The mean time interval between the SD and baseline visit was 536±182 and 591±78 days, respectively, for the female and male participants.

**Conclusions:**

SD administration of RPV LA was associated with prolonged and declining PK exposure. These data have significant implications for the design of LA PrEP studies.

**Abstract TUAC0103–Table 1:** Summary of time interval between single dose RPV and baseline PK

Sex	Single Dose (SD) of RPV	Plasma RPV at 24 hours after SD RPV (ng/ml) Mean (±STD)	Time (Days) between SD and Multiple Dose (MD) Baseline Visit Mean (±STD)	Plasma RPV at MD Baseline Visit (ng/ml) Mean (±STD)
Male	600 mg (N=2)	20.9 (12.3)	630 (100)	0.8 (0.8)
	1200 mg (N=2)	18.2 (3.7)	553 (51)	3.7 (1.1)
Female	1200 mg (N=5)	54.0 (12.1)	536 (182)	4.8 (2.9)

# Sub article: TUAC0104 Benefits of pre-exposure prophylaxis relative to drug resistance risk

**Published:** 2016-07-22

**Authors:** R Grant, V Fonner, M Rodolph, T Liegler, D Glidden, R Baggaley

**Affiliations:** 1 Gladstone Institutes, San Francisco, CA, United States; 2 World Health Organization, Geneva, Switzerland; 3 San Francisco AIDS Foundation, San Francisco, CA, United States; 4 Johns Hopkins University, Baltimore, MD, United States; 5 University of California, San Francisco, CA, United States

## Abstract

**Introduction:**

The risk of drug resistance (DR) with FTC/TDF or TDF pre-exposure prophylaxis (PrEP) occurs primarily in people who had acute HIV infection when starting or restarting PrEP. The risks and benefits of RNA testing before starting PrEP have not been determined.

**Methods:**

The risk of DR in PrEP programmes was compiled across 6 randomized clinical trials and one demonstration project. Resistance was measured using clinical genotypic tests.

**Results:**

All reviewed trials used rapid second- or third-generation antibody tests to guide PrEP initiation and retrospectively analyzed baseline specimens for HIV RNA among seroconverters. FTC DR occurred in 10 participants who received FTC/TDF PrEP, including 33% (5/15) of participants acutely infected when starting PrEP and in 3% (5/157) of participants with emergent infection. Including both baseline and emergent infections, there were 172 infections in the FTC/TDF arms compared with 270 among corresponding placebo controls, representing 98 infections averted and 10 (98/10) infections averted for every FTC resistant infection. Tenofovir resistance occurred in one participant who received TDF PrEP, including 10% (1/10) of participants acutely infected when starting PrEP and none (of 90) with emergent infection. There were 100 infections in the TDF arms and 153 infections in the placebo controls, representing 53 infections averted by TDF PrEP and 53 (53/1) infections averted for every tenofovir resistant infection. In the demonstration project, a screen for acute viral symptoms led to deferral of PrEP among 30 of 1603 (1.9%) participants, of whom 2 (6.7%) were subsequently found to be acutely infected with HIV. Overall, the absolute risk of excess DR during FTC/TDF PrEP was 0.05% (5/9222).

**Conclusions:**

This analysis supports recent World Health Organization PrEP implementation guidance suggesting that rapid third-generation antibody tests are sufficient to minimize the overall risk of DR from PrEP. DR risk is higher with FTC/TDF PrEP compared with TDF PrEP. RNA testing before starting PrEP minimizes DR risk further while increasing costs and the risk of HIV infection due to delayed PrEP initiation.

# Sub article: TUAC0105LB Residual dapivirine ring levels indicate higher adherence to vaginal ring is associated with HIV-1 protection

**Published:** 2016-07-22

**Authors:** E Brown, T Palanee-Philips, M Marzinke, C Hendrix, C Dezutti, L Soto-Torres, J Baeten

**Affiliations:** 1 Fred Hutchinson Cancer Research Center, Seattle, WA, United States; 2 University of Washington, Seattle, WA, United States; 3 University of the Witswaterand, Johannesburg, South Africa; 4 Department of Pathology and Medicine, Johns Hopkins University, Baltimore, MD, United States; 5 University of Pittsburgh, Pittsburgh, PA, United States; 6 NIAID, Rockville, MD, United States; 7 Departments of Global Health, Medicine and Epidemiology, University of Washington, Seattle, WA, United States

## Abstract

**Introduction:**

In MTN-020/ASPIRE, a vaginal ring containing dapivirine was found to decrease the risk of HIV-1 acquisition by 27% overall in an intention-to-treat analysis compared to placebo and by 37% in an analysis excluding data from two sites with lower adherence/retention. In subgroup analyses, no HIV-1 protection was seen in women aged ≤21, for whom adherence appeared lower. In studies of tenofovir-based prophylaxis, objective markers of adherence have been important in understanding HIV-1 protection when products are used.

**Methods:**

Rings were manufactured with 25 mg of dapivirine, and phase I studies indicated that ~4 mg of dapivirine on average are released during four weeks of continuous use; therefore, levels ≤22 mg were defined as having higher adherence for the present analysis. Starting one year into the trial, we tested the residual dapivirine levels (RDL) remaining in returned, used rings in ASPIRE. Visits at which participants did not return the ring, did not have access to the ring due to product hold or refusal or had RDL >22 mg, were categorized as less or non-adherent. The association between HIV-1 acquisition and adherence was assessed using time-varying covariate Cox models adjusted for age and study site, including visits occurring at month 12 and beyond.

**Results:**

Of the 2629 women enrolled in ASPIRE, 2359 were included in this analysis. Compared to placebo, higher adherence to the active dapivirine ring (i.e. RDL ≤22 mg) was associated with a 65% (95% CI: 23–84, p=0.009) reduction in HIV-1 risk. Results were similar for the full study population and when excluding the two sites with lower adherence/retention (risk reduction 67%, 95% CI: 23–86), and point estimates suggested HIV-1 protection for both women >21 years (risk reduction 72%, 95% CI: 21–90) and ≤21 years of age (risk reduction 50%, 95% CI: −78–86). Partial/low adherence (i.e. RDL >22 mg) was not significantly associated with HIV-1 protection (relative risk reduction 35%, 95% CI: −10–61, p=0.12).

**Conclusions:**

Residual dapivirine levels in returned rings, an objective marker of adherence, indicate that higher adherence to the dapivirine vaginal ring may provide >65% protection from HIV-1 acquisition.

# Sub article: TUAC0201 Strengthening HIV surveillance in the antiretroviral therapy era: baseline findings of HIV prevalence and incidence from KwaZulu-Natal, South Africa

**Published:** 2016-07-22

**Authors:** A Kharsany, C Cawood, D Khanyile, A Grobler, A Puren, T Kufa-Chakeza, N Samsunder, J Frohlich, Q Abdool Karim, G George, K Govender, C Toledo, Z Chipeta, L Zembe, M Glenshaw, L Madurai, A Bere, V Deyde

**Affiliations:** 1 Centre for the AIDS Programme of Research in South Africa (CAPRISA), Durban, South Africa; 2 Epicentre AIDS Risk Management, Cape Town, South Africa; 3 Centre for HIV and STIs, National Institute for Communicable Diseases, National Health Laboratory Service (NICD/NHLS), Johannesburg, South Africa; 4 Health Economics and HIV/AIDS Research Division (HEARD), University of KwaZulu-Natal, Durban, South Africa; 5 Centres for Disease Control and Prevention (CDC), Atlanta, GA, United States; 6 Centres for Disease Control and Prevention (CDC), Pretoria, South Africa; 7 Global Clinical and Virology Laboratory, Durban, South Africa

## Abstract

**Introduction:**

South Africa has over 6,000,000 persons living with HIV/AIDS, and the province of KwaZulu-Natal (KZN) is severely affected. We report on baseline findings from the population based on household surveys from the Umgungundlovu district of KZN, South Africa.

**Methods:**

A two-stage cluster-based sample of randomly selected EAs and households; enrolled one eligible individual 15–49 years per household (from 2014 to 2015). Structured questionnaires were administered and peripheral blood samples were collected for laboratory measurements. HIV incidence was measured using the HIV LAg-avidity assay, adjusting for ART use and viral load. Taking into account the sampling design and adjusting for non-response, weighted data were analyzed using SAS survey procedures.

**Results:**

Of the 14,624 eligible households visited, 11,299 participated and 9812 individuals were enrolled; 63.8% females and 36.2% males. Overall, HIV prevalence was 36.3% (95% CI: 34.8–37.8); 44.1% (95% CI: 42.3–45.9) females and 28.0% (95% CI: 25.9–30.1) males (risk ratio 1.57, 95% CI: 1.45–1.71, p<0.001). Prevalence was higher in females compared to males: 15–19 years (11.5% vs. 5.0%, p<0.001) and 20–24 years (32.4% vs. 10.1%, p<0.001). Prevalence peaked at 66.4% in females between 35 and 39 years when compared to 59.6% in males between 40 and 44 years. A higher proportion of males had detectable virus (66.1% males vs. 53.4% females, p<0.001); however, 83.3% of females 15–19 years; 81.4% and 89.9% of males 20–24 and 25–29 years, respectively, had detectable virus. Males had a higher median (IQR) log viral load (3.69 c/ml, IQR 0.3–4.66 vs. 1.83 c/ml, IQR 0–4.1; p<0.001), with no difference in males and females 15–19 years (males 3.57 c/ml, IQR 1.3–4.6 vs. females 4.1 c/ml, IQR 2.2–4.5; p=0.451). Overall, HIV incidence was 3.21/100 person-years (PY), 95% CI 2.39–4.03; 4.06/100 py, 95% CI 2.85–5.25 in females and 2.10/100 py, 95% CI 1.13–3.05 in males.

**Conclusions:**

Despite the scale up of HIV prevention and treatment programmes, HIV incidence remains unacceptably high in the rural subdistricts of KZN. The high incidence in women coupled with a higher proportion of HIV-positive males having detectable virus at higher median c/ml has implications for sustained HIV transmission.

# Sub article: TUAC0202 Spatial association between population viral load and HIV incidence

**Published:** 2016-07-22

**Authors:** B Riche, D Maman, S Wanjala, P Mendiharat, W Hennequin, A Vandenbulcke, I Mukui, F Subtil, R Ecochard

**Affiliations:** 1 Service de Biostatistique, Hospices Civils de Lyon, Lyon, France; 2 Clinical Research Department, Epicentre – Médecins Sans Frontières (MSF), Cape Town, South Africa; 3 Médecins Sans Frontières, Nairobi, Kenya; 4 Médecins Sans Frontières, Paris, France; 5 National AIDS and STDs Control Program, Nairobi, Kenya

## Abstract

**Introduction:**

Treatment as prevention programmes aims at reducing HIV incidence by increasing the proportion of HIV-positive individuals with undetectable viral load (VL) through ART roll-out. However, critical data on the relation between population VL and incidence are needed. We explored the spatial association between HIV incidence and population VL in a high HIV prevalence setting in western Kenya.

**Methods:**

We conducted a population-based survey of persons aged 15–59 years in Ndhiwa sub-county, Nyanza, Kenya, collecting spatial (cluster and health centre location) and individual (including, HIV status, incidence and VL) information. Population VL is defined as the proportion of individuals HIV positive with a VL >1000 cp/ml among the entire population. Population VL, HIV incidence and distance to the nearest health center (HC, delivering ART) were derived. A mixed Poisson regression model of incidence was used, adjusted on age (nine age groups), gender, distance to HC (three classes) and population VL in each cluster (six classes).

**Results:**

A total of 6076 individuals from 165 clusters participated in the survey. HIV prevalence was 24.1% (95% CI: 23.0–25.2). VL suppression among HIV-positive participants was 39.0% (95% CI: 35.9–42.2). Among all participants, 13.7% (95% CI: 12.9–14.6) were HIV-positive with a VL <1000 cp/ml. Incidence increased with population VL and was 1.7, 3.3, 4.8 and 5.4 new cases per 100 PY for a population VL of (5–10%), (10–15%), (15–20%) and (20–25%), respectively.

In the model, incidence was strongly associated with population VL. Relative risks were 1.26 (95% CI 0.6–2.6), 2.45 (95% CI 1.3–5.0), 3.40 (95% CI 1.8–7.0), 4.02 (95% CI 2.0–8.4) and 4.46 (95% CI 2.0–10.6) for a population VL of (5–10%), (10–15%), (15–20%) and (20–25%) compared to reference (0–5%).

**Conclusions:**

We found a strong association and gradient between HIV incidence and population HIV VL. This association suggests that population-level reduction of HIV incidence could be achieved by reducing population VL through ART roll-out in the general population.

# Sub article: TUAC0203 Mapping the HIV epidemic to improve prevention and care: the case of France

**Published:** 2016-07-22

**Authors:** L Marty, F Cazein, J Pillonel, D Costagliola, V Supervie

**Affiliations:** 1 Institut National de la Santé et de la Recherche Médicale (INSERM), Paris, France; 2 INVS, Paris, France

## Abstract

**Introduction:**

Despite decades of treatment efforts, in most settings, the number of new HIV infections remains unacceptably high and late HIV diagnosis remains common. To improve HIV prevention and care, a more focused response is needed using detailed data to map areas that are most impacted by HIV.

**Methods:**

We used data on newly diagnosed HIV cases from 2004 to 2012 and a back-calculation model, previously developed (Ndawinz et al. AIDS 2011, Supervie et al. AIDS 2014), to estimate, in France, at the national level, at the regional level and by HIV exposure group, three epidemiological indicators: HIV incidence, distribution of times from HIV infection to diagnosis and the number of undiagnosed HIV infections.

**Results:**

We estimated that in 2012, around 6800 (95% CI: 5900–7700) new HIV infections occurred in France, 24,600 (21,000–26,000) individuals were living with undiagnosed HIV and the median time from infection to diagnosis was 36 months (interquartile range (IQR): 12–64). HIV incidence and median time from infection to diagnosis were stable since 2004. HIV incidence and undiagnosed HIV prevalence rates were highest in French Guiana, French Antilles, Paris region (p<0.001; Figure 1a and b). Median time from infection to diagnosis was longest in French Guiana, Poitou-Charentes, Brittany, Limousin-Auvergne, Guadeloupe, Nord-Pas-de-Calais and Picardy (p<0.001; Figure 1c). The epidemic was mainly driven by both men who have sex with men and born abroad heterosexuals in the Paris region, and by born abroad heterosexuals in French Guiana.

**Conclusions:**

To the best of our knowledge, this is the first study that provides estimates of three main epidemiological indicators at a granular level, throughout the use of a detailed national data set. These estimates will be essential to tailor and evaluate a more focused HIV response.

**Abstract TUAC0203–Figure 1:** Estimates of (a) HIV incidence rates per 10,000; (b) undiagnosed HIV prevalence rates per 10,000; and (c) median time from infection to diagnosis (months).

# Sub article: TUAC0204 Ongoing high HIV incidence among women and men in Chókwè, southern Mozambique: a call for rapid scale up of combination HIV prevention

**Published:** 2016-07-22

**Authors:** R Nelson, R Thompson, I Casavant, S Pals, J Bonzela, D Mugabe, J Come, D Ujamaa, J Cardoso, A Auld, B Maculuve, S Wei, D Shodell, D MacKellar

**Affiliations:** 1 Centers for Disease Control and Prevention, Atlanta, GA, United States; 2 Chókwè Health Research and Training Center (CITSC), Chókwè, Mozambique; 3 Centers for Disease Control and Prevention, Maputo, Mozambique; 4 Jhpiego, Maputo, Mozambique; 5 Public Health Department Gaza, Xai-Xai, Mozambique; 6 Colorado Department of Public Health and Environment, Denver, CO, United States

## Abstract

**Introduction:**

In 2012, estimated HIV incidence among sexually active women in Chókwè Mozambique was 4.6 per 100 person-years (PY). In 2014, the Chókwè Health Demographic Surveillance System (CHDSS) incorporated HIV testing and counselling (HTC) within annual rounds of demographic surveillance. In this abstract, we report HIV incidence during the first two rounds of CHDSS HTC.

**Methods:**

CHDSS includes 95,589 residents in Chókwè town and six villages in southern Mozambique. During each round, all CHDSS households are visited and HTC is offered to all encountered residents. Round 1 was conducted in April 2014–May 2015, and round 2 in May–December 2015. Analyses are restricted to residents who tested HIV-negative in round 1 and retested in round 2. Adjusted incidence rates (IR) per 100 person-years were estimated with a generalized linear model using SAS 9.4.

**Results:**

Of 20,235 participants aged 15–59 years who tested HIV-negative in round 1, 10,826 (54%) retested in round 2. HIV incidence was high overall (adjusted IR, 4.7) and higher among residents in Chókwè town than villages (p<0.001). Among participants aged 15–24 years, incidence among females was higher than males (4.0 vs. 1.0; p<0.001); among participants aged 25–44 years, incidence was higher among males than females (10.8 vs. 7.9; p=0.02). Incidence was not statistically significantly different between males and females 45–59 years of age (p=0.08).

**Conclusions:**

HIV incidence is exceptionally high among residents aged 25–44 years in Chókwè District. Among persons under 25 years, HIV incidence is higher in women than in men; among older persons, incidence is higher in men. To reduce HIV incidence, combined evidence-based HIV prevention interventions such as HIV testing and linkage to care, antiretroviral treatment as prevention, and male circumcision should be scaled up in Chókwè district.

**Abstract TUAC0204–Figure 1:** Adjusted HIV incidence and 95% CIs, by sex and age-group.

**Abstract TUAC0204–Table 1:** Adjusted HIV incidence and 95% CI, total and by urbanicity

Demographic group	Participants	Incident infections	Follow-up person years	Crude IR per 100 person years	Adjusted IR per 100 person years
Total	10,826	473	8195.8	5.8 (5.3–6.3)	4.7 (4.2–5.4)
Chokwe Town	6670	329	4733.1	7.0 (6.2–7.7)	6.0 (5.2–6.9)
Villages	4152	144	3462.6	4.2 (3.5–4.9)	3.4 (2.8–4.2)

# Sub article: TUAC0205 District prevalence of unsuppressed HIV in South African women: monitoring programme performance and progress towards 90–90–90

**Published:** 2016-07-22

**Authors:** J Bor, A Brennan, MP Fox, M Maskew, W Stevens, S Carmona, W MacLeod

**Affiliations:** 1 Department of Global Health, Boston University School of Public Health, Boston, MA, United States; 2 Health Economics and Epidemiology Research Office, University of Witwatersrand, Johannesburg, South Africa; 3 Department of Epidemiology, Boston University School of Public Health, Boston, MA, United States; 4 National Health Laboratory Service, Johannesburg, South Africa

## Abstract

**Introduction:**

Population prevalence of unsuppressed HIV is a key determinant of HIV morbidity, mortality, and onward transmission, and can be reduced through both treatment and prevention strategies. We estimate unsuppressed HIV prevalence among reproductive-aged women in South Africa's 52 districts.

**Methods:**

District HIV prevalence for women between 15 and 49 years was estimated using national antenatal surveillance data, pooled for 2010–2012 to improve precision. Data were combined from District Health Information System (DHIS), National Health Laboratory Service (NHLS), and South African Census to estimate proportion of virally suppressed. We multiplied total numbers of patients remaining on antiretroviral therapy (ART) in 2012 (DHIS) by proportions of patients with viral loads (VL) in 2012 who were women aged 15–49 years and virally suppressed (NHLS). We divided the district population of women between 15 and 49 years (Census) to obtain the population prevalence of suppressed HIV. Subtracting this number from antenatal prevalence, we obtained *prevalence of unsuppressed HIV*. We also computed the ratio of these quantities, *percent of HIV-infected who are virally suppressed*. We assessed the relationship between each of these measures and district HIV prevalence in linear regression.

**Results:**

Prevalence of unsuppressed HIV varied widely across districts (5–33%, Figure 1a). By 2012, no district had achieved the 90×90×90 target of 72.9% population-level viral suppression in the study population of reproductive-aged women; however, there was a large variability in the percent of HIV-infected who were virally suppressed (9–39%, Figure 1b) and thus clear opportunity to identify the determinants of high-performing districts. Districts with the highest viral suppression had the highest HIV prevalence (p<0.001), suggesting successful targeting of resources, but also a need for renewed focus on districts in the second tier of HIV prevalence.

**Conclusions:**

Unsuppressed HIV prevalence and percent suppressed among the HIV-infected offer measures of unmet need and programme performance that can be estimated from routine programme and surveillance data.

**Figure 1:** Unsuppressed HIV prevalence and population viral suppression in South African districts.

# Sub article: TUAD0101 Social ecological contexts of HIV vulnerability among internally displaced women in Leogane, Haiti

**Published:** 2016-07-22

**Authors:** C Logie, C Daniel, Y Wang

**Affiliations:** 1 Factor-Inwentash Faculty of Social Work, University of Toronto, Toronto, Canada; 2 School of Social Work, Adelphi University, Long Island, WI, United States; 3 University of Toronto, Toronto, Canada

## Abstract

**Introduction:**

The confluence of poverty, increased gender-based violence and limited access to sexual health services elevate HIV infection risks among women displaced from natural disasters. Scant research has examined factors associated with condom use among internally displaced women in post-disaster settings, such as post-earthquake Haiti. Approximately, 65,000 people continue to experience protracted displacement in Haiti where they face chronic poverty, overcrowding, and unsafe living conditions. We examined factors associated with consistent condom use among internally displaced women in Haiti.

**Methods:**

This community-based study involved a cross-sectional survey with a peer-driven sample of internally displaced women in Leogane, Haiti. Peer health workers administered tablet-based structured interviews to internally displaced women (n=175). We conducted multivariate logistic regression analyses to assess correlates of past month condom use.

**Results:**

The 128 participants who reported being sexually active in the past 4 weeks were included in analyses. Two-thirds (n=84; 65.2%) reported consistent condom use in the past month. Three-quarters (n=95; 74.2%) of participants ate one meal or less per day. In multivariate logistic regression analyses controlled for age and income, consistent condom use in the past month was associated with meals per day (aOR 2.02, p=0.022), sexual relationship power (aOR 1.12, p=0.006), no reported intimate partner violence (aOR 2.82, p=0.022) and poor self-rated health (aOR 3.25, p=0.040). Participants who were less likely to report consistent condom use in the past month reported sex work involvement (aOR 0.09, p=0.004), shorter relationship duration (aOR 0.18, p=0.004), depression (aOR 0.62, p<0.001) and a higher number of sex partners in the past year (aOR 0.56, p< 0.001). This model explained 48.7% of the variation in consistent condom use scores (pseudo R^2^=0.487).

**Conclusions:**

Findings provide the first assessment of contextual factors associated with condom use among internally displaced women in post-earthquake Haiti. This research highlights the salience of a social ecological approach to understand the HIV vulnerability, underscoring intrapersonal (e.g. depression), interpersonal (e.g. relationship duration) and structural (e.g. food security, intimate partner violence) domains. Understanding social ecologies of HIV vulnerability among internally displaced women can inform complex, multilevel interventions that address food security, gender-based violence and depression, to advance HIV prevention in post-disaster settings.

# Sub article: TUAD0102 Still “at risk”: an examination of how street-involved youth understand, experience and engage with “harm reduction” in Vancouver's inner city

**Published:** 2016-07-22

**Authors:** N Bozinoff, D Fast, C Long, T Kerr, W Small

**Affiliations:** 1 British Columbia Centre for Excellence in HIV/AIDS, Vancouver, Canada; 2 Department of Medicine, St. Paul's Hospital, University of British Columbia, Vancouver, Canada; 3 Dalla Lana School of Public Health, University of Toronto, Toronto, Canada; 4 Faculty of Health Sciences, Simon Fraser University, Burnaby, Canada

## Abstract

**Introduction:**

Vancouver is an international leader in implementing interventions to reduce harms related to injection drug use, including a large needle exchange programme and North America's first government-sanctioned supervised injection facility. However, street-involved youth who use drugs continue to be vulnerable to HIV infection as a result of high rates of syringe sharing. To understand why youth in this setting continue to experience drug-related harms in the context of intensive public health intervention, we consider how these youths understand, experience and engage with harm reduction programmes in the context of entrenched marginalization.

**Methods:**

Twelve semi-structured interviews were conducted in 2013 with 13 youths (aged 17–28) recruited from the At-Risk Youth Study, a prospective cohort of 500 street-involved and drug-using youth. These interviews were embedded within a larger, 8-year programme of ethnographic research and explored the participants’ understandings of “harm reduction” in their use of specific services and their ideas about improving their day-to-day lives. Interviews were transcribed verbatim and a thematic analysis was performed.

**Results:**

Youth's understandings of and ideas about “harm reduction” were diverse, and went beyond public health efforts to minimize drug-related risks. Many youth articulated the limitations of existing programmes, indicating that while they reduce the risk of HIV transmission, they offer little meaningful support to improve youth's broader life chances. Youth described how they used “softer drugs” like marijuana to reduce the amount or frequency of substances deemed more harmful (e.g. crack cocaine, heroin) to their mental and physical health. They also indicated that using “softer drugs” allowed them to transition from intravenous routes of administration to oral, inhaled or intranasal routes. Finally, youth indicated that spatial considerations (e.g. distance from Vancouver's Downtown Eastside) strongly determined access to harm reduction services, and to the more expansive visions of “wellness” that they envisioned for themselves.

**Conclusions:**

In Vancouver, a large, well-established harm reduction infrastructure seeks to reduce drug-related harms such as HIV transmission among street entrenched youth. However, youth's multiple understandings, experiences and engagements with “harm reduction” in this setting illustrate the limitations of the existing infrastructure in improving their broader life chances and addressing their desires for structural change.

# Sub article: TUAD0103 Forced sex, migration and HIV infection among women from sub-Saharan Africa living in France: results from the ANRS Parcours study

**Published:** 2016-07-22

**Authors:** J Pannetier, A Ravalihasy, M Le Guen, N Lydié, R Dray-Spira, N Bajos, F Lert, A Desgress du Lou, Parcours Study Group

**Affiliations:** 1 Centre Population et Développement, Université Paris Descartes-IRD, Paris, France; 2 INSERM, CESP-U, Villejuif, France; 3 INPES, Saint-Denis, France; 4 INSERM, IPLESP UMRS, Paris, France; 5 IRD, Paris, France

## Abstract

**Introduction:**

In Europe, sub-Saharan African migrant women are a key population for HIV infection. Social hardships during migration may increase women vulnerability to sexual violence and HIV infection. The aim of this study is to assess the association between forced sex, migration and HIV infection among sub-Saharan African women living in France.

**Methods:**

Parcours is a life-event survey conducted from February 2012 to May 2013 in healthcare facilities in the Paris region, among two random samples of sub-Saharan migrant women: 570 receiving HIV care (156 acquired HIV in France) and 407 not diagnosed with HIV (reference group). Women were retrospectively asked whether they had ever been forced to have sex against their will and if happened, during which calendar year(s). Using mixed-effects logistic regression models, characteristics associated with an experience of forced sex after 14 years old in France, including migration history and living conditions each year after arrival in France, were first identified. Then, the frequency of forced sex after 14 years old in France was compared, adjusting for these characteristics, between women having acquired HIV either before or after migration and those HIV-uninfected.

**Results:**

Overall, 22.2, 23.1 and 18.3% of women HIV-infected before migration, HIV-infected after migration and HIV-uninfected, respectively, reported an experience of forced sex after 14 years old (childhood sexual abuse was about 4%), and, 3.8 17.3 and 4.2%, respectively, reported an experience of forced sex after arrival in France. Having migrated because of being threatened in the country of origin (aOR=5.96 (1.57–22.61)) and absence of stable (aOR=4.64 (1.69–12.79)) or own (aOR=2.72 (1.13,6.53)) housing in France were associated with a higher frequency of forced sex in France. Adjusting for migration history and living conditions, the frequency of forced sex in France was higher among women having acquired HIV in France compared to those HIV-uninfected (aOR=4.97 (1.63–15.12)), while no difference was found for those HIV-infected before migration (aOR=2.18 (0.78–6.04)).

**Conclusions:**

Among sub-Saharan African migrant women, HIV acquisition in France may be related to a context of sexual violence. Women whose migration was motivated by violence and those who experience social hardships in the host country are at high risk of sexual violence.

# Sub article: TUAD0104 Whoonga: off-label antiretroviral medication for recreational substance use and predicted implications for pre-exposure prophylaxis HIV prevention in South Africa

**Published:** 2016-07-22

**Authors:** C Kuo, D Operario, J Hoare, K Underhill, D Giovenco, M Atujuna, C Mathews, D Stein, L Brown

**Affiliations:** 1 Department of Behavioral and Social Sciences, Center for Alcohol and Addiction Studies, School of Public Health, Brown University, Providence, RI, United States; 2 Department of Psychiatry and Mental Health, University of Cape Town, Cape Town, South Africa; 3 Lifespan/Tufts/Brown Center for AIDS Research, Providence, RI, United States; 4 Center for Interdisciplinary Research on AIDS and Yale Law School, Yale University, New Haven, CT, United States; 5 Desmond Tutu HIV Foundation, Cape Town, South Africa; 6 Health Systems Research Unit, South African Medical Research Council, Tygerberg, South Africa; 7 Department of Psychiatry and Human Behavior, Alpert Medical School of Brown University, Providence, RI, United States

## Abstract

**Introduction:**

“Whoonga” is a colloquial term describing an illicit drug allegedly comprising antiretroviral medication used alone or in combination with cannabis, methamphetamine, heroin and other substances. Few studies characterize whoonga use among adolescents. Off-label use of antiretrovirals may diminish supply of antiretroviral treatment (ART) medication and contribute to non-adherence, medication resistance and an illicit drug epidemic.

**Methods:**

Emergent data on whoonga were derived from two adolescent HIV prevention studies conducted from 2015 to 2016 in Cape Town, South Africa. The first study was a baseline survey from an ongoing intervention study of family adolescent HIV prevention with N=399 adolescents and parents (adolescents: 100% Black African, 56% female, M=14 years; parents 100% Black African, 96% female, M=40 years). Participants were recruited through house-to-house community sampling and completed behavioural self-reports of whoonga use via a computerized mobile smartphone with audio computer-assisted self-interview software. The second study is an ongoing qualitative study of acceptability of HIV pre-exposure prophylaxis (PrEP) for adolescents involving focus groups and interviews with N=24 adolescents (M=100% Black African, 60% female, 16–17 years) and N=17 service providers. Adolescent participants were recruited using convenience sampling in community and clinic settings; service providers were recruited using respondent-driven sampling. We conducted descriptive analysis of quantitative survey data using SPSS and thematic analysis of qualitative data using NVivo. Brown University and University of Cape Town provided ethical approvals.

**Results:**

Nearly a fifth of adolescents reported whoonga use (3% used themselves, 14% knew someone who used). Administration included smoking (71%), snorting (15%), injecting (15%), ingesting (15%) and inserting (3%). Parents also reported whoonga use (4% used themselves, 7% knew someone who used). Administration included smoking (57%), ingesting (29%) and snorting (14%). Preliminary qualitative findings demonstrated clinicians knew of patient whoonga use and were concerned about how PrEP implementation would impact whoonga initiation and abuse. Adolescents used specific slang for individuals using whoonga and identified linkages between crime and whoonga abuse.

**Conclusions:**

Whoonga use is an emerging prevention challenge. Future studies should characterize the prevalence, composition, social and behavioural correlates of whoonga use, and further explore how the use of whoonga may be affected by PrEP implementation.

# Sub article: TUAD0105 Impact of a structural intervention to address alcohol use among gay bar-patrons in San Francisco: the PACE study

**Published:** 2016-07-22

**Authors:** J Hecht, A Plenty, J Lin, E Charlebois

**Affiliations:** 1 San Francisco AIDS Foundation, San Francisco, CA, United States; 2 Center for AIDS Prevention Studies, University of California, San Francisco, CA, United States

## Abstract

**Introduction:**

Men who have sex with men (MSM) have high rates of binge drinking (>50% in San Francisco (SF)), which can lead to increased sexual risk and other negative health outcomes. Heavy alcohol use is a recognized driver of the HIV epidemic in SF and gay bars have been identified as important venues for interventions addressing alcohol-related HIV risk. We sought to evaluate the impact on alcohol intake and blood alcohol concentration (BAC) of a pilot structural intervention to increase the availability of free water, coupled with messaging on pacing alcohol intake and normative feedback about BAC in a convenience sample of gay bars in San Francisco, CA, USA.

**Methods:**

From January 2012 to August 2014, study participants (n=1293) were recruited among exiting patrons of four gay bars in SF (two intervention bars and two control bars). Participants answered a brief survey regarding alcohol intake and sexual risk behaviours, and then completed a breathalyzer test to measure their BAC. Individuals’ measured BAC was displayed graphically in relation to others exiting the bar. Alcohol intake and measured BAC of participants were compared at baseline and post-intervention between control and intervention bar patrons using Pearson chi-square test.

**Results:**

No significant differences between intervention and control bars were found at baseline. Participants were 69% Caucasian, 11% Latino, 5% African-American, 7% Asian Pacific Islanders (API), 8% other race; mean age was 37.5 years. We found high levels of alcohol use and sexual risk across all participants (56% reported condomless sex with a potentially serodiscordant partner at last sex). Post-intervention, there were significant differences on measures of alcohol consumption: 30% of intervention bar participants had BAC levels over the legal limit (0.08 g/dl) compared to 43% of control bar participants, p<0.0001 and 78% of intervention bar participants were above the AUDIT-C cut-off for problematic drinking compared to 87% in control bars, p<0.001.

**Conclusions:**

It is feasible to partner with bar owners to implement a structural intervention to reduce BAC levels of customers. Increasing the availability of free water and alcohol intake pacing messaging in gay bars can decrease patron alcohol intake and may impact alcohol-related sexual risks for HIV.

# Sub article: TUAD0106LB ‘Sometimes I feel like the other life on heroin was better’: transitioning experiences of methadone clients and the potential implications in HIV prevention care and treatment in Nairobi, Kenya

**Published:** 2016-07-22

**Authors:** E Igonya, J Ndimbii, A Guise, F Owiti, T Rhodes, S Strathde

**Affiliations:** University of Nairobi, Nairobi, Kenya

## Abstract

**Introduction:**

In 2014, Kenya realized the ability to integrate new perspective in drug policy by introducing methadone (MAT) treatment for opiate-dependent persons. More than 500 people who use opiates have been enrolled in methadone in Nairobi. The study explored issues around access to and life experiences/changes with methadone, HIV care and treatment, health care and social support systems.

**Methods:**

In-depth interviews were conducted with HIV positive and negative men (n=8) and women (n=22) who were receiving methadone at a psychiatric hospital in Nairobi. Interviews were complimented by observations and informal conversations with clients within a community based drop-in centre as well as interviews with community stakeholders.

**Results:**

While clients were pleased with life changes brought about by methadone, a majority of study participants reported struggling with the transition from heroin and other drugs to methadone. In their daily lives, the labelled “MAT clients” struggle with social efficacy of MAT, thus immediate normalization of socioeconomic as well as sexual and reproductive lives in society. This results in tension between a new life on MAT and an old life on heroin and other drugs. This tension deepen a feeling of being a neglected population and lead some to revert to the “old life” of (injecting) using heroin, while others co-consume methadone and other drugs such as heroin. The temptations of the “old life” are exacerbated by the availability of heroin and other drugs in their environs; expectations of MAT; continued relations with friends who still use heroin and other drugs; a lack of income or being unoccupied; low self-esteem; and a lack of or limited social support. In addition, those HIV positive reported stigma from fellow MAT clients.

**Conclusions:**

The pressures of transition from heroin use to methadone is reported by some clients as involving a series of tensions that may lead to continued drug use and sexual risk of HIV, which may complicate the potential for methadone to support HIV prevention and treatment goals. Responses to these tensions could include pyscho-social support and structural interventions to facilitate the transition to use of methadone.

# Sub article: TUAD0201 Micro-level social and structural syndemic of HIV risk among Nepalese female sex workers

**Published:** 2016-07-22

**Authors:** K Deuba, S Anderson, AM Ekström, SR Pandey, R Shrestha, DK Karki, G Marrone

**Affiliations:** 1 Department of Public Health Sciences, Karolinska Institutet, Stockholm, Sweden; 2 Public Health and Environment Research Center, Kathmandu, Nepal; 3 Karolinska Institutet, Stockholm, Sweden; 4 FHI 360, Kathmandu, Nepal; 5 University of Bergen, Bergen, Norway; 6 Nobel College, Kathmandu, Nepal

## Abstract

**Introduction:**

Sex workers face stigma, discrimination and violence across the globe and are almost 14 times more likely to be HIV infected than other women in low- and middle-income countries. In Asia, condom campaigns at brothels have been effective in some settings, but for preventive interventions to be sustainable it is important to understand micro-level social and structural factors that enable sex workers to practice safer sexual behaviours. This study assesses the syndemic effects of micro-level social and structural factors of unprotected sex and the prevalence of HIV among female sex workers (FSW) in Nepal.

**Methods:**

In this quantitative study, 610 FSW were recruited using two-stage cluster sampling between September 2012 and November 2012 from 22 Terai highway districts of Nepal. Rapid HIV tests and face-to-face interviews were conducted to collect biological and behavioural information. A count of physical (sexual violence), social (poor social support and condom negotiation skills) and economic (unsafe sex to make more money) factors that operate at the micro-level was calculated to test the additive relationship to unprotected sex. Unprotected sex was assessed with the following question: “The last time you had sex with your client, did he use a condom?”. Point-biserial correlation was conducted to measure the size and significance of associations between each syndemic condition and unprotected sex. Statistically significant associations between independent variables and unprotected sex were computed using multivariable logistic regression.

**Results:**

The HIV prevalence was 1% in this presumably representative and large sample of FSW in Nepal. The prevalence of unprotected sex with client was high (24%). For each additional adverse physical, social and economic condition, the likelihood of unprotected sex with clients increased substantially: 1 problem=2.2 adjusted odds ratio (AOR); 95% confidence interval (CI)=1.3–3.7; 2 problems=3.1 AOR; 95% CI=1.8–5.4; 3–5 problems=7.3 AOR; 95% CI=3.9–13.9.

**Conclusions:**

Interactions between two or more adverse conditions linked to physical, social and economic environment increased the risk of unprotected sex among FSW. A more holistic approach, including efforts to improve condom negotiation skills and to address economic vulnerability and abuse, is required to address unprotected sex among FSW in Nepal.

# Sub article: TUAD0202 Physical and sexual violence against female sex workers in Cote d'Ivoire: prevalence, and the relationship between violence and structural determinants of HIV

**Published:** 2016-07-22

**Authors:** C Lyons, D Diouf, F Drame, A Kouamé, R Ezouatchi, A Bamba, M Thiam, B Liestman, S Ketende, S Baral

**Affiliations:** 1 Department of Epidemiology, Johns Hopkins Bloomberg School of Public Health, Baltimore, MD, United States; 2 Enda Sante, Dakar, Senegal; 3 Gaston-Berger University, Saint-Louis, Senegal; 4 Programme National de Lutte contre le Sida, Ministère de la Santé et de la Lutte contre le Sida, Abidjan, Cote D'Ivoire; 5 Gaston Berger University, Abidjan, Cote D'Ivoire; 6 Johns Hopkins Bloomberg School of Public Health, Baltimore, MD, United States

## Abstract

**Introduction:**

The HIV epidemic disproportionately affects female sex workers (FSW). Violence has been identified as an important measure in understanding HIV amongst FSW; however, limited data exist regarding the experience of physical and sexual violence amongst FSW in Cote d'Ivoire. Characterizing the prevalence of physical and sexual violence, as well as the relationship with structural HIV-related risks can inform the development and implementation of programmes and policies addressing health and human rights amongst FSW.

**Methods:**

FSW, 18 years or older, were recruited through respondent-driven sampling in Abidjan, Côte d'Ivoire. A total of 466 participants completed a socio-behavioural questionnaire. Prevalence estimates of physical and sexual violence were evaluated as both crude and RDS adjusted estimates. The relationships between protection, coercive sexual risk, economic work environment, and physical and sexual violence were analyzed using chi squared tests, and bivariate and multivariable logistic regression.

**Results:**

The RDS-adjusted prevalence estimate of physical violence amongst FSW in Cote d'Ivoire is 60.6%, and sexual violence is 44.1%. Among the study sample, police refusal of protection was associated with increased experience of sexual violence (odds ratio (OR): 3.14; adjusted odds ratio (aOR): 1.71; 95% CI: 1.02, 4.89). Being blackmailed because of FSW status was associated with physical (OR: 2.27; aOR: 2.08; 95% CI: 1.07, 4.04) and sexual violence (OR: 2.92; aOR: 1.96; 95% CI: 1.17, 4.65).

**Conclusions:**

Violence amongst FSW in Cote d'Ivoire is prevalent and shown to be severe and reoccurring. High levels of violence perpetrated by clients and low levels of reported protection highlight a need for improved work environments for FSW in Cote d'Ivoire. Considering the policy and risk environment in Cote d'Ivoire, targeting the macrostructure through improved work environment and increased protection may be an effective way to address the cascade of barriers in realizing health and human rights for FSW in Cote d'Ivoire.

# Sub article: TUAD0203 Experiences of childhood trauma increases HIV-risk behaviours in young women and men in urban informal settlements in South Africa

**Published:** 2016-07-22

**Authors:** A Gibbs, K Dunkle, T Khumalo, N Ntini, L Washington, N Mbatha, E Chirwa, S Willan, Y Sikweyiya, N Jama-Shai, R Jewkes

**Affiliations:** 1 Health Economics and HIV/AIDS Research Division, Durban, South Africa; 2 Gender and Health Research Unit, Medical Research Council, Pretoria, South Africa; 3 Project Empower, Durban, South Africa

## Abstract

**Introduction:**

Young adults in informal settlements experience high HIV-incidence. While childhood trauma is known to increase HIV risks factors including violence, multiple partnering and substance use in other settings, little is known about the impact of childhood trauma in informal settlements.

**Methods:**

We drew on cross-sectional data from 320 women and 319 men aged 18–38 in informal settlements in Durban, South Africa, comprising the control arm of a cluster randomized RCT. Questionnaires collected scores assessing childhood trauma before 18, including experiences of physical violence, sexual abuse, emotional violence and harsh parenting. Outcomes were HIV-risk factors assessed over the past 12 months: three or more main partners, three or more causal partners, three or more once-off partners, intimate partner violence (IPV) and non-partner sexual violence and problematic alcohol use. For each outcome we built (male/female) regression models controlling for clustering and potential confounding variables.

**Results:**

Mean ages were 24.4 years for women and 23.4 years for men. Before the age of 18, 76.2% of men and 71.3% of women reported witnessing or experiencing physical violence; 48.0% of men and 34.7% of women experienced sexual violence; and 62.1% of men and 56.9% of women experienced emotional violence.

For women, increasing childhood traumas were associated with more once-off sexual partners, experiencing IPV, non-partner sexual violence and problematic alcohol use.

For men, increasing childhood traumas were associated with more main, casual and once-off sexual partners and perpetrating IPV and non-partner sexual violence.

**Conclusions:**

Experiences of childhood traumas were consistently associated with increased HIV-risk behaviours amongst young women and men in urban informal settlements, particularly number of recent sexual partners and experience/perpetration of IPV and non-partner sexual violence. Intervening with children and caregivers before 18 to reduce childhood trauma is critical for reducing future HIV-risk.

**Abstract TUAD0203–Table 1:** Women: childhood traumas associated with HIV risks

	Childhood trauma mean (CI 95%)			
		
Past 12-month outcomes	Yes	No	aOR (CI)	p
3 or more once-off sexual partners	9.1 (7.5–10.7)	5.7 (5.0–6.4)	1.08 (1.00–1.16)	p<0.05
Experience of physical and/or sexual IPV	7.2 (6.5–8.0)	4.6 (3.8–5.4)	1.08 (1.01–1.15)	p<0.05
Experience of non-partner sexual violence	9.3 (8.1–10.5)	4.9 (4.3–5.4)	1.13 (1.06–1.21)	p<0.0001
Problematic alcohol use	9.4 (7.9–10.9)	5.4 (4.8–5.9)	1.07 (1.00–1.15)	p<0.05

**Abstract TUAD0203–Table 2:** Men: childhood traumas associated with HIV risks

	Childhood trauma mean (CI)			
		
Past 12-month outcomes	Yes	No	aOR (CI)	p
3 or more main sexual partners	8.8 (7.5–10.0)	6.7 (5.9–7.6)	1.08 (1.02–1.13)	p<0.01
3 or more casual sexual partners	9.2 (7.5–10.8)	6.9 (6.1–7.7)	1.06 (1.00–1.11)	p<0.05
3 or more once-off sexual partners	8.7 (7.3–10.1)	7.0 (6.1–7.8)	1.06 (1.00–1.11)	p<0.05
Perpetrating any IPV	9.3 (8.3–10.2)	5.1 (4.2–6.0)	1.14 (1.07–1.21)	p<0.0001
Perpetrating non-partner sexual violence	9.5 (8.3–10.7)	6.1 (5.3–6.9)	1.07 (1.02–1.13)	p<0.01

# Sub article: TUAD0204 HIV+ diagnoses during pregnancy increases risk of IPV postpartum among women with no history of IPV in their relationship

**Published:** 2016-07-22

**Authors:** AK Groves, HL McNaughton Reyes, D Moodley, S Maman

**Affiliations:** 1 Department of Sociology, Center on Health, Risk and Society, American University, Washington, DC, United States; 2 Department of Health Behavior, University of North Carolina at Chapel Hill Gillings School of Global Public Health, Chapel Hill, NC, United States; 3 Department of Obstetrics and Gynaecology, Nelson Mandela School of Medicine, University of KwaZulu Natal, Durban, South Africa

## Abstract

**Introduction:**

There have been mixed findings on the relationship between HIV and women's risk of intimate partner violence (IPV). Per the dual vulnerability model, it may be that HIV infection matters only for particular relationships. Specifically, when you add an HIV-positive diagnosis into an already stressed relationship (as indicated by IPV history) it may work synergistically to increase IPV risk. In contrast, women's relationships where there is no history of IPV may be more resilient to an HIV-positive diagnosis. Therefore, the aim is to test whether the positive association between HIV status and IPV will be exacerbated for women with a history of IPV.

**Methods:**

Data come from 1064 women who participated in the baseline antenatal visit and 9-month postpartum follow-up visit as part of a larger RCT. We conducted logistic regression analysis to examine our hypothesis. Model 1 assessed whether HIV diagnosis at baseline predicted physical IPV at follow-up, controlling for demographic covariates. Model 2 included an interaction between HIV diagnosis and history of IPV.

**Results:**

While HIV was not associated with postpartum IPV in the main effects model (AOR: 1.44, 95% CI: 0.78–1.97), there was a statistically significant interaction between HIV diagnosis and having a history of IPV (AOR: 0.40, 95% CI: 0.17–0.96). The findings were in the opposite direction as expected: among women who had a history of IPV in the relationship, HIV status did not predict IPV postpartum (AOR: 0.87, 95% CI: 0.49–1.55). Yet among women who had no history of IPV in the relationship, receiving an HIV-positive diagnosis during pregnancy predicted postpartum IPV (AOR: 2.17, 95% CI: 1.06–4.42).

**Conclusions:**

Receiving an HIV-positive diagnosis in pregnancy did not exacerbate postpartum IPV for women with a history of IPV in their relationship; the diagnosis may not signify new stress within the relationship. However, the findings have important implications for women with no history of IPV. That is, women who test HIV-positive and have no history of IPV should be counselled regarding their future risk of IPV in their relationship. Given the negative health ramifications of IPV during the perinatal period for women and their children, IPV prevention interventions are needed.

# Sub article: TUAD0205 Physical assault partially mediates the impact of transgender status on depression and poly-substance use among black MSM and black transgender women in the United States: results from POWER

**Published:** 2016-07-22

**Authors:** L Bukowski, R Coulter, N Riley, S Buehler, C Hoffmann, A Gehr-Seloover, R Stalll The Power Study Team

**Affiliations:** Graduate School of Public Health, University of Pittsburgh, Pittsburgh, PA, United States

## Abstract

**Introduction:**

Black men who have sex with men (BMSM) and black transgender women (BTW) are vulnerable to physical assault, poly-substance use, and depression, outcomes that drive new HIV infections and poor HIV-related health outcomes in both populations. Though BTW are different than BMSM, no studies have examined how the manifestations of these outcomes may differ between populations. In order to fill this gap, we examined differences in physical assault, poly-substance use and depression between BMSM and BTW, and we investigated whether physical assault mediates differences in poly-substance use and depression.

**Methods:**

Cross-sectional data for our analysis came from the first two years of the ongoing study, *Promoting Our Worth, Equality, and Resilience* (POWER). In 2014 and 2015, POWER employed time-location sampling (TLS) to recruit a community-based sample of BMSM and BTW (n=3426) who attended Black Pride events in six US cities. Participants completed a behavioural health survey and were offered onsite HIV-testing. A total of 2997 BMSM and 277 BTW (n=3274) provided complete data for our analysis. All TLS weighted multivariable models controlled for age, education and city.

**Results:**

BTW had significantly higher prevalence of physical assault than BMSM (44.8% vs. 12.3%, respectively). In multivariable models, compared to BMSM, BTW had greater physical assault (AOR=5.2; 95% CI: 3.9–7.0), poly-substance use (AOR=7.2; 95% CI: 4.9–10.6) and depression (AOR=3.3; 95% CI: 2.5–4.4). Physical assault attenuated the effects of transgender status on poly-substance use (AOR=3.9; 95% CI: 2.6–5.9) and depression (AOR=2.4; 95% CI: 2.5–4.4). The indirect effect of transgender status on poly-substance use via physical assault was 1.4 (95% CI: 1.2–1.7), and the indirect effect of transgender status on depression via physical assault was 1.3 (95% CI: 1.15–1.47). Physical assault partially mediated the relationships of transgender status with poly-substance use and depression.

**Conclusions:**

BTW face an epidemic of physical assault. If this epidemic continues, efforts to address depression and poly-substance use as well as other downstream health outcomes (e.g. HIV incidence, HIV-related health outcomes) among BTW will be futile. Interventions addressing structural inequity are necessary to alleviate the instances of physical assault perpetrated against BTW.

# Sub article: TUAD0301 Challenges to advancing HIV research in pregnancy: insights from the HIV research community

**Published:** 2016-07-22

**Authors:** R Faden, S Gilbert, K Sullivan, J Cadigan, C Krubiner, E Namey, M Little, A Lyerly, A Mastroianni

**Affiliations:** 1 Berman Institute of Bioethics, Johns Hopkins University, Baltimore, MD, United States; 2 School of Medicine, University of North Carolina, Chapel Hill, NC, United States; 3 FHI 360, Durham, NC, United States; 4 Kennedy Institute of Ethics, Georgetown University, Washington, DC, United States; 5 School of Law, University of Washington, Seattle, WA, United States

## Abstract

**Introduction:**

Concerns about including pregnant women in research have led to a dearth of evidence to guide safe and effective treatment and prevention of HIV in pregnancy. We aimed to identify the range of barriers to conducting research in this area.

**Methods:**

We conducted a series of consultations with HIV investigators and clinicians to elicit their views and experiences in conducting HIV research involving pregnant women. We solicited input from 55 colleagues in small groups or one-on-one sessions to discuss priorities and barriers to research with pregnant women. Content analysis was used to identify themes.

**Results:**

Participants discussed a breadth of areas of needed research, including safety, efficacy and appropriate dosing of: newer ARVs for pregnant women; emerging preventive strategies; and treatment for HIV's co-infections, including but not limited to tuberculosis. Challenges to conducting research on pregnancy and HIV included regulatory and legal barriers, such as restrictive interpretations of current regulations; financial disincentives stemming from funders’ views that pregnant women cost more to include and represent a small sub-population; social and cultural research norms, such as fear of reputational damage if harms arise for pregnant women or their foetuses; and logistical difficulties, such as challenges recruiting enough pregnant women to sufficiently power data analysis.

**Conclusions:**

Despite broad recognition of research gaps related to HIV and pregnancy, investigators face numerous challenges to advancing needed research. Clearer guidance for navigating the complex legal, regulatory and ethical landscape is needed to advance women's health at the intersection of pregnancy and HIV.

**Abstract TUAD0301–Table 1:** Pressing research gaps relevant to pregnancy and HIV

Treatment	Co-infection and co-morbidities	Other/cross-cutting
Choice of ARV to optimize short and long term maternal health Optimal dosing throughout pregnancy Continuation of therapy after treatment for PMTCT	Safety and efficacy of treatment for HIV+ pregnant women with: TB and malaria Opportunistic infections Hepatitis B and C Hypertension, eclampsia, pre-eclampsia Diabetes	PK studies New delivery mechanisms (e.g. rings and patches) HIV and fertility

**Prevention**	**Diagnostics**	**Vaccines**

Chemoprevention (PrEP, microbicides) Safe conception	Optimal HIV testing throughout pregnancy Resistance testing	HIV vaccine trials Other vaccine trials with HIV-infected participants

**Abstract TUAD0301–Table 2:** Barriers to conducting clinical research relevant to pregnancy and HIV

Ethical	Legal	Research environment and culture
Calculating risk/benefit ratio Ensuring informed consent, including what role, if any, the biological father should have in decision making Burden on pregnant women of research study requirements	Difficulty interpreting US human subjects regulations related to pregnant women (what is “minimal risk” in clinical research?) Concerns about study disapproval by IRBs Studies with pregnant minors raise additional regulatory concerns Liability if study results in harm	Reputational risk to the researcher if study results in foetal harm Belief that funders, regulators, and public would only view observational studies as ethical Easier to obtain funding and conduct research with other populations

**Financial**	**Analytical**	**Logistical**

Required follow-up and potential ancillary care for women who become pregnant on study are costly Pharma does not perceive pregnant women as a lucrative market segment (little incentive to study with potential liability as a disincentive) Funders fail to prioritize research on pregnancy	Data from pregnant women or women who become pregnant during study must be analyzed separately Low statistical power due to likely small sample size	Most trials do not enrol pregnant women and require women who become pregnant to withdraw, so even conducting opportunistic or observational studies is difficult Need for “buy-in” from men for studies in many international settings Locating participants for data collection at fixed time points in pregnancy

# Sub article: TUAD0302 Future pregnancy intentions and knowledge of methods for safer conception among female sex workers in Port Elizabeth, South Africa

**Published:** 2016-07-22

**Authors:** A Rao, S Baral, N Phaswana-Mafuya, A Lambert, Z Kose, M Mcingana, C Holland, S Ketende, S Schwartz

**Affiliations:** 1 Department of Epidemiology, Johns Hopkins University, Baltimore, MD, United States; 2 Human Sciences Research Council, Port Elizabeth, South Africa; 3 TB/HIV Care Association, Port Elizabeth, South Africa

## Abstract

**Introduction:**

Female sex workers (FSW) are disproportionately affected by HIV and experience high rates of pregnancy. The objective of these analyses is to assess fertility intentions and the impact of HIV on pregnancy intentions and safer conception knowledge among FSW in Port Elizabeth, South Africa.

**Methods:**

FSW in Port Elizabeth were recruited into a cross-sectional study using respondent-driven sampling. Participants completed an interviewer-administered questionnaire asking about future fertility intentions and were provided HIV testing and counselling. Robust Poisson regression was used to model adjusted prevalence ratios (aPrR) for correlates of positive fertility intentions among FSW<45 years. Knowledge of safer conception methods was described using Fisher's exact tests.

**Results:**

Overall 391 FSW were represented in the analyses. Just over 50% (203/391) had received a prior HIV diagnosis and an additional 12% (46/391) were diagnosed with HIV during the study. Slightly under half of FSW (185/391) reported future pregnancy intentions (47%). In bivariate analyses, knowledge of prior HIV diagnosis was negatively associated with pregnancy intentions as compared to HIV-negative women (PrR=0.68, 95% CI (0.55–0.85)). Older age, greater number of children living, and more years selling sex were also significantly negatively associated with pregnancy intentions. Being in a relationship was significantly positively associated with pregnancy intentions. In multivariate analyses, only parity remained significantly associated with future pregnancy intentions. Knowledge of safer conception methods, such as timed condomless sex, pre-exposure prophylaxis or self-insemination, was low and non-statistically significantly different between those with and without pregnancy plans (Table 1).

**Conclusions:**

Pregnancy intentions were high and not independently associated with HIV status. Moreover, there was limited knowledge of safer conception methods suggesting the need for specific advice for FSW on how to conceive safely given that most women were living with HIV and have specific sexual risks of HIV acquisition and transmission given their occupation.

**Abstract TUAD0302–Table 1:** Knowledge of HIV prevention strategies for conception by known HIV status among 391 female sex workers in Port Elizabeth, South Africa

	Overall, n=391	HIV−, n=142	HIV+, no prior dx, n=46	HIV+, prior dx, n=203	p-value
ARVs for the infected partner					
No	300 (76.7)	113 (79.6)	42 (91.3)	145 (71.4)	0.007
Yes	91 (23.3)	29 (20.4)	4 (8.7)	58 (28.6)	
PrEP for the uninfected partner					
No	374 (95.7)	135 (95.1)	45 (97.8)	194 (95.6)	0.883
Yes	17 (4.4)	7 (4.9)	1 (2.2)	9 (4.4)	
Treatment when pregnant					
No	203 (51.9)	91 (64.1)	30 (65.2)	82 (40.4)	<0.001
Yes	188 (48.1)	51 (35.9)	16 (34.8)	121 (59.6)	
Self-insemination					
No	387 (99.0)	140 (98.6)	46 (100.0)	201 (99.0)	1.00
Yes	4 (1.0)	2 (1.4)	0 (0.0)	2 (1.0)	
Timed intercourse					
No	387 (99.0)	140 (98.6)	46 (100.0)	201 (99.0)	1.00
Yes	4 (1.0)	2 (1.4)	0 (0.0)	2 (1.0)	
Sperm-washing/in-vitro fertilization					
No	363 (92.8)	130 (91.6)	44 (95.7)	189 (93.1)	0.694
Yes	28 (7.2)	12 (8.4)	2 (4.3)	14 (6.9)	
Using a sperm donor					
No	360 (92.1)	132 (93.0)	46 (100.0)	182 (89.7)	0.038
Yes	31 (7.9)	10 (7.0)	0 (0.0)	21 (10.3)	
Adoption					
No	300 (76.7)	109 (76.8)	44 (95.7)	147 (72.4)	0.001
Yes	91 (23.3)	33 (23.2)	2 (4.3)	56 (27.6)	

# Sub article: TUAD0303 “I got tested so I could not lose him”: HIV testing practices and subsequent sexual behaviours of sex workers and clients in Mombasa, Kenya

**Published:** 2016-07-22

**Authors:** TB Masvawure, Y Lafort, S Chabeda, A Restar, P Gichangi, J Tocco, T Sandfort, J Mantell

**Affiliations:** 1 New York State Psychiatry Institute (NYSPI), HIV Center for Clinical and Behavioral Studies, New York, United States; 2 Department of Sociology and Anthropology, College of the Holy Cross, Worcester, MA, United States; 3 Ghent University, Gent, Belgium; 4 International Centre for Reproductive Health, Mombasa, Kenya; 5 Mailman School of Public Health, Columbia University, New York, NY, United States

## Abstract

**Introduction:**

HIV testing is a critical step toward accessing treatment for individuals who test positive, but there is no consensus over whether knowing one's HIV status leads to less – or more – sexual-risk taking for individuals who test HIV-negative. We examined the HIV testing practices of female and male sex workers (FSWs, MSWs) and clients in Mombasa and how HIV testing relates to their subsequent sexual behaviours.

**Methods:**

We conducted 75 semi-structured interviews with sex workers and clients recruited from 18 bars/nightclubs in Mombasa to guide intervention development. Eligibility criteria were being ≥18 years, regular patron of venue, solicited vaginal/anal intercourse with sex worker/client at that venue in last 3 months, willingness to be audio-recorded, and being visibly sober.

**Results:**

Most participants had tested for HIV in the previous 12 months. HIV testing was more common among sex workers than clients, with some testing three times a year. HIV testing was undertaken both as a *response to* and as a *reason* for engaging in condomless sex. For instance, participants sought HIV testing following a high-risk sexual encounter, such as after condomless sex, condom breakage with commercial sex partners, or after a sexually transmitted infection. Some sex workers and clients, however, reported getting tested in order to engage in condomless vaginal and anal sex with main *and* regular commercial sex partners, particularly those with whom condom use was difficult to realize. Knowing a partner's HIV status or testing with a partner were frequently given as explanations for not using condoms with these partners. Many FSWs tested on the same or next day after they engaged in high-risk sex so that they could get post-exposure prophylaxis. Other participants sought testing weeks or months later.

**Conclusions:**

HIV testing is used by some sex workers and clients as a reason to engage in condomless sex with commercial partners. However, given the high HIV risk involved in sex work, HIV prevention programmes should continue to underscore the importance of both HIV testing and consistent condom use in these encounters.

# Sub article: TUAD0304 Behind closed doors: sex, reproduction and the household space in rural south-western Uganda

**Published:** 2016-07-22

**Authors:** A Tam, G Tumwekwase, E Kabunga, S Russell, J Seeley

**Affiliations:** 1 School of International Development, University of East Anglia, Norwich, United Kingdom; 2 MRC/UVRI Uganda Research Unit on AIDS, Entebbe, Uganda; 3 London School of Hygiene and Tropical Medicine, London, United Kingdom

## Abstract

**Introduction:**

In south-western Uganda, HIV prevalence and total fertility rates are high amongst married individuals. Sex and reproduction in marriage primarily takes place in the home. This space is associated with privacy and preconceived ideas of gender norms, which can pose challenges to the negotiation and management of sexual and reproductive health (SRH) behaviours. This study set out to understand the challenges, risk perceptions and strategies used by men and women at different stages of the life-course to manage and negotiate conflicting SRH issues in marriage.

**Methods:**

Data collection took place over 12 months within an existing general population cohort in rural south-western Uganda. Methods included life-story interviews and focus groups with individuals who had ever been married. Participants were randomly selected from six villages, where HIV prevalence ranges between 4.5 and 16%. In-depth interviews were also conducted with religious leaders, traditional healers and health workers. Iterative thematic analysis was used to interpret, code and organize the data.

**Results:**

Developing the home, unprotected sex and having children are central to the marital relationship in this setting. Failure to fulfil cultural expectations of gender roles and SRH behaviours within the household space were associated with marital dissatisfaction, relationship instability and extra-marital relations. This paper focuses on the SRH strategies described by men and women at different stages of marriage and the life-course. Strategies include: claiming space and time for sexual intimacy, techniques for managing menstruation, hygiene practices, and the various methods used to achieve fertility preferences and resolve SRH problems.

**Conclusions:**

Individuals in marital relationships face specific challenges in managing SRH risks due demands in fulfilling gender roles, maintaining the marital relationship and stability of the home, whilst also achieving sexual desires and fertility preferences. Understanding the context and cultural meanings of sex and reproduction can facilitate tailored SRH intervention with married individuals.

# Sub article: TUAD0305 Youth sexual and reproductive health in China: results from a national survey of 18,000 Chinese college students

**Published:** 2016-07-22

**Authors:** C Li, Y Zhao, H Zhang, Z Cheng, P Hong, X Liang, J Gaoshan, L Li, K Tang

**Affiliations:** 1 School of Public Health, Peking University, Beijing, China; 2 Institute for Medical Humanities, Peking University, Beijing, China; 3 Peking University Health Science Center, Beijing, China; 4 China Family Planning Association, Beijing, China; 5 United Nations Population Fund (UNFPA), Beijing, China; 6 Department of Global Health, Peking University, Beijing, China

## Abstract

**Introduction:**

Notwithstanding years of efforts guided by *the National Strategy on HIV/ADIS Prevention and Control in Schools*, a climbing trend of HIV infections among college students has been observed in China [1]. This study investigated the status quo of HIV knowledge and sexual behaviour patterns among college students, aiming to provide evidence for policy makers to identify priority areas of HIV/AIDS prevention and control [1].

**Methods:**

This study was conducted from January to August in 2015 through a multi-stage sampling approach. 130 colleges were selected from eastern, central and western China. The internet-based survey questionnaire was subsequently delivered to the focal points in each school for voluntary participation Logistic and linear regression were used to explore the association between risk factors and attitude/behavioural outcomes, under SAS 9.2 with a significant level of 0.05.

**Results:**

17,966 students were included, of which 90% came from the 130 schools. 94.8% of the surveyed population responded. Only 55.6% of the students had received sexual education from school. 20.3% of the respondents didn′t know that HIV could not be spread by sharing eating utensils. 47.0% in homosexual group had sex before (19.3% for heterosexual, 30.1% for bisexual). The proportion of condom use during the last sexual intercourse was 62.5%. 22.7% reported to have STI-related symptoms. Logistic regression results indicated that knowledge about sexual and reproductive health, family structures and sexual orientation were strongly associated with high-risk sexual behaviours, while gender, parents' education, stratifications of residential area and types of degree programmes were significant effect modifiers for knowledge on HIV/AIDS-related information.

**Conclusions:**

High prevalence of risk sexual behaviours suggested that students remain a vulnerable population in terms of HIV/AIDS and sexually transmitted infections (STIs), especially among sexual minority groups. Future school-based interventions should be designed with a more gender-sensitive and individualized approaches.

**Figure 1:** Cumulative number of Sexual Partners by Sexual Orientation.

## Reference

1. Ministry of Education. (2015). Retrieved from: http://www.moh.gov.cn/jkj/s3585/201508/e4c8a1e6809c4a8e9c49f7f8708873d1.shtml

# Sub article: TUAD0401 “We talk, we do not have shame”: reducing HIV and sex work stigma through social cohesion among FSW living with HIV in the Dominican Republic

**Published:** 2016-07-22

**Authors:** MA Carrasco, C Kennedy, C Barrington, M Perez, Y Donastorg, D Kerrigan

**Affiliations:** 1 Johns Hopkins University, Bloomberg School of Public Health, Baltimore, MD, United States; 2 Department of Health Behavior, University of North Carolina Gillings School of Global Public Health, Chapel Hill, NC, United States; 3 HIV Vaccine Research Unit, Instituto Dermatalogico y Cirugia de Piel Dr. Humberto Bogart Diaz, Santo Domingo, Dominican Republic

## Abstract

**Introduction:**

Layered HIV and sex work stigma pose a significant barrier for female sex workers (FSW) living with HIV to engage in HIV prevention behaviours and access HIV treatment and care. How FSW manage and address layered stigma is not well understood. We explore the experiences of layered stigma among participants in *Abriendo Puertas*, a multi-level HIV/STI prevention, treatment and care intervention for FSW living with HIV in Santo Domingo, Dominican Republic. Additionally, we examined social cohesion as a key community-driven strategy to address HIV and sex work stigma.

**Methods:**

Using purposeful sampling, we conducted 23 in-depth interviews and two focus groups (n=11) with FSW living with HIV participating in the *Abriendo Puertas* intervention. Transcripts were analyzed using thematic analysis. First, constructs related to Foucault's conceptualization of power and discipline were identified. Then, transcripts were analyzed to identify individual and community narratives resisting and subverting stigma and shame.

**Results:**

According to Foucault, modern power is productive, giving life to those who adhere to social norms while disallowing it from those who transgress them through various forms of social disciplining. Foucault also proposed that such disciplining is often internalized leading to self-disciplining. We found that FSW living with HIV experience various instances of societal disciplining including domestic violence, verbal abuse, social rejection and employer discrimination. They also experience self-disciplining in the form of hopelessness, low self-esteem and loss of the will to live. The enhancement of social cohesion through participation in *Abriendo Puertas* was experienced as a means to subvert oppressive social norms around sexuality and healthism. This was verbalized as regaining hope and improving self-efficacy, self-esteem, access to social support and motivation to adhere to HIV treatment. Indeed, social cohesion provided the psychosocial space to reconstruct identity in more positive terms than those afforded by society. This was done through the production, repetition and performance of de-stigmatized narratives in a safe space.

**Conclusions:**

Findings indicate the importance of social cohesion as a means to challenge oppressive social norms and reduce layered stigma. HIV prevention, treatment and care interventions for FSW living with HIV should include components to enhance social cohesion.

# Sub article: TUAD0402 Effects of a brief affirmation intervention on HIV-related distress and positive living intentions among individuals recently diagnosed with HIV in Lesotho

**Published:** 2016-07-22

**Authors:** K Hartson, D Sherman, S Mofelehetsi, T Ncheke, M Khasoane

**Affiliations:** 1 Department of Medicine, University of California, Center for AIDS Prevention Studies, San Francisco, CA, United States; 2 Department of Psychological and Brain Sciences, University of California, Santa Barbara, CA, United States; 3 LENASO, Maseru, Lesotho

## Abstract

**Introduction:**

In addition to the physical burden of the illness, HIV also carries a psychological burden as those recently diagnosed with HIV navigate identity-related concerns including fears regarding how to live a normal life with HIV, possible stigmatization, and whether to disclose one's HIV status. This study investigated using a novel psychological intervention to reduce the psychological distress associated with being HIV positive and, in turn, improve intentions to live positively.

**Methods:**

A total of 331 participants (207 women, mean age of 41) were recruited, following HIV diagnosis, from 10 clinics and hospitals in the Maseru and Leribe districts of Lesotho. Participants were randomly assigned to either the affirmation intervention condition or the control condition. Those in the affirmation intervention condition completed a brief, 10-minute exercise where they reflected on important values before receiving positive living counselling. Those in the control condition simply received positive living counselling without completing the affirmation task. Following the counselling sessions, all participants completed measures of their current HIV-related stress and distress and their intentions to engage in positive living behaviours. The impact of the affirmation intervention on participants’ HIV-related distress and positive living intentions was evaluated by separate one-way analyses of variance.

**Results:**

Overall, participants in the affirmation condition reported less HIV-related distress relative to those in the control condition. More specifically, participants in the affirmation condition reported experiencing significantly less stress (mean=1.79 vs. mean=2.17, p=0.006), fewer intrusive HIV-related thoughts (mean=2.17 vs. mean=2.62, p=0.011), fewer distressing emotions in response to HIV (mean=1.27 vs. mean=1.46, p=0.027), and were less worried about dying from HIV (mean=1.86 vs. mean=2.31, p=0.012). Additionally, those in the affirmation condition also reported greater positive living intentions including intentions to use condoms (mean=5.56 vs. mean=5.38, p=0.046) and to take medications properly (mean=5.53 vs. mean=5.30, p=0.029). Finally, the effect of the affirmation intervention on participants’ intention to live positively was mediated by the extent to which the intervention reduced participants’ HIV-related stress.

**Conclusions:**

These findings provide initial evidence in support of using brief affirmation interventions in clinical settings to reduce the stress associated with HIV diagnosis and to improve openness to positive living health messages.

# Sub article: TUAD0403 “Happy in my own skin”: impact of anti-stigma interventions on people living with HIV in Toronto, Canada

**Published:** 2016-07-22

**Authors:** JP-H Wong, K Fung, C Hui, H Luyombya, A Bisignano, D Maitland, K Poon, AT-W Li

**Affiliations:** 1 Daphne Cockwell School of Nursing, Ryerson University, Toronto, Canada; 2 Dalla Lana School of Public Health, University of Toronto, Toronto, Canada; 3 University of Toronto, Toronto, Canada; 4 University Health Network, Toronto, Canada; 5 Asian Community AIDS Services, Community Engagement & Ontario Positive Asians (OPA+) Coordinator, Toronto, Canada; 6 Committee for Accessible AIDS Treatment, Toronto, Canada; 7 Regent Park Community Health Centre, Toronto, Canada

## Abstract

**Introduction:**

HIV stigma negatively affects the physical and psychological wellbeing of people living with HIV (PLHIV). While many stigma interventions have been carried out worldwide, few focused on both individual and collective empowerment. Community Champions HIV Advocates Mobilization Project (CHAMP) is an intervention study undertaken to mobilize PLHIV and non-PLHIV community leaders in HIV championship in the African, Caribbean, Asian and Latino communities in Toronto, Canada.

**Methods:**

CHAMP tested two anti-stigma interventions: Acceptance and Commitment Training (ACT) that enhances psychological flexibility, and Social Justice Capacity Building (SJCB) that promotes collective empowerment. Participants were randomly assigned to take part in two intervention arms: SJCB only, or ACT plus SJCB. We used focus groups and validated scales to collect data before, immediately after, and 9-month after the interventions. In addition, monthly activity logs were used to capture participants’ post-intervention HIV and social justice championship activities.

**Results:**

A total of 31 non-PLHIV and 35 PLHIV participated in CHAMP. Study results showed significant reduction in felt and enacted stigma in all intervention groups. This paper reports specifically on the impact of CHAMP interventions on PLHIV. Participants reported more self-acceptance; less felt stigma; improved psychological wellbeing; new confidence to speak out against HIV stigma and social injustices; and having stronger social connections. Many had disclosed their HIV status to family and friends, and to leaders at church, college and community. Some took action to pursue their life goals. In addition, PLHIV participants collectively reported a total of 575 activities undertaken at personal, family, organizational and community levels. These activities included: advocating for social justice (n=102); support for PLHIV (n=55); HIV education (n=59); addressing HIV stigma (n=109); community building (n=101); and collective empowerment (n=149). After CHAMP, they have initiated and carried out four participant-driven championship projects.

**Conclusions:**

CHAMP demonstrated that the combined use of psychological and empowerment interventions are powerful in reducing stigma. ACT supported individual empowerment by addressing felt stigma and enhancing self-acceptance; SJCB enabled PLHIV to locate their experience of stigma and discrimination in a collective context and build alliances for change. Coordinated efforts to scale up these interventions will contribute to effective HIV response and PLHIV empowerment.

# Sub article: TUAD0404 HIV vulnerabilities, gender affirmation and social resilience among transgender women in Lima, Peru: a community-based approach to HIV prevention, care and treatment

**Published:** 2016-07-22

**Authors:** A Perez-Brumer, S McLean, A Silva-Santisteban, L Huerta, R de la Grecca, KH Mayer, J Sanchez, JR Lama, SL Reisner

**Affiliations:** 1 Mailman School of Public Health, Columbia University, New York, NY, United States; 2 The Fenway Institute, Fenway Health, Boston, MA, United States; 3 Universidad Peruana Cayetano Heredia, Lima, Peru; 4 Asociación Civil Impacta Salud y Educación, Lima, Peru; 5 Department of Global Health, University of Washington, WA, Seattle, United States; 6 Department of Epidemiology, Harvard T.H. Chan School of Public Health, Boston, MA, United States; 7 Boston Children's Hospital and Harvard Medical School, Boston, MA, United States

## Abstract

**Introduction:**

Transgender women (TW) experience unique vulnerabilities for HIV due to factors that limit access to and quality of services across HIV prevention, treatment and care. Yet, social determinants of HIV disparities remain inadequately understood. Using a strengths-based framework, we assessed HIV vulnerabilities and community-level resilience strategies that buffer against marginalization and oppression, and harness existing supports to link TW to needed HIV-related services in Peru.

**Methods:**

Between January and February 2015, 48 TW participated in a mixed-methods study including focus group discussion and brief survey. Audio files were transcribed verbatim and analyzed using an immersion crystallization approach to identify themes and relationships between themes. Descriptive analyses of survey data were conducted in Stata 13 and qualitative coding using Dedoose Version 6.1.18 (2015).

**Results:**

Among TW (mean age of 29 years) 29% were unsure of their HIV status, over 60% reported sex work as primary income, and 48% reported having ever been arrested. Reported HIV vulnerabilities included: economic (occupation, cost of treatment), social (exclusion, recognition, support) and policy (legal protections, national guidelines). Themes of economic marginalization, multilevel stigma and social recognition of gender identity emerged as salient across vulnerability groupings. Over half (52%) expressed distrust of healthcare providers, and 62% postponed care due to perceived transgender-related stigma. Half reported experiences of discrimination within healthcare settings (e.g. incorrect pronoun, legal versus preferred name). To circumnavigate HIV-service barriers, social resilience strategies emerged within HIV vulnerability domains (e.g. seeking healthcare in groups, using peer health promoters, vetting providers/clinics within social networks). Hormones were critical to affirming gender identity and being socially recognized; however, medical supervision of hormones was rare. Body modification was primarily self- or peer-administered, highlighting the importance of social networks to acquire desired and needed health-related resources and dissemination of peer-to-peer knowledge.

**Conclusions:**

At the intersection of HIV vulnerabilities and collective agency, social resilience emerges as a strategy used by TW to access needed healthcare services in Peru. Fostering TW solidarity is a key component to ensure acceptability and sustainability of gender-affirming HIV interventions. Cross-sex hormone therapy alongside HIV services, peer support, and education represents a community-based, gender-affirmative approach to caring for TW in Peru.

# Sub article: TUAD0405 Changing forms of HIV stigma along the HIV care and treatment cascade: findings from a multisite qualitative study in eastern and southern Africa

**Published:** 2016-07-22

**Authors:** O Bonnington, J Wamoyi, W Ddaaki, D Bukenya, F Odongo, M Skovdal, E McLean, C Nyamukapa, M Moshabela, A Wringe

**Affiliations:** 1 Epidemiology & Population Health, London School of Hygiene & Tropical Medicine, London, United Kingdom; 2 National Institute for Medical Research, Mwanza, United Republic of Tanzania; 3 Rakai Health Sciences Program, Rakai, Uganda; 4 Medical Research Council, Uganda Virus Research Institute, Entebbe, Uganda; 5 Kenya Medical Research Institute, Kisumu, Kenya; 6 University of Copenhagen, Copenhagen, Denmark; 7 London School of Hygiene & Tropical Medicine, Karonga, Malawi; 8 Manicaland Centre for Public Health, Manicaland, Zimbabwe; 9 Africa Centre for Population Health, Mtubatuba, South Africa

## Abstract

**Introduction:**

Despite expanding coverage of HIV care and treatment services, stigma remains pervasive for people living with HIV (PLHIV) in sub-Saharan Africa, undermining engagement in care. We aimed to explore the manifestation of stigma and discrimination at different stages of the HIV care cascade in seven health and demographic surveillance sites (HDSS) in Eastern and Southern Africa.

**Methods:**

Between 2015 and 2016, we conducted 35 in-depth interviews per site in Uganda, South Africa, Tanzania, Kenya, Malawi and Zimbabwe with

1. PLHIV purposively sampled from HIV clinics and HDSS databases linked to HIV clinic records,
2. health providers and
3. family members of people known to have died from HIV.

Topic guides explored patient and provider experiences of HIV testing, care and treatment services. Data were analyzed thematically, aided by NVivo 10 software.

**Results:**

Across all sites, anticipated stigma and discrimination were experienced at different points throughout the cascade. Poor privacy in some HIV testing facilities gave rise to concerns about confidentiality and subsequently fear of stigma. Additionally, powerlessness and coercion within patient-provider relationships often marked the initial cascade stages. To avoid being identified and stigmatized, patients sometimes changed their names when presenting at clinics, posing problems with monitoring of patients for better health outcomes. Non-integrated HIV services sometimes served to exacerbate “othering” of patients, while in settings where services were supposedly integrated people living with HIV (PLHIV) were kept in separate waiting areas, accentuating their differences negatively. Women in prevention of mother-to-child transmission (PMTCT) programmes often feared being questioned about absent partners, and “encouragement” of couple-testing resulted in some people shying away from testing, as did fear of being seen accessing the services by community members. Moreover, many PLHIV took medication in secrecy for fear of exposing their status to partners and others.

**Conclusions:**

Despite efforts to improve HIV care services, stigma remains pervasive across the HIV cascade in all of these sites, though it often manifests in different forms. Context-specific interventions are needed to address stigma and discrimination of PLHIV within the community and in health services, and greater reflection is required to ensure policies aiming to expand HIV treatment do not exacerbate stigma and result in negative HIV outcomes.

# Sub article: TUAE0101 An inferential analysis of the impact of exposure to a peer mentor mother model on uptake of PMTCT services and maternal behavioural outcomes

**Published:** 2016-07-22

**Authors:** S Chapman, N Kayuni Chihana, E Scheepers, K Schmitz

**Affiliations:** mothers2mothers, Department of Programs and Technical Support, Cape Town, South Africa

## Abstract

**Introduction:**

For nearly 15 years, mothers2mothers (m2m) has trained facility-based lay health workers in implementing a peer-to-peer “Mentor Mother” (MM) Model to support prevention of mother-to-child transmission (PMTCT) in sub-Saharan Africa. In 2015, m2m extracted a representative sample of their longitudinal client records to assess the relationship between exposure to facility-based MM visits, and key client health and behavioural outcomes along the PMTCT cascade.

**Methods:**

A stratified random sampling approach was used to identify a representative sample of 87 out of 350 m2m supported facilities in Kenya, Lesotho, Uganda, South Africa, Swaziland and Malawi. A census of longitudinal clients records was taken for clients (n=12 976) first enrolled in m2m care between June and November 2012, and concluding care in December 2014. Exposure status of clients to the m2m model was defined retrospectively by dividing the sample into a low exposure group comprised of clients who had one MM visit after an outcome under investigation had occurred and a high exposure group who had two or more MM visits before an outcome under investigation had occurred. Multivariate regression was then used to explore group differences after adjusting for key confounders.

**Results:**

Women with two or more MM visits were more likely to have an infant who was HIV-negative at the final test (adjusted odds ratio (AOR) =6.4, p<0.001). MM visit exposure status was also positively associated with key behavioural and PMTCT uptake indicators (see Table 1).

**Conclusions:**

Within a longitudinal cohort of clients receiving m2m support, exposure to more MM visits appears to be positively associated with better client behavioural and PMTCT outcomes along the treatment cascade. The study builds a pervasive case for the efficacy of peer-to-peer support models delivered by a paid cadre of lay councillors at the facility level.

**Abstract TUAE0101–Table 1:** Association between MM visit exposure and key behavioural and PMTCT uptake indicators

		Adjusted odds ratios		Unadjusted Frequencies	
			
Indicator		AOR	p	2+m2m visits	1 m2m visit
Maternal Behavioural Outcomes	Using family planning	1.26	0.012	74%	65%
	Exclusive breast feeding first 6 months	1.67	<0.001	84%	75%
Uptake of Maternal PMTCT Services	Antenatal prophylaxis	3.86	<0.001	96%	77%
	Postnatal prophylaxis	2.27	<0.001	86%	74%
Uptake of Infant PMTCT Services	Infant prophylaxis	1.60	0.017	96%	93%
	Infant CPT	1.63	0.001	78%	72%
	6–8 week PCR test	2.31	<0.001	75%	52%
Impact - Mother-to-Child Transmission Rate at 18 Months	Infant HIV status HIV-negative	6.4	<0.001	93%	70%

# Sub article: TUAE0102 Increasing retention of HIV-positive pregnant and postnatal women and HIV-exposed infants: measuring the effects of follow-up activities and improved patient management in rural Uganda

**Published:** 2016-07-22

**Authors:** J Joseph, K Suggu, N Hariharan, G Esiru, E Yavuz, J Gross, B Mirembe

**Affiliations:** 1 Clinton Health Access Initiative, Boston, MA, United States; 2 Ministry of Health, AIDS Control Programme, Kampala, Uganda; 3 Clinton Health Access Initiative, Kampala, Uganda

## Abstract

**Introduction:**

In March 2013, Uganda adopted Option B+, providing lifelong antiretroviral therapy (ART) for all HIV+ pregnant women; however, only 40% of mother-infant pairs were retained in care by the end of the breastfeeding period. Retention must improve in order to achieve Uganda's goal of eliminating mother-to-child-transmission. A pilot was conducted in 2014 to evaluate the effectiveness of a package of interventions consisting of phone and home-visit follow-ups, strengthening the use of appointment books to track attendance, and patient-held appointment calendars. The study's objective was to determine if retention increased for both HIV+ pregnant/postnatal women and HIV-exposed infants (HEI).

**Methods:**

A pre-post study was designed, selecting 20 rural facilities from six districts. Data were collected retrospectively for 6 months prior to and 6 months during the pilot period. Retention was defined as a woman or infant remaining in care for a minimum of 5 months after enrolment into the cohort, determined by the ART visit schedule. Retention rates were assessed by facility using weighted paired t-tests on cluster-level summaries.

**Results:**

A total of 686 women and 358 infants were included in the pre-pilot implementation period, and 604 women and 332 infants during the pilot. Retention in care for mothers increased from 72.8 to 80.3% (p=0.009). This was driven by women who initiated on ART during pregnancy as their retention rates increased from 68.5 to 76.0% (p=0.031). Women under 20 had almost three times more of an increase in retention compared to women of older ages. Retention for HEI increased from 41.3 to 61.1% (p=0.001). Thirty percent of appointments were missed during the pilot programme requiring follow-up, of those missed appointments, 28% received followed-up. There was a 70% return rate for missed appointments relative to a 12% return rate for appointments that received no follow-up.

**Conclusions:**

The results of the pilot showed a significant impact on retention that will result in fewer HIV+ infants and better health outcomes for HIV+ mothers. Despite a low missed visit follow-up rate, when conducted, the impact was substantial. In January 2016 Uganda adopted this model to be the national standard of care for follow-up of mother-infant pairs in all PMTCT sites nationwide.

# Sub article: TUAE0103 Impact of a systems engineering intervention on PMTCT service delivery in Côte d'Ivoire, Kenya, Mozambique: the SAIA cluster randomized trial

**Published:** 2016-07-22

**Authors:** K Sherr, A Silvis Rustagi, S Gimbel, R Nduati, F Cuembelo, A Kone, C Farquhar, J Wasserheit, S Gloyd, C de Schacht, SAIA Study Team

**Affiliations:** 1 University of Washington, Global Health, Seattle, WA, United States; 2 Health Alliance International, Seattle, WA, United States; 3 School of Nursing, University of Washington, Seattle, WA, United States; 4 Network of AIDS Researchers of Eastern and Southern Africa, Nairobi, Kenya; 5 Department of Paediatrics, University of Nairobi, Nairobi, Kenya; 6 Eduardo Mondlane University, Maputo, Mozambique; 7 University of Washington, Seattle, WA, United States

## Abstract

**Introduction:**

Improving prevention of mother-to-child transmission (PMTCT) effectiveness requires increasing the number of women-infant pairs passing through the multiple, sequential steps in the PMTCT cascade, and associated access to efficacious interventions. The Systems Analysis and Improvement Approach (SAIA) trial tested a package of systems engineering techniques to improve cascade flow. Prior systems engineering applications for PMTCT lacked comparison groups or randomization.

**Methods:**

The five-step SAIA intervention addresses cascade inefficiencies through 1) cascade analysis using an automated PMTCT Cascade Analysis Tool (P-CAT) with optimization function to identify largest potential gains across the cascade; 2) process mapping to identify workflow modifications; and 3–5) rapid, iterative testing of workflow modifications. A 9-month cluster randomized trial was conducted in 18 intervention/18 control facilities in Kenya, Côte d'Ivoire and Mozambique, stratified by country and clinic volume. Registry data quantified HIV testing during first antenatal care (ANC) visit, antiretrovirals (ARVs) for HIV-positive pregnant women, and screening HIV-exposed infants (HEI) by 6–8 weeks. Changes between baseline (01/2013–01/2014) and post-intervention (01/2015–03/2015) periods were compared using *t*-tests via intent-to-treat analyses.

**Results:**

Seventeen of 18 intervention facilities accepted the intervention. An average of one cycle was completed monthly falling into five categories: service reorganization; expanding patient knowledge; improving team communication; improving data quality; and introducing new norms or technologies. Examples of service reorganization include increasing blood draw frequency (increasing CD4 testing from 25 to 56%), and reorganizing ANC flow (reducing wait times from 7.5 to 3.5 hours). ARV coverage increased three-fold, and HEI screening increased 17-fold in intervention vs. control facilities, though differences were not significant overall. In pre-specified sub-group analyses, ARV coverage increased significantly in Kenya (+20.9% (95% CI: −3.1 to 44.9) in intervention vs. −21.2% (−52.7 to 10.4) in controls; p=0.02). HEI screening increased significantly in Mozambique (+23.1% (10.3–35.8) in intervention vs. +3.7% (−13.1 to 20.6) in controls; p=0.04). HIV testing did not differ significantly between arms.

**Conclusions:**

This first randomized trial of systems engineering to improve PMTCT saw substantially larger improvements in ARV coverage and HEI screening in intervention facilities compared to controls, which were significant in pre-specified sub-groups. The SAIA intervention was feasible and well-accepted by facility staff. Systems engineering could strengthen PMTCT services and protect infants from HIV.

# Sub article: TUAE0104 Returning HIV-exposed infants to care: results from a pilot integrating infant defaulter tracing into the national Option B+ programme in Lilongwe, Malawi

**Published:** 2016-07-22

**Authors:** E Kamanga, G Banda, I Mofolo, G Mwale, M Mwale, J Chikonda, J Sherman, J Chinkonde, M Herce

**Affiliations:** 1 University of North Carolina Project-Malawi, Lilongwe, Malawi; 2 Ministry of Health, Lilongwe District Health Office, Lilongwe, Malawi; 3 HIV and AIDS Section, UNICEF, Lilongwe, Malawi; 4 Division of Infectious Diseases, Department of Medicine, University of North Carolina, Chapel Hill, NC, United States

## Abstract

**Introduction:**

Despite high uptake of prevention of mother-to-child transmission (PMTCT) services early in the care continuum in Malawi's Option B+ Programme, including HIV counselling and testing (90%) and antiretroviral therapy (ART, 79%), challenges remain with the post-natal care continuum, including high loss to follow-up (40% by 24 months of age) for HIV-exposed infants (HEIs). Poor HEI care retention undermines early diagnosis and treatment of HIV-infected infants, and threatens progress in reducing vertical HIV transmission in Malawi and elsewhere in sub-Saharan Africa. In response to this challenge, in October 2013 we launched an HEI “defaulter” tracing programme at 20 health facilities in Lilongwe selected for high HEI census as part of the UNICEF Optimizing HIV Treatment Access (OHTA) initiative to increase timely uptake, adherence and retention along the PMTCT care continuum.

**Methods:**

We trained and mentored 737 community-based Health Surveillance Assistants (HSAs) from Ministry of Health (MOH) to perform tracing via phone contact and/or home visit. HSAs were mentored on accurate reporting using data collection tools from the MOH National HEI Follow-Up Programme, as well as OHTA-specific defaulter-tracing and HEI appointment registers. To support physical tracing, we provided 30 bicycles to health facilities and all HSAs with a modest monetary incentive (~$2 USD) for every HEI successfully reached.

**Results:**

From October 2013 to —September 2015, we traced 2707 HEIs who had fallen out of care (i.e. missing a scheduled follow-up appointment by≥14 days). Of these, 2078 HEIs (76.8%) were successfully reached by phone or home visit. Following tracing, 1969 of 2078 reached HEIs (94.8%) returned to clinic and were retained in care as assessed at 2, 12 or 24 months of age (depending on HEI age at the time of tracing). Of these, 50 HIV-infected infants were identified (2.5%, 50/1969) and all initiated ART (100%, 50/50).

**Conclusions:**

Integrating HEI defaulter tracing into the public health system facilitated improved HIV care retention for HEIs, and successful HIV diagnosis and ART initiation for HIV-infected infants. Such an approach, implemented by community agents receiving mentorship, incentives and field supervision, may be a strategy to strengthen HEI care retention and the post-natal PMTCT care continuum in Malawi.

# Sub article: TUAE0105 A multipronged approach to the elimination of MTCT in South Africa

**Published:** 2016-07-22

**Authors:** K Ng'oma, T Mtileni, I Odongo, P Robinson, M Mogashoa, S Bhardwaj, P Holele, Y Pillay

**Affiliations:** 1 UNICEF, Pretoria, South Africa; 2 National Department of Health, Pretoria, South Africa; 3 mothers2mothers, Pretoria, South Africa; 4 Right to Care, Johannesburg, South Africa; 5 CDC, Pretoria, South Africa

## Abstract

**Introduction:**

South Africa has the highest number of people living with HIV in the world, estimated at 6.4 million in 2012, with HIV prevalence of 29.5% in antenatal women, and mother-to-child transmission (MTCT) rate estimated at 3.5% at 6 weeks in 2010. In response to the Global Plan *towards the elimination MTCT* (EMTCT), the National Department of Health (NDOH) developed and implemented the national elimination MTCT Action Framework entitled “No child born with HIV by 2015 and improving the health and wellbeing of mothers, partners and babies in South Africa.” The framework enabled evidence-based, accelerated, programme scale-up and delivery of quality services, with innovative data-driven action plans in all provinces and districts.

**Description:**

Five key strategic pillars were identified for scaling up quality integrated prevention of MTCT services. Political leadership and commitment at the highest level resulted in accelerated national HIV response, including EMTCT. Responsive changes in policy, for example, the move from single dose nevirapine to more efficacious triple antiretroviral (ART) regimens for prevention of mother-to-child transmission (PMTCT), coupled with quality improvement initiatives and task shifting, resulted in rapid scale-up of quality EMTCT services. PMTCT was integrated into the maternal, child and women's health programme to maximize service delivery platforms. The routine use of “robot” dashboards and data for action reports ensured continuous monitoring of programme performance, and action planning. Significant progress was made towards targets of the plan. The number of children newly infected with HIV in South Africa declined by over 70% (2009–2014). Over 90% of HIV positive women were receiving treatment for PMTCT (2015), a significant increase from 63% in 2009. The coverage of early infant diagnosis of HIV increased from 45% in 2009 to 87% in 2014, MTCT rate declined to 2.6% in 2012/2013. Infant HIV positivity rates at around 6 weeks declined from 5.8% in 2009 to 1.5% in 2015.

**Lessons learned:**

High level political leadership, strong partnerships and robust monitoring and evaluation systems helped to accelerate response to elimination of MTCT.

**Conclusions/Next steps:**

An evidence-based multipronged approach was critical for the success seen in the journey towards the elimination of MTCT in South Africa.

# Sub article: TUAE0106 Highest risk of mother to child transmission of HIV or death in the first 6 months postpartum: results from 18 month follow-up of an HIV-exposed national cohort, South Africa

**Published:** 2016-07-22

**Authors:** A Goga, D Jackson, C Lombard, V Ramokolo, N Ngandu, G Sherman, A Puren, T Doherty, S Bhardwaj, N Noveve, T Ramraj, V Magasana, Y Singh, Y Pillay, South African PMTCT Evaluation Study Group

**Affiliations:** 1 South African Medical Research Council, Pretoria, South Africa; 2 Department of Paediatrics, University of Pretoria, Pretoria, South Africa; 3 UNICEF, New York, South Africa; 4 University of the Western Cape, Cape Town, South Africa; 5 South African Medical Research Council, Cape Town, South Africa; 6 University of Cape Town, Cape Town, South Africa; 7 University of Witwatersrand, Johannesburg, South Africa; 8 National Institute of Communicable Diseases, Johannesburg, South Africa; 9 National Institute of Communicable Diseases, Johannesburg, South Africa; 10 UNICEF, Pretoria, South Africa; 11 South African Medical Research Council, Durban, South Africa; 12 South African National Department of Health, Pretoria, South Africa

## Abstract

**Introduction:**

Few resource-limited, high HIV prevalence settings produce data on national 18–24 month infant “mother-to-child transmission of HIV (MTCT)-or-death” – the gold standard measurement of programme impact. We studied South African national MTCT and “MTCT-or-death” when health policy provided infant nevirapine during breastfeeding (Option A) and changed to triple antiretroviral therapy for all HIV infected women during pregnancy and lactation (Option B).

**Methods:**

A nationally representative cross-sectional survey was conducted to estimate early (4–8 weeks postpartum) MTCT. Facilities (n=580) were randomly selected following multistage probability proportional to size sampling methodology. Consenting caregivers of systematically or consecutively sampled infants (4–8 weeks old) receiving their 6 week immunization were interviewed. Infant dried blood spot specimens (iDBS) were drawn and tested for HIV exposure and, if positive, for infection (total nucleic acid polymerase chain reaction, or TNA PCR). Then, all HIV exposed infants (antibody, or maternal self-reported positive) were invited for facility-based follow-up at 3, 6, 9, 12, 15 and 18 months. At each follow-up visit, caregivers were interviewed and infants were tested for HIV infection. Analysis was weighted for sample ascertainment, population live births, consent to follow-up (if eligible) and loss to follow-up.

**Results:**

Analysis of 9120 iDBS at 4–8 weeks revealed 33.1% infant HIV exposure (95% Confidence Interval, 31.8–34.3%) and 2.6% (2.0–3.2) MTCT. In total, 1880 (71%) HIV-exposed infants (HEIs) were followed up at 18 months. Cumulative MTCT and “MTCT-or-death” by 3, 6, 9, 12, 15 months was 2.7% (2.6–12.6) and 2.8% (2.6–19); 3.5% (3.1–4.4) and 4.2% (3.5–5.4); 3.7% (3.2–4.6) and 5.1% (4.4–6.2); 3.9% (3.4–4.7) and 5.7% (5.0–6.8); 4.1% (3.5–4.8) and 6.0% (5.2–7.0), and at 18 months, 4.3% (3.7–5.0) and 6.2% (5.5–7.3) respectively. Eighty-one percent of MTCT and 67% of “MTCT-or-death” occurred by 6 months postpartum. Maternal receipt of CD4-cell-count result and avoiding breastfeeding protected against MTCT (Adjusted hazard ratio HRa, 0.3 (0.2–0.6), and 0.3, (0.07–0.9), respectively). Mixed feeding and infant nevirapine did not significantly increase MTCT-or-death (HRa 1.4 (0.8–2.4) and 2.1 (0.8–5.4), respectively). Having a refrigerator significantly protected against MTCT-or-death (HRa 0.5 (0.3–1.0), respectively).

**Conclusions:**

The first 6 months postpartum is a critical period for following up HEIs and providing regular HIV testing.

# Sub article: TUAX0101LB Effect of vorinostat, hydroxychloroquine and maraviroc combination therapy on viremia following treatment interruption in individuals initiating ART during acute HIV infection

**Published:** 2016-07-22

**Authors:** E Kroon, J Ananworanich, K Eubanks, J Intasan, S Pinyakorn, N Chomont, SR Lewin, S Palmer, L Trautmann, H Yang, N Chomchey, N Phanuphak, K Cooper, P Phanuphak, M de Souza, on behalf of the SEARCH 019 study group

**Affiliations:** 1 SEARCH, Thai Red Cross AIDS Research Centre, Bangkok, Thailand; 2 Walter Reed Army Institute of Research, U.S. Military HIV Research Program, Bethesda, MD, United States; 3 Henry M. Jackson Foundation for the Advancement of Military Medicine, Bethesda, MD, United States; 4 Cooper Human Systems, Cambridge, MA, United States; 5 CRCHUM and Department of Microbiology, Infectiology and Immunology, Universite de Montreal, Montreal, Canada; 6 The Peter Doherty Institute for Infection, University of Melbourne, Melbourne, Australia; 7 The Royal Melbourne Hospital, Melbourne, Australia; 8 The University of Sydney, Sydney, Australia; 9 The Westmead Institute for Medical Research, Sydney, Australia; 10 HIV-NAT, Thai Red Cross AIDS Research Centre, Bangkok, Thailand

## Abstract

**Introduction:**

Individuals who initiate antiretroviral therapy (ART) during acute HIV infection (AHI) have a lower frequency of latently infected cells and could have a greater chance for viremic control after treatment interruption (TI).

**Methods:**

A randomized study of vorinostat/hydroxychloroquine/maraviroc (VHM, n=10; 8 Fiebig III/2 Fiebig IV) plus ART versus ART alone (n=5; all Fiebig III) given for 10 weeks, followed by TI at week 10 was conducted in individuals treated since AHI with viral load (VL) suppression for >48 weeks and CD4 ≥450 cells/mm^3^. The VHM arm received three cycles of vorinostat 400 mg/day (14 days on/14 days off) plus hydroxychloroquine (400 mg/day) and maraviroc (1200 mg/day). VL was monitored weekly after TI. ART was resumed when confirmed VL >1000 copies/ml.

**Results:**

The participants were mainly male who were treated during Fiebig III AHI with high CD4 and about 3 years of VL suppression. Two individuals in the VHM arm had serious adverse events, and one withdrew from the study for renal insufficiency and thrombocytopenia.

Fourteen participants underwent TI (9 VHM+ART, 5 ART) and all experienced VL rebound with no difference between arms (range: 2–11 weeks). One participant in the ART arm had viremic control for 11 weeks. None had acute retroviral syndrome. All achieved VL suppression following ART, and there was no change in genotypic resistance profile.

**Conclusions:**

In this proof-of-concept study, all 14 individuals who initiated ART during Fiebig III/IV AHI experienced VL rebound following treatment interruption regardless of VHM treatment.

**Figure:** Abstract TUAX0101LB–Viral load kinetics following treatment interruption in VHM + ART vs. ART arms

**Table:** Abstract TUAX0101LB–Characteristics at randomization and after TI in VHM + ART vs. ART arms

Characteristics, median (range)	VHM + ART	ART
Age at randomization, years/gender	28 (22–51)/9 male: 1 female	26 (24–34)/4 male: 1 female
ART duration, weeks	224 (79–294)	155 (100–295)
CD4 count, cells/mm^3^	634 (501–1106)	1079 (537–1612)
Total HIV DNA (copies/10E6 PBMCs)	837 (0–2323)	594 (19–1878)
POST-TREATMENT INTERRUPTION		
Time from treatment interruption to VL detection, weeks	3 (2–5)	3.1 (3–11)
VL levels at first detection, copies/ml	222 (33–41,822)	156 (52–395)
Time from first VL detection to ART resumption, weeks	1 (0.1–4.1)	2 (1–5.3)
Time from ART resumption to VL suppression, weeks	2.9 (0.9–10.9)	2 (1.9–3.9)

# Sub article: TUAX0102LB Meeting the “Go” criteria: immunogenicity from HVTN100, a phase 1/2 randomized, double blind, placebo-controlled trial of clade C ALVAC-^®^ (vCP2438) and Bivalent Subtype C gp120/MF59^®^ in HIV-uninfected South African adults

**Published:** 2016-07-22

**Authors:** L-G Bekker, F Laher, Z Moodie, G Tomaras, N Grunenberg, M Allen, B Daniels, C Innes, K Mngadi, M Malahleha, P Gilbert, N Michael, S Phogat, C Diaz Granados, N Kanesa-Thasan, L Corey, G Gray, J McElrath, The HVTN100 Study Team

**Affiliations:** 1 The Desmond Tutu HIV Centre, University of Cape Town, Cape Town, South Africa; 2 The Perinatal HIV Research Unit, Johannesburg, South Africa; 3 Fred Hutchinson Cancer Research Centre, Seattle, WA, United States; 4 Duke Human Vaccine Institute, Durham, NC, United States; 5 HIV Vaccine Trials Network, Seattle, WA, United States; 6 National Institutes of Health, Bethesda, MD, United States; 7 South African Medical Research Council, Durban, South Africa; 8 Aurum Institute, Klerksdorp, South Africa; 9 CAPRISA, Durban, South Africa; 10 Setshaba Research Centre, Pretoria, South Africa; 11 Fred Hutchinson Cancer Research Centre, Seattle, WA, South Africa; 12 Walter Reed Army Institute of Research, Washington, DC, United States; 13 Sanofi Pasteur, Swift water, PA, United States; 14 Glaxo Smith Kline Biologicals, Cambridge, South Africa; 15 The Fred Hutchinson Cancer Research Centre, Seattle, WA, United States; 16 The South African Medical Research Council, Cape Town, South Africa

## Abstract

**Introduction:**

The RV144 ALVAC-HIV/AIDSVAX^®^ B/E/alum HIV vaccine trial conducted in Thailand demonstrated 31% vaccine efficacy at 3.5 years. Following RV144, vaccine components were modified to express HIV-1 antigens matched to circulating clade C strains, the adjuvant was changed to MF59 and a booster immunization was added. The vaccine regimen of clade C ALVAC-HIV (strains ZM96 and LAI) and Bivalent Subtype C gp120 (strains 1086.C and TV-1) /MF59 is being tested in a phase 1/2 trial, and HVTN100 is tested in 6 South African clinical trial sites. Archived RV144 samples were contemporaneously compared to vaccine-induced immune responses in HVTN100 samples. Four pre-specified immune criteria associated with vaccine take; potency and correlates of risk in RV144 guided the decision about whether or not to proceed to a phase 2b efficacy trial.

**Methods:**

Fifty-two HIV-uninfected adults (43% female) were enrolled and randomly assigned to receive vaccine (n=210) or placebo (n=42). Humoral and cellular responses were measured 2 weeks after the 6-month vaccination (ALVAC-HIV/Bivalent Subtype C gp120/MF59 boost) in HVTN100 (185 vaccine/37 placebo) and contemporaneously assayed RV144 (201 vaccine/24 placebo) samples from per-protocol participants. Twelve-month booster vaccinations are currently ongoing.

**Results:**

No safety concerns were identified. 100% of HVTN100 vaccine-recipients developed IgG binding antibodies to all three clade C gp120 vaccine-matched envelope insert antigens with significantly higher titres (3.6–8.8 fold, p<0.001) than in RV144 to the corresponding RV144 vaccine-matched antigens. CD4 T cell response rate to the ALVAC ZM96 envelope antigen in HVTN100 was 57.5% versus 41.4% to 92TH023 in RV144 (p=0.002), with a significantly greater 5-function poly-functionality score in HVTN100 (p<0.001). 80% (95% CI: 74.0%–85.4%) of participants in HVTN100 demonstrated an IgG response to at least one of the three vaccine-matched V1V2 antigens, above 63% threshold needed to predict 50% vaccine efficacy in a phase 2b trial under a V1V2 correlate of protection model.

**Conclusions:**

Cellular and humoral immune responses in HVTN100 met pre-specified criteria, supporting future evaluation in a phase 2b vaccine efficacy trial. This will also be critical for defining relevant correlates of protection of this regimen in Southern African.

# Sub article: TUAX0103LB Results of the SAPPH-IRe trial: a cluster randomized trial of a combination intervention to empower female sex workers in Zimbabwe to link and adhere to antiretrovirals for treatment and prevention

**Published:** 2016-07-22

**Authors:** F Cowan, C Davey, P Mushati, S Mtetwa, T Chiyaka, S Chabata, E Fearon, S Napierala Mavedzenge, V Cambiano, N Masuka, M Chemhuru, J Dirawo, D Hanisch, K Hatzold, O Mugurungi, J Busza, A Phillips, J Hargreaves

**Affiliations:** 1 University College London, London, United Kingdom; 2 CeSHHAR Zimbabwe, Harare, Zimbabwe; 3 London School of Hygiene and Tropical Medicine, London, United Kingdom; 4 RTI International, Women's Global Health Imperative, San Francisco, CA, United States; 5 Ministry of Health and Child Care, Harare, Zimbabwe; 6 United Nations Population Fund, Harare, Zimbabwe; 7 PSI Zimbabwe, Harare, Zimbabwe

## Abstract

**Introduction:**

Female sex workers (FSW) are often poorly engaged with HIV prevention and care. We conducted a cluster-randomized trial of combination prevention to empower female sex workers (SAPPH-IRe) embedded within Zimbabwe's National FSW Program (“Sisters”).

**Methods:**

We randomly allocated 14 clusters in matched-pairs to “usual care” (*Sisters*) or to SAPPH-IRe. A cluster was defined as the FSW population working around a clinic providing the services listed below.

Usual care *Sisters'* programme: sex-worker-friendly services, free HIV testing, referral to government health services for ART, contraception, condoms, STI syndromic management, health education and legal advice; all supported by peer educators.

SAPPH-IRe: the usual-care *Sisters* programme plus intensified community mobilization; provision of onsite ART; SMS reminders to promote repeat testing for HIV negative, pre-exposure prophylaxis for HIV negative and community-based adherence support to build a “sisterhood” to improve engagement with intensified prevention and care.

The primary outcome was the proportion of all FSW with detectable viral load (VL>1000 copies/ml) after 21 months.

A baseline survey was completed in November 2013 (n=2722 FSW, 57.5% HIV-positive, 50.5% HIV-positive and with VL>1000 copies/ml) and a separate endline survey in April 2016, both using respondent-driven sampling. Recruitment to the surveys was not linked to participation in the interventions since the aim was to assess the population impact of SAPPH-Ire. Pre-specified analyses are underway. Secondary outcome and process data were also collected.

**Results:**

The SAPPH-IRe intervention was implemented from May 2014 to March 2016. The table compares programme activity by arm. 2883 FSWs participated in the endline survey, providing questionnaire data and dried-blood spot (n=2876) for HIV antibody and VL testing which is now complete.

**Conclusions:**

The *SAPPH-IRe* intervention resulted in increased programme activity and provided on-site ART and PrEP. The endline results to be reported at conference will tell us the community level impact on the proportion of FSW with VL >1000 copies/ml.

**Table:** Abstract TUAX0103LB–Programme data May 2014–Mar 2016

	SAPPH-IRe sites	Usual Care Sisters sites
# community mobilisation meetings	537	145
# peer educator contacts	17,013	13,151
# FSW seen	4549	3574
# FSW clinic attendances	12,905	10,031
# HIV tests	2606	1151
# ART on-site initiations	768	0
# On-site PrEP initiations	487	0
# Adherence sisters paid	514	0

# Sub article: TUAX0104LB An HIV pre-exposure prophylaxis demonstration project and safety study for adolescent MSM aged 15–17 in the United States (ATN 113)

**Published:** 2016-07-22

**Authors:** S Hosek, R Landovitz, B Rudy, B Kapogiannis, G Siberry, B Rutledge, N Liu, J Brothers, J Rooney, CM Wilson, The Adolescent Trials Network for HIV/AIDS Interventions (ATN)

**Affiliations:** 1 Department of Psychiatry, Stroger Hospital of Cook County, Chicago, IL, United States; 2 University of California Los Angeles, Los Angeles, CA, United States; 3 New York University Medical Center, New York, NY, United States; 4 NICHD/MPIDB, Bethesda, MD, United States; 5 Westat, Rockville, MD, United States; 6 Gilead Sciences, Foster City, CA, United States; 7 University of Alabama at Birmingham, Birmingham, AL, United States

## Abstract

**Introduction:**

Adolescents represent a key population for implementation of pre-exposure prophylaxis (PrEP) interventions worldwide, yet TDF/FTC PrEP is not currently licensed under age 18. This open-label PrEP study examined safety of and adherence to PrEP along with changes in sexual risk behaviour among adolescent MSM in six US cities.

**Methods:**

ATN 113 (Project PrEPare) combined PrEP with behavioural risk reduction and adherence support. HIV-uninfected MSM aged 15–17 who reported HIV risk behaviour in the past 6 months were eligible. Youth were allowed to autonomously consent to study participation. Study visits occurred at baseline, monthly through week 12, then quarterly through week 48. Serial tenofovir diphosphate (TFV-DP) levels in dried blood spots (DBS) were used to measure adherence.

**Results:**

Between August 2013 and September 2014, 2864 individuals were approached, 260 (9%) were preliminarily eligible, and 78 were enrolled (mean age=16.5; 33.3% Mixed race, 29.5% Black, 20.5% Latino). Baseline STIs were diagnosed and treated in 15.4% of participants. Incident STIs were diagnosed in 12.3% of participants at week 24 and 10.6% at week 48. The HIV seroconversion rate per 100 person-years was 6.41 (95% CI: 4.90–25.87). Condomless sex was reported by the majority of participants throughout the study; no significant associations were found between condomless sex and adherence. Figure 1 shows TFV-DP levels. Non-adherent participants were significantly more likely than adherent participants to report worry that others would think they had HIV if they saw their PrEP pills (p=0.03).

**Conclusions:**

ATN 113 enrolled a diverse sample of YMSM at risk for HIV who self-consented to study participation. The majority of participants achieved protective drug levels during monthly visits, yet adherence decreased with quarterly visits. HIV incidence was still high despite PrEP provision, suggesting high background incidence. Regulatory approvals for youth under 18 years are required to foster support for youth-friendly settings that will optimize PrEP use.

**Abstract TUAX0104LB–Figure 1:** Tenofovir diphosphate levels (fmol/punch) and PrEP dosing estimates via DBS.

# Sub article: TUAX0105LB Truvada for HIV pre-exposure prophylaxis utilization in the United States (2013–2015)

**Published:** 2016-07-22

**Authors:** R Mera, S McCallister, B Palmer, G Mayer, D Magnuson, K Rawlings

**Affiliations:** 1 Epidemiology, Gilead Sciences, Foster City, CA, United States; 2 Clinical Research, Gilead Sciences, Foster City, CA, United States; 3 Medical Sciences, Gilead Sciences, Foster City, CA, United States; 4 Drug Safety & Public Health, Gilead Sciences, Foster City, CA, United States; 5 Medical Affairs, Gilead Sciences, Foster City, CA, United States

## Abstract

**Introduction:**

In 2012 Truvada (TVD) was licensed for pre-exposure prophylaxis (PrEP) to reduce the risk of sexually acquired HIV-1 for adults at high risk in the US.

We describe the TVD for PrEP utilization in the US, including user and prescriber characteristics.

**Methods:**

We used nationally representative (82% of retail pharmacies) de-identified data (Symphony Health Analytics) on individuals who received a TVD prescription from January 2013 to December 2015. The data warehouse contains medical claims, diagnosis codes and patient and provider demographics. A validated algorithm was used to quantify TVD for PrEP use by excluding TVD for HIV treatment, HIV post-exposure prophylaxis and chronic Hepatitis B treatment. Logistic regression analyses were used to compare demographic data from TVD use for HIV treatment or PrEP.

**Results:**

Between January 2013 and December 2015, 49,469 unique individuals started TVD for PrEP: 3746 in 2013; 14,756 in 2014; 30,967 in 2015. 87.5% were male. Mean age was 37.4 years, with 11.5% under age 25. Figure 1 shows regional distribution of TVD PrEP starts over time. There were 19,274 prescribers across 50 states. Four states (CA, NY, TX, FL) with the highest HIV incidence account for 43.0% of PrEP starts. Compared to HIV positive patients, uninfected individuals receiving TVD for PrEP were 3.1 times less likely to be female (95% CI: 3.0–3.2), but 1.98 times more likely to be under age 25 (95% CI: 1.93–2.05).

**Conclusions:**

The number of individuals starting on TVD for PrEP has increased nationally since the 2012 approval in the US and is primarily male. States with the highest number of new HIV cases also had the highest number of TVD PrEP starts. Despite positive trends in TVD for PrEP use, utilization must increase to ensure lifetime risk seroconversion decreases in areas of high prevalence HIV in the US.

**Abstract TUAX0105LB–Figure 1:** Distribution by region over time.

# Sub article: WEAA0101 Th17 cells are preferentially infected in the first 48 hours after vaginal transmission of SIVmac239 in macaques

**Published:** 2016-07-22

**Authors:** DJ Stieh, E Matias, AJ Fought, PA Marx, RS Veazey, TJ Hope

**Affiliations:** 1 Cell and Molecular Biology, Northwestern University, Chicago, IL, United States; 2 Preventative Medicine, Northwestern University, Chicago, IL, United States; 3 Pathology, Tropical Medicine, Tulane University, New Orleans, LA, United States; 4 Tulane National Primate Research Center, Covington, KY, United States; 5 Cell and Molecular Biology, OB/GYN, Biomedical Engineering, Northwestern University, Chicago, IL, United States

## Abstract

**Introduction:**

Macaque vaginal challenge with SIV is utilized to reproduce the circumstances of male-to-female HIV transmission. This model has provided insights into HIV vaginal transmission, but the critical window of the earliest events taking place after mucosal exposure remains undefined.

**Methods:**

We have recently developed a SIV-based dual reporter expression vector that facilitates the efficient identification of transmission susceptible sites in the rhesus macaque female reproductive tract (FRT) after vaginal exposure. This system demonstrated that initial infection events can be widespread throughout the FRT, highly variable in their localization, and that T cells are the primary target in initial infection. Because this system efficiently identifies regions of susceptibility to infection in the FRT, we have determined that we can identify small foci of SIVmac239 infection 48 hours after vaginal challenge with a mixture of wildtype SIVmac239 and the LICh dual reporter. Utilizing this novel approach to SIV challenge, we routinely identify SIVmac239 infected cells revealing their localization and fates in the FRT 48 hours after vaginal challenge.

**Results:**

Foci of infection with SIVmac239 are found throughout the FRT, from labia to ovary. We find that T cells are the major targets, and there is a strong bias for those with a Th17 phenotype. Infection of immature dendritic cells and macrophages is also observed representing approximately 25% of infected cells. 48 hours post inoculation, we find host responses to infection, evidenced by apoptosis, cell lysis, and phagocytosis of infected cells. RNA-Seq profiling of gene expression in tissues where SIV infection was established indicates that inflammatory responses and epithelial repair processes are occurring.

**Conclusions:**

Defining the location and phenotype of SIV infected cell foci and early host responses informs the development of interventions designed to decrease HIV acquisition. Preferential infection of Th17 cells could explain the known conditions that increase HIV acquisition, including sexually transmitted infections and bacterial vaginosis. How these conditions precisely influence mucosal barrier function or the density of target cells remains to be determined. However, the system presented here provides essential sampling of these foci, facilitating characterization of the earliest host responses to SIV/HIV infection.

# Sub article: WEAA0102 Effect of injectable hormonal contraceptives on vaginal epithelium thickness and genital HIV target cell density in women recently infected with HIV

**Published:** 2016-07-22

**Authors:** S Ngcapu, AM Carias, LJ Liebenberg, L Werner, GC Cianci, M McRaven, S Sibeko, NJ Garrett, J-M Kriek, LR McKinnon, S Abdool Karim, Q Abdool Karim, J-AS Passmore, TJ Hope

**Affiliations:** 1 Centre for the AIDS Programme of Research in South Africa (CAPRISA), Durban, South Africa; 2 Northwestern University, Chicago, IL, United States; 3 Oxford University, London, United States; 4 Institute of Infectious Diseases and Molecular Medicine, University of Cape Town Medical School, Cape Town, South Africa; 5 Department of Epidemiology, Mailman School of Public Health, Columbia University, New York, NY, United States; 6 National Health Laboratory Service, Cape Town, South Africa

## Abstract

**Introduction:**

Vaginal epithelial thinning and/or increased density of mucosal HIV-1 target cells are possible mechanisms by which injectable hormonal contraceptives (HCs) may increase risk for HIV-1 infection in HIV-1 negative women and the risk of her transmitting to her partner if infected. Here, the influence of injectable HCs on genital epithelial thickness, mucosal HIV-1 target cell density and depth in women with acute HIV infection was investigated.

**Methods:**

CD4+ T cell and CD68+ macrophage density, both target cells for HIV infection, was measured by immunofluorescent staining in vaginal tissue biopsies from acutely-infected women who were either using injectable HCs or not using contraception. Concentrations of 48 cytokines measured in cervico-vaginal lavage (CVL). Blood CD4 counts and plasma viral loads were performed during acute infection and 12 months post-infection.

**Results:**

Vaginal epithelial thickness was similar in women using injectable HCs compared to non-injectable HC users. The frequency of CD4+ T cells in the vaginal squamous epithelium of injectable HC users was significantly higher than non-injectable HC users (p=0.028). CD68+ macrophage cell density did not differ between women using injectable HCs and those not using injectable HCs, although macrophages were closer to the vaginal luminal surface in injectable HC users than those not using HCs (p=0.021). Furthermore, the frequency of mucosal CD68+ macrophages during the acute infection were positively associated with the concentration of the RANTES (beta coefficient (β)=0.779, p=0.024), MCP-1 (β=0.453, p=0.041), IP-10 (β=0.568, p=0.042), IL-7 (β=1.332, p=0.018), IL-9 (β=0.336, p=0.015), and IL-17 (β=1.058, p=0.007) in CVL, after adjusting for multiple comparisons.

**Conclusions:**

Women using injectable HC users had increased frequencies of CD4+ T cells in their vaginal stratified epithelium than those not using injectable HCs. CD68+ macrophages correlated with a broad panel of mucosal cytokines. This study provides valuable insight into possible underlying mechanisms by which genital inflammation may increase HIV-1 risk and subsequent clinical phenotypes during HIV-1 disease course, such as viral set point.

# Sub article: WEAA0103 Characterization of early events of SIVagm dissemination following intrarectal inoculation in adult AGMs

**Published:** 2016-07-22

**Authors:** K Raehtz, G Haret-Richter, C Ling Xu, B Policicchio, C Barrenas, D Ma, V Wijewardana, E Brocca-Cofano, J Stock, A Trichel, B Keele, J Estes, M Katze, I Pandrea, C Apetrei

**Affiliations:** 1 University of Pittsburgh, Center for Vaccine Research, Pittsburgh, PA, United States; 2 University of Washington, Department of Microbiology, Seattle, WA, United States; 3 Veterinary Medicine, University of Pittsburgh, Pittsburgh, PA, United States; 4 National Laboratory for Cancer Research, Frederick, MD, United States

## Abstract

**Introduction:**

African green monkeys (AGMs) do not normally progress to AIDS. Instead, they maintain a lifelong infection in spite of high viral replication. We investigated the impact of early virus replication and dissemination on the outcome of SIVsab infection in AGMs.

**Methods:**

Twenty-nine adult male AGMs were intrarectally inoculated with SIVsab and serially sacrificed at 1–12 and 42+ days postinfection (dpi). Virus spread was monitored by PCR. vRNA and vDNA were quantified in 38 different tissues from each animal, including the site of inoculation. Plasma viral loads were quantified by standard RT-PCR and single copy assay (SCA). The PCR results were confirmed by extensive *in situ* hybridizations. Single genome amplification was performed to assess the bottleneck of SIV transmission.

**Results:**

Plasma viremia was detectable as early as 2 dpi by SCA and 6 dpi by conventional PCR. vRNA and vDNA were detectable at the site of entry and draining LN as early as 1–3 dpi, in PBMCs at 3–4 dpi, in peripheral gut and lymphatics at 4–6 dpi and all other tissues 6 dpi. The highest levels of both vDNA and vRNA were found at the site of entry, LNs, peripheral gut and spleen. Multiple transmitted/founder viral variants were detected in all animals.

**Conclusions:**

Early virus enrichment occurred at the site of entry, where the virus became detectable by 1–3 dpi. Plasma virus also became detectable very early, which indicates that the initial viral expansion and dissemination were nearly simultaneous. Furthermore, virus was detected early in the distal LNs, indicating rapid viral dissemination through the lymphatic system, in addition to the bloodstream. Multiple transmitted/founder viruses were identified in all animals, demonstrating that there is little bottleneck of virus transmission. These results are similar to the early viral dynamics of rectal transmission in adult male rhesus macaques, indicating that following rectal transmission there is little opportunity to prevent virus spread.

# Sub article: WEAA0104 Early treatment of hyperacute HIV infection impacts phenotype and clonal repertoire of HIV-specific CD8^+^ T cells

**Published:** 2016-07-22

**Authors:** Z Ndhlovu, T Nkosi, N Mewalal, N Ismail, A Moodley, K Dong, T Ndun'gu, BD Walker

**Affiliations:** 1 Ragon Institute of MGH, MIT and Harvard, Harvard University, Cambridge, United States; 2 HIV Pathogenesis Programme, Nelson Mandela School of Medicine, University of KwaZulu Natal Durban, South Africa

## Abstract

**Introduction:**

Although natural immunity in some cases can lead to prolonged HIV suppression, it does not completely eliminate the virus. Consequently, most of what we know regarding the nature of HIV-specific responses is based on inadequate responses generated in the setting of high levels of persistent plasma viremia and marked CD4 cell decline in acute infection. We investigated the impact of antigen withdrawal through very early treatment of hyperacute infection on the functional qualities of HIV-specific CD8^+^ T cell responses.

**Methods:**

Ten subjects who initiated ART in Fiebig stage 1 and 12 subjects with untreated hyperacute HIV infection (UTx) were studied. We conducted a comparative longitudinal analysis of the clonality, phenotype and functional profile of HIV-specific CD8^+^ T cell responses generated during treated and untreated hyperacute infection. HIV-specific CD8^+^ T cells were measured using MHC class I tetramers. T cell receptors (TCRs) were sequenced from tetramer sorted CD8^+^ T cells.

**Results:**

In spite of rapid plasma virus suppression and blunted peak viremia, HIV-specific CD8^+^ T cell responses were detected in 7 of 10 (70%) ETx subjects studied, compared to 90% detection rate in UTx. Phenotypic analysis of tetramer^+^ cells showed that responses in ETx subjects expressed higher levels of interleukin-7 receptor alpha (CD127^+^), a marker associated with the development of long-term memory, compared to untreated subjects (p*=*0.0001). ETx responses were more fully differentiated with terminally differentiated effector cells account for the 90% of the responses (p*=*0.0001), whereas untreated responses were less differentiated, with effector cells account for 90% of the response (p*=*0.0001). Combined tetramer ICS staining of 2 ETx had >70% tetramer+ cells secreting IFN-g compared to <20% in UTx. Furthermore, longitudinal TCR analysis of tetramer sorted cells obtained from ETx revealed striking clonal stability over time, whereas UTx responses were characterized by successive waves of clonal loss and emergency of new clonotypes over time.

**Conclusions:**

We show that very early ART is associated with measurable CD8^+^T cell responses that are phenotypically and functionally superior to untreated hyperacute HIV infection. Our data suggest that prompt curtailment of HIV replication results in more functionally competent immune responses with potential for long-term survival.

# Sub article: WEAA0105LB Exhaustion of activated CD8 T cells predicts disease progression in primary HIV-1 infection

**Published:** 2016-07-22

**Authors:** GE Martin, N Pantazis, M Hoffmann, S Hickling, J Hurst, J Meyerowitz, CB Willberg, N Robinson, H Brown, M Fisher, S Kinloch, A Babiker, J Weber, N Nwokolo, J Fox, S Fidler, R Phillips, J Frater, SPARTAC and Cherub Investigators

**Affiliations:** 1 Nuffield Department of Medicine, University of Oxford, Oxford, United Kingdom; 2 Department of Hygiene, Epidemiology & Medical Statistics, Athens University Medical School, Athens, Greece; 3 Medical Research Council Clinical Trials Unit, University CollegeLondon, London, United Kingdom; 4 Division of Infectious Diseases and Hospital Epidemiology, Kantonsspital, St Gallen, Switzerland; 5 Institute for Emerging Infections, The Oxford Martin School, Oxford, United Kingdom; 6 Oxford Biomedical Research Centre, National Institute of Health Research, Oxford, United Kingdom; 7 Brighton and Sussex University Hospitals, Brighton, United Kingdom; 8 Division of Infection and Immunity, University College London, London, United Kingdom; 9 Division of Medicine, Wright Fleming Institute, Imperial College, London, United Kingdom; 10 Chelsea and Westminster Hospital, London, United Kingdom; 11 Department of Genitourinary Medicine and Infectious Disease, Guys and St Thomas’ NHS Trust, London, United Kingdom

## Abstract

**Introduction:**

The rate at which HIV-1-infected individuals progress to AIDS is highly variable and impacted by T cell immunity. CD8 T cell inhibitory molecules are upregulated in HIV-1 infection and associate with T cell dysfunction. Here we aimed to determine whether CD8 T cell immune checkpoint markers PD-1, Lag-3 and Tim-3 are associated with immune activation and disease progression.

**Methods:**

We evaluated participants (n = 122) with primary HIV infection (PHI) randomized to receive short-course antiretroviral therapy (ART), or no therapy, in the SPARTAC trial. Expression of PD-1, Tim-3, Lag-3 and CD38 on CD8 T cells from the closest pre-therapy time-point to seroconversion was measured by flow cytometry and correlated with surrogate markers of HIV disease (HIV-1 plasma viral load (pVL) and CD4 T cell count) and the trial endpoint (time to CD4 count <350 cells/µl or initiation of long-term antiretroviral therapy). To explore the functional significance of these markers, co-expression of Eomes, T-bet and CD39 was assessed.

**Results:**

Expression of PD-1 on bulk and CD38 CD8 T cells correlated with pVL and CD4 count and predicted the trial endpoint. Lag-3 expression was associated with pVL but not CD4 count. For all exhaustion markers, expression on CD38 CD8 T cells increased the strength of the associations. In Cox models, progression to the trial endpoint was most marked for PD-1/CD38 co-expressing cells, with evidence for a stronger effect within 12 weeks from seroconversion. The effect of PD-1 and Lag-3 expression on CD8 T cells retained statistical significance in Cox proportional hazards models including ART and CD4 count, but not pVL as co-variants (HR 1.76; p = 0.047 and HR 1.46; p = 0.024 respectively). In a cohort of similar individuals with untreated PHI, we demonstrated strong associations of PD-1 and Lag-3 with the T-bet^dim^/Eomes^hi^ CD8 population and CD39 expression, suggesting the expression of these markers during PHI represents functional exhaustion.

**Conclusions:**

Expression of “exhaustion” or “immune checkpoint” markers in early HIV infection is associated with clinical progression and may be impacted by immune activation and timing of therapy. New markers to identify exhausted T cells and novel interventions to reverse exhaustion may inform the development of new immunotherapeutic approaches.

# Sub article: WEAA0106LB HIV Cure research in a novel population of South African hyper-acute HIV infections detected in the blood donation setting: the Monitoring and Acute Treatment of HIV Study (MATHS)

**Published:** 2016-07-22

**Authors:** K van den Berg, M Vermeulen, T Xulu, C McClure, C Ingram, G Beck, M Stone, MP Busch, B Custer, EL Murphy, NHLBI Recipient Epidemiology and Donor Evaluation Study III (Reds III)

**Affiliations:** 1 Medical Division, South African National Blood Service, Johannesburg, South Africa; 2 South African National Blood Service, Johannesburg, South Africa; 3 Right To Care Health Services, Johannesburg, South Africa; 4 RTI International, Rockville, MD, United States; 5 Blood Systems Research Institute, San Francisco, CA, United States; 6 University of California San Francisco, San Francisco, CA, United States

## Abstract

**Introduction:**

All blood donations in South Africa are tested in parallel for HIV antibody and for RNA using highly sensitive individual-donation nucleic acid testing (ID-NAT). About 60 South African donors per year are detected to have RNA but not antibody (Fiebig stages I and II). We reasoned that with rapid initiation of antiretroviral therapy (ART), this population could be important for studying the elimination of HIV reservoir and HIV Cure.

**Methods:**

We plan to enrol 50–75 Fiebig stage I and II HIV-infected persons detected at the time of blood donation. HIV antibody (Abbott Prism HIV O Plus) and HIV RNA ID-NAT (Griffols, Emeryville, CA) were measured on samples taken at donation and enrolment. In collaboration with Right to Care Health Services, ART with Raltegravir/FTC/TDF is initiated at enrolment and switched to EFV/FTC/TDF at the 6th month. HIV reservoir will be measured prospectively on leukocytes obtained from peripheral blood and plasma/leukapheresis. Finally, 25 Elite controllers defined as antibody positive but HIV RNA negative on ID-NAT, are followed for HIV virology and immunology without treatment.

**Results:**

Since October 2015, we have enrolled 14 donors with hyperacute HIV infection, median age 26 years, 10 female, 12 Black, 1 Asian and 1 White, mean HIV RNA 506,000 copies and mean CD4 547 cells/mm^3^. Enrolment occurred a median of 13 days after donation, and ART was initiated a median of 2 days after enrolment. Participants were Fiebig stages I (n = 9) and II (n = 5) at donation and Fiebig stages I (n = 1), II (n = 6) and III (n = 7) at enrolment. In nine evaluable participants, viral suppression (<20 copies/mL on a sensitive viral load assay) occurred after a median of 35 days on ART. To date, seven Elite controllers have been enrolled.

**Conclusions:**

This study provides proof of principle that a partnership between the national blood service and a treatment at NGO can be used to detect and rapidly treat persons with hyperacute HIV infection in South Africa. Initial results suggest that half of enrolees were still in Fiebig stage I/II at enrolment and that rapid viral suppression can be achieved once they are started on ART.

# Sub article: WEAB0101 Acceptability of early HIV treatment among South African women

**Published:** 2016-07-22

**Authors:** N Garrett, E Norman, V Asari, N Naicker, N Majola, K Leask, Q Abdool Karim, S Abdool Karim

**Affiliations:** 1 Centre for the AIDS Programme of Research in South Africa (CAPRISA), Durban, South Africa; 2 Columbia University, New York City, NY, United States

## Abstract

**Introduction:**

WHO guidelines recommend immediate initiation of antiretroviral therapy (ART) for all individuals at HIV diagnosis regardless of CD4 count. There is a concern among some health care providers that there will be low uptake and/or poor adherence for ART in patients who are well and have high CD4 counts, but there is little data on uptake of earlier ART in resource-poor settings. This study assessed the acceptability of earlier treatment among HIV-positive South African women in a 10-year prospective cohort study (CAPRISA 002).

**Methods:**

CD4 count and HIV viral load were measured 3-monthly from acute infection until five years post-ART initiation for CAPRISA 002 participants. Acceptability of earlier ART initiation was assessed by

(i) describing temporal trends of CD4 count at initiation in relation to WHO guidance, (ii) virological suppression rates post-ART initiation at different CD4 count thresholds, and (iii) administration of a standardized questionnaire.

**Results:**

A total of 170/232 (73.3%) CAPRISA002 participants had initiated ART between January 2006 and December 2015. Mean CD4 count at initiation was 216 cells/µl (standard deviation (SD) 73.0; range 135–372) before 2010, and substantially increased to 531 cells/µl (SD 183; range 272–1095) by 2015 (p< 0.001). Median viral load simultaneously decreased from 5.3 (interquartile range (IQR) 4.6–5.8) to 4.1 (IQR 3.4–4.6) log copies/ml (p=0.004). Virological suppression rates at 3, 6, 12 and 18 months were consistently above 85% with no statistically significant differences for participants starting ART at higher versus lower CD4 count thresholds (Table 1). An early ART questionnaire revealed that 40/51 (78.4%) participants were willing to start ART at CD4 500 cells/µl or above, while 11/51 (21.6%) were unwilling. Within 6 months of questionnaire administration, 28/40 (70.0%) and 6/11 (54.5%) participants had initiated treatment (p=0.472).

**Conclusions:**

Temporal increases in CD4 counts, high virological suppression rates and positive patient perceptions confirm high acceptability of ART irrespective of CD4 treatment threshold for the majority of patients in this population.

**Abstract WEAB0101–Table 1:** Virological suppression after ART initiation at different CD4 count thresholds

CD4 Count at Initiation (cells/µl)	3 months	6 months	12 months	18 months
<350 (n=77)	87.9% (51/58)	85.9% (67/78)	87.8% (65/74)	92.8% (65/70)
≥350 (n=80)	95.3% (61/64)	92.7% (51/55)	92.1% (35/38)	100% (26/26)
P-value	0.190	0.273	0.544	0.319
<500 (n=132)	92.2% (94/102)	88.5% (100/113)	88.0% (88/100)	94.4% (85/90)
≥500 (n=35)	90.0% (18/20)	90.0% (18/20)	100% (12/12)	100% (6/6)
P-value	1.000	1.000	0.357	1.000

# Sub article: WEAB0102 Timing of pregnancy among HIV-positive women, postpartum retention and risk of virologic failure

**Published:** 2016-07-22

**Authors:** D Onoya, T Sineke, A Brennan, MP Fox

**Affiliations:** 1 Health Economics and Epidemiology Research Office, University of the Witwatersrand, Johannesburg, South Africa; 2 School of Public Health, Boston University, Boston, MA, United States

## Abstract

**Introduction:**

The wide implementation of the prevention of mother-to-child transmission (PMTCT) Option B+ approach in South Africa warrants a closer examination of postpartum antiretroviral therapy (ART) outcomes. In this study we examine the association between timing of the first pregnancy with the risk and predictors of postpartum ART failure and disengagement from HIV care in South Africa.

**Methods:**

This is a retrospective cohort study of 5780 HIV-positive women aged 15 to 49, initiated on ART between 2004 and September 2014 in Johannesburg, South Africa. The incidence and predictors of ART failure (two consecutive viral load >1000 copies/ml) and loss to follow up (LTFU, >3 months late for a scheduled visit) during 24 months post-delivery/equivalent time were assessed using Cox proportional hazards modelling.

**Results:**

Compared to non-pregnant women (rate 5.6 per 100 PY), women were more likely to be LTFU after a prevalent (rate 13.7 per 100 PY; HR 8.2, 95% CI: 6.3–10.6) or an incident pregnancy (rate 10.1 per 100 PY; HR 5.0, 95% CI: 4.0–6.2). The risks of ART failure following an incident pregnancy (rate 5.9 per 100 PY; HR 2.2, 95% CI: 1.6–2.9) and in the risk in the non-pregnant group (rate 7.6 per 100 PY; HR 1.9, 95% CI: 1.4–2.6) were higher than the risk after a prevalent pregnancy (rate 4.9 per 100 PY). Predictors of postpartum ART failure were being anaemic at delivery (HR 1.25, 95% CI: 1.01–1.54), having a low CD4 (<350 cells) (HR 1.9, 95% CI: 1.6 – 2.4) and meeting the definition for LTFU (HR 1.4, 95% CI: 1.1–1.9). When stratified by CD4 count, among women with low CD4 (<350) at delivery, the hazard of failure in the incident pregnancy group remained higher than in the prevalent pregnancy group (HR 2.5, 95% CI: 1.8–3.5). There was no difference in the high CD4 strata.

**Conclusions:**

The risk of HIV treatment failure remains high among postpartum women, particularly those who conceive while on ART. The results highlight the importance of strengthening retention and monitoring efforts for postpartum women to sustain the benefits of the PMTCT programme.

# Sub article: WEAB0103 Adverse obstetrical outcomes among HIV-positive and HIV-negative mother-infant pairs in Nigeria and South Africa: findings from the INFANT study

**Published:** 2016-07-22

**Authors:** P Datong, H Jaspan, S Osawe, J Gatei, J-A Coombs, KA Muldoon, R Mallick, C Gray, K Rosenthal, DW Cameron, A Abimiku

**Affiliations:** 1 Plateau State Specialist Hospital, Jos, Nigeria; 2 Seattle Children's Research Institute, Seattle, United States; 3 Faculty of Health Sciences, Institute of Infectious Disease and Molecular Medicine, University of Cape Town, Cape Town, South Africa; 4 Plateau State Human Virology Research Centre, Institute of Human Virology, Jos, Nigeria; 5 Clinical Epidemiology Program, Ottawa Hospital Research Institute Ottawa, Canada; 6 Public Health and Preventative Medicine, School of Epidemiology, University of Ottawa, Ottawa, Canada; 7 Clinical Laboratory Sciences, Institute of Infectious Disease and Molecular Medicine, University of Cape Town, Cape Town, South Africa; 8 Department of Pathology and Molecular Medicine, McMaster University, Hamilton, Canada; 9 Department of Medicine, The Ottawa Hospital and University of Ottawa, Ottawa, Canada; 10 School of Medicine, Institute of Human Virology, University of Maryland, Baltimore, MD, United States

## Abstract

**Introduction:**

Sub-Saharan Africa has disproportionately high rates of infant morbidity and mortality. Healthy obstetrical outcomes are critical for establishing strong developmental trajectories, especially among infants exposed to HIV perinatally. This analysis was designed to identify the prevalence and correlates of adverse neonatal outcomes among HIV+ and HIV− mothers and their infants.

**Methods:**

The INFANT study is a longitudinal cohort of healthy HIV+ and HIV− mother-infant pairs recruited from B Clinic in Khayelitsha, South Africa and Plateau State Specialist Hospital in Jos, Nigeria (April 2013 to March 2015). Adverse obstetrical outcomes included low birth weight(<2500 g), small for gestational age (<10 percentile), pre-term birth (<37 weeks) and a composite outcome for any adverse outcome. Using odds ratios (OR) and 95% confidence intervals (CI), bivariable and multivariable logistic regressions determined association between HIV and adverse obstetrical outcomes.

**Results:**

A total of 680 mother-infant pairs were recruited into the study – 490 pairs with HIV+ mothers and 190 with HIV- mothers. The mother's median age was 29 years (IQR: 25–33), 147 (21.65%) had less than elementary school education, 503 (74.19%) were married, and 477 (70.25%) lived without running water. A total of 170 (25.00%) births had an adverse obstetrical outcome -30 (4.41%) with low birth weight, 92 (13.53%) were small for gestational age, and 71 (10.44%) were pre-term. Adverse outcomes were higher among mothers with HIV (OR: 1.52, 95% CI: 1.08–2.13), those with less than elementary school (OR: 1.49, 95% CI: 1.12–1.97) and those without running water (OR: 1.48, 95% CI: 1.06–2.05). Married mothers (OR: 0.55, 95% CI:0.98–0.81) and mothers from the Khayelitsha site (OR: 0.35, 95% CI: 0.24–0.50) had lower odds of adverse outcomes. After adjusting for education, running water, marital status and study site, mothers living with HIV had significantly higher odds of adverse obstetrical outcomes (AOR: 1.45, 95% CI: 1.03–2.04).

**Conclusions:**

Although this study was designed to include healthy births, 25% of mother-infant pairs had an adverse obstetrical outcome, and 45% higher odds were documented among mothers with HIV. Comprehensive antenatal care for all women, including those living with HIV, is needed to optimize maternal and child health.

# Sub article: WEAB0104 High proportion of deaths attributable to HIV among post-partum women in Botswana despite widespread uptake of ART

**Published:** 2016-07-22

**Authors:** R Zash, S Souda, J Leidner, K Binda, C Hick, K Powis, J Makhema, M Mmalane, M Essex, S Lockman, R Shapiro

**Affiliations:** 1 Beth Israel Deaconess Medical Center, Division of Infectious Diseases, Boston, MA, United States; 2 Harvard School of Public Health, Harvard University, Boston, MA, United States; 3 Botswana Harvard AIDS Institute Partnership, Gaborone, Botswana; 4 Faculty of Health Sciences, University of Botswana, Gaborone, Botswana; 5 Goodtables Data Consulting, Norman, OK, United States; 6 Massachusetts General Hospital, Boston, MA, United States; 7 Brigham and Women's Hospital, Boston, MA, United States

## Abstract

**Introduction:**

Mortality in the post-partum period may be impacted by antiretroviral treatment (ART) received in pregnancy, and whether ART is continued in the post-partum period.

**Methods:**

HIV-infected and HIV-uninfected mothers were enrolled within 48 hours of delivery at five public hospital maternity wards throughout Botswana. Follow up visits were conducted by mobile phone at 1 and 3 months, then every 3 months until 24 months post-partum. Maternal deaths were reported by one of the approved contacts given by the mother at enrolment. Risk factors for maternal survival were assessed using Cox proportional hazard models.

**Results:**

Between February 2012 and March 2013, 3000 mothers (1499 HIV-infected and 1501 HIV-uninfected) were enrolled. There were 26 total maternal deaths in 24 months post-partum (411 per 100,000 person-years), 22 among HIV-infected women (769 per 100,000 person-years) and 4 among HIV-uninfected women (134 per 100,000 person-years). Maternal age, availability of indoor toilet, formal housing, Rh factor, preterm delivery and higher parity were associated with mortality in univariate, but not adjusted analyses. Maternal HIV-infection (aHR 5.0, 95% CI: 1.6, 15.2) and infant birth injury (aHR 3.8, 95% CI: 1.3, 11.4) were independent risk factors for maternal death in the post-partum period. Among HIV-infected women, when compared with the 924 women who received continuous 3-drug ART in pregnancy and post-partum, there was no significant increase in mortality among 281 women who discontinued ART after pregnancy (aHR 2.1, 95% CI: 0.6, 7.5); among 241 women who initiated or re-started ART in the post-partum period (aHR 1.1, 95% CI: 0.2, 5.1); or among 70 women who received no ART at any time (aHR 2.8, 95% CI: 0.3, 28.2). CD4 cell count in pregnancy was not associated with mortality (p=0.20) and longer ART duration prior to delivery (>2 years) did not decrease mortality (aHR 0.6, 95% CI: 0.1, 3.4).

**Conclusions:**

Despite high uptake of 3-drug ART in pregnancy and post-partum, HIV-infected women were 5 times more likely than HIV-uninfected women to die within 24 months after delivery, independent of CD4 cell count. Further research is needed to understand the increased risk of mortality among HIV-infected post-partum women.

# Sub article: WEAB0105 Birth weight and preterm delivery outcomes of perinatally vs. non-perinatally HIV-infected pregnant women in the U.S.: results from the PHACS SMARTT study and IMPAACT P1025 protocol

**Published:** 2016-07-22

**Authors:** J Jao, D Kacanek, P Williams, M Geffner, EG Livingston, RS Sperling, K Patel, A Bardeguez, S Burchett, N Chakhtoura, GB Scott, R Van Dyke, EJ Abrams, Pediatric HIV/AIDS Cohort Study (PHACS) & International Maternal Pediatric Adolescent AIDS Clinical Trials (IMPAACT) Network

**Affiliations:** 1 Department of Medicine, Obstetrics, Gynecology, and Reproductive Science, Icahn School of Medicine at Mount Sinai Mount Sinai hospital, New York, NY, United States; 2 Harvard T. H. Chan School of Public Health, Center for Biostatistics in AIDS Research, Department of Biostatistics, Boston, MA, United States; 3 Keck School of Medicine of the University of Southern California, The Saban Research Institute of Children's Hospital Los Angeles, Los Angeles, CA, United States; 4 Department of Obstetrics and Gynecology, Duke University Medical Center, Durham, NC, United States; 5 Department of Obstetrics, Gynecology, and Reproductive Science, Icahn School of Medicine at Mount Sinai, Mount Sinai hospital, New York, NY, United States; 6 Department of Epidemiology, Center for Biostatistics in AIDS Research, Harvard T.H. Chan School of Public Health, Boston, MA, United States; 7 Department of Obstetrics, Gynecology, and Women's Health, Rutgers New Jersey Medical School, The State University of New Jersey, Newark, NJ, United States; 8 Division of Infectious Diseases, Boston Children's Hospital and Harvard Medical School, Boston, MA, United States; 9 Maternal and Pediatric Infectious Disease Branch, Eunice Kennedy Shriver National Institute of Child Health and Human Development, Bethesda, United States; 10 Department of Pediatrics, Division of Pediatric Infectious Disease and Immunology, University of Miami Miller School of Medicine, Miami, FL, United States; 11 Department of Pediatrics, Division of Infectious Diseases, Tulane University School of Medicine, New Orleans, LA, United States; 12 ICAP, Columbia University Mailman School of Public Health, New York, NY, United States

## Abstract

**Introduction:**

The success of antiretroviral therapy (ART) has resulted in many perinatally HIV-infected (PHIV) youth reaching reproductive age. Pregnancy outcomes of PHIV women compared to women acquiring HIV non-perinatally (nPHIV) are poorly defined.

**Methods:**

We compared birth weight (BW) and preterm delivery (PTD) outcomes of PHIV versus nPHIV pregnant women enrolled in the Pediatric HIV/AIDS Cohort Study (PHACS) Surveillance Monitoring for ART Toxicities Study (SMARTT) or IMPAACT P1025 protocol. Women were 13-30 years old. Infants were HIV-uninfected singleton liveborns. Maternal PHIV status was identified by self-report, medical record review or HIV infection documented within 5 years of birth. BW *z*-scores (BWZ) and small-for-gestational-age (SGA) were calculated using U.S. standards. Mixed effects models were applied to assess the association of maternal PHIV status with infant BWZ log binomial models using generalized estimating equations were fit for PTD (delivery at <37 weeks) and SGA outcomes.

**Results:**

From 1998–2013, 2270 HIV-infected pregnant women delivered 2692 newborns (270 born to PHIV and 2422 to nPHIV women). Compared to nPHIV women, PHIV women were younger (mean age 21 vs. 25 years, p<0.01) and less often Black (55% vs. 67%, p<0.01). PHIV women were more likely to have a CD4 count < 200 cells/mm^3^ during pregnancy (19% vs. 11%, p=0.01), delivery HIV RNA level ≥400 copies/ml (28% vs. 23%, p<0.01), receipt of ≥3-class ART during pregnancy (23% vs. 2%, p<0.01), and pre-pregnancy body mass index (BMI) <18.5 kg/m^2^ (6% vs. 3%, p<0.01). PHIV were less likely to report tobacco (14% vs. 20%, p=0.01) and substance use (1.7% vs. 3.3%, p<0.01) during pregnancy. After adjustment, BWZ was 0.13 lower in infants of PHIV vs. nPHIV women (adjusted mean: −0.46 vs. −0.33, p=0.03). Black race, tobacco and substance use in pregnancy, and maternal pre-pregnancy BMI < 18.5 kg/m^2^ were also significantly associated with lower infant BWZ. No associations between maternal PHIV status and PTD or SGA were observed.

**Conclusions:**

Infants of PHIV versus nPHIV women may be at greater risk for lower BW, although the absolute difference was small. Future studies are warranted to understand mechanisms by which the intrauterine environment of PHIV women may affect fetal growth.

# Sub article: WEAB0201 Daily is better than thrice-weekly anti-tuberculosis therapy in HIV patients with culture-confirmed pulmonary TB: a randomized controlled clinical trial from south India

**Published:** 2016-07-22

**Authors:** G Narendran, S Ramesh Kumar, C Padmapriyadarsini, NS Gomathi, PA Menon, L Sekar, S Devarajulu Reddy, S Chandra, AK Hemanth Kumar, S Kumar, S Sekar, N Ravichandran, K Raja, J Lavanya, R Sridhar, M Lakshmi, A Mahilmaran, S Swaminathan

**Affiliations:** 1 Clinical Research, National Institute for Research in Tuberculosis, Chennai, India; 2 TB Sanatorium, Government Hospital of Thoracic Medicine, Chennai, India; 3 ART Centre, Madras Medical College, Chennai, India; 4 District TB Office, Government of Tamil Nadu, Chennai, India; 5 Pulmonology, Government Stanley Hospital, Chennai, India; 6 State TB Officer, Government of Tamil Nadu, Chennai, India; 7 Pulmonology, Institute for Thoracic Medicine, Chennai, India; 8 Department of Health Research (DHR), Indian Council of Medical Research, New Delhi, India

## Abstract

**Introduction:**

Benefit of daily over thrice-weekly anti-tuberculosis therapy (ATT) in pulmonary TB (PTB) patients with HIV on anti-retroviral therapy (ART) is unclear.

**Methods:**

Efficacy comparison (first head to head) of three ATT regimens of 6 months administered daily (**A**: 2EHRZ_7_/4HR_7_), part daily (**B**: 2EHRZ_7_/4HR_3_) or thrice weekly throughout (**C**: 2EHRZ_3_/HR_3_) in HIV-PTB. An open-label randomized clinical trial at the National Institute for Research in Tuberculosis, India, enrolled HIV-infected treatment-naive confirmed PTB patients (sputum smear or Xpert-MTB Positive). Clinical evaluation, two sputa smear for Acid Fast Bacillus and culture including drug susceptibility testing (Lowenstein Jensen medium), was done at baseline and monthly for 18 months. CD4 cell count, HIV viral load, liver and renal function tests, and chest X-ray were performed at 0, 2, 6, 12 and 18 months. Block randomization, stratified by baseline sputum smear grading (0 and 1+) or (2+ and 3+) and CD4 cell count (<150 or >150), was performed and patients allocated to fully supervised ATT regimens **A**, **B** and **C**. ART initiation was within 8 weeks of starting ATT. Pre-treatment rifampicin-sensitive cases were analyzed. Primary outcomes were failures and acquired rifampicin resistance (ARR). Secondary outcomes were death, default and toxicity. Intent to treat and efficacy analyses were performed. Outcomes compared using Chi-square test. (NCT00933790).

**Results:**

Till date, 324 patients were allocated to regimens A (110), B (109) and C (105) respectively. Baseline characteristics were comparable. Favourable responses (intention to treat (ITT)) in **A** and **C** were 90% (83/92) vs.77% (65/85) {p=0.013, crossing O'Brien–Fleming boundaries at second interim analysis}. B had 79% (70/89) efficacy. Failures were three in B and eight (four with ARR) in C. Adverse drug reactions were 24%, 19% and 10% in A, B and C regimens, respectively.

**Conclusions:**

Daily ATT resulted in higher cure, lower failure, no emergence of ARR but higher toxicity (mostly manageable) compared to thrice-weekly ATT.

**Abstract WEAB0201–Table 1:** Baseline characteristics of HIV patients with newly diagnosed PTB randomized to three ATT regimens

	Daily regimen A (n=110)	Part daily regimen B (n=109)	Intermittent regimen C (n=105)
Mean age in years ±SD	38±8	39±9	39±9
Mean weight in kilograms ±SD	42.6±8.1	42.0±7.5	44.4±7.5
Mean HB gms %±SD	9.7±2.2	9.6±2.0	10.0±2
Mean HCT %±SD	28.7±6.6	27.8±5.3	29.8±7.1
Mean HIV viral load(log_10_){copies/ml}±SD	4.8±1.2	4.9±1.0	4.9±1.2
Median CD4 cell count (IQR)cells/mm^3^	130 (65–220)	145 (79–262)	135 (65–252)
Median ATT-ART interval (IQR), in days	17 (3–36)	17 (5–45)	15 (2–34)
Mycobacterium TB sensitive to all drugs %	77	71	68

# Sub article: WEAB0202 Intensified tuberculosis case-finding among people living with HIV: diagnostic yield of Xpert MTB/RIF, urine lipoarabinomannan and liquid culture

**Published:** 2016-07-22

**Authors:** E Atuhumuza, C Yoon, J Katende, L Asege, S Mwebe, A Andama, D Armstrong, D Dowdy, M Kamya, FC Semitala, A Cattamanchi

**Affiliations:** 1 Infectious Diseases Research Collaboration, Kampala, Uganda; 2 Department of Medicine, University of California, San Francisco, CA, United States; 3 Makerere University Joint AIDS Programme, Kampala, Uganda; 4 Department of Medicine, College of Health Sciences, Makerere University, Kampala, Uganda; 5 Department of Medicine, Johns Hopkins University, Baltimore, MD, United States; 6 Department of Epidemiology, Johns Hopkins University, Baltimore, MD, United States

## Abstract

**Introduction:**

To reduce the burden of tuberculosis (TB) among people living with HIV (PLHIV), the WHO recommends symptom-based screening at every clinic visit, followed by Xpert MTB/RIF (Xpert) testing for all individuals who screen positive (intensified case finding [ICF]). However, the utility of this ICF strategy is unknown. It is also not clear how other TB diagnostics can increase sensitivity and/or reduce the time to diagnosis.

**Methods:**

We administered the WHO TB symptom screen to consecutive HIV-infected adults with CD4+ count ≤350 cells/ml, initiating antiretroviral therapy (ART) in Uganda from July 2013 to December 2015. We collected two spot sputum specimens from all patients and urine if CD4 was ≤200 cells/μl. We compared the proportion of culture-confirmed TB cases detected by individual tests (sputum Xpert, urine Determine TB lipoarabinomannan (LAM) [Grade II cut-point], a single sputum mycobacterial growth indicator tube [MGIT] culture) and test combinations (Xpert+LAM, Xpert+MGIT, LAM+MGIT, Xpert+LAM+MGIT). We also calculated the time to TB detection as the time from enrolment to first positive result.

**Results:**

Symptom screening was positive in 1012 of 1128 (90%) patients (median CD4 156 cells/μl, IQR 69-265), including 152 of 159 patients with TB. TB prevalence among symptomatic PLHIV was 15%. Of the 152 symptomatic and culture-confirmed TB cases, 49% (95% CI: 41–57) were identified with Xpert, 33% (95% CI: 20–48) with LAM and 77% (95% CI: 69–83) with a single MGIT culture. Compared to Xpert alone, the diagnostic yield increased to 68% (difference 19%, 95% CI: 2–35) with Xpert+LAM, 85% (difference 36%, 95% CI: 26–46) with Xpert+MGIT, 80% (difference 30%, 95% CI: 19–42) with LAM+MGIT and 86% (difference 37%, 95% CI: 26–47) with Xpert+LAM+MGIT. The false-positive rate was <3% for all strategies. Over 75% of patients were diagnosed on the same day for ICF strategies that used Xpert, LAM, or Xpert+LAM.

**Conclusions:**

TB prevalence was high among symptomatic PLHIV initiating ART. The currently-recommended ICF strategy (Xpert alone) missed nearly half of all TB cases. The inclusion of urine LAM increased diagnostic yield and same-day diagnosis, but without culture, any ICF strategy is likely to miss at least one-third of all patients with active TB in this setting.

# Sub article: WEAB0203 Cytomegalovirus viraemia and 12-week mortality among hospitalized adults with HIV-associated tuberculosis in Khayelitsha Hospital, South Africa: a prospective cohort study

**Published:** 2016-07-22

**Authors:** A Ward, D Barr, C Schutz, R Burton, A Boulle, G Maartens, RJ Wilkinson, G Meintjes

**Affiliations:** 1 University of Cape Town, Cape Town, South Africa; 2 The Wellcome Trust Liverpool Glasgow Centre for Global Health Research, University of Liverpool, Liverpool, United Kingdom; 3 Khayelitsha Hospital, Cape Town, South Africa

## Abstract

**Introduction:**

Mortality in hospitalized patients with HIV-associated tuberculosis remains high. Cytomegalovirus (CMV) organ disease is one of the co-infections found in autopsies of such patients. We investigated the association of CMV viraemia with mortality in this setting.

**Methods:**

HIV-infected inpatients in Khayelitsha Hospital with CD4 <350 cells/ml and new diagnosis of tuberculosis were enrolled from January 2014 to June 2015. Plasma CMV qPCR was performed and categorized as detectable (CMV+) or undetectable (CMV−). Endpoint was 12-week mortality.

**Results:**

We included 256 patients with median age of 36 years (IQR: 31–44 years), 49% male, 35% on ART, median CD4=64 cells/ml (IQR: 24–117) and 79(30.9%) CMV+. By 12 weeks, 26/77(38.0%) of CMV+ and 31/174(17.8%) of CMV− patients died (p=0.008); 5 were lost to follow-up. In CMV+ patients with <1000 copies/ml mortality was 12/36 (33.3%) compared to 14/41 (34.1%) in those with higher viral load (p=1.0).

Mortality was higher in older patients (≥36 years): 32.8% vs. 14.1% (p<0.001). Older patients were more likely to be CMV+ (38.0% vs. 23.6%, p=0.015) and a larger proportion of older patients had CD4 count <50 cells/ml (48.5% vs. 37.9%, p=0.106). In Kaplan-Meier analysis, CMV+ was associated with mortality in older, not younger patients.

In multivariate Cox proportional-hazards regression, age (aHR=1.70, 95% CI: 1.34–2.15 per 10 years increase) was associated with mortality; CMV status was not.

**Conclusions:**

CMV viraemia was associated with higher mortality, but not after adjusting for potential confounders. Older patients had higher mortality and were more likely to have CMV viraemia. CMV viraemia is likely a marker of more severe immunodeficiency rather than a direct contributor to mortality.

**Abstract WEAB0203–Figure 1:** Survival by CMV status: whole cohort and stratified by age. (a) Survival by CMV status, all patients; (b) patients aged less than 36 years; (c) patients aged 36 years and older.

**Abstract WEAB0203–Table 1:** Cox proportional hazards regression analysis of factors associated with 12-week mortality

	Univariate				Multivariate: n=232 with complete observations			
		
	HR	lower 95% CI	upper 95% CI	p	aHR	lower 95% CI	upper 95% CI	p
Age, per 10 years	1.75	1.41	2.19	**<0.001**	1.70	1.34	2.15	**<0.001**
Male sex	0.84	0.50	1.41	0.505	0.69	0.41	1.19	0.185
CD4, per 50 cells×10^6^ l	0.78	0.64	0.96	**0.017**	0.80	0.64	1.00	0.052
HIV viral load, Log10 copies/ml	0.90	0.80	1.03	0.117	0.94	0.82	1.07	0.316
Mycobacteraemia	1.48	0.88	2.49	0.138	1.00	0.56	1.76	0.990
Albumin, per 5 g/l	0.73	0.58	0.92	**0.006**	0.84	0.66	1.08	0.177
CMV viraemia	2.09	1.24	3.53	0.004	1.67	0.95	2.93	0.077
Likelihood ratio test=37.2 on 7 d.f., p<0.0001								

Bold values are statistically significant at <0.05.

# Sub article: WEAB0204 Yield of community health worker-driven intensified case finding for tuberculosis among HIV-positive patients in rural Malawi

**Published:** 2016-07-22

**Authors:** R Flick, K Simon, A Munthali, A Dimba, M Kim, P Kazembe, M Hosseinipour, S Ahmed

**Affiliations:** 1 University of North Carolina Project-Malawi, Lilongwe, Malawi; 2 Baylor College of Medicine Children's Foundation Malawi, Lilongwe, Malawi; 3 University of Colorado School of Medicine, Denver, CO, United States; 4 Baylor International Pediatric AIDS Initiative at Texas Children's Hospital, Baylor College of Medicine, Houston, TX, United States; 5 Malawi Ministry of Health National Tuberculosis Control Programme, Lilongwe, Malawi; 6 University of North Carolina at Chapel Hill, Chapel Hill, NC, United States

## Abstract

**Introduction:**

Tuberculosis (TB) is the most common cause of death in HIV-positive patients. Early detection and anti-TB treatment initiation improve outcomes and minimizes ongoing transmission. Intensified case finding (TB-ICF) among HIV-positive patients is recommended by the WHO; however, evidence from routine implementation at high-volume antiretroviral therapy (ART) clinics in resource-constrained settings is scarce. Here, we describe the yield of TB-ICF conducted by community health workers (CHWs) among HIV-positive patients accessing ART in rural Malawi.

**Methods:**

Thirteen CHWs employed by the Baylor Tingathe outreach programme were trained to conduct TB-ICF using a standardized symptom screening tool at a large rural district hospital. Patients were screened while awaiting routine services at ART clinic. Patients screened positive were triaged for assessment by a clinician and sputum analysis by smear microscopy and GeneXpert. Patients were followed up until final diagnosis and traced if necessary. Sixteen months of pre- and 6 months of post-intervention data were abstracted from registers and tools used by CHWs. Single-group interrupted time series analysis was used to assess impact of the intervention.

**Results:**

The mean number of monthly TB diagnoses made at ART clinic increased by a factor of 20 post-intervention (0.5 vs. 10.0 monthly diagnoses, p<0.0001). In the first month of the intervention an immediate increase of 6.7 monthly diagnoses occurred (p<0.0001, Figure 1). There was a statistically significant increase in the monthly trend of TB diagnoses relative to the pre-intervention trend of 0.78 per month (p=0.026). The yield of screening in the post-intervention period was 10.0% (46/459). Diagnoses were only made in children post-intervention (9/46, 19.6%).

**Conclusions:**

Implementation of TB-ICF with CHWs was associated with significant increases in the number and trend of monthly TB diagnoses. Screening resulted in favourable yields and helped link children to care. Future work is needed to ascertain the durability of this effect and the impact on treatment outcomes.

**Figure 1:** Actual and model-fitted monthly TB diagnoses at ART clinic before and after CHW TB-ICF.

# Sub article: WEAB0205LB High-dose rifampicin tuberculosis treatment regimen to reduce 12-month mortality of TB/HIV co-infected patients: the RAFA trial results

**Published:** 2016-07-22

**Authors:** CS Merle, S Floyd, A Ndiaye, T Galperine, A Furco, BC De Jong, H McIlleron, J Glynn, M Sarr, O Bah-sow, D Affolabi, on behalf of the Rafa Team

**Affiliations:** 1 TDR, WHO, Geneva, Switzerland; 2 London School of Hygiene and Tropical Medicine, IDEU, London, United Kingdom; 3 National Tuberculosis Programme, Dakar, Senegal; 4 Tenon Hospital, Paris, France; 5 University College London, London, United Kingdom; 6 Institute of Tropical Medicine, Antwerp, Belgium; 7 Clinical Pharmacology Laboratory, Cape Town University, Cape Town, South Africa; 8 Service de Pneumophtisiologie, Hospital Ignace Deen, Conakry, Guinea; 9 National TB Programme, Cotonou, Benin

## Abstract

**Introduction:**

Approximately 30% of TB/HIV patients die within 12 months of starting TB treatment. Current treatment strategies to reduce TB/HIV mortality rely largely on the optimal management of HIV disease. But, as supported by autopsy studies, the problem might also be seen from the TB perspective: more intensive TB treatment might also reduce mortality.

**Methods:**

We conducted an open label, three parallel arms, randomized controlled trial, among TB/HIV co-infected patients who were antiretroviral (ARV)-naive and with a CD4 cell-count ≥50 cells/mm^3^ at enrolment, in Benin, Guinea and Senegal. The trial arms were: Arm A – ARV initiation at 2 weeks combined with standard TB treatment; Arm B (control arm) – ARV initiation at 8 weeks combined with standard TB treatment; Arm C – ARV initiation at 8 weeks with high-dose rifampicin (15 mg/kg) during the first 2 months of TB treatment. The primary outcome was 12-month mortality.

**Results:**

In total, 778 TB/HIV patients were randomized (n = 262, 258 and 258 for arms A, B and C respectively). All TB cases were bacteriologically confirmed. CD4 cell-counts ranged from 50 to 949 (median 183), balanced across arms. By January 2016, all patients completed 12 months of follow-up post-randomization. The overall 12-month mortality rates were: 11.8, 15.5 and 10.9 per 100 person-years in arms A, B and C respectively. Using Cox regression, there was no evidence that overall mortality rates differed by treatment arm (p = 0.40). Restricting the analysis to patients with a baseline CD4 cell-count <100 cells/mm^3^, mortality was substantially reduced (p = 0.006) in Arm C, with high-dose rifampicin, compared with Arm B, but not in Arm A (p = 0.24) (Figure 1). There was no evidence of an increased risk of hepatotoxicity in Arm C.

**Conclusions:**

More aggressive TB treatment using high dose of rifampicin, in addition to ARV treatment, could reduce TB/HIV mortality among severely immunosuppressed co-infected TB/HIV patients.

**Figure 1:** Time to mortality, from randomization date in patients with less than 100 CD4 count and more than 100 CD4 count – Kaplan-Meier analyses.

# Sub article: WEAB0301 Sofosbuvir/velpatasvir fixed dose combination for 12 weeks in patients co-infected with HCV and HIV-1: the phase 3 ASTRAL-5 study

**Published:** 2016-07-22

**Authors:** N Bräu, D Wyles, S Kottilil, E Darr, K Workowski, A Luetkemeyer, O Adeyemi, P Ruane, B Doehle, KC Huang, A Osinusi, J McNally, M Natha, M Guion, T McLean, DM Brainard, JG McHutchison, S Naggie, M Sulkowski

**Affiliations:** 1 Mount Sinai School of Medicine, Mount Sinai hospital, New York, NY, United States; 2 University of California, San Diego, CA, United States; 3 University of Maryland, Baltimore, MD, United States; 4 David Geffen School of Medicine, Torrance, United States; 5 Emory University, Atlanta, GA, United States; 6 University of California, San Francisco, CA, United States; 7 Rush Medical College, Chicago, IL, United States; 8 Ruane Medical and Liver Health Institute, Los Angeles, CA, United States; 9 Gilead Sciences, Inc, Foster City, CA, United States; 10 Duke Clinical Research Institute, Durham, United Kingdom; 11 Johns Hopkins University School of Medicine, Baltimore, MD, United States

## Abstract

**Introduction:**

The once-daily fixed-dose combination (FDC) tablet of sofosbuvir/velpatasvir(SOF/VEL) administered for 12 weeks has demonstrated high efficacy in genotypes 1–6 HCV-infected patients. A prospective clinical trial was performed to evaluate the safety and efficacy of SOF/VEL in patients co-infected with HCV and HIV-1.

**Methods:**

This single-arm, open-label study enrolled treatment- naïve and -experienced HCV/HIV co-infected patients of all HCV genotypes with or without cirrhosis. Patients on stable antiretroviral (ARV) regimens with fully suppressed HIV RNA received SOF/VEL (400 mg/100 mg daily) for 12 weeks. ARV regimens included emtricitabine/tenofovir disoproxil fumarate or abacavir/lamivudine with raltegravir, cobicistat/elvitegravir, rilpivirine, ritonavir-boosted atazanavir, darunavir or lopinavir. Safety evaluations included adverse event (AE) and standard laboratory parameter monitoring including renal function monitoring, CD4 count and HIV-1 RNA levels. The primary endpoint was sustained virologic response 12 weeks after treatment (SVR12).

**Results:**

A total of 106 patients were enrolled and treated with SOF/VEL for 12 weeks. 86% were male, 45% were black, 77% had IL28B non CC genotypes, 29% had prior treatment failure (primarily PegIFN/RBV) and 16% had compensated cirrhosis. The genotype distribution was 62% GT1a, 11% GT1b, 10% GT2, 11% GT3 and 5% GT4. Median baseline CD4 count was 548 cells/µl (range: 183–1513 cells/µl) with a median estimated glomerular filtration rate of 97 ml/min (range 57–198 ml/min). Boosted protease inhibitor (PI) regimens were the most commonly used regimen (Table 1). In this interim analysis with 95% of patients beyond treatment week 4 time point, the most common AEs were fatigue (19%), headache (14%) and nausea (7%). One patient experienced a serious AE (toe infection), considered unrelated to study drugs. No patient experienced confirmed HIV virologic rebound (HIV-1 RNA≥400 copies/ml). No significant changes in lab abnormalities including renal function were observed. Efficacy and safety outcomes including complete SVR12, HIV parameters and the impact of HCV resistance variants on outcomes will be presented.

**Conclusions:**

The single tablet regimen of SOF/VEL administered for 12 weeks was well tolerated in HCV/HIV co-infected patients with GT 1–4, regardless of past treatment experience or presence of cirrhosis.

**Abstract WEAB0301–Table 1:** ARV Regimen at Enrollment

ARV regimen at enrollment	PI+NRTI	Integrase+NRTI	NNRTI+NRTI	Combination (at least 2 of the following classes: PI, NNRTI or integrase)
Number n (%)	50 (47%)	36 (34%)	13 (12%)	7 (7%)

PI: protease inhibitor, NRTI: nucleoside reverse transcriptase inhibitor, NNRTI: non-nucleoside reverse transcriptase inhibitor.

# Sub article: WEAB0302 Drug–drug interactions studies between HCV antivirals sofosbuvir and velpatasvir and HIV antiretrovirals

**Published:** 2016-07-22

**Authors:** E Mogalian, A Luetkemeyer, S Naik, M Natha, L Stamm, A Osinusi, G Shen, K Sajwani, J Mcnally, A Mathias

**Affiliations:** 1 Clinical Pharmacology, Gilead Sciences, Foster City, CA, United States; 2 San Francisco General Hospital, University of California, San Francisco, CA, United States; 3 Medical Affairs, Gilead Sciences, Foster City, CA, United States; 4 Clinical Research, Gilead Sciences, Foster City, CA, United States

## Abstract

**Introduction:**

A once-daily fixed-dose combination tablet composed of sofosbuvir (SOF; nucleotide analog NS5B inhibitor) and velpatasvir (VEL; pangenotypic NS5A inhibitor) is under regulatory review for the treatment of chronic HCV infection. Phase 1 studies were conducted in healthy volunteers to evaluate potential drug-drug interactions (DDIs) between SOF/VEL and HIV antiretroviral (ARV) regimens to support coadministration in HIV/HCV co-infected patients.

**Methods:**

These were multiple-dose, randomized, cross-over DDI studies. Subjects received SOF/VEL and ARVs EFV/FTC/TDF, RPV/FTC/TDF, DTG, RAL+FTC/TDF, EVG/COBI/FTC/TDF, DRV/r+FTC/TDF, ATV/r+FTC/TDF, LPV/r+FTC/TDF, or EVG/COBI/FTC/TAF alone and in combination. Steady-state plasma concentrations of SOF, its predominant circulating nucleoside metabolite GS-331007, VEL, and ARVs were analyzed on the last day of dosing for each treatment. Pharmacokinetic (PK) parameters were calculated and geometric least-squares means ratios and 90% confidence intervals (combination vs. alone) for SOF, GS-331007, VEL, and ARV AUC_tau_, C_max_ and C_tau_ were estimated and compared against lack of PK alteration boundaries of 70–143% for all analytes. Safety assessments were conducted throughout the study.

**Results:**

Of 237 enrolled subjects, 230 completed the studies; 5 subjects withdrew consent, 1 discontinued due to Grade 1 urticaria and 1 discontinued due to pregnancy. The majority of adverse events (AEs) were Grade 1 and there were no serious AEs. Table 1 reports the effect of coadministration on HIV ARVs and SOF/VEL. No clinically significant changes in the PK of HIV ARVs, except TDF, were observed when administered with SOF/VEL. Increased TFV exposure (~40%) was observed with SOF/VEL when administered as TDF.

**Conclusions:**

Study treatments were generally well tolerated. Results from these studies demonstrate that SOF/VEL may be administered safely with RPV, RAL, DTG, EVG, COBI, DRV/r, ATV/r and LPV/r (but not EFV) with a backbone of FTC/TDF or FTC/TAF. The safety and efficacy of SOF/VEL and ARVs are being evaluated in clinical studies of HIV/HCV co-infected subjects.

**Abstract WEAB0302–Table 1:** Effect of Coadministration on HIV ARVs and SOF/VEL

ARV with SOF/VEL	Effect on SOF/VEL AUC	Effect on ARV AUC
EFV/FTC/TDF	SOF: ↔ GS-331007: ↔ VEL: ↓53%	EFV: ↔ FTC: ↔ TFV: ↑81%
FTC/RPV/TDF	SOF: ↔ GS-331007: ↔ VEL: ↔	FTC: ↔ RPV: ↔ TFV: ↑40%
DTG	SOF: ↔ GS-331007: ↔ VEL: ↔	DTG: ↔
RAL+FTC/TDF	SOF: ↔ GS-331007: ↔ VEL: ↔	RAL: ↔ FTC: ↔ TFV: ↑40%
DRV/r+FTC/TDF	SOF: ↓28% GS-331007: ↔ VEL: ↔	DRV: ↔ RTV: ↔ FTC: ↔ TFV: ↑40%
ATV/r+FTC/TDF	SOF: ↔ GS-331007: ↔ VEL: ↑142%	ATV: ↔ RTV: ↔ FTC: ↔ TFV: ↔
LPV/r+FTC/TDF	SOF: ↓29% GS-331007: ↔ VEL: ↔	LPV: ↔ RTV: ↔ FTC: ↔ TFV: ↔
EVG/COBI/FTC/TDF	SOF: ↔ GS-331007: ↔ VEL: ↔	EVG: ↔ COBI: ↔ FTC: ↔ TFV: ↔
EVG/COBI/FTC/TAF	SOF: ↑37% GS-331007: ↑48% VEL: ↑50%	EVG: ↔ COBI: ↔ FTC: ↔ TAF: ↔ TFV: ↔

# Sub article: WEAB0303 Higher mortality in HIV-HBV co-infected persons with elevated HBV replication in the Temprano Trial

**Published:** 2016-07-22

**Authors:** MG Kouamé, R Moh, A Boyd, A Badjé, D Gabillard, JB N'takpé, S Maylin, SP Eholié, X Anglaret, C Danel, ANRS12136 Temprano Trial Group

**Affiliations:** 1 National Agency for AIDS Research (ANRS), Programme PAC-CI, Abidjan, Cote D'Ivoire; 2 Service des Maladies Infectieuses et Tropicale, CHU de Treichville, Infectious Diseases, Abidjan, Cote D'Ivoire; 3 INSERM, UMR S 1136, Institut Pierre Louis d'Epidémiologie et de Santé Publique, Public Health, Epidemiology, Paris, France; 4 INSERM U 897, Université de Bordeaux, Public Health, Bordeaux, France; 5 INSERM U 897, Université de Bordeaux, Statistics, Bordeaux, France; 6 Laboratoire de Virologie, Hôpital Saint-Louis, AP-HP, Virology, Paris, France

## Abstract

**Introduction:**

In West Africa, 10% of HIV-infected adults are co-infected with hepatitis B virus (HBV). The impact of HBV co-infection on mortality is unknown. We analyzed the association between HBV replication and mortality during long-term follow-up in the Temprano trial.

**Methods:**

Between March 2008 and July 2012, HIV-1 infected adults with CD4 <800/mm^3^ and no criteria for starting ART according to most recent WHO guidelines were randomized to deferred ART or early ART and to receive or not Isoniazid Preventive Therapy. At inclusion, hepatitis B surface antigen (HBsAg) was tested for all those included and plasma HBV DNA was quantified for HBsAg-positive samples using an in-house PCR technique (detection limit=2 copies/ml). All first-line ART regimens contained tenofovir/emtricitabine. Thirty-month mortality and severe morbidity were previously described in the paper reporting the final results of the trial. After their 30-month visits, all participants continued to be followed up until the last participants reached 30 months. Here we present mortality during and after the trial in all participants in Temprano. We used Cox regression to assess the risk of mortality in patients with high levels of HBV DNA at baseline, compared to other patients, adjusting for early/deferred ART and IPT.

**Results:**

Of the 2056 participants in Temprano (78% women, median age of 35 years, median CD4 count 465/mm^3^), 193 (9%) were HBsAg-positive. Of the 173 co-infected participants with available plasma HBV DNA, 119 (69%) had detectable HBV DNA (median 3880 copies/ml, IQR: 660–2,120,000), including 73 (42%) with HBV DNA >2000 copies/ml. Median follow-up time was 58 months (IQR: 40–69), totalling 9322 person-years (PY). During follow-up, 1814 (89%) patients started ART, 85 (4%) died and 187 (9%) were lost to follow-up. The incidence of mortality was 0.9/100 PY overall, 2.1/100 PY in HBsAg-positive patients with baseline HBV DNA >2000 copies/ml and 0.9/100 PY in other patients (p=0.02). In multivariate analysis, the risk of mortality was independently higher in patients with HBV DNA >2000 copies/ml (adjusted hazard ratio (aHR) 2.23, 95% confidence interval (CI): 1.02–4.85, p=0.04).

**Conclusions:**

In these West African HIV-infected adults with high baseline CD4 count, mortality was 2.2 times higher in patients with high levels of HBV replication.

# Sub article: WEAB0304LB TURQUOISE-I Part 2: safety and efficacy of ombitasvir + paritaprevir/r±dasabuvir with or without RBV in patients with HIV-1 and HCV GT1 or GT4 co-infection

**Published:** 2016-07-22

**Authors:** JK Rockstroh, C Orkin, RM Viani, D Wyles, A Luetkemeyer, A Lazzarin, R Soto-Malave, M Nelson, SR Bhagani, HHF Klinker, G Rizzardini, P-M Girard, NS Shulman, YB Hu, LM Fredrick, R Trinh, E Gane

**Affiliations:** 1 Universitätsklinikum Bonn, Bonn, Germany; 2 The Royal London Hospital, London, United Kingdom; 3 AbbVie, Inc., North Chicago, IL, United States; 4 University of California, San Diego, CA, United States; 5 San Francisco General Hospital, San Francisco, CA, United States; 6 Fondazione Centro San Raffaele del Monte Tabor, Milan, Italy; 7 Innovative Care P.S.C., Bayamon, Puerto Rico; 8 Chelsea and Westminster Hospital, London, United Kingdom; 9 Royal Free London Foundation Trust, London, United Kingdom; 10 Universitätsklinikum Wuerzburg, Wuerzberg, Germany; 11 ASST Fatebenefratelli Sacco, Milan, Italy; 12 Hopital Saint Antoine, Paris, France; 13 Liver Unit, Auckland City Hospital, Auckland, New Zealand

## Abstract

**Introduction:**

Ombitasvir, paritaprevir co-administered with ritonavir, and dasabuvir (OBV/PTV/r + DSV) comprise the three direct-acting antiviral (DAA; 3D) regimen±ribavirin (RBV) approved for HCV genotype (GT) 1 infection. Here we investigate the safety and efficacy of 3D±RBV for GT1, and the two DAA (2D) regimen of OBV + PTV/r approved for GT4, in HIV-1 co-infected patients with or without compensated cirrhosis.

**Methods:**

TURQUOISE-I, Part 2 is a phase 3 multicentre study. Eligible patients were HCV treatment-naïve or RBV/interferon-experienced, on an HIV-1 antiretroviral regimen containing atazanavir, raltegravir, dolutegravir, or darunavir (for GT4 only) and had plasma HIV-1 RNA <40 copies/mL at screening. Patients received OBV/PTV/r (25/150/100 mg) ±DSV (250 mg) ±weight-based RBV for 12 or 24 weeks per label guidelines. Interim safety and efficacy data are presented.

**Results:**

Table 1 presents baseline demographics on 227 treated patients as of 21 April 2016. Of the 194 GT1- and 26 GT4-infected patients with available data, 98 and 100% achieved sustained virologic response at post-treatment week (PTW) 4 (SVR4), respectively. Three patients experienced virologic failure: one GT1a patient relapsed at PTW4, a second relapsed at PTW12, and one GT1b patient experienced breakthrough at week 10. No patients discontinued treatment due to adverse events (AEs). Most AEs were mild to moderate in severity, and key lab abnormalities were rare (Table 2).

**Conclusions:**

The 2D and 3D regimens were well-tolerated and yielded high SVR4 rates in patients with HCV GT1 or GT4/HIV-1 co-infection. OBV + PTV/r±DSV±RBV is a potent HCV treatment option for patients with HIV-1 co-infection, regardless of treatment-experience or presence of compensated cirrhosis.

**Table 1:** Baseline demographics and disease characteristics

	GT1 N = 199	GT2 N = 28
Male, n (%)	156 (78)	26 (93)
White race, n (%)	172 (86)	25 (89)
Age, median (range), years	50 (26–69)	47 (30–63)
BMI, median (range), kg/m^2^	25 (17–41)*	24 (15–38)
HCV genotype 1a, n (%)	147 (74)	–
Cirrhosis, n (%)	22 (11)	0
Treatment-experienced, n (%)	64 (33)†	11 (39)
HCV RNA. median (range), log_10_IU/mL	6.5 (1.8–7.6)	6.0 (47–7.0)
CD4+ cell count, median (range), /μL‡	612 (133–2351)	731 (262–1533)

BMI, Body Mass Index

* N = 198

† N = 193

‡ N = 197GT1, N = 27 GT4

**Abstract WEAB0304LB–Table 2:** Safety and post-baseline laboratory abnormalities

Event, n (%)	GT1 N = 199	GT2 N = 28
Any AE	167 (84)	24 (86)
Serious AEs	9 (5)	1 (4)
RBV dose modifications due to hemoglobin decline	25 (13)	3 (11)
ALT Grade ≥3 (>5×ULN)	1 (1)	0
Total Bilirubin Grade >3 (>3 * ULN)	26 (13)	2 (7)
Patients on ATV-containing ART, n/N (%)	23/26 (88)	2/2 (100)
Hemoglobin Grade 2 (< 10 g/dL)	15 (8)	0
Hemoglobin Grade 3 (<8 g/dL)	0	0

AE, adverse event, RBV, ribavirin; ALT, alanine aminotransferase; ULN, upper limit of normal; ATV, atazanavir, ART, antiretroviral therapy

# Sub article: WEAB0305LB Hepatitis B viral load response to two antiviral regimens (tenofovir/lamivudine vs lamivudine) in HIV and HBV co-infected pregnant women in Guangxi, China: the Tenofovir in Pregnancy study

**Published:** 2016-07-22

**Authors:** A Kourtis, L Wang, J Wiener, S Liang, X Wei, W Liu, L Chen, C Shepard, A Wang, F Zhang, M Bulterys, TIP study Group

**Affiliations:** 1 US Centers for Disease Control and Prevention, Atlanta, GA, United States; 2 US Center for Disease Control and Prevention, Beijing, China; 3 China CDC, Nanning, China; 4 China CDC, Beijing, China

## Abstract

**Introduction:**

There is limited information on the value of HBV antiviral therapy during pregnancy to prevent transmission of HBV to the infant including agent choice and duration needed to achieve HBV viral load (VL) suppression by delivery.

**Methods:**

The Tenofovir in Pregnancy (TiP) study is a randomized controlled trial of the safety of a regimen containing tenofovir (TDF), lamivudine (3TC), and lopinavir/ritonavir, compared with zidovudine, lamivudine, and lopinavir/ritonavir, starting as early as 14 weeks gestation, in HIV/HBV co-infected pregnant women and their infants in Guangxi, China, recruited from 2012 to 2015. HBV VL response during pregnancy was compared in the two study arms, and associations of pre-treatment characteristics with such response were performed using Fisher's exact test and Poisson regression.

**Results:**

Thirty one of 35 women enrolled have delivered. The baseline median HBV VL was 4.01 log_10_ copies/ml in the TDF/3TC arm and 3.64 log_10_ copies/ml in the 3TC arm; proportions of HBeAg+ women were 38 and 20%, and median duration of antiviral therapy was 20 and 19 weeks, respectively. At delivery, 50.0% of mothers in the TDF/3TC arm and 73.3% in the 3TC-only arm achieved undetectable HBV VL (p = 0.27). The median decline of HBV DNA between enrolment and delivery was 2.60 log_10_ copies/ml in the TDF-3TC arm and 2.24 log_10_ copies/ml in the 3TC arm (p = 0.41). All women achieved delivery HBV DNA levels <6 log_10_ copies/ml. In multivariable analysis, maternal baseline HBV VL >200,000 IU/ml was the only factor significantly associated with not reaching undetectable HBV VL at delivery (relative risk = 0.12, 95% CI: 0.02–0.78).

**Conclusions:**

Initiation of HBV antiviral drugs from 14 to 28 weeks of gestation achieved HBV DNA suppression in 61% of pregnant women co-infected with HIV/HBV with no difference in the proportion of women achieving undetectable HBV DNA at delivery in the TDF/3TC or 3TC arms. Initiation of either regimen in the second trimester of pregnancy led to all women achieving HBV DNA level <6 log_10_ copies/ml at delivery, the threshold thought to predict breakthrough HBV transmission to the infant.

# Sub article: WEAC0102 Efficacy of on-demand PrEP with TDF-FTC in the ANRS IPERGAY open-label extension study

**Published:** 2016-07-22

**Authors:** J-M Molina, I Charreau, B Spire, L Cotte, J Chas, C Capitant, C Tremblay, D Rojas-Castro, E Cua, A Pasquet, C Bernaud, W Rozenbaum, C Delaugerre, V Doré, S Le Mestre, M-C Simon, J-F Delfraissy, L Meyer, ANRS IPERGAY Study Group

**Affiliations:** 1 INSERM UMR 941, University of Paris Diderot, Paris, France; 2 Infectious Diseases Department, Hospital Saint-Louis, APHP, Paris, France; 3 INSERM, SC 10-US 19, Villejuif, France; 4 INSERM, UMR 912, Marseille, France; 5 Infectious Diseases Department, Hospices Civils de Lyon, Lyon, France; 6 Infectious Diseases Department, Hospital Tenon, APHP, Paris, France; 7 Centre de Recherche du Centre Hospitalier de l'Université de Montréal, Montréal, Canada; 8 AIDES, MIRE, Pantin, France; 9 Infectious Diseases Department, Hospital de l'Archet, Nice, France; 10 Infectious Diseases Department, Hospital Gustave Dron, Tourcoing, France; 11 Infectious Diseases Department, CHU Hôtel Dieu, Nantes, France; 12 Virology Department, Hospital Saint-Louis, Paris, France; 13 INSERM-ANRS, Paris, France; 14 INSERM, Paris 11 University, Kremlin Bicêtre, France

## Abstract

**Introduction:**

In ANRS IPERGAY, on-demand pre-exposure prophylaxis (PrEP) with TDF-FTC reduced the incidence of HIV-1 infection in high-risk men who have sex with men (MSM) by 86% (Table 1). However, the cumulative follow-up time on TDF-FTC was limited, and the long-term efficacy and safety of this strategy remains to be assessed.

**Methods:**

From November 2014 to June 2016, participants (pts) who were followed or being screened in the ANRS IPERGAY trial were offered to continue follow-up every 2 months with open-label TDF-FTC. The primary study objectives of this open-label phase were to assess study retention, HIV incidence, safety and changes in sexual behaviour.

**Results:**

Among the 400 pts initially enrolled in the study, 336 (84%) were eligible for the open-label phase, and all but three (99%) signed a new informed consent form. Twenty-nine additional pts were also enrolled. Overall, 362 pts were enrolled for a cumulative follow-up time of 334 person-years (py), until 14 December 2015, with a median follow-up of 11.7 months. Study retention was good with only 23 pts discontinuing follow-up (6.4%). Only a single individual who had discontinued PrEP acquired HIV-1 infection and the overall incidence of HIV-1 infection was 0.3 per 100 py (95% CI: 0.00–1.67) (Table 1). Pts used a mean of 18 pills/month and 39% acquired a new sexually transmitted infection (STI). There were no significant changes between the double-blinded phase and the open-label phase in the median number of sexual intercourses or sexual partners, but there was a significant decrease in condom use for receptive anal intercourse (p=0.0004). Safety was good with a low rate of serious adverse events (6% of pts) and a single participant discontinued TDF-FTC because of an increase in creatinine plasma level. Drug-related gastrointestinal adverse events (mainly nausea and diarrhoea) were reported in 11% of pts.

**Conclusions:**

Open-label on-demand PrEP with oral TDF-FTC continued to be highly effective in high-risk MSM to prevent HIV infection and had a good safety profile.

**Abstract WEAC0102–Table 1:** Incidence of HIV infection according to IPERGAY phase

IPERGAY phase	Person-years of follow-up	Incidence of HIV infection per 100 person-years (95% CI)
IPERGAY double-blinded (placebo arm)	212	6.60 (3.61–11.07)
IPERGAY double-blinded (TDF-FTC arm)	219	0.91 (0.11–3.30)
IPERGAY open-label extension (open-label TDF-FTC)	334	0.30 (0.00–1.67)

# Sub article: WEAC0103 HPTN 073: successful engagement of Black MSM into a culturally relevant clinical trial for pre-exposure prophylaxis

**Published:** 2016-07-22

**Authors:** C Hucks-Ortiz, JP Lucas, DP Wheeler, SD Fields, The HPTN Black Caucus

**Affiliations:** 1 Division of HIV Services, John Wesley Community Health Institute, Inc. (JWCH), Los Angeles, CA, United States; 2 Science Facilitation Department, FHI 360, Durham, NC, United States; 3 School of Social Welfare, University at Albany State University of New York, Albany, NY, United States; 4 Mervyn M. Dymally School of Nursing, Charles R Drew University of Medicine and Science, Los Angeles, CA, United States

## Abstract

**Introduction:**

In the United States, Black men who have sex with men (BMSM) continue to be disproportionately impacted by high HIV incidence rates. Comprising less than 0.4% of the US population, BMSM accounted for more than 20% of new HIV infections in 2013. Identifying innovative and effective methods to deliver culturally tailored prevention methods to end this epidemic among BMSM is a public health priority. HPTN 073 is one of the first US studies to evaluate pre-exposure prophylaxis (PrEP) in a BMSM cohort.

**Methods:**

HIV-uninfected BMSM were enrolled in three US cities (Washington, DC; Los Angeles, CA; and Chapel Hill, NC). Under the study motto “My Life, My Health, My Choice”, all participants were offered once daily oral FTC/TDF combined with client-centred care coordination (C4). The C4 model provided counselling support to promote and support PrEP use, along with service referral, linkage, and follow-up strategies to assist participants in addressing unmet psychosocial needs. Each participant was followed for a total of 12 months.

**Results:**

All of the staff at the three sites were asked to participate in cultural responsiveness training as a part of implementation activities and then utilized a variety of culturally relevant recruitment and retention techniques such as webinars, street and online outreach, peer-to-peer engagement and partnerships with community service organizations. A total of 344 BMSM were screened and 226 were enrolled in HPTN 073. Among which, 209 (92%) participants completed 12 months of follow-up. Forty percent (40%) were aged 25 or less, 27% were unemployed/disabled, 31% did not have health insurance, 25% reported high school graduation or less. Among the total number enrolled, 178 men (79%) accepted PrEP over the course of the study.

**Conclusions:**

HPTN 073 demonstrated that BMSM can be successfully recruited, engaged, enrolled and retained in PrEP biomedical clinical trials using theory-based culturally tailored techniques. HPTN 073 provides a model for how best to integrate culturally specific recruitment approaches when targeting communities at risk for HIV acquisition. Utilizing theory-based culturally tailored programmes for BMSM that are reflective of their reality is the key to reaching this highly at-risk population of MSM who can benefit from the new HIV prevention biomedical advances.

# Sub article: WEAC0104 Correlates for levels of self-reported PrEP adherence among Black men who have sex with men in three US cities

**Published:** 2016-07-22

**Authors:** D Wheeler, S Fields, L Nelson, L Hightow-Weidman, M Magnus, S Shoptaw, G Beauchamp, L Emel, E Piwowar-Manning, Y Chen, P Watkins, K Mayer, HPTN073 Study Team

**Affiliations:** 1 State University of New York at Albany, Social Welfare, Albany, NY, United States; 2 Charles Drew University, Nursing, Los Angeles, CA, United States; 3 University of Rochester, Nursing, Rochester, NY, United States; 4 University of North Carolina, Chapel Hill, United States; 5 George Washington University, Washington, DC, United States; 6 University of California Los Angeles, Los Angeles, CA, United States; 7 Statistical Center for HIV/AIDS Research & Prevention (SCHARP), Seattle, WA, United States; 8 Fred Hutchinson Cancer Research Center, Seattle, WA, United States; 9 Johns Hopkins University, Baltimore, MD, United States; 10 FHI 360, NC, Durham, United States; 11 The Fenway Institute, Boston, MA, United States; 12 Harvard University, Cambridge, MA, United States

## Abstract

**Introduction:**

HPTN 073 study assessed the initiation, acceptability, safety and feasibility of pre-exposure prophylaxis (PrEP) for Black men who have sex with men (BMSM) in three US cities. Upon the PrEP initiation, the levels of PrEP use were monitored using self-reported adherence.

**Methods:**

HPTN 073 study enrolled 226 HIV-uninfected BMSM in three US cities (Los Angeles, CA; Washington DC; and Chapel Hill, NC, between August 2013 and September 2014). All study participants were offered once daily oral FTC/TDF and client centred care coordination, and were followed for 12 months, with scheduled clinical visits every 13 weeks.

**Results:**

Among the total 226 enrolled participants, 178 (79%) participants initiated PrEP. Proportions of self-reported high PrEP adherence (≥90%) ranged between 62 and 71%, while self-reported low PrEP adherence (<50%) ranged between 13 and 19% during weeks 13 through 52. High adherence is associated with age ≥25, higher education, full-time employment, no poly drug use and having a primary partner. Conversely, low adherence is associated with younger age, less education, non-full-time employment, poly drug use and no primary partner. Adjusted analysis shows that having a primary partner and no poly drug use are highly associated with high adherence, whereas converse is true for low adherence.

**Conclusions:**

Understanding the contextual factors that support and impede adherence (Figure 1) and targeting these in comprehensive intervention packages may maximize PrEP adherence and minimize lower adherence for BMSM. Our data support consideration of the need for addressing these factors as core elements for BMSM.

**Figure 1:** Self-reported PrEP adherence.

**Abstract WEAC0104–Table 1:** Correlates of self-reported adherence

	≥90% Self-reported adherence			<50% Self-reported adherence		
		
	OR (95% CI)	AOR (95% CI)	AOR p-value	OR (95% CI)	AOR (95% CI)	AOR p-value
Age ≥25	2.08 (1.25, 3.45)*	1.46 (0.84, 2.54)	0.1782	0.48 (0.26, 0.86)	1.49 (0.76, 2.95)	0.2482
Two-year degree or higher vs. HS or less Some college or vocational vs. HS or less	2.48 (1.28, 4.80)* 1.11 (0.59, 2.08)	1.59 (0.77, 3.28) 1.02 (0.52, 1.99)	0.2090 0.9513	0.34 (0.15, 0.74) 1.10 (0.55, 2.20)	0.46 (0.18, 1.18) 1.06 (0.46, 2.45)	0.1057 0.8922
Employed FT vs. unemployed PT or self-employed vs. unemployed	2.66 (1.39, 5.11)*1.04 (0.56, 1.95)	1.77 (0.85, 3.70) 1.01 (0.52, 1.99)	0.1275 0.9663	0.34 (0.16, 0.76)*0.90 (0.45, 1.81)	0.76 (0.28, 2.04)1.09 (0.47, 2.52)	0.5801 0.8372
Poly drug use	0.46 (0.22, 0.94)*	0.49 (0.24, 0.99)	0.0460	3.22 (1.36, 7.60)*	3.30 (1.37, 7.96)	0.0079
Primary partner	1.71 (1.09, 2.69)*	1.75 (1.10, 2.79)	0.0179	0.44 (0.24, 0.82)*	0.42 (0.22, 0.82)	0.0104

AOR, adjusted odds ratio.

* The factors included in the adjusted models are the factors that are <0.05 significance level in the unadjusted model. Analysis was done using generalized estimating equation with exchangeable covariance structure. The behavioural questions were asked for the past 3 months at each visit.

# Sub article: WEAC0105 Integrated delivery of PrEP and ART results in sustained near elimination of HIV transmission in African HIV serodiscordant couples: final results from The Partners Demonstration Project

**Published:** 2016-07-22

**Authors:** J Baeten, R Heffron, L Kidoguchi, N Mugo, E Katabira, E Bukusi, S Asiimwe, J Morton, K Ngure, N Bulya, J Odoyo, E Tindimwemba, J Haberer, M Marzinke, D Donnell, C Celum

**Affiliations:** 1 University of Washington, Seattle, WA, United States; 2 Kenya Medical Research Institute, Nairobi, Kenya; 3 Makerere University, Kampala, Uganda; 4 Kabwohe Clinical Research Centre, Kabwohe, Uganda; 5 Jomo Kenyatta University of Agriculture and Technology, Nairobi, Kenya; 6 Massachusetts General Hospital, Boston, MA, United States; 7 Johns Hopkins University, Baltimore, MD, United States; 8 Fred Hutchinson Cancer Research Center, Seattle, WA, United States

## Abstract

**Introduction:**

Antiretroviral therapy (ART) used by HIV-infected individuals and pre-exposure prophylaxis (PrEP) by HIV-uninfected individuals are highly efficacious HIV prevention tools. Assessing the effectiveness of these interventions and integrated delivery strategies in implementation settings is a priority.

**Methods:**

The Partners Demonstration Project, an open-label PrEP and ART delivery study, began in 2012 and enrolled antiretroviral-naïve, high-risk, heterosexual HIV serodiscordant couples from Kenya and Uganda. Couples were followed for 2 years. ART was recommended following national ART guidelines – initially CD4 <350 cells/µl but later for all HIV serodiscordant couples regardless of CD4 count. PrEP was offered as a “bridge” to ART in the partnership – that is, until ART initiation by the HIV-infected partner and for the first 6 months after ART initiation. We compared and observed HIV incidence to a counterfactual simulation model using bootstrapping methods and constructed with data from a prior prospective study of HIV serodiscordant couples (the partners PrEP study, placebo arm). In a previously reported interim analysis, with ~40% of total expected follow-up time accrued, we found that HIV incidence was substantially reduced (two incident infections compared to 40 expected infections); updated findings are presented here.

**Results:**

Of 1013 couples enrolled, 67% had an HIV-positive female partner and the median age was 29. Among a randomly selected sample of HIV-negative partners receiving PrEP, tenofovir was detected in 82% of plasma samples (483/587 visits). ART was initiated by 92% of HIV-positive partners by 24 months and viral suppression (<400 copies/ml) was achieved in 90%. As of January 2016, counterfactual simulations predicted that 63 incident HIV infections would be expected (incidence rate 5.1 per 100 person years, 95% CI: 3.9–6.4). However, only five incident infections have been observed (incidence rate 0.3, 95% CI: 0.1–0.7), for sustained HIV relative risk reduction of 94% (95% CI: 85–98, p<0.001).

**Conclusions:**

An integrated PrEP and ART strategy is highly effective for preventing HIV transmission within HIV serodiscordant couples, showing near elimination in a high risk cohort. The Partners Demonstration Project will complete follow-up and analysis in June 2016 with final results available in July 2016.

# Sub article: WEAC0106LB SEARCH test and treat study in Uganda and Kenya exceeds the UNAIDS 90-90-90 cascade target by achieving 81% population-level viral suppression after 2 years

**Published:** 2016-07-22

**Authors:** M Petersen, L Balzer, D Kwarsiima, N Sang, G Chamie, J Ayieko, J Kabami, A Owaraganise, T Liegler, F Mwangwa, K Kadede, V Jain, A Plenty, G Lavoy, D Black, E Bukusi, C Cohen, T Clark, E Charlebois, M Kamya, D Havlir, SEARCH Study Team

**Affiliations:** 1 Berkeley School of Public Health, University of California, Berkeley, CA, United States; 2 University of California, San Francisco, CA, United States; 3 Harvard T H Chan School of Public Health, Boston, MA, United States; 4 Infectious Diseases Research Collaboration (IDRC), Kampala, Uganda; 5 Kenya Medical Research Institute (KEMRI), Nairobi, Kenya; 6 Makerere University, Kampala, Uganda

## Abstract

**Introduction:**

The SEARCH Study (NCT01864683; first phase endpoint 2017) is a cluster randomized trial evaluating a “test and treat” HIV and multi-disease prevention strategy in rural Uganda and Kenya. We evaluated interim population-level HIV cascade coverage achieved over 2 years in the 16 SEARCH intervention communities.

**Methods:**

We enumerated residents via baseline household census. HIV serostatus and plasma RNA were measured annually at multi-disease health campaigns followed by home-based testing for non-attendees. Streamlined antiretroviral therapy (ART) (EFV/TDF/ + FTC or 3TC), including patient-centred care and viral load counselling, was universally offered. At baseline and after 1- and 2-year follow-up, we estimated (1) proportion of baseline HIV+ adult (≥15 years) stable (>6mo/past year) residents previously diagnosed; (2) of these, proportion ever on ART; (3) of these, proportion with viral suppression (RNA <500 copies/ml). We estimated population viral suppression as a cascade product and via direct HIV RNA measurement, using inverse weights to adjust for missing measures.

**Results:**

Of 77,773 baseline adult stable residents, 55% were women, 53% farmers, and 20% <20 years. Baseline HIV prevalence was 9.9% (West Uganda: 6.3%; East Uganda: 3.3%; Kenya: 19.5%). We achieved high cascade coverage by follow up year 2 (Figure): (1) 97.4% (95% CI: 97.3%, 97.5%) were previously diagnosed; (2) 93.2% had received ART (95% CI: 92.6%, 93.9%); (3) 89.5% were suppressed (95% CI: 88.6%, 90.4%). Population viral suppression at year 2 was 81.3% (95% CI: 80.3%, 82.3%) based on the cascade product and 82.8% (95%CI: 80.2%, 85.3%) by adjusted direct measure. Coverage was high among men and mobile populations: 97.5% (95% CI: 97.4%, 97.7%) of men and 97.1% (95% CI: 96.8%, 97.5%) of mobile populations tested at least once; among baseline HIV+, 80.3% (95% CI: 78.4%, 82.2%) of men and 81.7% (95% CI: 78.3%, 85.1%) of mobile populations had at least one suppressed RNA level.

**Conclusions:**

Using a multi-disease community-based approach and patient-centred streamlined care, we increased population viral suppression from 45 to 81%, exceeding the UNAIDS 90-90-90 cascade target within 2 years in SEARCH intervention communities.

**Figure 1:** Cascade coverage and population-level viral suppression among baseline HIV+ adult stable residents of SEARCH Study intervention communities.

# Sub article: WEAC0202 Transgender patients at risk: ensuring access to PrEP in an NYC community health centre

**Published:** 2016-07-22

**Authors:** A Radix, P Carneiro, S Stephanos, S Mosher, P Meacher, U Belkind, I Evans-Frantz, F Brigham, A Fortenberry, L Comstock, R Vail, S Weiss, S Pena, S Golub

**Affiliations:** 1 Callen-Lorde Community Health Center, New York, United States; 2 Epidemiology, Mailman School of Public Health, Columbia University, New York, NY, United States; 3 Hunter College, City University of New York, New York, United States

## Abstract

**Introduction:**

Transgender women (TGW) are known to be disproportionately affected by HIV. Although less is known about transgender men (TGM) recent studies have highlighted elevated risk in this population, especially among those who identify as MSM. Pre-exposure prophylaxis (PrEP) is an effective biomedical intervention to prevent incident HIV infections, but adherence is reported to be lower among TGW. In January 2014 Callen-Lorde Community Health Center, an LGBT-focused clinic in NYC that predominantly cares for HIV-infected and at-risk clients, implemented PrEP services. One of the goals was to create a programme that was trans-inclusive.

**Description:**

Almost 1500 clients have accessed PrEP since implementation of the programme. Careful tracking of PrEP uptake revealed low involvement by transgender clients, with only five receiving PrEP in the first 6 months. Challenges included community-level lack of knowledge, provider and client under-estimation of HIV risk, especially among TGM, and lower rates of HIV-testing, resulting in fewer opportunities to discuss PrEP. The clinic responded by offering HIV screening during all new transgender intake appointments and distributing trans-inclusive education materials and PrEP education videos that included transgender/genderqueer actors. The clinic has intentionally become a more diverse work place with transgender counsellors, testers, patient navigators and nurses.

**Lessons learned:**

The interventions were successful. A total of 118 transgender clients have accessed PrEP over 3 years, 8.4% of total prescriptions written. The majority 71.2% (84) have been TGW, 10.2% (12) genderqueer, and 18.6% (22) TGM. The populations differed by insurance and race with public “safety net” coverage being predominantly used by genderqueer and TGW (67% and 60.7%), whereas TGM predominantly used commercial insurance (64%). TGW were mostly non-white (70%), whereas TGM and genderqueer people were predominantly white (81% and 78%).

**Conclusions/next steps:**

As scale-up of PrEP continues, clinics considering implementation of PrEP need to ensure that they track utilization to monitor disparities among users. Addressing PrEP uptake among transgender clients requires a multi-faceted approach.

**Figure 1:** Uptake of PrEP by transgender clients at The Callen-Lorde Community Health Center.

# Sub article: WEAC0203 LifeSkills: results from a full-scale, randomized controlled trial examining the efficacy of a group-based behavioural intervention for HIV prevention among young transgender women

**Published:** 2016-07-22

**Authors:** R Garofalo, L Kuhns, S Reisner, K Biello, M Mimiaga

**Affiliations:** 1 Feinberg School of Medicine, Northwestern University, Chicago, IL, United States; 2 Ann and Robert H. Lurie Children's Hospital of Chicago, Chicago, IL, United States; 3 Pediatrics, Boston Children's Hospital, Boston, MA, United States; 4 Pediatrics and Adolescent Medicine, Harvard Medical School, Boston, United States; 5 Fenway Health, The Fenway Institute, Boston, MA, United States; 6 Department of Epidemiology, Brown University, Providence, RI, United States; 7 Department of Behavioral and Social Sciences, Brown University, Providence, RI, United States

## Abstract

**Introduction:**

HIV prevalence is high among transgender women. A global meta-analysis of HIV burden among transgender women found a 19% HIV prevalence and 49-fold increased odds of HIV infection compared to all adults of reproductive age. A US-based meta-analysis found an overall 28% laboratory-confirmed HIV prevalence. No evidence-based interventions (EBIs) exist for HIV prevention among transgender women in the Centers for Disease Control and Prevention (CDC) compendium of EBIs. We addressed this gap by testing a culturally specific, behavioural intervention for HIV prevention (“LifeSkills”) among young transgender women (YTW) in a randomized controlled efficacy trial. LifeSkills is theoretically driven and grounded in the social realities of YTW, with content developed using a community-based participatory approach with guidance from a multidisciplinary research team.

**Methods:**

We recruited 300 YTW, aged 16–29, in two US cities (Boston and Chicago), who were randomly assigned 2:2:1 in a 3-arm (LifeSkills, standard-of-care, and time-matched attention control) trial examining the efficacy of a multi-session, group-based intervention for HIV prevention. Participants were followed for 1 year, with visits at 4, 8 and 12 months post-randomization. Enrolment was completed between 2012 and 2015, with follow-up visits through September 2016. Generalized linear models examined differences in condomless sex (CS) acts between intervention and control arms.

**Results:**

Participants were racially/ethnically diverse; 49% Black, 12% Latina, 25% White, and 14% other. At enrolment, 22% of participants were HIV-infected (3% previously undiagnosed). Interim analysis with >90% of completed visits indicates feasibility and efficacy of the intervention to reduce CS acts compared to the standard-of-care control arm. We found a >20% difference in reduction of CS acts (vaginal and anal) from baseline with a significant 12-month arm x time interaction (*F*(3447) =12.29, p<0.0001). Intervention participants reported high satisfaction with the curriculum: 98% indicated they would refer a friend and 99% said the intervention met their expectations.

**Conclusions:**

Using the CDC “Guide to the Continuum of Evidence for Efficacy” as a framework, LifeSkills may be the first well-supported, EBI for HIV prevention among YTW. Additional research is needed to demonstrate independent replication of findings and guide implementation and dissemination of LifeSkills in other US communities and regions of the world.

# Sub article: WEAC0204 Differences between unknown HIV-positive and HIV-negative Black transgender women in the United States: results from Promoting Our Worth, Equality, and Resilience (POWER)

**Published:** 2016-07-22

**Authors:** L Bukowski, S Meanley, J Egan, D Matthews, R Stall, The Power Study Team

**Affiliations:** Graduate School of Public Health, University of Pittsburgh, Pittsburgh, PA, United States

## Abstract

**Introduction:**

HIV disproportionately burdens Black transgender women (BTW) in the United States. Improving HIV testing uptake to identify unknown HIV-positive individuals is critical to attenuating the HIV epidemic in this population. Understanding demographic and psychosocial differences between HIV-positive BTW who are unaware of their status and HIV-negative BTW may help elucidate means by which to increase HIV testing uptake in this population. Therefore, this analysis explores possible differences between unknown HIV-positive BTW and HIV-negative BTW.

**Methods:**

Cross-sectional data for our analysis came from the first 2 years of the ongoing study, POWER. In 2014 and 2015, POWER employed time-location sampling (TLS) to recruit a community-based sample of Black men who have sex with men and BTW (n=3426) who attended Black Pride events in six US cities. Participants completed a behavioural health survey and were offered onsite HIV-testing. Unknown HIV-positive BTW were identified for analysis if they reported a *negative* HIV-status within the survey but provided a *positive* HIV antibody screening test result through on-site testing. Self-report HIV-negative status was confirmed with on-site testing. Differences in HIV-status (unknown vs. negative) were evaluated using TLS-weighted independent logistic regression models adjusted for age, education and city.

**Results:**

A total of 253 BTW provided complete data for our analysis. We observed HIV prevalence of 37.9%. Of the 96 HIV-positive BTW, 50.0% were unaware of their HIV-status. Compared to HIV-negative BTW, unknown HIV-positive BTW reported significantly higher prevalence of past-year physical assault (40.4% vs. 58.3%, respectively) and past 2-year incarceration (31.9% vs. 52.1%, respectively). In independent multivariable models, physical assault (AOR=2.1; 95% CI: 1.0–4.2) and incarceration (AOR=2.3; 95% CI: 1.1–4.7) were associated with greater likelihood of unknown positive status.

**Conclusions:**

Developing and implementing interventions that address experiences of physical assault and a history of incarceration may assist in informing the HIV disparity among BTW in the United States. More research is needed to identify and understand the structural, community, and individual-level barriers and facilitators that shape BTW's engagement with HIV-testing and HIV-care.

# Sub article: WEAC0205 Factors affecting HIV testing among transgender people in Ontario, Canada: results from a respondent-driven sampling survey

**Published:** 2016-07-22

**Authors:** GR Bauer, M Shokoohi, R Hammond, AI Scheim

**Affiliations:** 1 Department of Epidemiology & Biostatistics, Schulich School of Medicine & Dentistry, The University of Western Ontario, London, ON, Canada; 2 Regional Knowledge Hub, and WHO Collaborating Centre for HIV Surveillance, Institute for Futures Studies in Health, Kerman University of Medical Sciences, Kerman, Iran, Islamic Republic of; 3 Sherbourne Health Centre, Toronto, ON, Canada

## Abstract

**Introduction:**

In Ontario, a high proportion of trans (transgender, transsexual or transitioned) people have never been tested for HIV. Whether this can be explained by actual level of HIV risk or by other factors requires exploration. To date, no prior study has identified predictors of HIV testing among trans people.

**Methods:**

The Trans PULSE Project conducted a respondent-driven sampling survey to recruit trans Ontarians age ≥16 (n=433). Descriptive statistics were weighted by the probability of recruitment to estimate population frequencies. Regression models predicting both lifetime testing and past-year testing were weighted, and variances adjusted for clustering within recruitment networks.

**Results:**

Of Ontario trans people, 55.7% (95% CI: 47.9–63.6) had ever been self-reportedly tested for HIV, and 22.1% (95% CI: 15.9–28.3) were tested within the past 12 months. Common reasons for not being tested for HIV were perceptions of low risk (36.2%) and not having sex recently (23.1%). However, being aware of their status (39.4%) and routine check-up (38.8%) were the most frequent reasons for being tested for HIV. Lifetime testing was highest in Aboriginal people (92.6%) and lowest among non-Aboriginal racialized people (39.9%). Lifetime testing was predicted by ethno-racial group. For both testing timeframes, a history of transphobic experiences and higher lifetime number of sex partners predicted increased odds of testing. Past-year sex partner number had no detectable effect on past-year testing.

**Conclusions:**

While a lower testing prevalence was observed than in estimates from US studies, this may in part reflect the lower overall risk of this province-wide sample. That higher lifetime number of sex partners was associated with testing indicates a logical decision-making component. Multiple possibilities exist - ranging from resiliency, to confounding by social participation, to health cynicism - for the observation that a history of transphobic experiences was strongly associated with increased odds of testing. A range of possibilities for each of our findings and directions for additional research will be presented.

# Sub article: WEAC0301 The epidemiology of perinatally HIV-infected adolescents: a CIPHER cohort collaboration global analysis

**Published:** 2016-07-22

**Authors:** A Slogrove, A Judd, V Leroy, Collaborative Initiative for Paediatric HIV Education and Research (CIPHER) Global Cohort Collaboration

**Affiliations:** 1 Center for Infectious Disease Epidemiology and Research, University of Cape Town, Cape Town, South Africa; 2 University College London, London, United Kingdom; 3 INSERM, U 897, Bordeaux, France; 4 Institut de Sante Publique, d'Epidemiologie et de Developpement, University Bordeaux, Bordeaux, France

## Abstract

**Introduction:**

The population of perinatally HIV-infected adolescents (PHA) continues to expand globally. This study aims to describe the geographic and temporal characteristics and outcomes of PHA.

**Methods:**

Through the Collaborative Initiative for Paediatric HIV Education and Research (CIPHER), individual retrospective data from 12 cohort networks were pooled. Included PHA entered care before age 10 years with no known non-vertical route of HIV infection and were followed beyond age 10 years. This initial analysis describes characteristics at first visit, start of antiretroviral therapy (ART), start of adolescence (age 10 years) and surviving patients at last follow-up.

**Results:**

Of 37,614 PHA included, 49.4% (18,591) were male and 79% were from sub-Saharan Africa (Table 1). Median (interquartile range, or IQR) follow-up during adolescence was 2.36 (1.00–4.35) years, ranging from 2.04 (0.87–3.77, sub-Saharan Africa) to 6.38 (3.51–8.01, Europe & Central Asia) years.

In total, 90.7% (34,132) of PHA received ART; 9.9% (3385) started after age 10 years. Age, CD4 count, CD4 percent and HIV viral load at first visit and ART start varied markedly across regions (Table 2). Although laboratory markers improved by age 10 years, median weight-for-age (WAZ), height-for-age (HAZ) and body mass index-for-age (BMIZ) WHO Z-scores changed little. Median HAZ at age 10 years and last visit remained well below zero in all regions, although BMIZ was less impaired.

Reported mortality between age 10 and 15 years was 3.08% (95% CI: 2.83–3.36), ranging from 0.78% in Europe & Central Asia to 4.72% in South America & Caribbean (Table 1).

**Conclusions:**

Reported mortality during adolescence was <5% in all regions represented in this global analysis of HIV-infected children surviving to age 10 years. Under-ascertainment of mortality and impaired growth are concerns.

**Abstract WEAC0301–Table 1:** Countries, periods of observation, duration of follow-up and cumulative mortality between 10 and 15 years of age by region

Region	Countries included	N (%)	Observation period	Duration of follow-up during adolescence – median (IQR) years	Cumulative mortality % (95% CI)
South & Southeast Asia	Cambodia, India, Indonesia, Malaysia, Myanmar, Thailand, Vietnam	2902 (7.7)	1994–2014	2.53 (1.17; 4.37)	2.98 (2.08; 4.25)
Europe & Central Asia	Belgium, France, Ireland, Italy, The Netherlands, Poland, Portugal, Romania, Russian Federation, Spain, Sweden, Switzerland, Ukraine, United Kingdom	3058 (8.1)	1982–2015	6.36 (3.51; 8.01)	0.78 (0.50; 1.21)
South America & Caribbean	Argentina, Brazil, Haiti, Honduras	903 (2.4)	1990–2015	4.92 (2.68; 7.37)	4.72 (3.33; 6.65)
North America	United States of America	1048 (2.8)	1991–2014	3.73 (2.01; 5.43)	1.09 (0.52; 2.24)
Sub-Saharan Africa	Benin, Botswana, Burkina Faso, Burundi, Cameroon, Central African Republic, Democratic Republic of Congo, Côte d'Ivoire, Ethiopia, Ghana, Guinea, Kenya, Lesotho, Malawi, Mozambique, Rwanda, Senegal, South Africa, Swaziland, Tanzania, Togo, Uganda, Zambia, Zimbabwe	29,703 (79.0)	1996–2015	2.04 (0.87; 3.77)	3.59 (3.26; 3.96)

**Abstract WEAC0301–Table 2:** Age, laboratory and anthropometric characteristics of perinatally HIV-infected adolescents (N=37,614) and ranges of medians across regions

	First visit		ART start		Age 10 years (±6 months)		Last visit	
				
	Total median (IQR)	Min & max region medians	Total median (IQR)	Min & max region medians	Total median (IQR)	Min & max region medians	Total median (IQR)	Min & max region medians
N	37,614		34,132		37,614		36,872	
Age in years	6.7 (4.4; 8.4)	0.7; 7.1	7.4 (5.1; 9.1)	1.0; 7.8	Not applicable	Not applicable	12.4 (11.0; 14.4)	12.0; 16.4
CD4 count in cells/µl	430 (205; 761) N=19,388	255.5; 1282	330 (171; 598) N=19,368	221; 1134	686 (446; 972) N=26,282	639; 797	688 (465; 948) N=31,230	578; 744
CD4%	16 (9; 25) N=13,422	10; 30	14 (8; 20) N=14,564	10; 28	28 (20; 34) N=18,029	26; 33	29 (21; 35) N=23,249	27; 32
Log10 HIV viral load	5.00 (4.35; 5.58) N=4137	4.96; 5.28	4.94 (4.16; 5.51) N=6167	4.83; 5.10	2.42 (1.69; 3.35) N=10,155	1.69; 2.60	2.30 (1.60; 3.18) N=14,006	1.59; 2.60
WAZ (≤age 10 years)	–1.79 (–2.81; –0.90) N=21,037	–2.71; –0.51	–1.70 (–2.70; –0.83) N=22,908	–2.89; –0.41	–1.42 (–2.18; –0.59) N=30,705	–1.93; 0.09	Not applicable	Not applicable
HAZ (all ages)	–1.92 (–2.91; –0.97) N=20,013	–2.37; –0.77	–1.98 (–2.94; –1.05) N=19,801	–2.44; –0.78	–1.54 (–2.36; –0.72) N=26,645	–1.91; –0.32	–1.60 (–2.46; –0.73) N=32,386	–1.78; –0.34
BMIZ (≥age 5 years)	–0.60 (–1.54; 0.22) N=19,892	–1.44; 0.16	–0.56 (–1.46; 0.25) N=19,697	–1.46; 0.20	–0.54 (–1.26; 0.13) N=26,530	–1.00; 0.38	–0.68 (–1.46; 0.09) N=32,295	–1.02; 0.50

# Sub article: WEAC0302 Prevalence and predictors of forced-sex among South African high school students

**Published:** 2016-07-22

**Authors:** S Naidoo, B Sartorius, H de Vries, M Taylor

**Affiliations:** 1 Discipline of Public Health Medicine, University of KwaZulu-Natal, Durban, South Africa; 2 Discipline of Public Health, University of KwaZulu-Natal, Durban, South Africa; 3 Department of Health Organisation, Policy and Economics Management, Maastricht University, Maastricht, The Netherlands

## Abstract

**Introduction:**

Gender violence in South Africa is a public health problem, including among adolescents [1,2]. Prevalence of sexual violence in adolescents ranges from 10 to 17% [1,2]. Forced sex, given the South African HIV epidemic, is a risk factor for HIV transmission. Understanding the predictors of forced sex among adolescents is important in developing preventative strategies.

**Methods:**

This study aimed to identify the prevalence and predictors of forced sex in high school students in 16 randomly selected schools in Ugu and eThekwini districts of KwaZulu-Natal, South Africa. All students in a single randomly selected grade 10 class at each school were invited to participate. Parents/guardians gave informed written consent, and students consented to participate in the study. The study had ethical approval from the Biomedical Research ethics Committee of the University of KwaZulu-Natal and the Provincial Department of Basic Education. The I-Change Theoretical model was used as a conceptual framework for development of a self-administered questionnaire which included questions on socio-economic status. Survey weights were utilized given the study's complex multi-stage random sampling strategy. Point estimates and associated 95% confidence intervals were calculated. Factor analysis was employed to identify underlying factors associated with the construct variables related to forced sex. Survey-weighted multivariable regression was performed to assess factors associated with forced sex status. Population attributable fractions for risk factors associated with forced sex were estimated.

**Results:**

Overall 54 out of 434 subjects reported forced sex (survey weighted prevalence: 14.2%, 95%CI: 9.1–21.5%). The prevalence of reported forced sex was higher amongst females at 15.0% (95% CI: 10.8–20.4) compared to 13.6% (95% CI: 6.5–26.5) amongst males (p-value=0.781). There was a higher prevalence of forced sex amongst students in the low SES category (24.8%; 95% CI: 11.6–45.4) compared to the combined medium-high SES categories (12.9%; 95% CI: 8.8–18.5) (p-value=0.036). After multivariable adjustment, urban location (39%), low SES (15%) and discordant mother/father vital status (20%) (specifically mother alive and father deceased) remained high-impact risk factors for forced sex.

**Conclusions:**

Public health and socio-economic interventions addressing household economics and family structure in urban communities are required to reduce the risk of forced sex among adolescents in South Africa.

# Sub article: WEAC0303 The impact of a cash transfer on young South African women's on mental health: HPTN 068

**Published:** 2016-07-22

**Authors:** A Pettifor, J Wang, A Selin, J Hughes, X Gómez-Olivé, R Wagner, C MacPhail, K Kahn, HPTN 068 Study Team

**Affiliations:** 1 Epidemiology, University of North Carolina at Chapel Hill, Chapel Hill, NC, United States; 2 SCHARP, Seattle, WA, United States; 3 Carolina Population Center, University of North Carolina at Chapel Hill, Chapel Hill, NC, United States; 4 MRC/Wits Rural Public Health and Health Transitions Unit, University of the Witwatersrand, Johannesburg, South Africa; 5 School of Health, University of New England, Armindale, Australia

## Abstract

**Introduction:**

Cash Transfers have been found to improve the mental health of recipients. Possible mechanisms for the improvement in mental health include a reduction in financial stress and a hope for a better future due to an improved financial situation.

**Methods:**

HPTN 068 was a 3-year randomized controlled trial to assess the impact of a cash transfer, conditioned on school attendance, on HIV incidence among young rural South African women. A total of 2328 young women were HIV negative at baseline and had at least one follow-up visit. Young women completed a survey using Audio Computer Assisted Self-Interview at baseline and at 12, 24 and 36 months. We assessed depression and anxiety using: the Short Form Children's Depression Index (CDI), The Center for Epidemiologic Studies Depression Score (CES-D) and the Revised Children's Manifest Anxiety Scale. Hope was measured with the Abler Hope Scale. CDI (≥7) and CES-D (≥16) were analyzed using log-binomial regression and robust variance to account for repeated measures. CMAS (summed score) and Hope (summed score) were analyzed using generalized estimating equations (GEE) with identity link, normal distribution and robust variance to account for repeated measures.

**Results:**

Overall, we saw no association between receipt of the conditional cash transfer and reduced depression or anxiety among young women (Table 1). In addition, there was no association between receipt of the cash transfer and increased hope for the future (Table 1).

**Conclusions:**

In this randomized control trial of a cash transfer, conditional on school attendance, we saw no impact of receiving the cash on depression, anxiety or hope for the future. High levels of school attendance and social protection coverage were observed in the cohort, and thus, it is possible that the addition of the cash transfer did not meaningfully reduce anxiety about poverty or improve future outlook above the baseline levels.

**Abstract WEAC0303–Table 1:** Association between cash transfer programme and mental health outcomes in young South African women, HPTN 068

Outcomes Range, Cronbach's alpha	CCT n=1214	Control n=1114	RR	95% CI	p
CDI Index (≥7)^a^, alpha=0.70	25.8%	25.9%	0.99	0.85–1.16	0.93
CES-D (≥16)^a^, alpha=0.84	28.4%	29.9%	0.96	0.86–1.06	0.39
CMAS Anxiety (0–14), alpha=0.89	2.51	2.72	–0.21^b^	–0.53–0.11	0.19
Abler Hope (0–39), alpha=0.97	31.9	32.0	–0.14^b^	–0.75–0.48	0.66

a ≥7 for CDI and ≥16 for CES-D indicates depressive symptoms.

b Risk difference.

# Sub article: WEAC0304 Why the disparities? The first national look at HIV-related risk behaviours among gay and bisexual male high school students, United States 2015

**Published:** 2016-07-22

**Authors:** L Kann, E Olsen, T McManus, S Zaza

**Affiliations:** Division of Adolescent and School Health, Centers for Disease Control and Prevention, Atlanta, GA, United States

## Abstract

**Introduction:**

In 2014, an estimated 22% of all new diagnoses of HIV in the United States occurred among 13- to 24-year-olds, and most of these diagnoses occurred among males who have sex with males. The 2015 national Youth Risk Behavior Survey (YRBS) is the first national survey in the United States to provide national estimates of the size of the sexual minority population in high schools and document the disparities in HIV-related risk behaviours between gay/bisexual and heterosexual male high school students.

**Methods:**

The 2015 national YRBS employed a three-stage national probability sample of 15,624 students in grades 9–12 (ages 14–17). Black and Hispanic students were oversampled. T-tests were used to determine significant pairwise differences between gay/bisexual and heterosexual male high school students.

**Results:**

Nationwide, 2.0% of male high school students identified as gay, and 2.4% identified as bisexual. Gay/bisexual male students were at least twice as likely as heterosexual male students to report being electronically bullied; bullied on school property; not going to school because of safety concerns; being physically forced to have sexual intercourse; experiencing physical and sexual dating violence; ever using cocaine, heroin, and methamphetamines; and ever injecting drugs. However, no significant differences were identified between gay/bisexual male students and heterosexual male students in ever drinking alcohol, ever using marijuana, ever having sexual intercourse, having sexual intercourse with four or more persons, being currently sexually active, using a condom at last sexual intercourse and drinking alcohol or using drugs before last sexual intercourse.

**Conclusions:**

Though males who have sex with males are disproportionally affected by HIV, behaviours that directly contribute to HIV infection (e.g. not using a condom) do not appear to be driving the disparities at least among male high school students nationwide. Nonetheless, the results clearly demonstrate significant disparities in many other health-risk behaviours that could present barriers and decrease access to HIV prevention and treatment technologies among gay/bisexual male students. The results also suggest the importance of addressing broader social determinants of health associated with increased risk for HIV infection including stigma, discrimination, lower educational attainment, unemployment and incarceration.

# Sub article: WEAC0305LB Changes in bone mass after discontinuation of PrEP with tenofovir disoproxil fumarate/emtricitabine (TDF/FTC) in young men who have sex with men: extension phase results of Adolescent Trials Network (ATN)110

**Published:** 2016-07-22

**Authors:** K Mulligan, S Hosek, BG Kapogiannis, RJ Landovitz, N Liu, SS Cofield, SE Perumean-Chaney, PL Havens, B Rutledge, CM Wilson, Adolescent Trials Network (ATN for Hiv/aids Interventions Protocol 110 Team

**Affiliations:** 1 University of California, San Francisco, CA, United States; 2 John Stroger Hospital of Cook County, Chicago, IL, United States; 3 National Institute of Child Health and Human Development, National Institutes of Health, Bethesda, MD, United States; 4 University of California, Los Angeles, CA, United States; 5 Westat, Rockville, MD, United States; 6 University of Alabama, Birmingham, AL, United States; 7 Medical College of Wisconsin, Milwaukee, WI, United States

## Abstract

**Introduction:**

PrEP with TDF/FTC is associated with modest bone loss in HIV-seronegative adults and adolescents. There is particular concern about bone loss during adolescence/early adulthood, a period of continuing bone growth. The aim of this study was to determine whether bone loss reversed with discontinuation of PrEP in HIV-seronegative young men who have sex with men (YMSM) ages 18–22.

**Methods:**

ATN110 is a 48-week open-label demonstration and safety study of TDF/FTC PrEP in 200 YMSM. As part of safety monitoring, bone mineral density (BMD) was measured by dual-energy X-ray absorptiometry (DXA). Participants who lost or failed to accrue bone after 48 weeks on TDF/FTC PrEP immediately entered an extension phase (EPH) in which DXA scanning was performed 24 and 48 weeks after discontinuation of PrEP. Results are mean±SD.

**Results:**

Of 135 participants who had DXA scans at the end of the 48-week TDF/FTC treatment phase, 105 (78%) were eligible for EPH. After exclusion of seroconverters (N = 6) and those who received PrEP through their regular providers (N = 16), EPH data are available for 74 participants. Among this group, average BMD changes from baseline to week 48 of the treatment phase were: spine −0.2±2.7% (P = 0.53); hip −1.4±3.6% (P = 0.002); whole body (WB) −0.6±2.5% (P = 0.03). Forty-eight weeks after discontinuation of TDF/FTC, BMD increased (spine +1.1±3.0% (P = 0.003); hip +1.0±3.8% (P = 0.04); WB +0.6±2.0% (P = 0.01)). Net BMD changes from baseline to the end of EPH (48 weeks on TDF/FTC followed by 48 weeks off TDF/FTC) were not statistically significant (spine +0.6±4.1% (P = 0.24); hip −0.5±4.2% (P = 0.34); WB −0.2±2.6% (P = 0.52)). Despite gains in BMD during EPH, there were small but statistically significant net decreases from baseline in Z-scores (SDs based on population norms for BMD) in the spine (−0.18±0.38 (P < 0.001)) and WB (−0.08±0.31 (P = 0.03)), with no significant change in the hip (−0.05±0.30 (P = 0.22)).

**Conclusions:**

On average, HIV-seronegative YMSM who lost BMD during TDF/FTC PrEP experienced partial or full recovery of BMD during the 48 weeks following discontinuation of PrEP, but some Z-scores declined slightly from baseline. While the risk of slight BMD loss is counterbalanced by protection from HIV acquisition, these results highlight the continuing need for strategies to mitigate bone loss in at-risk YMSM.

# Sub article: WEAC0401 Long-term alcohol use patterns and HIV disease severity typologies in US veterans: a joint trajectory analysis

**Published:** 2016-07-22

**Authors:** B Marshall, J Tate, K McGinnis, D Fiellin, K Bryant, A Justice

**Affiliations:** 1 Department of Epidemiology, Brown University, Providence, RI, United States; 2 Department of Internal Medicine, Yale University, New Haven, CT, United States; 3 Veterans Aging Cohort Study Coordinating Center, West Haven VA Healthcare System, West Haven, CT, United States; 4 VA Pittsburgh Healthcare System, Pittsburgh, PA, United States; 5 National Institute on Alcohol Abuse and Alcoholism, Bethesda, MD, United States

## Abstract

**Introduction:**

Although unhealthy alcohol use is common in HIV-infected populations, the effect of alcohol consumption on HIV disease progression is unclear. We examined the relationship between long-term alcohol use patterns and HIV disease severity among participants enrolled in the Veterans Aging Cohort Study (VACS).

**Methods:**

HIV-infected participants in care at eight US Veterans Health Administration sites were eligible. Between 2002 and 2010, we assessed alcohol consumption annually using the 3-item Alcohol Use Disorders Identification Test-Consumption (AUDIT-C). Overall disease severity was ascertained using the VACS index, a validated measure of morbidity and mortality. We identified trajectories of alcohol use and disease severity with group-based finite mixture modelling. We examined associations between membership in distinct alcohol use and VACS index trajectories using multinomial regression.

**Results:**

Of 3539 eligible participants, median age was 49 (IQR: 44–55), 98% were male, and 70% were African American. Group-based modelling identified four alcohol consumption patterns: abstainers (24%), low-risk drinkers (44%), moderate-risk drinkers (24%) and high-risk drinkers (8%) (left panel). We also found four VACS index trajectories: low (2% of sample), moderate (46%), high (36%) and extreme (16%) (centre panel). Membership in higher VACS index trajectories was associated with older age, African American race, HCV co-infection, history of injection drug use and lack of viral suppression (all p<0.001). Membership in VACS index and alcohol consumption trajectories was strongly correlated (right panel). No high-risk drinkers were in the low VACS Index group, whereas high-risk drinkers were most common in the extreme group. Abstainers were most common in the low and extreme VACS Index groups.

**Conclusions:**

Alcohol use patterns implying long-term hazardous drinking were associated with greater disease severity among HIV-infected veterans receiving care. Joint trajectory analyses revealed two distinct groups of abstainers (“sick quitters” and “healthy abstainers”). Further research is needed to identify mediators of long-term alcohol consumption patterns and HIV disease severity.

**Abstract WEAC0401–Figure 1:** Joint alcohol use and VACS index trajectories among study participants.

# Sub article: WEAC0402 Extended-release naltrexone lengthens time to heavy drinking among HIV+ released prisoners with alcohol use disorders

**Published:** 2016-07-22

**Authors:** S Springer, M Azar, R Barbour, A Krishnan, F Altice, A DiPaola

**Affiliations:** 1 Yale AIDS Program, Section of Infectious Disease, Yale School of Medicine, New Haven, CT, United States; 2 Center for Interdisciplinary Research on AIDS, Yale University School of Medicine, New Haven, CT, United States; 3 Department of Communication, State University of New York at Albany, Albany, NY, United States

## Abstract

**Introduction:**

Alcohol use disorders (AUDs) negatively impact every step in the HIV continuum of care. For HIV+ prisoners in particular, relapse to heavy alcohol use upon release is associated with poor retention in care and loss of HIV viral suppression. Extended-release naltrexone (XR-NTX) is an approved and effective monthly injectable medication to prevent relapse to alcohol use but has not been studied among HIV+ persons or among prisoners.

**Methods:**

We conducted a NIAAA-funded double blinded placebo-controlled trial of XR-NTX (randomized 2:1, XR-NTX: placebo) among HIV+ prisoners with AUDs who were released to the community in Connecticut, USA. Primary outcome of interest was time to first heavy drinking day (TFHDD). Due to elevated data missingness, a Little's MCAR test was first performed and confirmed that the data were missing at random. This missingness structure allowed multiple imputation and subsequent multivariate analysis via Bayesian modelling. A heavy drinking day was defined as ≥5 drinks for males and ≥4 drinks for females. Intervention time was 6 months, and total follow-up period was 12 months.

**Results:**

In total, 107 HIV+ prisoners were enrolled during the study period from 2010 to 2015. The first study drug injection occurred 1 week prior to release during incarceration, and five subsequent injections occurred monthly after release to the community. TFHDD was significantly longer in those that received XR-NTX versus placebo (80.4 vs. 73.5 days; p<0.001). In addition to the overall treatment effect of XR-NTX; age<30 years, lower Alcohol Use Disorder Identification Test (AUDIT) scores, and abstinence from opioids and/or cocaine during the intervention period were significantly associated with longer TFHDD (p<0.001).

**Conclusions:**

XR-NTX significantly lengthened the time to heavy drinking after release for HIV+ released prisoners, particularly among younger persons. Interventions aimed at preventing relapse to alcohol among HIV+ prisoners transitioning to the community should include XR-NTX.

**Abstract WEAC0402–Figure 1:** Kaplan Meier plot of time to first heavy drinking day by age & and study arm.

# Sub article: WEAC0403 Preliminary experience with medically assisted therapy for people who inject drugs in Mombasa County, Kenya

**Published:** 2016-07-22

**Authors:** AA Baghazal, L Tariko, K Shikely, S Patta, B Omar, H Musyoki, S Bertrand, S Abdallah

**Affiliations:** 1 County Government of Mombasa, Health, Mombasa, Kenya; 2 Health Records, Coast General Hospital, Mombasa, Kenya; 3 National AIDS and STI Control Programme, Ministry of Health, Nairobi, Kenya; 4 United Nations Office on Drugs and Crime, Nairobi, Kenya

## Abstract

**Introduction:**

Kenya modes of transmission study attributed 4% of all new HIV infections to injecting drug use. AFYA-PWID, a 4-year programme, funded through PEPFAR grant aims to reduce HIV morbidity and mortality among people who use drugs in Kenya.

**Methods:**

This programme is implemented via a four pronged approach: improving policies, strategies, guidelines and coordination; increasing access to comprehensive HIV prevention, care and support package for people who inject drugs; strengthening policy makers and community support and enhancing M&E Capacity. This programme's key focus is introduction and scale up of high impact, evidence-based interventions, specifically Medically Assisted Therapy (MAT) – alias opioid substitution therapy. Programme achievements in Mombasa County, since project started in mid-2014, include: functioning multi-sectorial technical working group, county-specific standard operating procedures for MAT in place, two MAT clinics established at public health facilities; and over 60 health workers and over 30 CSO staff trained, 20 policy makers, 80 law enforcement, 10 judiciary, 50 religious leaders and 10 media personnel sensitized.

**Results:**

From September to December 2015, a total 167 individuals had initiated MAT. All males who inject heroin, all heroin-dependent females regardless of injecting status and sexual partners of enrolled clients were eligible. In total, 26% MAT clients were females, 11% were aged ≤25, 24% were HIV-infected (18% males vs. 42% of females) and 28% were HCV infected (33% males vs. 16% females). Overall HBV prevalence was 2%. Hundred percent tested opiates positive at baseline urine toxicology: 30% dependent on heroin alone, two-thirds concurrently used cannabis and heroin, while 5% dependent on heroin, cannabis and benzodiazepine. Daily methadone maintenance doses ranged 36 to 140 mgs. After 3 months, random urine screening reported 50% opiates positivity. By end of 2015, 6% clients were lost to follow up, and 2.3% were died.

**Conclusions:**

This programme represents a major milestone for Mombasa County! Within less than 4 months of initiation, 167 highly marginalized and stigmatized clients were accessing long overdue MAT services. Despite limited psychosocial support and other intervention, there is high treatment retention rate – possibly due to optimal methadone dosing. However, there is urgent need for integrated service delivery and livelihood assistance for recovering MAT clients.

# Sub article: WEAC0404 Incarceration and people who inject drugs in Ukraine: modelling its role in HIV transmission and the impact of introducing OST in prisons

**Published:** 2016-07-22

**Authors:** J Stone, E Brooks-Pollock, FL Altice, L Azbel, P Smyrnov, NK Martin, P Vickerman

**Affiliations:** 1 School of Social and Community Medicine, University of Bristol, Bristol, United Kingdom; 2 Section of Infectious Diseases, Yale University School of Medicine, New Haven, CT, United States; 3 London School of Hygiene & Tropical Medicine, London, United Kingdom; 4 International HIV/AIDS Alliance, Kyiv, Ukraine; 5 Division of Global Public Health, University of California San Diego, San Diego, CA, United States

## Abstract

**Introduction:**

People who inject drugs (PWID) experience high incarceration rates, and current or previous history of incarceration is associated with increased HIV and Hepatitis C transmission and heightened injecting risks. We assess the contribution of incarceration to HIV transmission amongst PWID in Ukraine, and the impact of introducing opiate substitution therapy (OST) in prison.

**Methods:**

We developed a dynamic model of incarceration and HIV transmission amongst PWID, which was fit using a Bayesian framework to data from Ukraine. The model was calibrated to data on HIV prevalence amongst never and previously incarcerated PWID in 2013 (12.8 and 28.2%, respectively), and currently incarcerated PWID in 2011 (28.5%). Based on data on the frequency of syringe sharing, baseline projections assumed increased injecting risk amongst previously incarcerated PWID compared to never incarcerated community PWID (1.9–3.3 times greater in first 12 months after release and 1.4–2.0 times greater thereafter), but made no assumption about the level of risk amongst incarcerated PWID because of insufficient data. Sensitivity analyses considered less informative priors. We projected the 15-year: contribution of incarceration to cumulative HIV incidence among PWID and impact of introducing prison OST from 2015.

**Results:**

Despite uncertainty in the HIV transmission risk among currently incarcerated PWID, the model projected that 55% (95% credibility interval: 40–68%) of new HIV infections could be averted amongst PWID in Ukraine over the next 15 years, if incarceration had no effect on HIV transmission from 2015. This result was robust to less informative priors on the level of risk in previously and currently incarcerated PWID. Conversely, if prison OST was initiated in Ukraine, with 50% coverage of incarcerated PWID and OST maintained for 1 year after incarceration, the model suggests 20% (95% credibility interval: 15–25%) of HIV infections could be averted from 2015 to 2030.

**Conclusions:**

Incarceration and the increased transmission risk associated with previous incarceration are likely to be important contributors to HIV transmission amongst PWID in Ukraine. Interventions need to focus on reducing these risks, with OST in prison possibly being an important strategy to reach this aim.

# Sub article: WEAC0405 Modelling the potential impact of the incarceration on HIV incidence among people who inject drugs in Tijuana, Mexico

**Published:** 2016-07-22

**Authors:** A Borquez, D Abramovitz, L Beletsky, P Vickerman, P Gonzales-Zuñiga, G Rangel, M Hickman, SA Strathdee, N Martin

**Affiliations:** 1 Global Public Health, University of California San Diego, San Diego, CA, United States; 2 University of Bristol, Bristol, United Kingdom; 3 Comisión de Salud Fronteriza, Mexican Ministry of Health, Tijuana, Mexico

## Abstract

**Introduction:**

Incarceration rates are high among people who inject drugs (PWID) in Tijuana, Mexico with a higher HIV prevalence among those ever compared to never incarcerated. Using dynamic mathematical modelling, we estimate the contribution of incarceration to the HIV epidemic among PWID in Tijuana and the potential impact of reducing incarceration rates.

**Methods:**

Data on HIV prevalence and incidence by incarceration exposure were obtained from an ongoing cohort of PWID in Tijuana (“El Cuete IV” 2006–2015). HIV prevalence was 3.4 and 6.7% among male and female PWID, respectively. In total, 85 and 53% of male and female PWID, respectively reported previous incarceration. Relative risk of HIV infection among ever versus never incarcerated male and female PWID was 1.10 (95% CI: 0.26–4.73) and 3.24 (95% CI: 0.70–15.00), respectively. A deterministic mathematical model of HIV transmission among PWID was developed reproducing the differential HIV risk and incarceration patterns among PWID by sex. The model was embedded in a Bayesian statistical framework using a Markov Chain Monte Carlo (MCMC) algorithm to estimate uncertainty in the outputs. Epidemic fits were resampled from the posterior distribution, and the proportion of new infections attributable to incarceration was calculated over different time periods (1980–2016, 2016–2021 and 2016–2026).

**Results:**

The model estimated that from the start of the epidemic to date, 43.5% (95% CrI: 25.9–60.3%) of new infections were attributable to incarceration and without incarceration HIV prevalence could have been a relative 2.1 folds (95% CrI: 1.4–3.1) lower in 2016 (1.7% instead of 3.4%). In the absence of incarceration between 2016–2021 and 2016–2026, 7.7% (95% CrI: –8.8–22.8%) and 10.6% (95% CrI: –6.4–26.6%) of new infections would be averted.

**Conclusions:**

Preliminary modelling suggests that incarceration has contributed substantially to HIV incidence among PWID in Tijuana, and a reduction in incarceration could avert up to 10% of new infections in the next 10 years. In 2009, Mexico decriminalized the possession of certain drugs for personal consumption in an effort to reduce incarceration rates among users; however, the reform has not been enforced in Tijuana. Further delaying its enforcement undermines the efforts to control the epidemic among this population.

# Sub article: WEAD0101 Sustained effect of couples' HIV counselling and testing on reducing unprotected sex among HIV serodiscordant couples

**Published:** 2016-07-22

**Authors:** K Wall, W Kilembe, B Vwalika, L Haddad, N Htee Khu, I Brill, C Vwalika, E Chomba, A Tichacek, S Allen

**Affiliations:** 1 Epidemiology, Emory University, Atlanta, GA, United States; 2 Emory University Rwanda Zambia HIV Research Group, Lusaka, Zambia; 3 Emory University Rwanda Zambia HIV Research Group, Atlanta, GA, United States

## Abstract

**Introduction:**

Couples’ voluntary HIV counselling and testing (CVCT) has been shown to significantly reduce HIV/STI incidence in HIV discordant couples by increasing condom use. The long-term impact of CVCT on sustained behaviour change has not been published.

**Methods:**

From 1994 to 2012, heterosexual HIV discordant couples (M+F- and M-F+) were recruited in Lusaka, Zambia, into long-term follow-up. Baseline and time-varying covariates were measured every 3 months. The outcome was a time-varying composite measure of self-reported unprotected sex, sperm presence on a vaginal swab wet prep, incident pregnancy and incident HIV seroconversion. Multivariable, repeated outcomes survival analysis (Anderson-Gill) explored factors predictive of unprotected sex.

**Results:**

Among 3049 couples followed, an average of 2 years/couple, incidence of unprotected sex indicators decreased significantly after the first CVCT visit (p<0.001), and this decrease was sustained over follow-up (p-trend<0.05). Predictors of unprotected sex are shown in Table 1. Model findings were similar when also controlling for fertility intentions.

**Conclusions:**

In HIV discordant couples, reductions in unprotected sex after CVCT are significant and sustained over long-term follow-up. We recommend broad CVCT scale-up per WHO guidelines. Reinforced condom counselling may be needed in M+F- couples (especially oral and injectable users, female alcohol users and during pregnancy) and M-F+ couples (especially during pregnancy). The finding that oral and injectable method use was predictive of unprotected sex in M+F- couples potentially explains published associations between hormonal contraception and HIV seroconversion (uncontrolled confounding by unprotected sex). The finding that male circumcision in M+F- couples was associated with unprotected sex warrants further investigation.

**Abstract WEAD0101–Table 1:** Multivariable models of predictors of unprotected sex among HIV discordant couples

	M+F- couples (N=1393)			M-F+ couples (N=1656)				
				
Contraceptive method (vs. condoms alone)^a^	HR^b^	95% CI	*p*	HR^c^	95% CI	*p*		
OCPs	1.34	1.19	1.50	<0.0001				
Injectables	1.41	1.23	1.61	<0.0001				
**Pregnancy status (vs. not pregnant)^a^**								
Pregnant (not incident)	1.88	1.74	2.03	<0.0001	1.60	1.50	1.71	<0.0001
Post-partum (≤6 months)	0.90	0.76	1.08	0.260	0.86	0.73	1.02	0.081
**Woman alcohol use in the past yr (yes vs. no)**	1.15	1.01	1.30	0.041				
**Circumcised male partner (yes vs. no)**	1.23	1.04	1.47	0.019				

OCP: oral contraceptive pill; IUD: copper intrauterine device; HR: adjusted hazard ratio; CI: confidence interval; yr: year

a time-varying variables; p-values are two-tailed

b controlling for age, self-reported protected sex with the study partner, self-reported outside sex, and follow-up time since enrolment

c controlling for age, self-reported protected sex with the study partner, and follow-up time since enrolment

# Sub article: WEAD0102 Positive impact of a randomized controlled trial of the Uthando Lwethu (“Our Love”) intervention on rates of couples HIV testing in rural South Africa

**Published:** 2016-07-22

**Authors:** L Darbes, NM McGrath, MO Johnson, V Hosegood, K Fritz, T Ngubane, H van Rooyen

**Affiliations:** 1 School of Nursing, University of Michigan, Ann Arbor, MI, United States; 2 Center for AIDS Prevention Studies, University of California, San Francisco, CA, United States; 3 Faculty of Medicine, University of Southampton, Southampton, United Kingdom; 4 Africa Centre for Population Health, Mtubatuba, South Africa; 5 Social Sciences, University of Southampton, Southampton, United Kingdom; 6 International Center for Research on Women, Washington DC, United States; 7 Human Sciences Research Council, Sweetwaters, South Africa

## Abstract

**Introduction:**

Couples-based HIV testing and counselling (CHTC) is an effective strategy for reducing sexual transmission between partners. However, uptake of the service has been low. We tested the efficacy of a couples-based intervention to increase participation in CHTC in a high HIV-prevalence setting.

**Methods:**

We randomized 332 couples (664 individuals) from a rural community in KwaZulu-Natal South Africa for an RCT of a couples-based behavioural intervention comprising six sessions (two group sessions/four couple counselling sessions) (n=168 couples) or one group session (n=164 couples). The intervention explored barriers to HIV testing and promoted improved communication skills and positive relationship dynamics. The primary outcomes were participation in CHTC and the reporting of the number of unprotected sex acts in the past 90 days with primary partner. Couples were ineligible if they had mutually disclosed their HIV status or previously participated in CHTC.

**Results:**

Twenty-two couples (6%) were lost-to-follow-up before 9 months, with no difference by group, p=0.36. Using intent-to-treat analysis, at the final 9-month follow-up, a higher proportion of intervention couples had participated in CHTC than control couples (42 and 12%, respectively; p≤0.001), with a shorter time to CHTC than control group couples who participated in CHTC (Logrank p≤0.0001). For sexual behaviour, there was a significant reduction in the proportion of unprotected sex acts for intervention couples at 3-month follow-up (IRR=0.74, p≤0.022), but a negative binomial regression model accounting for couple clustering found no significant group-by-time interaction (p=0.08).

**Conclusions:**

To our knowledge, this is the first intervention that targeted increasing participation in CHTC. Results suggest that addressing relationship factors among African heterosexual couples can significantly improve rates of CHTC. The intervention had an impact on proportion of unprotected sex acts at first follow-up but this was not sustained over time. Our intervention reached a high number of couples that were unaware of their joint HIV status at baseline. Further, results show that it is possible to promote engagement in CHTC – which is an effective strategy that accomplishes HIV testing, mutual disclosure and can facilitate entrée into treatment for HIV-positive individuals in high prevalence settings.

# Sub article: WEAD0103 Assessment of couple relationship quality and links to HIV prevention, treatment and care in rural Malawi

**Published:** 2016-07-22

**Authors:** A Ruark, D Brewster-Lee, J Hembling, V Rhoe Davis

**Affiliations:** 1 Medicine, Brown University, Providence, RI, United States; 2 Catholic Relief Services, Baltimore, MD, United States

## Abstract

**Introduction:**

Couple relationship quality may impact partner-level behaviours related to HIV risk, including couples’ HIV testing, serostatus disclosure and care outcomes. The Couple Functionality Assessment Tool (CFAT) was developed to allow programmes to assess couple relationship quality in low-resource settings.

**Methods:**

The CFAT was pilot tested among 203 women and 198 men (married and cohabiting) in rural Malawi in August 2015. Factor analysis reduced the CFAT to 31 questions addressing five domains of relationship quality (intimacy, partner support, sexual satisfaction, decision making, communication and conflict management), plus questions on intimate partner violence and partner support for seeking HIV care. Regression analysis examined the relationship of the refined CFAT to key HIV-related behaviours.

**Results:**

Most participants reported that they and their partners had been tested for HIV and mutually disclosed their status (90% of women, 85% of men). Women with the highest relationship quality scores were significantly more likely than women with the lowest scores to report that they and their partners had been tested for HIV and mutually disclosed results (94% vs. 72%, p<0.01) and also to report other behaviours critical to HIV care and treatment adherence, such as deciding with partner how to manage household budget (p<0.001) and having a joint financial plan (p<0.001). Women with low relationship quality were also significantly more likely to report intimate partner violence and abuse. Men reported that they would be more supportive of women seeking PMTCT and of children's HIV testing than women perceived them to be (differences between the perceptions of men and women, significant at p<0.001 and p=0.002, respectively).

**Conclusions:**

The CFAT showed validity in this population, and findings suggest that strengthening the quality of couple relationship and giving couples tools to build good communication may support behaviours critical to HIV outcomes. Women may be underestimating men's support for PMTCT services and paediatric testing, which could create barriers to care. The greater violence and abuse experienced by women in low-quality relationships may also create HIV risk and impede care. The CFAT will enable projects aiming to improve HIV outcomes by enhancing couple functionality to measure relationship quality validly and reliably.

# Sub article: WEAD0104 Partner communication and support around HIV and how this relates to health-seeking behaviour: a qualitative study amongst HIV-positive individuals and couples in Karonga, Malawi

**Published:** 2016-07-22

**Authors:** J Renju, A Wringe, AC Crampin, O Koole, C Nyirenda, H Namadingo, E McLean, ALPHA Network

**Affiliations:** 1 London School of Hygiene and Tropical Medicine, London, United Kingdom; 2 Malawi Epidemiology and Intervention Research Unit, Karonga, Malawi

## Abstract

**Introduction:**

HIV policies and practices in Malawi and elsewhere in sub-Saharan Africa have strongly focused on couple testing, counselling and partner disclosure to improve treatment adherence and outcomes. Little is known about how HIV-positive concordant couples respond to these initiatives and the consequences for their relationships and HIV care-seeking behaviours. In the context of a larger qualitative study on the experiences of care-seeking among HIV-positive adults, communication around HIV and its influence on care-seeking behaviours was explored.

**Methods:**

In-depth interviews were carried out with 24 women and 17 men diagnosed with HIV, including eight couples who had mutually disclosed their status, purposefully sampled from ART clinics or households using the Karonga health and demographic surveillance system database. Participants were encouraged to explain their journey with HIV; topic guides explored communication with partners and other support networks, and experiences with HIV services. A framework analysis approach was used. Individual narratives from eight couples were compared with map communication within relationships to understand potential implications on health-seeking behaviours.

**Results:**

Communication about HIV testing, care and treatment in a relationship was primarily driven by a perceived need for support: some people disclosed their status in anticipation of specific support mechanisms, whereas others did not disclose for fear of being abandoned and losing any kind of support. Communication about HIV testing and treatment was often initiated by women and was often influenced by child-bearing and care. Despite knowing each other's HIV status, most partners were unable to accurately articulate their partner's HIV-related experiences suggesting communication was restricted to particular areas, implying an individual focus. Those that reported support generally defined it in practical rather than psychological terms (e.g. reminders to take drugs).

**Conclusions:**

Most participants reported that disclosing to their spouse was important, but following disclosure communications did not consistently extend to a meaningful understanding of the other's experience of living with HIV. Despite purportedly couple-friendly services, partners rarely attended the health facility together, suggesting HIV remains a solo journey. As policy moves towards universal ART, further consideration is required around how to engage partners in culturally appropriate ways to support improved communication and health-seeking behaviours.

# Sub article: WEAD0105 Intervention outcomes on mental health of PLH, family members and children: a randomized controlled trial in rural China

**Published:** 2016-07-22

**Authors:** L Li, L-J Liang, CQ Lin, Y Xiao, GP Ji

**Affiliations:** 1 Semel Institute, University of California, Los Angeles, CA, United States; 2 Anhui Provincial CDC, Hefei, China

## Abstract

**Introduction:**

HIV impacts families. This study examines the efficacy of an intervention that targets people living with HIV (PLH), their family members and children in rural China. The intervention outcomes on mental health were evaluated for all the three populations.

**Methods:**

The intervention trial utilized a two-arm design with 475 families impacted by HIV in rural Anhui, China, including 522 PLH, 475 sero-negative family members and 536 children aged 6–18 years. Previously piloted TEA (Together for Empowerment Activities) intervention was delivered at three levels: 1) TEA Gathering (small group for PLH and family members); 2) TEA Time (home-based family activities with children); and 3) TEA Garden (community events). Intervention effect was evaluated at baseline, 6-, 12-, 18- and 24-month follow-ups. Mixed-effects regression models were used to assess the improvement on the mental health measures – for PLH on depressive symptoms and coping with illness, for family members on depressive symptoms and caregiver burden and for children on self-esteem and daily stress. Estimated difference and standard error (SE) in changes from baseline between intervention and control from the regressions are shown.

**Results:**

For PLH, we found significant intervention effects on improved levels of coping with illness at the 6-month (4.45±0.84; P<0.0001), 12-month (3.19±0.85; P=0.0002), 18-month (3.09±0.85; P=0.0003) and 24-month follow-up (2.55±0.87; P=0.0034). Similarly, significant effect on reduction of depressive symptoms was observed at each of the follow-ups for PLH. For family members, significant intervention effects at the follow-ups were found on improved depressive symptoms but not on caregiver burden. For children, although intervention effects on the improved self-esteem were not significant between intervention and control, significant intervention effects on levels of daily stress were found at the 6-month (1.49±0.72; P=0.0386) and 12-month (1.68±0.74; P=0.0241) follow-ups.

**Conclusions:**

This is our first longitudinal outcome report based on the large-scale, randomized trial. Study findings support the feasibility in implementation and efficacy of the multilevel TEA intervention not only for PLH but also for family members and children. Intervention activities that connect various members in a family could be the key to link to the intervention outcomes.

# Sub article: WEAD0201 Meeting the reproductive intentions of PLHIV in Malawi

**Published:** 2016-07-22

**Authors:** L Irani, E McGinn, M Mellish, O Mtema, P Dindi

**Affiliations:** 1 UNICEF, Gender Section, New York, NY, United States; 2 Palladium, Washington, DC, United States; 3 Health Policy Plus Project, Palladium, Washington, DC, United States; 4 Health Policy Plus Project, Palladium, Lilongwe, Malawi

## Abstract

**Introduction:**

Malawi's HIV clinical management guidelines recommend provider-initiated family planning (PIFP) counselling and provision of condoms and injectables within ART settings. The USAID- and PEPFAR-funded Health Policy Project's 2015 study assessed how the reproductive rights of people living with HIV (PLHIV) are being addressed through the integration of Family Planning (FP) into ART services.

**Methods:**

Data were collected from a purposive sample of 41 public and private facilities across nine districts of Malawi. Facilities ranged from large high-volume hospitals to small health posts. Data collectors conducted 41 facility audits, 41 interviews with facility in-charges, 122 interviews with providers, 425 client exit interviews, 58 mystery client interviews and 3 focus group discussions with PLHIV (n=33).

**Results:**

Over half (52%) of female clients (n=315) reported not wanting another child. The majority of female clients (60%) were using contraception; half relied on condoms, whereas one-third were using injectables. Almost one-half (47%) reported not being told about side effects with their current method; 26% reported they were not told about other FP methods. Only 14% of clients reported receiving PIFP at that day's visit. Few clients (18%) reported receiving multiple services that day; however, 97% said they would prefer to receive fully integrated services. Clients identified fewer trips to the facility (78%) and reduced transportation costs (43%) as clear benefits of integrated services. Mystery client visits revealed extremely low levels of PIFP implementation (n=2), and also documented cases of harsh treatment (n=11) and instances where clients were denied services (ART=5, FP=11) because they were not registered at that facility. Fewer than half of the mystery clients reported a satisfactory experience. Some focus group discussion (FGD) participants recounted experiences of mistreatment from service providers.

**Conclusions:**

ART clients in Malawi have a high demand and need for effective FP services and express a preference for integrated services. Despite national guidelines on PIFP, few providers are initiating discussions on reproductive intentions with ART clients. Many HIV clients are relying on condoms to meet their reproductive intentions, and a large number are not receiving quality counselling on a range of methods. An unanticipated finding was the degree to which providers may be mistreating clients, which warrants further study.

# Sub article: WEAD0202 Sexual and reproductive health needs and experiences of youth living with HIV

**Published:** 2016-07-22

**Authors:** R Imakit, S Ajok, D Talima, A Namakula

**Affiliations:** 1 Research and Evaluation, Straight Talk Foundation, Kampala, Uganda; 2 Straight Talk Foundation, Kampala, Uganda

## Abstract

**Introduction:**

The 1994 International Conference on population and development marked the start of the rights-based approach to sexual and reproductive health (SRH), with focus on individuals and their needs, aspirations and rights. SRH needs of youth living with HIV (YLHIV) are often overlooked; meeting them is fundamental to SRH rights of YLHIV and to addressing the global HIV pandemic. Despite its significance, SRH of YLHIV is an often neglected area of research and programming and represents a priority on 2015 post-MDG agenda. Straight Talk Foundation aimed to investigate the dynamics underpinning sexual and reproductive health for YLHIV in Uganda.

**Methods:**

A cross-sectional and qualitative study design was adopted. The study was carried out among YLHIV who lived in either an urban setting (Kampala) or a rural or post war (Gulu) district. A sequential exploratory approach was used in data collection. Participants were systematically picked from a sample frame determined within their peer network. Thirty-nine semi-structured interviews with YLHIV and seven key informants with counsellors and medical staffs were carried out. Voice recorders were used to capture data, thus data were transcribed and exported to Nvivo version 10 for data analysis. Consent was sought from the youth.

**Results:**

YLHIV were sexually active, or in relationships with intentions of sexual activity, and with sero-discordant partners. Health facilities where YLHIV accessed ARVs from had no SRH services integrated. The sexual encounters of YLHIV were typically unplanned making negotiation of safe sex, such as disclosure and use of contraceptives challenging and inconsistent. YLHIV reported experiencing a lot of public HIV-related stigma and discrimination leading to social isolation, which reduces social support networks and led to poor self-esteem and consequently poorer motivation for self-protection during sex. Many of the YLHIV lacked SRH information for decision making thus fuelling myths and misconceptions which YLHIV commonly act upon; faced cultural taboos and the association of sexuality with immorality inhibiting discussion of sex, relationships and contraception between YLHIV and their parents.

**Conclusions:**

Social vulnerability of YLHIV to SRH threats is complex and multifaceted. In order to improve SRH for YLHIV, a holistic approach which addresses the broader social environment is required.

# Sub article: WEAD0203 “I always wanted a big family because I lost mine”: a qualitative analysis of parenting perspectives among young parents with perinatally acquired HIV

**Published:** 2016-07-22

**Authors:** H Allen, C Fair, C Trexler, L D'Angelo

**Affiliations:** 1 Elon University, Elon, NC, United States; 2 Human Service Studies and Public Health Studies, Elon University, Elon, NC, United States; 3 Adolescent Clinical Research, Children's National Medical Center, Washington, DC, United States; 4 Division of Adolescent and Young Adult Medicine, Children's National Medical Center, Washington, DC, United States

## Abstract

**Introduction:**

Globally, children with perinatally acquired HIV (PHIV) are now living into young adulthood and having children of their own. Little is known about the parenting perspectives of youth who may have experienced family disruption due to loss/illness of biological parents. This research explores the perceptions of adolescents and young adults (AYA) living with PHIV as they transition into parenthood.

**Methods:**

We conducted hour-long, semi-structured, audio-recorded interviews with a purposive sample of 16 AYA parents with PHIV who were current or former patients at two US paediatric/adolescent infectious diseases clinics. Participants were asked about their childhood family structure, rewards/challenges of parenting and anticipated future fertility desires/intentions. Analysis of the transcribed interviews was guided by grounded theory identifying key common themes across the interviews.

**Results:**

Mean age of participants was 22 years. The majority were black (7) or Hispanic (4) and female (14). Four AYA were raised by biological mothers, five by foster/adoptive parents and the others by relatives. Participants had a range of 1–3 children (mean=1.4), one of whom was HIV-positive. Participants expressed many normative parenting rewards and challenges such as the joy of their child's smile and financial concerns. Unique themes associated with HIV infection included a concern about not “being there” for their child due to sickness and worries that their child may experience HIV-related discrimination. Among those parents who intended to have another child, many were motivated by a strong desire to create a family of their own as a way to deal with HIV-related losses experienced in childhood. Finally, participants also noted the positive role played by paediatric and adolescent medical providers, even if they had transitioned to adult care. Participants reported the importance of emotional support offered by providers as well as concrete social services available in that care setting.

**Conclusions:**

AYA with PHIV who have children experience many of the same issues as other young parents. However, they also have HIV-specific experiences that influence their parenting such as illness, discrimination and childhood parental loss that may intensify their fertility desires. The positive impact providers have throughout a youth's childhood must be recognized and capitalized upon.

# Sub article: WEAD0204 Biographies of HIV and cervical cancer: understanding treatment-seeking for cervical cancer amongst HIV-positive women in Inner City Johannesburg

**Published:** 2016-07-22

**Authors:** J Stadler, A Chikandiwa, P Mayaud, H Rees, J Imrie, S Delany-Moretlwe

**Affiliations:** 1 Reproductive Health and HIV Institute, University of the Witwatersrand, Johannesburg, South Africa; 2 London School of Hygiene and Tropical Medicine, London, United Kingdom

## Abstract

**Introduction:**

Cervical cancer is preventable, yet in South Africa it is the leading cause of cancer mortality, particularly amongst women living with HIV/AIDS (WLHA). Low screening rates and poor uptake of treatment for CIN2 are contributing factors. We explored the challenges facing WLHA diagnosed with CIN2 in the “HPV for Africa Research Partnership” (HARP) study that screened, counselled and referred WLHA. We investigated why over a quarter of women in the study who needed surgical treatment did not access it.

**Methods:**

A purposive sub-sample (n=30) was selected for in-depth interviews (IDI), of which 15 had received surgical treatment for CIN2 and 15 had not. Of these, five of each were invited for a second interview. The McGill Illness Narrative Interview tool (www.mcgill.ca) was used to elicit:

1. a chronology of symptoms and illness experiences;
2. popular representations of illness; and
3. explanatory models of illness and treatment.

Recorded IDI at the study clinic, conducted in local languages, were transcribed and translated and coded in Nvivo 10 according to emergent themes.

**Results:**

Twenty study participants (16 treated and 4 untreated, including 12 taking ART) attended one IDI and nine participated in a second IDI. Participant′s illness narratives reflected shared experiences of intimate partner violence (IPV), domestic instability and material deprivation. These experiences shaped a collective perception of cancer as untreatable and hopeless that threatens productive and reproductive futures. In contrast, HIV was well understood and manageable. Cash availability and supportive household relationships facilitated women's treatment seeking for HIV and CIN2. However, all women in the study experienced challenges in accessing treatment.

**Conclusions:**

The biographies of WLHA diagnosed with CIN2 reveal structural and interpersonal violence that shaped individual experiences and perceptions of illness, helping us to understand their fatalistic outlooks and the delays and failures in seeking treatment. Health services need to not only address women's perceptions of cancer but also remove barriers to immediate treatment. Screen and treat options may be an important intervention for this population and a HPV vaccine for WLHA is a promising option to prevent cervical cancer.

# Sub article: WEAD0205 Findings from the Sexual Health and Ageing Programme (SHAPE) for older women with HIV: pilot study and future directions

**Published:** 2016-07-22

**Authors:** T Taylor

**Affiliations:** Medicine/Infectious Disease, SUNY Downstate Medical Center, Brooklyn, NY, United States

## Abstract

**Introduction:**

There are few sexual risk reduction interventions that prioritize the unique needs of older women living with HIV (OWLH). The lack of proven interventions is particularly problematic in light of documented risk behaviours in this population, including condomless sex (CS), often with serodiscordant partners. To address the dearth of work on HIV prevention for OWLH, we conducted research to develop the Sexual Health and Aging Programme (SHAPE).

**Description:**

SHAPE is a theoretically derived, gender- and generationally tailored, peer-delivered, small-group, skills-based intervention designed to reduce participants’ stress related to HIV disclosure and maintaining safer sexual behaviours and promoting successful ageing with HIV which incorporates HIV transmission prevention methods for OWLH (Treatment as Prevention) and their partners (PrEP). We pilot tested SHAPE with 58 OWLH, aged 45 years and older who reported CS in the prior 3 months to assess the feasibility, safety and acceptability of study procedures and evaluation process. We conducted 58 baseline ACASI surveys, 49 (84%) and 48 (83%) 3- and 6-month follow-up assessments, respectively, and we conducted seven 2-day SHAPE programmes with 33 women and seven booster sessions; 25 women also received a standard of care programme.

**Lessons learned:**

Participants found SHAPE to be highly acceptable and comfortable. Due to ceiling effects and the small size, the intervention had no effect on reducing CS or improving coping, HIV disclosure and safer sex self-efficacy compared with standard of care. Almost 80% of the participants were virally suppressed and had extremely high baseline coping self-efficacy (mean scores: 199–203).

**Conclusions/Next steps:**

We believe that SHAPE's impact would be greatest if it was targeted at the most vulnerable OWLH – those with inconsistent viral suppression, concomitant psychosocial factors and partner-related barriers. We propose to further refine and develop SHAPE as an adaptive intervention strategy to improve its ability to impact viral suppression and self-care management needed to foster *Healthy HIV Ageing* and strengthen its impact on transmission risk by developing new adaptive partner disclosure and couples-support intervention components for those OWLH who report condomless sex with serodiscordant partners.

# Sub article: WEAD0301 Measuring the impact of advocacy: civil society's influence over Global Fund concept notes in eight African countries

**Published:** 2016-07-22

**Authors:** G Oberth, O Mumba, L Bhayani

**Affiliations:** 1 Centre for Social Science Research, University of Cape Town, Cape Town, South Africa; 2 Eastern Africa National Networks of AIDS Service Organizations (EANNASO), Arusha, United Republic of Tanzania; 3 International HIV/AIDS Alliance, Lusaka, Zambia

## Abstract

**Introduction:**

One fifth of Global Fund grants are implemented by civil society organizations. However, the degree to which non-state actors are able to shape the content of those grants through the initial concept note is uncertain and hard to measure. As a result, it is not always clear if the Global Fund is investing appropriately in communities.

**Methods:**

Global Fund concept notes from Kenya, Malawi, Swaziland, Tanzania, Uganda, Zanzibar, Zambia and Zimbabwe were systematically measured to assess the inclusion of civil society priorities. National Civil Society Priorities Charters were used as indicators for civil society priorities. Each priority in the country's Charter was assessed for its inclusion in the Global Fund concept using a three-point scale (2=included, 1=partially included and 0=not included).

**Results:**

The percentage of civil society priorities that were included in Global Fund concept notes were as follows: Malawi (87%), Kenya (76%), Tanzania (67%), Zanzibar (67%), Uganda (64%), Swaziland (50%), Zimbabwe (40%) and Zambia (38%). Across the eight countries, civil society priorities on key populations were the most likely to get included in the concept notes (68%), while priorities on voluntary medical male circumcision were the least likely to get included (15%). Several contextual factors help explain these results. Using Afrobarometer survey data, civil society had greater influence over Global Fund concept notes in countries where people often attend community meetings (CI 95%, p=0.041), often join others to raise an issue (CI 95%, p=0.017) and feel completely free to say what they think (CI 95%, p=0.030). Using World Bank Governance Indicators, civil society had greater influence over Global Fund concept notes in countries where there is a greater degree of freedom of association and freedom of expression (CI 90%, p=0.083). In countries where civil society was more effective at influencing Global Fund concept notes, HIV prevalence was lower (CI 95%, p=0.021).

**Conclusions:**

This is some of the only statistical evidence to demonstrate that open and inclusive dialogue spaces are linked to a more effective civil society in the HIV response. An empowered civil society is vital, as the inclusion of their priorities is related to lower HIV prevalence.

# Sub article: WEAD0302 Global solidarity to win increased accountability and impact from PEPFAR country programs: an analysis of north-south collaborative advocacy strategies

**Published:** 2016-07-22

**Authors:** A Russell, F Mwanza, M Milanga

**Affiliations:** 1 Health Global Access Project (Health GAP), on behalf of the PEPFAR Watch Network, Kampala, Uganda; 2 Treatment Advocacy and Literacy Campaign, Lusaka, Zambia; 3 Health Global Access Project (Health GAP), Nairobi, Kenya

## Abstract

**Introduction:**

The U.S.-funded President's Emergency Plan for AIDS Relief (PEPFAR) is the largest funder of the HIV response in the hardest hit countries in the world. Holding PEPFAR accountable through civil society advocacy is a pre requisite to ensure that PEPFAR resources deliver effective, high impact prevention and treatment services for communities. After civil society criticism, in 2013 PEPFAR announced a commitment to support civil society engagement in the annual development of PEPFAR's Country Operational Plans (COPs)—the documents describing PEPFAR's budgets, targets and strategies for each country. However in most countries in 2014, civil society (and particularly key populations) engagement in shaping the PEPFAR COPs remained limited. The PEPFAR Watch Network emerged to increase community engagement in the COPs process, and comprises US-based organizations and civil society in PEPFAR-funded countries, advocating to shape PEPFAR funding and priorities based on the priority unmet need of people living with HIV and their communities.

**Description:**

We examine the impact of a global advocacy network of allies in high burden countries and in the US in challenging PEPFAR to increase meaningful engagement of CSOs, transparency and accountability to communities from 2014 – 2016.

**Lessons learned:**

Health GAP and other partners in the PEPFAR Watch Network learned that applying concerted and coordinated pressure both in Washington DC and in key recipient countries to amplify demands regarding PEPFAR service delivery is an effective strategy. The unique North-South partnership allowed advocates to successfully challenge decision makers in both Washington DC, and within PEPFAR-funded countries.

**Conclusions/Next steps:**

PEPFAR's stated commitment could have a substantial impact on the drive to end the AIDS epidemic, using civil society advocacy to bring PEFPAR’s priorities into alignment with the demands and priorities of people with HIV. PEPFAR investments are an area of untapped potential, which can be made more effective in the global AIDS response through the involvement of community advocates and key populations groups. High-impact watchdogging, monitoring and accountability by a North-South coalition of civil society partners can leverage new opportunities to engage with the PEPAR COPs process to ensure that critical HIV prevention and treatment services are in line with community needs.

# Sub article: WEAD0303 Demanding a high impact HIV response: civil society advocacy and the President's Emergency Plan for Aids Relief (PEPFAR) in Uganda

**Published:** 2016-07-22

**Authors:** R Nandelenga, D Namutamba

**Affiliations:** 1 International Community of Women Living with HIV/AIDS (ICW), Kampla, Uganda; 2 Programmes, International Community of Women Living with HIV/AIDS (ICW), Kampala, Uganda

## Abstract

**Introduction:**

The PEPFAR programme provides treatment, prevention and care for people living with or affected by HIV/AIDS in high burden countries including Uganda where it is the largest donor. Engagement of civil society in the development and implementation of PEPFAR's plans is therefore critical to ensuring that PEPFAR's priorities reflect real lived experience and that the plans emphasize the priorities of communities most affected. Uganda's faltering response to HIV makes it crucial to ensure funding is invested in high impact interventions.

**Description:**

In Uganda, the International Community of Women Living with HIV Eastern Africa (ICWEA) coordinates and convenes CSOs in a coalition focused on analysis and advocacy on PEPFAR and other major actors the AIDS response. This coalition has engaged in a series of high impact advocacy efforts, including development of civil society monitoring tools, training and empowerment of women living with HIV so that they are able to engage the implementing partners (IPs) and PEPFAR teams directly and demand programs that address the needs of the communities. Results of field assessments and other data are shared with the PEPFAR country teams to inform planning and programming and correct mistakes in real time. The coalition also links with advocates in the US regarding priority advocacy and policy matters.

**Lessons learned:**

CSOs and the PEPFAR country team have an engagement roadmap in line with the COP planning cycle and written information and feedback is shared regularly. CSOs provide formal recommendations to the in-country COP development and implementation process and to PEPFAR headquarters and understand better PEPFAR COP programming. Quarterly field assessments have enabled CSOs to provide feedback informed by evidence to the PEPFAR country teams and push for relevant corrective measures. The process is empowering and creates a sense of ownership to people with HIV and their communities, especially women and young women living with HIV who gather this data.

**Conclusions/Next steps:**

ICWEA with national and global partners has developed a PEPFAR COP engagement strategy, complementing Global Fund, to generate strong and effective advocacy in order to improve accountability of donors and address bottlenecks obstructing efforts to end AIDS in Uganda.

# Sub article: WEAD0304 PLHIV in the Caribbean: many islands, same issues. Lack of resources, fragmented health/care systems: an under-resourced community response

**Published:** 2016-07-22

**Authors:** C Albert-Hope, R Gustav, Y Simon, E Castellanos, R Irwin

**Affiliations:** 1 Caribbean Regional Network of People Living with HIV and AIDS (CRN+), Port of Spain, Trinidad and Tobago; 2 GNP+, Amsterdam, Netherlands; 3 CNET+, Belmopan, Belize; 4 GNP, Amsterdam, Netherlands

## Abstract

**Introduction:**

2014 figures: 280,000 (210,000–340,000) PLHIV in the Caribbean. However, treatment coverage is only 44% (33–54%) of people 15 years or older, 36% (32–42%) among children. Caribbean countries continue to face *economic and fiscal challenges*. Global economic crises and decline or withdrawal of donor resources impact adversely on the response; nowhere is this more acutely played out than in funding available to community-based PLHIV and allied organizations – who are the first point of call for people facing HIV *related stigma and discrimination*. This is still a major barrier for accessing HIV and other health services – especially considering the very small population of many islands and Caribbean territories. This is compounded by punitive laws (e.g. the criminalization of key populations), policies and practices that foster significant human rights violations, promote fear and discourages many PLHIV from disclosing their status.

**Description:**

In 2015, The Caribbean Regional Network of People Living with HIV and AIDS (CRN+) as part of the Positive Networks Consortium (led by GNP+) with the support of RCNF (Robert Carr Network Fund) conducted an advocacy strategy assessment amongst 10 countries (and one Caribbean municipality of the Netherlands Seba). A total of 225 people across the 11 sites (with over half PLHIV, and others community members from key populations and community workers) were interviewed about the work they did in relation to HIV, how funded, and priorities for advocacy and the response.

**Lessons learned:**

Whether from St Lucia (pop. 185 K) to Haiti (pop. 10.5 m) common themes ran through the results; included were the need for universal access to medications (ARVs) without stock outs, sub-optimal regimes in place, services not being delivered free from stigma and discrimination. The evidence illustrated that the input of community organizations to the response was not properly valued, and feelings they lacked the advocacy tools and strategies to change this.

**Conclusions/Next steps:**

The results influenced the CRN+ strategic planning process and advocacy agenda. Additionally, it has influenced the Positive Networks Consortium advocacy to support an enhanced community role in CCM's ' and other fora, as well as strategies to challenge punitive laws and practices toward key populations.

# Sub article: WEAD0305 Rapid response research to inform HIV policy decision-making: lessons learned from California's Collaborative HIV/AIDS Policy Research Centers

**Published:** 2016-07-22

**Authors:** IW Holloway, WT Steward, J Mortimer, P Curtis, D Van Gorder, A Leibowitz, S Morin, C Mulhern-Pearson, A Donnelly, AJ King, A Fox, C Pulsipher, D Evans, G Lemp

**Affiliations:** 1 Luskin School of Public Affairs, University of California, Los Angeles (UCLA), Los Angeles, CA, United States; 2 Center for AIDS Prevention Studies, University of California, Los Angeles, San Francisco, CA, United States; 3 California HIV/AIDS Research Program, University of California, Office of the President, Oakland, CA, United States; 4 AIDS Project Los Angeles, Los Angeles, CA, United States; 5 Project Inform, San Francisco, CA, United States; 6 San Francisco AIDS Foundation, San Francisco, CA, United States; 7 Los Angeles LGBT Center, Los Angeles, CA, United States

## Abstract

**Introduction:**

Responding to the HIV epidemic requires policy decisions that are well researched and informed by empirical evidence. The policy environment, however, is dynamic and fast-paced, and the opportunity to effect change may be limited to brief periods of time. To ensure research findings are ready within these “policy windows,” researchers must be able to launch and complete projects quickly. Responding to these realities, the California HIV/AIDS Research Program (CHRP) has, since 2009, funded two collaborative HIV/AIDS Policy Research Centers. Each consists of university and community-based agency partners that work statewide with consumers, advocates, and policymakers to conduct “rapid response” short-term projects designed to address questions that emerge in the dynamic health policy environment.

**Description:**

Policy research advisory committees meet annually in northern and southern California to prioritize HIV policy-related questions and concerns that would benefit from research. Following each meeting, policy centre investigators formulate specific research questions and study designs based on policy research advisory committee priorities. Data for each rapid response project are then collected, analyzed, and disseminated back to policy stakeholders, ideally in 6 months or less.

**Lessons learned:**

The HIV/AIDS Policy Research Centers have successfully addressed critical policy issues that emerged in California over the past 7 years. These include analyses of: state budget cuts to HIV prevention; enhanced surveillance efforts on federal funding for California; mandating condom distribution in correctional facilities; the impact of the state's Affordable Care Act implementation on HIV providers and patients; the effects of healthcare reform efforts on the care of HIV-positive individuals who also have mental health diagnoses; the impact of limiting physician visits, capping prescriptions, and charging co-pays for HIV medications; and examining various HIV workforce issues, such as the aging and specialty mix of physicians who provide HIV treatment in California.

**Conclusions/Next steps:**

The collaboration between academic and community partners through standing policy research centres has brought together synergistic skill-sets, knowledge bases, and professional relationships to successfully inform robust and timely analyses of HIV-relevant policy issues. Expansion of this funding model would help to ensure that research is able to respond to the rapid changes in policy environments.

# Sub article: WEAD0306LB The uptake of population size estimation studies for key populations in guiding HIV responses across sub-Saharan Africa: a systematic review

**Published:** 2016-07-22

**Authors:** S Baral, C Lyons, E Sullivan, S Kurani, J Sherwood, G Millett, J MacAllister

**Affiliations:** 1 Department of Epidemiology, Johns Hopkins Bloomberg School of Public Health, Baltimore, MD, United States; 2 The Foundation for AIDS Research (amfAR), Washington, DC, United States

## Abstract

**Introduction:**

There has been an increase in the focus on data to better inform the HIV response. This has included data focused on both defining the content of HIV programmes as well as the scale of these programmes in response to evidence-based need. To this end, population size estimation (PSE) studies for key populations have become increasingly common to define the necessary scale of specific programmes for these populations. This study aims to systematically assess the uptake of PSE in HIV policy and programme documents across the continent of Africa including sub-Saharan and North Africa to assess the ultimate utility of these studies.

**Methods:**

This study included two phases; Phase 1 included a systematic review of all PSE for key population including men who have sex with men (MSM), female sex workers (FSW), and people who inject drugs (PWID) across sub-Saharan Africa (SSA) from January 2009 to February 2016 using the Preferred Reporting Items for Systematic-Reviews and Meta-Analyses (PRISMA) guidelines. Phase 2 represented a review of 23 different types of documents used to inform HIV programming in countries with a focus on PEPFAR and the Global Fund (GF) investments.

**Results:**

A total of 71 PSE were identified; two of which were mentioned in GF Concept Notes, 12 in PEPFAR Country Operational Plans, and seven in national Ministry of Health documents; and 15 included plans of action for the data (Table 1).

**Conclusions:**

While there is an increasing trend in the completion of PSE studies for key populations in more generalized HIV epidemic settings involving significant investments of finances and human resources, there is limited evidence of effective uptake of these data to guide the HIV responses in these countries. While PSE are important to guide data-driven HIV responses, the data presented here suggest an opportunity to build capacity to ensure that available data appropriately guides responses and optimal decisions are made about data needs moving forward.

**Abstract WEAD0306LB–Table 1:** Research utilization indicators

Year of estimation (if mentioned)	Population	Location	Stakeholders developed an Interpretation and Use Plan for PSE	PSE used to identify a problem	PSE used to develop a plan of action/ recommendation to address that problem	PSE used to change a Global Fund policy as documented in concept notes	PSE used to change a PEPFAR policy as documented in country operational plans	PSE used to change a national MOH policy as documented in NSPs	Study results published in a peer-reviewed academic journal	Results/data translated into non-academic resources (briefs/ pamphlets/ advocacy tools)
N/A	PWID	East Africa								
N/A	PWID	South Africa								
N/A	PWID	Nigeria								
2009	MSM-SW	Lagos, Nigeria								
2009	MSM-SW	Kano, Nigeria								
2009	MSM-SW	Port Harcourt, Nigeria								
2008, 2009	FSW	Mauritius								
2009, 2010	PWID	Mauritius								
2010	MSM	Morocco								
2010	PWID	Morocco								
2010	FSW	Morocco								
2010	PWID	Nairobi, Kenya								
2009–2010	FSW	Nairobi, Kenya								
2010	MSM	Nairobi, Kenya								
2012	PWID	Kenya								
2012	FSW	Kenya								
2012	MSM	Kenya								
2012	FSW	Lagos, Nigeria								
2012	FSW	Anambra, Nigeria								
2012	FSW	Nigeria								
2011	MSM	Luanda, Angola								
2011–2012	FSW	Nyanza, Kenya								
2011–2012	FSW	Coastal Kenya								
2011–2012	FSW	Eastern Kenya								
2011–2012	FSW	Central Kenya								
2011–2012	FSW	Nairobi, Kenya								
2011–2012	FSW	Kenya								
N/A	FSW	Niger								
2013	MSM	Yaoundé, Cameroon								
2013	MSM	Douala, Cameroon								
2013	MSM	Bamenda, Cameroon								
2013	MSM	Bertoua, Cameroon								
2013	MSM	Bafoussam, Cameroon								
2013	MSM	Ngaoundere, Cameroon								
2013	MSM	Kribi, Cameroon								
2013	FSW	Douala, Cameroon								
2013	FSW	Bamenda, Cameroon								
2013	FSW	Bertoua, Cameroon								
2013	FSW	Bafoussam, Cameroon								
2013	FSW	Ngaoundere, Cameroon								
2013	FSW	Kribi, Cameroon								
2013	FSW	Yaounde’, Cameroon								
	MSM	Luanda Province, Angola								
2010–2013	PWID	Nador, Morocco								
2010–2013	PWID	Tanger, Morocco								
2010–2013	FSW	Rabat, Morocco								
2010–2013	FSW	Tanger, Morocco								
2010–2013	FSW	Fez, Morocco								
2010–2013	FSW	Agadir, Morocco								
2010–2013	MSM	Marrakesh, Morocco								
2010–2013	MSM	Agadir, Morocco								
2011	PWID	Dakar, Senegal								
2010	FSW	Rwanda								
										
2010	FSW	Kigali, Rwanda								
2011	FSW	Greater Accra, Ghana								
2011	FSW	Ghana								
	FSW	North Eastern Province, Kenya								
	FSW	Nairobi province, Kenya								
	FSW	Urban Kenya								
	MSM	Kenya								
	PWID	Kenya								
2010–2011	PWID	Coastal Kenya								
	PWID	Mainland Tanzania								
	PWID	Kenya								
	PWID	Zanzibar								
	PWID	Africa								
	PWID	Eastern Africa								
2013	FSW	Mekelle, Ethiopia								
	FSW	Durban, South Africa								
	FSW	Mettema Yohannis, Ethiopia								

# Sub article: WEAE0101 Acceptability and preferences for HIV self-testing in Zambia: a population-based formative study using a discrete choice experiment

**Published:** 2016-07-22

**Authors:** A Zanolini, J Chipungu, S Bosomprah, M Mafwenko, C Holmes, H Thirumurthy

**Affiliations:** 1 American Institutes for Research, Lusaka, Zambia; 2 Centre for Infectious Disease Research in Zambia, Lusaka, Zambia; 3 Johns Hopkins University, Lusaka, Zambia; 4 University of North Carolina at Chapel Hill, Chapel Hill, NC, United States

## Abstract

**Introduction:**

Uptake of HIV testing in Zambia remains low and Zambia is considering the use of HIV self-testing (HIVST) to increase awareness. We assessed acceptability and preferences for HIVST among adults in Lusaka province, Zambia.

**Methods:**

Households in Lusaka Province were randomly selected to participate in a household survey and one member aged ≥16 years randomly selected as a respondent. Respondents were asked about perceptions and preferences around HIVST after receiving information about the OraQuick oral fluid-based test. Preferences were assessed through a Discrete Choice Experiment (DCE). The DCE contained a full factorial design with cost (free, 10 Kwacha or 25 Kwacha), location to obtain the test (from voluntary counselling and testing departments in clinics, VCT; outpatients departments within clinics, and private chemists) and pre-counselling (provided or not) as attributes. Participants were asked to choose between two different HIVST models but also had an opt-out option to choose conventional modes of HIV testing or no testing. We used mixed logit regressions to analyze the DCE results.

**Results:**

Among 1617 participants, 47% had not tested in the past year. Seventy-four percent reported feeling comfortable with HIVST and 76% of those who have not tested in the past year reported they would definitely test if given a self-test. Only 2% reported having concerns serious enough to not recommend HIVST in Zambia. In the DCE, 73% of those who had tested in the past year chose HIVST over conventional modes of testing, and 88% of those who had tested in the past year chose HIVST over not testing. The most predictive attribute for a choice was presence of counselling, followed by lower cost especially for regular testers. The lowest relative preference was for location. When considering only two types of HIVST and excluding the opt-out option, participants had a negative preference for obtaining the test at VCT, a location found to be highly stigmatized.

**Conclusions:**

HIVST is highly acceptable among adults in Lusaka province, Zambia. There is a strong positive preference for the provision of some counselling accompanied with HIVST and for lower-cost self-tests, especially for those who were already regular testers.

# Sub article: WEAE0102 Benefits and adverse outcomes of HIV self-testing among high-risk MSM in China: an implementation perspective

**Published:** 2016-07-22

**Authors:** Y Qin, F Liu, W Tang, S Tang, C Liu, J Mao, W Zhang, A Taege, A Nowacki, C Wei, J Tucker

**Affiliations:** 1 UNC Project China, Guangzhou, China; 2 Cleveland Clinic Lerner College of Medicine of Case Western Reserve University, Cleveland, OH, United States; 3 Guangdong Provincial Center for Skin Diseases and STI Control & Prevention, Guangzhou, China; 4 UCSF, San Francisco, CA, United States

## Abstract

**Introduction:**

HIV self-testing (HIVST) holds great promise for reaching high-risk key populations who do not access facility-based services, but it has not been examined in the “real-world” implementation context. HIVST is a process by which a person performs and interprets a test in private. We sought to describe unsupervised HIVST use among men who have sex with men (MSM) in China.

**Methods:**

We conducted a nationwide online survey of MSM in China recruited from MSM websites and social media. Eligible men reported being at least 16 years of age, having anal sex with a man at least once, and having condomless anal/vaginal sex in the past 3 months. We analyzed benefits (e.g. first-time testing, increased testing frequency, post-testing counselling) and adverse outcomes (e.g. coercion, violence, suicidality) among MSM using HIVST. We compared MSM whose first-time HIV test was a self-test (first-time HIVST) to those whose first-time was at a facility and assessed correlates using multivariable logistic regression.

**Results:**

Among 1685 eligible men who clicked the banner, 1189 men completed the survey. A total of 28.7% (341/1189) of men reported ever using HIVST. The most common place to obtain an HIVST kit was online (171/341, 50.1%). Among those who had used HIVST, 58.7% (200/341) reported their first-time HIV test using HIVST. Multivariable analysis found that first-time HIV testing using HIVST was correlated with younger age (adjusted OR=1.05, 95% CI: 1.02, 1.08) and men who had not disclosed MSM behaviours to anyone (adjusted odds ratio (OR)=2.24, 95% confidence interval (CI): 1.57, 3.22). The most common adverse outcome was coercion (31/341, 9.1%). 40/341 (11.7%) of those who underwent HIVST reported a positive HIV self-test. Among men with a positive self-test, 30/40 (75.0%) received post-test counselling and 31/40 (77.5%) received subsequent confirmatory HIV testing. Among men with a negative self-test, 134/301 (44.5%) received post-test counselling and 118/301 (39.2%) confirmed their results.

**Conclusions:**

HIVST is common among Chinese MSM. A substantial portion of them have never previously tested, particularly younger MSM that are not open about their orientation. However, coercion and lack of subsequent test confirmation have been reported. Further implementation research is needed to better understand HIVST outside of research programs.

# Sub article: WEAE0103 “Not without us …”: views on the introduction of HIV self-testing among health care workers providing integrated HIV and sexual and reproductive health services

**Published:** 2016-07-22

**Authors:** C Madanhire, E Sibanda, N Ruhode, K Hatzold, FM Cowan, SN Mavedzenge

**Affiliations:** 1 Centre for Sexual Health and HIV AIDS Research, Harare, Zimbabwe; 2 Population Service International Zimbabwe, Harare, Zimbabwe; 3 Department of infection and Population Health, University College London, London, United Kingdom; 4 RTI International, North Carolina, United States

## Abstract

**Introduction:**

HIV self-testing (HIVST) has potential to increase uptake of HIV testing. Its success depends on various stakeholders’ support, including health care workers (HCW). In preparation for adoption and scale-up of HIVST in Zimbabwe we explored HCW views on HIVST.

**Methods:**

Between December 2015 and January 2016, focus group discussions (FGDs) were held with HCWs providing integrated HIV and sexual and reproductive health services at two Population Services International (PSI) Clinics in Harare and Chitungwiza. Discussions were audio-recorded, transcribed, translated and analyzed thematically.

**Results:**

Four FGDs were held with 10–13 HCWs each, including 18 nurses, 15 counsellors, 4 lab technicians and 6 administrative staff (total=43). HCW had mixed feelings about HIVST. While they generally believed that HIVST can increase testing uptake among men, well-to-do clients and those living in hard-to-reach areas, a recurrent theme was that HIVST poses a threat to HCW jobs. All cadres believed that jobs of HCW who primarily provided counselling were most threatened. HCWs providing other clinical duties (family planning, cervical cancer screening and ART) were perceived to be safer. HCWs had mixed views on whether self-testing would lead to optimized linkage to post-test services. Additionally, it was perceived that while HIVST might be cheaper, this was likely further justification for job losses. The potential for social harms (domestic violence, suicide, and forced-testing) was widely discussed. HCW described fear that devices showing negative results could be “traded” and used to deceive partners of HIV-positive individuals. A good HIVST program was viewed as one which worked with existing health delivery structures and centred on continued HCWs involvement, including counselling before and after testing, and storage of kits by HCWs – thought important due to fears that kits could find their way into uncontrolled informal markets. Educating the community about HIVST was highly recommended.

**Conclusions:**

The potential for HIVST to increase testing uptake, and to be cost-effective, is appreciated by HCWs. There is need to educate HCWs on how HIVST can enhance rather than compete with their roles, with less testing of HIV-negatives HCWs can focus on care, support and retention of HIV-positives, leading to better targeting of resources.

# Sub article: WEAE0104 Index client trailing: a home-based HIV counselling and testing strategy to identify and link people living with HIV to treatment

**Published:** 2016-07-22

**Authors:** N Manjezi, G Fatti, E Mothibi, N Shaikh, O Oyebanji, A Grimwood

**Affiliations:** Kheth'Impilo, Cape Town, South Africa

## Abstract

**Introduction:**

UNAIDS has set targets that 90% of people living with HIV should know their HIV status and that 90% of these should receive antiretroviral treatment by 2020. Implementing innovative programs to help achieve these ambitious targets in sub-Saharan Africa, the epicentre of the global AIDS epidemic, are essential. We report on results of an innovative program in which home-based HIV counselling and testing is offered to household members of known HIV positive clients in South Africa.

**Methods:**

Consenting HIV positive clients (index clients) identified at primary healthcare centres in three high HIV prevalence districts were visited at their homes by lay community-based healthcare workers. Consenting household members of index clients received HIV-related education and HIV counselling and testing. Household members testing HIV positive also received symptom screening for tuberculosis and were referred to HIV care and treatment facilities. The proportions of household members testing HIV positive and proportions linked to treatment facilities over a 14-month period during 2014–2015 were calculated.

**Results:**

14,779 index clients were visited in their homes. 66,766 household members received HIV-related education and counselling (4.5 household members per index client). Amongst these, 59,457 (89.1%) consented to HIV testing (91% and 81% of counselled females and males consented to HIV testing, respectively). Amongst those tested, 9219 (15.5%) were found to be HIV positive. Amongst people testing HIV positive, 8642 (93.7%) were successfully linked to HIV care and treatment facilities. 97.0% of those testing HIV positive received tuberculosis symptom screening, of whom 21.3% were symptom positive. Amongst 2837 children who received HIV testing, 70 (2.5%) were HIV positive and 100% were successfully linked to care and treatment.

**Conclusions:**

Index client trailing utilizing home-based HIV testing by lay healthcare workers in a high HIV prevalence setting resulted in a high uptake of HIV testing, a high yield of people newly diagnosed with HIV, a high proportion with potential concomitant tuberculosis, and a high proportion of adults and children were successfully linked to treatment facilities. This is a strategy which can help sub-Saharan Africa achieve the UNAIDS targets for HIV testing and antiretroviral treatment initiation.

# Sub article: WEAE0105 Results of a cluster-randomized trial of non-financial incentives to increase uptake of couples counselling and testing among clients attending PSI mobile HIV services in rural Zimbabwe

**Published:** 2016-07-22

**Authors:** EL Sibanda, M Tumushime, J Mufuka, S Gudukeya, S Napierala Mavedzenge, S Bautista-Arredondo, H Thirumurthy, S McCoy, N Padian, K Hatzold, A Copas, FM Cowan

**Affiliations:** 1 Centre for Sexual Health and HIV AIDS Research, Harare, Zimbabwe; 2 Population Services International Zimbabwe, Harare, Zimbabwe; 3 RTI International, San Francisco, CA, United States; 4 Mexican National Institute of Public Health, Morelos, Mexico; 5 University of North Carolina at Chapel Hill, Chapel Hill, NC, United States; 6 University of California, Berkeley, CA, United States; 7 Research Department of Infection & Population Health, University College London, London, United Kingdom; 8 Centre for Sexual Health and HIV/AIDS Research (CeSHHAR), Harare, Zimbabwe

## Abstract

**Background:**

Couples HIV testing (CHTS) is associated with greater uptake of HIV prevention/care and is more cost-effective than individual testing, but its uptake remains sub-optimal. Broaching CHTS often results in accusations of infidelity/distrust. Formative research suggests that incentives may mitigate this by changing focus of the pre-test discussion. We investigated the effectiveness of non-financial incentives to increase CHTS uptake among clients accessing PSI's outreach HTS in rural Zimbabwe.

**Methods:**

Sixty-eight rural communities in four districts were randomized 1:1 to incentives or no incentives following formative research on nature of incentives that might stimulate CHTS. In intervention communities, information was promoted that anyone testing with a partner could select a grocery item worth US$1.50. Standard mobilization was done in control communities. Three months after CHTS, willing couple-testers from four communities per arm individually completed a telephone survey to determine whether there were social harms resulting from incentives or CHTS. The effect of incentives on CHTS was estimated using logistic regression with random effects for communities. Testing in the trial is now complete; we report interim data from May to August 2015 in 57 communities but will present final data at the conference.

**Results:**

Of 9721 participants tested, 5652 (58.1%) were in incentives communities. 49.5% and 10.6% in incentive and non-incentive arms, respectively, tested with partners, odds ratio 6.88(95% CI: 4.86–9.72). HIV prevalence was 9.9% (95% CI: 8.2–11.8%) and 6.9% (95% CI: 6.1–7.8%) among couple-testers and individual-testers, respectively; 8.5% of couple-testers had discordant results. 413/697 (59%) eligible participants (176 couples) completed the telephone survey. Motivators for CHTS included desire to know each other's status (93%), incentives (37%) and planning a pregnancy (30%); 22% in incentive arm said they would not have tested without the offer of incentives. Relationship unrest was reported by eight individuals, (1.9%) in the telephone survey, six in incentive arm although none attributed this to incentives. Nine individuals (2.2%) regretted testing with partner, of whom four tested because of incentives. 65.9% said testing programs should offer incentives.

**Conclusions:**

Small incentives are a potentially scalable way to increase CHTS uptake. Although incentives were not reported to cause relationship disharmony, there is need to find better ways of supporting couples with positive/discordant HIV diagnosis.

# Sub article: WEAE0106LB Reaching the first 90: improving coverage of inpatient paediatric provider-initiated HIV testing and counselling using a quality improvement collaborative strategy at 24 health facilities in Tanzania

**Published:** 2016-07-22

**Authors:** G Dougherty, K Clarke, R Fayorsey, M Kamonga, S Kimambo, D Lutkam, C Madevu-Matson, H Mtiro, S Msuka, V Mugisha, M Panya, A Ramadhani, J Sipemba, P Urasa, M Rabkin

**Affiliations:** 1 International Center for AIDS Care and Treatment Programs (ICAP), Columbia University, New York, NY, United States; 2 US Centers for Disease Control and Prevention, Atlanta, GA, United States; 3 Christian Social Services Commission (CSSC), Dar es Salaam, United Republic of Tanzania; 4 US CDC Tanzania, Dar es Salaam, United Republic of Tanzania; 5 Ariel Glaser Pediatric AIDS Healthcare Initiative (AGPAHI), Dar es Salaam, United Republic of Tanzania; 6 ICAP Tanzania, Dar es Salaam, United Republic of Tanzania; 7 Tanzania National AIDS Control Program (NACP), MOHSW, Dar es Salaam, United Republic of Tanzania

## Abstract

**Introduction:**

Tanzania's paediatric HIV testing and treatment rates are suboptimal. Provider-initiated HIV testing and counselling (PITC) is necessary to identify and treat HIV-infected children, and expanding paediatric PITC services is a national priority. Despite rollout of guidelines and training, PITC has not been consistently implemented.

**Description:**

ICAP at Columbia University designed a Quality Improvement Collaborative (QIC) to improve paediatric PITC coverage. Working with CDC, NACP, AGPAHI and CSSC, ICAP launched the QIC at 24 health facilities. Each aimed to improve inpatient PITC coverage to ≥80%, while reducing HIV test kit stock outs and maintaining high linkage rates to care for HIV-infected children. ICAP provided training on QI methods, while AGPAHI and CSSC provided facility-level supportive supervision. Each facility identified contextually appropriate interventions; conducted rapid tests of change using PDSA cycles; and analyzed progress using run charts. ICAP convened quarterly meetings where facility teams compared progress, and a final “harvest” meeting enabled synthesis of lessons learned.

**Lessons learned:**

Change ideas included improvements in staff and client education, staffing, workflow, commodity management, documentation and referrals. A total of 16,569 of 25,282 children (66%) admitted during the intervention period received PITC services; 263 (1.6%) tested positive, and 255 (97%) were enrolled into care. All 24 facilities achieved the QIC target, and the overall inpatient PITC coverage rose from 25 to 70%. Despite increased testing volume, the average number of days with HIV test kit stock outs fell from 8.8 to 1.5/month.

**Conclusions/Next steps:**

Bridging the “know-do gap” is one of the greatest challenges facing HIV programmes. QIC methodology improved coverage of PITC (*what we know works*) by helping facilities to generate local innovations to ensure PITC is consistently implemented (*what we do*). In addition to building QI capacity and improving targeted outcomes, the PITC QIC resulted in a “change package” of successful initiatives that will be disseminated within Tanzania.

**Figure:** Abstract WEAE0106LB–% of children and adolescents admitted to inpatient wards who received HIV PITC and received results and % of rapid test kit (RTK) stock outs

# Sub article: WEAE0201 Current state of the global HIV care continuum

**Published:** 2016-07-22

**Authors:** R Granich, S Gupta, I Hall, J Aberle-Grasse, S Hader, J Mermin

**Affiliations:** 1 International Association of Physicians in AIDS Care (IAPAC), Washington, DC, United States; 2 International Association of Physicians in AIDS Care (IAPAC), Delhi, India; 3 Centers for Disease Control and Prevention, Atlanta, GA, United States

## Abstract

**Introduction:**

In 2014, UNAIDS issued the 90-90-90 HIV response targets: by 2020, 90% of individuals living with HIV will know their HIV status, 90% of people with diagnosed HIV infection will receive ART and 90% of those taking ART will be virally suppressed. Consistent methodology and routine reporting in the public domain are necessary for tracking progress towards the 90-90-90 targets.

**Methods:**

For 2011–2015, we searched PubMed, UNAIDS country progress reports, WHO/UNAIDS reports, national surveillance and programme reports, and conference presentations and/or abstracts for the latest available national HIV care continuum and estimation methods. We ranked continuum with described estimation methods for indicators to derive high, medium and low-quality continuum.

**Results:**

We identified 48 national care continuum in the public domain representing 58% of the 2013 global estimate of people living with HIV available. Eleven continuum were excluded from further analysis for either not providing estimates of viral load or substantial problems with representativeness. Of the remaining 37, four (with <1% of global burden) were high quality, using standard surveillance methods to derive an overall denominator and programme data from national cohorts for estimating steps in the continuum. Of the 37 countries with adequate data, the average proportion of the aggregate of people living with HIV from all countries receiving ART was 37%, and virally suppressed was 29%. Care continuum from only six countries in sub-Saharan Africa were available.

**Conclusions:**

Relatively few complete national continuum are available in the public domain, and there is a wide variation in methodologies for describing progress towards treatment and viral suppression targets. Standardized continuum of care monitoring and evaluation based on a national programme cohort of everyone living with diagnosed HIV would be a major step towards improving the use of scarce resources to achieve 90-90-90 through improved efficiency, transparency, accountability and impact.

**Figure 1:** Proportion of people living with HIV with diagnosed infection versus those on antiretroviral treatment (ART).

# Sub article: WEAE0202 Same-day HIV testing and antiretroviral therapy initiation results in higher rates of treatment initiation and retention in care

**Published:** 2016-07-22

**Authors:** S Koenig, N Dorvil, P Severe, C Riviere, M Faustin, C Perodin, C Paul, A Apollon, G Saintil, L Duverger, E Dumont, B Hedt-Gauthier, K Hennessey, V Rivera, J Devieux, JW Pape

**Affiliations:** 1 Division of Global Health Equity, Department of Internal Medicine, Brigham and Women's Hospital, Boston, MA, United States; 2 GHESKIO, Port-au-Prince, Haiti; 3 Harvard Medical School, Boston, MA, United States; 4 Analysis Group, Boston, MA, United States; 5 Center for Global Health, Weill Cornell Medical College, New York, NY, United States; 6 Florida International University, Miami, FL, United States

## Abstract

**Introduction:**

High rates of pre-ART attrition are widely reported. Retention may be improved if pre-ART services could be effectively provided in 1 day.

**Methods:**

We conducted a randomized study comparing standard and same-day ART initiation for adult patients (age >17 years) who presented for HIV testing with WHO stage 1 or 2 disease and CD4 count≤500 cells/mm^3^ at GHESKIO in Port-au-Prince, Haiti. All participants received same-day HIV and CD4 count testing, TB screening and physician evaluation. The standard group initiated ART at the third follow-up visit (day 21); the same-day group initiated ART on the day of presentation. The only difference in services provided was the timing of ART initiation. Participants were followed for 12 months.

**Results:**

Between August 2013 and October 2015, 762 participants were randomized to standard (n=384) or same-day ART (n=378) (see Table 1). Twenty-four participants in the standard and 18 in the same-day ART group transferred during the study period and were removed from all analyses; this left 360 participants in each group. ART was initiated within 90 days in 329 (91%) of participants in the standard and 100% in the same-day ART group (p<0.001). A total of 577 participants (80%) have completed 12 months of potential follow-up time (290 in standard; 287 in same-day ART groups). In the standard group, 212 participants (73%) were retained, 17 (6%) died, 56 (19%) were lost to follow-up (LTFU) and five (2%) were late returners. In the same-day ART group, 230 participants (80%) were retained, eight (3%) died, 46 (16%) were LTFU and three (1%) were late returners. Twelve-month retention was higher in the same-day ART group (p=0.046).

**Conclusions:**

Same-day ART is associated with higher rates of ART initiation and retention, compared with standard ART initiation. These findings suggest that immediate ART is feasible in PEPFAR “Test and Start” recommendations.

**Abstract WEAE0202–Table 1:** Outcomes for Participants in the Standard and Same-day ART Groups

		Standard group (n=360)	Same-day group (n=360)	p
**Baseline characteristics**	Female sex – no. (%)	183 (51%)	169 (47%)	0.296
	Age – mean (SD)	38 (10)	38 (10)	0.296
	CD4 count – mean (SD)	244 (129)	241 (124)	0.781
**Pre-ART outcomes**	Completed CD4 count – no. (%)	360 (100%)	360 (100%)	–
	Initiated ART within 90 days after HIV testing – no. (%)	329 (91%)	360 (100%)	**<0.001**
**Outcomes 12-months post-ART**	Retained in care – no. (%)	212 (73%)	230 (80%)	**0.046**
**(among participants with**	Late returners – no. (%)	5 (2%)	3 (1%)	0.486
**12 months of potential**	Lost to follow-up – no. (%)	56 (19%)	46 (16%)	0.302
**follow-up time)**	Died – no. (%)	17 (6%)	8 (3%)	0.062

The numbers in bold are statistically significant.

# Sub article: WEAE0203 Towards the last 90% of the 90-90-90 strategy: a review of viral suppression rates in a HIV programme in central and eastern Kenya

**Published:** 2016-07-22

**Authors:** M Kitheka, K Curran, P Gathii, M Mudany, S Bii

**Affiliations:** 1 Jhpiego Kenya, Jhpiego, Nairobi, Kenya; 2 Jhpiego, Baltimore, MD, United States; 3 Jhpiego, Nairobi, Kenya; 4 USAID, Nairobi, Kenya

## Abstract

**Introduction:**

A total of 1.6 million Kenyans live with HIV with 101,560 infected annually. Kenya has adapted UNAIDS’ 90-90-90 initiative under the Kenya AIDS Strategic Framework and the Acceleration plan for HIV Care and Treatment. To monitor the last 90%, Kenya introduced viral load (VL) monitoring, with VLs done for clients after 6 and 12 months on ART then annually thereafter. Health facilities submit samples to two national laboratories, one doing the dry blood spot and the other frozen plasma. Viral suppression is defined as VL less than 1000 copies/ml.

**Description:**

APHIAPLUS KAMILI, a PEPFAR/USAID funded project, is led by Jhpiego and supports care and treatment in 142 facilities in Eastern and Central Kenya. As of December 2015, the project supported 35,132 clients on ART, 9.5% (3332) of whom were children under 15 years. To launch VL monitoring, APHIAPLUS KAMILI sensitized providers on specimen collection, patient follow-up and financed specimen transport to laboratories. Results are posted online, and hard copies sent to facilities. This review analyzed VL results from January to December 2015 obtained from the National VL Laboratories database.

**Lessons learned:**

A total of 24,030 samples were sent to laboratories. In this review, we excluded results with any incomplete entry (age, sex and results) and all rejected samples. A total of 15,253 samples had complete data for review. The crude viral suppression rate was 84% (12,750) with 16% (2503) above 1000 copies/ml. In total, 84% (8864) of women and 82% (3886) of men were suppressed (P-value 0.003). Viral suppression classified by age was 77% (2656) for children age below 15 years and 85% (10,094) for clients aged above 15 years (P-value<0.001). Viral suppression for client type ranged from 68% among pregnant women, 77% suspected clinical failure, 82% immunological failure and 84% for routine VL monitoring (P-value=0.006).

**Conclusions/Next steps:**

The sites reviewed achieved fairly high levels of viral suppression among those clients who received VL testing, but are still below 90% for all populations. Viral suppression is higher among non-pregnant adults than among children, adolescents and pregnant women; these populations may need additional adherence support. The high suppression rates among clients with suspected treatment failure warrant additional investigation

# Sub article: WEAE0204 Eliminating CD4 thresholds in South Africa will not lead to large increases in persons receiving ART without further investment in testing, linkage and initiation

**Published:** 2016-07-22

**Authors:** J Bor, S Ahmed, MP Fox, S Rosen, I Katz, F Tanser, D Pillay, T Bärnighausen

**Affiliations:** 1 Department of Global Health, School of Public Health, Boston University, Boston, MA, United States; 2 Africa Centre for Population Health, Mtubatuba, South Africa; 3 Health Economics and Epidemiology Research Office, University of Witwatersrand, Johannesburg, South Africa; 4 Department of Epidemiology, School of Public Health, Boston University, Boston, MA, United States; 5 Massachusetts General Hospital, Boston, MA, United States; 6 Harvard Medical School, Boston, MA, United States; 7 Division of Women's Health, Brigham and Women's Hospital, Boston, MA, United States; 8 Faculty of Medicine, University College London, London, United Kingdom; 9 Department of Global Health and Population, Harvard T. H. Chan School of Public Health, Boston, MA, United States

## Abstract

**Introduction:**

It is hoped that eliminating CD4 count thresholds for ART eligibility will increase the number of HIV-infected persons receiving therapy and reduce transmission of HIV. However, little is known about the impact of relaxing eligibility thresholds on uptake of ART.

**Methods:**

Clinical records were analyzed for all patients presenting for HIV care in the Hlabisa sub-district public sector ART programme. We estimated the distribution of first CD4 counts for patients presenting in 2013 (Figure 1a). We then estimated the conditional probability of ART initiation within 6 months for each CD4 count under two counterfactual states of the world (Figure 1b): *if CD4-eligible* and *if not CD4-eligible*. Multiplying the conditional probabilities by the distribution of CD4 counts, we estimated the probability that a person would initiate ART under expanded guidelines (CD4<500, or elimination of CD4 criteria) and under older guidelines (CD4<350). We forecast the number of new initiators expected if South Africa adopts new WHO recommendations.

**Results:**

In 2013, 20.0% of patients presented at 350–500 cells and 38.9% >500 cells. 8.4% of patients 350–500 cells and 8.0% of patients >500 cells would have initiated ART under the old guidelines. 29.7% of patients 350–500 cells and 19.2% of patients >500 cells would initiate ART if CD4 criteria were eliminated. 62.1% of patients at 350–500 cells and 72.8% >500 cells are not expected to initiate under expanded guidelines despite being eligible. If these numbers hold nationally, then South Africa can expect 130,000 additional initiators per year from raising the threshold to 500 and a further 164,000 initiators per year from eliminating CD4 criteria, representing a 5% increase in persons on ART.

**Conclusions:**

Removing CD4 criteria alone, without improving HIV testing, linkage, and ART initiation procedures, will not achieve the country's 90-90-90 targets.

**Figure 1:** Distribution of first CD4 counts and probability of starting ART at different CD4 counts.

# Sub article: WEAE0205 First-year intervention outcomes of the Bukoba Tanzania combination prevention evaluation: promising HIV testing and linkage-to-care methods to achieve 90-90-90

**Published:** 2016-07-22

**Authors:** D MacKellar, H Maruyama, R Weber, O Ernest, S Porter, J Gikaro, G Alexander, G Kundi, J Byrd, K Kazaura, D Mbilinyi, F Morales, J Justman, R Josiah

**Affiliations:** 1 Division of Global HIV/AIDS, Centers for Disease Control and Prevention, Atlanta, GA, United States; 2 ICAP at Columbia University, Dar es Salaam, United Republic of Tanzania; 3 CTS Global, Inc., assigned to Centers for Disease Control and Prevention, Dar es Salaam, United Republic of Tanzania; 4 Centers for Disease Control and Prevention, Atlanta, GA, United States; 5 ICF International, Atlanta, GA, United States; 6 Centers for Disease Control and Prevention, Dar es Salaam, United Republic of Tanzania; 7 ICAP at Columbia University, New York, NY, United States; 8 National AIDS Control Program, Ministry of Health and Social Welfare, Dar es Salaam, United Republic of Tanzania

## Abstract

**Introduction:**

In an urban lake-zone district of 129,000 people, the Bukoba Tanzania Combination Prevention Evaluation (BCPE) aims to increase antiretroviral therapy (ART) coverage among eligible HIV-infected adult residents from an estimated 34% in 2013 to 80% after a 2-year intervention. First-year objectives to achieve this aim include conducting 45,000 HIV tests among adults (18,000 among men), registering 1360 persons for HIV care and initiating ART on 90% of eligible patients (CD4<350). This abstract summarizes BCPE first-year intervention outcomes, October 2014–September 2015.

**Description:**

BCPE interventions include provider-initiated (PITC) and community-based (CBHTC) HIV testing and counselling (HTC) and integrated linkage case management (LCM). Conducted in 11 outpatient-department clinics, PITC includes routine eligibility screening and referral for on-site HTC. CBHTC is offered at homes and at male-frequented venues throughout the district. Provided to consenting HIV-infected out-of-care clients for up to 90 days, LCM includes escort and expedited first-visit care at nine HIV clinics, and counselling on HIV care and disclosure. Outcomes of HTC and of LCM clients whose cases were closed through September 2015 were compiled and analyzed.

**Lessons learned:**

Of 56,907 HIV tests conducted, 48,752 (86%) were among adults >macr;15 years of age (18,648 tests among adult males). PITC accounted for 64% of tests and 79% of 2292 HIV-infected out-of-care persons identified (86% newly HIV diagnosed). Of 1519 (75%) clients of closed LCM cases (516 clients were still under management at compilation), 1369 (90%) had registered for HIV care, of whom 50% were initiated on ART. Similar percentages of HIV-infected out-of-care women and men consented to LCM, registered for care and were initiated on ART (Table).

**Conclusions/Next steps:**

BCPE HTC and linkage-to-care interventions met first-year objectives and are promising methods that might help similar programmes achieve 90-90-90 targets. Next steps for BCPE include initiating ART at CD4<500 (approved December 2015), increasing LCM participation rates, and in 2017, conducting the endline evaluation to assess achievement of 80% ART coverage among eligible adult residents.

**Abstract WEAE0205–Table:** First-year HIV-Testing and Linkage: case-management outcomes of the Bukoba Tanzania Combination Prevention Evaluation

	HTC outcomes			Linkage case management outcomes					
		
BCPE clients	Total tests	PITC tests	HIV+ Out-of-care	LCM consent	Closed cases	HIV-care registered	CD4 recorded	CD4 count <350	ART (of registered)
	n	(%)	n (%)	(%)	n	n (%)	(%)	n (%)	n (%)
All	56,907	(64)	2292 (4)	(86)	1519	1369 (90)	(83)	594 (47)	689 (50)
Female	34,207	(71)	1427 (4)	(85)	932	830 (89)	(81)	303 (40)	408 (49)
Male	22,700	(53)	865 (4)	(88)	587	539 (92)	(86)	291 (57)	281 (52)

# Sub article: WEAE0206LB LINK4HEALTH: a cluster-randomized controlled trial evaluating the effectiveness of a combination strategy for linkage to and retention in HIV care in Swaziland

**Published:** 2016-07-22

**Authors:** M McNairy, M Lamb, A Gachuhi, H Nuwagaba-Biribonwoha, S Burke, S Mazibuko, V Okello, P Ehrenkranz, R Sahabo, W El-Sadr, Link4Health Study Group

**Affiliations:** 1 ICAP Columbia University, New York, NY, United States; 2 Department of Medicine, Weill Cornell Medicine, New York, NY, United States; 3 ICAP Columbia University, Mbabane, Swaziland; 4 Ministry of Health, Kingdom of Swaziland, Mbabane, Swaziland, Gates Foundation, Seattle, WA, United States; 5 Gates Foundation, Seattle, WA, United States

## Abstract

**Introduction:**

Gaps in the HIV care continuum contribute to suboptimal individual health outcomes and increased HIV transmission. Practical interventions targeting known barriers to care are needed.

**Methods:**

Link4Health, a cluster-randomized controlled trial, evaluated the effectiveness of a combination intervention strategy (CIS) versus standard of care (SOC) on the combined outcome of linkage to care within 1 month and retention in care at 12 months after HIV diagnosis. CIS included: point-of-care CD4 at the time of HIV+ test, accelerated antiretroviral treatment (ART) for adults with CD4 <350 cells/µL, mobile phone appointment reminders, health educational packages and non-cash financial incentives. Ten study clusters in Swaziland, each consisting of a network of affiliated HIV clinics, were randomized to CIS versus SOC. Adults ≥18 years newly tested HIV+ and willing to receive HIV care at the study unit were enrolled from August 2013–November 2014 and followed for 12 months.

**Results:**

A total of 2201 individuals were enrolled (1100 CIS arm; 1101 SOC arm). The majority were female (59%); median age was 32 years (IQR 26–40). In intention-to-treat analysis, 64% (705/1100) adults at CIS sites achieved the primary outcome versus 43% (477/1; 101) at SOC sites (relative risk (RR): 1.48, 95% CI: 1.36–1.60, p < 0.0001), with similar result when adjusted for clustering. Participants in the CIS versus the SOC study arm had higher linkage to care within 1 month (92% vs. 83%, RR: 1.10, 95% CI: 1.07–1.14, p<0.0001); higher 12-month retention (65% vs. 45%, RR: 1.45, 95% CI: 1.34–1.56, p<0.0001); and lower death before ART initiation (1% vs. 2%, RR: 0.44, 95% CI: 0.21–0.91, p = 0.02). A higher proportion of those lost to follow-up at 12 months were pre-ART compared to ART patients. The effectiveness of the CIS intervention did not differ by age, sex, distance to clinic or clinic type.

**Conclusions:**

A combination strategy of pragmatic evidenced-based interventions, aimed at gaps in the HIV care continuum, was associated with a 50% increase in prompt linkage to care and 12-month retention. This strategy offers promise for enhanced outcomes among HIV-infected patients and for decreased transmission to others.

# Sub article: WEAE0301 Implementation of routine viral load monitoring in Lesotho, Malawi, Mozambique and Zimbabwe: a cascade analysis

**Published:** 2016-07-22

**Authors:** M Dhodho, M Frieden, A Shroufi, E Wanjiru, S Daho, E Simons, H Bygrave

**Affiliations:** 1 MSF Zimbabwe, Médecins Sans Frontières, Harare, Zimbabwe; 2 MSF South Africa, Médecins Sans Frontières, Cape Town, South Africa; 3 MSF Malawi, Médecins Sans Frontières, Blantyre, Malawi; 4 MSF Mozambique, Médecins Sans Frontières, Maputo, Mozambique; 5 Southern Africa Medical Unit, Médecins Sans Frontières, Cape Town, South Africa

## Abstract

**Introduction:**

Routine viral load (VL) to monitor the response to ART has been recommended by WHO since 2013. From 2012 routine VL testing to monitor ART was introduced in MSF projects in Lesotho, Malawi, Mozambique and Zimbabwe. All districts except Changara were rural settings where ART had been extensively decentralized. VL is performed annually in all sites except Malawi (2 yearly). To assess programmatic implementation of routine VL, an analysis was carried to assess performance at each step of the VL algorithm.

**Methods:**

Analyses were performed between January and November 2015 across six districts in four countries. Reviews of clinical and laboratory records of representative samples of patients were used to determine how each step of the routine VL algorithm (coverage of VL, uptake of enhanced adherence counselling, repeat VL testing (within 2–9 months), re-suppression and appropriate switch to second-line ART) was implemented within a defined period according to local guidelines (18 months preceding date of analysis in Lesotho, Mozambique and Zimbabwe and 30 months in Malawi). Results were presented to programme staff and barriers for implementation identified.

**Results:**

In those sites with low coverage of VL1 and VL2 challenges included lack of human resources to draw blood, dedicated staff to perform enhanced adherence counselling and lack of effective appointment and tracing mechanisms. Across all sites reluctance to task shift and decentralize second line ART care was cited as a barrier to switching.

**Conclusions:**

This analysis demonstrated limited compliance with a routine VL algorithm based on WHO recommendations. Scale-up plans for VL monitoring must address human resource issues and make implementation plans for provision of second-line in sites where ART care has been decentralized.

**Abstract WEAE0301–Table 1:** Outcomes of VL cascade analysis

Site	Buhera, Zimbabwe	Gutu, Zimbabwe	Thyolo, Malawi	Nsanje, Malawi	Roma, Lesotho	Moz Changara (3000 copies/ml is threshold for action throughout algorithm)
Year routine VL testing started	2012	2013	2012	2013	2014	2013
Number of patients in the analysis	4760	2978	7576	2785	3069	3095
Coverage of routine VL testing (VL1)	91%	74%	56%	32%	70%	62%
VL > 1000 copies/ml	14%	15%	9%	20%	10%	40%
EAC documented for patients with VL >1000 copies/ml	57%	76%	62%	56%	70%	70%
Repeat VL test performed (VL2)	68%	67%	55%	40%	42%	23%
Resuppressed to <1000 copies/ml	43%	39%	46%	32%	8%	22%
VL threshold for switch to second-line ART (copies/ml)	1000	1000	5000	5000	1000	3000
Eligible patients switched to second-line ART	37%	35%	15%	38%	37%	10%

# Sub article: WEAE0302 Viral load cascade and programmatic challenges after 2 years of routine HIV viral load testing in Maputo, Mozambique

**Published:** 2016-07-22

**Authors:** R Giuliani, A Torrens, L Molfino, C Silva, T Ellman, A Magaia

**Affiliations:** 1 Médecins Sans Frontières, Maputo, Mozambique; 2 SAMU – South Africa Medical UnitMédecins Sans Frontières, Cape Town, South Africa; 3 Ministry of Health, Maputo, Mozambique

## Abstract

**Introduction:**

Médecins Sans Frontières (MSF) together with the Ministry of Health (MoH) introduced routine viral load (VL) monitoring in December 2013 for ART-enrolled patients in Maputo city, Mozambique. This analysis aims to describe, the VL cascade outcomes and the programmatic challenges of routine VL monitoring and counselling intervention after 2 years of implementation.

**Methods:**

A retrospective cohort study design with routine programme data was used. The study was conducted between July 2013 and March 2015 in six MSF supported health centres in Maputo city where routine VL monitoring with enhance adherence counselling (EAC) for patients with detectable VL (=3000 cp/ml as per national guidelines) was implemented. All HIV patients more than 6 months on ART were included in the study. Data were analyzed using Stata software version 14. Percentages (%) were calculated to report coverage detectability at first VL, coverage of EAC, VL re-suppression and switch to second line treatment.

**Results:**

Among 45,591 ART eligible patients, 14,026 (30.8%) had at least one VL. Median age was 37.5 years (24–51), 91.4% were above 15 years and 76.3% were female. Detectability rate was 19.8% (2617) at VL≥3000 and 27.% (3569) at VL≥1000 cp/ml. 34.5% of patients <15 years had a VL≥3000 compared to 17.5% of adults ≥15 years. Seven hundred and two (26.8%) high VL patients did at least one EAC session. Six hundred and sixty-nine (36%) patients with high VL had a follow up VL at least 3 months after. Two hundred and forty-nine (37.3%) re-suppressed. Out of 420 patients with the second high VL, 197(47%) were referred and approved by the ART Committee to regime change and 59 (30%) have switched to second line so far.

**Conclusions:**

Viral load coverage remains low after 2 years. The implementation of routine VL requires a multi-sectorial approach and a well-established VL flow. Outcomes reveal high failure rate and the importance of implementing early adherence interventions to prevent developing of treatment failure, specifically for children and adolescents. Access to 2nd line ART for patients in failure is still limited. Ensuring access to 2nd line should be a priority alongside ensuring patients with low VL are fast tracked into a differentiated model of care.

# Sub article: WEAE0303 Roll out of targeted viral load testing in two rural districts within Masvingo Province, Zimbabwe

**Published:** 2016-07-22

**Authors:** J van Dijk, K Kamenova, A Shamu, K Pfeiffer, M Hobbins, J Ehmer, J Murungu

**Affiliations:** 1 SolidarMed, Masvingo, Zimbabwe; 2 Provincial Medical Directorate, Ministry of Health and Child Care, Masvingo, Zimbabwe; 3 SolidarMed, Lucerne, Switzerland; 4 AIDS & TB Programmes, Ministry of Health and Child Care, Harare, Zimbabwe

## Abstract

**Introduction:**

Following the World Health Organization's recommendation of viral load (VL) testing as the preferred approach for monitoring anti-retroviral therapy treatment outcomes, the Zimbabwe Ministry of Health and Child Care (MoHCC), with support of the medical relief organization SolidarMed, committed to improve access to targeted VL (TVL) testing, thus offering VL testing to patients with suspected treatment failure.

**Description:**

After training of Healthcare Workers (HCWs), TVL testing, as defined in the national guidelines, was newly introduced to all 48 health facilities (HF) in two districts in Masvingo Province in 2013. With limited access to VL measurements nationally, specimens were collected as dried blood spots and processed at an accredited laboratory in South Africa (SA) using bioMerieux-Platform. Samples were collected at four hospitals within the districts, transported via existing sample transportation networks and sent to SA by courier. Results were communicated back within 2 weeks via secured web-link to the central hub in Masvingo and distributed to the sites. Bi-monthly clinical mentoring visits to HF took place.

**Lessons learned:**

High sample success rate (99%), short turnaround time (TAT) of test-results and moderate costs (USD25/sample, all-inclusive) were major advantages of this approach. Low proportion of patients identified for TVL based on routine clinical and immunological screening by HCWs, and low percentage of repeat VLs done, remain concerning.

**Conclusions/Next steps:**

Zimbabwe MoHCC's VL scale-up strategy 2015–2018, plans to role out VL testing to district and Rural Health Center level in 2016. With current insufficient national laboratory capacity, outsourcing of sample processing to SA can allow for testing targets to be reached. However, the low number of TVL done so far in the two districts suggests that the overall success of roll-out heavily depends on the skills and awareness of HCWs to identify eligible patients, as well as on system strengthening to improve patient follow-up and timely switch to 2nd line treatment in case of confirmed treatment failure.

**Abstract WEAE0303–Table 1:** TVL in two districts in Masvingo Province, Zimbabwe, during 2013–2015

	2013	2014	2015
Number of patients on ART by year end	16,506	17,892	21,788
Patients on ART with 1st VL (N/%)	150/0.9%	315/1.8%	368/1.7%
VL test results available (N/%)	149/99.3%	313/99.4%	360/97.8%
VL undetectable (<20 copies/ml) (N/%)	44/29.5%	88/28.1%	119/33.1%
VL detectable at >3000 copies/ml (N/%)	76/51.0%	172/55.0%	167/46.4%
Patients with detectable VL who had a repeat VL done (N/%)	38/50.0%	71/41.3%	52/31.1%
Patients with confirmed virological failure on repeat VL (N/%)	37/97.4%	55/77.5%	43/82.7%

# Sub article: WEAE0304 Dried blood spots provide accurate enumeration of HIV-1 viral load in east Africa

**Published:** 2016-07-22

**Authors:** R Barnabas, R Coombs, M Chang, T Schaafsma, S Asiimwe, K Thomas, J Baeten, C Celum

**Affiliations:** 1 Department of Global Health, University of Washington, Seattle, WA, United States; 2 University of Washington, Seattle, WA, United States; 3 Integrated Community Based Initiatives, Kabwohe, Uganda

## Abstract

**Introduction:**

HIV-1 viral load monitoring of antiretroviral therapy (ART) confirms treatment efficacy and facilitates timely switching to second line regimens. However, collection of plasma for ART monitoring requires phlebotomy, controlled transport conditions and is costly, thereby limiting its use in community-based settings. The use of dried blood spot (DBS) cards to collect finger-prick blood and transport specimens at ambient temperature to a central laboratory for viral load testing would simplify monitoring but requires validation against the gold-standard method of plasma testing.

**Methods:**

In a randomized study of pre-exposure prophylaxis (PrEP) among HIV serodiscordant couples in Kenya and Uganda (the Partners PrEP Study), HIV-infected participants provided EDTA plasma and DBS specimens at the same visit. The Abbott RealTime HIV-1 assay was used to measure viral load in plasma (limit of quantification (LOQ): 80 copies/mL), and a modified assay was used for DBS whole blood specimens (LOQ: 520 copies/mL); all testing was done on samples transported to the University of Washington. We selected 165 HIV-positive participants including 34 men and women on ART, and 131 not on ART across a range of CD4 count categories. The plasma and DBS viral load results were compared using Bland-Altman plots and two by two tables.

**Results:**

The median viral load was 3.49 and 3.10-log_10_ copies/mL for plasma and DBS, respectively. The mean difference between plasma and DBS specimens was 0.17-log_10_ copies/mL (CI: 0.08 to 0.26-log_10_ copies/mL). The correlation between plasma and DBS results was 0.85 (p<0.0001). At the WHO viral suppression threshold of <1000 copies/mL in plasma and <624 copies/mL with DBS, the sensitivity and specificity of DBS was 87 and 86%, respectively, with positive and negative predictive values of 46 and 98%, respectively, for detecting treatment failures in a population with 13% virological failures. There were no significant differences by gender, CD4 count or ART use.

**Conclusions:**

DBS specimens provide a highly comparable result to plasma viral load and thus might be used for population-based ART monitoring. DBS and plasma specimens should be compared in the field to determine the appropriate threshold and optimal predictive values for identifying treatment failures in decentralized settings.

# Sub article: WEAE0305 Monitoring of HIV-1 RNA with point-of-care cepheid Xpert HIV-1 viral load in rural African communities is feasible and reliable in the era of broad scale up of ART

**Published:** 2016-07-22

**Authors:** S Moyo, T Mohammed, K Wirth, K Bennett, MP Holme, L Mupfumi, P Sebogodi, NO Moraka, C Boleo, CN Maphorisa, B Seraise, S Gaseitsiwe, RM Musonda, E van Widenfelt, KM Powis, T Gaolathe, EJ Tchetgen Tchetgen, JM Makhema, M Essex, S Lockman, V Novitsky

**Affiliations:** 1 Research Laboratory, Botswana Harvard AIDS Institute (BHP), Gaborone, Botswana; 2 Faculty of Medicine and Health Sciences, Division of Medical Virology, University of Stellenbosch, Tygerberg, South Africa; 3 Botswana Harvard AIDS Institute (BHP), Gaborone, Botswana; 4 Department of Epidemiology, Harvard T.H. Chan School of Public Health, Boston, MA, United States; 5 Department of Immunology and Infectious Diseases, Harvard T.H. Chan School of Public Health, Boston, MA, United States; 6 Bennett Statistical Consulting Inc., Ballston Lake, NY, United States; 7 Department of Medicine, Massachusetts General Hospital, Boston, MA, United States; 8 Department of Pediatrics, Massachusetts General Hospital, Boston, MA, United States; 9 Department of Biostatistics, Harvard T.H. Chan School of Public Health, Boston, MA, United States; 10 Division of Infectious Diseases, Department of Medicine, Brigham and Women's Hospital, Boston, MA, United States

## Abstract

**Introduction:**

Increasing scale-up of ART necessitates routine HIV-1 viral load (VL) testing for monitoring treatment failure and adherence. In rural settings, laboratory-based VL testing remains challenging (insufficient access to laboratory facilities, cold chain and sample transportation) and delays in result reporting may negatively impact the HIV treatment cascade. Point-of-care (POC) VL testing has the potential to alleviate these challenges, particularly in rural communities.

**Methods:**

We compared the performance of the Cepheid Xpert^^®^^ HIV-1 VL (POC assay) against the Abbott m2000sp/m2000rt (Abbott assay). ART-naïve individuals (n=168) in 12 rural communities participating in the Botswana Combination Prevention Project provided EDTA blood specimens during household surveys. POC assay testing was completed in mobile community-based facilities, while the Abbott assay was performed in the reference laboratory. Bland-Altman and Passing-Bablok regression were used to test for systematic and proportional differences. Correlation analysis was performed using the Spearman rank test.

**Results:**

We found very high correlation between the POC and Abbott assay results (r_s_=0.89). The POC assay results were 0.46 log_10_ (95% confidence interval (CI): −0.38–1.31; Figure 1A) higher than the Abbott on average, but this difference was not significant. In contrast, Passing-Bablok regression, no proportional bias was observed (slope=0.97; 95% CI: 0.91–1.03; Figure 1B), but a test for systematic bias was significant (intercept=0.58; 95% CI: 0.34–0.82). Agreement of 96, 93 and 88% was found at the 40, 400 and 1000 copies/mL thresholds, respectively. Seven samples with VL between 50 and 115 copies/mL on the POC assay were below the limit of detection with the Abbott assay. One sample with undetectable VL on the POC assay had VL of 66 copies/mL with the Abbott assay.

**Conclusions:**

The Xpert HIV-1 VL assay showed high agreement and high accuracy compared to the standard laboratory-based method of VL testing. This POC assay is a promising tool for monitoring ART scale-up in rural communities.

**Abstract WEAE0305–Figure 1:** Comparison of Xpert HIV-1 RNA Assay using difference plot and passing-bablok regression

# Sub article: WEAE0306LB Using epidemiology and collaborative funding to enable innovation in opportunistic screening to reduce the late diagnosis of HIV: interim results from a targeted primary care project in England (UK)

**Published:** 2016-07-22

**Authors:** O Brigstock-Barron, L Logan, H Sowerbutts, V Womack, M Osman, J Anderson, A Nardone

**Affiliations:** 1 Health & Wellbeing, Public Health England, London, United Kingdom; 2 Leeds City Council, Leeds, United Kingdom; 3 NHS Leeds North Clinical Commissioning Group, Leeds, United Kingdom; 4 Elton John AIDS Foundation, London, United Kingdom; 5 Centre for the Study of Sexual Health and HIV, Homerton University Hospital Trust, London, United Kingdom

## Abstract

**Introduction:**

The estimated percentage of diagnosed HIV in England remains below the UNAIDS target and rates of late HIV diagnosis remain high. In 2015, Public Health England (PHE) and the Elton John AIDS Foundation (EJAF) collaborated to co-fund a project aimed at increasing HIV testing in geographical areas with high incidence of HIV and high rates of late HIV diagnosis. PHE undertook an epidemiological analysis of large urban areas in England to select potential areas to run the intervention. This approach ensured investment was targeted to achieve the greatest impact.

**Methods:**

The selected intervention targets new patients registering with primary care services in high HIV prevalence districts of a large city in the north of England, offering them routine Blood Borne Virus (BBV) testing. The project has developed innovative promotional resources in languages targeting those most at risk (Czech, Tigrigna and Arabic) and targeted training to enable staff to deliver the intervention. Local primary care teams have also developed a new digital prompt protocol aimed at increasing HIV testing in patients presenting with associated clinical indicator illnesses.

**Results:**

During the first five months of implementation, 31 primary care practices were enrolled in which 8401 eligible new patients between the ages of 16 and 65 were registered. So far 19% (1616) of patients have been offered tests for HIV, HBV and HCV, of those, 87% (1405) were tested. Of those tested, there were five, nine and four positive results for HIV, HBV and HCV, respectively, representing positivity rates of 0.36, 0.64 and 0.28%.

**Conclusions:**

Using collaborative funding approaches targeted at the areas of high burden of infection/late diagnosis shows high impact. Interim findings demonstrate positivity rates higher than the background diagnosed prevalence's for those areas and high test uptake rates demonstrate good patient acceptability. Feedback so far has indicated that the scheme and associated training has increased healthcare workers’ awareness and confidence in offering BBV screening. Development of an effective digital prompt tool will allow clinical staff to identify individuals at risk more easily. Resources developed are of national and international relevance and show that specific targeting of limited funding in high prevalence areas is effective.

# Sub article: THAA0101 Frequent and “burst-like” reactivation from latency in SIVmac239M infected macaques

**Published:** 2016-07-22

**Authors:** M Pinkevych, A Reynaldi, C Fennessey, C Reid, P Nadella, L Lipkey, L Newman, V Ayala, S Jain, G Del Prete, J Estes, D Ott, J Lifson, C Ohlen, B Keele, M Davenport

**Affiliations:** 1 Kirby Institute for Infection and Immunity, University of New South Wales, Sydney, NSW, Australia; 2 AIDS and Cancer Virus Program, Leidos Biomedical Research Inc, Frederick National Laboratory for Cancer Research, Frederick, MD, United States

## Abstract

**Introduction:**

Anti-retroviral treatment interruption is usually followed by the recrudescence of infection within a few weeks. This is thought to arise from the reactivation and viral production of a number of latently infected cells. However, very little is known about the basic dynamics of reactivation from latency, such as the frequency of latent cell reactivation, whether reactivated cells live for a prolonged period, how much virus they produce and whether they die following reactivation. We have developed a novel system of “tagged” viruses to study the reactivation of individual latent cell “reactivation founders” and combine this with viral dynamics modelling to better understand HIV reactivation.

**Methods:**

Rhesus macaques were infected with 2.2 × 10^5^ IU of a modified SIVmac239 containing an SIV population with 10,000 different clonotypes differing only at a 34 bp “tag” inserted between the Vpx and Vpr genes (SIVmac239M). Animals were treated with TFV/FTC/RAL at day 6 for 82 days (n=3, group 1), or with TFV/FTC/IND/RTV on day 4 for >300 days (n=2, group 2) prior to treatment interruption. Illumina sequencing was used to identify the frequency of individual clonotypes following treatment interruption.

**Results:**

All animals showed a diversity of SIVmac239M clonotypes in serum prior to treatment, and rapid recrudescence of infection after treatment interruption. Analysis of the number and relative size of individual reactivation founder clonotypes revealed that between 3 and 63 unique clonotypes were detectable in different animals. Modelling of the ratios of the clonotypes in different animals showed that the frequency of reactivation from latency ranged from 27.3 reactivations per day to 0.46 per day. Moreover, the ratio of clonotypes also showed that the duration of viral production from individual latent after reactivation must be “burst-like” in order to produce the observed frequencies.

**Conclusions:**

The use of “tagged” SIVmac239M is an extremely powerful tool for analyzing the dynamics of HIV reactivation from latency. Analysis of the reactivating clonotypes shows that the frequency of reactivation is higher in animals treated for shorter periods of time. The rapid “burst-like” production of virus from reactivated cells suggests that these cells may be short lived following reactivation.

# Sub article: THAA0102 Excision of HIV-1 DNA by gene editing: *in vitro*, *ex vivo* and *in vivo* studies

**Published:** 2016-07-22

**Authors:** R Kaminski, W Hu, J Karn, K Khalili

**Affiliations:** 1 Lewis Katz School of Medicine, Temple University, Philadelphia, PA, United States; 2 Department of Molecular Biology and Microbiology, Case Western Reserve University, Cleveland, OH, United States

## Abstract

**Introduction:**

Cure strategy for HIV-1 infection and AIDS should include methods that directly eliminate the proviral genome from HIV-1 positive cells and/or eliminate infected cells harbouring latent virus.

**Methods:**

We modified the CRISPR/Cas9 system to enable recognition of specific DNA sequences positioned within the HIV-1 promoter spanning the 5' long-terminal repeats (LTR) and various viral genes including Gag. We applied CRISPR/Cas9 by several methods including plasmid, lentiviral and adeno-associated virus to model cells for latency, *in vitro* HIV-1 infection of CD4+ T-cells, CD4+ T-cells from HIV-1 positive patients and transgenic animals encompassing integrated copies of HIV-1 to assess efficacy of our gene editing molecule in excising a segment of HIV-1 for cells in *in vitro*, *ex vivo* and *in vivo* systems.

**Results:**

We demonstrated complete elimination of HIV-1 DNA from latently infected cells, a drastic decrease in HIV-1 replication in *in vitro* replication of PBMCs and CD4+ T-cells, suppression of HIV-1 expression in PBMCs and CD4+ T-cells for HIV-1+/AIDS patients due to InDel mutations in the viral genome and excision of viral DNA positioned between the LTR and Gag gene in tissues of HIV-1 transgenic mice upon injection of AAV-CRISPR/Cas9.

**Conclusions:**

CRISPR/Cas9 can offer an effective, precise, efficient and safe strategy for eradication of HIV-1 in several laboratory model systems and can be considered for its advancements toward clinical trials.

**Abstract THAA0102–Figure 1:** Suppression of HIV-1 replication in PBMCs and CD4+ T-cells of HIV-1 infected patients. PBMCs or CD4+ T-cells from HIV-1 infected volunteers were treated with LV-Cas9 or LV-Cas9 plus LV-gRNAs A/B. **A**. Viral DNA determined by qPCR and normalized to β-globin DNA show decrease in viral copy number with LV-gRNAs. **B**. LV-Cas9 or LV-Cas9 plus LV-gRNA A/B infected CD4+ T-cells show reduction in HIV-1 DNA copy number with LV-gRNAs. **C**. Quantitation of p24 Gag ELISA from infected cells as measure of viral replication.

# Sub article: THAA0103 CCR5 gene edited cells traffic to viral reservoir tissues and undergo SHIV-dependent positive selection in nonhuman primates

**Published:** 2016-07-22

**Authors:** CW Peterson, J Wang, P Polacino, S-L Hu, MC Holmes, H-P Kiem

**Affiliations:** 1 Fred Hutchinson Cancer Research Center, Seattle, WA, United States; 2 Sangamo BioSciences, Richmond, CA, United States; 3 University of Washington, Seattle, WA, United States

## Abstract

**Introduction:**

Gene editing of the CCR5 co-receptor locus in hematopoietic stem/progenitor cells (HSPCs) is a promising therapy for HIV infection. We have previously demonstrated the feasibility of this approach in nonhuman primates. Here, we leverage our expertise with gene editing in the pigtailed macaque (*Macaca nemestrina*) to interrogate the clonal persistence, trafficking, and antiviral efficacy of CCR5-edited cells. Our objectives were to understand how individual gene-edited HSPCs persist following autologous transplantation and virus infection, determine whether HSPC-derived, gene-edited progeny traffic to viral reservoir tissues, and develop strategies to increase the number of these cells *in vivo*.

**Methods:**

Zinc finger nucleases (ZFNs) are used to target the CCR5 locus in macaque HSPCs. Gene edited HSPCs are transplanted into animals either prior to infection with simian/human immunodeficiency virus (SHIV), or in SHIV-infected animals that are treated with a combination antiretroviral therapy (cART) regimen designed to approximate a well-suppressed HIV^+^ patient. Edited cells are measured in peripheral blood, bone marrow, gastrointestinal (GI) tract, lymph nodes, and at necropsy in a panel of 25 tissues, using methods including deep sequencing.

**Results:**

We observe up to 14-fold enrichment of CCR5 gene edited memory CD4^+^ T-cells in SHIV-infected animals, consistent with virus-dependent selection against CCR5 wt memory CD4^+^ T-cells. Gene edited cells are found in a broad array of anatomical sites. These include tissues that we have identified as viral reservoirs in our model, namely GI tract and lymph nodes. Spatial and temporal tracking of CCR5 mutations suggests that gene edited cells persist long-term, and are polyclonal. Homology directed repair (HDR) pathways can be exploited in macaque CD34^+^ HSPCs, facilitating knock-in of selectable markers at the disrupted CCR5 locus.

**Conclusions:**

Our gene editing strategy results in stable engraftment of CCR5-mutated and SHIV-resistant HSPCs and their progeny in blood, and in tissues known to serve as viral reservoirs. Importantly, gene-edited CD4^+^ T-cells undergo positive selection during active infection, further supporting the validity of this approach in the clinic. Our preliminary *ex vivo* HDR data suggest that these gene-edited cells could be engineered to undergo positive selection without the need for ongoing viral replication.

# Sub article: THAA0104LB No evidence of ongoing replication in tissue compartments during combination antiretroviral therapy

**Published:** 2016-07-22

**Authors:** G Bozzi, S Watters, FR Simonetti, E Anderson, R Gorelick, W Shao, J Bell, C Rehm, R Dewar, R Yarchoan, T Uldrick, F Maldarelli

**Affiliations:** 1 HIV Dynamics and Replication Program, NCI NIH at Frederick, Frederick, MD, United States; 2 Department of Infection and Immunity, University College London, London, United Kingdom; 3 Department of Infectious Diseases, University of Milan, L. Sacco Hospital, Milan, Italy; 4 AIDS and Cancer Virus Program, Leidos Biomedical Research, Inc., Frederick National Laboratory, Frederick, MD, United States; 5 Advanced Biomedical Computing Center, Leidos Biomedical Research, Inc., Frederick National Laboratory, Frederick, MD, United States; 6 Clinical Monitoring Research Program, Leidos Biomedical Research, Inc., Frederick National Laboratory, Frederick, MD, United States; 7 Laboratory of Immunoregulation, NIAID NIH Bethesda, Bethesda, MD, United States; 8 Frederick National Laboratory, Leidos Biomedical Research, Inc., Frederick, MD, United States; 9 HIV and AIDS Malignancy Program, NCI NIH Bethesda, Bethesda, MD, United States

## Abstract

**Introduction:**

Sources of HIV persistence during combination antiretroviral therapy (cART) remains uncertain, and the contribution of active cycles of HIV replication in tissue compartments is unknown. Genetic analyses are sensitive measures to detect ongoing cycles of virus replication, particularly in individuals who undergo cART early after infection, when HIV populations are relatively monomorphic and increases in genetic diversity are easily detectable. To investigate whether ongoing replication occurs in tissues, we studied HIV populations in blood and anatomic compartments from three individuals who initiated antiretroviral therapy shortly after HIV infection and maintained viral suppression for >8 years.

**Methods:**

Samples from three individuals in IRB-approved studies were studied. Individuals started cART soon after infection, maintained HIV RNA <50 c/mL for 8–16 years, and underwent autopsy for primary effusion lymphoma (N=1), or colonoscopy (N=2). HIV from autopsy was quantified (RT-PCR), and HIV sequences (*pro-pol*, 1200 nt) were obtained from tissues and peripheral blood lymphocytes (PBL) using single genome sequencing (SGS). Sequences were aligned, subjected to phylogenetic (MEGA) and compartmentalization (Slatkin-Maddison, FST, geographic subdivision) analyses; 263 sequences from autopsy and 293 from individuals undergoing colonoscopy were analyzed.

**Results:**

HIV DNA was detected in most tissues at autopsy (median 1.8 copies/1e6 cells, range 1–75/1e6 cells). HIV had limited genetic diversity in tissues and PBL (average pairwise difference, APD 0.3–0.6%). From the autopsy, PBL (N = 124), spleen (38), lymph node (30), ileum (30), jejunum (12), colon (5), effusion cells (10), kidney (5), lung (3), testes (1) were obtained; no HIV was recovered from frontal lobe, spinal cord, or the cell-free effusion fluid. HIV populations were well-mixed in tissues and non-divergent from PBL-derived HIV, with no evidence of compartmentalization in any tissue. Identical hypermutated sequences in PBL and several tissues demonstrated distribution of clonally expanded cells had occurred. In two individuals undergoing colonoscopy, analysis of HIV from ileum, colon, and PBL revealed no evidence of ongoing replication and no divergence from pretherapy plasma RNA obtained 12–16 years prior to colonoscopy.

**Conclusions:**

No evidence of ongoing replication was detected in tissues compared to peripheral blood in individuals undergoing cART, suggesting combination antiretroviral therapy blocks active HIV replication, including in tissues.

# Sub article: THAA0105 Allogeneic stem cell transplantation in HIV-1-infected individuals; the EPISTEM consortium

**Published:** 2016-07-22

**Authors:** AM Wensing, JL Diez-Martin, G Huetter, J Kuball, M Kwon, M Nijhuis, A Saez-Cirion, V Rocha, M Salgado, J Schulze zur Wiesch, A Stam, J Martinez-Picado

**Affiliations:** 1 Medical Center, Utrecht University, Utrecht, Netherlands; 2 Hospital General Universitario Gregorio Maranon, Madrid, Spain; 3 Cellex, Dresden, Germany; 4 Institute Pasteur, Paris, France; 5 Oxford University Hospital, Oxford, United Kingdom; 6 AIDS Research Institute IrsiCaixa, Badalona, Spain; 7 University Medical Center Hamburg-Eppendorf, Hamburg, Germany; 8 ICREA, Barcelona, Spain

## Abstract

**Introduction:**

To date, the only and most compiling evidence of a medical intervention that has been able to cure HIV-1 infection (the “Berlin patient”), involved an allogeneic stem cell transplant (SCT) from a donor who was homozygous for CCR5Δ32. Although this high-risk procedure is only indicated for certain haematological malignancies, the strategy raised tremendous scientific potential to gain insight in the mechanisms of HIV eradication.

**Methods:**

The EpiStem consortium aims to guide clinicians of HIV infected patients who require an SCT in donor search and CCR5 screening, ethical regulations, the SCT procedure, sampling procedures and in depth investigations to study HIV persistence. The patients are included in the EPISTEM observational cohort. Detailed analysis of the cohort should provide insight as to whether additional factors such as conditioning regimen, total body irradiation and graft versus host disease may contribute to the eradication of the potentially infectious viral reservoir in addition to the lack of a functional CCR5 receptor.

**Results:**

Nearly 30,000 cord blood units in multiple European blood banks and more than 1,000,000 adult donors have been genotyped for CCR5 to generate a registry of CCR5Δ32 available donors. Twenty HIV positive patients with diverse haematological malignancies have been registered to the EPISTEM cohort. Since 2012, 13 patients have been transplanted; 4 with a CCR5Δ32, 1 with a heterozygous, and 8 with a CCR5 WT donor. In 3 cases the donor cells came from cord blood and in 10 cases from an adult donor. So far, 5 patients have successfully passed the 12 months follow-up after transplantation, and 8 patients have died after transplantation, despite achieving full donor chimerism in most cases. Preliminary analysis of virological and immunological data from blood and tissue samples shows a systematic reduction of HIV-1 reservoirs to very low levels.

**Conclusions:**

EPISTEM is actively recruiting new cases and continues to systematically investigate HIV persistence over time to gain insight in potential HIV-1 eradication.

# Sub article: THAA0106LB Elimination of HIV-1 latently infected cells by PKC agonist gnidimacrin alone and in combination with a histone deacetylase inhibitor

**Published:** 2016-07-22

**Authors:** L Huang, W Lai, L Zhu, C-H Chen

**Affiliations:** Department of Surgery, Duke University Medical Center, Durham, NC, United States

## Abstract

**Introduction:**

Several classes of HIV-1 latency reversing agents (LRAs) including PKC agonists and HDAC have been investigated for their effects against latent HIV-1 infection. Although many LRAs were capable of reactivating latent HIV-1, it is less certain if they can actually reduce the latent viral reservoirs. To identify potent LRAs that can effectively eliminate/reduce latent HIV-1 reservoirs, we investigated the PKC agonist gnidimacrin (GM) alone and in combination with a selective HDAC inhibitor thiophenyl benzamide (TPB) for their effectiveness on elimination of HIV-1 latently infected cells.

**Methods:**

1) cell line model: U1 cells were treated with various concentrations of GM with/without the presence of TPB. The culture supernatant was then collected for P24 ELISA to quantify latent HIV-1 activation; 2) *ex vivo* model: PBMCs from HIV+ patients (with >5 years of ART and undetectable viral load) were treated with GM, GM + TPB, or vorinostat in the presence of anti-retroviral agents. The viral DNA in the lately infected cells was quantified with RT-PCR, and the frequency of latently infected cells was determined by using a limiting dilution viral outgrowth assay.

**Results:**

In U1 model, the EC_50_ of GM was 18 pM for latent viral activation and its potency was enhanced about three-fold in the presence of TPB. In *ex vivo* model, GM alone (20 pM) was able to reduce pro-viral DNA and the frequency of latently infected cells by 5–10 folds. Addition of TPB further increased the effect of GM by over three-fold.

**Conclusions:**

The results of this study demonstrate that GM is an extremely potent LRA that can effectively reduce HIV-1 latently infected cells *ex vivo* at pM concentrations. In the presence of TPB, the effect of GM was further enhanced. TPB exhibited good selectivity with no overlapping cytotoxic and effective doses in contrast to other tested HDACIs. Moreover, TPB may antagonize the potential side effects of GM by inhibiting high dose GM-induced inflammatory cytokine production. Thus, combination of GM and TPB provides a unique and effective option in reducing the latent HIV-1 reservoir.

# Sub article: THAA0201 Novel conserved element HIV/SIV DNA vaccines maximize breadth and magnitude of immune response

**Published:** 2016-07-22

**Authors:** BK Felber, X Hu, A Valentin, F Dayton, Y Cai, M Rosati, C Alicea, NY Sardesai, R Gautam, MA Martin, JI Mullins, GN Pavlakis

**Affiliations:** 1 Vaccine Branch, Center for Cancer Research, National Cancer Institute at Frederick, Frederick, United States; 2 Inovio Pharmaceuticals, Inc., Plymouth Meeting, PA, United States; 3 National Institute of Allergy and Infectious Diseases, NIH, Bethesda, MD, United States; 4 University of Washington, Seattle, WA, United States

## Abstract

**Introduction:**

HIV sequence diversity and potential “decoy” epitopes are hurdles in the development of an effective AIDS vaccine. To target immune responses towards invariable viral regions, we engineered DNA-based immunogens encoding conserved elements (CE) of HIV-1 selected on the basis of stringent conservation, functional importance, broad HLA-coverage, and association with viral control.

**Methods:**

DNA vectors were generated expressing seven collinearly arranged CE from p24^gag^ to cover >98% of group M sequences. By analogy, a similar vaccine was developed against SIV Gag. Heterologous DNA regimens consisting of CE prime were followed by a boost with DNA expressing either full-length Gag or a combination of CE+full-length Gag. Immune responses were evaluated in macaques vaccinated by IM/electroporation.

**Results:**

All HIV and SIV CE DNA-vaccinated macaques developed robust CE-specific memory responses with a significant fraction of cytotoxic T cells. In contrast, vaccination with HIV or SIV full-length *gag* DNA was very inefficient in inducing CE responses (50% responders; fewer CE recognized). Subsequent *gag* DNA vaccination significantly boosted the preexisting CE responses. Interestingly, vaccination with a combination of CE+*gag* DNA efficiently increased the breadth of preexisting CE responses, indicating a significant change in epitope hierarchy both for the HIV and SIV vaccine regimens. The induced T cell responses rapidly disseminated into secondary lymphoid organs and effector mucosal sites. CE responses were maintained for >2 years. A single booster vaccination with CE DNA resulted in rapid increase of pre-existing responses reaching up to ~7% of total T cells.

**Conclusions:**

Priming with CE DNA is critical to induce broad responses to vulnerable sites of the virus while avoiding variable or decoy targets that may divert effective T cell responses towards less protective viral determinants. Combination of CE and full-length immunogens provides a novel strategy to increase the breadth and magnitude of cellular and humoral immunity. This strategy allows for the development of robust T cell responses targeting a broad number of epitopes, including subdominant conserved viral epitopes. The expanded breadth of the responses could provide an advantage in restricting viral propagation. This vaccine regimen is currently under development for testing in an HVTN clinical trial.

# Sub article: THAA0202 CD8^+^ T cell breadth and *ex vivo* virus inhibition capacity distinguish between viremic controllers with and without protective HLA class I alleles

**Published:** 2016-07-22

**Authors:** CK Koofhethile, Z Ndhlovu, C Thobakgale, J Prado, N Ismail, Z Mncube, L Mkhize, M van der Stok, N Yende, BD Walker, PJR Goulder, T Ndung'u, Sinikithemba Cohort

**Affiliations:** 1 HIV Pathogenesis Programme (HPP), University of KwaZulu-Natal, Durban, South Africa; 2 Ragon Institute of Massachusetts General Hospital, Massachusetts Institute of Technology and Harvard University, Boston, MA, United States; 3 AIDS Research Institute IrsiCaixa, Hospital Universitari Germans TriasiPujol, Barcelona, Spain; 4 Centre for the AIDS Programme of Research in South Africa (CAPRISA), University of KwaZulu-Natal, Durban, South Africa; 5 Department of Paediatrics, University of Oxford, Oxford, United Kingdom; 6 University of KwaZulu-Natal, KwaZulu-Natal Research Institute for Tuberculosis and HIV (K-RITH), Durban, South Africa

## Abstract

**Introduction:**

The mechanisms of viral control and loss of viral control in chronically infected individuals with or without protective HLA class I alleles are not fully understood. We characterized longitudinally the immunological and virological features that may explain divergence in disease outcome in 70 HIV-1 C-clade infected antiretroviral therapy (ART)-naïve South African adults, 35 of whom possessed protective HLA class I alleles.

**Methods:**

Viral loads, CD4 counts, HLA typing and sequencing were performed by standard molecular methods. HLAs A*74:01, B*57, B*58:01, B*81:01 were considered protective. HIV-specific CD8^+^ T-cell immune responses were quantified using the interferon gamma (IFN-g) enzyme-linked immunosorbent spot (ELISPOT) assay after stimulation with overlapping peptides (OLP) and HLA-specific optimal peptides derived from the C clade HIV consensus sequence covering the whole proteome. T cell polyfunctionality and proliferation upon stimulation with Gag peptide pool was assessed by flow cytometry and CFSE assay, respectively. Virus inhibition was assessed by an *ex vivo* co-culture assay.

**Results:**

Over 5 years of longitudinal follow-up, 35% of individuals with protective HLA class I alleles lost viral control compared to none of the individuals without protective HLA class I alleles (p=0.06). Sustained HIV-1 control in patients with protective HLA class I alleles was associated with breadth of HIV-1 CD8^+^ T-cell responses against Gag and enhanced ability of CD8^+^ T cells to suppress viral replication *ex vivo*. In some cases loss of virological control was associated with reduction in both total breadth of CD8^+^ T cell responses and the ability of CD8^+^ T cells to suppress viral replication *ex vivo*, in the absence of differences in HIV-1-specific CD8^+^ T cell polyfunctionality or proliferation. In contrast, viremic controllers without protective HLA class I alleles possessed low breadth of HIV-1-specific CD8^+^ T cell responses characterized by reduced ability to suppress viral replication *ex vivo*.

**Conclusions:**

These data suggest that the control of HIV-1 in individuals with protective HLA class I alleles may be driven by broad CD8^+^ T cell responses with potent viral inhibitory capacity while control among individuals without protective HLA class I alleles may be mediated by CD8^+^ T cell independent mechanisms.

# Sub article: THAA0203 Neutrophils mediate potent and rapid anti-HIV antibody-dependent functions

**Published:** 2016-07-22

**Authors:** MJ Worley, AD Kelleher, SJ Kent, AW Chung

**Affiliations:** 1 Department of Microbiology and Immunology, University of Melbourne, Melbourne, VIC, Australia; 2 The Kirby Institute, The University of New South Wales, Sydney, NSW, Australia; 3 Immunovirology Laboratory, St. Vincent's Centre for Applied Medical Research, Sydney, NSW, Australia; 4 Melbourne Sexual Health Centre, Monash University, Melbourne, VIC, Australia; 5 Department of Infectious Diseases, Monash University, Melbourne, VIC, Australia

## Abstract

**Introduction:**

Functional antibodies have been shown to mediate innate immune effector responses such as antibody dependent cellular cytotoxicity (ADCC) and their importance in HIV protection was highlighted by the RV144 HIV vaccine trial. However, the majority of studies have focused on antibody activation of NK cells and monocytes, while neutrophils remain understudied. Neutrophils are of particular importance as they are abundantly present in the mucosal sites of HIV transmission, constitute 40–70% of white blood cells and can respond rapidly to stimuli. We investigated the repertoire of antibody dependent functions in HIV infected patients and examined any differences between long-term slow progressors (LTSP) and progressors.

**Methods:**

Neutrophils, PBMC, monocytes and NK cells were isolated from healthy donors and were evaluated for ADCC killing kinetics utilizing HIV-specific antibodies. In addition, antibody-dependent phagocytosis (ADP) and ADCC activity of purified plasma IgG from 33 HIV positive subjects not on antiretroviral therapy was evaluated using healthy donor neutrophils. Nineteen subjects were LTSP, who maintained CD4 T-cell >450 cells/µl (median 656/µl) for over 7 years after infection, while 14 were HIV progressors who progressed to treatment within 6 years of infection (median CD4 T-cell 478/µl) at the time of sample collection.

**Results:**

Neutrophils readily mediated HIV-specific ADCC and ADP and significant correlations between the functions were observed for LTSP R_s_=0.626 (p=0.004) and progressors R_s_=0.653 (p=0.014). Neutrophils mediated potent ADCC activity, having two-fold greater responses than NK cells, enhanced killing capacity compared to PBMC's, and induced similar responses to monocytes. Neutrophils required a higher concentration of HIV IgG to reach their maximal ADCC activity compared to NK cells, PBMC and monocytes. Similar levels of ADP and ADCC were observed in neutrophils between the LTSP and progressors patient groups.

**Conclusions:**

HIV-specific IgG can activate neutrophils to mediate ADP and ADCC against HIV-1 envelope protein. As both of these functions are highly correlated, the same Fc receptors/antibodies may be utilized to mediate these responses. Neutrophils may require more Fc receptor/antibody interactions for activation. The rapid action and high magnitude of ADCC by neutrophils highlights their potential importance early in HIV infections.

# Sub article: THAA0204 Impact of antibody isotype and association constant rate on antiviral functions against HIV-1

**Published:** 2016-07-22

**Authors:** SG Okala, D King, R Shattock

**Affiliations:** 1 Faculty of Medicine, Imperial College London, London, United Kingdom; 2 International AIDS Vaccine Initiative, London, United Kingdom

## Abstract

**Introduction:**

Both IgG and IgA are found at mucosal surfaces where HIV-1 enters the body in over 90% of the cases. However, few studies have investigated whether IgG and IgA recognizing the same epitopes presented different antiviral abilities that could prevent HIV-1 infection. Here, we compared the antiviral properties of IgG and IgA in various assays and examined the relationships between kinetics parameters of antibody-virions interactions and antiviral functions.

**Methods:**

A panel of 12 neutralizing monoclonal antibodies (mAbs) including IgG, monomeric (m)IgA and dimeric (d)IgA were compared for their ability to bind, capture, mediate viral aggregation, neutralize and inhibit HIV-1 virions transcytosis across epithelial cells. Kinetic parameters of mAbs and HIV-1_Bal_ gp140 interactions were measured using Bio-Layer Interferometry (BLI), HIV-1_Bal_ captured by the panel of mAbs was determined by ELISA, antibody mediated viral aggregation (AMVA) was detected using Nanoparticle Tracking Analysis (NTA), neutralization was assessed by TZM-bl assay and inhibition of viral transcytosis through a monolayer of HEC-1A cells was quantified by real-time reverse transcription polymerase chain reaction (RT-PCR).

**Results:**

We showed previously that the association constant rate (K_on_) correlates with antibody ability to capture viral particles, with IgG binding more quickly to its target and capturing more HIV-1 virions than its mIgA and dIgA counterparts with the same epitope specificity. In this study, we demonstrated that not only the K_on_ of neutralizing mAbs significantly correlates with viral capture but also with neutralization (p<0.01). Additionally, we found a significant relationship between the proportions of virions captured and the antibody neutralization activity (p<0.01). However, no relationship was observed between K_on_ and AMVA or between K_on_ and inhibition of viral transcytosis. Interestingly, dIgAs were able to induce viral aggregates and to inhibit HIV-1 transcytosis while IgGs and mIgAs recognizing the same epitopes failed to show these functions.

**Conclusions:**

Thus, this work implies that the association constant rates of mAbs might be used as an indicator of antibody ability to capture viral particles and to neutralize infectious virions. Moreover, it shows that antibody isotype influences antiviral functions and provided insights for the selection of potent antibody against HIV-1.

# Sub article: THAA0205 T regulatory cell depletion in controller macaques reactivates SIV and boosts CTLs

**Published:** 2016-07-22

**Authors:** T He, B Policicchio, E Brocca-Cofano, J Stock, C Xu, K Raehtz, T Gaufin, R Gautam, I Pandrea, C Apetrei

**Affiliations:** 1 Center for Vaccine Research, University of Pittsburgh, Pittsburgh, United States; 2 Tulane National Primate Research Center, Tulane University, Covington, United States

## Abstract

**Introduction:**

T regulatory cells (Tregs) may be involved in formation of latent reservoir, being susceptible to HIV/SIV infection. Resting Tregs harbour high levels of HIV/SIV. During acute infection, Tregs decisively contribute to the establishment of HIV reservoir by reversing CD4+ T cell immune activation status. During chronic infection, they contribute to the impairment of CTL responses, as Treg expansion correlates with loss of CTL function and their *ex vivo* depletion enhances T cell responses to HIV/SIV antigens. HLAB27+ and B57+ HIV-specific CD8+ T cells from elite controllers evade Treg suppression. We hypothesized that Treg depletion is a valid approach for HIV cure, in which a single intervention reduces the size of the reservoir, reactivates the virus and boosts cell-mediated immune responses.

**Methods:**

Five SIVsab-infected rhesus macaques (RMs), in which spontaneous supercontrol of virus replication (<3 copies/ml plasma) associates complete control of immune activation, were depleted of Tregs with Ontak (Denileukin diftitox), an engineered protein combining IL-2 and diphtheria toxin. Treg depletion was monitored by flow cytometry and immunohistochemistry; plasma viral load was measured by single copy assay; specific cellular immune responses to SIV antigens were monitored flow cytometrically by intracellular cytokine staining after stimulation with SIVsab peptides.

**Results:**

Ontak administration to SIVsab-infected RMs resulted in significant depletion (>75%) of the circulating Fox-P3+CD25+CD4+ T cells. Up to 60% and >50% of Tregs were depleted from gut and the lymph nodes, respectively. Ontak impact on overall CD8+ T cell counts was minimal. Treg depletion resulted in a major increase of the levels of CD4+ T cell activation (Ki-67). In the absence of antiretroviral therapy, virus rebound to 103 vRNA copies/ml of plasma occurred after Ontak administration. Importantly, Treg depletion resulted in a significant boost of the SIV-specific CD8+ T cells and rapid clearance of the reactivated virus.

**Conclusions:**

Treg depletion in chronically SIV-infected superelite controller RMs resulted in both reactivation of latent virus and a boost of CTL responses. The overall Treg ability to control immune responses was significantly impaired despite the fact that Treg depletion was incomplete. As no latency reversing agent in development has such a dual activity, our strategy holds great promises for cure research.

# Sub article: THAA0206 PD-1 blockade synergizes with ART for restoring anti-viral CD8 T cell function and possibly destabilizing the viral reservoir in SIV infected macaques

**Published:** 2016-07-22

**Authors:** G Mylvaganam, S Hicks, B Lawson, M Nega, V Velu, R Ahmed, G Freeman, RR Amara

**Affiliations:** 1 Yerkes National Primate Research Center, Emory University, Atlanta, GA, United States; 2 Harvard Medical School, Boston, MA, United States

## Abstract

**Introduction:**

The expression of the inhibitory receptor programmed death-1 (PD-1) on anti-viral CD8 T cells and virally infected CD4 T cells provides an immunological signature for both T cell dysfunction and viral latency during chronic SIV/HIV infection. We hypothesized that PD-1 blockade administered during the initiation of anti-retroviral therapy (ART) and under fully suppressive ART would have direct effects on both dysfunctional CD8 T cells and latently infected CD4 T cells. To test our hypothesis we developed a primatized anti-human PD-1 Ab to allow for repeated infusions in rhesus macaques (RMs) and administered PD-1 blockade to chronically SIV infected RMs in combination with ART.

**Methods:**

SIVmac251 infected RMs were administered 5 infusions (over 14 days) of a 3 mg/kg dose of primatized anti-PD-1 Ab 10 days prior to the initiation of ART. About 8 months post ART, RMs received 3 monthly infusions of 10 mg/kg anti-PD-1 or saline. ART was interrupted at 2 weeks after the final PD-1 Ab infusion.

**Results:**

PD-1 blockade administered during the initiation of ART enhanced proliferation of anti-viral CD8 T cells (p=0.02), increased their cytotoxic potential (p=0.04) and polyfunctionality (p=0.01). Importantly, the PD-1 Ab treated animals showed more rapid viral suppression (42 days in the PD-1 group vs. 140 days in saline group; p=0.01) and greater reconstitution of Th17 cells in the rectal mucosa (p=0.01) following initiation of ART. Moreover, PD-1 blockade administered under suppressive ART resulted in transient but significant increases in viremia, suggesting possible effects on destabilizing the latent viral reservoir. Following ART interruption, PD-1 Ab treated animals showed up to 80-fold reduction in set point viremia compared to set point levels prior to initiation of ART.

**Conclusions:**

These results reveal for the first time the potential of PD-1 blockade both on restoring anti-viral CD8 T cell function and possibly destabilizing the viral reservoir under ART. They highlight the potential of PD-1 blockade to work synergistically with other therapeutic agents such as vaccines and latency reversing agents to effectively diminish HIV reservoir under ART as a means to establish a functional cure.

# Sub article: THAB0101 Extended ART initiation criteria can be implemented successfully in rural South Africa

**Published:** 2016-07-22

**Authors:** SJ Steele, G Arellano, T Ellman, A Shroufi, G Van Cutsem

**Affiliations:** 1 Médecins Sans Frontières, Cape Town, South Africa; 2 Médecins Sans Frontières, Eshowe, South Africa; 3 SAMU, Medecins Sans Frontieres, Cape Town, South Africa

## Abstract

**Introduction:**

There is concern that earlier initiation of antiretroviral treatment (ART) in lower resource settings may compromise access to care for patients with lower CD4 counts, and that patients with higher CD4 counts may have lower retention in care (RIC). In July 2014, we extended ART initiation criteria from CD4 cell counts of ≤350 to ≤500 copies/µl in nine primary health clinics in KwaZulu-Natal, South Africa. Here we assess whether any compensatory reduction in initiation of sicker patients was seen and whether retention among those newly eligible was satisfactory in this public sector setting.

**Methods:**

In this retrospective cohort analysis, we compare proportions initiated on ART and RIC at 6 months among patients with baseline CD4 taken between 1 July and 31 December 2014 (CD4≤500 eligibility cohort) and between 1 July and 31 December 2013 (CD4≤350 eligibility cohort). Pregnancy, TB, age <15 years and WHO stage 3 or 4 were exclusion criteria. Outcomes were determined from baseline CD4 and analyzed using survival analysis.

**Results:**

There were 768 patients in the CD4≤350 eligibility cohort, with 31% having baseline CD4≤200; 51% 201–350, and; 12% 351–500. Of the 856 in the CD4≤500 eligibility cohort, 23% had a baseline CD4≤200, 37% 201–350 and 33% 350–500. In both cohorts, median age was 31 years and 67% were female. Among participants with CD4 351–500, percentage initiated on ART within 3 months increased 10-fold between the periods from 7% (95%CI: 3.4–13.0) to 70% (95% CI: 61–78); among those with CD4≤200 this increased from 70% (95% CI: 55–80) to 86% (95% CI: 79–93). The proportion initiated within 3 months among those with baseline CD4 201–350 remained unchanged at approximately 75%. RIC at 6 months was 82% (95% CI: 79–85%) in the CD4≤500 cohort and 80% (95% CI: 76–84%) in the CD4≤350 cohort.

**Conclusions:**

Expanding eligibility for ART to CD4≤500 resulted in rapid change in time to ART initiation among those with baseline CD4 351–500 without compromising initiation or RIC among those with a CD4≤350. Extended initiation criteria can be successfully implemented in high HIV prevalence, low resources-settings without compromising access to care for more vulnerable patients.

# Sub article: THAB0102 Immediate HIV treatment prevents new infections: causal evidence on the real-world impact of immediate versus deferred ART in rural South Africa

**Published:** 2016-07-22

**Authors:** C Oldenburg, J Bor, F Tanser, T Mutevedzi, M Shahmanesh, G Seage, V De Gruttola, M Mimiaga, K Mayer, D Pillay, T Bärnighausen

**Affiliations:** 1 Department of Epidemiology, Harvard T.H. Chan School of Public Health, Boston, MA, United States; 2 Department of Global Health, Boston University School of Public Health, Boston, MA, United States; 3 Africa Centre for Population Health, Mtubatuba, South Africa; 4 University of KwaZulu Natal, Durban, South Africa; 5 University College London, London, United Kingdom; 6 Department of Biostatistics, Harvard T.H. Chan School of Public Health, Harvard University, Boston, MA, United States; 7 Brown University School of Public Health, Providence, RI, United States; 8 Fenway Community Health, Boston, MA, United States; 9 Beth Israel Deaconess Medical Center, Boston, MA, United States; 10 Department of Global Health and Population, Harvard T.H. Chan School of Public Health, Harvard University, Boston, MA, United States

## Abstract

**Introduction:**

Immediate initiation of antiretroviral therapy (ART) reduces HIV transmission in serodiscordant couples in randomized controlled trials. However, the effect of immediate ART under real-life conditions is not well characterized. We investigated the effect of immediate ART eligibility on HIV incidence among HIV-uninfected household members using a large population-based longitudinal cohort in KwaZulu-Natal, South Africa. We use a quasi-experimental regression discontinuity design to estimate causal effects using the CD4 count-based threshold rule for ART initiation.

**Methods:**

Households members of patients seeking care at the Hlabisa HIV Treatment and Care Programme between January 2007 and August 2011 with CD4 counts up to 350 cells/µl were eligible for inclusion if they had at least two HIV tests and were HIV-uninfected at the time the index patient linked to care (N=4115). A regression discontinuity design was used to assess the intention-to-treat effect of immediate versus delayed ART eligibility on HIV incidence among household members. Exploiting the CD4-count based threshold rule for ART initiation (CD4<200 cells/µl until August 2011), we used Cox proportional hazards models to compare outcomes for household members of patients who presented for care immediately above versus immediately below the threshold.

**Results:**

Characteristics of household members of index patients initiating HIV care were balanced between those with an index patient immediately eligible for ART (N=2489) versus delayed for ART (N=1626). In the immediate group, median age was 20 years (IQR 16–48) and 61.4% were female, compared to median age 20 years (IQR: 16–47) and 62.4% female in the delayed group. Seventy-eight percent of index household members immediately eligible for ART initiated ART within 6 months of initiating care, compared to 22.3% of those delayed for ART. Overall HIV incidence among household members was 2.8 infections per 100 person-years (95% CI 2.5–3.1). Immediate eligibility for treatment resulted in a 45% decrease in HIV incidence in households (HR=0.55, 95% CI: 0.35–0.87).

**Conclusions:**

Outside of a tightly-controlled clinical trial setting, we demonstrate substantial reductions in household-level HIV incidence with immediate eligibility for ART. The benefit of ART uptake may extend outside of couples into spillover effects in households.

# Sub article: THAB0103LB Randomized trial of stopping or continuing ART among post-partum women with pre-ART CD4 >400 cells/mm^3^ (PROMISE 1077HS)

**Published:** 2016-07-22

**Authors:** J Currier, P Britto, R Hoffman, S Brummel, G Masheto, E Joao, B Santos, L Aurpibul, M Losso, MF Pierre, A Weinberg, N Chakhtoura, R Browning, A Coletti, D Shapiro, J Pilotto, PROMISE 1077HS Team

**Affiliations:** 1 Department of Medicine, UCLA, Los Angeles, CA, United States; 2 Center for Biostatistics in AIDS Research, T.H. Chan School of Public Health, Harvard University, Boston, MA, United States; 3 Division of Infectious Diseases, University of California, Los Angeles, Los Angeles, CA, United States; 4 Harvard School of Public Health, Boston, MA, United States; 5 Botswana Harvard AIDS Partnership, Gabarone, Botswana; 6 Hospital Servitors do Estado, Rio de Janeiro, Brazil; 7 Hospital Conceicao, Porto Alegre, Brazil; 8 RIHES, Chiang Mai University, Chiang Mai, Thailand; 9 HIV Unit, Hospital J.M. Ramos Meija, Buenos Aires, Argentina; 10 Centres GHESKIO, Port-au-Prince, Haiti; 11 University of Colorado Denver, CO, United States; 12 NICHD, Washington, DC, United States; 13 NIAID, NIH, Washington, DC, United States; 14 FHI360, Durham, NC, United States; 15 Fiocruz, Rio de Janeiro, Brazil

## Abstract

**Introduction:**

Health benefits of post-partum antiretroviral therapy (ART) for women with high CD4 counts have not been assessed in randomized trials.

**Methods:**

Asymptomatic, HIV+, non-breastfeeding women with pre-ART CD4 cell counts ≥ 400 cells/mm^3^ started on ART during pregnancy were randomized up to 42 days after delivery to continue or discontinue ART. LPV/RTV + TDF/FTC was the preferred ART regimen. The primary composite endpoint included death, AIDS-defining illness and serious non-AIDS events. The sample size was selected to provide 88% power to detect a 50% reduction from an annualized primary event rate of 2.07%. A post-hoc analysis evaluated WHO Stage 2 and 3 events. All analyses were intent to treat.

**Results:**

A total of 1652 women from 52 sites in Argentina, Botswana, Brazil, China, Haiti, Peru, Thailand and the US were enrolled (1/2010–11/2014). Median age was 28 years and major racial categories were Black African (28%), Thai (16%) and White (15%). Median entry CD4 count was 696 cells/mm^3^, median ART exposure prior to delivery was 19 weeks (IQR 13–24) and 94% had entry HIV-1 RNA <1000 copies/mL. After a median follow-up of 2.3 years, the primary composite endpoint rate was 0.34%, significantly lower than expected and not significantly different between arms. WHO Stage 2 and 3 events were reduced with continued ART (Table). Toxicity rates were higher in the continue arm but the difference was not statistically significant. Among women randomized to continue ART, 189/827 (23%) had virologic failure. Of the 155 with resistance testing, 134 (86%) failed without resistance to their current regimen, suggesting non-adherence.

**Conclusions:**

In the largest randomized trial to date evaluating post-partum ART, serious clinical events were rare among young women with high CD4 cell counts. Continued ART was safe and was associated with reductions in WHO 2/3 conditions. Virologic failure rates were high, underscoring the need to improve adherence in this population.

**Abstract THAB0103LB–Table 1:** Study endpoint table

Endpoint (time to first event)	Continue ART no. no./100 pyr		Discontinue ART no./100 pyr		Hazard Ratio	p-value
Primary End point	6	0.31	7	0.36	0.87 (0.29, 2.59)	0.80
AIDS Defining (WHO 4)	2	0.10	3	0.15	0.67 (0.11, 4.02)	0.66
Serious Non-AIDS (Renal)	2	0.10	1	0.05	2.00 (0.18, 22.01)	0.56
Death	2	0.10	4	0.20	0.52 (0.09, 2.81)	0.44
Secondary End points						
Composite Endpoint of HIV/AIDS related events and WHO stage 2–3 events	57	3.09	100	5.55	0.56 (0.40, 0.77)	≤0.001
WHO 2–3 events	38	2.02	80	4.36	0.47 (0.32, 0.68)	≤0.001
Grade 2 Toxicity and above	260	18.4	232	15.4	1.17 (0.98, 1.40)	0.08
Grade 3 and 4 Toxicity	188	12.0	160	9.8	1.21 (0.98, 1.50)	0.07

# Sub article: THAB0104 SALIF trial: switching suppressed first-line patients to tenofovir/emtricitabine/rilpivirine (TDF/FTC/RPV) is non-inferior to TDF/FTC/efavirenz (TDF/FTC/EFV) and could be an alternative treatment option in LMICs

**Published:** 2016-07-22

**Authors:** P Munderi, E Were, A Avihingsanon, P Abena Messomo Mbida, L Mohapi, BM Samba, M Jansen, P Mohammed, G Sawyerr, P Barnes, Y van Delft

**Affiliations:** 1 MRC/UVRI Uganda Research Unit on AIDS, Entebbe, Uganda; 2 Partners in Prevention, Eldoret, Kenya; 3 HIV-NAT The Netherlands Australia Thailand Research Collaboration, Bangkok, Thailand; 4 Cabinet Medical IDOC, Douala, Cameroon; 5 Perinatal HIV Research Unit, University of the Witwatersrand, Johannesburg, South Africa; 6 District Centre de Gaspard Kamara, Dakar, Senegal; 7 Global Clinical Development Operations, Janssen, Tilburg, Netherlands; 8 Global Public Health, Janssen, High Wycombe, United Kingdom; 9 Research & Development LLC, Janssen, Titusville, NJ, United States; 10 Global Public Health, Janssen, Tilburg, Netherlands

## Abstract

**Introduction:**

Current global antiretroviral therapy (ART) guidelines recommend immediate initiation of ART and thus an increasing number of HIV patients are becoming eligible for treatment. There is a pressing need for simple, efficacious, well tolerated, affordable ART regimens. Rilpivirine (RPV) has a good safety profile and is a well-tolerated non-nucleoside reverse transcriptase inhibitor (NNRTI) whose low recommended once-daily dosing makes it a good candidate component for an affordable fixed-dose-combination (FDC) in low- and middle- income countries (LMIC).

**Methods:**

SALIF (***S**witching **A**t **L**ow HIV-1 RNA **I**nto **F**ixed Dose Combinations*) was a 48 week, multicentre, phase 3b, randomized, open-label clinical study, designed to demonstrate non-inferiority of TDF/FTC/RPV to TDF/FTC/EFV (both as FDC) in maintaining HIV-1 RNA suppression (VL<400 copies/ml) among adults currently on first-line NNRTI-based (EFV or nevirapine) ART with HIV-1 RNA <50 copies/ml. The study was conducted in Cameroon, Kenya, Senegal, South Africa, Thailand and Uganda from 08/2013 to 10/2015.

**Results:**

A total of 424 individuals were included in the intention to treat (ITT) analysis (64.3% women, median age: 41 years, mean CD4: 547 cells/mm^3^, median time on ART: 5.2 years). Virological suppression (<400 copies/ml) after 48 weeks was maintained in 200/213 (93.9%) individuals switched to TDF/FTC/RPV and in 203/211 (96.2%) on TDF/FTC/EFV (FDA Snapshot difference −2.3%; 95% CI −6.4, 1.8); results were identical using a cut-off of <50 copies/ml. Virological failure (≥400 copies/ml) was observed in one individual in each arm, without the development of drug-resistance associated mutations. High adherence rates (>95% adherence based on tablet count) were documented in 95.8% (TDF/FTC/RPV) and 97.6% (TDF/FTC/EFV). In the TDF/FTC/RPV arm 17 individuals (8.0%) discontinued assigned treatment versus 10 (4.7%) in the TDF/FTC/EFV arm. Most frequent reasons were: adverse events (3.8% vs. 0.5%), loss to follow-up (0.9% vs. 1.4%) and subject withdrawal (0.9% vs. 1.4%).

**Conclusions:**

In adults with suppressed viral load on first-line NNRTI-based ART in a LMIC setting, switching to a FDC of TDF/FTC/RPV was non-inferior to TDF/FTC/EFV in maintaining high rates of viral suppression with a comparable tolerability profile.

**Abstract THAB0104–Table 1:** Safety overview

Number of individuals, n (%)	TDF/FTC/RPV (n=213)	TDF/FTC/EFV (n=211)	All Subjects (n=424)
SAEs	16 (7.5%)	11 (5.2%)	27 (6.4%)
At least one DAIDS grade 3 or 4 AE	40 (18.8%)	56 (26.5%)	96 (22.6%)
Fatal AEs	1 (myocardial infarction; unrelated to study medication)	0	1
Treatment limiting AEs	8 (3.8%)	1 (0.5%)	9 (2.1%)
At least one diabetes/hyperglycaemia event of interest	10 (4.7%)	4 (1.9%)	14 (3.3%)
At least one neuropsychiatric event of interest	60 (28.2%)	63 (29.9%)	123 (29.0%)

# Sub article: THAB0105 Virological outcomes of patients on second-line ART (boosted atazanavir versus boosted lopinavir)

**Published:** 2016-07-22

**Authors:** E Laker Agnes Odongpiny, D Nalwanga, A Kaimal, A Kiragga, SM Nabaggala, B Castelnuovo, R Parkes-Ratanshi

**Affiliations:** 1 Infectious Diseases Institute, Makerere College of Health Sciences, Kampala, Uganda; 2 Infectious Diseases, Institute of Public Health, University of Cambridge, London, United Kingdom

## Abstract

**Introduction:**

Since 2010, the WHO guidelines recommend the use of boosted Atazanavir (ATV/r) as a preferred protease inhibitor (PI) alongside boosted Lopinavir (LPV/r) in patients who have failed first line antiretroviral therapy (ART) in sub-Saharan Africa. However, there are no RCTs or observational studies comparing virological outcomes of ATV/r to LPV/r in sub Saharan Africa.

**Methods:**

This is a retrospective analysis of patients who were initiated on a standard second line therapy of 2NRTIs plus LPV/r or ATV/r between 1 January 2010 and 1 December 2014 in a large urban clinic in Uganda. Viral load (VL) monitoring has been made available in 2014; therefore most patients had a VL test done after this. We compared baseline characteristics of patients started on ATV/r and LPV/r and performed logistic regression analysis to determine factors associated with viral failure (VL>400 copies/ml).

**Results:**

A total of 285 patients begun ATV/r regimen versus 215 on LPV/r.Baseline characteristics for the 2 groups as in the table were not significantly different except median duration on first line. VLs were available for 230 (80.7%) patients on ATV/r and 173 (80.5%) on LPV/r. A total of 205 (87.6%) patients on ATV/r and 136 (80.5%) on LPV/r had a VL<400 copies/ml (p=0.050).Using logistic regression the following baseline characteristics at start of second line were not associated with the virological outcome: gender (OR=0.658, 95%CI: 0.343, 1.265), current second line regimen group (OR=0.475, 95% CI: 0.204, 1.104), BMI (OR=0.997, 95% CI: 0.992, 1.003), age (OR=0.999, 95% CI: 0.97, 1.04), CD4 cell count (OR=0.999, 95% CI: 0.996, 1.001), duration on second line ART (OR=1.001, 95% CI: 0.985, 1.029). However, there was trend towards likelihood of VL>400 copies/ml if duration on first line was shorter (OR=1.015, 95% CI: 1.003, 1.027).

**Conclusions:**

This study showed a generally high level of viral suppression between patient on second line regimens containing ATV/r or LPV/r.We did not find statistical differences in virological outcome of patients started on LPV/r as compared to ATV/r.

**Abstract THAB0105–Table 1:** Baseline Characteristics of patients at time of switch to second line regimen

Characteristic	ATV/r n=285	LTV/r n=215	p
Age in years, Mean (SD)	37 (9.0)	37 (9.2)	0.4979
Sex, n (%)			
Female	187 (65.6)	135 (62.8)	0.514
Male	98 (34.4)	80 (37.2)	
BMI, Median (IQR)	22.3 (19.7–54.4)	21.7 (19.3–24.5)	0.1554
Time (months) on first line therapy, Median (IQR)	39 (22–65)	40 (14–64)	0.0150
CD4 count cell/µl, Median (IQR)	109 (53–202)	114 (60–206)	0.5022
Viral load copies/mm^3^, Median (IQR)	4.8 (4.3–5.2)	4.9 (4.3–5.4)	0.2501

# Sub article: THAB0106LB Low acceptance of early antiretroviral therapy among post-partum women enrolled in IMPAACT PROMISE studies across the globe

**Published:** 2016-07-22

**Authors:** L Stranix-Chibanda, S Brummel, K Angelidou, C Tierney, A Coletti, K McCarthy, J Pilotto, M Mutambanengwe, V Chanaiwa, T Mhembere, M Kamateeka, G Masheto, R Chamanga, M Maluwa, S Hanley, E Joao, G Theron, A Chandawale, M Nyathi, B Santos, L Aurpibul, M Mubiana-Mbewe, R Oliveira, M Basar, N Chakhtoura, R Browning, J Currier, MG Fowler, PROMISE Study Team

**Affiliations:** 1 College of Health Sciences, Paediatrics and Child Health, University of Zimbabwe, Harare, Zimbabwe; 2 Department of Biostatistics, Center for Biostatistics in AIDS Research, Harvard T.H. Chan School of Public Health, Boston, MA, United States; 3 FHI 360, IMPAACT Operations Center, Durham, NC, United States; 4 Hospital Geral de Nova Iguacu, Laboratorio de AIDS e Imunologia Molecular – Fiocruz, Rio de Janeiro, Brazil; 5 College of Health Sciences, University of Zimbabwe – University of California San Francisco Collaborative Research Programme, Harare, Zimbabwe; 6 Makerere University – Johns Hopkins University Research Collaboration, Kampala, Uganda; 7 Harvard T.H. Chan School of Public Health, Botswana Harvard AIDS Institute Partnership, Gaborone, Botswana; 8 College of Medicine – Johns Hopkins Research Project, Blantyre, Malawi; 9 University of North Carolina Project, Lilongwe, Malawi; 10 Centre AIDS Prevention Research South Africa (CAPRISA), University of KwaZulu-Natal, Durban, South Africa; 11 Hospital Federal dos Servidores do Estado, Rio de Janeiro, Brazil; 12 Department of Obstetrics and Gynaecology, Stellenbosch University, Cape Town, South Africa; 13 Department of Obstetrics and Gynaecology, BJ Government Medical College, Pune, India; 14 Perinatal HIV Research Unit, Johannesburg, South Africa; 15 Hospital Nossa Senhora da Conceicao, Porto Alegre, Brazil; 16 Research Institute for Health Sciences, Chiang Mai University, Chiang Mai, China; 17 Centre for Infectious Disease Research in Zambia, Lusaka, Zambia; 18 Instituto of Pediatrics, Federal Universida de Rio de Janeiro, Rio de Janeiro, Brazil; 19 Frontier Science and Technology Research Foundation, Amherst, NY, United States; 20 Eunice Kennedy Shriver National Institute of Child Health and Human Development, Bethesda, MD, United States; 21 Division of AIDS, National Institute of Allergy and Infectious Diseases, Bethesda, MD, United States; 22 Division of Infectious Diseases, University of California Los Angeles, Los Angeles, CA, United States; 23 Department of Pathology, Johns Hopkins University School of Medicine, Baltimore, MD, United States

## Abstract

**Introduction:**

The PROMISE trials enrolled 5398 asymptomatic HIV-infected pregnant women not eligible for antiretroviral treatment (ART) and randomly assigned different antiretroviral strategies to assess vertical transmission during pregnancy and post-delivery, infant safety and maternal health. The START study subsequently demonstrated clear benefit in initiating ART regardless of CD4 count. The PROMISE study team informed active participants of these results and strongly recommended that women not receiving ART immediately initiate treatment to optimize their own health. We summarize PROMISE participants’ responses to these recommendations and their reasons given to either accept or decline early ART.

**Methods:**

A mixed methods approach was used to gather responses from participants receiving the START information. Staff actively contacted participants to return to the clinic and delivered START results, utilizing a structured script and assessing comprehension. Women not on ART were advised to accept the offer to initiate ART, during a client-centred counselling session. Women selected their primary reason for accepting or rejecting the offer of early ART from a set of closed options. We report the uptake of early ART and the primary reasons in support of their decisions.

**Results:**

The 1483 women not on ART were advised to initiate ART. The offer was accepted by 984 women (66%) but 499 (34%) declined. Acceptance rates by country varied.

Women declined ART as they wanted more time to consider (200/494; 40%) and felt well, and knew CD4 count was high (89/494; 18%). The women accepting early ART did so for health concerns (444/792; 56%) and because of the recommendation given by the protocol team (348/792; 44%).

**Conclusions:**

A substantial number of women were not willing to initiate early ART after a single counselling session. Over one third needed more time to consider the offer to start early ART for their own health. This finding is important to ART programme implementers scaling up test-and-treat strategies.

**Figure:** Abstract THAB0106LB–HIV-infected mothers accepting Early ART after single counselling session

# Sub article: THAB0201 Who benefited most from immediate treatment in START? A subgroup analysis

**Published:** 2016-07-22

**Authors:** J-M Molina, B Grund, F Gordin, I Williams, M Schechter, M Losso, M Law, E Ekong, N Mwelase, A Skoutelis, MJ Wiselka, L Vanderkerckhove, T Benfield, D Munroe, J Lundgren, J Neaton Insight Start Study Group

**Affiliations:** 1 University of Paris Diderot, Paris, France; 2 ANRS, Paris, France; 3 Division of Biostatistics, University of Minnesota, Minneapolis, MN, United States; 4 VA Medical Center, Washington DC, United States; 5 University College London Medical School, London, United Kingdom; 6 Hospital Escola Sao Francisco de Assis, Rio de Janeiro, Brazil; 7 Hospital General de Agudos JM Ramos Mejia, Buenos Aires, Argentina; 8 The Kirby Institute, Sydney, Australia; 9 Institute of Human Virology Nigeria, Abuja, Nigeria; 10 University of Witwatersrand, Johannesburg, South Africa; 11 Evanlegismos General Hospital, Athens, Greece; 12 Leicester Royal Infirmary, Leicester, United Kingdom; 13 Universitaire Ziekenhuizen Gent, Ghent, Belgium; 14 Hvidovre University Hospital, Hvidovre, Denmark; 15 Slidell, Los Angeles, United States; 16 Rigshospitalet, University of Copenhagen, Copenhagen, Denmark

## Abstract

**Introduction:**

The strategic timing of antiretroviral treatment (START) trial showed that immediate antiretroviral therapy (ART) in asymptomatic adults with >500 CD4 cells/mm^3^ reduces the risk of primary events (a composite of serious AIDS, serious non-AIDS conditions or death) by 57% versus deferring ART until CD4<350. We investigated which subgroups benefitted most from immediate treatment.

**Methods:**

Within subgroups defined by eight predefined baseline characteristics (Figure), we estimated event rates for the START primary endpoint, and the number need to treat (NNT) immediately for 1 year to prevent one event compared with the deferral strategy. Using proportional hazards models, we estimated hazard ratios (HR) of immediate versus deferred ART within subgroups and tested for interactions between treatment groups and subgroups to assess heterogeneity of the treatment effect across subgroups.

**Results:**

Among the 4685 participants followed for a mean of 3 years, the event rates (absolute risk) for the primary endpoint in the immediate and deferred ART arms were 0.6 and 1.38 per 100 person-years (PY), respectively, NTT=128. Across all 8 subgroups, HRs consistently favoured the immediate arm (Figure). While HRs were similar across subgroups (p>0.25 for all interactions), the event rates and reductions in absolute risk were higher among older participants (NNT=50 for age>50 years), those with higher baseline HIV RNA level (NNT=67 for HIV RNA>50,000 copies/ml), higher Framingham risk score (NNT=69). There is a trend towards lower NNT among participants with lower baseline CD4 levels.

**Conclusions:**

In asymptomatic ART-naïve adults with >500 CD4 cells/mm^3^, immediate ART was superior to deferral across all subgroups, with similar relative risk reduction. Due to higher absolute risk, older participants, those with higher plasma HIV RNA level, lower baseline CD4 count, and higher Framingham risk score will benefit more from immediate treatment. Immediate ART also reduces transmission risk, which is essential for cost-effectiveness considerations, but not reflected in NNT.

**Abstract THAB0201–Figure 1:** Incidence of serious AIDS or serious non-AIDS events and NNT by subgroups.

# Sub article: THAB0202 Increased risk of suicidal behaviour with use of efavirenz: results from the START trial

**Published:** 2016-07-22

**Authors:** A Arenas-Pinto, B Grund, S Sharma, E Martinez, N Cummins, J Fox, KL Klingman, D Sedlacek, S Collins, PM Flynn, WM Chasanov, E Kedem, C Katlama, J Sierra-Madero, V Ormaasen, P Brouwers, D Cooper, for the Insight Start Study Group

**Affiliations:** 1 MRC Clinical Trials Unit at UCL, University College London, London, United Kingdom; 2 Division of Biostatistics, University of Minnesota, Minneapolis, MN, United States; 3 Department of Infectious Diseases, Hospital Clinic, Barcelona, Spain; 4 Mayo Clinic, Rochester, MN, United States; 5 Guy's and St. Thomas’ NHS Foundation Trust, London, United Kingdom; 6 Division of AIDS (DAIDS), NIH – National Institute of Allergy and Infectious Diseases, Washington, United States; 7 University Hospital Plzen, Plzen, Czech Republic; 8 HIV i-Base, London, United Kingdom; 9 St. Jude Children's Research Hospital, Memphis, TN, United States; 10 Cooper University Hospital, Camden, NJ, United States; 11 Clinical Immunology Unit, Rambam Health Care Campus, Haifa, Israel; 12 Hospitalier Pitié-Salpétrière, Paris, France; 13 Instituto Nacional de Ciencias Médicas y Nutrición Salvador Zubirán, Mexico City, Mexico; 14 Oslo University Hospital, Oslo, Norway; 15 National Institute of Mental Health, Washington, United States; 16 The Kirby Institute, Sydney, Australia

## Abstract

**Introduction:**

Randomized trials have shown increased risk of suicidality associated with efavirenz (EFV). The strategic timing of antiretroviral treatment (START) trial randomized ART-naïve HIV-positive adults with high CD4 cell counts to immediate versus deferred ART (initiation at CD4 <350 cells/mm^3^). EFV-based regimens were common.

**Methods:**

We compared the rate of suicide or investigator reported suicidal and self-injurious behaviour (“suicidal behaviour”) between the immediate versus deferred ART groups using Cox proportional hazards models. Before randomization, the initial ART regimen was pre-specified for each participant. We compared the treatment groups, overall, and separately for those with EFV-containing and EFV-free pre-specified regimens using intention to treat (ITT) analyses (all of follow-up and first year), and after censoring participants in the deferred arm at ART initiation.

**Results:**

Of the 4685 participants, median age 36 years, 270 (5.8%) had prior psychiatric diagnoses. EFV was pre-specified for 3516 participants (75%); less often in those with psychiatric diagnosis (40%) than without (77%). While the overall ITT comparison shows no difference in suicidal behaviour between immediate and deferred ART (51 events, HR=1.07, p=0.80) (Table), subgroup analyses suggest that those who pre-specified EFV-containing regimens were at an increased risk of suicidal behaviour compared to those who did not. In the deferred group, 13 of 24 events occurred after ART start, median time 5.6 months. When censoring follow-up at ART start in the deferred group, the HR for EFV versus their ART-naïve controls was 4.16 (p=0.02) and the HR for non-EFV regimens versus their ART-naïve controls was 1.04 (p=0.93) (p=0.05 for difference in HRs). Excess risk associated with EFV was greater for those with a psychiatric diagnosis at baseline (table footnotes c–d). (Table. Suicidal and self-injurious behaviour)

**Conclusions:**

These findings suggest that participants using EFV in the immediate ART group, particularly those with a prior psychiatric diagnosis, had an increased risk of suicidal behaviour compared to ART-naïve controls.

**Abstract THAB0202–Table 1:** Suicidal and self-Injurious behaviour

	No. pts with events (rate per 100 PY)							
	
Comparison	Pre-specified ART regiment	No. pts	Imm. ART	Def. ART	HR^a^ (95% CI)	P	HR Ratio	Int. p^b^
By intent-to-treat (ITT) (mean follow-up 3.0 yrs)	Any regimen (all pts)	4685	27 (0.39)	24 (0.34)	1.07 (0.6, 1.9)	0.80	NA	NA
ITT, subgroup analysis (mean follow-up 3.0 yrs)	EFV based	3516	18 (0.34)	11 (0.21)	1.42 (0.7, 3.0)	0.37	1.92	0.23
	No EFV	1169	9 (0.53)	13 (0.72)	0.74 (0.3, 1.8)	0.50		
ITT, 1st year only, subgroup analysis (mean	EFV-based	3515	9 (0.52)	2 (0.11)	3.75 (0.8. 17.5)	0.09	3.68	0.15
follow-up 1.0 yrs)	No EFV	1169	7 (1.25)	7 (1.19)	1.02 (0.4. 2.9)	0.96		
Immediate: follow-up from EFV/non-EFV ART start (mean 3.0yrs);	EFV-based^c^	3516	17 (0.35)	3 (0.08)	4.16 (1.2, 14.4)	0.02	4.00	0.05
Deferred: Follow-up censored at ART start (mean follow-up 2.1 yrs).	No EFV^d^	1137	9 (0.59)	8 (0.69)	1.04 (0.4, 2.7)	0.93		

a Estimated in CDX proportional hazards models, stratified by psychiatric diagnosis

b Interaction between indicators for treatment group and pre-specified regimen

c Of these events, 6 and 0, in the immediate us deferred arms respectively, occurred among 108 participants with prior psychiatric diagnoses

d Of these events, 5 and 2, in the immediate vs deferred arms respectively, occurred among 162 participants with prior psychiatric diagnoses. Of the 1169 participants without EFV in the pre-specified regimen, 32 were excluded (in the immediate group, 7 never started ART, and for 25, the first ART regimen contained EFV). Follow-up in the immediate group was censored at EFV start.

# Sub article: THAB0203 STRIIVING: switching to abacavir/dolutegravir/lamivudine fixed dose combination (ABC/DTG/3TC FDC) from a PI, INI or NNRTI based regimen maintains HIV suppression at week 48

**Published:** 2016-07-22

**Authors:** JE Lake, B Trottier, J Garcia-Diaz, H Edelstein, P Kumar, UF Bredeek, M Loutfy, C Brennan, J Koteff, B Wynne, J Hopking, M Aboud

**Affiliations:** 1 Division of Infectious Diseases, UCLA, Los Angeles, CA, United States; 2 Clinique Med.e L'actuel, Montreal, Canada; 3 Ochsner Clinic Foundation, New Orleans, LA, United States; 4 Highland Hospital, Alameda Health System, Oakland, CA, United States; 5 Georgetown University, Washington, DC, United States; 6 Metropolis Medical, San Francisco, CA, United States; 7 Maple Leaf Research, Toronto, Canada; 8 ViiV Healthcare, Research Triangle Park, NC, United States; 9 GlaxoSmithKline, London, United Kingdom; 10 ViiV Healthcare, London, United Kingdom

## Abstract

**Introduction:**

STRIIVING is a phase 3b, randomized, open-label, North American, 48-week (W) study and is the first to evaluate the efficacy, safety, pharmacokinetics and health outcomes of switching from a protease inhibitor (PI), non-nucleoside reverse transcriptase inhibitor (NNRTI) or integrase inhibitor (INI) based regimen to ABC/DTG/3TC FDC in virologically suppressed (HIV-1 RNA <50 copies/ml (c/ml)) participants. Here we report the W48 efficacy and safety results.

**Methods:**

Participants were randomized 1:1 to switch to ABC/DTG/3TC FDC at baseline (early switch, or ES) or maintain current ART (cART) with a switch to ABC/DTG/3TC at W24 (late switch, or LS). The primary endpoint was non-inferiority (-10% margin) of ABC/DTG/3TC relative to cART in maintaining plasma HIV-1 RNA<50 c/ml at W24 by FDA snapshot. Plasma HIV-RNA<50 c/ml was summarized as a secondary endpoint at W48 after all subjects switched to the DTG-based FDC.

**Results:**

A total of 553 participants were randomized and treated (ABC/DTG/3TC 275, cART 278). Baseline characteristics were similar across treatment arms. The primary endpoint of non-inferiority at W24 was met (HIV-1 RNA<50 c/ml; ABC/DTG/3TC 85% vs. cART 88%). At W48, 219/275 (80%) of ES subjects were virologically suppressed with HIV-1 RNA <50 c/ml. The proportion of LS subjects (n=222/244) with HIV-1 RNA <50 c/ml at W48 was comparable to the proportion at W24 for the ES arm (LS to ABC/DTG/3TC 91%; ES to ABC/DTG/3TC 85%). There were no protocol defined virologic failures or treatment emergent resistance in either arm. In the ES arm, 10 (4%) subjects withdrew due to AEs in the first 24 weeks of ABC/DTG/3TC; in the LS arm, 4 (2%) withdrew due to AEs following switch to ABC/DTG/3TC.

**Conclusions:**

In virologically suppressed patients, switching to once daily ABC/DTG/3TC FDC was non-inferior to continuing cART with no evidence of virologic failure or treatment emergent resistance through 24 weeks. Early switch subjects also maintained virologic suppression from W24 through W48. There were fewer withdrawals due to AEs in the LS versus ES arm. The safety profile of ABC/DTG/3TC in STRIIVING is consistent with current labelling for ABC/DTG/3TC.

# Sub article: THAB0204 Experiences with long-acting injectable ART: a qualitative study among people living with HIV participating in a phase II study of cabotegravir+ rilpivirine (LATTE-2) in the United States and Spain

**Published:** 2016-07-22

**Authors:** D Kerrigan, A Mantsios, D Margolis, M Murray

**Affiliations:** 1 Department of Health, Behavior and Society, Johns Hopkins University, Baltimore, MD, United States; 2 ViiV Healthcare, Raleigh-Durham, United States; 3 ViiV Healthcare, London, United Kingdom

## Abstract

**Introduction:**

Adherence to antiretroviral therapy (ART) among people living with HIV (PLHIV) is essential to improve individual outcomes and curb ongoing transmission. Challenges with daily oral medication have stimulated the development of long-acting injectable (LAI) ART as a means to address barriers.

**Methods:**

We conducted 39 in-depth(s1) interviews including with 27 PLHIV (25 men, 2 women) and 12 clinical providers participating in a Phase IIb study (LATTE-2) evaluating a long-acting intramuscular ART regimen in the United States and Spain. Participants were treatment-naïve upon study entry and randomized to daily oral ART or LAI ART every 4 or 8 weeks. Interviews explored participant and provider attitudes and experiences with daily oral and LAI ART. Interviews were audiotaped, transcribed, coded and analyzed using thematic content analysis. All trial participants had completed a minimum of 32 weeks of LAI ART following 20 weeks of oral ART.

**Results:**

Almost all participants experienced some level of side effects associated with LAI ART, mostly temporary soreness at the injection site. Yet, all reported being satisfied and interested in continuing LAI ART. Participants relayed practical and emotional benefits of LAI ART compared to oral ART. Practical benefits included convenience and logistical ease of receiving an injection every 4 or 8 weeks versus a daily pill. In many cases, participants reported LAI ART helped them manage stigma. LAI ART was seen as more discreet with less possibility of others discovering one's HIV status and it did not involve the “daily reminder of living with HIV.” Most participants felt LAI ART could be beneficial to all PLHIV bu0074 particularly those with oral ART adherence challenges. While providers recognized the benefits of LAI ART, they expressed concerns LAI ART candidates would still need to be able to adhere to clinic visits for injections and concerns regarding the clinical management of LAI ART if it were necessary to stop the regimen given its long-acting nature.

**Conclusions:**

LAI ART was preferable to a daily oral regimen among PLHIV participating in a Phase IIb trial given its practical and emotional benefits. Further research is needed regarding appropriate candidates for LAI ART including among women and “non-adherent” populations.

# Sub article: THAB0205LB Superior efficacy of dolutegravir/abacavir/lamivudine (DTG/ABC/3TC) fixed dose combination (FDC) compared with ritonavir (RTV) boosted atazanavir (ATV) plus tenofovir disoproxil fumarate/emtricitabine (TDF/FTC) in treatment-naïve women with HIV-1 infection (ARIA Study)

**Published:** 2016-07-22

**Authors:** C Orrell, D Hagins, E Belonosova, N Porteiro, S Walmsley, V Falcó, C Man, A Aylott, A Buchanan, B Wynne, C Vavro, M Aboud, K Smith

**Affiliations:** 1 University of Cape Town, Desmond Tutu HIV Foundation, Cape Town, South Africa; 2 Chatham County Health Department, Savannah, GA, United States; 3 Orel Regional Center for AIDS, Orel, Russian Federation; 4 Fundación IDEAA, Buenos Aires, Argentina; 5 University Health Network, Toronto, ON, Canada; 6 Hospital Vall d'Hebron, Barcelona, Spain; 7 ViiV Healthcare, Research Triangle Park, NC, United States; 8 GlaxoSmithKline, Stockley Park, United Kingdom; 9 ViiV Healthcare, Philadelphia, PA, United States; 10 ViiV Healthcare, Brentford, United Kingdom

## Abstract

**Introduction:**

The FDC of DTG/ABC/3TC is built upon an unboosted integrase-strand transfer inhibitor (INSTI) and may offer a simplified regimen for the treatment of HIV-1 infection. To gain additional data for women on this regimen, we conducted ARIA, an international, randomized, open-label study to evaluate the safety and efficacy of DTG/ABC/3TC versus ATV + RTV + FTC/TDF (ClinicalTrials.gov: NCT01910402).

**Methods:**

Treatment-naïve adult women, with HIV-1 RNA ≥500 copies (c)/mL were randomized (1:1, stratified by plasma HIV-1 RNA and CD4+ count) to 48 weeks of treatment with DTG/ABC/3TC or ATV + RTV + FTC/TDF once daily. The primary endpoint was the proportion of women achieving an HIV-1 RNA <50 c/mL at week 48 (Snapshot algorithm). Women who became pregnant were withdrawn, and where possible offered an option to enter a DTG/ABC/3TC pregnancy study.

**Results:**

495 women were randomized and treated. Median age was 37 years. Subjects were well matched for demographic and baseline (BL) characteristics. DTG/ABC/3TC was superior to ATV + RTV + FTC/TDF, with 82% and 71%, respectively, achieving HIV-1 RNA <50 c/mL at week 48 (adjusted difference 10.5%, 95% CI: 3.1%–17.8%, p = 0.005). Differences were driven by lower rates of both discontinuations due to adverse events (AEs) and Snapshot virologic failures in the DTG/ABC/3TC group. Increases in CD4+ count were similar between treatment groups. The safety profile of DTG/ABC/3TC was favourable compared to ATV + RTV + TDF/FTC, with fewer drug-related AEs reported in the DTG/ABC/3TC group. Of six DTG/ABC/3TC subjects who met protocol-defined virologic withdrawal criteria, none had treatment-emergent primary INSTI or ABC/3TC resistance mutations, compared with four ATV + RTV + TDF/FTC subjects who met virologic withdrawal criteria of which one had an emergent NRTI mutation, M184M/I/V.

**Conclusions:**

DTG/ABC/3TC demonstrated superior efficacy and a favourable safety profile compared with ATV + RTV + FTC/TDF in treatment-naïve women after 48 weeks of treatment. The study provides important information to help guide treatment decisions for women.

**Abstract THAB0205LB–Table 1:** Key results from ARIA study in treatment-naïve women

	DTG/ABC/3TC, N = 248	ATV + RTV + TDF/FTC, N = 247
Race: White (%), African heritage (%)	46, 41	43, 44
Median BL CD4+ (cells/mm^3^)	340	350
Median BL HIV-1 RNA (log_10_ c/mL)	4.41	4.43
Week 48 HIV-1 RNA <50 c/mL (Snapshot, %)	82	71
Median change in CD4+ at Week 48 (cells/mm^3^)	234	200
Virologic failure (Snapshot, %)	6	14
Discontinuations due to AEs (%)	4	7
Pregnancies (%)	2	3
Drug-related AEs (%)	33	49

# Sub article: THAB0206LB Cabotegravir + rilpivirine as long-acting maintenance therapy: LATTE-2 week 48 results

**Published:** 2016-07-22

**Authors:** D Margolis, D Podzamczer, H-J Stellbrink, T Lutz, J Angel, G Richmond, B Clotet, F Gutierrez, L Sloan, S Griffith, M St Clair, D Dorey, S Ford, J Mrus, H Crauwels, K Smith, P Williams, W Spreen

**Affiliations:** 1 ViiV Healthcare, Durham, NC, United States; 2 Hospital Universitario Bellvitge, Barcelona, Spain; 3 ICH, Hamburg, Germany; 4 Infektiologikum, Frankfurt, Germany; 5 The Ottawa Hospital, Ottawa, Canada; 6 Broward Health, Fort Lauderdale, FL, United States; 7 Hospital Germans Trias i Pujol, Barcelona, Spain; 8 Hospital General de Elche, Alicante, Spain; 9 North Texas Infectious Disease Consultants, Dallas, TX, United States; 10 GlaxoSmithKline, Mississauga, Canada; 11 Parexel, Durham, NC, United States; 12 Janssen Research and Development, Beerse, Belgium

## Abstract

**Introduction:**

Cabotegravir (CAB) and rilpivirine (RPV) are under development as long-acting (LA) injectable nano-suspensions. LATTE-2 was designed to select an intramuscular (IM) regimen of CAB LA + RPV LA and to evaluate safety and efficacy of 2-drug IM ART, relative to 3-drug oral ART (CAB + ABC/3TC) to maintain viral suppression of HIV-1.

**Methods:**

Phase 2b, multicentre, parallel group, open-label study in ART-naïve HIV-infected adults. Enrolled patients with plasma HIV-1 RNA <50 c/mL during the 20-week Induction Period on daily oral CAB 30 mg + ABC/3TC were randomized 2:2:1 to IM CAB LA + RPV LA every 4 weeks (Q4W), every 8 weeks (Q8W), or oral CAB + ABC/3TC (PO) in the Maintenance Period (MP). Dosing regimens were evaluated according to antiviral activity, protocol-defined virologic failure (PDVF) and safety at the pre-specified week 48 endpoint in MP (ITT Maintenance Exposed (ME)).

**Results:**

309 patients were enrolled (ITT-Exposed): 91% male, 20% non-white and 19% > 100,000 c/mL HIV-1 RNA. 286 patients were randomized into the MP. At Week 48, 92 (Q8W), 91 (Q4W) and 89% (PO) remained suppressed (ITT-ME). More patients on Q8W (5%), relative to Q4W (<1%) and PO (0%) had HIV-1 RNA >50 c/mL at Week 48; 5/6 Q8W patients subsequently achieved HIV-1 RNA <50 c/mL. Three ME patients had PDVF during MP (PO (W8); Q8W (W4, W48)); one with NNRTI/INI mutations (Q8W (W48)). Grade 1/2 injection site pain occurred commonly, median duration 3 days, <1% ISR withdrawals. MP SAEs occurred in IM (7%) and PO (5%), none drug-related.

**Conclusions:**

Both Q8W and Q4W IM dosing demonstrated good virologic response rates, and were generally well tolerated through 48 weeks. Q4W dosing resulted in modestly lower rates of virologic non-response than Q8W. Q4W dosing was chosen for progression into phase 3 studies while Q8W and Q4W remain under evaluation within LATTE-2.

**Table:** Abstract THAB0206LB–Week 48 Outcomes

Week 48 Snapshot Study Outcomes (ITT-ME)	CAB LA+RPV LA Q8W (n=115)	CAB LA+RPV LA Q4W (n=115)	Oral CAB 30 mg+ABC/3TC (n=56)
%HIV-1RNA <50 c/mL at W48: Dlff in Proportions (95% C1)*	92%** (2.9: −6.6, 12.4)	91%** (2.0: −7.6, 11.6)	89%
Snapshot Virologic Non-response	8 (7%)	1 (<1%)	1 (2%)
Data in window not <50 c/mL	6 (5%)	1 (<1%)	0
Discontinued due to lack of efficacy (PDVF)	1 (<1%)	0	1 (2%)
Discontinued due to Other* Reasons while Not Suppressed	1 (<1%)†	0	0
Snapshot No Virologic Data	1 (<1%)	9 (8%)	5 (9%)
Discontinued due to AE or Death	0	6 (5%)†‡	2 (4%)‡
Discontinued due to Other Reasons while Suppressed	1 (<1%)†	3 (3%)	3 (5%)
Other Results			
Number of injections Number of ISR events Grade 1 – mild (%) Grade 2 – moderate (%) ISR Duration ≤7 days	2126 1275 1017 (80%) 245 (19%) 1135 (89%)	358S 1568 1321 (84%) 234 (15%)1414 (90%)	NA
Median CD4+ cells/mm^3^ Baseline Change from Baseline at W48 (IQR)***	449 +248 (152, 347)	499 +258 (133, 355)	518 +307 (199, 566)

Intent to Treat- Maintenance Exposed (ITT-ME)

BL=baseline (last value prior to first Induction Period dose at Week −20)

IQR=Interquartile range

* W48 represents 68 weeks on study (20 Week Induction Period followed by a 48 Week two drug Maintenance Period)

** Met pre-specified threshold for concluding IM regimen is comparable to oral regimen (Bayesian Posterior Probability >90% that true IM response rate is no worse than −10% compared to the oral regimen)

*** Based on observed values at Week 48 (Q8W: n=112; Q4W: n=104; Oral: n=50)

† Withdrew consent due to intoierabllity of injections

‡ Acute HCV (n=2), rash (n=l), depressive reaction (n=l), psychotic state (n=l), DILI (n=1), Churg Strauss vasculitis (n=l), epilepsy (death) (n=l)

# Sub article: THAC0101 A small proportion of acts of anal intercourse within homosexual male serodiscordant couples in three countries are high-risk for HIV transmission

**Published:** 2016-07-22

**Authors:** BR Bavinton, N Phanuphak, F Jin, I Zablotska, B Grinsztejn, G Prestage, AE Grulich, Opposites Attract Study Group

**Affiliations:** 1 The Kirby Institute, The University of New South Wales, Sydney, Australia; 2 Thai Red Cross AIDS Research Centre, Bangkok, Thailand; 3 Instituto de Pesquisa Clínica Evandro Chagas, Rio de Janeiro, Brazil; 4 Australian Research Centre in Sex, Health and Society, La Trobe University, Melbourne, Australia

## Abstract

**Introduction:**

There are few data about the range of strategies used to prevent sexual HIV transmission within homosexual male serodiscordant couples (HM-SDC).

**Methods:**

Opposites Attract is an ongoing cohort study of HM-SDC. At baseline, HIV-positive partners (HPP) had viral load (VL) tested; HIV-negative partners (HNP) reported the previous three months' sexual behaviour and perception of the HPP's last VL test. Each act of condomless anal intercourse (CLAI) within couples was categorised by HIV prevention strategy.

**Results:**

By February 2016, 331 couples were enrolled (Australia=151, Brazil=91, Thailand=89). At baseline, 78.8% of HPPs had undetectable VL (UVL); however, only 55.9% of HNPs perceived their partners to have UVL (96.4% of HPPs who were perceived to have UVL actually did). In the previous three months, 53.2% of couples had CLAI: 46.5%, 28.1%, and 15.7% of HNPs reported insertive CLAI, receptive CLAI with withdrawal, and receptive CLAI with ejaculation respectively. Eighteen HNPs (5.4%) took daily pre-exposure prophylaxis (PrEP). Over the previous three months, HNPs reported a total of 8439 acts of anal intercourse with their HPP (mean per couple=25.5). Of these, 4627 (54.8%) were protected by condoms, while there were 3812 (45.2%) acts of CLAI. Of the CLAI acts, 2488 (65.3%) were when the HNP perceived his HPP to have UVL; 94 (2.5%) were protected by PrEP in the HNP; and 244 (6.4%) were protected by perceived UVL in the HPP and PrEP in the HNP. Of the remaining 986 CLAI acts where the perceived VL was detectable or unknown and were not protected by PrEP, 484 were when the HNP was insertive (strategic positioning) and 428 were when the HNP was receptive (277 with withdrawal and 151 with ejaculation). Overall, 53.9% of all anal intercourse acts reported by HNPs were protected by condom use, 33.6% by perceived UVL, 4.2% by PrEP, and 6.0% by strategic positioning; while 3.4% were receptive with withdrawal, and 1.9% were receptive with ejaculation.

**Conclusions:**

Couples used condoms, PrEP or perceived UVL for prevention in the vast majority of anal intercourse acts. Only a very small proportion of events were not protected, and the majority of receptive CLAI acts involved withdrawal.

# Sub article: THAC0102 Is pre-exposure prophylaxis needed for men who have sex with men in West Africa? HIV incidence data from a prospective multi-country cohort study (CohMSM ANRS 12280)

**Published:** 2016-07-22

**Authors:** C Couderc, B Dembélé Keita, C Anoma, AS Wade, A Ouédraogo, A Coulibaly, S Ehouman, AK Diop, M Somda, Y Yomb, E Henry, B Spire, C Laurent

**Affiliations:** 1 Université de Montpellier, TransVIHMI, IRD UMI 233 / INSERM U 1175, Montpellier, France; 2 ARCAD-SIDA, Bamako, Mali; 3 Espace Confiance, Abidjan, Cote D'Ivoire; 4 Division SIDA/IST, Ministère de la santé, de l'Hygiène Publique et de la Prévention, Dakar, Senegal; 5 Centre Muraz, Bobo-Dioulasso, Burkina Faso; 6 REVS+, Bobo-Dioulasso, Burkina Faso; 7 Alternatives Cameroon, Douala, Cameroon; 8 Coalition PLUS, Paris, France; 9 SESSTIM UMR 912, Inserm / IRD / Université Aix-Marseille, Marseille, France

## Abstract

**Introduction:**

The World Health Organization (WHO) recommends from September 2015 to use pre-exposure prophylaxis (PrEP) as part of a comprehensive HIV prevention package for people at substantial risk of HIV infection (incidence greater than 3 per 100 person-years in the absence of PrEP). Men who have sex with men (MSM) are one of the most vulnerable populations and may be eligible to PrEP. However, few data are available among this population in Africa. We therefore estimated the incidence of HIV infection among MSM in four West African countries.

**Methods:**

A prospective cohort study was conducted in 2013–2014 in Bamako (Mali), Abidjan (Côte d'Ivoire), Dakar (Senegal) and Bobo-Dioulasso (Burkina Faso). Men over 18 years, reporting at least one sexual relationship with another man within the last three months, and HIV-negative (status confirmed at inclusion in the study) were eligible. A 6-month follow-up was offered to them including a quarterly HIV screening (M3 and M6) along with pre- and post-screening counselling and free condoms. If necessary, treatment for sexually transmitted infections was provided.

**Results:**

A total of 440 HIV-negative MSM were recruited. Of them, 316 (71.8%) had at least one screening test during follow-up: 168 (53.2%) in Mali, 73 (23.1%) in Côte d'Ivoire, 54 (17.1%) in Senegal and 21 (6.6%) in Burkina Faso. The median age was 23.7 years (interquartile range (IQR): 20.8–28.0). These men were followed up for a total period of 167.9 person-years. During follow-up, HIV screening tests were performed after a median time from inclusion of 3.2 months (IQR: 3.0–3.6) and 6.3 months (IQR: 6.0–6.6). Eight seroconversions were observed (six at the first screening test and two at the second test), giving an incidence rate of 4.8 per 100 person-years (95% confidence interval (CI): 2.4–9.5).

**Conclusions:**

Based on HIV incidence observed in this study, MSM living in West African countries are eligible for PrEP according to the WHO-recommended criteria. Operational research is now needed to guide the implementation of specific programs for prevention and comprehensive care including PrEP in this context.

# Sub article: THAC0103 Reaching the unreachable: MSM recruitment strategy using social networks to HIV prevention services in Guatemala city

**Published:** 2016-07-22

**Authors:** R Mendizabal-Burastero, C Galindo-Arandi, MP Yancor, I Vela, JM Aguilar-Martinez

**Affiliations:** Colectivo Amigos contra el Sida, Guatemala, Guatemala

## Abstract

**Introduction:**

Men who have sex with men (MSM) report a 9% HIV prevalence in Guatemala, and only 45% of them had an HIV test done in the last 12 months. Evaluation of prevention programs report that these interventions target the same MSM with the same messages repeatedly; and the coverage of HIV prevention interventions represent less than 10% of MSM estimation. The need to improve access to HIV testing is an urgency in Guatemala.

**Description:**

Colectivo Amigos contra el Sida (CAS) is a mostly gay association that work in HIV prevention. Several strategies were developed to increase HIV tested and diagnosed MSM in Guatemala city in 2014 and 2015. First, a strong group of volunteers were conformed and trained, a result of the implementation of HIV prevention model Mpowerment. An intense work of “snowball” outreach by social networks was performed. Facebook, Twitter, Grindr and WhatsApp were used to promote HIV testing services, by direct message for promotion. Each promoter started with their own social network, and the inclusion of new volunteers, that shared their networks, helped to continue the recruitment. Also, the inclusion in sex encounters groups in WhatsApp or Facebook also provided an interesting platform to find MSM. The MSM “social stars” also represented a good way to reach more MSM networks.

**Lessons learned:**

A total of 7244 gay men and other MSM were recruited between July 2014 and December 2015, with nearly 50% getting tested for HIV. Nearly 200 HIV cases were diagnosed, making a twist in male/female ratio in national HIV statistics, from 1.4 in 2013 to 2.1 in 2015. Compared to MSM estimations, 23% of MSM in Guatemala city were tested for HIV, and 22% of the HIV estimated cases in MSM for these city were diagnosed and linked to an HIV service. These new strategies to outreach MSM seems to be promising in a low income country.

**Conclusions/Next steps:**

Expansion to other cities in Guatemala is needed to increase access to MSM for HIV services. More intense work with other community based organization (CBO), to use these model is also a major challenge. Training of other CBOs in Central America will also help to improve current strategies for these population.

# Sub article: THAC0104 Trends in internet use to meet sex partners among men who have sex with men

**Published:** 2016-07-22

**Authors:** G Paz-Bailey, B Hoots, M Xia, T Finlayson, J Prejean, D Purcell, for the Nhbs Study Group

**Affiliations:** 1 Centers for Disease Control and Prevention, Atlanta, GA, United States; 2 ICF Macro, Atlanta, GA, United States; 3 Centers for Disease Control and Prevention (CDC), Atlanta, GA, United States

## Abstract

**Introduction:**

Internet-based platforms are increasingly prominent interfaces for sexual networking among men who have sex with men (MSM). We used data among MSM participating in the National HIV Behavioral Surveillance to evaluate changes from 2008 to 2014 in using the internet to meet sex partners and in having met the last sex partner online. We also investigated the association of internet use and partner seeking and testing behaviour in 2014.

**Methods:**

MSM were recruited through venue-based sampling in 2008, 2011, and 2014 in 20 U.S. cities. Among men reporting ≥ 1 male partner in the past 12 months, we used log-linked Poisson regression with GEE to calculate adjusted prevalence ratios (APR) and 95% confidence intervals (CI) to compare internet use (IU) to meet sex partners and meeting the last sex partner online by year. Models were adjusted for age, race, and education. We used the Wilcoxon rank sum and chi-square tests to compare factors associated with increased IU. IU was categorized as ≤ once a month, > once a month but < once a week, and ≥ once a week.

**Results:**

IU at least once a week increased from 20% in 2008 to 44% in 2014 (APR=2.2, 95% CI: 2.1–1.3). Similarly, having met the last partner online increased from 19% in 2008 to 32% in 2014 (APR=1.7, 95% CI: 1.6–1.8). Median number of partners in the past 12 months increased with increasing IU (≤ once a month: median of 2 partners, interquartile range (IQR): 1–5; >once a month: 4, IQR: 2–9; ≥ once a week: 5, IQR: 3–12, P<0.0001). HIV testing in the past 12 months also increased with increasing IU (59, 68, and 71%, respectively, P<0.0001). While the percent HIV-positive and aware of their status was similar by frequency of IU (16%), the percent HIV-positive but unaware decreased as IU increased (6, 5, and 4%, P<0.0001).

**Conclusions:**

Both internet use to meet sex partners and meeting the last partner online have increased since 2008. Although men who used the internet more frequently reported more partners, they were also more likely to report testing and were less likely to be HIV-positive but unaware.

# Sub article: THAC0105LB Incidence and correlates of STIs among Black men who have sex with men participating in a US PrEP study

**Published:** 2016-07-22

**Authors:** L Hightow-Weidman, M Magnus, G Beauchamp, C Hurt, S Shoptaw, L Emel, E Piwowar-Manning, K Mayer, L Nelson, L Wilton, P Watkins, S Fields, D Wheeler

**Affiliations:** 1 Behavior and Technology Lab, Institute for Global Health and Infectious Diseases, University of North Carolina at Chapel Hill, Chapel Hill, NC, United States; 2 George Washington University, Washington, DC, United States; 3 Fred Hutchinson Cancer Research Center, Seattle, WA, United States; 4 Medicine/Infectious Diseases, University of North Carolina at Chapel Hill, Chapel Hill, NC, United States; 5 UCLA, Los Angeles, CA, United States; 6 The Fenway Institute, Boston, MA, United States; 7 University of Rochester, Rochester, NY, United States; 8 Binghamton University, Binghamton, NY, United States; 9 FHI 360, Durham, NC, United States; 10 Charles R. Drew University, Los Angeles, CA, United States; 11 University of Albany-SUNY, Albany, NY, United States

## Abstract

**Introduction:**

HPTN 073 assessed the feasibility, acceptability and safety of pre-exposure prophylaxis (PrEP) for Black men who have sex with men (BMSM). Understanding the relationship between PrEP uptake and sexually transmitted infection (STI) acquisition is critical to informing best practices in PrEP delivery for BMSM, a population most highly affected by HIV in the US.

**Methods:**

From August 2013 to September 2014, we enrolled 226 HIV-uninfected BMSM in three cities (Los Angeles, CA; Washington, DC; and Chapel Hill, NC). All participants received client-centred care coordination and were offered daily oral PrEP with emtricitabine/tenofovir. Men were followed for 12 months with scheduled clinical visits and STI testing (rectal and urine NAAT for gonorrhoea and chlamydia, RPR for syphilis) at weeks 26 and 52. Logistic regression was used to examine the association between STI prevalence and baseline factors. Person-years (PY) follow-up time was calculated to the first STI event or last STI date from either the PrEP acceptance date or enrolment date depending if BMSM accepted PrEP.

**Results:**

Baseline STI prevalence was 14%; no differences were noted among study sites. Men <25 were more likely to have a baseline STI (25.3% vs. 6.7%; OR = 4.39, 95% CI: 1.91, 10.11**)**. Sixty participants (26.5%) acquired ≥1 STI during follow-up, 9 participants had an STI at both follow-up visits. Higher rates of STIs were seen during follow-up among those with STIs at baseline (Table 1). STI rate was 32.8/100 PY (24.3, 43.2) among those who accepted PrEP compared to those who declined 26.8/100 PY (12.9, 49.3).

**Conclusions:**

While we found higher rates of STIs in younger BMSM, the overall rates of STI in this trial were lower than in prior PrEP trials with no increase over time. BMSM with STIs at PrEP initiation may require additional counselling on STI acquisition risk and more frequent STI testing during follow-up.

**Abstract THAC0105LB–Table 1:** Characteristics of Incident STIs by PrEP Acceptance and by visit

	Week 26 PrEP	Week 26 PrEP	Week 52 PrEP	Week 52 PrEP
	Accept % (n/N)	Not Accept % (n/N)	Accept % (n/N)	Not Accept % (n/N)
Site				
GWU CRS	20.0% (11/55)	18.2% (2/11)	21.8% (12/55)	18.2% (2/11)
UCLA CRS	14.9% (7/47)	10.5% (2/19)	25.0% (12/48)	22.2% (4/18)
UNC AIDS CRS	16.7% (10/60)	0.0% (0/6)	10.9% (7/64)	0.0% (0/6)
Age				
<25	25.4% (18/71)	30.0% (3/10)	22.9% (16/70)	16.7% (2/12)
> = 25	11.0% (10/91)	3.8% (1/26)	15.5% (15/97)	17.4% (4/23)
Baseline Any STI diagnosis				
No	12.7% (17/134)	12.1% (4/33)	15.6% (22/141)	16.8% (6/32)
Yes	**39.3% (11/28)**	0.0% (0/3)	**34.6% (9/26)**	0.0% (0/3)
Any condomless sex				
No	14.7% (10/68)	17.4% (4/23)	17.9% (15/84)	12.0% (3/25)
Yes	19.8% (16/81)	0.0% (0/11)	17.6% (13/73)	37.5% (3/8)
Any condomless				
receptive Sex				
No	16.2% (16/99)	14.8% (4/27)	16.1% (19/118)	11.1% (3/27)
Yes	20.0% (10/50)	0.0% (0/7)	23.1% (9/39)	50.0% (3/6)
Any condomless				
insertive Sex				
NO	19.5% (17/87)	15.4% (4/26)	15.8% (16/101)	11.1% (3/27)
Yes	14.5% (9/62)	0.0% (0/8)	21.4% (12/56)	50.0% (3/6)
Any alcohol/drug 2 hrs.				
before or during Sex				
No	15.8% (15/95)	8.3% (2/24)	15.7% (16/102)	18.5% (5/27)
Yes	20.4% (11/54)	20.0% (2/10)	21.8% (12/55)	16.7% (1/6)
Self-Report adherence				
< = 50 pct				
No	16.2% (18/111)	0.0% (0/0)	14.8% (13/88)	0.0% (0/0)
Yes	25.9% (7/27)	0.0% (0/0)	35.7% (5/14)	0.0% (0/0)
Self-Report adherence				
>= 90 pct				
No	20.8% (11/53)	0.0% (0/0)	18.2% (6/83)	0.0% (0/0)
Yes	16.5% (14/85)	0.0% (0/0)	17.4% (12/69)	0.0% (0/0)
Average C4 sessions				
Mean (SD)	30 (10.2)	28 (13.4)	28 (13.4)	28 (13.4)
Min, Max	15, 45	10, 65	10, 65	10, 65
25th, 75th %tile	23, 40	19, 33	19, 33	19, 33

# Sub article: THAD0101 Healthcare supply-related barriers to adherence among HIV-positive patients followed within the Cameroonian antiretroviral treatment program: the deleterious effect of stock outs (EVOLCAM – ANRS 12288)

**Published:** 2016-07-22

**Authors:** C Tong, M Suzan-Monti, L Sagaon-Teyssier, O Ossanga, C Laurent, G Maradan, A Ambani, L Vidal, B Spire, S Boyer, EVOLCAM Study Group

**Affiliations:** 1 Aix Marseille Université, UMR_S 912, IRD, Marseille, France; 2 Institut National de la Santé et de la Recherche Médicale (INSERM), UMR_S 912 (SESSTIM), Marseille, France; 3 ORS PACA, Observatoire Régional de la Santé Provence Alpes Côte d'Azur, Marseille, France; 4 Université Catholique d'Afrique Centrale, Yaoundé, Cameroon; 5 UMI 233 Institut de Recherche pour le Développement (IRD), INSERM U 1175, Université de Montpellier, Montpellier, France

## Abstract

**Background:**

Adherence to antiretroviral treatment (ART) is the main driver of virological success, an essential issue in the fight against HIV. As international financial resources are decreasing while number of ART-treated patients is increasing, healthcare supply barriers may play an important role in ART adherence. This study aimed to investigate individual and healthcare supply-related factors of non-adherence and >2 days treatment interruption (TI) among HIV-positive patients followed within the Cameroonian ART program.

**Methods:**

Present analyses included 1875 ART-treated patients in 19 HIV services in the Centre and Littoral regions of Cameroon. Data on adherence were collected using a face-to-face questionnaire. Adherence was evaluated using a validated algorithm measuring respect of the dosing schedule during the last four weeks. Two-level hierarchical logistic models were used to investigate correlates of non-adherence and >2 days TI.

**Results:**

Among study patients, 29.3% were highly adherent, 49.7% were non-adherent and 21.0% reported TI. Common factors associated with a lower risk of non-adherence and TI were current or recent tuberculosis treatment at the individual level and medium-sized hospitals at the healthcare supply level, whereas binge drinking at the individual level and occurrence of ART stock outs at the healthcare supply level were risk factors of those two outcomes. Lower educational level and having benefited from an interview with a counsellor during the past year were additional individual factors associated with a lower risk of non-adherence while people feeling stigma and taking multi-tablets ART regimen were more likely to be non-adherent. Regarding individual factors of TI, we found that older patients and those living as a couple or being single were less likely to report TI. Conversely, patients dissatisfied with their doctor's listening or having consulted a traditional healer were more likely to report TI. Furthermore, patients followed-up in decentralized hospitals in the Littoral region were at lower risk of non-adherence.

**Conclusions:**

Our study highlights suboptimal adherence outcome of the Cameroonian ART program, and a deleterious effect of ART stock outs on both adherence and TI. Unless effective ART supply management measures are urgently implemented to secure ART access, progress in the fight against HIV may be jeopardized.

# Sub article: THAD0102 Outcomes of a psychosocial support programme for HIV-infected young mothers at an antiretroviral access clinic

**Published:** 2016-07-22

**Authors:** R Phillip, T Mudzviti, T Shamu, C Chimbetete

**Affiliations:** Newlands Clinic, Harare, Zimbabwe

## Abstract

**Introduction:**

Through the maturation of antiretroviral rollout programmes several challenges have been observed in young women accessing care. These young women are reaching sexual maturity and are now having children of their own. There is a risk of these infants becoming vertically infected unless optimal adherence is maintained. Newlands Clinic runs a support group for such young women who have either become married or have children. The young mother's support group has been established to provide psychosocial support and improve the quality of life of HIV infected young women.

**Description:**

The young mothers’ support group is a support group for women less than 25 years who are in relationships, those that have been married, staying with partners with or without children. The group is structured as a monthly focused group discussion in which participants receive training and counselling concurrently in vocational skills, health issues including adherence, legal matters and continued life skill training. This training is provided by a qualified nurse counsellor equipped to deal with both medical and psychosocial issues.

**Lessons learned:**

Through the support group, 26 young mothers with a mean age of 21.5 (SD=2.10) have been retained in care from 2012 to 2015. All of the young mothers are on antiretroviral therapy (ART) with a median duration of 196 weeks (IQR=97–332) and 15 of them have maintained undetectable viral load (<20 copies per ml). The programme has seen 18 of the young mothers maintained on first line and 8 receiving second line ART. We could ascertain post weaning HIV status for 17 of their children, 16 were negative and 1 was positive. Eleven of the young mothers have been treated for sexually transmitted infections (STIs) from 2012 through to 2015.

**Conclusions/Next steps:**

Perinatally infected young girls are attaining sexual maturity and having children of their own. The prevalence of STIs among this group is very high and is a cause of concern. Creation of support groups to partner with the medical programmes helps in retaining patients in care and improving treatment outcomes which has an overall benefit to quality of life.

# Sub article: THAD0103 Approaches to care for the HIV-infected adolescents across national HIV/AIDS programs participating in the New Horizons advancing paediatric HIV care collaborative

**Published:** 2016-07-22

**Authors:** N Rakhmanina, B Corrigan, J Kose, K Manson, New Horizons Advancing Pediatric HIV Care Collaborative

**Affiliations:** 1 Elizabeth Glaser Paediatric AIDS Foundation, Washington, DC, United States; 2 Pediatrics, Children's National Health System, George Washington University, Washington, DC, United States; 3 Janssen-Cilag Ltd, London, United Kingdom

## Abstract

**Introduction:**

*New Horizons* (NH) *Advancing Pediatric HIV Care* is a multi-sector collaborative to advance a holistic integrated approach, promote best practice sharing and research, and leverage resources for improved management of treatment-experienced paediatric patients on second- and third-line antiretroviral treatment. The NH collaborative also aims to create a framework of support for HIV-infected adolescents and their care providers. The objective of this analysis is to describe the national approaches for adolescent disclosure of HIV status and transition to adult HIV care in country programs currently participating in the NH collaborative.

**Methods:**

Data were collected from four national HIV/AIDS programs (Kenya, Zambia, Swaziland, and Lesotho) during a NH technical support workshop in South Africa (November 2015). Data were extracted from country presentations on national approaches and guidelines for disclosure of HIV status and transition to adult care.

**Results:**

All four countries reported initiating partial disclosure (discussing infection without specific naming of HIV) starting at age 4–8 years. Three countries reported full disclosure of HIV status by age 10 years, and three countries require full disclosure before initiating ART and transition to adult care. Among the four countries, only Kenya's National Adolescent Package of Care included standardized national tools for the transition to adult care. There are no national guidelines for the age of transition, but the reported standard practice ranges are all ≥15 years. All countries have national health strategies for adolescents; however, the focus on adolescents in the national paediatric, adult and consolidated (paediatric and adult) HIV guidelines varies greatly (Table).

**Conclusions:**

A standardized approach based on the best available evidence is needed to guide adolescent HIV care on a national level in resource-limited settings. Informed by this analysis, the NH collaborative is developing capacity building tools on disclosure and transition to adult care to be shared among national programs participating in NH activities, and globally.

**Abstract THAD0103–Table 1:** National HIV strategy and guidelines by country

Country	National adolescent health strategy	Adolescent focus in national HIV strategy/guidelines	Disclosure: partial (PD) full (FD)	Transition to adult care
Kenya	National Adolescent Package of Care, 2014	Integrated in adult guidelines as a separate chapter Referred to as “special population”	PD from 6 years FD by 13–16 years	By 19 years National transition algorithm and evaluation tools
Zambia	Adolescent Health Strategic Plan, 2011–2015; Adolescent Health Communication Strategy, 2013–2015	Included in consolidated paediatric and adult guidelines as Separate Population	PD from 5 years FD by 10 years	From 15 years
Swaziland	National Adolescent Strategic Plan, 2014–2018	Integrated in both paediatric and adult guidelines Separate section in paediatric guidelines “Special Considerations for Adolescents” to address consent, disclosure and psychosocial support	PD at 4–8 years FD at 8–10 years	By 21 years (19–21 years range)
Lesotho	National Adolescent Health Policy, 2012; Draft Adolescent Health Strategy, 2015	Included in consolidated paediatric and adult guidelines as Special Population	PD from 5 years FD from 10 years	By 20 years (15–18 years range)

# Sub article: THAD0104 A comparative study of policy and practice factors influencing progression through the HIV care continuum in Kisumu and Nairobi in Kenya

**Published:** 2016-07-22

**Authors:** C Cawley, S Oti, B Njamwea, A Nyaguara, F Odhiambo, FO Otieno, M Njage, T Shoham, K Church, J Todd, B Zaba, A Wringe, ALPHA (analysing Longitudinal Population-Based Hiv/aids Data on Africa) Network

**Affiliations:** 1 London School of Hygiene and Tropical Medicine, London, United Kingdom; 2 African Population and Health Research Center, Nairobi, Kenya; 3 KEMRI Center for Global Health Research, Kisumu, Kenya; 4 Nyanza Reproductive Health Society, Kisumu, Kenya; 5 Consultant, Nairobi, Kenya

## Abstract

**Introduction:**

The extent to which national HIV policies are implemented in health facilities in Africa has rarely been described. We describe HIV policy formulation with regards to HIV testing, and access to and retention in HIV care, and investigate the extent to which national HIV policies are implemented in health facilities serving the populations of two health and demographic surveillance sites (HDSSs) in Kenya (Nairobi and Kisumu).

**Methods:**

Twenty national HIV policy documents published between 2003 and 2013 were reviewed, with policy indicators extracted relating to HIV testing and counselling (HTC), prevention of mother-to-child transmission (PMTCT) and HIV care and treatment. Additionally, facility surveys were conducted in 44 HIV clinics (10 in Nairobi, 34 in Kisumu) serving the HDSS populations. Policy implementation across the HIV care continuum was assessed by comparing reported practices from health facility surveys with findings from the HIV policy review across pre-defined indicators that covered service coverage, quality of care, coordination of care, medical management and patient support.

**Results:**

Explicit policies existed for most aspects of HIV service delivery, and were widely implemented across all facilities, particularly indicators relating to access to treatment and retention in care. There were policy implementation gaps in relation to testing: national guidelines stated that key populations should have tailored access to HTC, but only 16/44 (36%) facilities offered HTC targeted at high-risk groups. In addition, frequent stock-outs of HIV test-kits were reported at 50% (5/10) of facilities in Nairobi and 70% (24/34) of facilities in Kisumu. Formulation and implementation of policy relating to Option B+ was weak, with only 20% of facilities in Nairobi (2/10) and 6% (2/34) of facilities in Kisumu offering this as the standard of care for HIV-positive pregnant women.

**Conclusions:**

Levels of policy implementation were similar in the two HDSS, and service performance in relation to ART access and retention in care was strong. However, weaknesses in relation to quality of care exist along the HIV care continuum, and service access problems were noted in relation to HIV testing. Health facilities are likely to require additional support to ensure delivery of future policies such as “test and treat”.

# Sub article: THAD0105 Barriers in access to health services for people living with HIV in Moldova

**Published:** 2016-07-22

**Authors:** S Bivol, V Catranji, L Caraulan

**Affiliations:** 1 Center for Health Policies and Studies, Chisinau, Republic of Moldova; 2 CBS AXA Agency, researcher, Chisinau, Republic of Moldova; 3 Center for Health Policies and Studies, HIV, Chisinau, Republic of Moldova

## Abstract

**Introduction:**

The purpose: to establish levels of access to health services of people living with HIV (PLHIV) through Tanahashi dimensions of access: availability, accessibility, acceptability, contact and effective coverage with services and compare them to general population (where available). Findings will be used to inform specific interventions to reduce barriers in access to services.

**Methods:**

Study design: quantitative cross-sectional study based on face-to-face interviews. Study population: 450 PLWH over 18 years, recruited through community groups and service provision points, sampling based on quotas regarding geographic distribution, age, gender.

**Results:**

The study included 45% women 55% men; mean age 36 years, 63% urban, 37% rural, 41% employed, 36% unemployed, and 23% economically inactive respondents. Geographic accessibility was high: 96% had access to a primary care doctor in their locality, lower in rural residents (44%). Financial accessibility was lower than in general population: 55% had health insurance versus 83% in the general population, main reason being unemployment (84%) and 56% had to renounce seeking health services due to anticipated costs, almost twice higher than in the general population (30%), with higher shares among women (61%), age over 40 years (62%) and urban residents (62%). General acceptability of general health services was high: positive attitudes at 86%, but only 30% of PLWH disclosed their HIV status to their last general health provider. Compared to general population, respondents seek health services more often, as 50% saw a health provider compared to 21% in the general population. Of them, 65% were prescribed medicines and 44% women and 52% did not buy prescribed medicines due to costs. Coverage with HIV-related treatment was high, as 97% saw an HIV provider and 74% were on ART.

**Conclusions:**

While access to HIV care and treatment was high, the study identified specific systemic barriers in access to general health services of PLWH compared to general population, such as higher economic barriers. These call for structural solutions, such as increasing access to health insurance and decreasing private expenditures for medicines, in order to increase effective coverage with health services overall.

# Sub article: THAD0106LB Engaging community stakeholders in preparation for HIV Vaccine Trials Network (HVTN) 703/HIV Prevention Trials Network (HPTN) 081, an antibody mediated biomedical HIV prevention trial in sub-Saharan Africa

**Published:** 2016-07-22

**Authors:** J Lucas, S Karuna, K Hinson, N Sista, N Yola, F Ntombela, N Mgodi, N Luthuli, S Wakefield, P Andrew, E Greene, S Delany-Moretlwe, G Gray, R White

**Affiliations:** 1 FHI 360, Durham, NC, United States; 2 HIV Vaccine Trials Network, Seattle, WA, United States; 3 Desmond Tutu HIV Foundation, Cape Town, South Africa; 4 CAPRISA, Durban, South Africa; 5 University of Zimbabwe (UZ-UCSF Collaboration), Harare, Zimbabwe; 6 HIV Vaccine Trials Network, Johannesburg, South Africa; 7 WRHI, Johannesburg, South Africa; 8 South African Medical Research Council, Cape Town, South Africa

## Abstract

**Introduction:**

HIV prevalence among heterosexual women in sub-Saharan Africa (SSA) continues to be highest in the world, with acquisition rates in some locations as high as 6–8% per year. Effective long-acting biomedical HIV prevention options, such as broadly neutralizing antibodies (bnAbs), could significantly reduce HIV incidence. HIV Vaccine Trials Network (HVTN) 703/HIV Prevention Trials Network (HPTN) 081 is a phase 2b study evaluating safety, tolerability, and HIV prevention efficacy of VRC01 (a monoclonal bnAb developed by the National Institutes of Health Vaccine Research Center) among heterosexual women in SSA. Engaging community stakeholders prior to study initiation fosters researcher-stakeholder partnerships and is essential to facilitate community awareness, understanding and support particularly for complex experimental biomedical studies.

**Description:**

In March 2016, the South African Medical Research Council (SAMRC), HPTN, and HVTN convened a stakeholder meeting with 85 attendees from seven SSA countries and the United States, including representatives from IRBs, community/research health clinics, traditional healing communities, governmental and non-governmental organizations, advocacy organizations and community advisory boards. The consultation facilitated diverse audience dialogue, detailed explanation of intricate biomedical concepts such as bnAbs and monoclonal antibody infusions and stakeholder questions and recommendations about HVTN 703/HPTN 081 implementation prior to initiation.

**Lessons learned:**

Based on experience and knowledge of community norms, attendees discussed facilitators and barriers to study participation, including understanding the complex concept of monoclonal antibodies. Interactive, participatory processes enabled stakeholders to express considerations regarding the women-only trial design and the evolving role of PrEP in HIV prevention efficacy trials in SSA. Researchers were encouraged to consider the feasibility of intravenous (IV) administration of VRC01. While HVTN 703/HPTN 081 is a proof of concept study, participants felt strongly that if VRC01 was found efficacious it should move to licensure while more efficient bnAb delivery systems (e.g. subcutaneous injections) and vaccines are explored.

**Conclusions/Next steps:**

The SAMRC, HPTN and HVTN recognize that trial success requires local implementers, potential participants and advocates as partners with trial designers. These partnerships facilitate early ownership of the research by key decision-makers and policy makers. This inclusion enables generous local insight into successful implementation of complex biomedical HIV prevention interventions and adds value of transparent, authentic dialogue about a wide range of trial considerations.

# Sub article: THAD0201 A randomized study of short-term conditional cash and food assistance to improve adherence to antiretroviral therapy among food insecure adults with HIV infection in Tanzania

**Published:** 2016-07-22

**Authors:** SI McCoy, P Njau, C Fahey, N Czaicki, N Kapologwe, S Kadiyala, W Dow, N Jewell, N Padian

**Affiliations:** 1 University of California, Berkeley, CA, United States; 2 Ministry of Health and Social Welfare, Dar es Salaam, Tanzania, United Republic of; 3 Health for a Prosperous Nation, Dar es Salaam, Tanzania, United Republic of; 4 Regional Medical Office, Shinyanga, Tanzania, United Republic of; 5 London School of Hygiene & Tropical Medicine, London, United Kingdom

## Abstract

**Introduction:**

Food insecurity is a barrier to antiretroviral therapy adherence and retention in care among people living with HIV infection (PLHIV). We evaluated the effectiveness of short-term cash and food assistance to mitigate food insecurity and improve adherence and retention among PLHIV in Shinyanga, Tanzania.

**Methods:**

At three HIV care and treatment facilities, 805 participants were randomized into one of three arms in a 3:3:1 ratio, stratified by site: nutrition assessment and counselling (NAC) plus cash transfers (~$11/month), NAC plus food basket and NAC only. Eligible participants were: 1) ≥18 years; 2) PLHIV; 3) initiated antiretroviral therapy ≤90 days before enrolment; and 4) food insecure, ascertained by the validated Household Hunger Scale. Cash or food transfers were provided for ≤6 months, conditional on visit attendance. Participants were followed for 6 months to determine treatment adherence measured by the medication possession ratio (MPR) and scheduled appointment adherence.

**Results:**

At enrolment, 64% of participants were female, average age was 37 years, mean CD4 count was 213 cells/ml, and mean body mass index was 21.4 kg/m^2^. Food security increased significantly among all participants at 6 months and was non-significantly higher among NAC+food (40%) and NAC+cash groups (41%) compared to NAC only (31%, p=0.41). Six-month adherence data were available for 789 participants (98% of study cohort). In an intent-to-treat analysis adjusted for site and multiple comparisons, MPR was significantly higher among those randomized to NAC+cash (n=347, MPR=83%) compared to NAC+food (n=338, MPR=78%, p=0.01) and NAC only (n=104, MPR=71%, p<0.01). Achievement of MPR≥95% was non-significantly higher in the NAC+cash group (57%) compared to the NAC+food group (50%, p=0.13) and NAC only group (46%, p=0.13). Appointment adherence was 81% overall and significantly higher among those randomized to NAC+cash (83%) compared to those in the NAC+food (75%, p<0.01) and NAC only (71%, p<0.01) groups.

**Conclusions:**

Preliminary data indicate that short-term conditional cash assistance may improve medication possession and appointment adherence better than food assistance and NAC alone among food-insecure PLHIV initiating treatment in Tanzania. Future studies are needed to investigate the optimal size and conditions of financial incentives, cost-effectiveness and whether benefits are sustained.

# Sub article: THAD0202 Economic empowerment of sex workers to improve their health, safety and wellbeing: innovative interventions and lessons learned from the Stepping Up, Stepping Out programme

**Published:** 2016-07-22

**Authors:** N Jagessar, L Papua, E Ndunda, M-L Wijne, S Hendriks

**Affiliations:** 1 AIDS Fonds, Sex Work Program, Amsterdam, Netherlands; 2 Organisasi Perubahan Sosial Indonesia (OPSI), Jakarta, Indonesia; 3 Health Options for Young Men on HIV, AIDS and STIs (HOYMAS), Nairobi, Kenya; 4 Netherlands Ministry of Foreign Affairs, The Hague, Netherlands

## Abstract

**Introduction:**

Worldwide countless women and men earn money through sex work. They are 12 times more likely to be living with HIV compared to the general population. However, sex workers who are more empowered to access additional sources of income and have more control over their financial resources, are better able to negotiate safe sex, extricate themselves from violent clients and access health and support services. It also improves their financial security and livelihood during times they cannot work.

**Description:**

As part of Aids Fonds' Stepping Up, Stepping Out (SUSO) programme, a variety of innovative economic empowerment interventions were developed by sex worker-led organizations in 11 countries in Africa, Asia and Latin-America, reaching over 2500 sex workers. One of the interventions is a savings and credit cooperation, giving sex workers the opportunity to save up and take loans for investments. The concept was implemented in Kenya by HOYMAS (Health Options for Young Men on HIV, Aids and STIs) and in Indonesia by OPSI (Organisasi Perubahan Sosial Indonesia).

**Lessons learned:**

In both countries the cooperative is run by sex workers. Funds can be accessed by sex workers who have 1) completed a financial skills training and 2) made regular savings in their account. The experience in Kenya showed that for sustainable results, developing the habit of saving proved more important than the amount saved. In Indonesia we learned that repayment schemes of loans will vary as not all types of investment become profitable equally fast. Not taking this reality into account could lead people into further debt. While in both countries the cooperation required focus and determination of the people involved, it provided them with an opportunity to turn their dreams into reality.

**Conclusions/Next steps:**

Economic empowerment is a powerful strategy to reduce sex workers' vulnerability to HIV. The following elements are essential (confirmed by the Dutch Radboud University that evaluated SUSO):

- Alternative sources of income outside the sex industry are not necessarily required. Earning more from sex work in better working conditions is also economic empowerment.
- Economic empowerment increases sex workers' self-esteem.
- Basic financial skills are a pre-condition for the success of other economic empowerment interventions.

# Sub article: THAD0203 Exploring the consequences of cash transfers for adolescent boys and girls in inner city Johannesburg

**Published:** 2016-07-22

**Authors:** N Khoza, J Stadler, C MacPhail, A Chikandiwa, H Brahmbhatt, S Delany-Moretlwe

**Affiliations:** 1 Faculty of Health Sciences, University of the Witwatersrand, Johannesburg, South Africa; 2 Collaborative Research Network for Mental Health and Wellbeing, University of New England, New South Wales, Australia; 3 Department of Population, Reproductive and Family Health, John Hopkins Bloomberg School of Public Health, Baltimore, MD, United States

## Abstract

**Introduction:**

Cash transfers (CTs) are increasingly being explored as a structural approach for HIV prevention. While there is much interest in expanding CT programmes to leverage health-related (including HIV) outcomes, there are few available data on the potential social consequences of offering CTs to adolescent boys and girls. This paper explores the consequences of CTs on adolescent recipients.

**Methods:**

Using qualitative data collected during a pilot randomized controlled trial of three different cash transfer strategies (monthly payments unconditioned vs. conditioned on school attendance vs. a single direct payment conditioned on a clinic visit) conducted in 120 consenting adolescents aged ≥16 years in the inner-city of Johannesburg. In-depth interviews were conducted with a sub-sample of 41 participants (18 girls and 23 boys), 6 months after receiving CT and up to 12 months after the cash was withdrawn. Interviews were conducted in English/isiZulu, transcribed and translated by two trained fieldworkers. Codes were generated using an inductive approach; initial transcripts were coded based on emerging issues, and subsequently transcripts coded deductively. Atlas-ti 7.5 was used to organize and code data.

**Results:**

Overall, CTs were highly acceptable to recipients; they were used for personal items and reduced household stress. This was interpreted by recipients as a sign of maturity and independence. There were, however, distinctive gender differences in the meaning adolescents placed on the CTs. Boys’ spending and saving patterns reflected a concern with maintaining their public social status, through which they asserted an image of the responsible adult. In contrast, girls’ spending and saving reflected domestic concerns. Some girls mentioned CTs as protective against transactional sexual relationships. Although generally regarded positively, adolescents reported some negative consequences of CTs, such as easier access to alcohol, cigarettes, recreational narcotics, and vulnerability to predatory moneylenders. While this was a concern raised by participants, it did not come out in the trials’ quantitative data.

**Conclusions:**

These data suggest that CTs benefit individuals as well as households in dense, urban environments, and may instil responsibility in young adults. However, negative consequences of CTs need to be monitored and interventions that address alcohol and drug use could be included in CT programmes.

# Sub article: THAD0204 Supervision, school and adolescent-sensitive clinic care: reducing unprotected sex among HIV-positive adolescents through combination social protection interventions

**Published:** 2016-07-22

**Authors:** E Toska, LD Cluver, ME Boyes, M Isaacsohn, R Hodes, L Sherr, Mzantsi Wakho Cohort Study

**Affiliations:** 1 Centre for Evidence-Based Intervention, Department of Social Policy & Intervention, University of Oxford, Oxford, United Kingdom; 2 University of Cape Town, AIDS and Society Research Unit, Centre for Social Science Research, Cape Town, South Africa; 3 Department of Psychiatry and Mental Health, University of Cape Town, Cape Town, South Africa; 4 Health Psychology and Behavioural Medicine Research Group, School of Psychology and Speech Pathology, Curtin University, Perth, Australia; 5 School of Medicine, Emory University, Atlanta, GA, United States; 6 University College London, London, United Kingdom

## Abstract

**Introduction:**

Each day, 440 new adolescent HIV-infections happen in sub-Saharan Africa: preventing onward transmission presents a powerful challenge to HIV-positive adolescents. There is very limited evidence on interventions that reduce sexual risk-taking among HIV-positive adolescents.

**Methods:**

Antiretroviral therapy (ART)-initiated adolescents (10–19 years old) from 53 government facilities in South Africa: 1059 were interviewed (90.1% of eligible sample, 4.1% refused, the rest excluded due to severe cognitive delays (0.9%), other reasons (1.2%) and could not be traced (3.7%)). Voluntary informed consent from adolescents and caregivers was obtained. Potential social protection included nine “cash/ cash-in-kind” and “care” provisions. Analyses used multivariate logistic regression, controlling for adolescent age, gender, location, informal housing, vertical/horizontal infection and caregiving arrangement. Potential interactive and additive effects of combinations were tested in logistic regressions and marginal effects models.

**Results:**

Adolescents reported high rates of unprotected sex: 18% in the full sample, and 28% among girls. In the final model, adolescents reported lower rates of unprotected sex if they received strong parental supervision (OR 0.61, CI: 0.38–0.98, p=0.041), had access to school (OR 0.53, CI: 0.35–0.81, p=0.003), and received adolescent-sensitive care when accessing sexual health services (OR 0.42, CI: 0.25–0.71, p=0.001). There were no interactive effects. In marginal effect models, receiving more than one social protection provision had an additive effect on predicted probabilities of unprotected sex rates, controlling for covariates (chart). This effect was even stronger among adolescent girls: without any interventions 49% were likely to report unprotected sex; with 1–2 of the interventions 14–38%; and with all interventions only 9%.

**Conclusions:**

These findings provide exciting new evidence that combinations of social protection intervention can increase safe sex among HIV-positive adolescents, particularly among HIV-positive adolescent girls. Single interventions may not suffice to address the sexual health needs of this highly vulnerable population as they transit from adolescence to adulthood.

**Abstract THAD0204–Figure 1:** Unprotected sex among HIV-positive adolescents by access to social protection interventions (controlling for socio-demographic co-factors).

# Sub article: THAD0205 Equity in adherence to antiretroviral therapy among economically-vulnerable adolescents living with HIV in Uganda

**Published:** 2016-07-22

**Authors:** L Gauer Bermudez, L Jennings, F Ssewamala, P Nabunya, C Mellins, M McKay

**Affiliations:** 1 International Center for Child Health and Asset Development, School of Social Work, Columbia University, New York, NY, United States; 2 Department of International Health, Social and Behavioral Interventions, Johns Hopkins Bloomberg School of Public Health, Baltimore, MD, United States; 3 University of Chicago School of Social Service Administration, Chicago, IL, United States; 4 HIV Center for Clinical and Behavioral Studies, New York State Psychiatric Institute & Columbia University Medical Center, New York, NY, United States; 5 Silver School of Social Work, McSilver Institute for Poverty Policy and Research, New York University, New York, NY, United States

## Abstract

**Introduction:**

Studies from sub-Saharan Africa indicate that children made vulnerable by poverty have been disproportionately affected by HIV with many exposed via mother-to-child transmission. Yet, for youth living with HIV, adherence to life saving treatment regimens are likely to be affected by a complex set of economic and social circumstances that challenge their families and exacerbate health problems.

**Methods:**

Using baseline data from the National Institute of Child and Human Development (NICHD) funded Suubi+Adherence study, bivariate and multivariate regression analyses were employed to examine the extent to which measures of economic and social equity were associated with self-reported adherence among Ugandan adolescents aged 10–16 (n=702) living with HIV.

**Results:**

Greater asset ownership, specifically familial possession of seven or more tangible assets, was associated with greater odds of self-reported adherence (OR 1.69, 95% CI: 1.00–2.85). Our analyses also indicated that distance to the nearest health clinic impacts youth's adherence to an ARV regimen. Youth who reported living nearest to a clinic were significantly more likely to report optimal adherence (OR 1.49, 95% CI: 0.92–2.40). Moreover, applying the composite equity scores, we found that adolescents with greater economic advantage in ownership of household assets, financial savings and caregiver employment had higher odds of adherence by a factor of 1.70 (95% CI: 1.07–2.70).

**Conclusions:**

These findings suggest that economic and social determinants of adherence be given due priority in the design and development of programmes affecting youth with HIV in sub-Saharan Africa. Specifically, interventions that aim to improve financial assets, enable participation in formal financial institutions and provide geographically closer HIV treatment services such as through mobile clinics may offer promising returns for greater equity in ARV uptake and adherence among adolescent populations living in low resource environments.

# Sub article: THAE0102 The implications of macroeconomic stability on achieving sustainable, domestic financing for HIV in Zambia

**Published:** 2016-07-22

**Authors:** T Fagan, C Zulu, W Zeng, V Menon

**Affiliations:** 1 Palladium, Health, Washington, DC, United States; 2 USAID, Lusaka, Zambia

## Abstract

**Introduction:**

Zambia's reclassification as a lower-middle-income country in 2011 underscored its need to begin transitioning to more sustainable, domestic sources of HIV financing. Nonetheless, at US$366 in 2015, donor contribution per people living with HIV infection (PLHIV) remains among the highest in sub-Saharan Africa. Donor funding for HIV in Zambia declined for the first time in 2015, a trend likely to continue as the Zambian government (GRZ) is asked to take greater ownership of the country's HIV response.

**Methods:**

The USAID- and PEPFAR-funded Health Policy project performed detailed secondary analysis of GRZ's budgets for 2012–2015. Two types of line items were allocated to the total budget for HIV: HIV-specific items, including anti-retroviral (ARV) procurement, HIV mainstreaming and awareness and the National AIDS Council, all allocated at 100%; and health systems line items, including salaries for health workers, administrative costs and development activities, all allocated at approximately 22% based on Clinton Health Access Initiative's estimates of the human resource requirement of current treatment targets.

**Results:**

Between 2012 and 2015, GRZ's contribution to Zambia's HIV response increased nominally from ZMW488 million to ZMW965 million. The budget for ARV procurement increased from ZMW50 million to ZMW226 million, while salaries for the proportion of health worker time (22%) allocated to HIV care and treatment grew from ZMW223 million to ZMW501 million. However, during this period, the kwacha (ZMW) depreciated by 60% against the US dollar from 5.14 ZMW/US$ to 8.62 ZMW/US$. In real terms, GRZ's HIV budget grew by just 5.6% annually from 2012 to 2015 and in fact declined by 26% between 2014 and 2015.

**Conclusions:**

Zambia's macroeconomic instability, driven largely by falling global prices for copper, which accounts for 10% of the country's GDP and 70% of export earnings, threatens the sustainability of domestic sources for HIV financing. Although total donor funding has plateaued since 2012, the resource requirement to reach the 90-90-90 targets is expected to grow from US$8 million in 2016 to US$144 million in 2020. Therefore, preserving the value of existing streams of financing and identifying new sources of domestic funding have never been more important to maintain and build upon the gains already made.

# Sub article: THAE0103 An HIV/AIDS investment case for Namibia: health impacts and resource needs for alternative programme scale up packages over 2016–2030

**Published:** 2016-07-22

**Authors:** E Korenromp, M Haacker, M Turay, K Rotich, M Strauss, T Barihuta, I Semini, J Stover, A-M Nitschke, N Forster, N Poku

**Affiliations:** 1 Avenir Health, Modelling and Policy Analysis, Geneva, Swaziland; 2 Harvard T.H. Chan School of Public Health, Boston, MA, United States; 3 UNAIDS Namibia, Windhoek, Namibia; 4 Health Economics and HIV and AIDS Research Division, University of KwaZulu-Natal, Durban, South Africa; 5 UNAIDS Southern Africa Regional Office, Johannesburg, South Africa; 6 Avenir Health, Modelling and Policy Analysis, Glastonbury, CT, United States; 7 Namibia Ministry of Health and Social Services, Windhoek, Namibia

## Abstract

**Introduction:**

As antiretroviral therapy (ART) coverage and cost grow while donor funding falls, Namibia must strategize its intervention package, to ensure sustainability and maximize impact. We projected impacts and costs over 2016–2030 for scenarios: Constant coverage at 2015 levels; National Strategic Framework (NSF) 2017 targets; Maximum scale-up aligned with UNAIDS *Fast Track* targets; and Resource-constrained optimization with and without technical efficiencies.

**Methods:**

The dynamic Spectrum-Goals model was fitted to surveillance, survey and programmatic data. Scenarios varied in coverage of FSW and MSM outreach, workplace intervention, ART (82% of CD4<500/μl from 2017 in NSF and Optimized scenarios, to 90% of all PLWH by 2030 in Maximum). Voluntary male medical circumcision, community mobilization, mass media, condom promotion, youth outreach, prevention of mother-to-child transmission (PMTCT) and post-exposure prophylaxis were scaled-up without variations across scenarios. Unit costs for HIV prevention and treatment services varied over time with economic context; programme support costs increased less-than-linearly with service costs. Maximum and technical efficiency scenarios assumed that viral suppression and infectivity reduction during ART improved from 75% at 2015 to 95% by 2030. Optimization followed intervention-specific cost per infection averted over 2015–2030.

**Results:**

Maximum and optimized scenarios with technical efficiency could reduce annual new infections by 50% over 2015–2030. Only maximum scale-up reduced annual HIV/AIDS deaths by almost 50% by 2030. Optimization with technical efficiencies, by prioritizing FSW and ART for lower-CD4 patients while rationalizing HIV testing and workforce prevention, reduced new infections by 35% at 2030, compared to NSF. Annual resource need increased from US$ 190 million in 2015, to US$ 231–252 million for NSF and Optimized, and US$ 257 million for Maximum by 2020.

**Conclusions:**

By optimizing the NSF, Namibia could considerably enhance health impacts while containing cost within expected budgets. Reaching *Fast Track* targets will require increasing (domestic) financing.

**Figure 1:** Namibia HIV infections impact from programme scale-up scenarios_03Feb2016.

# Sub article: THAE0104 Potential domestic source financing for scaled up antiretroviral therapy in 97 countries from 2015 to 2020

**Published:** 2016-07-22

**Authors:** A Dutta, C Barker

**Affiliations:** Palladium, Washington, DC, United States

## Abstract

**Introduction:**

Recent global initiatives such as 90-90-90 focus on rapidly scaling up antiretroviral therapy (ART). Substantial additional resources will be needed, and there is increased emphasis on shared responsibility and mobilizing domestic resources. Few studies have projected domestic resources available for ART specifically and the potential funding gap.

**Methods:**

In a study by the USAID-and-PEPFAR-funded Health Policy project, we estimated financial resources available to meet facility-level ART service delivery costs in 97 non-OECD countries with significant HIV epidemics based on country-specific funding trends from the Global Fund, PEPFAR and domestic contributions (DCs). DCs were based on publicly known country-reported procurement estimates, Global Fund's counterpart financing thresholds and proportional contributions to HIV responses as reported to UNAIDS. Separately, we modelled uncertainty in the annual resource requirements for antiretroviral drugs, laboratory tests and facility-level personnel and overhead, and compared the financial requirements to all sources of estimated funding.

**Results:**

We estimated that these countries can contribute US$6.2 billion in domestic resources to ART commodity procurement and US$13.5 billion for site-level overhead and personnel from 2015 to 2020 if ART eligibility is expanded to all people living with HIV, ART coverage increases in line with recent trends, and current funding obligations remain constant. Under optimistic assumptions, DCs could account for the majority of site-level resources available for ART in Eastern and Southern Africa (68%), Asia and the Pacific (88%), Eastern Europe and Central Asia (96%), Latin America and the Caribbean (51%) and the Middle East and North Africa (80%). West and Central Africa would see only 24% from these sources. The 6-year funding gap for reaching 90-90-90 is estimated to be US$24.2 to US$25 billion, depending on PEPFAR contributions, and the estimated commodity gap alone is US$16.8 billion.

**Conclusions:**

Additional resource mobilization from domestic or innovative financing sources or efficiency gain is needed to meet global ART targets.

**Abstract THAE0104–Figure 1:** Six-year ART financing by funding source and region.

# Sub article: THAE0105 Countries with concentrated epidemics among key populations still receive disproportionally lower PEPFAR COP funding than generalized epidemics

**Published:** 2016-07-22

**Authors:** L Lazar, A Grosso, G Millett, B Honermann, J Sherwood, J MacAllister, K Lindsey, C Chandra, S Blumenthal, C Lyons, S Baral

**Affiliations:** 1 Public Policy Office, amfAR, The Foundation for AIDS Research, Washington, DC, United States; 2 Public Health Solutions, New York, NY, United States; 3 Department of Epidemiology, Johns Hopkins School of Public Health, Baltimore, MD, United States

## Abstract

**Introduction:**

Men who have sex with men (MSM) and people who inject drugs (PWID) continue to be disproportionately affected by HIV. Previous work (Grosso et al., 2012) demonstrated that in 2009–2010, PEPFAR countries with concentrated epidemics in MSM and PWID received significantly less funding than countries with generalized epidemics. This analysis assesses changes in PEPFAR allocations from 2010 to 2014.

**Methods:**

Utilizing a previously published algorithm, countries (n=19) and regions (n=4) with available MSM and PWID HIV data that received PEPFAR funding within 2010–2014 were categorized epidemiologically; Category A: transmission primarily in MSM or PWID; Category B: transmission primarily in MSM, PWID and heterosexuals; Category C: generalized epidemic. PEPFAR funding data came from amfAR's Country Operational Plans (COPs) database. The sample comprises 67% of all COP funding. Multivariate regression analysis of overall funding by epidemic type was conducted, controlling for population, number of people living with HIV (PLHIV) and gross domestic product per capita.

**Results:**

From 2010 to 2014, relative funding to countries with mixed/concentrated epidemics increased from 2009 to 2010 baseline by 38% in Category A countries and 29% in Category B countries. However, Category A countries received $146 million less and Category B countries received $160 million less COP funding than Category C countries after adjusting for other factors. Category A and B countries received 9.9% of total COP funding to the sample despite comprising 26.4% of PLHIV among the sample. In 2014, Category C countries received 2.2 times more funding per PLHIV than Category A countries and 4.4 times more than Category B countries.

**Conclusions:**

While PEPFAR COP processes have made measurable improvements since 2010 to earmark key population (KP)-specific funds, allocations to countries with concentrated/mixed epidemics continue to be disproportionately lower. Greater transparency in funding decisions and increased proportionality of resource allocation, based on epidemiological evidence, will improve KP service delivery, accelerating progress towards HIV eradication.

**Abstract THAE0105–Figure 1:** Total PEPFAR funding per person living with HIV (PLHIV) by epidemic category.

# Sub article: THAE0106 HIV prevention research and development funding trends 2000–2015: tracking investment flows from research to rollout of new prevention technologies

**Published:** 2016-07-22

**Authors:** E Donaldson, D Mattur, K Fisher, T Harmon, P Harrison, JA Izazola-Licea, A Naeveke, M Warren

**Affiliations:** 1 AVAC, New York, NY, United States; 2 Joint United Nations Programme on HIV/AIDS (UNAIDS), Geneva, Switzerland; 3 International AIDS Vaccine Initiative (IAVI), New York, NY, United States

## Abstract

**Introduction:**

The HIV Vaccine & Microbicides resource tracking working group tracked year-to-year and long-term trends in research and development (R&D) investments and expenditures for biomedical HIV prevention, including HIV vaccines, microbicides, pre-exposure prophylaxis (PrEP), treatment as prevention and medical male circumcision from 2000 to 2015.

**Methods:**

R&D data were collected via annual surveys and direct outreach on disbursements by public, private and philanthropic funders for product development, clinical trials, trial preparation, community education and policy advocacy efforts to estimate annual investment in HIV prevention R&D. Investment trends were assessed and compared by year, prevention type, research phase, funder category and geographic location.

**Results:**

The working group collated and analyzed 2015 data for all areas of HIV prevention R&D. The group found that in 2015 overall investment in HIV prevention research reflected slight increases in US and private sector funding. With two phase II vaccine trials starting in 2015 funding for vaccines increased marginally, while funding towards microbicides decreased as ongoing efficacy trials began to wind down. In 2014, the working group began collecting data on PrEP implementation research and funding increased from 2014 to 2015 with several demonstration projects beginning in 2015.

**Conclusions:**

Overall, fewer individual funders supported HIV prevention research than in previous years, with the US public sector and the Bill & Melinda Gates foundation accounting for over 80% of all funding. Expanding and diversifying the investment base could provide a critical range of perspectives, human capacity and innovative concepts to the HIV prevention research agenda. With a shift in the funding formula for AIDS at the US NIH and adoption of Sustainable Development Goals focused on ending AIDS as part of a public health approach, it is critical to ensure continued prioritization of HIV prevention R&D on the US and global development agenda by evaluating research in the context of public, private and philanthropic funding. Mapping of funding trends is critical as HIV prevention R&D progresses through the pipeline from research to rollout to identify investment needs, prioritize research areas, assess impact of public policies that affect spending levels and provide the fact base for advocacy to sustain investments.

# Sub article: THAE0202 System-level barriers to FP-HIV integration in Malawi

**Published:** 2016-07-22

**Authors:** L Irani, E McGinn, M Mellish, O Mtema, P Dindi

**Affiliations:** 1 UNICEF, Gender Section, New York, NY, United States; 2 Palladium, Health Policy Plus Project, Washington, DC, United States; 3 Palladium, Health Policy Plus Project, Lilongwe, Malawi

## Abstract

**Introduction:**

Malawi has several policies for integrating family planning (FP) and HIV services. The 2011 national HIV clinical management guidelines recommended provider-initiated FP counseling (PIFP) and provision of condoms and injectables at ART settings. The USAID- and PEPFAR-funded Health Policy Project's 2015 study assessed the extent of integration occurring at the facility level, with special attention to identifying systems-level barriers.

**Methods:**

Data were collected from a purposive sample of 41 public and private facilities across nine districts of Malawi. Facilities ranged from large high-volume hospitals to small health posts. Data collectors conducted 41 facility audits, 41 interviews with facility in-charges, 122 interviews with providers and 425 client exit interviews.

**Results:**

While 85% of ART clinics had condoms available, only 31% had injectables on hand; only 20% had a range of four or more types of contraceptives available. Fewer than half of the ART registers were tracking FP provision; the rest either kept separate registers or had no mechanism for documenting FP provision. About one-fifth of providers working at these facilities had no FP training, and only one-quarter had received any training on FP-HIV integration specifically. Although 93% of providers said they had time to counsel ART clients on FP, only 14% of clients reported being asked about their fertility intentions or FP on that day's visit. Even though providers reported referring out for FP, few knew detailed information about where and when those other services could be accessed. Clinic hours and provider availability were also identified as hindering FP service provision for ART clients. Almost half (44%) of the facilities reported stockouts or expirations of FP commodities in the past three months; one-third reported stockouts of HCT kits and one-quarter reported ARV stockouts.

**Conclusions:**

Although national policies support FP-HIV integration, systems at facility level are not yet adequate to fully implement integration. The study and analysis offer recommendations for how facilities can improve their organization of services, strengthen both internal and external referral processes, increase training of providers on PIFP, improve patient registers and other M&E systems to better capture data and address both FP and HIV commodity and supply stockouts.

# Sub article: THAE0203 Evaluating the costs and efficiency of integrating family planning services into HIV and AIDS treatment services in Zambia

**Published:** 2016-07-22

**Authors:** S Faye, B Johns, E Baruwa, K Ambrose

**Affiliations:** Abt Associates Inc, International Health, Bethesda, MD, United States

## Abstract

**Introduction:**

Integrating HIV and AIDS services with other health services is a key strategy for achieving an AIDS-free generation. In particular, integrating family planning (FP) and HIV services can improve health outcomes and continuity of care, and make service delivery more sustainable by supporting the efficient utilization of resources. At the request of USAID's Office of HIV/AIDS and the USAID Zambia mission, the Health Finance and Governance project used quantitative indicators to assess the costs and efficiencies of two models of FP and ART service integration in Zambia.

**Methods:**

We conducted a cross-sectional, non-randomized comparison of two integration models – “internal referral” (IR), where patients can be counselled on FP within the ART clinic but are referred to the FP clinic onsite for further services, and “one-stop-shop” (OSS), where patients can be counselled and receive an FP method within the ART clinic. The models were compared using three indicators of efficiency: percentage of missed FP opportunities at ART clinics, time spent counseling ART patients on FP, and unit cost per ART patient counselled on FP and given an FP method. Data were collected from health management information systems, patient files and exit interviews at ten sites in Zambia for the period from October 2013 to September 2014.

**Results:**

The study found no statistically significant difference in efficiency between OSS and IR models for any of the proposed indicators, including cost. Additional costs of FP provision were US$3 on average per patient using OSS, and USD$8 on average per patient using IR. FP counseling added an average of 3 minutes to ART consultation time (p=0.03), but there was no statistically significant difference in that added time between the two models (p=0.65). There was widespread variation in the practice of integration among sites and models. Weak referral systems and poor client tracking limited potential integration gains.

**Conclusions:**

Providing a comprehensive package of ART and FP services to HIV-positive women costs relatively little regardless of the integration model used. However, improved referral and client tracking systems could increase efficiency. Additional time and effort is required for facilities to consistently collect data on efficiency, referrals and client tracking.

# Sub article: THAE0204 Screening for hypertension and diabetes at the time of HIV testing in Umlazi Township, Durban, South Africa

**Published:** 2016-07-22

**Authors:** I Bassett, T Hong, P Drain, S Govere, H Thulare, M Krows, M-Y Moosa, B Mhlongo, D Wexler, M Huang, S Frank, E Hyle, R Parker

**Affiliations:** 1 Department of Medicine/Division of Infectious Disease, Massachusetts General Hospital, Boston, MA, United States; 2 Department of Medicine, Medical Practice Evaluation Center, Massachusetts General Hospital, Boston, MA, United States; 3 Harvard University Center for AIDS Research (CFAR), Boston, MA, United States; 4 University of Washington, Seattle, WA, United States; 5 AIDS Healthcare Foundation/Ithembalabantu Clinic, Durban, South Africa; 6 Department of Infectious Disease, School of Clinical Medicine, University of KwaZulu Natal, Durban, South Africa; 7 Department of Medicine, Diabetes Unit, Massachusetts General Hospital Boston, MA, United States; 8 Harvard Medical School, Boston, MA, United States; 9 Division of General Medicine, Massachusetts General Hospital Boston, MA, United States; 10 Biostatistics Center, Massachusetts General Hospital, Boston, MA, United States

## Abstract

**Introduction:**

The South African HIV treatment guidelines recommend screening for hypertension and diabetes before initiation of antiretroviral therapy. Our objective was to assess the prevalence and risk factors for hypertension and diabetes at HIV testing in Durban.

**Methods:**

We enrolled adults (≥18 years) presenting for voluntary HIV testing at a high-volume clinic in Umlazi Township. We asked about mode of transport to clinic (walk, public bus/taxi, car) and measured a seated blood pressure before rapid HIV testing. Among those HIV-infected, we measured height, weight and random glucose by point-of-care glucometer. We defined hypertension as systolic blood pressure ≥140 mmHg or diastolic blood pressure ≥90 mmHg. We defined likely diabetes as random blood glucose ≥11.1 mmol/l and likely impaired glucose tolerance (IGT) or prediabetes as ≥7.8 to <11.1 mmol/l (based on International Diabetes Federation definition 2 hours post a 75 g oral glucose load). We defined obesity as body mass index ≥30 kg/m^2^. We used separate multivariate logistic regression models to determine risk factors for hypertension and IGT/diabetes among HIV-infected participants.

**Results:**

Among 3082 enrollees, 1572 (51%) were female and 1170 (38%) were HIV-infected, with median CD4 count 299/µl (IQR 154–459). The majority (78%) walked to clinic; 22% took a public bus/taxi and <1% arrived by car. HIV-uninfected participants were less likely to have hypertension (6%, 118/1799) compared to HIV-infected participants (13%, 157/1,153, p<0.001). Among HIV-infected participants, 4% (21/492) met criteria for likely IGT or diabetes and 20% (100/492) were obese. Adjusting for gender, age≥35 (OR 2.5, 95% CI: 1.4–4.4, compared age<25) and BMI≥30 (OR 2.1, 95% CI: 1.4–3.1) were associated with hypertension. Adjusting for obesity, not walking to clinic (OR 2.9, 95% CI 1.2-7.1, as compared to walking) and hypertension (OR: 2.8, 95% CI 1.0–7.5) were associated with IGT/diabetes.

**Conclusions:**

Screening for hypertension and diabetes was high-yield in a Durban HIV clinic. Traveling to clinic by public bus/taxi or car instead of walking, which may be a marker for low physical activity, was associated with nearly triple the odds of IGT/diabetes. Screening for chronic non-communicable diseases can be successfully performed at the time of HIV testing in resource-limited settings.

# Sub article: THAE0205 One-stop shopping for TB and HIV services improved initiation of antiretroviral therapy for patients who are co-infected in eastern Uganda

**Published:** 2016-07-22

**Authors:** A Mukuye, N Lukoda, B Crandall

**Affiliations:** Management Sciences for Health, Kampala, Uganda

## Abstract

**Introduction:**

Initiation of antiretroviral therapy (ART) for patients co-infected with HIV and TB is low in certain districts of Eastern Uganda. One way to improve this is a one-stop shop where TB and HIV co-infected patients access a full package of services at one location.

**Description:**

Strengthening TB and HIV & AIDS Responses in Eastern Uganda (STAR-E), a USAID project funded by PEPFAR and implemented by Management Sciences for Health, supported a health facility-based intervention in TB treatment sites in Kapchorwa District between January and September 2015.

The project provided on-site mentorship to 15 health workers at 5 health facilities on implementing a “one-stop shop” (OSS) model for treating TB/HIV co-infected patients. Health workers from the TB clinic, ART clinic and mother-baby care point were mentored on the Uganda national guidelines for treating TB/HIV co-infection, and given job aids, guidelines and diagnostic charts. The health workers then mentored others. Data was collected at baseline and during implementation.

**Lessons learned:**

Following establishment of the OSS approach, the percent of TB/HIV co-infected patients started on ART increased from 42 to 100% (see Table).

Provision of TB/HIV co-infected treatment services with the one-stop shop approach increased co-infected patients' initiation of ART.

**Conclusions/Next steps:**

Continued implementation of vertical HIV/AIDS and TB programmes that treat each disease separately is inadequate and should be replaced by new models of care that integrate services and maximize efficient use of already-limited resources. This intervention indicates integration may be effective for timely initiation of ART amongst TB patients in a low resource rural African setting. Scale up of this approach to health facilities providing TB/HIV services should be considered.

**Abstract THAE0205–Table 1:** TB/HIV treatment – October 2014 to September 2015

Quarter	Total cases detected	Tested for HIV	% who tested	HIV +	% HIV +	CPT	% on CPT	ARV	% on ART
Oct–Dec 2014	30	28	93	19	67.9	19	100	8	42
Jan–Mar 2015	32	32	100	16	50.0	16	100	14	88
Apr–Jun 2015	23	23	100	15	65.2	15	100	13	87
Jul–Sep 2015	29	29	100	13	44.8	13	100	13	100

# Sub article: THAE0206 Promising practice: integrating gender and gender-based violence into community-based organizations capacity building, HIV prevention, counselling and testing programmes

**Published:** 2016-07-22

**Authors:** H Bryant, E Oliveras, E Colua, E Marinda

**Affiliations:** 1 FHI 360, Social and Economic Development, Washington, DC, United States; 2 FHI 360, Mozambique Country Office, Maputo, Mozambique; 3 FHI 360, M&E, Maputo, Mozambique; 4 Health Information Matrix, Johannesburg, South Africa

## Abstract

**Introduction:**

In Mozambique, HIV prevalence is 13.1% for women, 9.2% for men, one in three women experience physical violence; 12% of women over 15 experience sexual violence. Risk factors include early marriage, transactional sex and male dominance in decision making. Under the PEPFAR Gender-based Violence Initiative, USAID/Mozambique supported the Capable Partners Program (CAP) to scale up GBV prevention within their capacity building programme. CAP and the Health Policy Project provided training and technical assistance to help six CBOs design and implement social and behavioural change communication activities that address gender norms/GBV and HIV together.

CBOs organized series of 8–12 small group debates addressing gender-based risk factors and barriers to HIV prevention and testing. CAP developed videos to spur meaningful debates and ensured quality activities. Interventions aimed at preventing sexual transmission of HIV and promoting HIV testing reached 70,892 women and men aged 15–49 in four provinces during 2012–2015.

**Methods:**

A 2014 quantitative cross-sectional endline population survey interviewed 1531 men and women aged 15–49 about gender norms and testing. Propensity score matching compared people exposed to CAP to those not exposed.

**Results:**

Among the exposed group, 21% agreed it is acceptable for men to make all decisions for the family without including the wife, versus 33% among the unexposed. The exposed were less likely to think it acceptable for teachers to request sex from their students (12% vs. 24%) and to think that men can have sex with girls younger than 14 (16% vs. 26%). Furthermore, 32% of the exposed indicated that they tested with their sexual partner compared to 5% of the unexposed (p<0.01). Qualitative results indicate a strong, but not yet pervasive, effect on awareness about the legal framework and GBV reporting.

**Conclusions:**

Community-based interventions that integrate GBV with HIV prevention are positively associated with more balanced community gender norms and lead to increased preventive behaviors.

# Sub article: THAE0301 Estimating country cost implications associated with new WHO HIV treatment guideline revisions: forecasting Cambodia's 5-year programme costs for adults

**Published:** 2016-07-22

**Authors:** JR Campbell, K Gustafson, P Jalan, B Ngauv, S Sovannarith, A Wilhelm, S Sopheap, C Vichea, C Middlecote, LP Sun

**Affiliations:** 1 Clinton Health Access Initiative, Applied Analytics, Boston, MA, United States; 2 Clinton Health Access Initiative, Phnom Penh, Cambodia; 3 National Centre for HIV/AIDS, Dermatology, and STDs, Ministry of Health, Phnom Penh, Cambodia; 4 Clinton Health Access Initiative, Health Access Program, London, United Kingdom

## Abstract

**Introduction:**

HIV treatment coverage rates in Cambodia are high. Cambodia's National Centre for HIV/AIDS, Dermatology, and STD (NCHADS) wanted to understand the cost and feasibility of expanding the national HIV treatment programme to include eligibility for all HIV-positive patients regardless of CD4 count and expanding access to viral load (VL) monitoring. In order to optimize both available resources and treatment quality, the financial impact of adopting the 2015 World Health Organization HIV treatment guidelines must be considered. They also wanted to explore the impact of new technologies and antiretroviral drugs (ARVs) on HIV treatment programme costs and drug transitions.

**Methods:**

We used a five-year forecast Excel-based morbidity model, to run five comparison scenarios side-by-side evaluating different treatment policy decisions:

1. baseline of the current treatment eligibility at CD4≤350 copies/ml and accounting for key populations;
2. increased treatment eligibility to CD4≤500 copies/ml;
3. increased treatment eligibility to treat-all;
4. treat-all and reduced CD4 monitoring;
5. treat-all, reduced CD4 monitoring, and initiating and gradually transition eligible efavirenz (EFV) patients to low-dose EFV.

All scenarios had an increase in VL coverage. Cambodian HIV epidemic and programme data were used to estimate patient numbers and costs for first-line and second-line ARVs and buffer stocks, labs and human resources. Patient years were disaggregated by patient types and assigning different treatment statuses such as pre-ART, PMTCT, newly initiating patients, stable patients and non-stable patients.

**Results:**

Estimated patient-years on treatment increased by 8% when adopting treat-all, with a large jump in the first year with additional pre-ART patients initiating treatment. This surge resulted in approximately $5 million cost increase over five years. Savings were found in reducing CD4 monitoring ($2.6 million) and further savings with transitions to low-dose EFV ($2.4 million), netting lower five-year costs with these shifts compared to the baseline scenario. By year five, 45% of first-line patients are on low-dose EFV regimens, the cost per patient year decreases from $260 to $233 in scenario-five compared to scenario-three.

**Conclusions:**

NCHADS used these results in consideration for their new guideline revisions. They decided that treat-all was feasible and would be adopted, and low-dose EFV would be incorporated into the programme in lieu of EFV for eligible patients.

# Sub article: THAE0302 Anticipated reductions in long-term tuberculosis incidence and associated cost savings with adoption of the treat all people living with HIV policy in Botswana, 2016–2035

**Published:** 2016-07-22

**Authors:** B Kgwaadira, T Katlholo, L Fiebig, R Boyd, M Wame, L Kuate, D Agegnehu, W Dikobe, N Sangrujee, C Petlo, B Nkomo, A Avalos, M Skiles, H Phillips, T Wuhib, A Finlay-Vickers

**Affiliations:** 1 Botswana National Tuberculosis and Leprosy Programme, Ministry of Health, Gaborone, Botswana; 2 Robert Koch Institute, Berlin, Germany; 3 US Centers for Disease Control and Prevention, Gaborone, Botswana; 4 Careena Centre for Health, Gaborone, Botswana; 5 KNCV, Gaborone, Botswana; 6 US Centers for Disease Control and Prevention, Atlanta, GA, United States; 7 epartment of AIDS Prevention and Care, Ministry of Health, Gaborone, Botswana; 8 US Agency for International Development, Gaborone, Botswana; 9 UNAIDS, Gaborone, Botswana

## Abstract

**Introduction:**

Botswana is a high tuberculosis (TB)/HIV burden country with the second highest HIV prevalence in the world of 18.5% in the general population, an estimated TB incidence of 385 cases per 100,000 population and TB/HIV co-infection rate of 60% in 2014. In 2012, Botswana expanded antiretroviral therapy(ART) eligibility to include all people living with HIV (PLHIV) with a CD4 count of <350 cells/µl^3^ or WHO clinical Stage 3/4 disease. To inform the national decision to increase coverage by providing ART to all PLHIV (Treat All policy), we modeled the expected reductions in TB incidence and resulting TB-related cost savings from 2016–2035 comparing 1) a baseline scenario with current ART eligibility and HIV prevention coverage levels to 2) a Treat All scenario for PLHIV.

**Methods:**

The HIV Spectrum Model was used to generate annual estimates of the number of PLHIV on ART and not on ART for each scenario from 2016 to 2035. TB and multidrug-resistant TB (MDR-TB) incidence and differential TB risk was modeled for each scenario; annual numbers of expected TB and MDR-TB cases among PLHIV were calculated. Base costs per TB and MDR-TB case were estimated from the cost of anti-TB treatment drugs and laboratory tests for diagnosis and clinical monitoring per national TB guidelines.

**Results:**

Under the baseline scenario the annual number of TB cases among PLHIV would increase through 2035. Adopting a Treat All policy in Botswana would potentially prevent 71,862 TB/HIV cases, including 2605 MDR-TB/HIV cases, resulting in cumulative TB cost savings of over $40 million from 2016 to 2035. By 2035, we predict Treat All could reduce TB incidence from an estimated 1321/100,000 among PLHIV (baseline) in Botswana to 568/100,000 (Treat All) and contribute a 36% reduction in overall TB incidence between 2015 and 2035.

**Conclusions:**

While our projection is subject to several limitations, sensitivity analysis suggests that a marked reduction in TB incidence is robust. Immediate adoption of a Treat All policy in Botswana would be an important, effective TB prevention and control intervention. Additional TB control strategies will be needed to meet the End TB milestone of reducing TB incidence by 95% by 2035.

# Sub article: THAE0303 Assessing progress, impact and next steps in rolling out voluntary medical male circumcision for HIV prevention in fourteen priority countries in eastern and southern Africa as of 2015

**Published:** 2016-07-22

**Authors:** P Stegman, K Kripke, E Njeuhmeli, J Samuelson, M Schnure, S Dalal, T Farley, C Hankins, A Thomas, J Reed, N Bock

**Affiliations:** 1 Project SOAR, Avenir Health, Washington, DC, United States; 2 United States Agency for International Development (USAID), Washington, DC, United States; 3 World Health Organization, Geneva, Switzerland; 4 Palladium Group, Washington, DC, United States; 5 Sigma3 Services, Nyon, Switzerland; 6 Amsterdam Insititue for Global Health and Development, Amsterdam, Netherlands; 7 US Department of Defence, Naval Health Research Center, San Diego, CA, United States; 8 Jhpiego, Washington, DC, United States; 9 U.S. Centers for Disease Control and Prevention, Atlanta, GA, United States

## Abstract

**Introduction:**

In 2007, the World Health Organization and the Joint United Nations Programme on HIV/AIDS (UNAIDS) identified 14 priority countries across eastern and southern Africa for scaling up voluntary medical male circumcision (VMMC) services. Several years into this scale-up effort, we reflect on progress made thus far.

**Methods:**

Using the Decision-Makers' Program Planning Tool (DMPPT) 2.1, we assessed the age-specific impact, cost-effectiveness and coverage attributable to circumcisions performed through end 2014. The analysis also compared impact of actual progress to that of achieving 80% coverage among men aged 15–49 in 12 VMMC priority countries and Nyanza Province, Kenya. The models were populated with age-disaggregated VMMC service statistics, and with population, mortality and HIV incidence and prevalence projections exported from country-specific Spectrum/Goals files, assuming achievement in each country of the new 90-90-90 treatment targets.

**Results:**

Over 9 million VMMCs had been conducted through 2014: 43% of the estimated 20.9 million VMMCs required to reach 80% coverage by end 2015. Assuming each country reaches the 90-90-90 HIV treatment targets, the modelling analysis projected that VMMCs conducted through 2014 will avert a total of 240,000 infections by 2025, compared to 1.1 million if each country had reached 80% coverage by 2015. The median estimated cost per HIV infection averted was $4400. Nyanza province in Kenya, the 11 priority regions in Tanzania, and Uganda have reached or are approaching MC coverage targets among males ages 15–24, while coverage in other age groups is lower. Across all countries modelled, over 50% of the projected HIV infections averted were attributable to circumcising the 10- to 19-year-olds.

**Conclusions:**

The priority countries have made considerable progress in VMMC scale-up, and VMMC remains a cost-effective strategy for epidemic impact, even assuming near-universal HIV diagnosis, treatment coverage and viral suppression. Examining circumcision coverage by 5-year age groups will provide countries with better insights into the progress of their VMMC programmes and help them to make more informed decisions about next steps.

# Sub article: THAE0304 Cost effectiveness of on demand PrEP in men who have sex with men (MSM) in the ANRS IPERGAY study

**Published:** 2016-07-22

**Authors:** I Durand-Zaleski, P Mutuon, I Charreau, C Temblay, D Rojas, J Chas, C Chidiac, C Capitant, B Spire, L Meyer, JM Molina, ANRS Ipergay

**Affiliations:** 1 Assistance Publique Hopitaux de Paris, Public Health, Paris, France; 2 INSERM, Paris, France; 3 Université de Montréal, Montreal, Canada; 4 AIDES, Paris, France; 5 Assistance Publique Hopitaux de Paris, Infectious Diseases, Paris, France; 6 Hospitalier et Universitaire de Lyon, Lyon, France

## Abstract

**Introduction:**

ANRS IPERGAY showed the efficacy of on demand PrEP with TDF/FTC in preventing HIV acquisition among MSM.

**Methods:**

A prospective economic evaluation was performed during the trial from the healthcare system perspective to determine the cost of PrEP per HIV-infection averted in the TDF/FTC arm. Both hospital and non-hospital resources were considered. Costs for counseling were added. Drugs (TDF/FTC and drugs for STIs), tests (for HIV and STIs), visits and hospital admissions were valued with the national tariff and based on their mean use during the trial (15 tablets of TDF/FTC per month). Robustness of results was tested by sensitivity analyses. The incremental cost-effectiveness ratio (ICER) of PrEP per HIV-infection averted was calculated for one year and compared to the yearly and lifetime cost of one HIV-infection in France (€20,170 and €535,000 respectively).

**Results:**

The trial enrolled 400 participants and found that the number needed to treat for one year to prevent one HIV-infection was 17.6. The cost of counseling was 690 € per person-year. The total one-year costs of PrEP were €4004 per participant, of which 78% were drug costs (€500 for 30 tablets of TDF/FTC). PrEP ICER was €70,470 per infection averted. Using TDF/FTC costs of €60 for 30 tablets, the one-year cost was €1253 per patient and the ICER was €22,052 per infection averted, similar to the yearly cost of treating HIV-infection. Sensitivity analyses in Figure 1 show the contrubtion of drug costs and NNT results on the ICER.

**Conclusions:**

In France, the ICER of on demand PrEP in MSM with TDF/FTC at the current price is higher than the cost of treating a patient with HIV infection for one year but much lower than the lifetime cost of HIV infection. Using the lower cost of TDF/FTC however, PrEP becomes cost-neutral on a yearly basis.

**Figure 1:** Sensitivity analysis of the ICER at current and low drug prices.

# Sub article: THAE0305 The cost-effectiveness of HIV pre-exposure prophylaxis (PrEP) in high-risk men who have sex with men (MSM) and transgendered women (TGW) in Brazil

**Published:** 2016-07-22

**Authors:** PM Luz, B Osher, B Grinsztejn, RL MacLean, E Losina, CJ Struchiner, RA Parker, KA Freedberg, F Mesquita, RP Walensky, VG Veloso, AD Paltiel

**Affiliations:** 1 National Institute of Infectious Diseases, Oswaldo Cruz Foundation, Rio de Janeiro, Brazil; 2 Department of MedicineMedical Practice Evaluation Center, Massachusetts General Hospital, Boston, MA, United States; 3 Harvard University, Center for AIDS Research, Boston, MA, United States; 4 Divison of Rheumatology, Department of Medicine, Department of Orthopedic Surgery, Brigham and Women's Hospital, Boston, MA, United States; 5 Massachusetts General Hospital, Division of General Medicine, Medical Practice Evaluation Center, Department of Medicine, Biostatistics Center, Boston, MA, United States; 6 Harvard Medical School, Boston, MA, United States; 7 Massachusetts General Hospital, Division of General Medicine, Medical Practice Evaluation Center, Department of Medicine, Division of Infectious Disease, Boston, MA, United States; 8 Harvard University, Center for AIDS Research, Department of Epidemiology, Department of Health Policy and Management, Boston, MA, United States; 9 Boston University School of Public Health, Department of Biostatistics, Department of Epidemiology, Boston, MA, United States; 10 World Health Organization (WHO), Brasilia, Brazil; 11 Brigham and Women's Hospital, Division of Infectious Disease, Boston, MA, United States; 12 Yale School of Public Health, New Haven, United States

## Abstract

**Background:**

The effectiveness of tenofovir-based oral PrEP for preventing HIV infection has ranged from 44–96% in clinical trials. We examined the cost-effectiveness of PrEP in MSM and TGW in Brazil.

**Methods:**

We used the CEPAC-International model of HIV prevention and treatment to simulate clinical outcomes, costs and cost-effectiveness of daily TDF-FTC PrEP among high-risk MSM and TGW in Brazil. Our comparator, *no PrEP*, featured guideline-concordant care, including universal ART access. In the *PrEP* strategy, high-risk HIV-negative adults age<40 years received daily PrEP, HIV testing every 4 months and annual creatinine. Base case parameters, derived from Brazil-specific sources, included mean age (31 years), annual HIV incidence (age≤40 years: 4.3/100PY; age>macr;40 years: 1.0/100PY), PrEP efficacy (86%), PrEP drug costs ($12.50/m) and PrEP programme costs ($0.99/m). We varied key parameters in sensitivity analyses.

**Results:**

Compared to *no PrEP*, *PrEP* decreased lifetime HIV infection risk by 37%. *PrEP* increased per person discounted (undiscounted) survival from 20.7 (36.9) to 22.4 (41.1) years and decreased lifetime medical costs from $4090 ($10,910) to $3470 ($7660); *PrEP* was therefore cost-saving (Figure 1). *PrEP* remained cost-saving under key parameter variation, including PrEP cost, initial cohort age and HIV testing frequency on/off PrEP. When PrEP was only used until age 30, PrEP ceased to be cost-saving, but its incremental cost-effectiveness ratio (ICER) remained<1x Brazil's per capita GDP. The ICER of *PrEP* also remained cost-saving or<1xGDP when PrEP efficacy and HIV incidence varied widely (Figure 1), but exceeded 1xGDP when HIV incidence was ≤ 0.24 infections/100PY at base case PrEP efficacy (86%).

**Conclusions:**

PrEP is cost-saving for MSM and TGW in Brazil. Our results strengthen local PrEP demonstration project results and offer justification for a future national PrEP programme for MSM/TGW in Brazil.

**Figure 1:** Two-way sensitivity analysis of PrEP efficacy and incidence.

**Abstract THAE0305–Table 1:** Base case results of an analysis of PrEP cost-effectiveness in Brazil

Strategy	Total cohort cost/patient	Total cohort LE/patient	HIV+ cases (% of cohort)	% reduction in lifetime HIV infection risk	Average years to infection (SD)
No PrEP	$4090	20.7	50.07	–	13.7 (13.7)
PrEP	$3470	22.4	31.57	37	24.2 (15.8)

All costs discounted at 3%/year; PrEP: pre-exposure prophylaxis; LE: life expectancy; HIV+: HIV-positive; SD: standard deviation

# Sub article: THAX0101 Identifying patterns of HIV-1 transmission among MSM communities in Japan for target selection of an active prevention programme

**Published:** 2016-07-22

**Authors:** T Shiino, J Hattori, K Sadamasu, M Nagashima, A Hachiya, W Sugiura, K Yoshimura, the Japanese Drug Resistance HIV-1 Surveillance Network

**Affiliations:** 1 Infectious Disease Surveillance Center, National Institute of Infectious Diseases, Shinjuku-ku, Japan; 2 National Cancer Institute, National Institutes of Health, Frederick, MD, United States; 3 Department of Microbiology, Tokyo Metropolitan Institute of Public Health, Shinjuku-ku, Japan; 4 Clinical Research Center, Nagoya Medical Center, Nagoya, Japan; 5 Glaxo Smith Kline K.K, Tokyo, Japan; 6 AIDS Research Center, National Institute of Infectious Diseases, Shinjuku-ku, Japan

## Abstract

**Introduction:**

Understanding the transmission dynamics of HIV-1 among a risk population can provide clues for developing an efficient prevention strategy. In order to characterize the transmission dynamics of HIV-1 spread among men who have sex with men (MSM) in Japan, we conducted sequence-based transmission clustering using advanced phylogenetic inferences followed by a network analysis.

**Methods:**

Protease-reverse transcriptase sequences from 4386 subtype B-infected individuals registered in the Japanese Drug Resistance HIV-1 Surveillance Network between 2003 and 2012 were included in the analysis. The number of patients infected within 6 months was estimated using a BED assay for plasma samples. Phylogenetic relationships of these sequences were inferred using three different methods, and depth-first searches of monophyletic groups with >95% in interior branch test, >95% in Bayesian posterior probability and <10% diversity were identified as a transmission cluster (TC). Time of the most recent common ancestor (tMRCA) and basic reproduction number (R0) of the TC were estimated with Bayesian inference. Transmission networks were estimated by linking two individuals in a TC whenever their sequences showed less than 1.5% genetic distance. Correlation between some network coefficients and demographic parameters in each TC were analyzed.

**Results:**

We identified 312 TCs that included 3708 individuals. The majority of TCs involved men (3625 cases) and MSMs (2656 cases). Orientation towards clustering was significantly associated with sex, risk behaviour and recent seroconversion of the individual. Number of individuals, tMRCA, median age and R0 of TCs did not vary within the major geographic region of a TC. Network analysis of the 44 largest TCs showed that density as well as degree centralization indices was correlated with tMRCA, suggesting a consistent developing pattern of transmission clusters in the Japanese MSM population from a dense and local transmission network to a sparse and widely distributed one.

**Conclusions:**

Our results suggest that HIV-1 spreads through MSM communities with a consistent pattern in Japan. Since patients in the cluster may have good awareness of HIV testing, the network information of the MSM community estimated from viral sequence may help to select a target for the active prevention programme, that is, PrEP, in Japan.

# Sub article: THAX0102 A study of potential HIV transmission hotspots among men who have sex with men and transgender women in Lima, Peru

**Published:** 2016-07-22

**Authors:** M Villaran, A Brezak, S Ahmed, A Ulrich, A Duerr, J Herbeck, J Mullins, E Seto

**Affiliations:** 1 IMPACTA, Lima, Peru; 2 Vaccine and Infectious Disease Division, Fred Hutchinson Cancer Research Center, Seattle, WA, United States; 3 Department of Biology, University of Washington, Seattle, WA, United States; 4 Department of Laboratory Medicine, University of Washington, Seattle, WA, United States; 5 Department of Epidemiology, University of Washington, Seattle, United States; 6 Vaccine and Infectious Diseases, Fred Hutchinson Cancer Research Center, Seattle, WA, United States; 7 University of Washington, Department of Global Health, Seattle, WA, United States; 8 School of Public Health, University of Washington, Seattle, WA, United States

## Abstract

**Introduction:**

Innovative prevention strategies for men who have sex with men (MSM) and transgender women (TGW) that effectively reduce incidence are critically needed; building these strategies will require identifying transmission networks and associated drivers of ongoing HIV transmission. The objective of this study was to identify key drivers and geographic sources of transmission among MSM/TGW in Lima, Peru through two aims: 1) To recognize geographic transmission hotspots through mapping locations of venues at which participants reported having sexual encounters and their residences; 2) To identify clusters of related incident infections through phylogenetic analysis and link these clusters to real-time data on sexual encounters, high-risk behaviours and attendance at social venues (bars, clubs, saunas, etc.).

**Methods:**

Between September 2013 and October 2015, MSM and TGW (n=3191) were screened for participation in a 24-month follow-up study (HIV prevalence=20.5%). HIV-uninfected individuals (n=2078) agreed to monthly HIV testing and completing surveys covering drug and alcohol use, sexual activity and attendance at social venues. Cohort HIV incidence was 8.6 per 1000 person-years (n=303). Locations of HIV-infected participants' residence and social venues where participants reported a sexual encounter were mapped and analyzed using the Getis-Ord-Gi* method to identify HIV hotspots. Putative transmission networks were identified by phylogenetic analysis of partial *pol* sequences obtained from incident HIV infections. Phylogenetic clusters were linked with individuals' data on venue attendance and sexual partners.

**Results:**

In the geographic analysis, 7 of the 20 social venues were identified as transmission hotspots (99% confidence); no neighbourhoods were identified as hotspots. Phylogenetic analysis indicated 13 clusters of highly-related infections (bootstrap values >90%). Within clusters with sufficient behavioural data covering the time of infection (n=7), all or most members reported sexual encounters in the 60 days prior to HIV diagnosis with partners they met at specific venues that had been identified as transmission hotspots in the geographic analysis.

**Conclusions:**

Both the phylogenetic and geographic cluster analyses identified related HIV incident cases associated with specific venues. These results support offering HIV prevention services, testing and linkage-to-care efforts at high-risk social venues rather than in neighbourhoods.

# Sub article: THAX0103 Using phylogenetics of HIV to inform prevention among young black men who have sex with men in Chicago

**Published:** 2016-07-22

**Authors:** E Morgan, A Nyaku, R D'Aquila, J Schneider

**Affiliations:** 1 Department of Public Health Sciences, University of Chicago, Chicago, IL, United States; 2 Northwestern University, Chicago, IL, United States

## Abstract

**Introduction:**

Phylogenetic analysis of HIV sequence data has potential to guide public health efforts and to clarify transmission network dynamics. Young black men who have sex with men (YBMSM) are now at greatest risk for HIV infection and a driver of continuing spread of HIV in Chicago. We identified characteristics associated with membership in phylogenetic clusters of HIV sequences and degree of connectivity among a cohort of YBMSM in Chicago.

**Methods:**

uConnect is the largest cohort study of YBMSM (aged 16–29) in Chicago (n=618). Survey data were collected using respondent-driven sampling. We analyzed HIV-1 genetic sequences from dried blood spots. We determined pairwise genetic distance, inferred transmission events between persons whose *pol* sequences were ≤1.5% genetically distant, and constructed clusters of HIV that connected persons (≥1 tie to another sequence). We determined demographic and risk attributes associated with both membership in a phylogenetic cluster and degree of connectivity within each cluster.

**Results:**

Our sample contained 214 (34.6%) HIV-diagnosed persons, from whom we analyzed 77 (36.0%) HIV *pol* sequences. Of 77 HIV sequences, 42 (54.5%) had a tie to genomes from ≥1 other person. In adjusted zero-inflated Poisson regression analyses, we determined that self-identity as either straight (Relative risk (RR)=9.12; 95% CI: 3.47–23.96) or bisexual (RR=5.94; 95% CI: 3.75–9.41), depressive symptoms (RR=1.91; 95% CI: 1.36–2.68) and recreational use of both marijuana (RR=12.14; 95% CI: 6.91–21.32) and cocaine/crack (RR=5.22; 95% CI: 2.81–9.72) were associated with greater connectivity within a phylogenetic cluster. We also found being currently insured (RR=0.61; 95% CI: 0.42–0.88), being in a relationship (RR=0.40; 95% CI: 0.27–0.59 and a greater number of confidants (RR=0.78; 95% CI: 0.67–0.92) to be associated with a lower degree of connectivity. We found no significant predictors of membership in a cluster.

**Conclusions:**

We determined that self-reported sexual identity, depressive symptoms and recreational drug use are associated with a high degree of connectivity within potential HIV transmission networks. Increasing access to mental health services and youth-focused drug use prevention programmes may reduce HIV transmission among YBMSM.

# Sub article: THAX0104 HIV phylogenetic analysis sheds light on transmission linkages in young women in high HIV burden districts in KwaZulu-Natal, South Africa

**Published:** 2016-07-22

**Authors:** T de Oliveira, P Khumalo, C Cawood, R Dellar, F Tanser, G Hunt, A Grobler, A Kharsany, L Madurai, Q Abdool Karim, S Abdool Karim

**Affiliations:** 1 College of Health Sciences, Nelson R Mandela School of Medicine, University of KwaZulu-Nata, Durban, South Africa; 2 Centre for the AIDS Programme of Research in South Africa (CAPRISA), University of KwaZulu-Natal, Durban, South Africa; 3 Epicentre AIDs Risk Management (Pty) Limited, Cape Town, South Africa; 4 University of Oxford, Oxford, United Kingdom; 5 Africa Centre for Health and Population Studies, University of KwaZulu-Natal, Mtubatuba, South Africa; 6 Centre for HIV and STIs, National Institute for Communicable Diseases, National Health Laboratory Service (NICD/NHLS, Johannesburg, South Africa; 7 Global Clinical and Virology Laboratory, Durban, South Africa

## Abstract

**Introduction:**

Strategies to reduce HIV transmission would benefit greatly from a better understanding of the sexual networks that drive HIV transmission in young women. Phylogenetic analysis has recently emerged as a powerful tool to examine the underlying dynamics of HIV-1 transmission.

**Methods:**

Samples were obtained from the HIV Incidence Provincial Surveillance System (HIPPS), a household-based study designed to monitor HIV prevalence and incidence trends in Vulindlela and Greater Edendale in rural KwaZulu-Natal. HIV genotyping of the pol region (1250 bp) was performed for 999 samples with viral load of >1000 c/ml. The best fitting evolutionary model (GTR+G+I) was calculated and a maximum likelihood tree constructed with 100 bootstrap replicates. Clusters of linked infections were identified (i.e. ≥2 sequences with bootstrap support ≥90 and diversity ≤4%). Statistical analysis was performed using Stata 10.0/SE.

**Results:**

We identified 27 phylogenetic clusters (average bootstrap=99.2% and diversity=1.7%). Of these, 10 were mixed gender clusters; nine dyads and a cluster with four individuals (one male and three females). In total, 12 females were linked to 10 males. All of the males were not on ART and had high viral load (Mean: 187,423 c/ml; Median: 106,165 c/ml). The mean age of males was 32.1 years and 27.7 years for the females. Six of the women were aged ≤24 years (Mean age: 20.3 years) and were linked to five men with a mean age of 28.4 years (p=0.014). The mean age difference in the remaining mixed clusters was 1.7 years. The age difference between linked women and men decreases as the woman age.

**Conclusions:**

Our results show that all the men linked to woman were not on ARVs and had a high viral load. Furthermore, the age disparity between young women (≤24 years) and linked men was on average less than 10 years. While we need to increase our sample coverage to test this results further, this study indicates that phylogenetics can provide key insight into the underlying dynamics of HIV-1 transmission in South Africa. In conclusion, breaking the HIV transmission cycle from older men to young women is crucial to control the epidemic.

# Sub article: THAX0105 Streamlined quasispecies and subtype analysis of HIV-1 sequences generated by high-throughput sequencing using the high-performance integrated virtual environment (HIVE)

**Published:** 2016-07-22

**Authors:** B Hora, N Gulzar, Y Chen, F Cai, C Su, K Karagiannis, K Smith, V Simonyan, SA Shah, M Ahmed, AM Sanchez, M Stone, MS Cohen, BF Haynes, MP Busch, R Mazumder, TN Denny, F Gao

**Affiliations:** 1 Duke Human Vaccine Institute, Duke University Medical Center, Durham, NC, United States; 2 Department of Medicine, Duke University Medical Center, Durham, NC, United States; 3 George Washington University, Washington, DC, United States; 4 Bridge Consultants Foundation, Karachi, Pakistan; 5 Blood Systems Research Institute, San Francisco, CA, United States; 6 Medicine, Epidemiology and Microbiology and Immunology, University of North Carolina at Chapel Hill, Chapel Hill, United States

## Abstract

**Background:**

High-throughput sequencing (HTS) has recently been used to characterize HIV-1 genome sequences. While sequences of HIV-1 from each sample can be easily analyzed with small gene fragments, it has been challenging to analyze half or whole genome sequences due to short HTS reads. To reliably identify diverse quasispecies populations and determine subtype of HTS sequences generated from long HIV-1 genome sequences, we have developed a new pipeline that defines distinct virus populations in each sample and determines subtypes or recombination breakpoints of the sequences using the High-performance Integrated Virtual Environment (HIVE).

**Methods:**

Viral RNA was extracted from 70 plasma samples from two chronic infection cohorts; External Quality Assurance Program Oversight Laboratory (EQAPOL) and the Center for HIV/AIDS Vaccine Immunology (CHAVI). The 3' half genome was amplified by RT-PCR and PCR amplicons were then sequenced by HTS on MiSeq. Raw reads were assembled and analyzed with the Geneious software and the HTS analysis tools in HIVE.

**Results:**

Final consensus sequences of 3'-half genomes were first generated for all viruses using Geneious. Genetic analysis of these sequences identified 17 A1s, 4 Bs, 30 Cs, 1 D, 6 CRF02_AG and 12 unique recombinant forms (URFs). Sequences from 41 viruses (58.6%) contained 1-178 ambiguous bases each, suggesting the presence of quasispecies viral populations in each sample and the single consensus generated by Geneious could not fully represent the viral population in these samples. We then analyzed the same raw reads using HIVE and found one species in 5 samples (7.1%), 2-10 species in 45 samples (64.3%), 11-20 species in 13 samples (18.6%), 21-30 species in 5 (7.1%), and 31-37 species in 2 samples (2.9%). Three samples contained two predominant populations which were not identified by Geneious. The subtyping and recombinant analysis results of the main species consensus sequences were the same as those determined by Geneious.

**Conclusions:**

HIVE provides a useful platform with specialized tools to analyze HTS data generated for the half HIV-1 genome by identifying multiple distinct quasispecies populations and determining subtypes or recombination patterns of each species consensus sequences in the same samples.

# Sub article: FRAB0101LB Enhanced infection prophylaxis reduces mortality in severely immunosuppressed HIV-infected adults and older children initiating antiretroviral therapy in Kenya, Malawi, Uganda and Zimbabwe: the REALITY trial

**Published:** 2016-07-22

**Authors:** J Hakim, V Musiime, AJ Szubert, A Siika, J Mallewa, C Agutu, SL Pett, M Bwakura-Dangarembizi, A Lugemwa, S Kaunda, M Karoney, K Maitland, A Griffiths, C Kityo, P Mugyenyi, AJ Prendergast, AS Walker, DM Gibb, REALITY Trial Team

**Affiliations:** 1 University of Zimbabwe Clinical Research Centre, Harare, Zimbabwe; 2 Joint Clinical Research Centre, Kampala, Uganda; 3 MRC Clinical Trials Unit, UCL, London, United Kingdom; 4 Moi University School of Medicine, Eldoret, Kenya; 5 Department/College of Medicine and Malawi-Liverpool-Wellcome Trust Clinical Research Programme, Blantyre, Malawi; 6 KEMRI Wellcome Trust Research Programme, Kilifi, Kenya; 7 Joint Clinical Research Centre, Mbarara, Uganda; 8 Queen Mary University of London, London, United Kingdom

## Abstract

**Introduction:**

Mortality from infections is high in the first 6 months of antiretroviral therapy (ART) among HIV-infected adults and children with advanced disease in sub-Saharan Africa. Whether an enhanced package of infection prophylaxis at ART initiation would reduce mortality is unknown.

**Methods:**

The REALITY 2×2×2 factorial open-label trial (ISRCTN43622374) randomized ART-naïve HIV-infected adults and children >5 years with CD4 <100 cells/mm^3^. This randomization compared initiating ART with enhanced prophylaxis (continuous cotrimoxazole plus 12 weeks isoniazid/pyridoxine (anti-tuberculosis) and fluconazole (anti-cryptococcal/candida), 5 days azithromycin (anti-bacterial/protozoal) and single-dose albendazole (anti-helminth)), versus standard-of-care cotrimoxazole. Isoniazid/pyridoxine/cotrimoxazole was formulated as a scored fixed-dose combination. Two other randomizations investigated 12-week adjunctive raltegravir or supplementary food. The primary endpoint was 24-week mortality.

**Results:**

1805 eligible adults (n = 1733; 96.0%) and children/adolescents (n = 72; 4.0%) (median 36 years; 53.2% male) were randomized to enhanced (n = 906) or standard prophylaxis (n = 899) and followed for 48 weeks (3.8% loss-to-follow-up). Median baseline CD4 was 36 cells/mm^3^ (IQR: 16–62) but 47.3% were WHO Stage 1/2. 80 (8.9%) enhanced versus 108(12.2%) standard prophylaxis died before 24 weeks (adjusted hazard ratio (aHR) = 0.73 (95% CI: 0.54–0.97) p = 0.03; Figure 1) and 98(11.0%) versus 127(14.4%) respectively died before 48 weeks (aHR = 0.75 (0.58–0.98) p = 0.04), with no evidence of interaction with the two other randomizations (p > 0.8). Enhanced prophylaxis significantly reduced incidence of tuberculosis (p = 0.02), cryptococcal disease (p = 0.01), oral/oesophageal candidiasis (p = 0.02), deaths of unknown cause (p = 0.02) and (marginally) hospitalisations (p = 0.06) but not presumed severe bacterial infections (p = 0.38). Serious and grade 4 adverse events were marginally less common with enhanced prophylaxis (p = 0.06). CD4 increases and VL suppression were similar between groups (p > 0.2).

**Conclusions:**

Enhanced infection prophylaxis at ART initiation reduces early mortality by 25% among HIV-infected adults and children with advanced disease. The pill burden did not adversely affect VL suppression. Policy makers should consider adopting and implementing this low-cost broad infection prevention package which could save 3.3 lives for every 100 individuals treated.

**Figure 1:** All-cause mortality.

# Sub article: FRAB0102LB 12-week raltegravir-intensified quadruple therapy versus triple first-line ART reduces viral load more rapidly but does not reduce mortality in severely immunosuppressed African HIV-infected adults and older children: the REALITY trial

**Published:** 2016-07-22

**Authors:** C Kityo, A Siika, AJ Szubert, J Mallewa, M Bwakura-Dangarembizi, S Kabahenda, S Mwaringa, SL Pett, A Griffiths, A Lugemwa, S Wachira, G Musoro, C Rajapakse, T Etyang, J Abach, P Wavamunno, L Nyondo-Mipando, A Reid, K Nathoo, J Hakim, DM Gibb, AS Walker, REALITY trial team

**Affiliations:** 1 Joint Clinical Research Centre, Kampala, Uganda; 2 Moi University School of Medicine, Eldoret, Kenya; 3 MRC Clinical Trials Unit at UCL, London, United Kingdom; 4 Department/College of Medicine and Malawi-Liverpool-Wellcome Trust Clinical Research Programme, Blantyre, Malawi; 5 University of Zimbabwe Clinical Research Centre, Harare, Zimbabwe; 6 Joint Clinical Research Centre, Fort Portal, Uganda; 7 KEMRI Wellcome Trust Research Programme, Kilifi, Kenya; 8 Joint Clinical Research Centre, Mbarara, Uganda; 9 Joint Clinical Research Centre, Gulu, Uganda

## Abstract

**Introduction:**

Early mortality after initiating antiretroviral therapy (ART) is high among HIV-infected adults and children with advanced disease in sub-Saharan Africa. Intensifying ART with an integrase inhibitor should reduce viral load (VL) faster, but whether this reduces early mortality is unknown.

**Methods:**

The REALITY 2×2×2 factorial open-label trial (ISRCTN43622374) randomized ART-naïve HIV-infected adults and children >5 years with CD4 <100 cells/mm^3^ from Kenya, Malawi, Uganda and Zimbabwe. This randomization compared initiating ART with 2NRTI + NNRTI with or without 12-week raltegravir intensification. Two other randomizations investigated 12-week enhanced infection prophylaxis or supplementary food. The primary endpoint was 24-week mortality.

**Results:**

1805 eligible adults (n = 1733; 96.0%) and children/adolescents (n = 72; 4.0%) (median 36 years; 53.2% male) were randomized to raltegravir-intensified (n = 903) or standard (n = 902) ART and followed for 48 weeks (3.8% loss-to-follow-up). Median baseline CD4 was 36 cells/mm^3^ (IQR: 16–62) and VL 230,000 c/mL (72.5% ≥100,000 c/mL). At 4, 12, 24 and 48 weeks, VL was <50 c/mL in 42.8%, 74.1%, 77.2% and 82.9% in 12-week raltegravir-intensified versus 14.5%, 54.6%, 76.0% and 79.5% standard ART (p < 0.001, <0.001, 0.59, 0.12, respectively) (Figure 1). CD4 increases through 24 weeks were similar (p = 0.82), although a small difference became apparent at 48 weeks (+163 cells/mm^3^ intensified versus +148 cells/mm^3^ standard, p = 0.04). 97 (10.9%) intensified versus 91(10.2%) standard ART died before 24 weeks (adjusted hazard ratio (aHR) = 1.09 (95% CI: 0.82–1.46) p = 0.54); 110 (12.4%) versus 115 (13.0%) respectively died before 48 weeks (aHR = 0.98 (0.75–1.27) p = 0.86), with no evidence of interaction with the two other randomizations (p > 0.7). There was no difference in time to first WHO 3/4 event or death (p = 0.31). Serious adverse events (AEs), grade 3/4 AEs and drug-related AEs (adjudicated blind to randomization) were similar in both groups (p > 0.3).

**Conclusions:**

12-week raltegravir-intensified ART was well tolerated, resulted in faster VL reduction through 24 weeks and increased CD4 at 48 weeks, but did not reduce mortality or WHO 3/4 events.

**Figure 1:** VL and CD4.

# Sub article: FRAB0103LB Raltegravir (RAL) 1200 mg once daily (QD) is non-inferior to RAL 400 mg twice daily (BID), in combination with tenofovir/emtricitabine, in treatment-naïve HIV-1-infected subjects: Week 48 results

**Published:** 2016-07-22

**Authors:** P Cahn, R Kaplan, P Sax, K Squires, J-M Molina, A Avihingsanon, W Ratanasuwan, E Rojas, M Rassool, X Xu, A Rodgers, S Rawlins, B-Y Nguyen, R Leavitt, H Teppler, for the Oncemrk Study Group

**Affiliations:** 1 Fundacion Huesped, Buenos Aires, Argentina; 2 Desmond Tutu HIV Foundation, Cape Town, South Africa; 3 Brigham & Women's Hospital, Harvard Medical School, Boston, MA, United States; 4 Thomas Jefferson University, Philadelphia, PA, United States; 5 Hopital Saint-Louis, Paris, France; 6 HIV-NAT Research Collaboration, Bangkok, Thailand; 7 Faculty of Medicine Siriraj Hospital, Mahidol University, Bangkok, Thailand; 8 Cericap Multiclinicas, Guatemala City, Guatemala; 9 Helen Joseph Hospital, University of Witwatersrand, Johannesburg, South Africa; 10 Merck & Co., Inc., Kenilworth, NJ, United States

## Abstract

**Introduction:**

The investigational reformulated raltegravir (RAL) 600 mg tablet for once daily (QD) use at 1200 mg dose could provide a more convenient option for treatment of HIV-1 infection.

**Methods:**

ONCEMRK is a phase 3, multicentre, double-blind, randomized, controlled trial to evaluate if reformulated RAL 1200 mg QD is non-inferior to RAL 400 mg twice daily (BID). Treatment-naïve HIV-1-infected subjects were assigned (2:1) to reformulated RAL 2×600 mg QD or RAL 400 mg BID, both with tenofovir/emtricitabine, for up to 96 weeks. Randomization was stratified by screening HIV-1 RNA (vRNA) and chronic hepatitis B/C status. The primary efficacy endpoint was the proportion of subjects with vRNA <40 copies/mL at Week 48 (non-completer = failure).

**Results:**

Of 802 subjects randomized, 797 received study therapy and were included in the analyses; 732 (92%) completed 48 weeks of treatment. The study population was 85% male, 59% white, mean age 35.9 years, mean CD4 count 415/mm^3^, mean plasma vRNA 4.6 log_10_ copies/mL, 28.4% had baseline vRNA >100,000 copies/mL, 2.9% had hepatitis B and/or C co-infection. Subjects in both groups achieved a rapid decline in vRNA (>50% reaching vRNA <40 copies/mL by Week 4). At Week 48, RAL 1200 mg QD was non-inferior to RAL 400 mg BID (vRNA <40 copies/mL in 88.9% and 88.3%, respectively, Δ(QD-BID) = 0.5%, 95% CI (−4.2, 5.2)). Study results did not differ significantly by baseline vRNA or hepatitis co-infection status. RAL 1200 mg QD also had comparable immunologic efficacy, as measured by change from baseline in CD4 cell counts. Both treatment regimens were well tolerated with comparable incidence of clinical adverse events (Table 1) and laboratory values exceeding predefined limits of change (based on DAIDS toxicity criteria).

**Conclusions:**

In HIV-1-infected treatment-naïve subjects receiving tenofovir/emtricitabine, reformulated RAL 1200 mg QD demonstrated potent and non-inferior efficacy compared to RAL 400 mg BID at Week 48. RAL 1200 mg QD was safe and well tolerated with a safety profile similar to RAL 400 mg BID.

**Abstract FRAB0103LB–Table 1:** Key efficacy and safety results, ONCEMRK Week 48

Endpoint	RAL 1200 mg QD (N = 531)	RAL 400 mg BID (N = 266)	Δ QD-BID (95% CI)
HIV RNA <40 copies/mL			
All subjects (NC = F)	88.9%	88.3%	0.5 (−4.2, 5.2)
Baseline vRNA >100,000 copies/mL (OF)	86.7%	83.8%	2.9 (−6.5, 14.1)
Baseline CD4 ≤200 cells/mm^3^ (OF)†	85.1%	87.9%	−2.8 (−16.0, 14.0)
Hepatitis B and/or C co-infection (OF)	100%	85.7%	14.3 (−11.7, 52.2)
HIV RNA <200 copies/mL (NC = F)	91.1%	91.4%	−0.2 (−4.4, 4.0)
Mean CD4 Change (95% CI), cells/mm^3^ (OF)	232 (215, 249)	234 (213, 255)	−2.1 (−31, 27)
One or more clinical adverse events	82.7%	86.8%	−4.2 (−9.2, 1.3)
Drug-related‡ adverse events	24.5%	25.6%	−1.1 (−7.6, 5.1)
Serious adverse events	5.8%	9.4%	−3.6 (−8.0, 0.2)
Serious drug-related adverse events	0.2%	0.8%	−0.6 (−2.5, 0.4)
Discontinued§ due to adverse event	0.8%	2.3%	−1.5 (−4.1, 0.1)

All subjects also received tenofovir/emtricitabine.

† Combination of two pre-specified groups (550 and >50 to £200 cells/mm^3^).

‡ Determined by the investigator to be related to study drug.

§ Study medication withdrawn.

NC = F: Non-Completer = Failure, as defined by FDA snapshot approach (all missing data treated as failures); OF: Observed Failure approach.

# Sub article: FRAB0104LB Dolutegravir-lamivudine as initial therapy in HIV-infected, ARV naive patients: 48 week results of the PADDLE trial

**Published:** 2016-07-22

**Authors:** P Cahn, MJ Rolón, MI Figueroa, A Gun, P Patterson, O Sued

**Affiliations:** 1 Fundacion Huesped, Clinical Research, Ciudad de Buenos Aires, Argentina; 2 Fundacion Huesped, Clinical Research lab, Ciudad de Buenos Aires, Argentina

## Abstract

**Introduction:**

Based on the results of the GARDEL trial, we designed a proof of concept study to evaluate the antiviral efficacy, safety and tolerability of a dual therapy regimen with dolutegravir (DTG) 50 mg QD plus lamivudine (3TC) 300 mg QD as initial HAART among ARV-naïve patients.

**Methods:**

Pilot study including 20 HIV-infected ARV-naïve adults. Eligible participants had no IAS-USA defined NRTI resistance, HIV-1 RNA <100,000 copies/mL at screening and negative HBsAg. Viral load (pVL) was measured at baseline, on days 2, 4, 7, 10, 14, 21, 28 and on weeks 6, 8, 12, and thereafter every 12 months up to 96 weeks. Primary endpoint was the proportion of patients with HIV-1 RNA <50 copies/mL in an ITT-exposed analysis at 48 weeks (FDA-snapshot algorithm). Week 24 interim analysis was already presented at EACS 2015. Week 48 results are reported here.

**Results:**

Median HIV-1 RNA at baseline was 24,128 copies/mL (IQR: 11,686–36,794). Albeit as per protocol, all patients had pVL <100,000 copies/mL at screening, four patients had ≥100,000 copies/mL at baseline. Median CD4+ T-cell count was 507 per cubic millimetre (IQR: 296–517). A rapid antiviral response was observed. Median VL decay baseline to Week 12 was 2.74 logs. All participants had pVL <50 copies from Week 8 onwards up to Week 24. At Week 48, 90% (18/20) reached the primary end point of a pVL <50 copies/mL. No major tolerability/toxicity issues were observed. Eighteen patients completed 48 weeks of the study, one patient (with undetectable viral load at last visit) committed suicide, in the context of a severe stress and emotional trauma deemed unrelated to study medication. One patient presented a low level protocol-defined confirmed virological failure at Week 36, being the only observed failure. This patient resuppressed to pVL <50 copies/mL prior to treatment intensification. Resistance tests revealed: RT: no emergent substitutions; integrase: not amplified.

**Conclusions:**

Dual therapy with DTG/lamivudine produced rapid virologic suppression with a favourable safety/tolerability profile in HIV-infected, treatment-naive individuals. Observed failure rate was 5%. This is the first report of a successful InSTI/lamivudine-based dual therapy in ARV-naïve patients after 48 weeks of treatment.

# Sub article: FRAC0101 CHALO! A social media based peer-delivered intervention increases HIV testing in men who have sex with men in Mumbai, India: a randomized trial

**Published:** 2016-07-22

**Authors:** VV Patel, S Rawat, C Lelutiu-Weinberger, A Dange, C Kamath, R Poojary, M Bisht, SA Golub

**Affiliations:** 1 Medicine, Montefiore Medical Center, Bronx, NY, United States; 2 Humsafar Trust, Mumbai, India; 3 Department of Psychology, Hunter College, City University of New York, New York City, NY, United States

## Abstract

**Introduction:**

Social media can provide effective delivery of HIV prevention messages to men who have sex with men (MSM). This study is the first to engage MSM in India in an online intervention to investigate the efficacy of two types of HIV prevention message framing: Approach (i.e. a good outcome to be achieved) versus Avoidance (a bad outcome to be avoided).

**Methods:**

Using participatory processes, we developed messages targeting HIV testing to fit either approach- or avoidance frames. Using social media, we recruited MSM ages 18 or older living in Mumbai. After completing a screener and baseline survey online, participants were randomized to receive either 16 approach- or 16 avoidance-framed messages via their preferred modality: private Facebook group, individual WhatsApp messaging or email. Peers delivered messages 2/week for 12 weeks (February-May 2015), and participants completed a post-intervention survey. Primary outcomes were

1. recent HIV test (past 6 months) and
2. intention to test in the next month.

**Results:**

Over 82% of participants (n=200) were retained, and 53% (n=130) completed follow-up; there were no baseline differences by messaging arm or follow-up completion. Participants were primarily between 18 and 29 years old (64%), identified as gay (67%) or bisexual (26%), had an average of four male partners in the past 3 months, and 39% reported inconsistent condom use. There was a significant increase in recent HIV testing from baseline to follow-up (32 to 44%; p<0.05). Among those who did not report recent HIV testing (n=50), intentions to test increased significantly as well (32 to 56%; p=0.01). Participants reported liking approach messages more, but believing that avoidance messages were more effective. A significantly higher proportion of participants in the avoidance condition reported recent testing or intention to test (82%), compared to those in the approach condition (65%), p=0.03.

**Conclusions:**

This first study of a social media-based HIV intervention in India demonstrates preliminary evidence for increasing HIV testing in an urban online sample of MSM, with potential for wide national reach. Further work is needed to better understand how message-framing impacts HIV testing and risk behaviours in Indian MSM for future tailored online interventions.

# Sub article: FRAC0102 Access to HIV self-testing doubles the frequency of HIV testing among gay and bisexual men at higher risk of infection: a randomized controlled trial

**Published:** 2016-07-22

**Authors:** MS Jamil, G Prestage, CK Fairley, AE Grulich, KS Smith, M Chen, M Holt, AM McNulty, BR Bavinton, DP Conway, H Wand, P Keen, J Bradley, J Kolstee, C Batrouney, D Russell, M Law, JM Kaldor, RJ Guy

**Affiliations:** 1 The Kirby Institute, University of New South Wales (UNSW), Sydney, Australia; 2 Australian Research Centre in Sex, Health and Society, La Trobe University, Melbourne, Australia; 3 Central Clinical School, Monash University, Melbourne, Australia; 4 Melbourne Sexual Health Centre, Alfred Health, Melbourne, Australia; 5 Centre for Social Research in Health, UNSW Australia, Sydney, Australia; 6 Sydney Sexual Health Centre, Sydney Hospital, Sydney, Australia; 7 ACON, Sydney, Australia; 8 Victorian AIDS Council, Gay Men's Health Centre, Melbourne, Australia; 9 Cairns Sexual Health Service, Cairns North, Australia; 10 James Cook University, Townsville, Australia

## Abstract

**Introduction:**

Frequent testing of gay and bisexual men (GBM) at higher-risk of HIV is central to current prevention strategies. We conducted the first randomized trial to determine if access to HIV self-testing would increase testing frequency in two groups of higher-risk GBM; those who had tested within the past 2 years and those who had not.

**Methods:**

In this wait-list control randomized trial, HIV-negative higher-risk GBM reporting condomless anal intercourse or >5 male sexual partners in the past 3 months were recruited at three clinical and two community-based sites in Australia. Enrolled participants were randomly assigned (1:1) via computer-generated randomization codes to have free access to HIV self-testing (intervention) or not (standard-care). Participants completed 3-monthly online questionnaires. The primary outcome was the number of HIV tests over 12 months, analyzed by intention-to-treat. The study was designed to evaluate the primary outcome overall and in two strata: frequent (last HIV test ≤2 years ago) and infrequent (>2 years ago or never tested) testers.

**Results:**

Between Dec-2013 and Nov-2014, 180 men were randomized to self-testing and 179 to standard-care. The intention-to-treat analysis included men who completed any follow-up questionnaire: 179 (98%) in self-testing; and 164 (92%) in standard-care. The mean number of HIV tests over 12 months in the self-testing and standard-care arms was 3.9 and 1.6 per-person overall (rate ratio (RR): 2.39, 95% CI: 2.08–2.76, p<0.001), 4.0 and 1.8 among frequent testers (RR: 2.23, 1.93–2.59, p<0.001), and 3.2 and 0.6 among infrequent testers (RR: 5.54, 3.15–9.74, p<0.001), respectively. There was no statistical difference between the two arms in the mean number of facility-based HIV tests (1.4 vs. 1.6, RR: 0.89, 0.75–1.06) and any STI test (1.6 vs. 1.7, RR: 0.93, 0.79–1.10).

**Conclusions:**

HIV self-testing among higher-risk GBM increased HIV testing frequency by more than two-fold overall, and more than five-fold among infrequent testers, without reducing facility-based HIV/STI testing frequency. Self-testing should be provided more widely to achieve public health goals of increasing HIV testing frequency.

**Abstract FRAC0102–Table 1:** Mean number of HIV and sexually transmitted infection (STI) tests over 12 months in intention-to-treat population. Mean(SD) unless otherwise specified

	Overall			Frequent testers			Infrequent testers		
			
Type of test	Self-testing (n=177)	Standard-care (n=164)	Rate-ratio (95% CI)	Self-testing (n=147)	Standard-care (n=140)	Rate-ratio (95% CI)	Self-testing (n=30)	Standard-care (n=24)	Rate-ratio (95% CI)
**Self/facility-based HIV**	3.9 (0.2)	1.6 (0.1)	**2.39 (2.08–2.76)**	4.0 (0.2)	1.8 (0.1)	**2.23 (1.93–2.59)**	3.2 (0.5)	0.6 (0.2)	**5.54 (3.15–9.74)**
**Facility-based HIV**	1.4 (0.1)	1.6 (0.1)	0.89 (0.75–1.06)	1.6 (0.1)	1.8 (0.2)	0.88 (0.73–1.05)	0.8 (0.3)	0.6 (0.2)	1.41 (0.73–2.73)
**Any STI**	1.6 (0.1)	1.7 (0.2)	0.93 (0.79–1.10)	1.8 (0.1)	1.9 (0.2)	0.92 (0.77–1.09)	0.9 (0.3)	0.7 (0.2)	1.35 (0.73–2.49)
**Chlamydia/ Gonorrhoea**	1.5 (0.1)	1.6 (0.1)	0.94 (0.80–1.12)	1.6 (0.1)	1.8 (0.2)	0.93 (0.78–1.11)	0.9 (0.3)	0.6 (0.2)	1.33 (0.71–2.50)
**Syphilis**	1.4 (0.1)	1.5 (0.1)	0.90 (0.76–1.08)	1.5 (0.1)	1.7 (0.1)	0.89 (0.74–1.07)	0.8 (0.3)	0.6 (0.2)	1.41 (0.72–2.76)

The bold values in the table are statistically significant (p<0.001).

# Sub article: FRAC0103 Community-based voluntary counselling and testing successfully identifies HIV-positive ART eligible individuals in rural South Africa

**Published:** 2016-07-22

**Authors:** S Shenoi, A Moll, J Madi, V Guddera, T Madondo, D Turner, R Brooks, T Kyriakides, L Andrews, G Friedland

**Affiliations:** 1 Infectious Diseases, School of Medicine, Yale University, New Haven, CT, United States; 2 Church of Scotland Hospital, Tugela Ferry, South Africa; 3 Philanjalo NGO, Tugela Ferry, South Africa; 4 Umvoti AIDS Centre, Greytown, South Africa; 5 School of Medicine, Yale University, New Haven, CT, United States; 6 Yale Center for Analytical Sciences, New Haven, CT, United States

## Abstract

**Introduction:**

Community-based voluntary counselling and testing (CBVCT) is a validated strategy to increase HIV awareness and testing. South Africa has the largest global epidemic of HIV, and a substantial proportion is unaware of being infected. New testing strategies are needed. We describe a successful CBVCT strategy in rural South Africa.

**Methods:**

A team of nurses and community health workers provided health education, rapid HIV testing and concurrent TB screening in congregate community settings in rural KwaZulu Natal from 2010 to 2015. Those identified with HIV were offered confirmatory testing, CD4 staging, individual counselling based on CD4 count and referral to care and antiretroviral treatment (ART) according to national guidelines.

**Results:**

CBVCT was performed at 849 community sites including municipality events, pension pay points and taxi ranks. Among 13,278 screened, the median age was 41(IQR 23–57), 8099 (70.8%) were women and 11,435 (86.1%) accepted HIV testing. Twelve hundred and forty-four (9.4%) individuals were identified as HIV-infected. Among 720 (57.9%) accepting phlebotomy, the median CD4 count was 424 (IQR 270–583); 447 (62%) qualified for antiretroviral therapy (ART). A substantial proportion of participants (4510, 39.4%) reported first-time HIV testing. Preliminary analysis identifies correlates of HIV-positive test result including young age (p<0.001), contact with a TB patient (p<0.001), chronic diarrhoea (p<0.001), recurrent pneumonia (p<0.001) and type of community site of HIV testing (p<0.001). Taxi ranks yielded the greatest proportion of community members (176/1123, 15.7%) with HIV-positive test result. Among all HIV-positive men, the greatest proportion (74, 25%) was identified at municipality events.

**Conclusions:**

Community members accept HIV testing outside of health care facilities and by non-clinical personnel. Utilizing a variety of community testing sites reaches different demographic groups, including high-priority young men and women. CBVCT can detect a large number of HIV infected individuals, the majority of whom are eligible for ART. Scale-up of CBVCT may provide needed increase in levels of HIV awareness, testing and diagnoses in rural areas.

# Sub article: FRAC0104 Promoting male partner and couples HIV testing through secondary distribution HIV self-tests: a randomized trial

**Published:** 2016-07-22

**Authors:** H Thirumurthy, S Masters, B Obonyo, S Napierala Mavedzenge, S Maman, E Omanga, K Agot

**Affiliations:** 1 Department of Health Policy and Management, University of North Carolina at Chapel Hill, Chapel Hill, NC, United States; 2 Impact Research and Development Organization, Kisumu, Kenya; 3 RTI International, San Francisco, CA, United States; 4 Department of Health Behavior, University of North Carolina at Chapel Hill, Chapel Hill, NC, United States

## Abstract

**Introduction:**

There is a vital need to achieve higher uptake of HIV testing among men and couples in sub-Saharan Africa. Providing multiple HIV self-tests to individuals for distribution to their sexual partners, that is, “secondary distribution,” is a promising strategy with potential to increase awareness of HIV status. This strategy may be particularly useful for promoting male partner testing and couples testing in antenatal and postpartum settings.

**Methods:**

We conducted a randomized trial at three clinics in Kisumu, Kenya (NCT02386215). Women seeking antenatal and postpartum care, aged 18–39 years, and reporting their primary partner was not known to be HIV-infected, were randomized to an HIV self-testing (HIVST) group or a comparison group. In the HIVST group, women were provided two OraQuick self-tests, a demonstration and instructions on how to use the self-tests, and encouragement to distribute a self-test to their partner. In the comparison group, women were provided invitation cards for their partner to seek counsellor-administered HIV testing at the clinics. Follow-up interviews were conducted with women after they reported their partner had tested, and all women were interviewed at 3 months. The primary outcome was HIV testing by the male partner within 3 months, and the secondary outcome was couples testing within 3 months. Chi-squared tests were used to compare outcomes in the intervention and comparison group.

**Results:**

Between June 11, 2015 and October 16, 2015, 600 women were randomly assigned to the HIVST group (n=297) or the control group (n=303). Follow-up was completed for 570 (95.0%) women. Male partner testing uptake was 90.5% (257/284) in the HIVST group and 51.7% (148/286) in the comparison group (difference=38.7%, 95% CI: 31.9–45.5%, p<0.001). Couples testing was also significantly higher in the HIVST group than the comparison group (75.0% vs. 33.2%, difference=41.7%, 95% CI: 34.3–49.2%, p<0.001). One adverse event was reported in the HIVST group, and none were reported in the comparison group.

**Conclusions:**

Secondary distribution of HIV self-tests by pregnant and postpartum women was highly effective in promoting male partner and couples testing. As countries scale-up HIVST, further implementation of secondary distribution interventions can help increase HIV testing uptake among hard-to-reach populations.

# Sub article: FRAC0105LB The impact of universal test and treat on HIV incidence in a rural South African population: ANRS 12249 TasP trial, 2012–2016

**Published:** 2016-07-22

**Authors:** C Iwuji, J Orne-Gliemann, E Balestre, J Larmarange, R Thiebaut, F Tanser, N Okesola, T Makowa, J Dreyer, K Herbst, N Mc Grath, T Barnighausen, S Boyer, T De Oliveira, C Rekacewicz, B Bazin, M-L Newell, D Pillay, F Dabis, for the Anrs 12249 Tasp Study Group

**Affiliations:** 1 Africa Centre for Population Studies, Somkhele, South Africa; 2 Research Department of Infection and Population Health, University College London, London, United Kingdom; 3 INSERM U1219 Bordeaux Population Health, Universite de Bordeaux, Bordeaux, France; 4 IRD, Centre Population & Développement (UMR 196 Paris Descartes IRD), Paris, France; 5 School of Nursing and Public Health, University of KwaZulu-Natal, Durban, South Africa; 6 Faculty of Medicine and Faculty of Human, Social and Mathematical Sciences, University of Southampton, Southampton, United Kingdom; 7 Department of Global Health & Population, Harvard School of Public Health, Harvard University, Boston, MA, United States; 8 INSERM, UMR912 (SESSTIM), Marseille, France; 9 Université Aix Marseille, UMR S_912, IRD, Marseille, France; 10 Agence Nationale de Recherches sur le Sida et les hépatites virales, Paris, France; 11 Faculty of Medicine, University of Southampton, Southampton, United Kingdom; 12 Global Health Research Institute, University of Southampton, Southampton, United Kingdom; 13 Faculty of Medical Sciences, University College London, London, United Kingdom

## Abstract

**Introduction:**

The population impact of universal test and treat (UTT) on HIV transmission has not yet been evaluated.

**Methods:**

A cluster-randomized trial was implemented in 2×11 rural communities in KwaZulu-Natal, South Africa. All residents ≥16 years were offered rapid HIV testing and provided dried blood spots (DBS) during 6-monthly home-based survey rounds. HIV-positive participants were referred to cluster-based trial clinics to receive ART regardless of CD4 count (intervention arm) or according to national guidelines (control arm). Standard of care ART was also available in the Department of Health clinics. HIV incidence was estimated on repeat DBS using cluster-adjusted Poisson regression.

**Results:**

Between 03/2012 and 04/2016, 13,239 and 14,916 individuals (63% women, median age 30 years) were registered in the intervention and control arms. Contact frequency per round among registered individuals ranged from 64 to 83%, HIV ascertainment from 74 to 85%. Baseline HIV prevalence was 29.4% (95% CI: 28.8–30.0), with 7578 individuals identified as HIV-positive. 1,513(36%) of 4172 HIV-positive individuals not previously in care linked to trial clinics within 6 months of referral. ART initiation in trial clinics at 3 months was 90.9% (576/634) and 52.3% (332/635) in the intervention and control arms; viral suppression (<400 copies/mL) 12 months after ART initiation was 94.9% (300/316) and 94.2% (194/206), respectively. Overall ART coverage at entry was 31% and 36% in the intervention and control arms, reaching 41% in both arms by closing date. Repeat DBS tests were available for 13,693 individuals HIV-negative at baseline, yielding 461 seroconversions in 20,833 person-years (PY). HIV incidence was 2.16 per 100 PY (1.88–2.45) in the intervention arm and 2.26 (1.98–2.54) in the control arm (adjusted relative risk: 0.95 (0.82–1.10)). Severe adverse events rates were 3.4% (45/1323) and 3.5% (57/1604) in the intervention and control arms. Follow-up will be completed by 06/2016.

**Conclusions:**

Our trial shows high acceptance of home-based HIV testing and high levels of viral suppression among individuals on ART. However, overall linkage to care remains poor. No reduction in HIV incidence was demonstrated. Several factors are being investigated, including determinants of poor linkage, change in national ART guidelines, migration and geography of sexual networks.

Funded by ANRS, GiZ and 3ie; Clinical Trials registration NCT00332878.

# Sub article: FRAD0101 “One shouldn't convict people for hypothetical risks”: frustratingly slow incorporation of the prevention impact of antiretroviral therapy into criminal law and policy

**Published:** 2016-07-22

**Authors:** EJ Bernard, P Eba, C Kazatchkine

**Affiliations:** 1 HIV Justice Network, Brighton, United Kingdom; 2 Human Rights and Law Division, UNAIDS, Geneva, Switzerland; 3 Canadian HIV/AIDS Legal Network, Toronto, ON, Canada

## Abstract

**Introduction:**

The prevention impact of antiretroviral therapy (ART) is now established as a key component of the HIV response. But despite this remarkable scientific advancement, many people living with HIV around the world remain vulnerable to the risk of unjust prosecutions for alleged HIV non-disclosure, potential or perceived exposure or non-intentional transmission because up-to-date science on HIV risk has not been recognized in criminal law and policy.

**Description:**

We undertook a desk review of criminal proceedings, policy documents and newspaper reports collated on the HIV Justice Network website to better understand the implications of increased knowledge and awareness of the prevention benefit of ART as they relate to HIV non-disclosure, exposure and/or transmission laws, policies and prosecutions.

**Lessons learned:**

Despite recognition by WHO and other normative agencies of the impact of ART on the risks of HIV transmission, criminal justice actors and lawmakers have been frustratingly slow to incorporate up-to-date HIV science into criminal law and policy. The key component of recognizing the prevention impact of ART on HIV risk has been collaboration between scientists, clinicians, lawyers and advocates. This is as true in the Netherlands, the first country to consider low viral load as a factor in assessing HIV risk in 2005, as it has been in, for example, the United States Court of Appeals for the Armed Forces (2015) and the Czech Republic (2015). Without this co-ordinated effort higher courts and lawmakers generally ignore up-to-date science even if lower courts occasionally make more rational, informed decisions, for example, in Austria, Canada and Germany.

**Conclusions/Next steps:**

It is vitally important that criminal justice system actors and law- and policymakers are educated so that HIV-related criminal laws and policies are applied rationally and fairly. Scientists and clinicians must, therefore, work more closely with HIV and human rights activists, advocates and lawyers in jurisdictions where the prevention impact of ART is not currently legally recognized, in order to prevent miscarriages of justice and to ensure that the prevention benefit of ART is correctly understood by criminal justice actors, policymakers, and the media as well people living with HIV and people likely to make a criminal complaint.

# Sub article: FRAD0102 Inconsistencies in legal frameworks on adolescent HIV and sexual and reproductive health services in five southern African countries

**Published:** 2016-07-22

**Authors:** A Müller, K Daskilewicz, S Spencer, T Meer, L Artz

**Affiliations:** Gender Health and Justice Research Unit, University of Cape Town, Cape Town, South Africa

## Abstract

**Introduction:**

While specific disaggregated HIV prevalence data for adolescents (aged 12–18) does not exist, young people (aged 15–24) account for 39% of all HIV infections globally, most of which occur in Sub-Saharan Africa (SSA). Conflicting laws surrounding the age of consent, sexual activity between adolescents and mandatory reporting – which in some instances criminalize certain sexual activities between adolescents – have a deleterious impact on the extent to which adolescents can access and, by extension, receive HIV counselling and testing and other sexual and reproductive health (HCT/SRH) services. The legal frameworks around HCT/SRH service provision in Malawi, Mozambique, Namibia, Zambia and Zimbabwe, five SSA countries with high adolescent HIV prevalence rates, were analysed for their impact on adolescents’ access to HCT/SRH services.

**Methods:**

Following desktop-based analyses of legal and policy frameworks, we conducted in-depth interviews with representatives of organizations providing adolescent HCT/SRH services, as well as academics, advocates, and policy makers, in the five countries. Interview data were analysed thematically and compared across specific issues and countries.

**Results:**

Laws regulating adolescent HCT, SRH and sexual activity are inconsistent and differentially interpreted within and across the five countries analysed. Conflicts exist between laws regulating age of consent to HCT and other SRH services, with the effect that adolescents have more barriers to preventative than diagnostic and curative services. Where consent to sex is regulated at a higher age than consent to HCT/SRH services, adolescents in effect need to disclose illegal sexual activity when accessing HCT/SRH services. Laws that criminalise homosexuality (in Malawi, Zambia and Zimbabwe) put lesbian, gay, bisexual and transgender adolescents at risk when attempting to openly access services. As a result, reporting obligations for healthcare providers with regards to teenage sexuality impede confidential service provision, and providers often use their discretion in deciding to whom and how to provide services.

**Conclusions:**

Laws and policies regulating HCT/SRH are often in conflict with HCT policies and create additional barriers for adolescents. National law and policy reforms need to be harmonized and aligned to the principles of adolescent-friendly HCT/ SRH services. Healthcare providers need to receive training on the legal frameworks and their obligations.

# Sub article: FRAD0103 Sensitizing judges on HIV, human rights and the law: the regional judges’ forum in Africa

**Published:** 2016-07-22

**Authors:** A Saha, C Grant, P Patel, M Getahun, T Sellers

**Affiliations:** 1 HIV, Health & Development (BPPS), UNDP, Addis Ababa, Ethiopia; 2 Regional Centre for Africa, UNDP Consultant, Johannesburg, South Africa; 3 UNDP Regional Consultant for Africa, Rolleston, New Zealand; 4 UNDP Regional Centre for Africa Addis Ababa, HIV, Health & Development, Addis Ababa, Ethiopia; 5 UNDP Regional Service Centre for Africa, HIV, Health & Development, Addis Ababa, Ethiopia

## Abstract

**Introduction:**

Sub-Saharan Africa accounts for 69% of people living with HIV. In many African jurisdictions, people living with or affected by HIV encounter stigma, discrimination and violations of rights that increase the impact of HIV on their lives and create barriers to services. Protecting human rights is essential for access to services. The judiciary can play a critical role in upholding the rights of people infected and affected by HIV. In 2014, African judges requested support to form a regional judges’ forum to learn about new developments in HIV-related jurisprudence and to share challenges they face.

**Description:**

UNDP supported two meetings of the Africa Regional Judges’ Forum in 2014 and 2015. Judges chaired sessions on HIV science, key populations, HIV laws, human rights, case law related to people living with HIV (PLHIV), men who have sex with men (MSM), sex workers, access to medicines, etc. Expert resource people, including HIV science and legal experts, and key population experts provided up to date information for the judges’ deliberations in these sessions.

**Lessons learned:**

The Forum was based on issues raised by judges, and provided a collegial space for them to identify participants, themes, and the agenda. Judges who have made ground-breaking rulings acted as resource persons, complemented by thematic and key population experts. Twenty-six judges from 16 African countries participated in these two meetings and discussed HIV science, human rights of key populations (sex workers, transgender people, MSM and prisoners) and access to antiretroviral therapy. Mock trials and case law discussions focused on issues like forced sterilization of women with HIV, discrimination against PLHIV in workplace, HIV treatment for prisoners and registration of LGBTI organizations. An online database on HIV laws/bills, case law, regional and international treaties and covenants, was launched.

**Conclusions/Next steps:**

Results (2014–2015): Participants of the Forum were resource persons at a training of court users in Kenya. Kenya High Court ruled criminalizing HIV transmission in law as unconstitutional; Botswana HC ruled that foreign prisoners were entitled to ART – a ruling that was upheld by the Court of Appeals; the South African Chapter of International Association of Women Judges developed a Strategic Plan that incorporated HIV, law, gender and human rights, and transgender issues.

# Sub article: FRAD0104 Enforcing the laws on public morality against key populations: the dilemma of the Ghana police service

**Published:** 2016-07-22

**Authors:** TS Ndeogo, J Blantari, E Awotwi

**Affiliations:** 1 Public Health Unit, Ghana Police Hospital, Accra, Ghana; 2 Ghana Police Hospital, Accra, Ghana; 3 United Nations Population Fund, Accra, Ghana

## Abstract

**Introduction:**

Ghana has recorded significant progress in the fight against HIV over the last decade with current infection rates plateauing around the prevalence of 1.47% among the general population aged 15–49 years. However, significant challenges exist amongst key populations (KPs) with prevalence of 11% (FSW) and 17% (MSM), which plays a key role in the HIV transmission dynamics. Laws criminalizing KP activities stand in the way of progress in addressing HIV. Application of the law is often based on prejudice, and not science/evidence. Lack of enforcement of protective laws is problematic, with increased intolerance and rights violations of sex workers.

**Description:**

To promote rights-based policing approaches towards KPs, the Ghana Police Service AIDS Control Program with support from UNFPA implemented orientation sessions in six police regions. The program targeted 611 police personnel from senior officer (SPO), inspectorate/middle and the operational levels. Sessions were organized through focus group discussions (FGD) to a) solicit information on how the police would identify a sexual minority, b) define laws that classify KPs, and c) understand what constitutes causation of sexual offences against public morals. Sensitization sessions immediately followed to address issues arising from the FGDs.

**Lessons learned:**

FDGs revealed respondents’ appropriate interpretation of the laws ranged from 77% (SPO) to 70% (inspectorate/middle) and 38% (operational). Participants could not state key aspects of the law in the Criminal Offences Act. Discussants dwelt on perceptions (including the way of dressing) rather than facts regarding the causation of sexual offence; with appropriateness of responses ranging from 63% (inspectorate/middle) to 59% (SPO) and 41% (operational). After sensitization sessions, participants accepted the need for reform and observed:

- senior officers who protect rights of KPs are compelled to arrest them due to political/societal pressure,
- morality should be decoupled from law enforcement,
- police must constantly dialogue with the judiciary to protect KP rights.

**Conclusions/Next steps:**

Acceptance of misconduct is a major breakthrough to reform. This program, with approval of the police hierarchy, is taking steps to punish subsequent perpetrators of abuse of KPs. Consensus building among the police and efficient use of available resources will help to sustain the program.

# Sub article: FRAD0105 Nothing about us without US: community-based action research to ensure HIV policy in the US reflects the experiences and needs of sex workers

**Published:** 2016-07-22

**Authors:** P Saunders, S Outlaw, D Demeri, J McCracken

**Affiliations:** 1 Best Practices Policy Project, Morristown, NJ, United States; 2 Best Practices Policy Project, Washington, United States; 3 New Jersey Red Umbrella Alliance, Atlantic City, United States; 4 University of South Florida, St. Petersberg, FL, United States

## Abstract

**Introduction:**

The project includes advocacy and organizing efforts led by US sex workers and the production of a series of reports to reveal how current HIV policies impact sex workers. An overarching goal of the project is to ensure that sex workers are acknowledged in the National AIDS Strategy as essential partners in ending HIV and to consider the impact of US policy on sex work worldwide.

**Description:**

The project is multi-pronged involving community building, documentation and advocacy. In 2015, a community based research team was established with key partner organization of sex worker lead groups. A survey was implemented with 25 US respondents about transgender people's experiences related to HIV. The emerging issues were explored with 40 respondents in open-ended interviews in person, phone, or email, ranging from 30 minutes to 2 hours. The team used statistical and thematic analysis of the qualitative data. Throughout 2015 team members built their capacity to engage in policy and media advocacy, developing a letter to the Office of National AIDS Strategy in partnership with other groups and created statement that were distributed widely in the media and the HIV sector.

**Lessons learned:**

The project findings include:

- that sex workers are not mentioned in the National AIDS Strategy of the United States, a silence that has contributed to a profound health and rights crisis for sex workers and people profiled as sex workers (such as transgender people).
- the criminalization of the lives of sex workers is the central barrier to health and rights.
- the movement for sex worker rights in the United States is incrementally developing its capacity to effectively shift policy discourse and to publicize best practice initiatives via media interventions.
- ongoing resources are required to begin to establish employment opportunities in sex worker-led organizations in the United States for sex workers and transgender people to build for change in the HIV sector.

**Conclusions/Next steps:**

This project is a milestone releasing the first US national report ever created by sex workers to confront the impact of outmoded and restrictive HIV policies. The project continues documenting the experiences of sex workers to guide our future work.

# Sub article: FRAD0106LB Interim outcomes of the New York Plan to End the AIDS Epidemic by the end of 2020: assessing a Fast Track model

**Published:** 2016-07-22

**Authors:** K Hagos, C King, V Shubert, D Daskalakis, D Tietz, M Harrington, K Smith, C Ferrusi

**Affiliations:** 1 New York State Department of Health, AIDS Institute, Albany, NY, United States; 2 Executive Management, Housing Works, Inc., Brooklyn, NY, United States; 3 Department: Research and Public Policy, Housing Works, Inc., Brooklyn, NY, United States; 4 Bureau of HIV/AIDS Prevention and Control, New York City Department of Health and Mental Hygiene, Long Island City, NY, United States; 5 Treatment Action Group (TAG), New York, NY, United States; 6 Administration, Callen-Lorde Community Health Center, New York, NY, United States; 7 Office of Grants and Data Management, AIDS Institute, New York State Department of Health, New York, NY, United States

## Abstract

**Introduction:**

In 2014, a coalition of government and civil society representatives secured a commitment by New York State's (NYS) Governor to end AIDS as an epidemic by the end of 2020. NYS has defined the end of the epidemic as reducing the number of new HIV infections to just 750 (from an estimated 3000) by the end of 2020. Once the number of new infections has fallen below all-cause mortality among people with HIV, NYS will achieve the first ever decrease in HIV prevalence in New York State.

**Methods:**

A state-appointed Task Force of government and community stakeholders developed a detailed Ending the Epidemic (ETE) Blueprint to: identify persons who remain undiagnosed; link and retain diagnosed persons in care to maximize viral suppression; and broaden access to pre-exposure prophylaxis (PrEP). The Blueprint builds upon a unique NYS HIV response that includes housing and nutrition supports, harm reduction programming, and social marketing to address stigma and promote testing, treatment and prevention. Public health agencies work closely with civil society to implement this Fast Track agenda, and an online “ETE Dashboard” tracks key metrics for accountability and planning.

**Results:**

In NYC, which represents 80% of new diagnoses and persons living with HIV in NYS, HIV surveillance data indicate that 72% of an estimated 87,000 persons infected with HIV are virally suppressed (≤200 copies/mL), and findings from a multi-year survey of men who have sex with men (MSM) show PrEP awareness at 86% in 2015 (up from 34% in 2012) and PrEP use among MSM at 16% (up from 1.6% in 2012 and 2.8% in 2014). NYS has documented a 40% reduction in new HIV infections over the last decade, and preliminary data shows NYS recently went 17 months with no new cases of mother to child transmission for the first time since the outbreak of the disease.

**Conclusions:**

Recent analyses indicate significant ongoing improvements in HIV treatment effectiveness and in comprehensive prevention awareness and use, putting New York on a path to end its HIV epidemic by the end of 2020 via replicable strategies to dramatically reduce new HIV infections and end AIDS deaths.

# Sub article: FRAD0201 Empirical impact of constitutional rights protections on HIV-related health systems and availability of essential medicines

**Published:** 2016-07-22

**Authors:** M Kavanagh

**Affiliations:** 1 Political Science Department, University of Pennsylvania, Philadelphia, PA, United States; 2 Leonard Davis Institute of Health Economics, University of Pennsylvania, Philadelphia, PA, United States

## Abstract

**Introduction:**

While there is much support among ethicists for a “rights based” approach to HIV treatment, care and prevention, there is also significant social science debate about the real-world effects of enshrining health as a right within national legal frameworks. Rights protection might empower populations to demand quality access to critical HIV services. Or, as critics argue, engaging law and courts might have no real impact or, at worst, distort policy away from public health goals toward individual club-goods for those with access to lawyers. This research empirically tests the hypothesis that protecting health as a right improves access to HIV treatment and quality of service provision in the public health setting.

**Methods:**

This study uses a mixed-methods political economy approach with two nested stages based on a large-N dataset and 125 in-depth interviews conducted in South Africa, India, and Thailand. A dataset coding all written constitutions in the world from 1970 to 2010 for an enforceable right to health was analyzed as a variable in a multi-level regression (OLS, FGLS, and ADL models) against variables capturing the common social, economic, and political explanations for cross-national variation in mortality and public goods provision. The results of this analysis were then tested against data gathered in “process tracing” interviews and archival research to identify causal mechanisms for the impact of rights on health services.

**Results:**

This study finds an empirically visible, significant, positive impact of protecting health as a constitutional right on the level, quality, and accessibility of HIV-related health services including access to antiretroviral therapy. A small, but statistically significant impact can be quantified on the availability of HIV- and non-HIV-related essential medicines, out of pocket expenses, and health workforce. Qualitative evidence shows that constitutionalizing health shifts the political economy of HIV by providing an avenue to challenge failures in health governance at the national and local level and, contrary to worries of upper/middle-class capture, has been largely utilized to improve quality and accessibility of public services.

**Conclusions:**

Protecting health as a right and building the institutional capacity to enforce that right should be understood as an important HIV and health-system strengthening intervention.

# Sub article: FRAD0202 The Free Trade Agreement that will adversely impact access to generic medicines in the Asia-Pacific … and no, it's not the TPP!

**Published:** 2016-07-22

**Authors:** K Bhardwaj

**Affiliations:** HIV, Health and Human Rights, New Delhi, India

## Abstract

**Introduction:**

Regional Comprehensive Partnership (RCEP) Agreement is between 16 countries in Asia-Pacific: developed (Japan, Australia, New Zealand, Singapore, South Korea), developing (China, India, Malaysia, Indonesia, Philippines, Thailand, Vietnam, Brunei Darussalam) and least developed countries (LDCs) (Myanmar, Cambodia, Laos). Scale-up of HIV treatment was only possible due to affordable generic medicines which have been adversely impacted by the WTO-mandated patent-regime. Developing countries and LDCs are advised to use trade-related aspects of intellectual property rights (TRIPS) flexibilities to ensure continued generic production/import. TRIPS-plus measures that are included in FTA negotiations require patent protection far in excess of that required by the WTO regime.

**Methods:**

The study examined four leaked texts of the IP proposals of Japan, South Korea, India and ASEAN to identify TRIPS-plus demands. It also compiled the use of TRIPS flexibilities by countries in the region and analysed the changes that would be required as a result of TRIPS-plus demands and their potential impact on access to generic medicines in the region and beyond.

**Results:**

The study found multiple TRIPS-plus demands in the RCEP negotiations including: (a) substantive demands requiring governments patent news uses/forms of old medicines (evergreening); (b) enforcement demands that would impact the export and transit of generic medicines; (c) demands limiting the ability of patent offices to require crucial information from patent holders; (d) demands impacting LDCs. TRIPS-plus demands prevent countries from using TRIPS flexibilities. In the region, Malaysia (2003), Indonesia (2004, 2007, 2012), Thailand (2006, 2008) and India (2012) have issued compulsory licenses for HIV, heart disease, cancer medicines. India, Philippines and Thailand restrict evergreening patents. RCEP countries feature some of the most important generic producers that the world relies on like China (API) and India (API, finished formulations) while Thailand has government production of medicines. If TRIPS-plus in RCEP is accepted, repercussions will be felt far beyond Asia-Pacific.

**Conclusions:**

One in three people living with HIV (PLHIV) have access to treatment in Asia-Pacific. HIV-positive pregnant women in South Asia have the world's lowest rate of access to ARVs needed for prevention of mother-to-child transmission (PMTCT). In several countries (China, Indonesia, Philippines) rates of new infections and AIDS-related deaths are increasing. As middle-income, developing countries are facing severe funding cuts from Global Fund, MNC pharma is withdrawing lower prices/excluding from voluntary licenses. RCEP will make a bad situation worse and TRIPS-plus demands should be rejected outright and countries should pro-actively make greater use of TRIPS-flexibilities to ensure access to generic HIV, hepatitis C and TB treatment.

# Sub article: FRAD0203 Access to ARVs and South African Patent Law Reform: reflection and ways forward for the Fix the Patent Law Campaign

**Published:** 2016-07-22

**Authors:** C Tomlinson, Y Hu, C Waterhouse, L Rutter

**Affiliations:** 1 Fix the Patent Law Campaign, Cape Town, South Africa; 2 Médecins Sans Frontières, Access Campaign, Geneva, Switzerland; 3 Médecins Sans Frontières, Access Campaign, Johannesburg, South Africa; 4 Treatment Action Campaign, Johannesburg, South Africa

## Abstract

**Introduction:**

Over the past 15 years, South Africa has been a pioneer of expanding generic medicine access, with constant battles led by civil society advocates to challenge patent monopolies of pharmaceutical companies that block sustainable access to affordable ARVs. Yet, in South Africa, many access challenges for newer ARVs and other medicines needed by people living with HIV remain, while ongoing patent law reform has triggered intensive debates and advocacy.

**Description:**

This research will examine strategies pursued by civil society organizations in South Africa over the past 15 years, their impact on medicines access and lessons learned. It will further review the ongoing reform of South Africa's intellectual property (IP) system, and the opportunities and challenges to adopting pro-public health patent laws.

**Lessons learned:**

Over the past 15 years, significant victories have been won through strategies employed by civil society organizations to secure access to generic ARVs in South Africa, including filing competition commission cases and calling for the granting of compulsory licenses. Today, a first line ARV regimen is 96% cheaper than in 2000, supporting the scale-up of treatment. However, fighting access battles drug-by-drug on an *ad hoc* basis has not changed systemic problems, such as current patent law lacking accommodation of public health needs. Seeking to adopt a more systemic approach to improve accessibility of medicine for all, the Fix the Patent Laws Coalition was founded in 2011, with 18 patient groups joining to date. During 2013, the South African government released a draft policy committing to pro-public health reform of the country's intellectual property laws. Pharmaceutical companies have responded with various attempts to derail such reform.

**Conclusions/Next steps:**

South Africa is at a critical stage in the ongoing battle for access to medicine versus expanding intellectual property protection. While there is opportunity for broad legislative reform to facilitate access to newer ARVs and all medicines there is significant push back from pharmaceutical companies. Fix the Patent Laws Coalition's experience demonstrates the need for greater international solidary and coordination with adequate technical and political support in reforming the national patent laws for public health and pushing back on pharmaceutical companies’ effort to thwart reform.

# Sub article: FRAD0204 Removing global patent barriers to the new generation of hepatitis C drugs

**Published:** 2016-07-22

**Authors:** T Amin, P Radhakrishnan, O Mellouk, L Di Giano

**Affiliations:** 1 Initiative for Medicines, Access, & Knowledge (I-MAK), Lewes, DE, United States; 2 ITPC, Marrakesh, Morocco; 3 Fundacion GEP, Mara Del Plata, Argentina

## Abstract

**Introduction:**

Globally, at least 4–5 million people living with HIV (PLHIV) are co-infected with the hepatitis C virus (HCV). As other AIDS-related deaths decrease with increased ART use, the burden of HCV-related morbidity and mortality in PLHIV has become the leading cause of death. HCV can be cured in 12 weeks or less by combinations of highly effective direct-acting antivirals (DAAs), but access to these drugs is virtually non-existent in low- and middle-income countries (LMICs).

Non-sub Saharan African (non-SSA) MICs are excluded from discount programs and voluntary licenses, resulting in prices significantly higher than generics. Patent laws often do not have sufficient safeguards to enable generic access. DAA/ARV voluntary licenses restrict generic suppliers from providing treatment to over 40 MICs, including those with the highest number of people living with HCV. With the branded company as the only option, lack of competition keeps DAA prices out of reach for non-SSA MICs, whose governments are unable to fund treatment at the high branded prices.

**Description:**

This presentation will discuss the coordinated global legal effort to challenge patents on key DAAs, with a special focus on Sovaldi, known by its generic name sofosbuvir. This case study will share the scientific and legal basis for patent challenges, raise important questions about innovation and access, explain civil society′s rationale and process for challenging patents, showcase new impact analysis and conclude with recommendations for the way forward.

**Lessons learned:**

1. Utilizing patent challenges as strategy to address treatment gap has proven successful.
2. Community networks have significant capacity to take patent work forward.
3. Government intervention is a necessary parallel strategy in curtailing the problem of drug pricing in developed and developing countries

**Conclusions/Next steps:**

1. Simultaneous coordinated patent challenges is the most effective strategy to combat the exclusion of MICs from access programs and voluntary licenses, which increases pressure on pharmaceutical companies, but also strengthens the position of country patent offices and governments in addressing patents and public health.
2. Only by challenging these patents, through stronger patent examination and patent challenges, there can be real change in addressing the challenges facing the current patent system and public health.

# Sub article: FRAD0205 Access to antiviral drugs for treating HCV for HIV-positive patients in Russia: results and recommendations of the registration, policy and procurement analysis

**Published:** 2016-07-22

**Authors:** K Babikhina, S Golovin

**Affiliations:** 1 Treatment Preparedness Coalition, Moscow, Russian Federation; 2 Treatment Preparedness Coalition, St. Petersburg, Russian Federation

## Abstract

**Introduction:**

The number of people with HCV in Russia is 5 million based on expert estimates. At least 200,000 people are co-infected with HIV and HCV. Treatment of chronic hepatitis C should be provided free of charge for the HIV/HCV co-infected patients within the framework of the national programme for HIV, HCV and HBV. “Treatment Preparedness Coalition” analyzed the regulatory framework and data on the procurement of HCV drugs to understand the scope and nature of HCV treatment provided for HIV/HCV co-infected people.

**Methods:**

We used the data of the website grls.rosminzdrav.ru to identify HCV drugs registered in Russia (December 2015). Then, we compared the results with the list of drugs in the decree on financing the procurement of HIV/HCV/HBV drugs (Decree No 1438). Then, we analyzed 850 contracts for HCV drugs concluded in 2015. The analysis included three non-proprietary names of pegylated interferon (PEG-IFN) and four non-proprietary names of direct-acting antivirals (DAAs).The number of patients was calculated by dividing the total amount of items purchased by the recommended daily dose and treatment duration.

**Results:**

In 2015, the following DAAs were registered in Russia: simeprevir; daclatasvir; asunaprevir; paritaprevir/ritonavir; dasabuvir; ombitasvir; telaprevir; and boceprevir. Three international non-proprietary names of PEG-IFN were registered (December 2015): PEG-IFN alpha-2a, PEG-IFN alpha-2b and Ce-PEG-IFN alpha-2b. The list of drugs in Decree No 1438 included only the three pegylated interferons. The other drugs were purchased using funds allocated for other groups of patients, including monoinfected patients.

In total, approximately 600 patients could receive interferon-free therapy. The total number of patients who could receive DAA-based therapy with interferon, including interferon-free therapy, is approximately 1000. The price for the treatment course of DAAs is in the range of 10,000–12,000 USD. The number of patients receiving pegylated interferon is in the range of 4500–9000 (depending on the treatment duration).

**Conclusions:**

The majority of HIV/HCV co-infected patients still receive PEG-IFN-based therapy. In our opinion, this is mostly due to high price of DAAs. Some preferred options for treating HCV are still not registered in Russia, including sofosbuvir and sofosbuvir/ledipasvir. None of the DAAs are included in Decree No 1438.

# Sub article: FRAD0206 Compulsory licensing as an ongoing alternative: comparing price negotiations for lopinavir/ritonavir (LOP/r) and efavirenz (EFV) in Brazil

**Published:** 2016-07-22

**Authors:** E Fonseca, F Bastos

**Affiliations:** 1 School of Business Administration of São Paulo, Fundacao Getulio Vargas, Sao Paulo, Brazil; 2 Fundacao Oswaldo Cruz, Rio de Janeiro, Brazil

## Abstract

**Introduction:**

Brazil′s AIDS response has been cited as the developing world′s largest and most innovative AIDS treatment program, but has been facing challenges due to budget cuts and political instability in recent years. In the 2000s, Brazil took controversial decisions to reduce the cost of providing free access to ARVs, such as price negotiations of patented drugs with multinational pharmaceutical companies and, for the first time in Latin America, launched compulsory licensing (CL). This study compares the price negotiations of LOP/r and EFV, as well as the underlying mechanisms that may explain the decision to grant CL for EFV but not for LOP/r.

**Methods:**

We used a chronological policy-analysis narrative. This study profits from first-hand empirical data collected in 2012/15, including governmental documents (e.g. legislation, congressional speeches and debates, etc.), newspaper articles, and 20 interviews with key informants.

**Results:**

In 2003, LOP/r and EFV represented 37% of total ARVs expenditure in Brazil. Between 2001 and 2005, procurement of LOP/r surpassed EFV in terms of costs and purchase volume. LOP/r was crucial for 2nd line treatment as it reduces the number of pills/per day and side effects. Given the costs associated with LOP/r and EFV and the failed negotiations with their producers, the MoH declared both medicines “of public interest” in 2005 and 2007, respectively. However, CL was issued just for EFV. The negotiations of LOP/r were conducted with little participation of civil society and public laboratories, and in a context of institutional transition in the MoH. On the other hand, the negotiation of EFV was coordinated by MoH industrial policy experts, by the then newly created Secretary of Science, Technology and Strategic Health Inputs, in close collaboration with civil society and local producers. After importing generic copies of EFV, Brazil developed an innovative public-private partnership to produce EFV domestically.

**Conclusions:**

The generalizability of these findings depend on other countries’ intellectual property regimes and differing capacities for local drug production. Lessons from Brazil′s experience show that civil society support has been key in price negotiations and that cooperation between local manufactures may foster new opportunities for generic ARVs development.

# Sub article: FRAE0101 An exploratory assessment of the feasibility and acceptability of home-based support to streamline HIV pre-exposure prophylaxis (PrEP) delivery

**Published:** 2016-07-22

**Authors:** A Siegler, A Liu, K Mayer, K Thure, R Fish, E Andrew, M Gelman, P Sullivan

**Affiliations:** 1 Epidemiology, Emory University, Atlanta, GA, United States; 2 Bridge HIV, San Francisco Department of Public Health, San Francisco, CA, United States; 3 The Fenway Institute, Boston, MA, United States

## Abstract

**Introduction:**

PrEP is highly efficacious in preventing HIV transmission. The US Centers for Disease Control and Prevention, the Southern African HIV Clinicians Society and the European AIDS Clinical Society recommend quarterly HIV and periodic STI screening for those on PrEP. The burden of indefinite PrEP follow-up visits is high for providers, patients and the healthcare system. A home-based testing system could greatly streamline PrEP delivery and facilitate rapid scale-up.

**Methods:**

We conducted in-depth interviews and exit surveys in San Francisco, Boston and Atlanta with 12 medical providers and 16 participants, exploring the acceptability of a PrEP home care system that would be used in lieu of 1–3 of the quarterly provider visits per year. Participants were shown kit mock-ups and self-collected all specimens. Providers were shown laboratory and behavioural results from mock-ups. We discussed with all their interest in, willingness to use, and concerns regarding a home care kit for PrEP. We also conducted a brief exit survey.

**Results:**

Participants and providers had favourable reactions to the kit, with some participants less enthusiastic about home counselling because they felt it unnecessary (Figure). In total, 15/16 participants were able to self-collect all specimens necessary to conduct standard tests for HIV, STI and renal function. Across specimen collection methods, only 2/16 participants rated any as “difficult” or “very difficult.” One representative participant noted, “I would highly encourage this to be out in the world” and a provider noted, “I think it's great … we have to decentralize (care).

**Conclusions:**

There is strong interest in a PrEP home care system from both MSM on PrEP and providers prescribing PrEP. With a mocked-up kit, specimen collection was feasible and largely acceptable. Future research is needed to pilot test the home care kit in an unsupervised setting to determine adequacy of sample collection and acceptability of the overall home care system.

**Abstract FRAE0101–Figure 1:** Provider and patient interest in a PrEP home testing system and home counselling system.

# Sub article: FRAE0102 Good adherence in trial of topical pre-exposure prophylaxis integrated into family planning services

**Published:** 2016-07-22

**Authors:** LE Mansoor, Q Abdool Karim, KT Mngadi, C Montague, N Yende-Zuma, H Dawood, TN Gengiah, N Samsunder, C Baxter, JL Schwartz, GF Doncel, F Ntombela, A Grobler, SS Abdool Karim

**Affiliations:** 1 Centre for the AIDS Programme of Research in South Africa (CAPRISA), Durban, South Africa; 2 CONRAD, Arlington, VA, United States

## Abstract

**Introduction:**

Evidence guiding scale-up of pre-exposure prophylaxis (PrEP) in African women is required for implementation of new WHO guidelines. The CAPRISA 008 trial, which provided participants from CAPRISA 004 with post-trial access to tenofovir gel, assessed adherence and effectiveness of PrEP provision integrated into family planning (FP) services.

**Methods:**

CAPRISA 008 was a 36-month, two-arm, open-label, randomized controlled and non-inferiority trial. Eligible women (n=372) were randomly assigned to receive tenofovir gel at clinical trial clinics (n=183) or at FP clinics (n=189). Adherence, retention, HIV incidence and service preference were assessed.

**Results:**

At baseline, women assigned to trial and FP clinics were similar, and study retention rates were 92.3 and 92.1%, respectively. Adherence (% reported sex acts covered by 2 gel doses) was 73.9% (95% confidence interval (CI): 70.7–77.1) in trial clinics and 79.9% (CI: 76.7–83.2) in FP clinics. Higher adherence (mean difference=6.0% (CI: 1.5–10.6)) in FP clinics met the pre-defined non-inferiority criteria. Mean monthly sex acts and returned empty applicators were 4.5 (CI: 4.0–5.0) and 6.0 (CI: 5.5–6.5) in trial clinics compared to 3.6 (CI: 3.2–4.1) and 5.2 (CI: 4.7–5.7) in FP clinics respectively. Genital tenofovir was detected at 1 year in 68/156 women (43.6%; CI: 36.1–51.4) at trial clinics and 62/157 women (39.5%; CI: 32.2–47.3) at FP clinics (p=0.492). Adjusting for peri-coital gel use, genital tenofovir was detected in 58.3% of 139 women reporting sex within 7 days but only in 28.2% of 174 women reporting no recent sex. HIV incidence was 3.6 per 100 women years (wy) (CI: 1.9–6.3) in trial clinics and 3.5 per 100 wy (CI: 1.8–6.0) in FP clinics (p=0.928). Overall HIV incidence rate was 44% lower than in an age-comparable historical CAPRISA 004 placebo control group (3.5 vs. 6.2 per 100 wy). At study exit 75.1 and 80.3% of women from trial and FP clinics expressed preference for receiving PrEP from FP clinics.

**Conclusions:**

Integration of topical PrEP into FP services for African women is feasible, acceptable and achieves adherence equivalent to a clinical trial setting, providing evidence for PrEP scale-up using this strategy for this challenging high-priority population.

# Sub article: FRAE0103 Experiences of PrEP discontinuation in African HIV serodiscordant couples: qualitative results from The Partners Demonstration Project

**Published:** 2016-07-22

**Authors:** MA Wyatt, EE Pisarski, M Tam, E Nakku-Joloba, TR Muwonge, E Katabira, NC Ware

**Affiliations:** 1 Global Health and Social Medicine, Medical School, Harvard University, Boston, MA, United States; 2 Harvard Global, Cambridge, MA, United States; 3 Epidemiology and Biostatistics, School of Public Health, Makerere University College of Health Sciences, Kampala, Uganda; 4 STD Clinic/Ward 12, Mulago Hospital, Kampala, Uganda; 5 Infectious Diseases Institute, Kampala, Uganda; 6 School of Medicine, Makerere University College of Health Sciences, Medicine, Kampala, Uganda

## Abstract

**Introduction:**

Public health programmes worldwide are considering new WHO guidelines recommending antiretrovirals (ART) as pre-exposure prophylaxis (PrEP) for all individuals at substantial risk for HIV. Unlike ART, PrEP is taken by uninfected individuals during limited periods of high risk exposure and discontinued when transmission risk is reduced. Understanding user experiences of PrEP uptake and discontinuation will inform future implementation efforts. Using qualitative data from the Kampala, Uganda site of the Partners Demonstration Project, we describe user preferences for PrEP use and discontinuation.

**Methods:**

The Partners Demonstration Project was a prospective implementation study that evaluated an integrated strategy for delivering PrEP and ART to HIV serodiscordant couples. HIV-uninfected partners discontinued PrEP when ART had been taken by infected partners for 6 months. In-depth interviews were conducted with 48 serodiscordant couples from the Project. Interview topics included understandings of PrEP, adherence and experiences of PrEP use and discontinuation. Interviews were inductively analyzed to identify themes reflecting PrEP use and discontinuation. Categories representing themes were developed and organized sequentially to describe user preferences for discontinuing PrEP.

**Results:**

Initially, participants expressed doubts about taking PrEP. However, as PrEP use became routine, users gained confidence in PrEP's capacity to protect them against HIV infection. PrEP gave uninfected individuals a newfound sense of control over their prevention methods, and ultimately, their health. When PrEP was discontinued, some users felt once again vulnerable to HIV acquisition. Reasons for this heightened sense of risk included lack of confidence in ART to prevent transmission, doubts about partners' adherence to ART and fear of risk through unprotected sex outside the partnered relationship. Most users preferred to remain on PrEP or be given the opportunity to reinitiate PrEP in the future.

**Conclusions:**

Uninfected partners in HIV serodiscordant relationships offered PrEP as part of an integrated PrEP and ART delivery strategy preferred to exercise control over their prevention choices. Resistance to discontinuing PrEP may present a challenge to efficient and effective implementation of PrEP in public health settings of resource scarcity. Messaging about PrEP discontinuation should include explanations of how ART prevents HIV transmission after PrEP is discontinued, while also addressing user concerns about risk.

# Sub article: FRAE0104 Eliminating barriers to increase uptake of PrEP in a community-based clinic in San Francisco

**Published:** 2016-07-22

**Authors:** S Gibson, P-C Crouch, J Hecht, J Gagliano, T Patriarca, J Auerbach, R Grant, W Lyon, C Hall

**Affiliations:** 1 San Francisco AIDS Foundation, San Francisco, CA, United States; 2 University of California, San Francisco, CA, United States

## Abstract

**Introduction:**

The US Food and Drug Administration approved Pre-Exposure Prophylaxis (PrEP) for HIV prevention in July 2012. As demand for PrEP increased in San Francisco, barriers to access surfaced: lack of provider knowledge of PrEP, finding a culturally competent provider, issuance of same-day prescriptions and coverage of laboratory and medication costs. San Francisco AIDS Foundation (SFAF) launched a PrEP programme in 2014 to address barriers, increase access and reduce new HIV infections among MSM.

**Description:**

The programme, led by Nurse Practitioners, leverages Registered Nurses and volunteer HIV test counsellors (HTC) trained to provide culturally competent care. Clients self-refer or are referred by HTC. NPs perform a full medical evaluation and PrEP counselling, addressing adherence and stigma. Utilizing point-of-care HIV and chemistry testing, clients initiate PrEP the same-day. Follow-up visits are conducted by RNs, providing ongoing PrEP and adherence counselling. Abnormal lab results are referred to the NP for evaluation. MDs are available for consultation as needed. Benefits Navigators work with medically eligible clients to access PrEP through applying for full-assistance programmes, activating copay cards and initiating health insurance. Navigators interface with insurance companies when clients are met with barriers. There is no cost for the lab work and evaluation services. With the available benefit programmes, most clients obtain PrEP at no or very low cost.

**Lessons learned:**

Between November 2014 and January 2016, 797 participants enrolled in the programme and 95% received a prescription for Truvada. 89% reported condomless anal sex in the past 12-months. 69% were Caucasian, 22% were Latino/Hispanic, 6% were African-American and 11% were Asian/Pacific Islander. Mean age was 34.4 years. 25% were treated for an STI at enrolment. There were no new HIV infections among participants in the PrEP programme. Comparatively, the clinic diagnosed 54 new HIV infections among men not enrolled in the programme during the same time period.

**Conclusions/Next steps:**

A community-based organization, led by NPs, RNs, benefits navigators and HTCs can determine if clients are medically eligible to begin PrEP, assist with identifying and addressing barriers to accessing PrEP so clients can safely initiate medication the same-day, contributing to fewer HIV diagnoses among MSM in San Francisco.

# Sub article: FRAE0105 Expanded PrEP implementation in communities in NSW, Australia (EPIC-NSW): evidence-based implementation study

**Published:** 2016-07-22

**Authors:** I Zablotska, AE Grulich, R Guy, J Amin, C Selvey, H-M Schmidt, K Price, D Cooper, EPIC-NSW Study Group

**Affiliations:** 1 The Kirby Institute, University of New South Wales (UNSW), Sydney, Australia; 2 NSW Ministry of Health, North Sydney, Australia; 3 ACON, Sydney, Australia

## Abstract

**Introduction:**

In New South Wales (NSW) Australia, the new HIV Strategy 2016–2020 aims to virtually eliminate HIV transmission by 2020, and HIV pre-exposure prophylaxis (PrEP) is identified as a key tool. A partnership of key NSW government, community, medical and research organizations has taken an innovative approach to rapid scale-out of PrEP implementation and evaluating the strategy. We describe PrEP implementation issues, innovative solutions and evidence-based design of the NSW PrEP implementation study.

**Description:**

The number of people meeting the PrEP-eligibility risk criteria, defined in the existing NSW PrEP guidelines, was estimated using data from previous Australian studies to be 3700. Calculations of HIV transmission probability by behavioural risk factor estimated that new HIV diagnoses in NSW would be reduced by ~50% in 12 months by preventing infection in these people with PrEP. The NSW partnership designed the expanded PrEP implementation in communities in NSW (EPIC-NSW) study which aims to provide PrEP to all 3700 people and evaluate PrEP implementation within the new HIV strategy. The study features:

1. large-scale, rapid roll-out (March–September 2016);
2. real-life implementation (service procedures per guidelines);
3. innovative data collection with minimal burden on health services (extraction and ongoing linkage of data from clinical data collection systems);
4. cumulative follow-up of 7400 person-years, and
5. assessment of the trial impact on HIV incidence in the cohort and the general population.

We will report on EPIC-NSW design features and roll-out and estimated impact on HIV incidence.

**Lessons learned:**

Mobilization and partnership of the entire HIV sector is crucial for rapid PrEP roll-out and beneficial for establishing access to PrEP. An evidence-based approach, community mobilization and creative use of available data sources enable efficient and effective implementation and evaluation of public health strategies.

**Conclusions/Next steps:**

EPIC-NSW is the only PrEP implementation trial internationally aimed at monitoring PrEP impact at the population level. Data from this study will evaluate the state HIV prevention strategy and PrEP contribution in reaching the goal of virtually eliminating HIV by 2020. The design and results of this trial will inform policy, investment, community education and interventions in other similar settings internationally.

# Sub article: FRAE0106LB Optimizing the frequency of kidney safety monitoring in HIV-uninfected persons using daily oral tenofovir disoproxil fumarate pre-exposure prophylaxis

**Published:** 2016-07-22

**Authors:** K Mugwanya, R Heffron, C Wyatt, N Mugo, C Celum, J Kiarie, E Katabira, A Ronald, J Baeten, for the Partners PrEP Study and Partners Demonstration Project Teams

**Affiliations:** 1 Department of Global Health, University of Washington, Seattle, WA, United States; 2 Medicine, Icahn School of Medicine at Mount Sinai, New York, NY, United States; 3 Kenya Medical Research Institute, Nairobi, Kenya; 4 Department of Obstetrics and Gynecology, University of Nairobi, Nairobi, Kenya; 5 School of Medicine, Makerere University, Kampala, Uganda; 6 University of Manitoba, Winnipeg, MB, Canada

## Abstract

**Introduction:**

Optimal kidney safety monitoring is a key knowledge gap for wide-scale implementation of tenofovir-based pre-exposure prophylaxis (PrEP) for HIV prevention. We compared 6-monthly to 3-monthly kidney monitoring for the occurrence of clinically relevant decline in creatinine clearance (CrCl; <60 mL/minute).

**Methods:**

Data were from two prospective PrEP studies in Kenya and Uganda: the Partners Demonstration Project (n = 955), a recently completed open-label study that used 6-monthly serum creatinine monitoring to estimate creatinine clearance, and the Partners PrEP Study, a placebo-controlled trial that used 3-monthly monitoring (n = 4404 receiving PrEP, n = 1573 receiving placebo). CrCl ≥60 mL/minute was required for enrolment in both studies.

**Results:**

With 6-monthly monitoring, the cumulative proportion of participants with unconfirmed CrCl <60 mL/minute was 0.7% at Month 6 and 1.1% at Month 12, affecting 10 (1%) participants; 2 of these (0.2% overall) had CrCl <60 mL/minute confirmed on repeat testing, both at Month 6. With quarterly monitoring, the cumulative proportion of participants with unconfirmed CrCl <60 mL/minute was 1.4% at Month 3, 2.0% at Month 6, and 2.7% at Month 12, affecting 120 (2.7%) participants; 29 of these (0.7%, overall) had CrCl <60 mL/minute confirmed on repeat testing (cumulative proportion: 16 (0.4%), 21 (0.5%), and (0.7%) at Months 3, 6, and 12, respectively). The corresponding cumulative frequency of confirmed CrCl <60 mL/minute in the placebo group was 0.3% at Month 3 and 0.3% at Month 6. Of the 29 participants experiencing confirmed declines in the Partners PrEP Study, 28 (97%) had baseline CrCl 60–90 mL/minute, 19 (66%) were aged ≥45 years, and 16 (55%) had baseline weight ≤55 kg (adjusted p <0.05).

**Conclusions:**

In these two large cohorts of HIV-uninfected persons using PrEP, the occurrence and pattern of clinically relevant decline in CrCl were not qualitatively different based on quarterly or 6-monthly CrCl monitoring. Most measurements of CrCl <60 mL/minute did not confirm on repeat testing. These data suggest that 6-monthly CrCl monitoring could be equally safe and require fewer resources for a majority of persons receiving PrEP, with more frequent monitoring potentially indicated for those with specific risk factors (older age, lower baseline CrCl, lower weight).

# Sub article: FRAE0201 Six-monthly appointments as a strategy for stable antiretroviral therapy patients: evidence of its effectiveness from 7 years of experience in a Médecins Sans Frontières supported programme in Chiradzulu district, Malawi

**Published:** 2016-07-22

**Authors:** C Cawley, S Nicholas, E Szumilin, S Perry, I Amoros Quiles, C Masiku, A Wringe

**Affiliations:** 1 London School of Hygiene & Tropical Medicine, London, United Kingdom; 2 Epicentre (Médecins Sans Frontières), Paris, France; 3 Médecins Sans Frontières, Paris, France; 4 Médecins Sans Frontières, Lilongwe, Malawi

## Abstract

**Introduction:**

HIV clinics are struggling to absorb new patients in Malawi, and overburdened health-workers and long waiting times can be detrimental to adherence. We evaluated a strategy of six-monthly appointments (SMA) for stable ART patients in Chiradzulu District, Malawi, where Medecins sans Frontieres is supporting the Ministry of Health's HIV programme.

**Methods:**

Stable patients (aged ≥15, on first-line ART ≥12 months, CD4 count ≥300 and without opportunistic infections or ART intolerance, not pregnant or breastfeeding) were eligible for clinical assessments every 6 months instead of 1–2 months at 11 HIV clinics. Early SMA enrolees were defined as patients who started SMA within 6 months of eligibility, late SMA enrolees were those starting >6 months after eligibility. Kaplan-Meier methods were used to calculate cumulative probabilities of death and loss to follow-up (LTFU) among those eligible for SMA, stratifying by SMA enrolment status and baseline characteristics. Cox regression, using SMA enrolment as a time-dependent variable, was used to estimate crude and adjusted hazard ratios for the association between SMA and death or LTFU.

**Results:**

Between 2008 and 2015, 18,957 individuals were eligible for SMA (contributing 43,888 person-years of observation), of whom 15,308 (80.8%) ever enrolled. Median time from SMA eligibility to enrolment was 6 months (interquartile range 0–17 months). The cumulative probability of death or loss to follow-up 5 years after first SMA eligibility was 56.3% (95% confidence interval (CI): 52.4–60.2%) among those never SMA enrolled; 13.9% (95% CI: 12.5–15.6%) among early SMA enrolees and 8.1% (95% CI: 7.2–9.0%) among late SMA enrolees.

After adjusting for age, gender, year of first SMA eligibility and other baseline variables (CD4 count, months on ART and in cohort), a significantly higher rate of death or LTFU was observed among patients during non-SMA periods compared to those during SMA periods (adjusted rate ratio: 1.87, 95% CI: 1.68–2.08, p<0.001).

**Conclusions:**

SMA represents a promising strategy for managing stable ART patients and should be rolled out, particularly with “test and treat” on the horizon, which will further stretch HIV clinics. However, further implementation research is needed, and selection biases which may explain poor retention among those eligible but never SMA-enrolled should be investigated.

# Sub article: FRAE0202 Improved survival and retention in HIV treatment and care: the value of community ART groups for HIV patients on ART in rural northern Mozambique

**Published:** 2016-07-22

**Authors:** LF Jefferys, J Hector, MA Hobbins, J Ehmer, N Anderegg

**Affiliations:** 1 SolidarMed, Pemba, Mozambique; 2 SolidarMed, Lucern, Switzerland; 3 Institute of Social and Preventive Medicine, University of Bern, Bern, Switzerland

## Abstract

**Introduction:**

Community ART groups (CAG) allow patients to pick up medication on a rotational basis and have been implemented as a strategy to improve adherence of HIV-positive patients on combination antiretroviral therapy (ART) in Mozambique. Participation in a CAG is voluntary but guided by inclusion criteria. The purpose of this analysis was to review the association between baseline characteristics and joining a CAG, and to examine the benefit of CAGs on mortality and lost to follow-up (LTFU).

**Methods:**

This observational study was conducted in Ancuabe, Mozambique. We included all HIV-positive adults (≥15 years) starting ART 2010–2015, that met the CAG eligibility criteria (non-pregnant, follow-up ≥6 months). Multivariable logistic regression was used to examine associations between joining a CAG and the baseline characteristics sex, age, WHO stage and CD4 cell count at baseline as well as the total days late for appointments within the first 6 months before being eligible for CAG-participation. Mortality rates and the risk of being LTFU between CAG-participants and non-participants were examined using cox proportional hazards regression, adjusted for all baseline covariates.

**Results:**

A total of 1306 patients were included (62.9% female) with a median CD4 cell count of 257 cells/µl (interquartile range (IQR): 149–352), a median age of 33.1 years (IQR: 26.2–41.3) and a median of 23 days late within first 6 months (IQR: 6–49). During 2866 person-years, 10.5% of patients died, 22.6% were LTFU and 13.8% joined a CAG. The odds of joining a CAG were increased by female sex (odds ratio (OR): 1.73, 95%-confidence interval (CI): 1.21–2.46) and an older age (OR: 1.02, 95% CI: 1.01–1.03); no other baseline covariate showed a significant association with CAG-participation. CAG-participation reduced the mortality rate by 55.1% (adjusted hazard ratio (aHR): 0.449, 95% CI: 0.264–0.762) and the risk of being LTFU by 84.3% (aHR: 0.157, 95% CI: 0.086–0.288).

**Conclusions:**

Patients that were in a CAG did not have significantly different baseline CD4 cell count or adherence to appointments in the first 6 months of treatment than those not entering a CAG, however despite this CAG-participation remarkably lowered the risk of both being LTFU and dying. These results support the implementation of CAGs in rural settings.

# Sub article: FRAE0203 SEARCH streamlined HIV care is associated with shorter wait times before and during patient visits in Ugandan and Kenyan HIV clinics

**Published:** 2016-07-22

**Authors:** SB Shade, W Chang, JG Kahn, D Mwai, F Mwangwa, D Kwarisiima, A Owaraganise, J Ayieko, DV Havlir, MR Kamya, ED Charlebois, ML Petersen, TD Clark, EA Bukusi, CR Cohen, V Jain

**Affiliations:** 1 Center for AIDS Prevention Studies, University of California, San Francisco, San Francisco, CA, United States; 2 Institute for Health Policy Studies, University of California, San Francisco, CA, United States; 3 Health Economics Unit, School of Economics, University of Nairobi, Nairobi, Kenya; 4 Infectious Diseases Research Collaboration, Kampala, Uganda; 5 Makerere University, Kampala, Uganda; 6 Kenya Medical Research Institute, Nairobi, Kenya; 7 University of California, HIV, ID and Global Medicine, San Francisco, CA, United States; 8 University of California, Berkeley, CA, United States; 9 Department of Obstetrics, Gynecology & Reproductive Sciences, Bixby Center for Global Reproductive Health, University of California, San Francisco, CA, United States

## Abstract

**Introduction:**

Long patient wait time is reported as an operational barrier to retention in HIV care in resource-limited settings. Patients may perceive waiting several hours to see a clinician for only a few minutes as an unacceptable opportunity cost. The SEARCH HIV test-and-treat cluster randomized trial (NCT:01864603) in 32 rural Ugandan and Kenyan communities is implementing a “streamlined” HIV care delivery model in government-supported clinics that aims to reduce wait times to address this problem.

**Methods:**

We examined differences in patient wait time before and during clinical visits conducted under “streamlined” and standard government HIV clinic care. Components of streamlined HIV care aimed at reducing wait time included:

1. nurse-driven triage for patient evaluation;
2. 3-month ART refills (vs. 1 or 2 month) for stable patients; and
3. consolidation of services at encounter (ART, phlebotomy, medication dispensing).

We conducted a time-and-motion study of patient clinical visits. We compared mean patient wait time before and during clinical visits among SEARCH study patients with CD4 =500 cells/µl (n=119), SEARCH patients with CD4<500 cells/µl (n=234) and other government clinic patients (n=745).

**Results:**

Mean visit length was over 1 hour shorter among SEARCH patients with CD4 ≥500 cells/µl and SEARCH patients with CD4 <500 cells/µl compared to other government clinic patients, even though mean time with providers was similar between groups (see Table). This difference was due to wait times that were >30 minutes shorter both before and during visits. Time spent receiving health education, HIV care, laboratory services, medication dispensing and other services did not differ between patient groups.

**Conclusions:**

Streamlined HIV care delivery led to shortened wait times both before and during HIV clinic visits. These efficiency improvements may contribute towards improved retention in HIV care.

**Abstract FRAE0203–Table 1:** Mean wait time and time receiving services by patient types

	SEARCH Patients CD4=500 (hours:minutes) mean (SD)	SEARCH Patients CD4<500 (hours:minutes) mean (SD)	Other government clinic patients (hours:minutes) mean (SD)
Total visit length	1:08 (1:02)	1:13 (1:03)	2:35 (1:33)
**Wait time before visit**	**0:21 (0:36)**	**0:28 (0:43)**	**1:13 (1:13)**
**Wait time during visit**	**0:19 (0:30)**	**0:23 (0:35)**	**0:58 (1:00)**
**Time receiving services**	**0:27 (0:24)**	**0:22 (0:20)**	**0:24 (0:29)**
–Health education	<0:01 (0:03)	0:01 (0:07)	0:08 (0:21)
–HIV care	0:18 (0:18)	0:12 (0:11)	0:08 (0:13)
–Laboratory services	0:03 (0:09)	0:01 (0:08)	0:01 (0:08)
–Medication dispensing	0:01 (0:02)	0:03 (0:07)	0:04 (0:10)
–Other	0:03 (0:09)	0:03 (0:09)	<0:01 (0:05)

# Sub article: FRAE0204 Implementation of combination ART refills models in rural Swaziland

**Published:** 2016-07-22

**Authors:** L Pasipamire, B Kerschberger, I Zabsonre, S Ndlovu, G Sibanda, S Mamba, S Mazibuko, N Lukhele, SM Kabore, B Rusch

**Affiliations:** 1 Médecins Sans Frontières (MSF), Nhlangano, Swaziland; 2 Médecins Sans Frontières (MSF), Matsanjeni, Swaziland; 3 Médecins Sans Frontières (MSF), Hlatikulu, Swaziland; 4 Swaziland National AIDS Programme, Mbabane, Swaziland; 5 Médecins Sans Frontières (MSF), Mbabane, Swaziland; 6 Médecins Sans Frontières (MSF), Geneva, Switzerland

## Abstract

**Introduction:**

The WHO advocates for differentiated HIV care and considers a broad range of community-based care models for patients stable on anti-retroviral therapy (ART). These care models aim to better respond to patient needs and to alleviate pressure on health systems caused by rapidly growing patient numbers. Most settings, however, utilized a single community-based care model only. We operationalize a combination of community ART care models in public health sector and assessed early outcomes.

**Methods:**

Three community ART delivery care models were deployed in the rural Shiselweni region (Swaziland), from 02/2015 to 12/2015. First, treatment clubs (TC) are groups of 30 patients stable on ART who meet every 3 months at a secondary health facility for patient education and drug-refills. Second, community ART groups (CAG) comprise a maximum of six patients who alternate to attend the primary health clinic for consultation and pick up drugs for the other group members. Third, comprehensive outreach care (COC) integrates drug refills into existing mobile clinic outreach activities for geographically isolated communities. We described baseline factors at enrolment, and 6 month retention in community care models and proportion of patients transferred back to routine clinical care.

**Results:**

On average, 47 patients enrolled into community-ART care each month: 51.1% into TC (242 patients in eight groups), 34.0% in CAG (164 patients in 38 groups) and 14.9% in COC (65 patients in two remote communities). All patients had a VL<1000 copies/ml, the median CD4 was 512 (TC), 528 (CAG) and 657 (COC) cells/µl (p=0.27), the median age was 40, 40 and 45 years (p=0.11), and 74.8, 66.5 and 64.6% were females (p=0.03). Retention in care after 6 months was highest in TC (97.5%) when compared to CAG (79.2%) and COC (78.4%) (p<0.01). In total, 53/471 patients (11.3%) returned back to and were retained in routine clinic care, and one (0.21%) was recorded as death in COC.

**Conclusions:**

Concurrent implementation of three community ART care models was feasible. Although a proportion of patients returned back to clinic care, overall ART retention was high and should encourage programme managers to apply differentiated care models adapted to their specific setting.

# Sub article: FRAE0205 Provision of streamlined HIV care associated with reduced economic burden of care-seeking among HIV-infected adults

**Published:** 2016-07-22

**Authors:** A Jakubowski, J Kabami, D Mwai, K Snyman, T Clark, J Ayieko, A Owaraganise, F Mwangwa, M Petersen, C Cohen, E Bukusi, M Kamya, D Havlir, E Charlebois, H Thirumurthy

**Affiliations:** 1 Department of Health Policy and Management, University of North Carolina at Chapel Hill, Chapel Hill, NC, United States; 2 Infectious Diseases Research Collaboration, Mbarara, Uganda; 3 University of Nairobi, Nairobi, Kenya; 4 University of California, San Francisco, CA, United States; 5 Kenya Medical Research Institute, Nairobi, Kenya; 6 Infectious Diseases Research Collaboration, Kampala, Uganda; 7 University of California, Berkeley, CA, United States; 8 Makerere University College of Health Sciences, Kampala, Uganda

## Abstract

**Introduction:**

HIV-infected adults and their households often face a large economic burden stemming from out-of-pocket health expenditures, clinic transportation costs and lost from work or usual household activities. In Kenyan and Ugandan communities that began receiving streamlined HIV care (appointment reminders, quarterly visits with patient-centred care providing reduced waiting and overall visit duration) as part of the SEARCH test-and-treat trial, we examined changes in costs incurred by HIV-infected adults over a 1 year period.

**Methods:**

Data were obtained through household surveys administered to a random sample of HIV-infected adults in 32 communities participating in the SEARCH trial (NCT01864603). In the 16 SEARCH intervention communities employing streamlined HIV care, we compared out-of-pocket costs (in US$) and time costs incurred by HIV-infected patients receiving antiretroviral therapy (ART) under standard HIV care at baseline (n=1230) to costs incurred by a larger sample of patients receiving ART, including those with high CD4 cell counts, under streamlined HIV care 1 year later (n=1589). We also examined changes in these costs separately in the three regions of Kenya and Uganda where the SEARCH trial is occurring. Comparison of means was performed using two-sided t-tests.

**Results:**

Patients receiving ART under streamlined care spent less than half the time seeking and receiving healthcare than adults receiving ART under standard care (4.40 hours per month at baseline vs. 1.78 hours per month at follow-up, p<0.001). Time spent away from employment or usual activities was also significantly reduced, from 13.0 hours per month at baseline to 8.17 hours at follow-up (p<0.01). The reductions in time costs were largest in SEARCH intervention communities in Uganda compared to Kenya. Out-of-pocket healthcare and transportation costs incurred by patients did not differ significantly between baseline and 1 year later ($2.98 and $2.46 in past month at baseline and 1 year, respectively).

**Conclusions:**

Following the introduction of streamlined care for HIV-infected individuals, there was a significant reduction in time spent seeking healthcare and being away from employment and other usual activities. Streamlined care provision may partially reduce the economic burden faced by individuals receiving HIV care and contribute to improvements in patients' employment outcomes and economic well-being.

# Sub article: FRAE0206LB Discontinuation from community-based antiretroviral adherence clubs in Gugulethu, Cape Town, South Africa

**Published:** 2016-07-22

**Authors:** A Nofemela, C Kalombo, C Orrell, L Myer

**Affiliations:** 1 Department of Epidemiology and Biostatistics, University of Cape Town, Cape Town, South Africa; 2 Centre for Infectious Diseases Epidemiology & Research, University of Cape Town, Cape Town, South Africa; 3 Department of Health, Western Cape Government, Cape Town, South Africa; 4 Desmond Tutu HIV Foundation, Cape Town, South Africa

## Abstract

**Introduction:**

Community-based adherence clubs are an attractive model of care for the growing number of stable patients on antiretroviral therapy (ART) in high-burden settings, but little is known about the reasons why patients exit these clubs over time.

**Methods:**

We used routinely-collected club data linked to a large primary healthcare (PHC) clinic. These clubs enrol stable ART patients (>6–12 months on ART with viral suppression and CD4 >200) into a lay-counsellor-led programme of 2–4 monthly ART collection, with adherence support, at a community venue. Viral load (VL) monitoring is conducted annually. In analysis, we examined reasons for discontinuation from clubs over time, while proportional hazards models were used to examine risk factors for lost to follow-up (LTF), defined as >6 months without a club visit before the end of November 2015 without an alternate outcome.

**Results:**

Between June 2012 and October 2015, 3359 patients entered a club (median age, 37 years; 71% female; median duration of ART use, 3.5 years). 4% of all club visits resulted in a referral of patients by counsellors back to the PHC for review by a nurse or doctor; these were usually due to a clinical comorbidity (primarily TB, diabetes or hypertension), defaulting ART, or elevated VL. Rates of death, transfer out, and LTF from the clubs were 0.3, 0.9, and 9.4 per 100 person-years in the clubs, respectively. After three years of club operations, 26% of patients were LTF; independent of gender, LTF was increased in patients <25 years of age (hazard ratio, 1.7; 95% CI: 1.2–2.5). In the subset of patients who had VL monitoring, prior raised VL in the clubs was strongly predictive of subsequent LTF (HR, 4.4; 95% CI: 2.9–6.7). There was no association between time on ART before entry into clubs and either referral back to clinic or LTF (p = 0.921).

**Conclusions:**

Referrals of stable ART patients from counsellor-led, community-based adherence clubs back to PHC are an important feature of community-based care, and expanding this model of care to include common co-morbidities may reduce these referrals. While the majority of patients are retained effectively in clubs, LTF is an ongoing concern.

# Sub article: TUPDA0101 Selection of HIV-1 variants with higher transmission potential by 1% tenofovir gel microbicide

**Published:** 2016-07-22

**Authors:** N Ngandu, J Carlson, D Chopera, N Ndabambi, S Goodier, N Garrett, N Samsunder, Q Abdool Karim, S Abdool Karim, C Williamson

**Affiliations:** 1 Pathology, University of Cape Town, Cape Town, South Africa; 2 Health Systems Research Unit, South African Medical Research Council, Cape Town, South Africa; 3 Microsoft Research, Los Angeles, CA, United States; 4 Centre for the AIDS Program of Research in South Africa, University of KwaZulu Natal, Durban, South Africa

## Abstract

**Introduction:**

Women in the CAPRISA 004 trial assigned to use 1% tenofovir microbicide gel had higher HIV-1 viral loads and slower antibody avidity maturation compared to placebo participants. This study sought to determine whether tenofovir gel selected for viruses with altered characteristics which, in turn, influenced disease progression.

**Methods:**

We analyzed bulk *gag* sequences from the earliest time-point post-infection for 71 (n=28 TFV and n=43 placebo) participants who became infected during the CAPRISA 004 microbicide trial conducted in Durban, South Africa. The median (IQR) days post infection at which the sequence data were obtained were 84 (84–91) for women assigned to tenofovir gel and 84 (77–84) for women assigned to placebo. Genetic distances between sequences were estimated in MEGA version 5.1 [1]. The transmission index of a sequence was calculated as the mean of the expected log-odds of transmission for each site in the sequence, as estimated by a logistic regression model that included a second-order polynomial of cohort frequency, the number of co-varying sites, and offsets and cohort-frequency interactions for each protein domain. Transmission indices were computed out-of-sample using leave-one-out cross-validation.

**Results:**

Sequences from the two groups (tenofovir and placebo) of the trial were interspersed on the phylogenetic tree, showing no lineage effects on the viruses infecting the two groups. Viruses within the tenofovir group were less diverse from each other compared to those from the placebo group (p<0.0001), suggesting constrained diversity of viruses infecting the tenofovir group. Furthermore, viruses from the tenofovir group were closer to the consensus sequence of regional strains (p=0.003), and had higher transmission index (p=0.01), than those from the placebo group. There was a modest correlation between the transmission index and the baseline viral load (Spearman r=0.2, p=0.04) but not with viral load at set-point (12 months post-infection).

**Conclusions:**

The 1% tenofovir gel may have increased the transmission barrier to select for more consensus-like viral variants with a higher transmission index.

## Reference

1. Tamura K, Peterson D, Peterson N, Stecher G, Nei M and Kumar S. MEGA5: molecular evolutionary genetics analysis using maximum likelihood, evolutionary distance, and maximum parsimony methods. Mol Biol Evol. 2011 Oct;28(10):2731–9.

# Sub article: TUPDA0102 Characterization of HIV-1 genomes from 74 acutely infected subjects in the acute infection cohort RV217

**Published:** 2016-07-22

**Authors:** M Rolland, S Tovanabutra, G Kijak, E Sanders-Buell, M Bose, C Owen, L Maganga, S Nitayaphan, K Rono, A Sekiziyivu, La Eller, J Kim, N Michael, M Robb

**Affiliations:** 1 Military HIV Research Program, Silver Spring, MD, United States; 2 MMRP, Mbeya, United Republic of Tanzania; 3 AFRIMS, Bangkok, Thailand; 4 WRP-Kericho, Kericho, Kenya; 5 MUWRP, Kampala, Uganda; 6 MHRP, Silver Spring, MD, United States

## Abstract

**Introduction:**

Since the early events of HIV infection are critical for disease progression and provide a path to develop anti-HIV strategies, we analyzed viral evolution during the first 6 months of HIV infection in 74 treatment-naive individuals enrolled in the RV217 acute infection cohort in East Africa and Thailand.

**Methods:**

More than 2000 HIV-1 negative individuals were enrolled and bi-weekly testing for HIV-1 RNA allowed to identify subjects during the earliest days of infection in Kenya (n=9), Tanzania (n=18), Uganda (n=18) and Thailand (n=29). HIV-1 genomes were sequenced following PCR amplification by endpoint-dilution from plasma samples.

**Results:**

We sequenced 802 HIV-1 genomes from 74 subjects at a median of 4 days after HIV-1 diagnosis, a diagnosis that occurred a median of 6 days after the last negative visit. For a subset of 42 individuals, we sequenced 857 additional genomes obtained at a median of 32 and 171 days post HIV diagnosis. Sequences from the first time point were obtained before peak viremia, which occurred 12 days after diagnosis with viral loads reaching 6.55 log_10_ copies/ml (range: 3.96–8.46 log_10_ copies/ml). The median viral load setpoint (SPVL) was 4.31 log_10_ copies/ml (range: 2.43–5.96 log_10_ copies/ml). In East Africa, most individuals (n=25) were infected with circulating recombinant forms (CRF) comprising subtype A1, C and D with 13 individuals infected with subtype A1 and seven with subtype C; in Thailand, 23 of 29 subjects were infected with CRF01_AE.

We identified multiple HIV-1 variants among sequences from 18 of 48 subjects, with some variants found at very low level initially and some variants being identified only temporarily. SPVL for subjects replicating multiple founders was significantly higher than for subjects with single founders (4.82 vs. 4.08 log_10_ copies/ml, p=0.021).

**Conclusions:**

Analysis of HIV-1 sequences earlier in infection and more frequently than before revealed a greater complexity in HIV-1 evolutionary processes and identified viral determinants associated with higher viral loads. Identifying HIV-1 features that determine SPVL will allow to better predict the transmission risk associated with specific HIV-1 variants.

# Sub article: TUPDA0103 HIV-associated alteration in gut microbiota are associated with increased inflammation and infection of enteric CD4+ T cells

**Published:** 2016-07-22

**Authors:** B Palmer, C Neff, S Li, J Schneider, T Campbell, C Lozupone

**Affiliations:** Medicine, University of Colorado, Denver, Aurora, CO, United States

## Abstract

**Introduction:**

HIV infection is associated with dramatic alterations of the enteric microbiome that often persist despite long-term otherwise successful Antiretroviral Therapy (ART). Alterations in gut microbiota have been correlated with HIV disease progression, inflammatory markers in the gut and with inflammatory and bacterial translocation markers in blood. A better understanding of the immune-modulatory properties in gut microbes that correlate with disease will allow for the exploration of microbial drivers of immune activation, HIV disease pathogenesis and co-morbidity.

**Methods:**

We are collecting gut microbiome data from a large cohort of individuals living in Colorado and identifying bacteria whose prevalence correlates with disease status, ART, and with inflammatory and metabolic disease markers. To explore the immune-modulatory properties of these bacteria, we culture peripheral blood mononuclear cells (PBMC) and lamina propria mononuclear cells (LPMC) isolated from resected gut tissue with bacteria isolated from patient stool and cultured bacteria that are increased or decreased with HIV infection. We then measure the impact of the stimulation on pro- and anti-inflammatory cytokines, T cell activation, T regulatory cells, HIV co-receptors and levels of HIV infection.

**Results:**

Many bacterial species significantly change with HIV and correlate with inflammatory and translocation markers in blood in our study population. Although stimulations of PBMC/LPMC with most bacteria induce both pro- and anti-inflammatory cytokines, cultured bacteria that increase with HIV and faecal bacteria from HIV-infected individuals induce lower levels of T regulatory cells and anti-inflammatory IL-10. Furthermore, incubation of LPMCs with numerically dominant bacteria of the HIV-associated (*Prevotella copri*) but not health-associated (*Bacteroides uniformis*) gut microbiome resulted in increased infectivity of immune cells by HIV.

**Conclusions:**

These data suggest that a loss of anti-inflammatory (beneficial) bacteria with HIV infection has the potential to drive chronic inflammation observed in HIV-infected individuals. Furthermore, our infectivity assays indicate a potential role of the HIV-associated gut microbiome in disease transmission and progression. We are currently further exploring microbiome associations with HIV disease, inflammation and metabolic disease in populations in both the US and Zimbabwe.

# Sub article: TUPDA0104 The association of injectable progestin-only contraceptives and endogenous progestins with HIV target cell frequency in the cervix and HIV acquisition risk

**Published:** 2016-07-22

**Authors:** EH Byrne, MN Anahtar, KE Cohen, A Moodeley, N Padavattan, N Ismail, BA Bowman, GS Olson, A Mabhula, A Leslie, T Ndung'u, BD Walker, MS Ghebremichael, KL Dong, DS Kwon

**Affiliations:** 1 Ragon Institute of MGH, MIT and Harvard, Cambridge, MA, United States; 2 Females Rising through Education, Support and Health, Durban, South Africa; 3 HIV Pathogenesis Programme, Durban, South Africa; 4 KwaZulu Natal Research Institute for Tuberculosis and HIV, Durban, South Africa; 5 Division of Infectious Diseases, Massachusetts General Hospital, Boston, MA, United States; 6 Howard Hughes Medical Institute, Chevy Chase, MD, United States

## Abstract

**Introduction:**

Multiple observational studies have suggested that injectable progestin-only contraceptives (IPCs) are associated with HIV acquisition risk. However, the biological mechanism of this potential link was unclear. We aimed to understand immunological changes associated with exogenous and endogenous progestins that could mechanistically help explain a link to acquisition risk.

**Methods:**

HIV-negative South African women ages 18–23 were enrolled in a prospective cohort study, the Females Rising through Education, Support and Health (FRESH) study. These women were at high risk of acquiring HIV, were living in Umlazi and were not pregnant. During the study, they were tested for HIV-1 two times per week; behavioural data along with blood and cervical samples were collected every 3 months.

**Results:**

We characterized 423 HIV-uninfected women from the FRESH cohort. Of these, 152 women used IPCs, 222 used no long-term contraceptive and 43 used other forms of contraception. IPC users had a higher risk of acquiring HIV (12.06 per 100 person-years, 95% CI 6.41–20.63) compared to women using no long-term contraceptive (3.71 per 100 person-years, 1.36–8.07; adjusted hazard ratio 2.93, 95% CI 1.09–7.868, p=0.0326). In the cervix, CCR5+CD4 T cells (HIV target cells) were 3.92 times more prevalent in IPC users than in women using no long-term contraceptive (p=0.0241). Of women using no long-term contraceptive, those in the luteal phase of the menstrual cycle had 3.25 times the frequency of cervical target cells compared with those in the follicular phase (p=0.0488).

**Conclusions:**

High progestin levels, either due to the use of IPCs or the luteal phase of the menstrual cycle, are associated with an increased frequency of HIV target cells in the cervix compared to women with low progestin levels, in the follicular phase of the menstrual cycle. Because the female genital tract is the site of HIV entry in most women who become infected, the higher density of HIV target cells in a high-progestin state provides a potential biological mechanism for the epidemiological observation of increased HIV acquisition risk in IPC users.

# Sub article: TUPDA0105 The mechanisms and role of HPV in enhancing HIV transmission in women in South Africa

**Published:** 2016-07-22

**Authors:** L Liebenberg, LR McKinnon, K Leask, A Rositch, N Garrett, N Samsunder, A Kharsany, A Grobler, A Singh, J-A Passmore, SS Abdool Karim, Q Abdool Karim

**Affiliations:** 1 Centre for the AIDS Programme of Research in South Africa (CAPRISA), Durban, South Africa; 2 Department of Medical Microbiology, University of KwaZulu Natal, Durban, South Africa; 3 Department of Medical Microbiology, University of Manitoba, Winnipeg, Canada; 4 Johns Hopkins Bloomberg School of Public Health, Baltimore, MD, United States; 5 University of Cape Town, Cape Town, South Africa; 6 National Health Laboratory Service, Johannesburg, South Africa; 7 Department of Epidemiology, Columbia University, New York, NY, United States

## Abstract

**Introduction:**

Young women bear a disproportionately high burden of sexually-acquired HIV infection. Human papillomavirus (HPV), a common sexually transmitted infection is a known contributor to this burden through its established association with higher rates of HIV acquisition. However, the mechanism of this relationship remains unclear. Here we explored whether the immunological impact of HPV promotes a mucosal immune environment that favours the establishment of HIV infection in young women in KwaZulu-Natal, South Africa.

**Methods:**

This cohort study was nested within the CAPRISA 004 1% tenofovir gel study. Stored genital specimens from HIV uninfected participants (N=779) were utilized to determine the presence of 37 HPV genotypes using commercially available Linear Array kits. Concentrations of 48 cytokines were quantified by multiplexed ELISA assays, and the presence of CD4+ targets for HIV infection was investigated by flow cytometry. HIV infection was monitored monthly using two commercially available rapid tests and confirmed by western blot and PCR.

**Results:**

Baseline HPV prevalence was 73.8% (95% CI: 70.7, 76.9), with 70.3% of these infected participants presenting with an oncogenic strain. Participants with prevalent HPV infection were 2.8 times more likely to acquire HIV infection compared to those without HPV infection (HR 2.8, 95% CI: 1.3, 5.9, p=0.006). HIV risk was independent of the oncogenicity of HPV strains at baseline (HPV oncogenic strains HR 2.9 (95% CI: 1.3, 6.1) vs. non-oncogenic strains HR 2.8 (95% CI: 1.3, 6.1)), and was also increased in the presence of multiple concurrent infections (HR 4.0; 95% CI: 1.8, 8.8). Compared to HPV uninfected women, acquisition, clearance, or persistence of HPV were each significantly associated with >6-fold increased rates of HIV acquisition, and elevated concentrations of several cytokines associated with HIV infection (including IL-8, MIP-1a, RANTES, IL-1a, IL-6). Further, in line with cytokine involvement in chemotaxis, the influx of CD4+ T cell targets for HIV infection was associated with HPV infection (p=0.012).

**Conclusions:**

These data provide a plausible causal immunological link between two viral infections of critical public health importance and suggest that increased HPV vaccination rates in young women could have important additional HIV prevention benefits.

# Sub article: TUPDA0106 The endotoxin-lipoprotein hypothesis, obesity and HIV

**Published:** 2016-07-22

**Authors:** S Martinez, A Campa, F Huffman, J Makhema, S Moyo, OD Williams, R Marlink, M Baum

**Affiliations:** 1 Florida International University, Miami, FL, United States; 2 Botswana Harvard AIDS Institute Partnership, Gaborone, Botswana; 3 Harvard School of Public Health, Boston, MA, United States

## Abstract

**Introduction:**

An obesity paradox has been documented in many conditions including HIV infection, where those who are obese may have a survival advantage or improved disease outcomes. Lipopolysaccharide (LPS), an endotoxin, is a constituent of Gram-negative bacterial cell walls and known to produce proinflammatory responses. Sequestration of LPS by higher circulating lipoproteins in obesity has been suggested as mechanism for the obesity paradox known as the endotoxin-lipoprotein hypothesis and warrants further investigation in HIV.

**Methods:**

A retrospective cross-sectional analysis of data and specimens from a nutritional study was conducted in 60 HIV+ ART-naïve adults who were in the early stages of HIV disease in Botswana, Africa. Anthropometrics and bioimpedance were obtained. Blood was drawn for LPS, total cholesterol as a measure of circulating lipoproteins, CD4 count, and HIV viral load. Regression analyses were adjusted for age, gender and smoking.

**Results:**

Among 60 ARV-naïve HIV+ asymptomatic adults, the median age was 33 years (IQR: 29–39) and 76.4% were women. The overweight/obese group had higher total cholesterol levels (4.08±0.85 vs. 3.50±0.65 mmol/l, p=0.007) than the normal weight group. Plasma LPS levels at or above the median (0.058 EU/ml) were associated with lower BMI (OR=0.79; 95% CI: 0.630, 0.990; p=0.041), lower fat mass % (OR=0.852, 95% CI: 0.757, 0.958, p=0.007), and higher HIV viral load (OR=2.608, 95% CI: 1.111, 6.124; p=0.028). Higher LPS levels were also associated with a lower odds of overweight/obesity, BMI≥25 kg/m^2^ (OR=0.035, 95% CI: 0.004, 0.283; p=0.002). LPS levels at or above the median were also associated with lower total cholesterol levels (OR=0.360, 95% CI: 0.150–0.862; p=0.022), controlling for age, gender, smoking, and HIV viral load.

**Conclusions:**

A possible explanation for the lower levels of LPS observed in those with higher BMI might be similar to the endotoxin lipoprotein hypothesis first described by [1] to explain the endotoxin lipoprotein paradox in congestive heart failure. Higher circulating lipoproteins may reduce inflammation by binding to lipopolysaccharides and decreasing the release of proinflammatory cytokines. In HIV infection, viral load contributes to increase LPS.

## Reference

1. Rauchhaus M, Coats AJ, Anker SD. The endotoxin-lipoprotein hypothesis. Lancet 2000;356:930–933.

# Sub article: TUPDB0101 Assessment of the World Health Organization early warning indicators of HIV drug resistance in Namibia for public health action, 2015

**Published:** 2016-07-22

**Authors:** N Mutenda, T Nakanyala, N Hamunime, T Mekonen, F Tjituka, S Natanael, G Mazibuko, S Mwinga, D Mabirizi, E Sagwa, H Walkowiak, A Kiesling, S Aptekar, M Jordan, S Hong

**Affiliations:** 1 Directorate of Special Programmes, Ministry of Health and Social Services, Windhoek, Namibia; 2 Systems for Improved Access to Pharmaceuticals and Services Program, Management Sciences for Health, Windhoek, Namibia; 3 Systems for Improved Access to Pharmaceuticals and Services Program, Management Sciences for Health, Arlington, VA, United States; 4 Management Sciences for Health, Systems for Improved Access to Pharmaceuticals and Services Program, New York, NY, United States; 5 Department of Public Health and Community Medicine, Tufts University School of Medicine, Boston, MA, United States; 6 Division of Geographic Medicine and Infectious Diseases, Tufts Medical Center, Boston, MA, United States

## Abstract

**Introduction:**

Early warning indicators (EWIs) of HIV drug resistance (HIVDR) are a key element of the World Health Organization (WHO) public health strategy to minimize and monitor emergence of HIVDR at facilities providing antiretroviral treatment (ART) in countries that are rapidly scaling up treatment. Namibia has instituted a routine EWI monitoring system and developed HIVDR survey strategies.

**Methods:**

In 2015, we abstracted the following WHO EWIs from adult and paediatric patients from all ART sites in the state health sector. These included 50 main ART sites and 163 Integrated Management of Adolescent and Adult Illness (IMAI) sites and outreach points): *EWI 1: On-time Pill Pick-up, EWI 2: Retention in Care at 12 months, EWI 3: Pharmacy Stock-outs, EWI 4: Dispensing Practices*, and *EWI 5: Viral Suppression at 12 months*.

**Results:**

All 213 ART sites in Namibia were included. For *EWI 1*, 43% of sites achieved either excellent or fair performance (>90% or 80–90% of patients on-time collection) for adults and 40% for children. For *EWI 2*, 53% of sites achieved either excellent or fair performance (>85% or 75–85% retention) for adults and 37% for children. For *EWI 3*, 5% of sites achieved excellent performance (100% of months with no stock-outs) for adult patients and 14% for children. For *EWI 4*, 97% of sites achieved excellent performance (0% mono- or dual-therapy) in adults and 91% for children. For *EWI 5*, low rates of viral load (VL) completion among patients eligible for routine VL testing significantly affected monitoring of viral suppression.

**Conclusions:**

Namibia has successfully institutionalized EWI monitoring into routine ART programme functioning. Strengthening patient adherence to treatment, retention in care, and ensuring the continuous availability of antiretroviral medicines are all high priorities to minimize emergence of HIVDR and achieve the 90-90-90 (HIV epidemic control) goals. Additionally, improving routine VL monitoring and data capturing is a priority to enable monitoring of viral suppression rates. As a result of these data, programme leaders and healthcare providers in regions throughout the country are implementing service quality improvement projects and operational research to improve patient care and minimize the emergence of HIVDR.

# Sub article: TUPDB0102 High prevalence of antiretroviral drug resistance among HIV-infected pregnant women in Buenos Aires, Argentina

**Published:** 2016-07-22

**Authors:** I Zapiola, DM Cecchini, S Fernandez Giuliano, M Martinez, CG Rodriguez, MB Bouzas

**Affiliations:** 1 Virology Unit, Hospital Muñiz, Buenos Aires, Argentina; 2 Working Group in Prevention of Mother-to-Child Transmission, Hospital Cosme Argerich, Buenos Aires, Argentina

## Abstract

**Introduction:**

The presence of primary mutations in the viral genome is a major cause of drug resistance, which can lead to treatment failure. Thus, monitoring the presence of drug resistance-associated mutations (RAMs) in HIV-infected pregnant women (HPW) is crucial for optimizing antiretroviral therapy (ART) selection. Until recently, genotypic resistance tests were not routinely available for HPW in Argentina and information about the prevalence of RAMs in this population is limited.

**Objective:**

To determine trends in the prevalence of RAMs in HPW assisted in a public hospital in Buenos Aires, Argentina.

**Methods:**

HPW were recruited as part of a prospective sentinel epidemiological survey (period 2008–2014). Baseline plasma samples were sequenced using TRUGENETM HIV-1 Genotyping Kit. RAMs were identified in ART-naive and ART-experienced patients, according to the WHO guidelines and the IAS-USA mutation list, respectively. RAMs prevalence was compared for two periods: 2008–2011 versus 2012–2014.

**Results:**

Overall, 136 HPW were included: 77 (56.6%) naïve and 59 (43.4%) ART-experienced (24 with ongoing ART and 35 with a history of exposure to ART). A total of 37 (27.2%) women had at least one RAM: 25/94 (26.5%) in 2008–2011 and 12/42 (28.5%) in 2012–2014 (p>0.05). Among the naïve, 15 (19.5%) had at least one RAM: 10/49 (20.4%) in period 2008–2011 and 5/28 (17.8%) in 2012–2014 (p>0.05). Transmitted resistance was observed mainly for non-nucleoside reverse transcriptase inhibitors (NNRTIs): 14.3% in 2008–2011 and 17.8% in 2012–2014, being K103N the most common mutation. Among the ART-experienced HPW, 37.3% had RAMs: 33.3% in 2008–2011 and 50% in 2012–2014 (p>0.05). In the experienced HPW with ongoing ART subgroup, 50% had nucleoside reverse transcriptase inhibitors-RAMs, and 45.8% had NNRTI-RAMs. In the experienced group with a history of (but not ongoing) ART-exposure, 17.1% had NNRTI-RAMs.

**Conclusions:**

This sentinel study demonstrates an overall high prevalence of RAMs in HPW in Buenos Aires city, which remained stable over the two periods analyzed. Considering the>15% prevalence found in naïve HPW is above the threshold suggested by WHO for routine resistance surveillance in a certain population, access to genotypic tests should be warranted.

# Sub article: TUPDB0103 HIV-1 drug resistance and genetic diversity in ART-naïve patients infected in 2013-2015 in Kazakhstan

**Published:** 2016-07-22

**Authors:** N Dzissyuk, A Abishev, A Zhanpeisova, G Nagashbekova, G Tazhibayeva

**Affiliations:** Republican Center for Prevention and Control of AIDS, Almaty, Kazakhstan

## Abstract

**Introduction:**

Treatment of HIV-positive patients in Kazakhstan was launched 10 years ago. Every year there is an increasing number of cases of HIV-1 drug resistance among patients with failing first-line antiretroviral therapy (ART). The aim of the study was to analyze prevalence of HIV drug resistance in ART-naïve patients infected in 2013–2015 in Kazakhstan.

**Methods:**

A total of 376 plasma samples from newly HIV-infected patients from eight regions of Kazakhstan were tested. Isolation of HIV RNA, RT-PCR, sequencing of *pro* (1–99) and *rev* (35–265) genes were performed using diagnostic kit “AmpliSens-HIV-Resist-Seq.” For interpretation of HIV drug resistance, the software “Deona” was used. HIV subtypes were determined using the programme Comet HIV-1 (http://comet.retrovirology.lu). The level of transmitted HIV drug resistance was determined by the software CPR (http://cpr.stanford.edu/cpr.cgi).

**Results:**

In the study group, the patients were 56.6% males; median age: 35.7 years; median CD4: 478/mm^3^; risk factors: heterosexuals (64.7%), MSM (3.0%), IDUs (31.4%), unknown (0.9%).

A total of 56.1% were infected with subtype of HIV-1 A1 (IDU-A variant); 38.6% – CRF02_AG; 2.9% – subtype B; 2.4% – other (CRF03_AB, CRF07_BC and URF). CRF02_AG is more prevalent in the southern regions of Kazakhstan (70.4–85.0%), while in the central and eastern regions of the country dominated HIV-1 subtype A1 (55.1–96.2%).

The presence of mutations of HIV-1 resistance to first-line antiviral drugs among ART-naïve patients has been identified in six regions of Kazakhstan (from 1.9 to 4.2%). Three patients (0.8%) were identified TAMs to NRTIs: M41L, L210W and K219E/R. Four patients (1.1%) were identified resistance mutations to NNRTI: K101E, K103N, Y181C.

**Conclusions:**

The study showed that in Kazakhstan the level of primary HIV-1 drug resistance to NRTI/NNRTI is low. The highest level (4.2%) of primary HIV-1 drug resistance observed in large cities, where there is the greatest number of patients on ART.

# Sub article: TUPDB0104 Cross-sectional assessment of virological failure, drug resistance and third-line regimen requirements among patients receiving second-line ART in 3 large HIV programmes in Kenya, Malawi and Mozambique

**Published:** 2016-07-22

**Authors:** B Schramm, V Carnimeo, A Rakesh, DL Ardiet, L Cossa, O Bouchaud, C Alloui, WL Oo, P Gonzales Dias, I Mukui, W Omwoyo, R Manuel, AM de Pedro, A Telnov, Z Chirwa, B Chilima, S Nicholas, A Vubil, L Serrano, V Opolo, C Zeh, M Peeters, L Molfino, L Salumu, A Vandenbulcke, I Amoros, J-F Etard, M Pujades Rodríguez, S Balkan, E Szumilin

**Affiliations:** 1 Epicentre, Paris, France; 2 Médecins Sans Frontières, Chiradzulu, Malawi; 3 Clinical Research Department, Epicentre, Paris, France; 4 Médecins Sans Frontières, Maputo, Mozambique; 5 CHU Avicenne, Assistance Publique-Hôpitaux de Paris et Université, Service des Maladies Infectieuses et Tropicales, Paris, France; 6 Médecins Sans Frontières, Homa Bay, Kenya; 7 National AIDS and STIs Control Programme, Nairobi, Kenya; 8 Ministry of Health, Homa Bay, Kenya; 9 National HIV/AIDS Program, Ministry of Health, Maputo, Mozambique; 10 Ministry of Health, Maputo, Kenya; 11 Médecins Sans Frontières, Geneva, Switzerland; 12 Treatment and Care, HIV and AIDS Department, Ministry of Health, Lilongwe, Malawi; 13 Preventative Health Services, Ministry of Health, Lilongwe, Malawi; 14 National Health Institut, Maputo, Mozambique; 15 UMI 233 Institut de Recherche pour le Développement, University of Montpellier, Montpellier, France; 16 Centers for Disease Control and Prevention, Kenya Medical Research Institute, Kisumu, Kenya; 17 Médecins Sans Frontières, Paris, France; 18 Médecins Sans Frontières, Nairobi, Kenya; 19 Médecins Sans Frontières, Lilongwe, Malawi; 20 University of Leeds, Leeds, United Kingdom

## Abstract

**Introduction:**

With access to viral load (VL) monitoring, the number of patients receiving second-line antiretroviral treatment (ART) is increasing in resource-limited countries. We assessed virological response and second-line drug resistance in three large HIV-programmes to inform regimen-requirements, to evaluate patient outcomes and support forecasting of effective third-line drugs.

**Methods:**

Between November 2014 and December 2015, patients aged≥5 years receiving a standard second-line regimen for≥6 months were recruited in three HIV outpatient-clinics supported by Médecins Sans Frontières in Kenya, Malawi and Mozambique. VL was quantified and resistance-genotyping performed if VL≥500 HIV RNA copies/ml (virological failure). Sequences were interpreted with Stanford and ANRS algorithms. Virological failures are assessed 6 and 12 months after coun**s**elling or regimen change.

**Results:**

A total of 824 patients were included (median age 41 years, 45.4% males). In Kenya, among 355 participants (26.9 month median duration of second-line; 71.6% 3TC-TDF-LPV/r), 18.3% (65/355) had VL≥500 copies/ml, 16.9%≥1000 copies/ml. Among those aged≥19 years, 31.2% (20/64) had≥500 copies/ml. Overall 24.6% (16/65) had major PI-resistance, 72.3% major NRTI-resistance, 80% major NNRTI-resistance, and 9.2% major etravirine-resistance (Stanford). Nineteen patients (29.2%) required replacement of ineffective NRTIs, 21 (32.3%) needed to start a third line regimen (change of PI-component), with three children requiring paediatric formulations. Six months after regimen change 77.8% (14/18) had VL <20 copies/ml. In Malawi: among 242 patients (36.3 month median duration of second-line; 81.4% 3TC-TDF-ATV/r), 16.5% had VL≤500 copies/ml, 13.2%≤1000. Among those aged≤19 years, 29.4% (10/34) had VL≤500. Sequencing (37/40) detected 2.9% major PI-resistance, 78.4% major NRTI-resistance, 83.8% major NNRTI-resistance, 18.9% major etravirine-resistance. Seven patients required switch to a third-line regimen, 12 required NRTI-replacement. Complete resistance and regimen data will be available from all sites, including Mozambique (227 patients, 91.2% TDF-3TC-LPV/r).

**Conclusions:**

These findings indicate good virological suppression in patients receiving second-line ART. Failure rates were notably higher among children and adolescents, highlighting the need for enhanced monitoring. Resistance data were essential to inform optimal regimen choice. Preliminary results indicate good short-term outcomes of patients who needed ART change. Increased access to resistance genotyping and affordable salvage ARVs, including paediatric formulations, are needed.

# Sub article: TUPDB0105 Effect of PI resistance mutations on viral load in patients on PI monotherapy

**Published:** 2016-07-22

**Authors:** JA Thompson, C Kityo, AS Walker, J Hakim, A Kambugu, JJ van Oosterhout, A Siika, A Mweemba, P Van den Eede, DT Dunn, NI Paton, for the Earnest Trial Team

**Affiliations:** 1 MRC Clinical Trials Unit, University College London, London, United Kingdom; 2 Joint Clinical Research Centre, Kampala, Uganda; 3 University of Zimbabwe, Clinical Research Centre, Harare, Zimbabwe; 4 Infectious Disease Institute, Kampala, Uganda; 5 University of Malawi, Blantyre, Malawi; 6 Moi University, Clinical Research Centre, Eldoret, Kenya; 7 University Teaching Hospital, Lusaka, Zambia; 8 Janssen, Beerse, Belgium

## Abstract

**Introduction:**

Protease inhibitor (PI) resistance mutations are uncommon in patients failing boosted-PI (bPI) containing regimens; longitudinal data assessing impact of PI resistance on outcomes are sparse.

**Methods:**

We assessed the development of PI-resistance mutations over time in patients failing first-line NNRTI-based regimens and randomized within the EARNEST trial to bPI-monotherapy (standardized to lopinavir/ritonavir bd, with 12-week raltegravir induction to induce rapid VL suppression). VLs and resistance tests were performed blinded. Resistance testing was done retrospectively in one laboratory on all stored samples (12–16 weekly) between first confirmed virological failure (VL >1000 copies/ml) and switch to combination therapy.

**Results:**

A total of 405 patients started bPI-monotherapy and had ≥1 follow-up VL sample. Median treatment duration was 108 (IQR: 98–124) weeks until switch to combination therapy following an interim review. One hundred forty-eight (37%) developed virological failure on bPI-monotherapy. Median VL at bPI-monotherapy failure was 3681 c/ml, subsequently increasing by 0.48log_10_ c/ml per year (95% CI: 0.31–0.65). 28(26%) of the 106 with genotypes at bPI-monotherapy failure had major/minor PI mutations, increasing to five (62%) of the eight with genotypes 96 weeks after failure. The most common mutations were V82A (39%), I54V (39%) and M46I (32%). Rate of emergence of new mutations peaked at 37 weeks after failure (1.81 mutations/year; 95% CI: 1.31–2.51; see Figure 1). In a multivariable model, each new mutation was associated with a mean increase of 0.12log_10_ c/ml (95% CI: 0.06–0.18, p<0.001). Q58E and I47A were associated with significantly (p<0.02) larger increases of 0.74 (95% CI: 0.24–1.20) and 1.02 (95% CI: 0.47–1.56) log10 c/ml, respectively. I47A slowed the increase in VL by 0.43log_10_ c/ml per year (95% CI: −0.01 to 0.87, p=0.05).

**Conclusions:**

Overall, the rate of accumulation of PI resistance mutations was slow in patients with virological failure on bPI monotherapy with lopinavir/ritonavir; declines after 37 weeks of failure suggest fitness costs may prevent additional mutations developing. The impact of resistance on VL is also limited, although I47A/Q58E appears to have greater effects.

**Figure 1:** Graph of rate to mutation development after failure and percentage of patients with each number of mutations after failure.

# Sub article: TUPDB0106 Dolutegravir plus rilpivirine in suppressed heavily pre-treated HIV-infected patients

**Published:** 2016-07-22

**Authors:** A Díaz, JL Casado, F Dronda, C Gómez-Ayerbe, MJ Vivancos, S Bañón, C Quereda, S Serrano, A Moreno, E Navas, MÁ Rodríguez, MJ Pérez-Elías, S Moreno

**Affiliations:** 1 Infectious Diseases, Ramon y Cajal University Hospital, Madrid, Spain; 2 Hospital Pharmacy, Ramon y Cajal University Hospital, Madrid, Spain

## Abstract

**Introduction:**

Patients with previous multiple virological failures are frequently suppressed with complex, toxic regimens. We aimed to explore the role of dolutegravir (DTG) 50 mg plus rilpivirine (RPV) 25 mg once daily in fully suppressed patients with a history of repeated treatment failures.

**Methods:**

Ongoing cohort study. Heavily pretreated patients with multiple virological failures and resistance mutations who were on complex suppressive therapy were switched to a QD dual regimen with DTG + RPV. Patients were excluded if resistance to integrase inhibitors (INI) or RPV was shown. Follow-up visits were scheduled at 4, 12, 24 and 36 weeks after switching. The main outcome variable was persistence of undetectable HIV RNA.

**Results:**

We included 38 subjects. At study entry, median age was 53.4 years, 34% were women, 68% were prior IDU, 70% were HCV-positive, and 34% had AIDS. Median nadir and current CD4+ cell count were 179 and 592 cells/mm^3^, respectively. Patients had received ART for a median 19.4 years with exposure to a median 3.6 drug families (100% NRTI, 89.5% NNRTI, 97.4% PI, 61.7% INI, 10.5% T20, 10.5% CCR5 receptor antagonist; 87% NRTI+NNRTI+PI, 52.6% NRTI+NNRTI+PI+INI). Previous failures were documented to one or more regimens including NRTI (100%), PI (71%), NNRTI (68.4%), and INI (8.1%). Patients with primary ART mutations were: 64.7% to NRTI, 37% to NNRTI, and 31.6% to PI. No INI-associated mutations were detected. Median time of undetectable HIV RNA load with current regimens was 6.7 years. Patients were taking a median 4.3 pills before switching. Only 1/38 (2.6%) patients left the study due to gastrointestinal toxicity, and another one because of DDI with omeprazole. HIV-RNA remained below 37 copies/ml in 100% (38/38) at week 4, and 97% (33/34) at week 24. CD4 count kept stable after switching (median 633 cells/mm^3^ at week 24). We observed a statistically improvement in triglycerides and liver tests, with no changes in total, HDL- and LDL-cholesterol. CKD-EPI eGFR decreased from 85 to 74 ml/min/1.73m^2^ (95% CI: 5.1–16.6, p=0.0002) at week 24.

**Conclusions:**

Switching to a simple, dual regimen of DTG plus RPV in heavily pretreated, multiply failed, suppressed HIV-infected patients is safe and highly efficacious.

# Sub article: TUPDC0101 Achieving UNAIDS 90-90-90 targets in a high HIV burden district in KwaZulu-Natal, South Africa

**Published:** 2016-07-22

**Authors:** A Grobler, A Kharsany, C Cawood, D Khanyile, A Puren, L Madurai

**Affiliations:** 1 Centre for the AIDS Programme of Research in South Africa (CAPRISA), Durban, South Africa; 2 Epicentre AIDS Risk Management (Pty) Limited, Pietermaritzburg, South Africa; 3 Centre for HIV and STIs, National Institute for Communicable Diseases, National Health Laboratory Service (NICD/NHLS), Johannesburg, South Africa; 4 Global Clinical and Virology Laboratory, Amanzimtoti, South Africa

## Abstract

**Introduction:**

With the goal of eliminating new HIV infections, the Joint United Nations Programme on HIV/AIDS (UNAIDS) set ambitious 90-90-90 targets to be achieved by 2020: including 90% of people living with HIV knowing their HIV status, 90% of these receiving antiretroviral therapy (ART) and 90% of these having viral suppression. Delivery of medical care to HIV-positive individuals requires a sequence of diagnostic tests, assessments and monitoring, termed the “HIV treatment cascade.” The aim of this analysis is to quantify the current achievement and gaps in this cascade for participants enrolled in the HIV Incidence Provincial Surveillance System (HIPSS) in South Africa.

**Methods:**

HIPSS is a household survey of HIV-prevalence and incidence in the uMgungundlovu District in KwaZulu-Natal in 2014 and 2015. Households within selected enumeration areas were randomly selected and a single randomly selected eligible (15–49 years) individual was invited to complete a questionnaire and provide blood samples for HIV-antibody and viral load testing.

**Results:**

A total of 9812 participants (3547 (36.1%) males and 6265(63.9%) females) were enrolled. HIV prevalence was 28.0% (95% CI: 25.9–30.1) among males and 44.1% (95% CI: 42.3–45.9) among females. First 90: 51.8% (95% CI: 47.4–56.3) of males and 64.6% (95% CI: 61.9–67.3) of females who are HIV positive knew their HIV status, p<0.001. Second 90: 69.1% (95% CI: 63.4–74.9) of males and 70.3% (95% CI: 67.6–73.0) of females who knew their HIV status were on ART. Third 90: 85.5% (95% CI: 80.1–90.1) of males and 89.7% (95% CI: 87.3–92.0) of females on ART had suppressed viral load (<1000 copies/ml). Among all HIV positive participants 44.1% of males and 58.2% of females had suppressed viral load. More than 80% of both males and females who have not tested for HIV reported that they did not test for HIV because they were afraid to know their results.

**Conclusions:**

All three elements of UNAIDS 90-90-90 targets were below 90%. The gap was largest for the first 90, especially amongst men where only half knew their status. Campaigns to increase HIV-testing are needed and reduce fear, especially amongst men. The target best accomplished is achieving suppressed viral load on ART; highlighting the success of ART in achieving viral suppression, if accessed.

# Sub article: TUPDC0102 Analysis of age- and sex-specific HIV care cascades in South Africa suggests unequal progress towards UNAIDS 90-90-90 treatment targets

**Published:** 2016-07-22

**Authors:** WB MacLeod, N Fraser, J Bor, Z Shubber, S Carmona, Y Pillay, M Gorgens

**Affiliations:** 1 Health Economics and Epidemiology Research Office, Department of Internal Medicine, School of Clinical Medicine, Faculty of Health Sciences, University of the Witwatersrand, Johannesburg, South Africa; 2 Department of Global Health, Boston University School of Public Health, Boston, MA, United States; 3 Center for Global Health and Development, Boston University School of Public Health, Boston, MA, United States; 4 World Bank, Washington, DC, United States; 5 Department of Molecular Medicine and Haematology, Faculty of Medical Sciences, University of the Witwatersrand, Johannesburg, South Africa; 6 National Department of Health, Pretoria, South Africa

## Abstract

**Introduction:**

South Africa has adopted UNAIDS’ treatment targets of 90% of people living with HIV (PLHIV) tested for HIV, 90% of those tested on antiretroviral treatment (ART), and 90% of those on ART having a suppressed HIV RNA viral load (VL). Using innovative record linkage techniques, we constructed HIV care cascades (HCC) to measure progress to these targets.

**Methods:**

We defined the HCC for April 2014–March 2015 using four categories:

1. PLHIV,
2. engaged in HIV care,
3. on ART, and
4. virally suppressed.

PLHIV numbers were estimated using population size and HIV prevalence data from StatsSA and the Human Sciences Research Council. Numbers engaged in care were calculated as the number of individual patients with a CD4 count or viral load during this period, as assessed in newly-deduplicated data from the National Health Laboratory Service. Patients on ART were reported from District Health Information System. Persons with a VL test result <400 copies/ml were considered suppressed.

**Results:**

Figure 1 shows HCC by sex and age group. Overall there were an estimated 6.47 million PLHIV (61% female, 6% aged <15 years), of whom 53% were in care, 46% on ART and 26% VL suppressed. An estimated 3.02 million PLHIV are either not yet diagnosed with HIV or diagnosed but not engaged in HIV care. Men and children aged <15 years fared worse across the cascade.

**Conclusions:**

Large gaps remain across the HCC in South Africa, particularly those engaged in HIV care. In order to meet the 90-90-90 treatment targets, 73% of PLHIV in South Africa need to be virologically suppressed (far higher than the current 26%). Increasing the testing and initiation of those newly diagnosed on ART will have the largest impact on meeting these targets. Excellent monitoring data linking patients to their laboratory results are essential.

**Abstract TUPDC0102–Figure 1:** South African HIV care cascade by sex and age group, April 2014–March 2015.

# Sub article: TUPDC0103 Cross-sectional estimates of HIV incidence remain high in rural communities in Botswana in the era of successful scale-up of ART

**Published:** 2016-07-22

**Authors:** S Moyo, T Mohammed, KP Kotokwe, C Boleo, L Mupfumi, S Gaseitsiwe, RM Musonda, K Bennett, E van Widenfelt, T Gaolathe, M Mmalane, MP Holme, N Khan, R Wang, K Wirth, EJ Tchetgen Tchetgen, S Lockman, JM Makhema, M Essex, V Novitsky

**Affiliations:** 1 Botswana Harvard AIDS Institute (BHP), Gaborone, Botswana; 2 Division of Medical Virology, Faculty of Medicine and Health Sciences, University of Stellenbosch, Stellenbosch, South Africa; 3 Bennett Statistical Consulting Inc., Ballston Lake, NY, United States; 4 Department of Immunology and Infectious Diseases, Harvard T.H. Chan School of Public Health, Boston, MA, United States; 5 Department of Medicine, Massachusetts General Hospital, Boston, MA, United States; 6 Department of Pediatrics, Massachusetts General Hospital, Boston, MA, United States; 7 Department of Biostatistics, Harvard T.H. Chan School of Public Health, Boston, MA, United States; 8 Department of Medicine, Brigham and Women's Hospital, Boston, MA; 9 Department of Neurology, Brigham and Women's Hospital, Boston, MA, United States; 10 Department of Epidemiology, Harvard T.H. Chan School of Public Health, Boston, MA, United States; 11 Division of Infectious Diseases, Department of Medicine, Brigham and Women's Hospital, Boston, MA, United States

## Abstract

**Introduction:**

The successful scale up of the national ART programme in Botswana has almost reached the UNAIDS “90-90-90” goal (Gaolathe et al., CROI-2016). Cross-sectional estimate of HIV incidence needs to take into account ARV use to avoid mis-classification of HIV recency.

**Methods:**

Using cross-sectional sampling, HIV recency was estimated at the baseline of the Botswana Combination Prevention Project in 30 rural communities from November 2013 to November 2015. The algorithm for estimation of HIV recency combined Limiting-Antigen Avidity Assay (LAg) data, ART status and HIV-1 RNA load (as described in Rehle et al., *PLoS One* 2015;10:e0133255). The LAg cut-off normalized optical density was 1.5. ART status was documented. The HIV-1 RNA cut-off was 400 copies/ml. The mean duration of recent infection was 130 days and the false recent rate was zero.

**Results:**

During the baseline household survey, a total of 3596 individuals tested HIV-positive among 12,570 individuals with definitive HIV status. Among those testing HIV-positive, 3585 (99.7%) had a research blood draw available, of whom 3580 (99.9%) had LAg data generated. Of those, 326 were identified as LAg-recent cases. Among those, 278 individuals were considered chronically infected based on their documented ART status. Among the remaining 48 ART-nave individuals, 14 had an HIV-1 RNA load ≤400 copies/ml. The Botswana MoH electronic medical records system was queried for these 14, 10 were found in the MoH data and evidence for initiation of ART was found for 5 individuals. ARV-nave status could not be confirmed in 9 individuals. Thus, 34 LAg-recent, ARV-nave individuals with HIV-1 RNA above 400 copies/ml were classified as individuals with recent HIV infections. HIV incidence was estimated at 1.06% (95% CI: 0.70–1.42%). Including 9 virologically suppressed individuals with uncertain ART status brings the estimate of HIV incidence to 1.34% (95% CI: 0.91–1.77%).

**Conclusions:**

Using an algorithm including LAg-Avidity EIA, documented ART status and HIV-1 RNA load, cross-sectional HIV incidence in 30 rural communities in Botswana was estimated at 1.06–1.34% in 2013–2015. Given the high level of ART scale-up in Botswana, studies able to identify HIV transmission sources and reduce HIV incidence are warranted.

# Sub article: TUPDC0104 How close to 90-90-90? Measuring undiagnosed HIV infection, ART use and viral suppression in a community-based sample from Namibia's highest prevalence region

**Published:** 2016-07-22

**Authors:** T Nakanyala, SV Patel, S Sawadogo, AD Maher, KM Banda, A Wolkon, S Chaturvedi, P Luphala, AM-A Agovi, MR Chipadze, C Ntema, D Prybylski, I Mabuku, DW Lowrance, N Hamunime, S Agolory, W McFarland

**Affiliations:** 1 Directorate for Special Programs, Ministry of Health and Social Services, Windhoek, Namibia; 2 Division of Global HIV and AIDS, US Centers for Disease Control and Prevention, Atlanta, United States; 3 Division of Global HIV and AIDS, US Centers for Disease Control and Prevention, Windhoek, Namibia; 4 Global Health Sciences, University of California San Francisco California, Windhoek, Namibia; 5 Global Health Sciences, University of California San Francisco California, San Francisco, United States; 6 Total Control of the Epidemic, Development Aid People to People, Zambezi, Namibia; 7 Directorate for Special Programs, Ministry of Health and Social Services, Zambezi, Namibia; 8 Division of Global HIV and AIDS, US Centers for Disease Control and Prevention, Port-au-Prince, Haiti

## Abstract

**Introduction:**

Data on the continuum of HIV care are necessary to track progress in response to the epidemic; however, they are difficult to obtain, particularly at a sub-national level. We directly measured HIV diagnosis, receipt of ART, and viral suppression in a community-based sample of adults in Zambezi, the region of highest HIV prevalence in Namibia.

**Methods:**

A cross-sectional, household-based survey was conducted from 12/2014 to 7/2015 in five purposefully selected sites of Namibia's Zambezi region. Adults received HIV rapid testing using the national algorithm, completed behavioural interviews, and submitted dried blood spots (DBS) in their homes. Previous HIV diagnosis and receipt of ART within the past 90 days were measured through self-report and verified in patient-carried records when available. HIV-RNA viral load was quantified using DBS (Abbott Real-Ti*me* HIV-1 m2000 platform). Multivariable logistic regression was used to characterize disparities in outcomes.

**Results:**

We enrolled 2163 adults, of whom 1312 (60.7% (95% CI: 58.6–62.7)) were female and 461 (21.3% (95% CI: 19.6–23.1) were HIV-positive. Among HIV-positives, 293 (63.6% (95% CI: 59.0–68.0)) were previously diagnosed. Among those diagnosed, 242 (82.6% (95% CI: 77.8–86.8)) were receiving ART. Of 209, DBS tested from participants receiving ART, 170 (81.3% (95% CI: 75.4–86.4)) were virally suppressed (i.e. <1000 copies/µl), which equates to 36.9% (95% CI: 32.5–41.5) viral suppression among all HIV-positive adults. HIV diagnosis was significantly lower among men (Adjusted odds ratio (AOR): 0.24, p<0.001) and youth (<25 years) (AOR: 0.15, p=0.02). Receipt of ART was somewhat lower among rural residents (AOR: 0.33, p=0.08). Viral suppression was significantly lower among youth (<25 years) (AOR: 0.27, p=0.002).

**Conclusions:**

With 83% of previously diagnosed adults receiving ART and 81% of those on ART achieving viral suppression, the second and third benchmarks of the UNAIDS “90-90-90” targets are within reach for adults in Zambezi region. However, serostatus awareness among HIV-positive adults was well below the 90% target, especially among men and youth. Thus, overall prevention impact may be limited with only 37% of HIV-positive adults having unsuppressed virus. If the population-level prevention benefits of ART are to be maximized, “test and start” policies must be strengthened with new interventions to improve serostatus awareness.

# Sub article: TUPDC0105 Assessment of the impact of early ART on sexual behaviour in INSIGHT strategic timing of antiretroviral treatment (START) trial

**Published:** 2016-07-22

**Authors:** A Rodger, F Lampe, W Burman, A Gruhlich, G Friedland, W El-Sadr, J Neaton, S Emery, G Corbelli, JM Molina, C Orkin, J Gatell, J Gerstoft, K Ruxrungtham, M Barbosa de Souza, A Phillips, for the International Network for Strategic Initiatives in Global Hiv Trials (insight) Start Study Group

**Affiliations:** 1 Research Department of Infection, University College London, London, United Kingdom; 2 University of Colorado, Denver, CO, United States; 3 Kirby Institute, Sydney, NSW, Australia; 4 Yale University School of Medicine, New Haven, CT, United States; 5 Columbia University, New York, NY, United States; 6 University of Minnesota, Minneapolis, MN, United States; 7 European AIDS Treatment Group, Bruxelles, Belgium; 8 Université de Paris Diderot, Paris, France; 9 Barts Health NHS Trust, London, United Kingdom; 10 University of Barcelona, Barcelona, Spain; 11 Copenhagen University Hospital, Copenhagen, Denmark; 12 Chulalongkorn University, Bangkok, Thailand; 13 Universidade Federal do Rio de Janeiro, Rio de Janeiro, Brazil

## Abstract

**Introduction:**

Antiretroviral treatment (ART) reduces HIV infectiousness, but effect on sexual behaviour is unclear. The effect of early versus deferred ART on condomless sex was assessed in the START trial.

**Methods:**

HIV+ people with CD4>500 mm^3^ were enrolled from 35 countries (April 2009 to December 2013) and randomized to immediate or deferred (CD4<350 cells/mm^3^) ART. A sexual behaviour questionnaire was completed at baseline, 4, 12 and 24 months. Three risk measures were used:

- all condomless sex (CLS)
- condomless sex with HIV negative (or unknown status) partners (CLS-D)
- HIV transmission risk sex defined as CLS-D and not on ART for=6 months *or* viral load (VL) >200 copies/ml *or* no VL within 6 months (CLS-D-HIV-risk). The pre-planned primary outcome was CLS-D at month 12, with separate analyses for men-who-have-sex-with-men (MSM) and heterosexuals.

**Results:**

A total of 4685 HIV+ participants were randomized; 2620 (55.9%) MSM; 808 (17.3%) heterosexual men and 1257 (26.8%) women. Recruitment region: 33% Europe/Israel; 25% Latin America; 21% Africa; 11% North America; 8% Asia; 3% Oceania. Median (IQR) age 36 years (29, 44); 45% reported White, 30% Black and 14% Hispanic ethnicity. Seventeen percent (764/4605) reported CLS-D at baseline (MSM (20%), heterosexuals (13%)). Among MSM, there was no difference in CLS-D prevalence at month 12 or month 24. Among heterosexuals, at month 12, CLS-D prevalence was 11% in the immediate arm versus 8% in the deferred arm (p=0.066) and at month 24, 10% versus 6% (p=0.004). In both MSM and heterosexuals, CLS-D-HIV-risk was substantially lower in the immediate versus deferred arms at months 12 and 24 due to VL suppression on ART.

**Conclusions:**

While no difference in condomless sex with sero-different partners (CLS-D) was noted between immediate versus deferred arms among MSM at 12 or 24 months, among heterosexuals there was evidence of higher levels of CLS-D in immediate versus deferred.

**Abstract TUPDC0105–Table 1:** Sexual behaviour at baseline, 12 and 24 months in the START Trial: comparison of immediate ART versus deferred ART in MSM and heterosexuals

*p- values by chi-squared tests*	MSM immediate ART (N=1314)	MSM deferred ART (N=1306)	p	Heterosexual immediate ART (N=1012)	Heterosexual deferred ART (N=1053)	p
Baseline CLS*	427/1090 (39.2%)	429/1094 (39.2%)		239/914 (26.1%)	253/941 (26.9%)	
**12 months CLS**	359/1201 (29.9%)	367/1148 (32.0%)	0.28	177/909 (19.5%)	195/931 (20.9%)	0.43
**24 months CLS**	357/1062 (33.6%)	349/1032 (33.8%)	0.92	153/719 (21.3%)	110/714 (15.4%)	0.004
Baseline CLS-D*	253/1281 (19.8%)	257/1283 (20.0%)		132/1002 (13.2%)	122/1039 (11.7%)	
**12 months CLS-D**	156/1232 (12.7%)	155/1185 (13.1%)	0.76	101/933 (10.8%)	80/959 (8.3%)	0.066
**24 months CLS-D**	173/1064 (16.3%)	152/1035 (14.7%)	0.32	69/724 (9.5%)	40/720 (5.6%)	0.004
Baseline CLS-D-HIV-risk*	253/1281 (19.8%)	257/1283 (20.0%)		132/1002 (13.2%)	122/1039 (11.7%)	
**12 months CLS-D-HIV-risk**	3/1232 (0.2%)	130/1185 (11.0%)	<0.001	6/933 (0.6%)	74/959 (7.7%)	<0.001
**24 months CLS-D-HIV-risk**	6/1064 (0.6%)	97/1035 (9.4%)	<0.001	6/724 (0.8%)	33/720 (4.6%)	<0.001

* See “Methods” for definitions of (i) CLS, (ii) CLS-D (iii) CLS-D-HIV-risk.

# Sub article: TUPDC0106 Analysis of the effectiveness of the OI/ART programme in Hwange district, Matebeleland North Province, Zimbabwe: lessons learned from viral load roll-out programme

**Published:** 2016-07-22

**Authors:** N Masuka, TP Goverwa-Sibanda, T Maphosa, ES Tshuma, W Kurauone, K Ngarivume, T Masungo, RW Carroll, P Greiger-Zanlungo, G Blick

**Affiliations:** 1 Provinical Medical Director, Ministry of Health and Child Care, Bulawayo, Zimbabwe; 2 Provinicial Maternal and Child Health Officer, Ministry of Health and Child Welfare, Bulawayo, Zimbabwe; 3 Provinical STI/HV Coordinator, Ministry of Health and Child Care, Bulawayo, Zimbabwe; 4 Ministry of Health and Child Care, Bulawayo, Zimbabwe; 5 District Medical Officer, Ministry of Health and Child Care, Victoria Falls, Zimbabwe; 6 Medical Director, BEAT AIDS Project Zimbabwe, Victoria Falls, Zimbabwe; 7 Laboratory Scientist, BEAT AIDS Project Zimbabwe, Victoria Falls, Zimbabwe; 8 Outreach and Testing Coordinator, World Health Clinicians, Inc., Norwalk, United States; 9 Montefiore Mount Vernon Hospital, Mount Vernon, United States; 10 Chief Medical Officer, World Health Clinicians, Inc, Norwalk, United States

## Abstract

**Introduction:**

Hwange district (HD) is one of the districts with a high HIV burden in Zimbabwe (ZIM) with a prevalence of 18% against a national prevalence of 14.9%. In HD, 11,661 (96%) adults and 622 (81%) children were receiving ART through the public health sector by end 2015. The need to monitor such a large cohort of patients becomes very important with the country's adoption of the “90-90-90” commitment. Despite the adoption of viral load (VL) as gold standard to monitor patients on ART in December 2013, VL testing in ZIM is still very low, having achieved 3% in 2014. This was even lower in Hwange district. The Ministry of Health and Child Care began a nationwide VL Roll-Out Programme in June 2015.

**Methods:**

The district received a TaqMan96 VL analyzer through partner support and 2408 routine VL tests were performed through December 2015. This is higher than what has been achieved in most rural districts in Zimbabwe. We analyzed the VL results dataset to assess OI/ART programme performance in relation to achieving the third “90”.

**Results:**

A total of 2409 VL results were analyzed, of which 759 (63%) were female and 424 (37%) were male. The median age was 40 (IQR: 33, 48); 1170 (99%) were on ART and, of these, 90% were on ART for more than 12 months. Viral suppression (VL<1000) was achieved in 82.7% (86% of females, 79% males; p=0.006). Fifty-seven percent of those below 20 years of age compared to 85% of those above 20 were virally suppressed (p=0.001). There were no statistically significant differences between patients followed-up through outreach into the rural communities (81.2%) and at the municipal hospital (83.1%) (p=0.31).

Mean PCR was 45,119 copies/ml (median 19; IQR: 0, 83); Mean log PCR 1.37 (median 1.28; IQR: 0, 1.92). Eighteen percent on ART had PCR >1000 copies/ml (14% >10,000; 7%>100,000).

**Conclusions:**

Routine VL testing to be done on all patients on treatment and follow-up to be made on all patients who were not virally suppressed. There is need to capacitate the programme to conduct drug resistance testing for failing patients. Special focus and close monitoring needs to be placed on adherence support for adolescents and young adults to improve adherence to treatment.

# Sub article: TUPDD0101 A right to preventative care in prisons: motivating prisoners’ access to condoms in southern Africa

**Published:** 2016-07-22

**Authors:** A Raw

**Affiliations:** Health Rights Programme, Southern Africa Litigation Centre, Johannesburg, South Africa

## Abstract

**Introduction:**

Despite high levels of HIV transmission in prisons and the southern Africa region's disproportionate share of the global HIV burden, South Africa and Lesotho are the only two countries in the region in which condoms are, in policy at least, made accessible to prisoners. Criminal sanctions against consensual same-sex sexual acts in many jurisdictions and prohibitions on sexual contact amongst prisoners are often cited as legal impediments to policy change on the issue.

**Methods:**

The paper analyses statutory frameworks and jurisprudence in selected jurisdictions in the southern Africa region where condom access is refused to prisoners. A legal argument is developed to establish that prisoners have a right to preventative care which includes access to condoms, irrespective of criminal provisions outlawing consensual same-sex sexual acts or legal restrictions on sexual contact in prisons.

**Results:**

Prisoners have a legal right to access preventative care interventions and prison authorities are legally obliged to prevent the spread of disease in prisons. By applying a “doctrine of double effect” to the position of prison health authorities who distribute preventative measures such as condoms, the fulfilment of this obligation is legally justified irrespective of criminal sanctions or administrative prohibitions against sexual contact between prison inmates.

**Conclusions:**

Advocacy to advance prison health services in southern Africa, through ensuring access to condoms and other measures to prevent the sexual transmission of HIV between prisoners, can be strengthened by legal arguments within existing legal frameworks. While criminal sanctions against consensual same-sex sexual acts ought to be challenged as infringing human rights protections and harming public health imperatives in their own right, motivating access to preventative care for prisoners need not necessarily be reliant in legal argument on the reform of those laws. Supporting advocacy initiatives within the existing legal frameworks may assist in countering popular justifications cited as legal impediments to making HIV prevention methods accessible in prisons.

# Sub article: TUPDD0102 “Mmangwana o tshwara thipa kabohaleng” – the mother of a child holds the knife on the sharper edge: improving health outcomes for children of sex workers

**Published:** 2016-07-22

**Authors:** I Lakhani, D Dlamini

**Affiliations:** 1 Advocacy and Human Rights Defence, Sex Worker Education and Advocacy Taskforce, Cape Town, South Africa; 2 Advocacy and Human Rights Defence, Sisonke National Sex Worker Movement of South Africa, Cape Town, South Africa

## Abstract

**Introduction:**

“Children of sex workers deserve the right to education, health and safety, the government should respect my job and decriminalise sex work for my children's future” (Duduzile Dlamini, Sisonke Mobilizer and Mother's for the Future founder).

While sex workers enter the industry for numerous and complex reasons, one reoccurring theme that motivates many sex workers is children. A survey of 200 South African female sex workers by the Sex Workers Education and Advocacy and Taskforce found that those participating in the study were supporting 279 children in total. Studies have also shown incredibly high HIV prevalence rates among female sex workers in South Africa (ranging between 39.7 and 71.8%). What these studies show is that mothers whose primary source of income is sex work are in a very precarious and extremely vulnerable situation. Not only is their work fully criminalized (they constantly face the threat of arrest and violence at the hands of the police), but the stigma and discrimination they face as sex workers greatly limits their ability to access health services for themselves and their children. It is in this context that “Mothers for the Future” (M4F) was founded in 2013.

**Description:**

M4F is a programme that supports mothers who do sex work by providing a safe space as well as knowledge sharing, skills building, stigma reduction in addition to advocating for the decriminalization of sex work.

**Lessons learned:**

This programme illustrates that access to healthcare cannot be addressed in isolation from the broader social justice struggles. Through the use of a number of methodologies and tools M4F attempts to create integrated support that ranges from addressing urgent short-term needs such as accessing toiletries, medication, school fees etc. and the more long term goal of law reform and decriminalizing sex work. M4F is a powerful example of the efficacy of sex worker-led interventions and how providing comprehensive support to mothers can result in better overall health outcomes for their children.

**Conclusions/Next steps:**

Creating better health outcomes for the children of sex works needs more than just a service delivery approach. Holistic support within a strong human rights framework is essential.

# Sub article: TUPDD0103 Human rights and ethical dilemmas in the implementation of Option B+ in Malawi

**Published:** 2016-07-22

**Authors:** A Munthali, B Chinsinga, J Kadzandira, B Ngwira, F Masiye, B Kaunda-Khangamwa

**Affiliations:** 1 Centre for Social Research, University of Malawi, Zomba, Malawi; 2 The Polytechnic, University of Malawi, Blantyre, Malawi; 3 College of Medicine, University of Malawi, Blantyre, Malawi

## Abstract

**Introduction:**

Malawi pioneered Option B+ in July 2011. There have been concerns that with Option B+ pregnant and lactating women are forced to undergo HTC and start ART. The approach is also perceived as discriminatory as it offers ART only to women and not their spouses who may also be HIV+. Overall the delivery of Option B+ is perceived as a threat to patient rights concerning consent, confidentiality and counselling. This study explored people's perceptions about human rights and ethical issues surrounding the delivery of Option B+ in Malawi.

**Methods:**

We collected data in 15 districts across Malawi. We conducted 18 key informant interviews at national level, 84 interviews with women on Option B+ and their spouses, 28 interviews with community leaders; 56 focus group discussions with community members, 42 focus group discussions with women on Option B+ and 42 interviews with service providers. Content analysis was used to analyze the data.

**Results:**

While some study participants viewed Option B+ as mandatory, hence breaching women's right to making decisions, most of them reported that women make their own decisions after appropriate counselling. Most study participants had no problems with the prioritization of pregnant and lactating women as it aimed at ensuring babies were born HIV uninfected. A few study participants, however, said that the procedure is ethically unfair as it does not offer ART to spouses who may also be HIV+ and this may cause strained relationships within the household. Lack of male involvement, fear of divorce, fear of stigma and discrimination and in some cases the low quality of counselling services constitute the most common barriers to Option B+ implementation.

**Conclusions:**

There were a few participants who raised human rights and ethical issues surrounding implementation of Option B+. However the advantages of the programme including improved health and ensuring children are born free of HIV outweigh the human rights and ethical concerns.

# Sub article: TUPDD0104 A rights-based approach to HIV, fair migration and health: a global framework for action

**Published:** 2016-07-22

**Authors:** A Torriente, M Licata, S Mabhele

**Affiliations:** 1 International Labour Office (ILO), ILOAIDS, Geneva, Switzerland; 2 International Labour Office (ILO), ILOAIDS, Pretoria, South Africa

## Abstract

**Introduction:**

The ILO estimates that there are 232 million migrant workers worldwide – 48% are women. Many face obstacles that place their health at risk and heighten their vulnerability to HIV. To reduce migrants’ HIV risk, it is essential to ensure their equal access to health services in countries of origin, transit and destination.

Migration alone is not a risk factor for HIV, but factors associated with migration are. These include discrimination, poor living/working conditions, sexual violence during migration and unfair migration practices.

**Description:**

The ILO has developed a Framework Guidance Document to promote engagement and support contributions of all partner organizations to the new UNAIDS Strategy 2016–17 in the areas of migration and mobility. The Framework contains four key elements:

- Analysis of new/emerging trends and developments around labour migration and associated HIV vulnerabilities
- Analysis of gender-specific dimensions of migration and their impact on HIV, health and migration policies
- Overview of international human rights law guiding guide labour migration governance and its interaction with health and social protection.
- Overview and examples of good practices around HIV and health programmes for migrants across countries/regions

The end result is a global framework for action which includes:

1. recommendations and evidence-informed guidance on integrating access to HIV and health services into labour migration processes at all stages of migration; and
2. roles/responsibilities of national stakeholders on policy making and programme implementation around HIV and health issues in the context of migration.

**Lessons learned:**

A fair labour migration agenda must address health deficits experienced by migrants in countries of origin, transit and destination. Bilateral agreements and migration policies and programmes should thus integrate health service access for migrant workers.

**Conclusions/Next steps:**

Based on the Framework, the ILO will seek to engage organizations working on migration and HIV in coordinated efforts to:

- guide development and implementation of rights-based programmatic and policy responses integrating fair labour migration practices that increase migrants’ access to health services, and
- guide national stakeholders in integrating HIV and health programmes for migrant workers into ational migration policies and bilateral agreements.

# Sub article: TUPDD0105 Implementing a human rights monitoring and response system (REAct) in Burundi

**Published:** 2016-07-22

**Authors:** A Virga, C Nininahazwe, N Ndayizeye, JD Kabanga, The Link Up project

**Affiliations:** 1 International HIV/AIDS Alliance, Brighton, United Kingdom; 2 RNJ+ (Burundi's National Network of Young People Living with HIV), Bujumbura, Burundi; 3 Humure, Bujumbura, Burundi; 4 Alliance Burundaise contre le SIDA (ABS), Bujumbura, Burundi

## Abstract

**Introduction:**

Link Up is a four year project funded by the Dutch government aimed at improving the sexual and reproductive health of young people from key populations in Bangladesh, Burundi, Ethiopia, Myanmar and Uganda. Rights-Evidence-Action (REAct), is a human rights monitoring and response system developed by the International HIV/AIDS Alliance to help provide evidence for human rights programming in Link Up.

**Description:**

Alliance Burundaise contre le SIDA (ABS) has trained two of its implementing partners – RNJ+ (young people living with HIV) and Humure (LGBTI association) – on REAct. REAct is a community-driven system, which community-based organizations use to document human rights-related barriers in accessing HIV and health services and human rights violations. Information collected through interviews is used to provide individual responses to violations and to inform human rights-based HIV programming.

ABS, RNJ+ and Humure will be implementing REAct in Burundi's fragile and conflict affected context. The Burundi team is already aware of many cases of physical violence and discrimination faced by young people living with HIV and the LGBT and sex work communities when accessing HIV and SRHR services.

**Lessons learned:**

It was important to develop customized questionnaires in French, specific to people living with HIV, LGBTI and sex workers in Burundi.

- Young people living with HIV and young LGBT people are well-placed to administer the questionnaires and respond to clients’ immediate needs because of their regular contact with clients
- Rights violations identified by implementing organizations in preparation for the documentation of cases through REAct include refusal to provide post-abortion care (abortion is highly restricted); discrimination of children living with HIV in schools; refusal to treat MSM in healthcare facilities; breach of confidentiality in healthcare settings; and failure to obtain clients’ informed consent to receive services

**Conclusions/Next steps:**

Collecting evidence of human rights violations is an essential step in advocating for law reform and policies to address violence and discrimination against young people from key populations. It is possible to document human rights violations in conflict affected countries; and ensuring the safety and security needs of interviewers and interviewees is a critical part of the training for the project.

# Sub article: TUPDD0106 Removing human rights barriers to evidence-based HIV prevention, care and treatment: what do we know?

**Published:** 2016-07-22

**Authors:** A Stangl, D Singh, M Windle, K Sievwright, K Footer, A Iovita, S Mukasa, S Baral

**Affiliations:** 1 Global Health, International Center for Research on Women, Washington, DC, United States; 2 Epidemiology, Johns Hopkins Bloomberg School of Public Health, Baltimore, MD, United States; 3 Joint United Nations Programme on HIV/AIDS, Geneva, Switzerland

## Abstract

**Introduction:**

Repressive legal environments and widespread human rights violations act as structural impediments to efforts to engage key populations at risk of HIV infection in HIV prevention, care, and treatment efforts. The identification of human rights programmes and rights-based interventions that enable coverage of and retention in evidence-based HIV prevention and treatment approaches is crucial for achieving an AIDS-free generation.

**Methods:**

We conducted a systematic review of studies that assessed the effectiveness of human rights interventions on improving HIV-related outcomes between 1/1/2003 and 28/3/2015. A comprehensive and systematic search protocol was iteratively developed including databases for both peer-reviewed and non-peer-reviewed reports. Ancestry searches for articles included in the review were also conducted. Studies of any design that sought to evaluate an intervention falling into one of the following key human rights programme areas defined by UNAIDS were included with independent and dual-data abstraction at all stages: HIV-related legal services; monitoring and reforming laws, policies, and regulations; legal literacy programmes; sensitization of lawmakers and law enforcement agents; and training for health care providers on human rights and medical ethics related to HIV.

**Results:**

Of 31,861 peer-reviewed articles and reports identified, 24 were included in our review representing 15 different populations across 14 countries. The majority of studies incorporated two or more of the principles of the human rights-based approach, most often non-discrimination and accountability, and sought to influence two or more elements of the right to health, namely availability and acceptability. Half of the interventions addressed multiple UNAIDS’ key programme areas, with monitoring and reforming laws and sensitizing law makers the most common. However, most interventions targeted a single socio-ecological level, namely public policy. Outcome measures varied considerably, making comparisons between studies difficult, and only a few studies explicitly referenced the promotion and protection of human rights.

**Conclusions:**

The majority of studies reported a positive influence of human rights interventions on HIV-related outcomes. Yet, limited financial support for methodologically sound evaluations of human rights interventions limits the generalizability of these findings. Fast-tracking HIV prevention and expanding treatment approaches to achieve sufficient coverage will require effective structural interventions implemented in coordination with biomedical approaches.

# Sub article: TUPDD0107LB Impact of closing space for civil society on LGBT groups in Kyrgyzstan, Indonesia, Kenya and Hungary

**Published:** 2016-07-22

**Authors:** SLM Davis, M Hart

**Affiliations:** 1 Center for Human Rights and Global Justice, New York University, New York, NY, United States; 2 Global Philanthropy Project, New York, NY, United States

## Abstract

**Introduction:**

In response to both the threat of terrorism and the growing populist pressure for transparency and government accountability, many countries are using new laws and tactics to restrict freedom of association and freedom of expression. The study by Global Philanthropy Project, a network of private foundations, aimed to assess the specific impact of civil society restrictions on LGBT groups through four case studies.

**Methods:**

Kyrgyzstan, Indonesia, Kenya and Hungary were selected as representatives of trends in the four regions of Central Asia, Southeast Asia, East Africa and Europe. Over 2 months, researchers conducted desk review, as well as guided interviews with 19 LGBT activists, scholars, human rights experts and UN officials from the four countries.

**Results:**

The report finds that while the four contexts had key differences, overall LGBT groups have always faced restrictions. In recent years a combination of new “LGBT propaganda” laws, resurgent nationalism, religious fundamentalism and political scapegoating of LGBT people as “foreign agents” promoting “foreign values” together combine to heighten the risk environment to individuals and groups. LGBT groups are also forming new alliances and developing innovative approaches to continue their work.

**Conclusions:**

UN partners and donors should monitor ways that closing space for civil society impacts on LGBT groups, which are critical as partners, watchdogs and HIV service providers and should support LGBT alliance-building and advocacy to resist closing space.

# Sub article: TUPDD0201 A Hackathon for HIV and STD prevention: using mobile technologies to expand access to information and HIV prevention, testing and care services among young key populations in Brazil

**Published:** 2016-07-22

**Authors:** C Euzébio de Lima, D De Castro, M Elizabeth de Lima Pereira, D Ferreira Santana, P Aguiar

**Affiliations:** 1 UNAIDS, Brasilia, Brazil; 2 Brazilian Association of Companies for the Sensual and Erotic Market (ABEME), São Paulo, Brazil

## Abstract

**Introduction:**

Brazil already counts on more mobiles than inhabitants: over 269 million active mobile phone lines in the country. More than 40% of young people in Brazil (aged 18–24) report mobile phone as most used device to access internet. The Brazilian Association of Companies for the Sensual and Erotic Market and UNAIDS have joined forces to promote the 1st Hackathon on HIV and STD Prevention, aiming to develop innovative mobile apps and games that can support the expansion of access to information and health services among young people – a vulnerable group that has been facing significant increase on HIV infection rates in the past 10 years.

**Description:**

A hackathon is a digital marathon that gathers several different professionals involved in software programming including IT programmers, designers, communication professionals, entrepreneurs and others interested in technology. The Hackathon on HIV and STD Prevention was developed in two phases: 1) Information sessions (TED-Talk style meetings were carried out with the participation of specialists from health sector, sexuality, entrepreneurs and technology professionals, presenting different perspectives and opportunities in the market to the teams participating to the Hackathon; 2) Technology Marathon – 24 hour event for the development of apps/games and for the selection of best projects. Mentorship for the IT teams was provided by young MSM and young people living with HIV and health professionals (including UNAIDS technical advisers) to guarantee that the projects developed were based on real needs in prevention and human rights.

**Lessons learned:**

The use of IT tools open up a world of new possibilities to promote HIV prevention and care especially with young key populations. Bringing together young IT professionals with young MSM and PLHIV is fundamental to the development of innovative projects that are feasible, attractive and with cutting-edge and appropriate language.

**Conclusions/Next steps:**

Mobile apps market assessment needs to be taken into consideration given the huge number of free mobile apps/games competing for the attention of young audiences. The partnership with the erotic market is innovative and fruitful and represents an enormous potential to eroticize safe sex and to support the reinvention and update of HIV prevention messages.

# Sub article: TUPDD0202 “*Once you start watching this show, Intersexions, it's easier to get to the point that you really want to talk*”: how viewers' identification with Intersexions II facilitated new communicative spaces

**Published:** 2016-07-22

**Authors:** L Myers, H Hajiyiannis, T Motuba, R Delate, L Mahlasela

**Affiliations:** 1 Centre for AIDS Development, Research and Evaluation, Cape Town, South Africa; 2 Centre for AIDS Development, Research and Evaluation, Johannesburg, South Africa; 3 Centre for Communication Impact, Pretoria, South Africa

## Abstract

**Introduction:**

The successful South African television drama series, *Intersexions*, consisted of 26 interlinked episodes. While *Intersexions I* portrayed the risks of multiple and concurrent partnerships by mapping a fictional sexual network, *Intersexions II* (2013) portrayed how keeping secrets can increase risk of HIV infection through what is left unsaid or hidden in one's personal and sexual relationships.

**Methods:**

A qualitative post-broadcast evaluation consisting of 14 focus groups and 12 interviews was conducted in 6 provinces with 122 regular viewers of the series. Participants needed to have watched at least half the episodes and reflected a mix of urban, peri-urban and rural localities. Discussions were audio-recorded, transcribed and analyzed using NVivo.

**Results:**

By portraying relevant and realistic themes, *Intersexions’* unusual dramatic formula succeeded in raising awareness about the influence of communication and the sexual network on HIV risk. Reported forms of interpersonal communication demonstrated that the drama series provided a useful tool to communicate sexual health content and life lessons with sexual partners, family and friends. Such conversations strengthened norms around open communication and greater critical consciousness about the limits of trust and the potential consequences of having sexual secrets. The episodes became more than *just an episode* in the way they prompted meaningful conversation and critical reflection about topics that were previously considered too taboo to comfortably discuss. Watching *Intersexions* thus spurred a new level of openness in a number of participants' family, peer and sexual relationships and for some, engagement with the series sparked meaningful self-reported behaviour change.

**Conclusions:**

The power of mass media to create much needed spaces for interpersonal dialogue and conversation about sexuality, relationships and HIV prevention is significant. Both *Intersexions* series appear to have broken through the silence and cultured *HIV fatigue* that often accompanies efforts to raise awareness about HIV prevention, care and support. By moving viewers beyond the simple *ABCs* of HIV prevention to asking critical questions about the quality of their relationships and the ways that communication and secrets in particular contribute to HIV risk, *Intersexions* may have contributed to a more complex and multifaceted shift in the national consciousness around HIV prevention.

# Sub article: TUPDD0203 Development of a mobile-based application to increase uptake of HIV testing among young U.S. men who have sex with men

**Published:** 2016-07-22

**Authors:** K Biello, J Coffey-Esquivel, S Hosek, M Belzer, P Sullivan, M Mimiaga, S Oleson, S Taylor, L Kreh, K Mayer

**Affiliations:** 1 Behavioral & Social Sciences and Epidemiology, Brown University, Providence, RI, United States; 2 Fenway Health, The Fenway Institute, Boston, MA, United States; 3 Department of Psychiatry, Stroger Hospital of Cook County, Chicago, IL, United States; 4 Division of Adolescent Medicine, Children's Hospital Los Angeles, Los Angeles, CA, United States; 5 Department of Epidemiology, Rollins School of Public Health, Atlanta, GA, United States; 6 Keymind, Falls Church, VA, United States; 7 Division of Infectious Diseases, Harvard Medical School/Beth Israel Deaconess Medical Center, Boston, MA, United States; 8 Department of Global Health and Population, Harvard T. H. Chan School of Public Health, Boston, MA, United States

## Abstract

**Introduction:**

Young men who have sex with men (YMSM) accounted for 72% of new HIV infections among all persons aged 13 to 24, and 30% of new infections among all MSM. However, overall rates of testing among young adults are suboptimal. Though mobile app use is nearly universal among US YMSM, there are currently no HIV prevention mobile phone apps developed specifically for YMSM, suggesting a need for further research to develop these interventions.

**Methods:**

We conducted six focus groups with 33 participants (mean age=21.8, range 17–24; 36% Hispanic/Latino, 33% Black, non-Hispanic; 18% White, non-Hispanic, 12% other) in Boston, Chicago and Los Angeles. During focus group discussions, participants were shown an app developed to increase HIV testing for adult MSM, HealthMindr and screenshots of potential adaptations and provided feedback on the utility of the app, functionality of the various app features and how to best optimize the current app for use by young people prior to adaptation.

**Results:**

Participants found the essential functions of the current app to be generally useful, including the ability to locate an HIV/STI testing site near them. They suggested adding the ability for the app to “ping” them when near a testing site and due for a test. Participants remarked that this would help overcome the obstacles associated with planning, such as forgetting to test and would remove the responsibility of finding a testing location. While participants felt that the app contained an abundance of useful information and access to materials on HIV testing, PrEP and sexual health, they described the language and appearance as being very “medical” and unappealing to youth, suggesting changes to the interface, including use of avatars, infographics and less formal language.

**Conclusions:**

Overall, participants liked the app and suggested that it would help them to increase the frequency and acceptance of HIV/STI testing and improve their general sexual health. However, suggestions for changes were provided. Further app development and testing is warranted.

# Sub article: TUPDD0204 Characteristics of bisexual men who use the internet to seek sex with other men in Ontario, Canada

**Published:** 2016-07-22

**Authors:** R Souleymanov, DJ Brennan, N Lachowsky, S Fantus, A Ceranto

**Affiliations:** 1 Faculty of Social Work, University of Toronto, Toronto, Canada; 2 Fife House, Toronto, Canada

## Abstract

**Introduction:**

The use of the Internet to seek sexual partners is associated with increased HIV sexual risk behaviours for men who have sex with men (MSM). Little research, however, focuses specifically on bisexual MSM who use the Internet to seek sexual partners. In this analysis, we examined: 1) differences between bisexual and other-identified MSM with regard to HIV status, online sexual health outcomes and condom use/non-use; 2) factors associated with condom use/non-use during the most recent anal intercourse for bisexual men.

**Methods:**

Data were drawn from an online survey targeted at MSM in Ontario. Using logistic regression analysis and chi-square tests we assessed differences between bisexual-identified MSM and other-identified MSM (gay, queer) with regard to HIV status, online sexual health outcomes and condom use/non-use. Following this, we used logistic regression to examine factors associated with condom use during the most recent anal intercourse for bisexual men. All results were considered significant at p<0.05.

**Results:**

The study included a total of 1830 MSM. Among these men, 438 (24.0%) indicated “bisexual” as their sexual orientation. Bisexual men were less likely than non-bisexual-identified men to be HIV-positive (2.4% vs. 9.9%; OR=0.23, 95% CI: 0.18–0.43), to have been recently tested for STIs (45.4% vs. 65.3%; OR=0.44, 95% CI: 0.35–0.55) and to receive sexual health information online (74% vs. 85.3%; OR=0.63, 95% CI: 0.47–0.85). Bisexual men were more likely than non-bisexual-identified men to report condom-use during their last male anal sex (65.7% vs. 59.2%; OR=1.32, 95% CI: 1.02–1.72). Substance use emerged as a significant factor associated with condom use during the most recent anal intercourse for bisexual men (OR=0.50, 95% CI: 0.29–0.88).

**Conclusions:**

Bisexual men who use the Internet to seek sex with other men may exhibit distinct sexual risk behaviours (decreased rates of STI testing, less sexual health information sought online, increased condom use) compared to other MSM. In addition, further research is needed to understand the link between condom use and substance use within the context of preventing HIV risk among bisexual men who use the Internet to seek sex with other men.

# Sub article: TUPDD0205 Masivukeni: a multimedia ART initiation and adherence intervention for resource-limited settings

**Published:** 2016-07-22

**Authors:** RH Remien, CA Mellins, R Robbins, H Gouse, CS Leu, J Rowe, M Henry, L Myer, D Stein, J Joska

**Affiliations:** 1 New York State Psychiatric Institute, Columbia University, New York, NY, United States; 2 Department of Psychiatry and Mental Health, University of Cape Town, Cape Town, South Africa; 3 Center for Teaching and Learning, Columbia University, New York, NY, United States; 4 Department of Public Health and Family Medicine, University of Cape Town, Cape Town, South Africa

## Abstract

**Introduction:**

The need for ART initiation and adherence with staggering numbers of people and a reliance on lay counsellors for ART counselling remain challenging in South Africa and globally. Masivukeni, a theoretically-derived behavioural intervention, was developed to enhance the capabilities of lay counsellors to deliver ART adherence counselling to patients initiating ART. Masivukeni is a structured, laptop-based multimedia intervention requiring minimal training and supervision.

**Methods:**

We conducted a randomized controlled trial of Masivukeni at two township clinics in Cape Town, South Africa. Masivukeni is consistent with standard of care counselling (SOC) guidelines (3–4 sessions prior to/ during ART initiation, follow-up sessions for defaulters, and inclusion of treatment support partners (buddies)). Masivukeni using videos, visually-relevant content and interactive exercises that focus on key domains related to Social Action Theory (e.g. HIV/AIDS knowledge, motivation for health, mood, problem-solving skills and social support). Patients eligible for ART-initiation who provided consent were allocated 2:1 to Masivukeni or SOC. Patients were seen 12-months post-initiation, corresponding with routine clinic-based viral load testing. Qualitative interviews with counsellors were also conducted.

**Results:**

Participants included 456 HIV+ adults with 337 (74%) completing 12-month follow-up; most patients lost to follow-up transferred out of the clinic or did not initiate treatment. At enrolment 20% had TB; 73% were female; mean age was 33 years; 42% had some employment; and 96% were impoverished (<R5000/month). At follow-up, over 90% of all patients (across study arms) achieved viral suppression (VL<400copies/ml), however, proportionally, nearly twice as many SOC participants did not initiate ART compared to those in the Masuvkeni arm. Among Masivukeni participants, viral suppression was improved with increased buddy participation in counselling sessions. Counsellors found the multimedia computerized intervention easy to use, enhancing their competence and confidence in their counselling role. They also reported greater patient learning and retention.

**Conclusions:**

Masivukeni has promise as an adherence counselling tool, improving the numbers of patients who initiate treatment and strengthening the work of and empowering counsellors by standardizing and guiding their counselling interactions, providing visual aids solidifying patient understanding of HIV and its treatment and contributing to rapid ART initiation and viral suppression.

# Sub article: TUPDD0206 “TáNaMão” (inHand) app, a pocket risk calculator and free prevention service locator for cell phones and tablets

**Published:** 2016-07-22

**Authors:** A Mathias, L Abreu, T Zampieri, A Spiassi, E Gutierrez

**Affiliations:** 1 Programa Municipal de DST/AIDS de São Paulo, São Paulo, Brazil; 2 Centro de Testagem e Aconselhamento Henrique de Sousa Filho (CTA Henfil), São Paulo, Brazil

## Abstract

**Introduction:**

Recent data from epidemiological reports and surveys demonstrated increase of HIV infection, low use of condoms and lack of awareness on the use of antiretroviral drugs among young population from Sao Paulo City. Furthermore, risk management and pre-exposure prophylaxis need to be publicized to encourage their adoption by this population.

**Description:**

In order to address this problem, the STD/AIDS Program from the São Paulo City Department of Health commissioned a task force to create an app to provide accessible information in timely manner for young people. As a result, “TáNaMão” app (meaning “InHand” in English) was developed with the participation of representatives of key-populations. The user can input data and the app can calculate STD/HIV risk and provide guidance and inform the nearest place to obtain free prevention supplies and healthcare.

**Lessons learned:**

After successful test period with healthcare workers and key-population members, the app was released in social media, blogs and press. The app can be downloaded for Android and iOS. Noteworthy, Apple Store considers draws representing sexual interactions too explicit and blocks them.

**Conclusions/Next steps:**

“TáNaMão” app was downloaded 3660 times in 6 months after release, meaning that a broader promotion is required. A plan to increase app downloads through partnership with key people in online social networks and popular webpages is ongoing. A new version will have more friendly and enjoyable interface.

# Sub article: TUPDD0301 Does sexual identity matter in accessing services? Risk profile and health-seeking behaviours of different sexual identity types of young men who have sex with men in Myanmar

**Published:** 2016-07-22

**Authors:** W Tun, PP Aung, A Bajracharya, E Yam, C Ryan, SM Oo, ZW Thein, AK Paing, N Pasricha, L Willenberg, P Agius, TT Sein, S Htun, NZ Latt, S Luchters

**Affiliations:** 1 HIV and AIDS Program, Population Council, Washington, DC, United States; 2 Burnet Institute, Yangon, Myanmar; 3 Phnom Penh, Population Council, Cambodia; 4 HIV/AIDS Alliance, Yangon, Myanmar; 5 Marie Stopes International, Yangon, Myanmar; 6 Burnet Institute, Melbourne, Australia

## Abstract

**Introduction:**

Men who have sex with men (MSM) are disproportionately affected by HIV compared to the general population in Myanmar (6.6% vs. <1%). While there is increasing information on risky behaviours of MSM in Myanmar, an in-depth understanding of how the risk profile and health-seeking behaviours differ by sexual self-identities is needed to tailor HIV/STI services. This analysis compares MSM with different sexual self-identities among young Myanmar MSM.

**Methods:**

A behavioural cross-sectional survey was conducted in six townships in 2014 as a baseline for an evaluation of the Link Up project, a global consortium led by the International HIV/AIDS Alliance, to address sexual reproductive health needs of young key populations. Men (18–24 years) who had sex with a man in the previous 6 months were recruited using respondent-driven sampling to complete an interviewer-administered survey. Characteristics are compared using chi-squared test across different sexual identities: “tha-nge” (hidden MSM, insertive partner (*hidden/i*)), “apone” (hidden MSM, receptive partner (*hidden/r*)) and “apwint” (open MSM; typically receptive (*open/r*)).

**Results:**

The study enrolled 623 MSM. Respondents self-identified as hidden/i (54%), hidden/r (16%) and open/r (29%). Open/r had the highest proportion reporting STI symptoms in the last 12 months (open/r: 37%; hidden/i: 23%; hidden/r: 24%; p<0.01). All groups were equally likely to have sought STI treatment (57–65%). Open/r had the highest proportion ever having tested for HIV (open/r: 87%; hidden/i: 52%; hidden/r: 68%; p<0.001); self-reported HIV-positivity was 15.7% (open/r), 1% (hidden/i) and 7% (hidden/r) (p<0.001). While the majority of open/r and hidden/r (90–95%) revealed their male-male sexual behaviour, only 78% of hidden/i revealed such (p<0.001). Open/r were the most likely to have accessed an MSM-friendly drop-in centre (open/r: 78%; hidden/r: 69%; hidden/i: 49%; p<0.001) or been reached by a peer educator (open/r: 67%; hidden/r: 56%; hidden/i: 47%; p<0.001).

**Conclusions:**

Despite MSM being a vulnerable group overall, certain sexual identities of MSM may be even more vulnerable. The findings suggest the importance of tailoring MSM outreach and facility-based services to meet the nuanced needs of different sexual identities within MSM, particularly the hidden MSM who access services the least but are not without risk.

# Sub article: TUPDD0302 Measuring gender norms among very young adolescents (ages 10–14) and young people (ages 15–24) in Uganda: tool validity and associations with key HIV outcomes

**Published:** 2016-07-22

**Authors:** L Vu, J Pulerwitz, B Zieman, J Okal, E Yam, Link Up Project

**Affiliations:** 1 HIV and AIDS Program, Population Council, Washington, DC, United States; 2 HIV and AIDS Program, Population Council, Nairobi, Kenya

## Abstract

**Introduction:**

Gender norms are strongly associated with HIV-related factors, and they often form early in life. Very young adolescents (VYAs) ages 10–14 could benefit from gender transformative interventions, yet no tools are validated to measure gender norms among VYAs. The GEM Scale measures views towards gender norms and has proven valuable in evaluating HIV programmes for older adolescents/adults, in numerous settings. We assessed GEM Scale utility among Ugandan VYAs and compared responses of VYA and older youth.

**Methods:**

We conducted a two-stage cluster-sampled survey of 297 VYAs and 658 15–24 year-olds in rural and urban communities near Kampala. The survey included a 24-item GEM Scale. Using confirmatory factor analyses (CFA), we separately evaluated the scale among 10–14s and 15–24s. To maintain between-group comparability, we created a modified scale, omitting items with low CFA factor loadings (<0.30). We trichotomized scores into low-, moderate- and high-equitability groups and assessed bivariate associations between gender-equitability and key outcomes.

**Results:**

The GEM Scale proved an effective measure among both VYAs and older youth. For YVAs, CFA identified one latent construct with good fit (root-mean-square-of-error-approximation=0.04; Comparative-Fit-Index=0.93; Tucker-Lewis-Index=0.92), and consistency (α=0.74); eight of the original items had low factor loadings. The five items with low factor loadings in both age groups were omitted to form the final 19-item scale. The scale results were comparable for males and females.

Nearly 87% had low-to-moderate gender equitability. VYAs had lower mean scores than 15–24s (33.5 vs. 37.1; p<0.001). VYAs were three times as likely to agree that “*a man should have the final word on decisions in his home*” (p<0.001), or “*a woman who has sex before marriage does not deserve respect*” (p<0.001). Analyses showed that gender equitable norms were significantly (p<0.05) associated with key outcomes, including HIV knowledge, HIV testing and condom use.

**Conclusions:**

The GEM Scale provides a valid measure of gender norms across a wide age range, including VYAs, and is associated with important HIV outcomes. Moreover, support for equitable gender norms was generally low and lower among VYAs compared to their older counterparts. This gap may provide opportunities for gender transformative interventions for VYAs.

# Sub article: TUPDD0303 The “One Man Can” model: community mobilization as an approach to promote gender equality and reduce HIV vulnerability in South Africa

**Published:** 2016-07-22

**Authors:** A Anderson, E Stern, T Mokganyetji, D Rebombo, C MacPhail, N Khoza, S Treves-Kagan, A Selin, D Peacock, A Pettifor, S Lippman, K Kahn, R Twine

**Affiliations:** 1 Research, Monitoring and Evaluation, Sonke Gender Justice, Cape Town, South Africa; 2 School of Public Health, University of Cape Town, Cape Town, South Africa; 3 Community Education and Mobilization, Sonke Gender Justice, Johannesburg, South Africa; 4 University of New England, Armidale, Australia; 5 School of Public Health, Wits University, Johannesburg, South Africa; 6 University of North Carolina Chapel Hill, Chapel Hill, NC, United States; 7 Sonke Gender Justice, Cape Town, South Africa; 8 Department of Epidemiology, University of North Carolina Chapel Hill, Chapel Hill, NC, United States; 9 Department of Epidemiology, University of California, San Francisco, CA, United States; 10 MRC/Wits Rural Public Health and Health Transitions Research Unit, Agincourt, South Africa

## Abstract

**Introduction:**

Among other factors driving the HIV epidemic in sub-Saharan Africa are social norms reinforcing restrictive gender roles and inequitable gender relationships. These limit women's ability to protect themselves from HIV, while simultaneously put social pressure on men to take on a range of sexual health risks. To understand the benefits of engaging men and boys for gender equality and HIV prevention, this study explored the impacts of Sonke Gender Justice's “One Man Can” (OMC) community mobilization approach in a multi-level HIV intervention. The study assessed whether OMC community mobilization activities targeting young men could promote gender-equitable norms that decrease women's HIV vulnerability and men's HIV risk behaviour in South Africa.

**Methods:**

The OMC community mobilization intervention evaluation was a randomized controlled trial implemented in Agincourt, rural northeast, South Africa between 2012 and 2014. Young men (18–35 years) were the primary targets for the intervention, which included workshops and innovative outreach activities on gender equality and health. This analysis draws from qualitative data collected from intervention implementers and community members at the last time point during the intervention. Directed content analysis was employed as an analytic approach.

**Results:**

Our findings indicate significant attitudinal and some behavioural changes around gender equality and HIV risk amongst OMC intervention implementers and community members. At the interpersonal level, adoption of gender-equitable beliefs had positive effects of improved communication and a more balanced division of labour between intimate partners. At the community level, the results were mixed. OMC activities increased awareness and interest in reducing gender inequality and HIV risk. However, intervention implementers experienced some resistance from community leaders in providing training on aspects of gender equality, condom use, abortion rights and homosexuality. HIV interventions that incorporate community mobilization should explore avenues to actively engage local leaders in supporting shifts in norms around these issues.

**Conclusions:**

Community mobilization is potentially a powerful tool to promote equitable gender norms and build consciousness and action around HIV prevention. When men are mobilized to recognize how harmful gender norms negatively impact their lives, the changes they make towards improving their own health can have added benefits for the women in their lives.

# Sub article: TUPDD0304 Similar and different factors associated with transactional sex with main partners and casual sexual partners in young women in urban informal settlements in South Africa

**Published:** 2016-07-22

**Authors:** A Gibbs, N Ntini, T Khumalo, L Washington, N Mbatha, E Chirwa, S Willan, Y Sikweyiya, N Jama-Shai, R Jewkes

**Affiliations:** 1 Health Economics and HIV/AIDS Research Division, Durban, South Africa; 2 Project Empower, Durban, South Africa; 3 Gender and Health Research Unit, Medical Research Council, Pretoria, South Africa

## Abstract

**Introduction:**

Transactional sex is a significant risk factor for HIV-acquisition amongst young women. Yet there may be significant variation in the forms of transactional sex with main partners and transactional sex with casual partners. Our study sought to describe the prevalence of each form of transactional sex and compare the risk factors for each amongst young women in urban informal settlements.

**Methods:**

We drew on cross-sectional data from 320 women aged 18 to 38 in informal settlements in Durban, South Africa who comprised the control arm of a cluster randomized RCT. Primary outcomes were transactional sex with a main partner in the past 12 months and transactional sex with a kwapheni (casual) partner in the past 12 months, both assessed using five items. Other measures included socio-demographics, violence experienced and alcohol and drug use. We built Gaussian random effects regression models for each form of transactional sex.

**Results:**

Women were young (mean ages 24.4 years). 61.1% (CI: 52.1–69.5) reported transactional sex with a main partner in the past 12 months and 49% (CI: 95% 41.9–56.2) reported transactional sex in the past 12 months with a kwapheni. There was significant overlap between both forms with 41.9% (CI: 95 34.2–49.9) reporting both. Transactional sex with a main partner was significantly associated with being in a more controlling relationship (aOR1.07, p<0.05), experiencing economic violence from an intimate partner in the past 12 months (aOR2.29, p<0.01), experiencing past year non-partner sexual violence (aOR1.86, p<0.05) and women's greater alcohol use (aOR1.1 p=0.001). Transactional sex with a casual (kwapheni) partner was associated with greater hunger (aOR2.4, p<0.05), experiencing economic violence from an intimate partner in the past 12 months (aOR1.76, p<0.05), experiencing non-partner sexual violence in the past 12 months (aOR2.26, p<0.01) and using drugs in the past year (aOR2.57, p<0.0001).

**Conclusions:**

Transactional sex with main partners and casual partners in this population is high. Risks associated with both forms of transactional sex emphasize the ways in which men's controlling behaviours and women's experiences of lack of economic autonomy shape women's engagement in transactional sex. Reducing both forms of transactional sex requires interventions to empower women, including their economic autonomy.

# Sub article: TUPDD0305 Paying for sex and associated risks among young male pavement dwellers in Dhaka City, Bangladesh

**Published:** 2016-07-22

**Authors:** T McClair, T Hossain, N Sultana, E Yam, B Zieman, S Hossain, R Yasmin, N Sadiq

**Affiliations:** 1 Johns Hopkins Bloomberg School of Public Health, Baltimore, MD, United States; 2 Population Council, Dhaka, Bangladesh; 3 Population Council, Washington, DC, United States; 4 Marie Stopes Bangladesh, Dhaka, Bangladesh; 5 International HIV/AIDS Alliance, Dhaka, Bangladesh

## Abstract

**Introduction:**

Dhaka City is home to thousands of migrants from Bangladesh’s rural areas who live in the streets as “pavement dwellers.” Bangladesh development programmes often prioritize addressing girls’ substantial health needs, but young males also have vulnerabilities. This study assesses their practice of paying for sex, and its association with HIV-related risks.

**Methods:**

As part of the global Link Up project, trained interviewers recruited 447 male pavement dwellers from seven Dhaka City pavement dweller “hotspots.” At each hotspot, interviewers used random selection techniques (like spinning a bottle) to invite males aged 15 to 24 to participate in a survey that covered HIV-related risks and behaviours. We conducted descriptive analysis to examine socio-demographic characteristics, paying for sex (giving money, goods or services in exchange for sex in past year), sexually transmitted infection (STI) symptoms (past 6 months) and high-risk sex (unprotected last sex with non-primary partner). Among those who had ever had sex (N=321), we conducted multivariate logistic regression analysis to assess whether transactional sex was associated with STI symptoms or high-risk sex, controlling for socio-demographic characteristics and early sexual debut.

**Results:**

Median participant age was 18 years, 7% completed education above primary school and 98% reported earning any income, a median of US$76/month. Eighty-nine percent were never married and 4% were living with a parent/guardian. Seventy-two percent had ever had sex and 44% had early sexual debut (<age 15). Physical abuse was reported by 77% of participants and sexual abuse by 13%. Of those who had ever had sex, 80% had paid for sex, 52% engaged in high-risk sex, 79% had had symptoms consistent with STIs and 3% had ever received an HIV test. In multivariate analysis, those who had paid for sex had significantly increased odds of reporting recent STI-related symptoms (adjusted odds ratio (AOR)=1.75, 95% confidence interval (CI): 1.14–2.68), and had greater odds of engaging in high-risk sex (AOR=2.13, 95% CI: 1.48–3.08).

**Conclusions:**

Young, pavement dwelling males in Dhaka City have unique vulnerabilities. The adverse factors associated with paying for sex highlight the need for targeted programmes that promote condom use, STI screening/treatment and HIV testing in this population.

# Sub article: TUPDD0306 Love with HIV: a latent class analysis of intimate relationships among women living with HIV enrolled in Canada's largest multisite community-based research study

**Published:** 2016-07-22

**Authors:** A Carter, S Greene, C Hankins, LA Brotto, D Money, M Kestler, S Patterson, N O'Brien, K Salters, E Ding, K Webster, V Nicholson, M Sanchez, M Desbiens, D Dubuc, SY Lin, RS Hogg, A de Pokomandy, MR Loutfy, A Kaida, CHIWOS Research Team

**Affiliations:** 1 British Columbia Centre for Excellence in HIV/AIDS, Vancouver, Canada; 2 Simon Fraser University, Burnaby, Canada; 3 McMaster University, Hamilton, Canada; 4 University of Amsterdam, Amsterdam, Netherlands; 5 Department of Obstetrics and Gynecology, Faculty of Medicine, University of British Columbia, Vancouver, Canada; 6 BC Women's Hospital and Health Centre, Women's Health Research Institute, Vancouver, Canada; 7 Division of AIDS, Department of Medicine, University of British Columbia, Vancouver, Canada; 8 McGill University Health Centre, Montreal, Canada; 9 ViVA, Positive Living Society of BC, Vancouver, Canada; 10 Women's College Research Institute, Toronto, Canada

## Abstract

**Introduction:**

Quantitative studies traditionally reduce relationships to single-item variables and investigate sexual risk-taking. To broaden understanding of relationships and sexuality, we characterized types of intimate relationships among women living with HIV (WLWH) using multiple measures and examined differences in affection and associated psychosocial characteristics.

**Methods:**

Using a critical feminist approach, we analyzed questionnaire data for 1335 WLWH (≥16 years) in the multi-site, community-based Canadian HIV Women's Sexual and Reproductive Health Cohort Study. We conducted latent class analysis, incorporating eight indicators: marital status, duration, sex with regular partner in past 6-months, physical intimacy, emotional closeness, relationship power, exclusivity and couple HIV-serostatus. We assessed construct validity by examining prevalence of affection (*Someone to love and make you feel wanted*) and identified covariates using multinomial logistic regression.

**Results:**

We delineated five latent classes: no relationship (47%), relationship without sex (9%), and three types of sexual relationships – short-term/casual (16%), long-term/unhappy (7%) and long-term/happy (22%). Women in the latter two classes had high probabilities of reporting an exclusive married/common-law/living-apart relationship of ≥3-years duration relative to women in short-term/casual relationships, yet they diverged on contentment with physical intimacy (44% unhappy vs. 97% happy), emotional closeness (24% vs. 86%), power (43% vs. 82%), and couple HIV-serodiscordance (59% vs. 71%). Affection was most prevalent in long-term/happy relationships (64%) and relationships without sex (48%), compared to long-term/unhappy (39%), short-term/casual (37%) and no relationship (23%) (p<0.0001). Relative to no relationship: women >50 years were less likely to be in any relationship; women reporting sex work (AOR: 3.03(95% CI: 1.64, 5.61)) and violence (6.64 (3.33,13.26)) were more likely to be in short-term/casual relationships; women without depression (2.90 (2.04,4.12)) were more likely to be in long-term/happy relationships. No differences by gender, sexual orientation or ethnicity were observed.

**Conclusions:**

Nearly half of Canadian WLWH were not in relationships. Women's relationships were heterogeneous, though HIV serodiscordance was common and one-fifth reported long-term/happy and loving sexually active relationships. Sex, however, did not equate with affection, and relationships without sex had higher levels of love than some sexual relationships. A nuanced focus on promoting healthy relationships may offer a more comprehensive approach to supporting women's sexual well-being, particularly among older WLWH and those with experiences of sex work, violence and depression.

# Sub article: TUPDE0102 The effectiveness of a quality improvement collaborative to accelerate elimination of mother to child transmission (eMTCT): key outcomes and determinants from a demonstration phase collaborative implemented in South Africa, 2012–2015

**Published:** 2016-07-22

**Authors:** A Chirowodza, D Williams, C Diergaardt, O Adetokunboh, S Gede, N Gobodo, N Makeleni, N Tuswa, M Eckard, T O'rie, N Shingwenyana, B Green, I Oluwatimilehin

**Affiliations:** 1 South to South Programme for Comprehensive Family HIV Care and Treatment, Department of Paediatrics and Child Health, Faculty of Medicine and Health Sciences, Cape Town, South Africa; 2 Amathole District, Department of Health, East London, South Africa; 3 Pixley ka Seme District, Department of Health, De Aar, South Africa; 4 Cape Winelands District, Department of Health, Worcester, South Africa

## Abstract

**Introduction:**

South Africa has made substantial improvements in prevention of mother-to-child transmission (PMTCT) services in South Africa as demonstrated by a reduction in vertical transmission in the recent past. Challenges remain in health programme implementation for key antenatal and postnatal services. We describe impact and determinants for successful implementation of a quality improvement collaborative (QIC) approach as a method to accelerate the achievements of elimination of mother-to-child transmission (eMTCT) goals in South Africa.

**Methods:**

In partnership with the Department of Health, we implemented a QIC in 55 health facilities in three provinces over two phases: a pilot phase and a demonstration phase. Learning sessions and quality improvement (QI) projects were conducted focusing on key elements of the PMTCT cascade. QI teams received coaching and on-site training. To assess performance, we compiled control charts using PMTCT indicators from district health information system. The Wilcoxon signed-ranks test was used to test for significance in increases or decreases between pre-post intervention medians. Influencing factors such as facility level QI skills, organizational culture and facility type and location were measured to understand influencing factors.

**Results:**

We observed marked regional variation in improvement for early booking rates in two out of three provinces (24% in the Eastern Cape, p<0.001; 4% in the Western Cape). All sites improved antenatal HIV retest rates (31% in the Eastern Cape, p<0.001; 11% in the Northern Cape, p<0.001; 74% in the Western Cape, p<0.001). Postnatal visit within 6 days rates improved (varying from 6 to 15% in supported provinces). Exclusive breastfeeding rates improved (28% increase in the Eastern Cape, p<0.001; 15% in the Northern Cape, p<0.001; 12% in the Western Cape, p<0 001). The 18 month rapid test uptake rates improved for all provinces (Eastern Cape 28%; *p*<0.001, Northern Cape 20%; *p*<0.001 and Western Cape 25%; *p*<0.001). Factors influencing performance were baseline rates, facility type and size, quality improvement skills, leadership and buy in for quality improvement.

**Conclusions:**

The collaborative approach achieved rapid improvements in eMTCT programme outcomes in a wide range of facilities across South Africa. Performance variability may be attributed to contextual, organizational and system factors.

# Sub article: TUPDE0103 Continuous quality improvement for voluntary male medical circumcision training: experiences and results from the field

**Published:** 2016-07-22

**Authors:** D Jacobs, R Mabuse, T Maartens, F Dikgale, J Sithole, A Thambinayagam, J Ndirangu

**Affiliations:** 1 Quality & Performance Institute, University Research Co., LLC, Pretoria, South Africa; 2 USAID ASSIST Project, University Research Co., LLC, Pretoria, South Africa; 3 USAID Southern Africa, Pretoria, South Africa

## Abstract

**Introduction:**

Continuous quality improvement (CQI) is a deliberate and a systematic process designed to intervene in issues of quality in voluntary medical male circumcision (VMMC) using eight WHO quality standards. University Research Company (URC) conducted VMMC baseline assessment at 134 Department of Health (DoH) facilities where implementing partners (IPs) are providing this service. Following this assessment, gaps were identified which revealed non-compliance of sites to different areas of WHO quality standards. URC saw the need to train those who manages this service at a programmatic level as well as those at site level to improve the quality of service provided as well as to reduce adverse events. A CQI training exercise empowers the providers not only to identify gaps and come up with improvement strategies, but also to assess themselves, identify bottlenecks and shortfalls and improve going forward.

**Description:**

MMC baseline assessment was conducted in eight provinces: Mpumalanga (MP); Eastern Cape (EC); Gauteng (GP); Kwazulu Natal (KZN); Limpopo (LP); Northern Cape (NC) and North West (NW) to look at compliance of sites to WHO quality standards. Three days training was then conducted by URC staff to empower the providers so that they can provide good quality service. In total 279 healthcare workers were trained across the eight provinces. Post training, two subsequent assessments were then conducted to assess improvement in the quality of service and compliance with the standards.

**Lessons learned:**

Post CQI training results shows that provinces improved significantly on average from 76.4% (baseline) to 92.8% (reassessment). Individual provinces scored as follows: EC (81.4 to 96%), GP (78.2 to 97.3%), KZN (80.4 to 94.2%), LP (81.4 to 98.5%), MP (63.4 to 90.1%), NC (69.2 to 94.9%) and NW (78.0 to 80.6%).

**Conclusions/Next steps:**

CQI training has empowered those providing the MMC service with skills, knowledge and understanding of the programme to enable them to provide safe, high-quality MMC service. Results after training showed that provinces improved significantly in their performance as far as providing quality service is concerned.

# Sub article: TUPDE0104 Effects of continuous quality improvement as a tool for inspiration amongst health care workers and HIV+ mothers on rates of HIV and malnutrition amongst HIV-exposed infants in rural Rwanda

**Published:** 2016-07-22

**Authors:** W Leonard, D Uwamahoro, T Uwacu, A Ishimwe, A Mutaganzwa, E Chang, JD Ngirabega

**Affiliations:** 1 The Ihangane Project, Aptos, CA, United States; 2 Ruli District Hospital, Ruli, Rwanda; 3 Center for AIDS Research and Education, University of California, Los Angeles, CA, United States; 4 East African Health and Research Commission, Arusha, United Republic of Tanzania

## Abstract

**Introduction:**

Frontline health care workers are most effective when they feel valued, capable and optimistic about the future. The Ihangane Project and Ruli District Hospital, serving 200,000 people in the Northern Province of Rwanda, created a Continuous Quality Improvement (CQI) programme that promotes these principles amongst health care workers and HIV+ mothers as a key strategy to increase adoption of prevention of mother-to-child transmission (PMTCT) protocols and ultimately eliminate mother to child HIV transmission and dramatically decrease malnutrition amongst HIV-exposed infants.

**Description:**

Health care workers and HIV+ mothers at seven health centres associated with Ruli District Hospital designed and implemented a CQI programme that builds trust, fosters capacity and utilizes trends to demonstrate linkages between care quality and durable good health outcomes. Our approach focuses on five pillars of quality care: Clinical Care, Mother-Centred Systems, Data Management, Logistics and Health Education. Pillars are assessed using an Observational Check List (OCL) every four months, followed by a collaborative meeting to review results. Health care workers identify areas of strength and weakness in current practices and consider interventions for improvement. In collaboration with HIV+ mothers, they design and implement improvements in their system of care.

**Lessons learned:**

From March 2013 through December 2015, 332 HIV-exposed infants and 302 HIV+ mothers enrolled in the health centres’ PMTCT programmes. Quality across all pillars of care increased by 118% (39 to 85%). Nurses’ ability to accurately diagnose malnutrition increased by 74% (46 to 80%), and infant HIV testing at appropriate intervals increased by 800% (from 8 to 72%). Improvement in care quality contributed to a 66% (41 to 14%) decrease in acute (underweight) malnutrition and a 62% (61 to 23%) decrease in chronic (stunting) malnutrition amongst HIV-exposed infants, and a 63% (1.6 to 0.6%) decrease in the rate of mother-to-child HIV transmission.

**Conclusions/Next steps:**

Good health outcomes are possible even in extremely resource-limited settings. A continuous quality improvement programme that enables health care workers and their patients to improve their systems of care and connects actions to good health outcomes is a cost-effective approach to building effective and resilient health systems that can reach and sustain health goals.

# Sub article: TUPDE0105 Improving male partner testing in PMTCT: a quality improvement initiative in Kinango Hospital, Kwale County, Kenya

**Published:** 2016-07-22

**Authors:** D Mwakangalu, M Nzaro, Y Mwamzandi, BS Mwero, J Makori, R Mlongo, A Bunu

**Affiliations:** 1 Pathfinder International, Mombasa, Kenya; 2 Pathfinder International, Nairobi, Kenya; 3 Kenya Ministry of Health-Kwale County, Nairobi, Kenya

## Abstract

**Introduction:**

HIV testing for pregnant women during the antenatal period has been successful across Kenya with 92% of ANC mothers having their HIV status determined (KAIS 2013). Despite this success, couple testing has remained low despite intensive health education. HIV-infected male partners with unknown HIV status pose a number of risks including infecting their HIV-negative pregnant partners and potentially increasing the risk of mother-to-child transmission. Testing couples together removes burden of disclosure from HIV-infected partners and improves adherence, retention in care and enhanced partner support.

**Description:**

To address low couples testing for HIV, the USAID-funded APHIAplus Nairobi-Coast project, led by Pathfinder International, supported Kinango Hospital's quality improvement team to conduct a root cause analysis and implement a quality improvement “change idea” to improve couple testing at the hospital MCH Department. From August 2014, pregnant women who presented to the MCH were issued letters inviting their partners to accompany them to the facility for ANC visits. The project ensured community awareness of the initiative through mentions during morning health talks and individual counselling sessions. Male partners were offered rapid HIV testing as couples according to the national guidelines. Data were captured on routine MOH data collection tools.

**Lessons learned:**

Pregnant women attending first ANC visit and who had their male partners tested for HIV increased from 4 (1%) between August 2013 and July 2014 to 336 (84%) between August 2014 and July 2015. A total of 39 pregnant women and 17 males tested positive and were provided assisted disclosure and enrolled in HIV care. Twenty-six of the 27 infants exposed to HIV tested negative through early infant diagnosis testing by DNA PCR. Seven mother-baby pairs were referred to other facilities, and four were lost to follow-up.

**Conclusions/Next steps:**

Couple testing for HIV can be successfully integrated with ANC visits by inviting partners to attend ANC visits. This approach can help improve uptake of HIV testing and counselling among male partners, help with disclosure and improve MTCT rates. Using existing quality improvement teams, change ideas like this one can be successfully replicated.

# Sub article: TUPDE0106 Increasing linkage to HIV care for newly diagnosed HIV-infected persons through quality improvement approach in urban slums in Kenya

**Published:** 2016-07-22

**Authors:** S Kegoli, A Njoroge, J Motoku, C Muriithi, I Mutisya

**Affiliations:** 1 Prevention, Eastern Deanery AIDS Relief Program, Nairobi, Kenya; 2 Eastern Deanery AIDS Relief Program, Nairobi, Kenya; 3 Care and Treatment, Eastern Deanery AIDS Relief Program, Nairobi, Kenya; 4 HMIS, Eastern Deanery AIDS Relief Program, Nairobi, Kenya; 5 Division of Global HIV/AIDS, U.S. Centers for Disease Control and Prevention, Nairobi, Kenya

## Abstract

**Introduction:**

Kenya AIDS strategic framework targets to identify 90% of people living with HIV (PLHIV), provide treatment to 90% while ensure 90% on ART achieve virological suppression. Despite scale up HIV testing coverage, only 60% and 31% of PLHIV are linked to care and ART, respectively. Pre-enrolment loss to follow-up and delayed linkage to care are associated with increased morbidity and mortality.

Eastern Deanery AIDS Relief Program has 14 facilities within 95 slums in Nairobi, serving 2,157,690 people. Despite scaled up HIV testing in facilities and community, enrolment into care, remained low necessitating innovative strategies to address the challenge. The program utilized the Kenya HIV quality improvement framework guidance to improve linkages to care.

**Description:**

A Continuous Quality improvement (CQI) activity was initiated involving Plan-Do-Study-Act (PDSA) cycles between October 2012 and September 2015. The goal was to improve linkage to care of newly diagnosed PLHIV from 60% to >95%. PDSA cycle results were reviewed quarterly and lessons learnt summarized, effective strategies retained and new ones adopted where necessary.

**Lessons learned:**

PDSA cycle one in October 2012 involved use of patient escorts by counsellors which led to increased linkage from 60 to 80.8% by June 2012. PDSA cycle two in September 2012 introduced linkage registers for counsellors to track intra-facility linkages in addition to patient escorts and telephone calls, and home visits for patients not linked on the testing day improved linkages from 74.2 to 91.8% by March 2013. PDSA cycle three in October 2013 and combined patient escorts, linkage register and training of counsellors within quality improvement teams. At the end of PDSA cycle three, linkages improved and were sustained at 98% by September 2015.

**Conclusions/Next steps:**

Successful linkages to HIV care and treatment services may be achieved through adoption of multiple strategies that are feasible and affordable such as use of patient escort, improved HCW skills and use of linkage registers.

**Abstract TUPDE0106–Graph 1:** Trends of improved linkages to care of newly diagnosed HIV infected persons between Oct 2012 and Sept 2015.

# Sub article: WEPDA0101 Novel mode of recognition of the glycan-V1V2 region of HIV-1 envelope by a new lineage of broadly neutralizing antibodies

**Published:** 2016-07-22

**Authors:** E Cale, J Gorman, N Radakovich, G Ozorowski, E Crooks, K Osawa, M Asokan, N Doria-Rose, A Ward, P Kwong, J Binley, J Mascola

**Affiliations:** 1 Vaccine Research Center, NIAID, National Institutes of Health, Bethesda, MD, United States; 2 The Scripps Research Institute, La Jolla, CA, United States; 3 San Diego Biomedical Research Institute, San Diego, CA, United States

## Abstract

**Introduction:**

Studies of naturally occurring broadly neutralizing antibodies (bnAbs) have yielded valuable clues for HIV-1 vaccine design. To date, bnAbs targeting the glycan-V1V2 region of HIV-1 Env have been cloned from only four individuals. These bnAbs have an unusually long heavy chain complementarity-determining region 3 (CDRH3) that penetrates the glycan shield to access the underlying epitope. We isolated a new lineage of glycan-V1V2 bnAbs, N90-VRC38, that has more conventional immunogenetic qualities, and we determined how this lineage is able to target the glycan-V1V2 epitope with a shorter CDRH3 loop.

**Methods:**

HIV-specific B cells from the clade B virus-infected NIAID Donor N90 were sorted by flow cytometry using Env-bearing virus-like particles or soluble trimers as HIV-specific probes. Antibody heavy and light chains were recovered by RT-PCR, expressed and purified, and evaluated for multiclade neutralization activity using the TZM-bl neutralization assay. Two structures of mAb N90-VRC38.01, one complexed with BG505 SOSIP trimer and another complexed with a WITO.33 V1V2 scaffold, were solved by negative-stain electron microscopy (EM) and by X-ray crystallography, respectively. Binding stoichiometry of the antibody was determined by native PAGE and by biolayer interferometry (BLI) under saturating conditions.

**Results:**

The broadest of the 11-member N90-VRC38 clonal lineage, N90-VRC38.01, neutralized 29% of 208 Env-pseudotyped viruses. This antibody has a neutral, short 18 amino acid (AA) CDRH3; in contrast, other glycan-V1V2 bnAbs exhibit long (>26 AA), negatively charged CDRH3s. N90-VRC38.01 uses both its CDRH3 and CDRL1 to make hydrogen bonds with side chains of strands A, B and C of the V1V2 region. This is unlike other glycan-V1V2 bnAbs, which make main-chain protein-protein contacts with strand C of the epitope.

**Conclusions:**

The N90-VRC38 lineage represents a new type of glycan-V1V2-binding bnAb that binds via a CDRH3 loop that is far shorter than those of other V1V2-directed bnAbs. This raises the possibility that eliciting V1V2-specific bnAbs by vaccination may be more readily achievable than previously thought, as antibodies with such shorter CDRH3 loops are common in the human IgG repertoire.

# Sub article: WEPDA0102 Non-classical CD8^+^ T cells restricted by HLA class II emerge in HIV infection and show antiviral efficacy

**Published:** 2016-07-22

**Authors:** S Ranasinghe, P Lamothe-Molina, D Soghoian, S Kaizer, M Cole, A Shalek, N Yosef, B Jones, M Carrington, H Streeck, D Kaufmann, L Picker, J Kappler, BD Walker

**Affiliations:** 1 Ragon Institute of MGH, MIT and Harvard, Cambridge, MA, United States; 2 University of California, Berkeley, CA, United States; 3 Cancer and Inflammation Program, NCI, Frederick, MD, United States; 4 Institute for Medical Biology, Essen, Germany; 5 Centre de Recherche du Centre hospitalier de l'Université de Montréal, Montreal, Canada; 6 Oregon Health Sciences University, Portland, OR, United States; 7 National Jewish Health, Denver, CO, United States; 8 Howard Hughes Medical Institute, Chevy Chase, MD, United States

## Abstract

**Introduction:**

CD8+ T cells typically recognize infected cells through viral peptides presented on HLA class I. However, CD8+ T cell responses restricted by HLA class II have also been reported in CD4 knockout mice and in a macaque AIDS vaccine model, where Gag-specific CD8+ T cells restricted by class II were elicited by CMV vector immunization. This raises a critical question: do class II-restricted CD8+ T cell responses exist in natural HIV-infection?

**Methods:**

We detected class II-restricted CD8+ T cell populations in 3 of 101 treatment-naive HIV-infected individuals screened using a novel “CD8 HLA-DR” IFNy ELISpot assay. CD8+ T cells targeted HIV Gag37 or Gag41 peptides presented by LCL stably expressing recombinant DR01 and DR11. Antibody blocking of class I and II, and flow cytometric staining with class II tetramers confirmed the restriction. TCR sequencing was conducted from class II tetramer-sorted cells.

**Results:**

Our detailed analysis demonstrates the existence of Gag-specific CD8+ T cell responses restricted by HLA-DR, which exhibit potent cytolytic functions that are comparable to class I-restricted cytotoxic T lymphocytes. The HLA-DR-restricted CD8+ T cells were Perforin+GranzymeB+ and efficiently lysed HIV-infected autologous CD4+ T cells and macrophages (p<0.01). Although these responses are rare, detected in only 3% of HIV controllers screened, in one individual it was the most immunodominant CD8+ response encompassing 12% of total CD8+ T cells directly *ex vivo* (in the absence of T cell expansion). Moreover, these HLA-DR-restricted CD8^+^ T cells showed a typical patterns of TCR usage that were characterized by two different co-expressed TCR alpha chains, and intriguingly, a single TCR beta clonotype that was shared with CD4^+^ T cells targeting the same peptide-HLA class II complex. Indeed, TCR beta clonotype TRBV2 was shared between 100% of CD8+ and 73.9% of CD4+ targeting the DR11-Gag41 complex, with 2/27 sequences identical in VDJ and CDR3 motifs.

**Conclusions:**

These data not only reveal the presence of atypical CD8+ T cells governed by unusual TCR cross-reactivity in HIV infection, but may also have relevance for future CMV vector-based HIV vaccines as they move into Phase 1 trials in humans, where their ability to induce unconventional CD8 T cells remains unknown.

# Sub article: WEPDA0103 Presence of HIV-1C broadly neutralizing antibodies in pregnancy and at delivery

**Published:** 2016-07-22

**Authors:** T Mduluza, S Dzoro, K Bedi, WS Mpoloka

**Affiliations:** 1 Biochemistry, University of Zimbabwe, Harare, Zimbabwe; 2 School of Laboratory Medicine and Medical Sciences, University of KwaZulu-Natal, Durban, South Africa; 3 Biological Sciences, University of Botswana, Gaborone, Botswana; 4 Botswana AIDS Institute Partnership, Research Laboratory, Gaborone, Botswana

## Abstract

**Introduction:**

HIV neutralizing antibody assays are now being widely employed in different laboratories in search for correlates of protective immunity. There are strong arguments in favour of a beneficial role of some broadly neutralizing antibodies in prevention of HIV infection if these antibodies exist prior to exposure. The neutralizing antibodies could be useful in immunotherapy.

**Methods:**

Archived plasma samples collected from 54 mothers at recruitment, before and after delivery from a PMTCT study were assayed for presence of neutralizing antibodies using TZM-bl cells. The participating mothers were divided into HIV transmitters and non-transmitter, indicated at delivery. The virus panel comprised of reference strains (92TH021 and MBA2’ – clade AE; 92BR020 – clade B; and 93IN905, ZA012, ZM651 – clade C, and results were expressed as the plasma inhibition dilution causing 50% or 90% reduction in viral replication.

**Results:**

Plasma samples were from 21 non-transmitters and 33 transmitters. Non-transmitters, compared to the transmitting mothers, had 332 CD4+ cells/µl and 103 024 HIV RNA copies/ml compared to 321 CD4+ cells/µl and 158 164 HIV RNA copies/ml, respectively. Broadly neutralizing antibodies were present in baseline plasma from both HIV-1 transmitters and non-transmitters. When a more stringent cut off value for the neutralizing activity was applied, a decline in the potency and breadth of neutralization was observed in only two plasmas showing high 90% infection inhibition of three out of seven strains. Potent neutralizing antibodies to the South African strain (ZA012) were more frequently detected among the non-transmitting mothers. Neutralization titres were significantly higher in non-transmitters for 50% inhibition of the subtype C strains, 93IN905 (p<0.001) and ZM651 (p=0.014), and for 90% inhibition of 92TH021, MBA2’, 92BR020 and 93IN905 (p<0.001), ZM651 (p=0.006).

**Conclusions:**

Despite an overall reduced potency at 90% infection inhibition, non-transmitting mothers had significantly higher potency and breadth (cross-clade neutralization) for 90% viral inhibition at delivery compared to the transmitting mothers. These results provide evidence of the presence of HIV-1 broadly cross-neutralizing antibodies during pregnancy and at delivery that directly could be utilized in the management of HIV infection hence contribute to reduced mother to child transmission.

# Sub article: WEPDA0104 PML/TRIM19-dependent inhibition of retroviral reverse-transcription by Daxx

**Published:** 2016-07-22

**Authors:** J Dutrieux, G Maarifi, D Portilho, N Arhel, M Chelbi-Alix, S Nisole

**Affiliations:** 1 Institut National de la Santé et de la Recherche Médicale (INSERM), UMR-S 1124, Paris, France; 2 Institut National de la Santé et de la Recherche Médicale (INSERM), U941, University Institute of Hematology, Saint-Louis Hospital, Paris, France

## Abstract

**Introduction:**

Promyelocytic leukemia protein (PML), also known as TRIM19, belongs to the family of tripartite motif (TRIM) proteins. PML is mainly expressed in the nucleus, where it forms dynamic structures known as PML nuclear bodies that recruit many other proteins, such as Sp100 and Daxx. While the role of PML/TRIM19 in antiviral defence is well documented, its effect on HIV-1 infection remains unclear.

**Methods:**

HeLa or MEF cells derived from wt or PML KO mice were transduced with GFP-encoding vectors derived from HIV-1, SIV, EIAV and MLV. Human T lymphocytes were infected with HIV-1. PML knockdown was performed using siRNA, shRNA or by inducing its degradation by arsenic. Retroviral transductions were quantified by flow cytometry and qPCR whereas HIV-1 propagation in T-cells was followed by ELISA. Localization of PML, Daxx and HIV-1 capsid protein was determined by immunofluorescence and *in situ* proximity ligation assay. Retro-transposition events were estimated by the number of G418R foci after transfection of HeLa cells (over-expressing Daxx or not) with neo-marked retrotransposons.

**Results:**

Infection by HIV-1 and other retroviruses triggers the formation of PML cytoplasmic bodies, as early as 30 minutes post-infection. Quantification of PML cytoplasmic bodies revealed that they last approximately 8 hour, with a peak at 2 hour, post-infection. PML re-localization is blocked by reverse-transcription inhibitors and is not observed following infection with unrelated viruses, suggesting it is specifically triggered by retroviral reverse-transcription. Furthermore, PML knockdown dramatically increases reverse-transcription efficiency. However, although it is required for retroviral restriction, PML does not inhibit directly retroviral infection, but acts through the stabilization of one of its well-characterized partners, Daxx. In the presence of PML, cytoplasmic Daxx is found in the vicinity of incoming HIV-1 capsids and inhibits reverse-transcription whereas in the absence of PML, Daxx is degraded. Interestingly, Daxx not only interferes with exogenous retroviral infections but can also inhibit retro-transposition of endogenous retroviruses.

**Conclusions:**

We show for the first time that PML and Daxx cooperatively interfere with an early step of retroviral infection by targeting the reverse-transcription step. Our findings unravel a novel antiviral function for PML, and its nuclear body-associated protein Daxx as a broad cellular inhibitor of reverse-transcription.

# Sub article: WEPDA0105 Human sPD1-based HIV-1 gag-fusion DNA vaccine induces high frequency of broadly reactive T cell responses in mice and rhesus macaques

**Published:** 2016-07-22

**Authors:** S Chen, J Tang, Z Chen

**Affiliations:** Microbiology, University of Hong Kong, Hong Kong

## Abstract

**Introduction:**

Ongoing AIDS epidemic commonly involves diverse HIV-1 subtypes even in a single geographical location (e.g. Hong Kong). Therefore, vaccine-induced host immunity should be broadly reactive for protection. We have previously reported a vaccine strategy that can induce potent cellular immunity by fusing murine soluble programmed death-1 (sPD1) with HIV-1 Gag-p24. Using this strategy, we further investigated a novel human sPD1-based HIV-1 Gag-fusion DNA vaccine in mice and rhesus macaques.

**Methods:**

The mosaic-like immunogen design was based on hundreds of Gag sequences covering three major circulating HIV-1 subtypes B/B’, C/CB’ and 01_AE in China. The novel DNA vaccine contained human sPD1 fused together with two Gag, thus achieving the epitope coverage about 97% of all three subtypes according to the HIV database analysis. This vaccine was evaluated through *in vivo* electroporation in mice and rhesus macaques. The immunogenicity profiles were determined using ELISA, ELISpot, ICS and Tetramer assays. The vaccine-induced protection was also investigated in immunized mice challenged with a replicating-competent EcoHIV.

**Results:**

*In vitro* analysis confirmed the design and expression of the fusion immunogen, which was also able to interact with both human and murine PD-L1/L2. *In vivo* experiments indicated that the novel vaccine not only induced potent T cell immune responses similar to murine sPD1-p24_fc_ as we previously published but also had an enhanced breadth across three subtypes. Moreover, vaccine-induced Gag-specific CD8^+^ T cells conferred significant protection against EcoHIV infection in mice. Notably, in rhesus macaques vaccine-induced T cell responses were broadly reactive and comparable to that elicited by a heterologous vaccinia prime and ad5 boost regimen.

**Conclusions:**

We found that the human sPD1-based HIV-1 Gag-fusion DNA vaccines are highly immunogenic in two animal species, and confers substantial protection against EcoHIV-1 infection in mice. The immunogenicity of our vaccine in rhesus macaques is promising and may warrant future development for human use.

# Sub article: WEPDB0101 Deferred antiretroviral therapy is associated with lower estimated glomerular filtration rate in HIV-positive individuals with high CD4 counts

**Published:** 2016-07-22

**Authors:** A Mocroft, AC Achra, M Ross, L Ryom, A Avihingsanon, E Bakowska, W Belloso, A Clarke, H Furrer, G Lucas, M Ristola, MS Rassool, J Ross, C Somboonwit, C Wyatt, On behalf of the INSIGHT and START Study Group

**Affiliations:** 1 University College London, London, United Kingdom; 2 University of New South Wales, Sydney, Australia; 3 Icahn School of Medicine at Mount Sinai, New York, NY, United States; 4 University of Copenhagen, Copenhagen, Denmark; 5 Chulalongkorn University, Bangkok, Thailand; 6 Wojewodzki Szpital Zakazny, Warsaw, Poland; 7 Hospital Italiano de Buenos Aires, Buenos Aires, Argentina; 8 Royal Sussex County Hospital, Brighton, United Kingdom; 9 Bern University Hospital, University of Bern, Bern, Switzerland; 10 Johns Hopkins University of Medicine, Baltimore, MD, United States; 11 Helsinki University Central Hospital, Helsinki, Finland; 12 University of Witwatersrand, Johannesburg, South Africa; 13 University Hospitals Birmingham NHS Foundation Trust, Birmingham, United Kingdom; 14 University of South Florida, Tampa, FL, United States

## Abstract

**Introduction:**

The impact of antiretroviral therapy (ART) on renal function in HIV-positive persons with high CD4 is largely unknown. We evaluated changes in estimated glomerular filtration rate (eGFR) among participants randomized to immediate or deferred ART within the INSIGHT START trial.

**Methods:**

eGFR was calculated from locally measured creatinine using MDRD and CKD-EPI at months 4, 8, 12 and annually. Participants with baseline and ≥1 follow-up eGFR were included. We analyzed change in eGFR at each visit from baseline using random effects models.

**Results:**

4629 of 4685 START participants (99%) were included; characteristics were balanced between the immediate (n=2294) and deferred ART arms (n=2335). In both arms, median baseline CD4 was 651/mm^3^ and eGFR (CKD-EPI) 111 ml/min/1.73 m^2^. Mean follow-up was 2.6 years. ART was initiated in 2271 participants (99.0%) in the immediate and 1126 (48.2%) in the deferred arm, accounting for 94 and 28% of follow-up time, respectively. 89% of initial regimens in both arms included TDF. In the primary randomized comparison those in the deferred arm had a lower eGFR over follow-up (Table) with no evidence that the eGFR slope was different comparing the immediate and deferred arms (p>0.2). The lower mean eGFR in the deferred arm remained significant in secondary analyses (Table). In a model adjusted for time and baseline eGFR, the mean change in eGFR (CKD-EPI) in the deferred versus immediate arm in those of non-black zand black race was 0.23 (95% CI:−0.42, 0.87) and −2.43 (95% CI: −3.42, −1.43; p<0.0001 test for interaction), respectively.

**Conclusions:**

Deferring ART initiation in patients with high CD4 led to a small but significantly lower eGFR compared to those starting immediately, and was most pronounced in those of black race. These results suggest asymptomatic HIV infection may promote kidney disease despite preserved immune function.

**Abstract WEPDB0101–Table 1:** Mean change in eGFR by randomization arm in INSIGHT START trial

Outcome	Model 1* Deferred arm (vs. immediate arm) (95% CI), P	Adjusted Model 2** Deferred arm (vs. immediate arm) (95% CI), P	Adjusted Model 3*** Deferred arm (vs. immediate arm) (95% Cl), P
eGFR-CKD-EPI	−0.56 (−1.11 to −0.003), 0.049	−1.85 (−2.50 to −1.21), <0.001	−1.72 (−2.34 to −1.11), <0,001
eGFR-MDRD	−1.26 (−2.14 to −0.38), 0.005	−3.43 (−4.51 to −2.35), <0.001	−3.21 (−4.25 to −2.17), <0.001

* Model 1: adjusted for baseline eGFR and years since randomization.

** Model 2: additionally adjusted for current receipt of TDF and boosted PI.

*** Model 3: Model 2 + additionally adjusted for age, gender, race, region of enrollment, time since HIV diagnosis, use of injecting drugs, CD4, viral load, proteinuria, body mass index, hepatitis B/C, diabetes, hypertension, dyslipidaemia, cardiovascular disease, smoking status, ACE inhibitors or NSAIDS, all measured at randomization.

# Sub article: WEPDB0102 The effect of HIV infection on the age at presentation of HBV-driven hepatocellular carcinoma in South Africa

**Published:** 2016-07-22

**Authors:** T Maponga, H Vermeulen, B Robertson, S Burmeister, W Preiser, M Kew, M Andersson

**Affiliations:** 1 Division of Medical Virology, University of Stellenbosch, Cape Town, South Africa; 2 Radiation Oncology, Tygerberg Hospital, Cape Town, South Africa; 3 Department of Radiation Medicine, University of Cape Town, Cape Town, South Africa; 4 Department of Surgery, University of Cape Town, Cape Town, South Africa; 5 Department of Medicine, Groote Schuur Hospital & University of Cape Town, Cape Town, South Africa

## Abstract

**Introduction:**

Hepatocellular carcinoma (HCC) is the third most common cause of cancer mortality worldwide. Over 60% of HCC cases arise from chronic infection with HBV and/or HCV. Although HIV is known to impact on the natural progression of HBV infection, its impact on the epidemiology of HCC is not completely understood. This study investigated the occurrence of HIV among a cohort of patients incidentally diagnosed with HCC at four hospitals in South Africa.

**Methods:**

A total of 107 patients diagnosed with HCC were recruited at Tygerberg and Groote Schuur hospitals in the Western Cape and at Chris Hani Baragwanath and Charlotte Maxeke hospitals in Gauteng, South Africa following informed consent. Study subjects were recruited between December 2012 and October 2015. Demographic, laboratory and clinical data together with blood specimens were collected. When unknown at the time of diagnosis, patients were tested for HBsAg, HBeAg and HIV on the Abbott Architect.

**Results:**

Of 107 recruited HCC cases, 68/106 (64.1%) were positive for HBsAg. HIV seropositivity was seen in 22/100 (22%) of all HCC cases. HBeAg was seen in 10/17 (59%) of HIV-infected compared to 9/46 (20%) among HBV-monoinfected cases, p=0.005. Among HBsAg-positive HCC cases, 19/66 (29%) were HIV-infected compared to only 3/34 (9%) among those that were HBsAg-negative, p=0.04. The proportion of females among the HBV/HIV co-infected HCC cases of 6/18 (33%) was significantly higher it was among those that were HBV-monoinfected 6/47 (13%), p=0.005. HIV/HBV co-infected females presented younger, at median age 37.0 years (range: 30–44) compared to 50 years (range: 24–83) in HBV-monoinfected women, p=0.08.

**Conclusions:**

There is a high prevalence of HIV and HBV co-infection among HCC patients in South Africa. There is a trend towards younger age at diagnosis of HCC among HIV-positive women compared to those who are HIV-negative. Larger multi-centred studies are needed to more accurately evaluate the impact of HIV infection on the epidemiology of HCC among sub-Saharan populations where HIV is highly prevalent and HBV-driven HCC is common.

# Sub article: WEPDB0103 Antiretroviral treatment adherence, viremia, and psychiatric diagnosis throughout adolescence among perinatally HIV-infected youth

**Published:** 2016-07-22

**Authors:** A Bucek, C-S Leu, S Benson, P Warne, E Abrams, KS Elkington, C Dolezal, A Wiznia, CA Mellins

**Affiliations:** 1 HIV Center for Clinical and Behavioral Studies, Columbia University, New York, NY, United States; 2 ICAP, Columbia University, Mailman School of Public Health, New York, NY, United States; 3 Jacobi Medical Center, Albert Einstein College of Medicine, Bronx, NY, United States

## Abstract

**Introduction:**

Adolescence is associated with suboptimal medication adherence and is a time of increased risk for mental health and substance use disorders (SUD) that strongly predict antiretroviral treatment (ART) non-adherence in adults. Few studies of perinatally HIV-infected (PHIV+) youth have examined the relationships of psychiatric disorders to adherence and viral load (VL), longitudinally. This study utilized a diagnostic measure of psychiatric disorders including SUD to investigate the association of disorder with ART adherence and viremia as PHIV+ youth age.

**Methods:**

We analyzed data from three follow-up interviews (FU2-4, N=179) spanning 2.7 years of a longitudinal study of PHIV+ youth (13–24 years at FU2; 51% female; 67% African-American/Black) in New York City. At FU2 and FU4, six categories of psychiatric disorder (anxiety, behaviour, mood, SUD, any disorder, any disorder excluding SUD) were assessed with the Diagnostic Interview Schedule for Children. At each interview, participants reported on missed doses within the past week and 3 VL results ±90 days from the interview were abstracted from medical charts.

Multiple logistic regression analyzed cross-sectional associations, at FU2 and FU4, between psychiatric disorders and two outcomes: 1) missed doses and 2) most recent VL>1000. Multiple linear regression analyzed the relationship between FU2 psychiatric disorder and proportion of VL tests >1000 across FU2-4. Analyses adjusted for age and sex.

**Results:**

At FU2, 53% of youth had *any disorder*, 35% missed doses in the past week, and 47% had a VL>1000.

Cross-sectionally, at FU2, *behavioural disorder* was associated with missed dose (p=0.009) and VL>1000 (p=0.019) and *mood disorder* was associated with missed dose (p=0.041). At FU4, *behavioural disorder* was associated with missed dose (p=0.009). *Behavioural disorder* (p=0.041), *SUD* (p=0.016), and *any disorder* (p=0.008) at FU2 were significantly associated with higher proportion of VLs >1000 across FU2-4. Other associations were not significant (p>0.05).

**Conclusions:**

This is the first study to identify that adolescent psychiatric diagnoses were concurrently associated with poor adherence and prospectively associated with viremia over time. Psychiatric disorders in adolescence may predict viremia over the next 2–3 years. Assessment and treatment of psychiatric and substance abuse problems may be critical to improving adherence and preventing poor health outcomes during this vulnerable stage.

# Sub article: WEPDB0104 HIV associated neurocognitive disorder in a peri-urban HIV clinic in KwaZulu-Natal, South Africa

**Published:** 2016-07-22

**Authors:** JC Mogambery, H Dawood, D Wilson, A Moodley

**Affiliations:** 1 Infectious Diseases Unit, Department of Internal Medicine, Infectious Diseases, Grey's Hospital, Pietermaritzburg, South Africa; 2 Department of Internal Medicine, University of KwaZulu Natal, Pietermaritzburg, South Africa; 3 CAPRISA, University of KwaZulu-Natal, Durban, South Africa; 4 Department of Internal Medicine, Edendale Hospital, Pietermaritzburg, South Africa; 5 Department of Neurology, Grey's Hospital, Pietermaritzburg, South Africa; 6 Department of Neurology, University of KwaZulu-Natal (UKZN), Pietermaritzburg, South Africa

## Abstract

**Introduction:**

The prevalence of HIV associated neurocognitive disorder (HAND) in KwaZulu-Natal has not been established. This prospective, cross-sectional study determined the prevalence of HAND in ART nave patients attending a peri-urban HIV clinic. The impact of HAND on functional capacity and factors associated with HAND were examined. Alternate neurocognitive tools were tested against the international HIV dementia scale (IHDS) score. An association between HAND and non-adherence to ART was explored.

**Methods:**

Between May 2014 and May 2015, 146 ART nave outpatients were assessed prior to commencing ART electively. HAND was diagnosed using an IHDS score ≤10. Functional capacity was assessed using the eastern cooperative oncology group (ECOG) score. The get-up-and-go test and centre for epidemiological studies depression scale-revised (CESD-R) were performed at the same consultation and correlation between these two tests and IHDS was determined. A HIV viral load done 6 months after initiating ART was used as a surrogate marker for adherence to ART.

**Results:**

The prevalence of HAND determined by the IHDS was 78/146 (53%). ECOG score was 0 in 99.9% of patients with HAND. CD4 count ≤ 200 cells/mm^3^ (p=0.17) and alcohol consumption (p=0.17) were not associated with HAND. There was no correlation between the get-up-and-go test, CESD-R and the IHDS score. Of the 129/146 patients with 6 month viral loads assays a detectable viral load was found in 24/69(35%) with HAND and 12/60(20%) without HAND. There was no significant association between HAND and a detectable viral load after 6 months of ART use (p=0.06).

**Conclusions:**

Whilst the prevalence of HAND was high, it was not associated with impaired functional capacity. This finding suggests that early asymptomatic disease was prevalent in this population. A low CD4 count was not associated with HAND. The get-up-and-go-test and CESD-R were not useful in the diagnosis of HAND. A further study is needed to determine whether a relationship between HAND and non-adherence to ART exists.

# Sub article: WEPDB0105 Ageing and associated morbidity in HIV-positive persons in the Cohort of the Spanish AIDS Research Network (CoRIS)

**Published:** 2016-07-22

**Authors:** B Alejos, V Hernando, J del Amo, R Rubio, M Montero, M Rivero, J Hernández-Quero, R Muga, F Gutierrez, S Moreno, Coris

**Affiliations:** 1 Institute of health Carlos III, Madrid, Spain; 2 Hospital Doce de Octubre, Madrid, Spain; 3 Hospital de La Fe, Valencia, Spain; 4 Hospital de Navarra, Pamplona, Spain; 5 Hospital de San Cecilio, Granada, Spain; 6 Hospital GermansTrias i Pujol, Barcelona, Spain; 7 Hospital de Elche, Elche, Spain; 8 Hospital Ramon y Cajal, Madrid, Spain

## Abstract

**Introduction:**

Improved survival among HIV-positive people due to the success of antiretroviral therapy has increased the risk of developing comorbidities linked to ageing. We describe the pattern of morbidity according to age in HIV-positive persons in the Cohort of the Spanish AIDS Research Network (CoRIS).

**Methods:**

We calculated the age distribution in CoRIS, from 2004 to 2014, as the proportion of total person-years (py) in each age group (<50; 50–55; 56–60; 61–65; >65). We calculated incidence rates (per 1000py) for each comorbidity and the distribution of the number of comorbidities in persons ≥50 compared with persons < 50 years. Age was modelled as a time-dependent variable.

**Results:**

Overall, 9569 (34385py of follow-up) persons were included, 83.5% were men, median age at entry was 35 years (interquartile range (IQR): 29–43) and median CD4 count 384 (IQR: 203–582). Figure 1 shows changes in current age distribution by year; the proportion of total py aged ≥50 years increased from 8.8 to 21.2%, from 2004 to 2014. Among those aged ≥50 years, 17% of the total py presented one comorbidity and 4% two or more, compared to 8 and 1%, respectively, among those aged < 50 years. Table 1 presents incidence rates for each comorbidity by age group. The most common comorbidities were psychiatric and Non-AIDS-Defining Malignancies (NADM). Comorbidity rates for cardiovascular, kidney-associated, bone fractures, metabolic and NADM were significantly higher for persons aged ≥50 years.

**Conclusions:**

Non-AIDS events have emerged as an important cause of comorbidity and multi-morbidity, especially among those with older age, and pose a new challenge for HIV treatment and care.

**Figure 1:** Age distribution in CoRIS since 2004 to 2014.

**Abstract WEPDB0105–Table 1:** Incidence rates (95% CI) per 1000 person-years for each comorbidity by age group

		Rate (95% CI) per 1000 person-years, by age group			
		
Comorbidity	n	All	<50 years	≥50 years	P-value
**Cardiovascular**	85	2.49 (2.01–3.08)	1.38 (1.01–1.88)	8.75 (6.53–11.72)	<0.001
**kidney-associated event**	89	2.61 (2.12–3.21)	2.07 (1.61–2.67)	5.60 (3.89–8.06)	<0.001
**Liver-associated event**	94	2.76 (2.25–3.37)	2.63 (2.10–3.29)	3.46 (2.18–5.48)	0.299
**Bone**	122	3.58 (3.00–4.28)	3.19 (2.60–3.91)	5.81 (4.06–8.31)	0.004
**Psychiatric**	212	6.30 (5.50–7.20)	6.30 (5.44–7.29)	6.28 (4.44–8.88)	0.987
**Metabolic**	87	2.55 (2.07–3.15)	1.69 (1.28–2.24)	7.40 (5.38–10.17)	<0.001
**Other non-AIDS infections**	11	0.32 (0.18–0.58)	0.27 (0.14–0.55)	0.57 (0.18–1.77)	0.281
**Non-AIDS malignancies (more than one event per person is considered)**	181	5.32 (4.60–6.16)	5.16 (4.40–6.06)	13.61 (10.77–17.20)	<0.001

# Sub article: WEPDB0106 Low rates of cholesterol screening despite cardiovascular risk in protease inhibitor-treated HIV patients in Botswana

**Published:** 2016-07-22

**Authors:** M Mosepele, L Mokgatlhe, PF Hudson, V Letsatsi, R Gross

**Affiliations:** 1 Faculty of Medicine, University of Botswana, Gaborone, Botswana; 2 Botswana Harvard Partnership, Gaborone, Botswana; 3 Immunology and Infectious Diseases, Harvard T.H. Chan School of Public Health, Boston, MA, United States; 4 Biostatistics, University of Botswana, Gaborone, Botswana; 5 Infectious Disease, University of North Carolina, Chapel Hill, NC, United States; 6 Princess Marina Hospital Infectious Disease Care Clinic, Gaborone, Botswana; 7 University of Pennsylvania Perelman School of Medicine, Philadelphia, PA, United States; 8 Botswana-UPenn Partnership, Gaborone, Botswana

## Abstract

**Introduction:**

Treatment of human immunodeficiency virus (HIV) with protease inhibitors (PIs) is associated with increases in serum cholesterol levels. This association is strongest among those on 1st generation ritonavir boosted PIs. However, little is known about routine cholesterol screening and statin use to reduce risk of cardiovascular disease (CVD) among HIV-infected patients on PIs in sub-Saharan Africa (SSA) and how to increase appropriate screening.

**Methods:**

Cholesterol screening and statin use was retrospectively assessed among HIV-infected patients on ritonavir boosted PI-containing antiretroviral (ART) between 2008 and 2012 at a large public urban HIV clinic in Botswana. Non-fasting lipid profile blood testing was prospectively recommended to the patient by the study team at time of enrolment for those without a lipid profile in the prior 12 months. Proportion of patients screened per year was calculated, and statin recommendation ascertained for each participant using atherosclerosis risk score (ASCVD) and Framingham risk score (FRS) as of 2012.

**Results:**

A total of 375 patients, median age 40 years, on ritonavir boosted PIs were enrolled. Sixty-four percent were female. Proportion of patients screened for hypercholesterolemia ranged between 19 and 30% per year during four years of observation, with 3% having hypercholesterolemia (>5.0 mmol/L) and 1% using statins. After enrolment, the proportion of patients screened increased to 80%, and 31% had hypercholesterolemia. ASCVD guidelines recommended statin therapy for 14.3% of participants versus 9.4% by FRS.

**Conclusions:**

Cholesterol screening during routine care among high risk HIV patients was low in a clinical setting in Botswana. The high rate of hypercholesterolemia and indication of statin therapy for nearly 15% of patients highlights a huge gap in addressing CVD risk reduction among PI-treated patients. The fact that patients obtained testing when directed to do so by study staff suggests that patient behaviour is not the barrier to testing. Future work should explore innovative ways to increase and sustain cholesterol screening and statin use to reduce CVD risk among HIV-infected patients in Botswana.

# Sub article: WEPDC0102 Evaluation of the AccuCirc device for early infant male circumcision in Kisumu, Kenya: uptake and safety

**Published:** 2016-07-22

**Authors:** I Nyaboke, V Pengo, S Ojuok, M Athiambo, E Okello, F Otieno, R Plank, RC Bailey

**Affiliations:** 1 Nyanza Reproductive Health Society, Kisumu, Kenya; 2 Division of Infectious Diseases, Brigham and Womens Hospital, Boston, MA, United States; 3 School of Public Health, University of Illinois at Chicago, Chicago, IL, United States

## Abstract

**Introduction:**

As countries in sub-Saharan Africa scale up medical male circumcision (MMC), they are considering long term sustainable strategies, including early infant male circumcision (EIMC). AccuCirc is a single use, disposable device that comes in a sterile pre-packaged kit and may have advantages over the Mogen clamp, which is the currently approved device for EIMC in Kenya. This study assesses the safety and acceptability of the AccuCirc device for EIMC in 600 male infants in Kisumu, Kenya.

**Methods:**

Infant boys are recruited through informational talks and materials at antenatal and maternal child health clinics, maternity wards and during post-natal visits. Mothers ≥18 years and their healthy infants aged ≤60 days with no genital abnormalities nor history of bleeding disorder and meeting weight-for-age criteria are enrolled. They are given a dorsal penile block, and circumcised using the AccuCirc. During the one-hour post-op observation period, questionnaires are administered to mothers to assess knowledge about EIMC and levels of satisfaction. Three days after circumcision the wound is assessed and mothers are asked additional questions. Data are entered into RedCap and analyzed using STATA version 13.1.

**Results:**

Among 541 mothers and babies screened, 359 (66%) were eligible. Of these 110 (31%) opted for a Mogen clamp procedure; 249 (69%) were enrolled and circumcised using AccuCirc. The median age of mothers was 26 years (IQR=22, 30); 20% were unmarried and 62% had greater than a primary education. The median age of infants circumcised was 16 days (IQR=7, 32); 27% were ≤7 days and 73% were ≤30 days. There were no severe adverse events (AEs); there were 17 (6.8%) moderate AEs, all due to bleeding that occurred immediately after device removal. All were resolved in less than one hour. There were also 9 (3.6%) incomplete cuts, which required completion using surgical scissors or the Mogen clamp. Bleeding and incomplete cuts were more frequent in older/heavier babies. No infections or injuries to the glans were observed.

**Conclusions:**

The AccuCirc device may be an efficient and safe alternative for EIMC. Restricting procedures to babies ≤30 days may reduce AEs. These results contribute evidence needed as countries transition from adult toward infant circumcision.

# Sub article: WEPDC0103 The interaction of low male circumcision and high partner concurrency on HIV risk in Africa: evidence from demographic and health surveys

**Published:** 2016-07-22

**Authors:** M Little, A Fox

**Affiliations:** 1 Institute for Health, Healthcare Policy and Aging Research, Rutgers University, New Brunswick, NJ, United States; 2 Department of Public Administration, University at Albany, Albany, NY, United States

## Abstract

**Introduction:**

HIV rates vary widely across sub-Saharan Africa. High rates of multiple concurrent sexual partners (MCP) and low rates of male circumcision (MC) have each separately been identified as underlying drivers of HIV in sub-Saharan Africa. However, their joint contribution to HIV risk has not been examined empirically.

**Methods:**

Using Demographic and Health Survey data on couples with linked serostatus, the joint impact of MC and MCP on HIV risk for men and women was examined through multilevel models that accounted for the prevalence of both indicators at the regional-level. “High-risk individuals” were categorized as those whose partner had other sexual partners and where the male was uncircumcised and “high-risk regions” as those where >5% of the population had multiple partners and <80% of the population was circumcised. A varying-intercept model was run to identify the intercepts for MC prevalence and MCP prevalence and their interaction on individual risk for HIV infection, adjusting for individual demographic and behavioural covariates. The models were run on nearly 30,000 observations across 96 regions within 11 sub-Saharan countries.

**Results:**

Assessing the joint impact of MC and MCP at the individual-level, higher-risk individuals had up to a 15% higher likelihood of contracting HIV (p<0.05). Assessing the joint impact at the regional level, living in a high-risk region was associated with up to a 3.2 times higher likelihood of being HIV-positive for men and up to nearly two times higher likelihood for women (p<0.001). The lowest-risk regions had a mean HIV prevalence of 1.4% and highest-risk regions had a prevalence of 21.6%. With nearly a three-times higher relative risk, the regional-level interaction risk factor was more predictive of the HIV status for both sexes than the individual-level interaction factor (p<0.001).

**Conclusions:**

MC and MCP should not be addressed as separate interventions. While much emphasis has been placed on scaling up male circumcision, in the absence of concerted efforts to reduce sexual concurrency, increased circumcision may have a less-than-anticipated impact. Adopting an integrated approach to addressing male circumcision and sexual concurrency is critical to achieving the Sustainable Development Goal target 3.3: Ending the AIDS epidemic by 2030.

# Sub article: WEPDC0104 Barriers to and facilitators of VMMC uptake among older men aged 25-39 years in Nyanza Region, western Kenya: the TASCO study

**Published:** 2016-07-22

**Authors:** K Agot, J Grund, E Mboya, P Musingila, E Omanga, D Emusu, E Odoyo-June, S Ohaga, B Otieno-Nyunya

**Affiliations:** 1 Research, Impact Research & Development Organization, Kisumu, Kenya; 2 Division of Global HIV & TB (DGHT), Centers for Disease Control and Prevention, Atlanta, GA, United States; 3 Centers for Disease Control and Prevention, Kisumu, Kenya

## Abstract

**Introduction:**

Kenya began rolling out voluntary medical male circumcision (VMMC) in 2008, with a goal of reaching 80% of approximately 1 million uncircumcised males aged 15–49 years by 2013. While over 700,000 males were circumcised by 2013, client demand for VMMC was primarily among younger clients, and uptake was lowest among men aged ≥25 years. We present reasons reported by men ≥25 years for going or not going for VMMC.

**Methods:**

Between May 2014 and December 2015, we conducted a cluster randomized controlled study to assess the impact of two interventions (enhanced interpersonal communication and dedicated clinics for men aged ≥25 years) on the uptake of VMMC. We administered a questionnaire to men aged 25–39 years who were already circumcised at enrolment and those who received VMMC during the study on their reasons for going for VMMC. We also interviewed men who were uncircumcised at enrolment and those who remained uncircumcised at the end of the study, on their reasons for not going for circumcision.

**Results:**

We interviewed 3200 circumcised men and 2781 uncircumcised men. For men who were circumcised before the study (n=2848), reduction in HIV risk (43.7%) (95% CI: 41.9, 45.6) and culture/religion (18.4%) (95% CI: 17.1, 19.9*)* were the two most important reasons for getting circumcised, while for men circumcised during the study (n=352), reduction in HIV risk (50.9%) (95% CI: 45.6, 55.6) and improved genital hygiene (16.2%) (95% CI: 12.5, 20.2) were the top reasons for getting circumcised. For those uncircumcised at enrolment (n=2781), lost wages was the main barrier (34.2%) (95% CI: 32.6, 36.0) followed by pain (27.8%) (95% CI: 26.1, 29.4) while among those who remained uncircumcised when the study closed and were interviewed at exit (n=1265), top barriers were inconvenient time/venue (43.2%) (95% CI: 40.4, 46.0) and lost wages (26.0%) (95% CI: 23.7, 28.5).

**Conclusions:**

Reduction in HIV risk remains the primary reason why men aged 25–39 pursue circumcision while losing wages is the primary reason others remain uncircumcised. Demand creation efforts for older men must amplify key client-level facilitators while overcoming primary barriers.

# Sub article: WEPDC0105 Safety of the no-flip technique and spontaneous detachment for ShangRing circumcision in boys and men: results from a randomized controlled trial

**Published:** 2016-07-22

**Authors:** QD Awori, R Lee, P Li, D Ouma, N Obura, M Oundo, D Mwamkita, B Chirchir, M Barasa, M Kirui, P Macharia, J Oketch, P Otiende, N Nyangweso, M Maina, N Kiswi, M Goldstein, J Nyanchoka, MA Barone

**Affiliations:** 1 ShangRing Research Project, EngenderHealth, Nairobi, Kenya; 2 Urology, Weill Cornell Medical College, New York, NY, United States; 3 Bon Sante Consulting, Nairobi, Kenya; 4 NASCOP, Ministry of Health, Nairobi, Kenya; 5 Homa Bay County Referal Hospital, VMMC, Homa Bay, Kenya; 6 Vipingo Health Centre, VMMC, Kilifi, Kenya; 7 ShangRing Research Project, EngenderHealth, New York, NY, United States

## Abstract

**Introduction:**

Use of medical devices for voluntary medical male circumcision (VMMC) can offer several advantages. The ShangRing, a disposable single-use circumcision device, is simple to use, safe, and well-accepted in males 13 years old and above. We evaluated the safety, effectiveness, and acceptability of circumcision in males 10 years and above using a modified “no-flip” ShangRing technique, in addition to allowing spontaneous detachment of the device (as opposed to ring removal seven days after circumcision).

**Methods:**

We enrolled men and boys seeking VMMC at two sites in Kenya. Participants were randomized to standard ShangRing removal seven days after circumcision vs. spontaneous device detachment. Weekly follow-up visits included evaluation of the degree of detachment if the ring was still in place, occurrence of adverse effects (AEs), and status of wound healing. Participants in the spontaneous detachment group could request device removal at any point during follow-up.

**Results:**

230 men and boys underwent ShangRing circumcision using the no-flip technique; 114 and 116 were randomized to the seven-day and spontaneous detachment groups, respectively. Mean ages in the two groups were 17.4 and 19.0 years, respectively. Mean circumcision times between the groups were similar (7.3±2.5 vs. 7.0±2.6; p=0.4). All circumcisions were successfully completed using the ShangRing. Six (5.2%) and two (1.7%) moderate AEs were reported in the seven-day and spontaneous detachment groups, respectively, and were similar (p=0.17); there were no severe AEs. 84(72.4%) participants in the spontaneous detachment group wore the ring until it fell off; the remainder requested earlier removal. The probability of complete spontaneous detachment on seven, 14, and 28 days post-circumcision was 0.11, 0.63, and 1.00, respectively. Satisfaction with cosmetic results was high and similar in both groups – 98.9 and 96.0% (p=0.3).

**Conclusions:**

These results demonstrate the safety, acceptability, and effectiveness of the “no-flip” technique in males 10 years old and above, with 100% eligibility for all screened participants. Spontaneous detachment of the ring was safe and effective and was acceptable to a majority of men and boys. Use of the ShangRing as a single visit may significantly reduce the burden of service provision at health facilities.

# Sub article: WEPDC0106 Adolescent girls’ support of male peers and sexual partners receiving voluntary medical male circumcision services: implications for demand creation

**Published:** 2016-07-22

**Authors:** M Kaufman, K Dam, K Hatzold, L Van Lith, G Ncube, G Lija, C Bonnecwe, W Mavhu, C Kahabuka, K Seifert-Ahanda, A Marcell, L Mahlasela, ME Figueroa, E Njeuhmeli, A Tobian, and Adolescent Vmmc Technical Advisory Group

**Affiliations:** 1 Department of Health, Behavior & Society, School of Public Health, Johns Hopkins Bloomberg, Baltimore, MD, United States; 2 Center for Communication Programs, Johns Hopkins University, Baltimore, MD, United States; 3 Population Services International, Harare, Zimbabwe; 4 Ministry of Health and Child Welfare, Harare, Zimbabwe; 5 Ministry of Health and Social Welfare, Dar es Salaam, United Republic of Tanzania; 6 National Department of Health, Pretoria, South Africa; 7 CSK Research Solutions, Dar es Salaam, United Republic of Tanzania; 8 United States Agency for International Development (USAID) Washington/Global Health Bureau/Office of HIV/AIDS, Washington, DC, United States; 9 School of Medicine, Johns Hopkins University, Baltimore, MD, United States; 10 Centre for Communication Impact, Pretoria, South Africa

## Abstract

**Introduction:**

Voluntary medical male circumcision (VMMC) in sub-Saharan Africa has reached a large number of adolescent males (ages 10–19). While some research has evaluated what attracts these boys to services, little is known as to female adolescents’ involvement, if any, in the decision-making process and support of males being circumcised. This study explored adolescent girls’ support of their male peers and sexual partners undergoing the procedure.

**Methods:**

Twelve focus group discussions (FGDs) were conducted with female adolescents (ages 16–19) in South Africa, Tanzania, and Zimbabwe. These FGDs focused on the girls’ opinions and perceptions of VMMC, including their perceived influence on VMMC uptake. In addition, 92 interviews were conducted with male adolescent VMMC clients 6–8 weeks post-procedure, which asked about their experiences in sharing their VMMC status or experience with girls. Audio recordings were transcribed, translated into English, and coded by two independent coders using qualitative coding software. Coders discussed discrepancies until at least 85% agreement was reached. Coded text was then assessed for themes.

**Results:**

Overall, girls are supportive of VMMC. Girls discussed preferring circumcised male sexual partners over uncircumcised ones, citing the former's sexual appeal, hygiene, better sexual performance, and reduced chances of passing on infections (including HIV). Additionally, girls discussed being supportive of boys’ decision to be circumcised and both overtly and covertly influencing their peers/partners to undergo VMMC. This was corroborated by some older boys who described how girls made them feel that if they got circumcised, then such girls would be more interested in them. In some instances, older boys reported girls using VMMC as criteria for selecting male partners. Girls discussed not necessarily offering tangible support during the healing process, but rather emotional support in making the decision to get circumcised. Younger boys (<15 years) reported not interacting with girls much at all regarding VMMC.

**Conclusions:**

Findings show that adolescent girls are involved in the VMMC decision-making process, especially with older adolescent boys. Given the apparent role of female peers/partners as support in influencing VMMC uptake, demand creation initiatives should continue to engage females in promoting VMMC to their male counterparts.

# Sub article: WEPDC0107 Safety of a facility-based versus community-based model of early infant male circumcision using the Mogen clamp in western Kenya: Mtoto Msafi Mbili Study

**Published:** 2016-07-22

**Authors:** F Adera, M Young, T Adipo, F Otieno, S Nordstrom, S Mehta, RC Bailey

**Affiliations:** 1 Nyanza Reproductive Health Society, Kisumu, Kenya; 2 Obstetrics and Gynecology, Emory University, Atlanta, GA, United States; 3 Obstetrics and Gynecology University of Illinois at Chicago, Chicago, IL, United States; 4 Epidemiology, University of Illinois at Chicago, Chicago, United States

## Abstract

**Introduction:**

As countries in sub-Saharan Africa (sSA) scale up male circumcision, they are considering long term sustainable strategies, including early infant male circumcision (EIMC). An important aspect of introducing EIMC in sSA settings is safety. We present AE rates associated infant circumcisions achieved during the Mtoto Msafi Study.

**Methods:**

A standard delivery package (SDP) included training health providers in four facilities to deliver safe EIMC and all health facility staff to educate, promote and mobilize mothers in antenatal, maternal neonatal child health (MNCH) and immunization clinics and surrounding communities. A SDP-PLUS model included all SDP activities in four facilities plus provision of EIMC services in the community by trained domiciliary midwives (DM). Infant boys were recruited through informational talks at MNCH and maternity wards, during post-natal visits and in the community by the DMs. Mothers ≥16 years and their healthy infants aged ≤60 days with no genital abnormalities nor history of bleeding disorder and meeting weight-for-age criteria were eligible. They were circumcised using the Mogen clamp after a dorsal penile block. Follow-up to assess the wound occurred three days after circumcision or as needed.

**Results:**

Among 1681 babies screened, 1598 (95%) were eligible and circumcised: 561 in the SDP and 1037 in the SDP-PLUS community. Reasons for ineligibility were: underweight-for-age (34%), rashes or infections (18%), fever (15%) genital abnormalities (12%), jaundice (8%) and other (13%). Median age of mothers was 24 years (IQR=20, 28); median age of infants was 8 days (IQR=136) and median weight was 3.6 kg (IQR=3.1, 4.4). Follow-up occurred in 72% of babies. There were six moderate (0.3%) and five severe (0.3%) adverse events (AEs). Among SAEs, three were in the context of training. Three were deaths, two of which were unrelated to EIMC, one possibly related. One was intra-operative bleeding requiring suturing, and one was a post-operative hematoma. No AEs were associated with procedures done in the community by DMs.

**Conclusions:**

EIMC can be provided in an sSA community setting safely with low occurrence of AEs. SAEs possibly related or unrelated to the procedure may occur, requiring emergency response. These results contribute evidence needed as countries transition from adult toward infant circumcision.

# Sub article: WEPDC0201 Vaginal bacteria associated with increased risk of HIV acquisition in African women

**Published:** 2016-07-22

**Authors:** RS McClelland, JR Lingappa, G John-Stewart, J Kinuthia, K Yuhas, W Jaoko, S Srinivasan, KN Mandaliya, TL Fiedler, MM Munch, BA Richardson, J Overbaugh, DN Fredricks

**Affiliations:** 1 University of Washington, Seattle, WA, United States; 2 Kenyatta National Hospital, Nairobi, Kenya; 3 University of Nairobi, Nairobi, Kenya; 4 Fred Hutchinson Cancer Research Center, Seattle, WA, United States

## Abstract

**Introduction:**

Disruptions of the vaginal microbiota have been associated with increased HIV-1 risk. This study utilized molecular characterization of vaginal microbiota to test the hypothesis that specific vaginal bacteria are associated with increased risk of HIV-1 acquisition.

**Methods:**

A nested case-control study was conducted in cohorts of women in Kenya, Uganda, Tanzania, Zambia, Botswana and South Africa. Vaginal microbiota was compared at the pre-seroconversion sample in women who acquired HIV-1 (cases) versus women in the same cohort who remained seronegative (controls). Characterization of vaginal microbiota included deep sequence analysis of broad-range 16S rRNA gene polymerase chain reaction (PCR) products, and bacterium-specific quantitative PCR (qPCR) assays for selected bacteria.

**Results:**

Among 349 women (87 cases and 262 controls), 40 were from a female sex worker cohort, 112 were from a cohort of pregnant and post-partum women and 197 were HIV-seronegative women in discordant couples cohorts. Their median age was 28 years (interquartile range 22–35) and 77 (22.1%) were pregnant. Vaginal bacterial community diversity was higher in women who acquired HIV-1 compared to seronegative controls (mean Shannon Diversity Index 1.3 (standard deviation (SD) 1.0) versus 0.9 (SD 0.9), p=0.02. Based on comparison of relative abundance in cases versus controls, 15 taxa were selected for qPCR testing. Of these, *Eggerthella* species type-1, *Gemella asaccharolytica*, *Leptotrichia/Sneathia*, *Megasphaera* and *Mycoplasma hominis* each showed a significant association with HIV-1 acquisition when undetectable levels were compared to tertiles representing increasing bacterial concentrations. High correlation between species precluded including multiple species together in a single multivariable model. These results remained significant after adjustment for age, pregnancy, contraceptive type, number of sex partners, frequency of sex and recent unprotected intercourse (Figure 1).

**Conclusions:**

Women's HIV-1 susceptibility may be influenced by the presence and quantity of key vaginal bacteria, including a number of fastidious bacteria recently linked to bacterial vaginosis.

**Figure 1:** Association between vaginal bacterial concentration and odds of HIV acquisition.

# Sub article: WEPDC0202 A brief, trauma-informed intervention is feasible and acceptable, increases safety behaviour, and reduces HIV risk among drug-involved women who trade sex

**Published:** 2016-07-22

**Authors:** M Decker, C Tomko, E Wingo, S Peitzmeier, A Sawyer, N Glass, S Sherman

**Affiliations:** 1 Johns Hopkins Bloomberg School of Public Health, Baltimore, MD, United States; 2 Baltimore City Health Department, Baltimore, MD, United States; 3 Johns Hopkins School of Nursing, Baltimore, MD, United States

## Abstract

**Introduction:**

The HIV epidemic among female sex workers (FSWs) is shaped by structural, social network and behavioural factors. Violence is pervasive and associated with risk behaviour and infection, yet interventions to respond to violence are limited.

**Methods:**

Our intervention was developed in partnership with practitioners and clients from community-based organizations, who prioritized violence-related support, connection to services and responding to the myth that sex workers cannot be raped. The brief (3–5 minute), trauma-informed intervention (INSPIRE) was implemented with drug-involved FSWs in Baltimore, MD and evaluated for feasibility, acceptability and effect via a quasi-experimental, single group pretest-posttest study; baseline n=60; n=39 (65%) at follow-up; non-differential by baseline measures.

**Results:**

At follow-up, participants had improved condom negotiation confidence (p=0.04), and reduced frequency of sex trade under the influence of drugs/alcohol (p=0.04). Endorsement of sex work-related rape myths decreased (p=0.04), and safety behaviour scores increased (p<0.001). Participants improved knowledge and use of support services for sexual violence and intimate partner violence. At follow-up, 68% knew at least one place to obtain assistance reporting violence to police, and 29% had approached such a programme. Participants emphasized the value of a safe and supportive space to discuss violence; their feedback and that of community partners indicated high feasibility and acceptability of this brief, low-dose intervention.

**Conclusions:**

Findings indicate the feasibility and acceptability of brief, trauma-informed discussion of safety and resources in the context of HIV risk reduction for FSWs and suggest the potential for impact. This approach appears to prompt engagement in safety strategies, decrease the extent of sex trade under the influence and bolster confidence in condom negotiation. INSPIRE influenced endpoints identified as valuable by community partners, specifically connection to support services and countering structural forces that falsely blame sex workers for violence. Future implementation research can advance limitations of our pilot study, including the short follow-up duration and attrition. These early results can inform scalable interventions that address the impact of trauma on HIV acquisition and care trajectories for FSWs, and in doing so address the dual epidemics of violence and HIV to support health and human rights.

# Sub article: WEPDC0203 HIV pre-exposure prophylaxis (PrEP) formulation preference among women participating in the qualitative component of the ASPIRE (MTN-020) study

**Published:** 2016-07-22

**Authors:** A van der Straten, MK Shapley-Quinn, K Reddy, H Cheng, J Etima, K Woeber, P Musara, T Palanee-Phillips, J Baeten, ET Montgomery, MTN-020/ASPIRE Study Team

**Affiliations:** 1 RTI International, WGHI/ Center for Global Health, San Francisco, CA, United States; 2 Center for AIDS Prevention Studies (CAPS), University of California San Francisco, San Francisco, CA, United States; 3 Wits Reproductive Health and HIV Institute, Johannesburg, South Africa; 4 Makerere University – Johns Hopkins University Research Collaboration, Kampala, Uganda; 5 Medical Research Council, Durban, South Africa; 6 UZ-UCSF Collaborative Research Programme, Harare, Zimbabwe; 7 University of Washington, Seattle, United States

## Abstract

**Introduction:**

During MTN-020/ASPIRE, a phase III trial of the Dapivirine vaginal ring in Africa, preferences for various PrEP delivery forms (including the ring) were explored in a subsample of participants receiving exit in-depth interviews (IDIs).

**Methods:**

Participants were presented with pictures and descriptions of 9 possible PrEP formulations (vaginal gel, ring, suppositories and films; oral tablets, injections, implants, male and female condoms) and asked to discuss these, first in relation to the ring and second to select the formulations they would be most/least interested in for future use. IDIs were summarized in reports for rapid review of key findings; levels of interest in products were tabulated and themes related to product preference were extracted.

**Results:**

In the qualitative subsample (N=71; Malawi n=12; South Africa n=34, Uganda n=13, Zimbabwe n=12), baseline median age was 26 (range 18–45 years), all had a primary sex partner; 41% reported using a male condom at last sex; the most common current contraceptives were injections (52%) and implants (24%). Participants expressed most interest for future PrEP formulated as rings (94%), implants (39%) and injections (34%). Positive attributes of these methods included being long-acting, discreet, familiar and easy-to-use. The ring was also liked for reliability, lack of side effects and comfort. Opinions were divided for implants and injections (28 and 32% uninterested in future use, respectively) due to needle-phobia, pain upon administration, low reversibility and fear of side effects based on previous contraceptive experience. Formulations participants had least interest in included: oral tablets (61%), vaginal gel (55%) and film (41%). Attributes of tablets that were disliked included the daily regimen, difficulty in swallowing and stigma related to taking HIV medicines. The gel, films and other vaginal formulations were disliked because of the act of vaginal insertion, coital use, effect on sex and lack of familiarity.

**Conclusions:**

Diverse PrEP formulations elicited interest in this subsample, with long-acting methods being favoured. Despite high interest in the vaginal ring, other vaginal products did not generate much interest. Familiarity, reliability, absence of side effects and low burden in terms of administration and use were determined as important attributes to consider for new PrEP formulations.

# Sub article: WEPDC0204 Sexual behaviour of men and women within age-disparate partnerships in South Africa: implications for young women's HIV risk

**Published:** 2016-07-22

**Authors:** B Maughan-Brown, M Evans, G George

**Affiliations:** 1 Southern Africa Labour and Development Research Unit (SALDRU), University of Cape Town, Cape Town, South Africa; 2 Institute for Humanities in Africa (HUMA), University of Cape Town, Cape Town, South Africa; 3 Department of Sociology, University of Cape Town, Cape Town, South Africa; 4 Health Economics and HIV and AIDS Research Division (HEARD), University of KwaZulu NatalDurban, South Africa

## Abstract

**Introduction:**

Age-disparate partnerships are hypothesized to increase HIV-risk for young women. However, the evidence base remains mixed. Most studies have focused only on unprotected sex among women in the partnership. Consequently, little is known about other risky behaviours, such as transactional sex, alcohol use and concurrency, as well as the behaviours of the men who partner with young women. We therefore examined various sexual behaviours of both young women and of men in partnerships with young women in order to investigate whether age-disparate partnerships involve riskier sexual behaviour.

**Methods:**

We used nationally representative data from South Africa (2012) on partnerships reported by 16–24 year old women (n=818) and by men in partnerships with 16–24 year old women (n=985). We compared sexual behaviours in age-disparate partnerships and age-similar partnerships, using multivariate logistic regression to control for potential confounders and to assess rural/urban differences.

**Results:**

Young women in age-disparate partnerships were more likely to report unprotected sex than in similar-aged partnerships (aOR: 1.51; p<0.05). Men in partnerships with young women were more likely to report unprotected sex (aOR: 1.92; p<0.01), transactional sex (aOR: 2.73; p<0.01), drinking alcohol before sex (aOR: 1.60; p<0.1), and concurrency (aOR: 1.39; p<0.1) when their partners were five or more years younger. Significant associations between age-disparate partnerships and transactional sex (aOR: 4.14; p<0.01) and alcohol use (aOR: 2.24; p<0.05) were only found in urban areas.

**Conclusions:**

Results provide evidence that young women's age-disparate partnerships involve greater sexual risk, particularly through the risky behaviours of their male partners, with the risk amplified for young women in urban areas.

# Sub article: WEPDC0205 Evidence for selection effect and Hawthorne effect in behavioural HIV prevention trial among young women in rural South Africa

**Published:** 2016-07-22

**Authors:** M Rosenberg, A Pettifor, R Twine, J Hughes, FX Gomez-Olive, RG Wagner, A Selin, C MacPhail, K Kahn

**Affiliations:** 1 Center for Population and Development Studies, School of Public Health, Harvard University, Cambridge, MA, United States; 2 Department of Epidemiology, University of North Carolina – Chapel Hill, Chapel Hill, NC, United States; 3 MRC/Wits Rural Public Health and Health Transitions Research Unit (Agincourt), University of the Witwatersrand, Johannesburg, South Africa; 4 Department of Biostatistics, University of Washington, Seattle, WA, United States; 5 Carolina Population Center, University of North Carolina – Chapel Hill, Chapel Hill, NC, United States; 6 School of Health, University of New England, Armidale, Australia

## Abstract

**Introduction:**

HPTN 068 was a randomized controlled trial to examine whether cash transfers conditional on school attendance reduce the risk of HIV acquisition in young South African women. Findings indicated no difference in HIV acquisition between study arms, with low HIV incidence and high levels of school enrolment in both treatment and control groups. We examine whether school enrolment trajectories of the study participants differed from the underlying study population, and whether differences could be attributed to existing differences in school enrolment at baseline (selection effect) or differences that arose during study participation (Hawthorne effect).

**Methods:**

Using census data from the Agincourt Health and Socio-Demographic Surveillance System within which the HPTN 068 trial was nested, we constructed a cohort of 2525 young women between the ages 13 and 20 in 2011. Using log-binomial regression models, we compared 2011 and 2012 school enrolment between those who did (n=1145) and did not (n=1380) enrol in HPTN 068 in 2011. To isolate the Hawthorne effect we restricted the cohort to those enrolled in school at time of study enrolment. We adjusted for key socio-demographic characteristics and stratified by age.

**Results:**

Nearly all HPTN 068 participants (97%) were still enrolled in school in 2012 compared to 86% of non-participants. The magnitude of association between study enrolment and school enrolment was strongest among those who were older at baseline. Small but statistically significant effects remained in the restricted cohort. Similar preliminary findings were observed in 2013.

**Conclusions:**

HPTN 068 participants, regardless of study arm, were more likely to be enrolled in school than non-participants. Our findings suggest that both selection and Hawthorne effects may have diminished the differences in school enrolment between study arms and is one plausible explanation for the null study effect. The Hawthorne-specific findings generate hypotheses for how to structure school retention interventions to prevent HIV.

**Abstract WEPDC0205–Figure 1:** The association between enrollment in the HIV prevention trial in 2011 and school enrollment in 2011, 2012, and 2013, stratified by age, in both the full cohort, and a restricted cohort of those enrolled in school in 2011.

# Sub article: WEPDC0206 Adherence to topical PrEP: qualitative findings from the FACTS 001 trial

**Published:** 2016-07-22

**Authors:** J Stadler, S Delany-Moretlwe, D Baron, S Ju, G Gray, H Rees, FACTS 001 Study Group

**Affiliations:** 1 Reproductive Health and HIV Institute, University of the Witwatersrand, Johannesburg, South Africa; 2 CONRAD, Arlington, TX, United States; 3 SAMRC, Cape Town, South Africa

## Abstract

**Introduction:**

In the FACTS 001 phase III trial, a peri-coital (before and after sex) vaginal application of tenofovir 1% gel did not prevent HIV-1 infection amongst young South African women. Given that sub-optimal adherence can dilute estimations of efficacy, understanding what shaped gel use in FACTS 001 is critical to comprehend these outcomes.

**Methods:**

A random sample of 145 participants was selected from the trial population, weighted to reflect enrolment distribution at the nine trial sites. Participants were invited to an in-depth interview (IDI) at their product discontinuation visit. IDIs were conducted in a language of the participant's choice and were recorded, transcribed and translated. Nvivo 10 was used to code interview text that reflected the key research objectives and themes arising from the literature on other HIV prevention trials: risk of HIV; perceived efficacy and acceptability of the gel; comprehension of the peri-coital dosing regimen; and relationship dynamics.

**Results:**

A total of 136 participants were interviewed. Interviewees broadly represented the main trial population: a cohort of predominantly young (average 22 years), single (87%) and unemployed women (78%), who mostly resided with their parents (62%). Recognizing their risk of HIV infection, they expressed hopefulness that the trial gel would provide protection. Women liked the gel because it enhanced their sexual experiences, and they believed it improved their health. However, the required dosing regimen, especially the post-sex dose, was not always feasible under certain circumstances, particularly when living apart from partners, and when attempting to conceal gel use from partners.

**Conclusions:**

Despite their perceived vulnerability to HIV, the hope for an effective product, and favourable experiences of using the gel, many FACTS 001 participants were unable to integrate the gel into their lives and use it consistently enough to provide protection against HIV. Social contextual factors such as residential and intimate partner dynamics can be critical in limiting women's agency to use HIV prevention products. This has implications for the PrEP field and indicates the need for programmes to support adherence that considers women's everyday lives.

# Sub article: WEPDD0101 Promise or Peril? The nature of medical pluralism along the cascade of care for HIV/AIDS in eastern and southern Africa

**Published:** 2016-07-22

**Authors:** M Moshabela, D Bukenya, G Darong, J Wamoyi, T Zuma, J Renju, C Nyamukapa, W Ddaaki, O Bonnington, J Seeley, V Hoosegood, A Wringe

**Affiliations:** 1 Africa Centre for Population Health, University of KwaZulu Natal, Durban, South Africa; 2 Uganda Virus Research Institute Programme on AIDS, Medical Research Council, Entebbe, Uganda; 3 Africa Centre for Population Health, University of KwaZulu Natal, Mtubatuba, South Africa; 4 Tanzanian National Institute of Medical Research, Mwanza, United Republic of Tanzania; 5 Kilimanjaro Christian Medical Centre, Moshi, Tanzania, United Republic of; 6 London School of Hygiene and Tropical Medicine, London, United Kingdom; 7 Biomedical Research and Training Institute, Harare, Zimbabwe; 8 Imperial College, London, United Kingdom; 9 Rakai Health Sciences Program, Rakai, Uganda; 10 Southampton University, Southampton, United Kingdom; 11 Africa Centre for Population Health, Mtubatuba, South Africa

## Abstract

**Introduction:**

There are concerns that medical pluralism may delay patients' progression through the HIV cascade and contribute to poor treatment outcomes. However there is a dearth of evidence regarding pluralistic practices among PLHIV in different African settings in the context of widespread ART use. We aimed to address this gap by documenting patients' experiences with HIV care across medical systems, identify dominant patterns of medical pluralism and explore their implications for health outcomes.

**Methods:**

We purposively selected 180 participants from each stage of the cascade in six demographic surveillance sites in five east and southern African countries: Uganda, Kenya, Tanzania, Malawi, Zimbabwe and South Africa. In-depth interviews were conducted using shared tools across sites. We used pathways to care analysis to code and map the health care-seeking journeys of participants, which were compared with their illness experiences using a constant comparison method.

**Results:**

Identified patterns of medical pluralism included use of dominant public sector clinics and hospitals with use of

1. private sector practitioners and chemists,
2. indigenous sector traditional healers and herbalists and
3. religious sector faith healers and prophets.

These patterns differed depending on the cascade stage, available sources of health care and other contextual factors in each country. Sequential medical pluralism, adopted for alternative care purposes, appeared more common prior to ART, largely around HIV testing and linkage to care, both associated with delays. Concurrent medical pluralism, used for complementary purposes, appeared more common among ART patients. Patients engaged in medical pluralism predominantly to compensate for aspects of HIV care needed but not received from their main public sector providers, rather than to seek substitute services.

**Conclusions:**

Sequential medical pluralism may act as a bottleneck towards ART initiation. Concurrent medical pluralism suggests a tendency towards complementary forms of health care utilization among ART users, which may be deemed necessary by individual patients in order to complete their desired or required package of health care. Complementary approaches may serve a purpose of resolving tensions and minimize competition between sources of health care, but carry the risk of drug-drug interactions.

# Sub article: WEPDD0102 Claims of a cure: use of CD4 cell count results to guide traditional treatment in Bushbuckridge, South Africa

**Published:** 2016-07-22

**Authors:** C Audet, S Ngobeni, R Wagner

**Affiliations:** 1 Medical Center, Health Policy & Global Health, Vanderbilt University, Nashville, TN, United States; 2 University of the Witwatersrand, Johannesburg, South Africa

## Abstract

**Introduction:**

Traditional healers play an important role in providing health care in much of sub-Saharan Africa, due to both their greater accessibility and acceptability. In rural northeastern South Africa, studies have documented HIV patients using both traditional and allopathic healers, often “ping-ponging” back and forth between the two systems. The initial use of traditional healers can cause delays in initiation of life-saving medicine for serious conditions such as HIV.

**Methods:**

We conducted 27 in-depth interviews and 133 surveys with a random sample of traditional healers living in Bushbuckridge, South Africa, to document illnesses treated, methods for diagnosis, self-reported effectiveness of treatments and the monetary fees they charged for a variety of ailments, including HIV.

**Results:**

Healers in the rural Bushbuckridge were mostly female (77%), older (median=58 (IQR: 50–67) years), with low levels of education (median=3.7 (IQR: 3.2–4.2) years). Our qualitative interviews revealed that healers treating probable HIV-infected patients first referred them to the clinic for testing and confirmation. Subsequently if the patient preferred traditional treatment, they differentiated between two categories of known HIV-infected patients: (1) those with CD4<350 cells/mm^3^ and (2) those with >350 cells/mm^3^. Only patients with “low” CD4 cell counts were routinely referred back to health facilities for antiretroviral therapy. Among those surveyed, 39 (30%) reported successfully treating adult HIV-infected patients with CD4 cell >350 cells/mm^3^. Healers who reported treating HIV-infected patients treated more patients (median 8.7 vs. 4.8 per month; p=0.03), had been practicing for less time (median 16.9 vs. 22.8 years; p=0.03), and had lower levels of education (2.8 vs. 4.1 years; p=0.017). Both groups experienced similar number of blood exposures during their treatments (median 1.5 vs. 1.0; p=0.24). Healers charged a median of 1500 Rand (~92 USD) to treat patients (with high CD4) for HIV.

**Conclusions:**

Traditional healers in rural South Africa do refer suspected HIV-infected patients to biomedical care, yet continue to treat patients once confirmed, particularly when patients have a CD4 cell count >350 cells/mm^3^. Given that patients with higher CD4 cell counts have fewer physical symptoms of HIV-infection, a greater emphasis on patient education and healer engagement are warranted.

# Sub article: WEPDD0103 Moonlight methadone for Muslims on medically assisted therapy curbing drug relapse in Malindi, Kenya

**Published:** 2016-07-22

**Authors:** S Abdallah, F Ibrahim, M Kirimo, A Mongi, J Baya, M Shossi, A Omar

**Affiliations:** 1 UNODC, Mombasa, Kenya; 2 Kilifi County, Kilifi, Kenya; 3 Malindi Sub-County Hospital, Malindi, Kenya; 4 The Omari Project, Malindi, Kenya

## Abstract

**Introduction:**

It is widely acknowledged that heroin dependence results in homelessness, family disruption, social instability and marginalization. Anecdotal reports indicate many members of society regard people who use drugs as sinners. Although 2011 UNODC Study Kenya revealed 55% of all PWID are Christian, 42% Muslim. Nairobi PWID comprise 16% versus 72% at Coast. Evidence regarding spiritual support for PWID is limited.

**Description:**

Following initiation of Medically Assisted Therapy (MAT) programme in Malindi, enrolled Muslim MAT clients expressed a desire to fast during Ramadhan 2015. From May 2015 they requested MAT clinic team to dispense methadone after sunset for Muslim clients or wean them off methadone 6 weeks before Ramadhan.

Unfortunately national guidelines for MAT don’t recommend take home doses, dispensing by non-pharmacists or beyond operational hours. International MAT experts restricted detox for incarcerated MAT clients and advised against shifting MAT Clinic operational hours to assure structured way of life for clients. Religious leaders recommended MAT clients adopt Islam’s waiver from fasting for sick, pregnant or nursing women. Malindi hospital unwilling to dispense methadone at 24-hour main pharmacy due to security concerns. Mathari MAT Clinic in Nairobi rejected a similar plea as its Muslim MAT Clients were few (<10).

**Lessons learned:**

On 1st day of Ramadhan 2015, almost 40 clients missed daily methadone dose. By 3rd day, severe withdrawal drove a few Muslim clients to MAT pharmacy for daily dose. Clients who showed up on subsequent days required re-induction. However, by 7th Ramadhan, 29 clients still kept away. Rumours that some fasting clients were taking heroin after sunset and at pre-dawn to manage their withdrawal prompted Malindi MAT Clinic team to unanimously approve evening dispensing for fasting Muslim clients. Eligibility for evening doses for MAT defaulters: 3-days re-induction at daytime. Three (3) fasting clients refused to comply. On 12th Ramadhan, 26 fasting clients accessed evening services. All routinely reported immediately after prayers; within 40 minutes, all doses dispensed. This service was halted on Eid day.

**Conclusions/Next steps:**

Moonlight dispensing enabled Muslim clients to fast after years of drug use while improving client-provider relations. As Kenya scales up MAT programme, prioritize spiritual recovery for MAT Clients.

# Sub article: WEPDD0104 Taking on faith: a narrative analysis of discussions about HIV used by participants platforms for contesting faith in nine high HIV-burden communities in the Western Cape, South Africa

**Published:** 2016-07-22

**Authors:** S Nomsenge, A Thomas, G Hoddinott, G Carolus, V Bond, and On behalf of HPTN 071 (PopART) Study Team

**Affiliations:** 1 Department of Paediatrics and Child Health, Desmond Tutu TB Centre, Stellenbosch University, Cape Town, South Africa; 2 Zambart, Lusaka, Zambia; 3 Department of Global Health and Development, LSHTM, London, United Kingdom

## Abstract

**Introduction:**

The history of HIV in southern Africa is interwoven with faith. Faith-based institutions and prominent faith leaders were often key in championing HIV rights. Community-based care networks were often underpinned by faith-based principles of altruism and care. Conversely, faith institutions may also be associated with harmful moralizing and sexual conservatism linked to stigma and with a focus on abstinence and partner fidelity. With increasing ART availability, faith is sometimes now equated with “alternative health beliefs” that hinder uptake and adherence.

**Methods:**

Between December 2012 and May 2013, we conducted research to describe the HIV landscape in 9 study communities in the Western Cape, South Africa. In each study community, we spent approximately 10 days conducting semi-structured observations, group discussions (48, participants=232) and interviews (32) with residents and health service stakeholders (some also faith leaders). We present a narrative analysis of how HIV is used in community discussions in relation to interpreting “faith.”

**Results:**

Over the course of data collection participants often used this HIV research as an opportunity to talk about faith. A core faith-based dilemma underpins the narratives which we interpret as “charity *versus* justice.” On the one hand, HIV requires people to act charitably – holding empathetic attitudes, showing community solidarity and acting selflessly. When describing this response, participants often also drew on wider post-colonial, post-*Apartheid* narratives of returning to cherished cultural values of inclusivity. On the other hand, HIV is also often aligned with stigmatized (or otherwise morally marginalized) social groups. In this positioning, faith-based values of chastity, being held accountable to moral choices, and penance for sin are called upon to justify marginalizing people living with HIV. Several faith-based leaders circumvented this dilemma by drawing a distinction between matters of the body (like HIV) and soul. This distinction enabled discussion of experiences of living with HIV and sexual morality on separate registers (empathy *and* justice).

**Conclusions:**

Faith is a double-edged sword for HIV interventions, both preaching charity and being used to justify alienating individuals who are deemed responsible for acquiring HIV. Attempts to re-interpret this narrative (to facilitate faith-based participation in health messaging) should be further investigated.

# Sub article: WEPDD0105 Capacity building of traditional health practitioners to mainstream HIV and AIDS prevention in Zimbabwe

**Published:** 2016-07-22

**Authors:** F Dube, M Dube, A Mpofu, T Magure, I Taramusi

**Affiliations:** National AIDS Council, Harare, Zimbabwe

## Abstract

**Introduction:**

The purpose of this project was to strengthen the capacity of traditional health practitioners (THPs) in HIV and AIDS prevention and treatment. The National AIDS Council spearheaded the local implementation of the project, which was simultaneously implemented in South Africa and Botswana, funded by the SADC. Specifically, the project sought to train 360 THPs in HIV prevention and treatment in Zimbabwe in 2 years.

**Description:**

A three-member project steering committee, comprising NAC, Ministry of Health and Child Care and a representative of the THPS, was established. After this, an all-inclusive stakeholders consultative meeting to sensitize them on the objectives of the project and obtain their buy-in was held. A manual was then developed and translated into Ndebele and Shona. Six people, including three THPS, were trained as facilitators.

A total of 296 THPs were trained in five provinces. At the end of the SADC funded project, the National AIDS Council adopted the project and has trained an additional 180 THPs. During the training, most THPs confirmed claims that condoms contain HIV causing worms and that the THPs could treat AIDS. These claims and others were demystified during training. Forty percent of the trained THPs underwent HIV counselling and testing, provided during training. In addition, condoms were provided to the THPs to dispense to their clients at home. One hundred healers have been followed up 12 months after training and 90% of them found to be practising what they learnt, in particular referring clients for HIV counselling and testing, record keeping and art adherence counselling.

**Lessons learned:**

- Most THPs have serious misconceptions about causes of HIV and that they treat AIDS.
- The misconceptions are due to lack of knowledge on the causes of HIV and its management.
- THPs are willing to collaborate with mainstream health care as long as this does not lead to loss of income.
- Engaging THPs should not discredit their work but amplify it.
- THPS can become good partners in responding to HIV and AIDS.

**Conclusions/Next steps:**

NAC continues to fund this intervention to ensure all provinces are covered and also monitor post course behaviour.

# Sub article: WEPDD0106 Developing an ethical indigenous research protocol: sowing seeds for the inclusion of indigenous peoples with HIV

**Published:** 2016-07-22

**Authors:** CDP Montalvo Pacahuala, T Stratton, R Masching, A King, C Aspin

**Affiliations:** 1 Investigación, International Indigenous Working Group on HIV & AIDS (IIWGHA), Lima, Peru; 2 Coordination, International Indigenous Working Group on HIV & AIDS (IIWGHA), Vancouver, Canada; 3 Research, Canadian Aboriginal AIDS Network, Halifax, Canada; 4 Faculty of Health Sciences, Simon Fraser University, Burnaby, Canada; 5 Dentistry, Royal University of Phnom Penh, Phnom Penh, Cambodia

## Abstract

**Introduction:**

Indigenous responses to HIV/AIDS have been underway for over two decades. The International Indigenous Working Group on HIV and AIDS (IIWGHA) includes 17 Indigenous leaders from 5 continents, 13 countries and multiple Indigenous communities. IIWGHA is an international voice that links Indigenous Peoples with their leadership, governments, AIDS service organizations, cooperatives and others in collective action to reduce these health inequities. Robust responses to HIV/AIDS must be grounded in country and population-specific evidence generated through high-quality research. The distinct histories of research exploitation within our communities have led to distrust; therefore it is imperative that research becomes Indigenous-led. We are generating a meaningful engagement protocol for use within IIWGHA to ensure that research is conducted in a culturally respectful way, responsive to the diverse needs of IIWGHA members.

**Description:**

Our initiative responds to the dearth of Indigenous HIV-focused research. Our goal is to sow seeds for community-led research benefitting Indigenous populations affected by HIV. This protocol will strategically support Indigenous researchers implementing research meeting the highest standards of scientific excellence, and respectful and ethical community engagement, contributing to knowledge building and strengthening partnerships, which underpin and inform strategic action.

**Lessons learned:**

We ground ourselves in Indigenous knowledge and paradigms that inform our understandings of the world. We will also incorporate knowledge generated through the academy and apply Two-Eyed Seeing and community-based research frameworks. Mutually respectful, ethical partnerships address socioeconomic and structural health determinants. Decolonizing methodologies and processes are central themes in this protocol development and modalities of research partnerships. Key principles include: 1. Inclusion of Indigenous peoples throughout the research process; 2. Research in good faith, with free, prior and informed consent; 3. Evidence of vulnerability and risks; 4.Grounding research in the strengths, cultures and ancestral practices of Indigenous peoples; 5. Honouring both Indigenous and western ways of knowing.

**Conclusions/Next steps:**

This work brings together Indigenous HIV research networks from IIWGHA member countries for a constructive and critical look at Indigenous HIV research. Next steps will include finalizing a research protocol for use nationally and internationally by members of IIWGHA and its collaborators and funders.

# Sub article: WEPDE0101 Scaling up partner testing in maternal and child health clinic settings: a case study of Gucha Sub-County Hospital, western Kenya

**Published:** 2016-07-22

**Authors:** J Nyakerario Omare, A Kituku

**Affiliations:** Care International, Health Sector, Kisii, Kenya

## Abstract

**Introduction:**

Male partner involvement is generally low in maternal and child health clinic (MCH) settings due to a myriad of factors including lack of policy guidance and socio-cultural factors. The objective of this study was to assess male involvement in PMTCT services, including partner counselling and testing in the MCH and to gather recommendations for improvement and strengthening of these services.

**Description:**

Two mentor mothers were trained in mid-2013 and placed at Gucha Sub-county hospital, Western Kenya to support PMTCT interventions at the facility, including encouraging partner testing among women receiving PMTCT services. All women attending 1st ANC services were counselled on the importance of partner involvement and testing, provided with an invitation card for the partner to accompany them to the clinic for their next ANC visit. Couples who attended clinic were provided with a variety of services including HIV counselling and testing. Community mobilization and sensitizations were done around the facility catchment on importance of partner involvement and testing. Routinely collected data for partner counselling and testing uptake from MCH registers was compared before (2012) and after placement of the mentor mothers (2014).

**Lessons learned:**

A total of 1690 and 1619 pregnant women attended the facility for ANC services for the first time in 2012 and 2014, respectively. Forty-three percent (741/1690) and (97%) 1582/1619 were counselled and tested for HIV. Among these, 17/741(3%) partners were counselled and tested for HIV in 2012 as compared to 953/1582 (60%) in 2014. Among the partners tested, 0/17 (0%) were HIV discordant in 2012 while 14/953 (1.4%) were discordant in 2014. A total of 12 community sensitization meetings were held in 2014.

**Conclusions/Next steps:**

Scaling up partner testing is feasible but requires both facility and community level coordination. With good mobilization and community education, partner testing can be institutionalized. With improved partner testing, uptake of PMTCT interventions can be improved. There is need for County governments to put in place guidance of male involvement in RMNCH services.

# Sub article: WEPDE0102 Counselled to compliance: experiences of Option B+ for the prevention of mother to child transmission in four health and demographic surveillance sites in sub-Saharan Africa

**Published:** 2016-07-22

**Authors:** E McLean, J Renju, J Wamoyi, D Bukenya, W Ddaaki, K Church, A Wringe, ALPHA Network

**Affiliations:** 1 London School of Hygiene & Tropical Medicine, London, United Kingdom; 2 Malawi Epidemiology and Intervention Research Unit, Karonga, Malawi; 3 National Institute for Medical Research, Mwanza, United Republic of Tanzania; 4 MRC/UVRI Uganda Research Unit on AIDS, Entebbe, Uganda; 5 Rakai Health Sciences Program, Kalisizo, Uganda

## Abstract

**Introduction:**

Option B+ for the prevention of mother-to-child transmission (PMTCT) was intended to improve PMTCT coverage and retention in HIV care for mothers and virtually eliminate perinatal infections. Although Option B+, and wider test and treat policies, are to be rolled-out in many countries, little is known about how pregnant women and health workers perceive Option B+ and its influence on HIV care-seeking behaviours. This qualitative study explored these experiences in four African settings.

**Methods:**

Thirty in-depth interviews (IDIs) were conducted with HIV-positive women, purposefully sampled from ART clinics or health and demographic surveillance datasets in Karonga, (Malawi), Kisesa (Tanzania), Kyambuliwa and Rakai (Uganda). Twenty IDIs were conducted with health-care providers. IDIs explored health worker and patients’ understanding and experiences of Option B+. A framework analysis using a thematic approach was conducted, and findings compared across sites.

**Results:**

Health providers and women generally considered the main benefit of Option B+ as being to protect the unborn baby with few references to potential or expected health benefits of early ART for the mothers. Across all sites, health workers reported a desire to maximize opportunities to initiate HIV positive women onto ART through Option B+ programmes. Both providers and women reported instances of repeated counselling sessions until consent to initiate ART was obtained, particularly in women for whom the positive test result was new or unexpected. Some pregnant women responded to a perceived lack of autonomy over Option B+ participation by covertly refusing to adhere to ART, while others avoided antenatal clinics completely.

**Conclusions:**

Most women willingly initiated ART through Option B+, however there was an occasional disconnect between health worker actions, and the “readiness” of women to start lifelong treatment resulting in practices that could be perceived as coercive. This could be further exacerbated by the perceived requirements among some health workers to meet Option B+ programme targets. ART initiation following an HIV diagnosis should be accompanied by greater efforts to ensure preparedness for life-long ART. These findings may also be relevant to guiding the implementation of universal test and treat policies.

# Sub article: WEPDE0103 MomConnect: MHealth strengthening of demand and supply sides of the South African health system to improve PMTCT

**Published:** 2016-07-22

**Authors:** P Barron, Y Pillay, J Sebidi, A Fernandes

**Affiliations:** 1 School of Public Health, University of the Witwatersrand, Johannesburg, South Africa; 2 National Department of Health, Pretoria, South Africa

## Abstract

**Introduction:**

MomConnect is a South African National Department of Health initiative that aims to use the ubiquity of cell phones. It sends pregnant women messages appropriate to their stage of pregnancy to strengthen the **demand** for health services and empower women. It enables these women to interact with the health system, obtain further health information and to provide feedback on the quality of care that they receive to improve supply of services.

**Description:**

Since its launch in August 2014 MomConnect has registered 583,929 pregnant women. More than 95% (34,887) of all facilities dealing with pregnant women have recorded MomConnect registrations, indicative of its universal roll out.

**Lessons learned:**

Women interact free of charge with a help desk. Over 200,000 questions have been asked. Examples related to HIV include “Why are health care workers initiating ARV treatment without CD4 Count”; “What is the safest method of delivery if you are HIV positive”; “What are the chances of the baby getting infected with the virus when one has cracked nipples should breastfeeding continue?” Each question is answered on a daily basis and if serious women are directed to a health facility. Women also have the ability to compliment the service. To date, 4173 compliments received more than six times the complaints (690) received. Examples of complaints impacting on PMTCT include:

“Lack of confidentiality for HIV Patients, as files were labelled, consulting rooms designated only for HIV patients, treatment room labelled ARV room.” The resultant action was that the district focal person visited facility and met the manager. They looked at areas that patient complained and removed all the signs related to HIV.

“Complained that she went to the clinic to collect HIV treatment and she was sent off without medication.” Client advised to return to facility, where she received the necessary ARVs after managers of facility were briefed.

**Conclusions/Next steps:**

MomConnect has empowered pregnant women and improved the demand for better quality as well as improving supply and the quality of health services. The help desk is being upgraded to be more responsive to feedback from pregnant women. MomConnect data are being integrated with national data system.

# Sub article: WEPDE0104 Viral load sample logistics for HIV-positive women in rural settings: experience from the INSPIRE MoMent Nigeria PMTCT study

**Published:** 2016-07-22

**Authors:** O Adeyemi, S Erekaha, E Ogum, F Yunusa, H Swomen, A Barde, C Chime, I Ebagua, NA Sam-Agudu

**Affiliations:** 1 Clinical Department, Institute of Human Virology Nigeria, Abuja, Nigeria; 2 London School of Hygiene and Tropical Medicine, London, United Kingdom; 3 Molecular Laboratory, Institute of Human Virology Nigeria, Abuja, Nigeria; 4 Department of Epidemiology and Prevention, Institute of Human Virology, University of Maryland Baltimore, Baltimore, MD, United States

## Abstract

**Introduction:**

Due to its earlier detection of treatment failure, viral load (VL) is preferred over CD4 count in treatment monitoring. In Nigeria, VL is not routinely available to rural patients at primary healthcare centres (PHCs). Whole blood samples require transport on ice and processing within 6 to 8 hours. Routine sample transport is difficult in rural areas due to distance and terrain. We present our experience in the establishment of a rural VL transport logistics system in North-Central Nigeria.

**Description:**

The MoMent study evaluates PMTCT service uptake, ARV adherence and retention among mother-infant pairs in rural areas. VL is a study proxy for maternal ART adherence, and in order to measure this outcome, a VL sample transport system had to be developed *de novo*. Field Research staff were trained with a VL sample collection and transport SOP alongside clinical PHC staff. Study sites were mapped to the Central Lab; sites too distant were linked to a hub facility for sample centrifugation and storage before delivery to the central processing lab. Sample collection and transport materials were provided to each study site. Where official vehicles were not available, commercial transport was costed and utilized.

**Lessons learned:**

In total, 28 staff received 1-day training on VL sample collection and transport. In 13 months, 201 maternal VL samples were collected and transported from 20 PHCs to the Central Lab; 28/201 (13.9%) were transited through the hub. Overall, 4/201 (1.9%) samples were rejected, comparable to rejection rates from secondary and tertiary clinics; major reason being sample lysis. Average collection, transport and processing cost per VL sample was 55.50 USD; rejected samples cost 57.50 USD to repeat. High turnover rate of trained PHC staff (7/17, 41.2%) was also observed.

**Conclusions/Next steps:**

Routine VL in rural Nigeria is feasible if resources can be directed at training, staff retention and quality assurance. Additionally, we recommend mapping of facilities and strategic placement of molecular labs or point-of-care testing to reduce transport cost and logistic challenges. However, processing cost remains high, especially for rejected samples. Innovative financing and technology is needed to provide equitable access to VL monitoring for HIV-positive women in hard-to-reach areas.

# Sub article: WEPDE0105 Early retention in antenatal care among HIV-positive women enrolled in the Option B+ programme in Kinshasa, DRC

**Published:** 2016-07-22

**Authors:** J Ditekemena Dinanga, MM Gill, A Loando, C Nyombe, J Bakwalufu, N Mbonze, F Fwamba, V Ilunga, R Machekano

**Affiliations:** 1 Elizabeth Glaser Pediatric AIDS Foundation (EGPAF), MSA, Kinshasa, Democratic Republic of the Congo; 2 Elizabeth Glaser Pediatric AIDS Foundation - Global, Washington, DC, United States; 3 Kinshasa University School of Public Health, Kinshasa, Democratic Republic of the Congo; 4 Programme National de Lutte Contre le Sida, Kinshasa, Democratic Republic of the Congo

## Abstract

**Introduction:**

Effective retention in prevention of mother-to-child HIV prevention (PMTCT) programmes implementing universal, lifelong treatment (“Option B+”) is critical to achieving paediatric HIV elimination. Innovative strategies are needed to strengthen retention in PMTCT/antenatal care (ANC). The Elizabeth Glaser Pediatric AIDS Foundation (EGPAF) assessed early loss to follow-up of HIV-positive pregnant women in ANC following introduction of a standard operating procedure (SOP) in select facilities in Kinshasa, Democratic Republic of the Congo. The SOP included guidance to health providers and mentor mothers (HIV-positive expert patients) on 1) linking newly and known HIV-positive women to these mothers, 2) counselling at first ANC, 3) tracking those who miss antenatal appointments through phone calls and home visits and 4) documenting appointments and follow-up activities.

**Methods:**

A quasi-experimental study was conducted from May to November 2015 in 16 EGPAF-supported health facilities, purposively selected for high volume and high HIV prevalence. Facilities were randomized to receive the SOP enhancement or no intervention. All records of HIV-positive women who attended their first ANC visit were abstracted during the data collection period. Multiple logistic regressions were used to identify determinants of second ANC visit attendance by HIV-positive pregnant women enrolled in the PMTCT Option B+ programme.

**Results:**

One-hundred seventy-four women were included in the analysis: 43.7% (n=76) in the intervention and 56.3% (n=96) in the comparison group. Women's average age was 31 years (SD: 6.4). Approximately 86.2% of participants were assessed as WHO Clinical Stage I. Overall attrition at the second ANC visit was 25.8% (n=45). After multivariable logistic regression, being in the comparison group remained independently associated with early attrition (AOR=3.49, 95% CI: 1.58–7.71, p=0.002). Women attending facilities without SOP implementation were 3.5 times more likely to miss the second ANC visit (n=35, 35.7%) compared to the women from the intervention group (n=10, 13.2%).

**Conclusions:**

Study findings demonstrated a positive effect of the SOP intervention on second ANC visit attendance. This SOP should be expanded to include the full range of ANC visits and delivery. This tool should be promoted and scaled up to contribute to the improvement of the retention in care for PMTCT clients.

# Sub article: WEPDE0106LB Self-reported antenatal adherence to predict postnatal viral rebound among women initiating ART during pregnancy in Cape Town, South Africa: a prospective study

**Published:** 2016-07-22

**Authors:** T Phillips, K Brittain, EJ Abrams, A Zerbe, A Ronan, CA Mellins, RH Remien

**Affiliations:** 1 Division of Epidemiology and Biostatistics, University of Cape Town, Cape Town, South Africa; 2 ICAP-Columbia University, Mailman School of Public Health, New York, NY, United States; 3 College of Physicians & Surgeons, Columbia University, New York, NY, United States; 4 HIV Center for Clinical and Behavioral Studies, Columbia University, New York, NY, United States; 5 New York State Psychiatric Institute, New York, NY, United States

## Abstract

**Introduction:**

Maintaining postnatal viral suppression is critical to minimize risk of breastfeeding mother-to-child transmission and ensure ongoing maternal health. Determining simple ways to identify women at risk of postnatal viraemia will have benefits for both mother and child.

**Methods:**

HIV+ women initiating antiretroviral therapy (ART) during pregnancy at a large primary care clinic were recruited and followed in the MCH-ART study in Cape Town, South Africa. Consenting women completed up to eight study visits from ART initiation through 12 months postpartum, including viral load (VL) measurement, demographics and self-report of missed ART doses in the previous 30 days. We investigated time to VL >1000 copies/ml and associations between antenatal adherence and viral rebound among women suppressed at delivery.

**Results:**

Overall, 339 women with VL ≤50 copies/ml at delivery were included in this analysis (median age 28 years, median 18 weeks on ART). From ART initiation through delivery, 28% of women reported any missed ART doses; 16% reported one or more missed doses/month on average. Using product limit methods 79% of women maintained VL ≤1000 copies/ml at 12 months postpartum. In a proportional hazards model adjusted for age, duration of antenatal ART and ART history, reporting one or more missed doses per 30 days on average during pregnancy was associated with a more than twofold increase in the hazard of postnatal viral rebound (adjusted hazard ratio [aHR] 2.64, p < 0.001). Previous ART use increased the hazard of viral rebound; increasing age and weeks on ART were protective. When stratified by age, the association between missed doses and viral rebound was stronger among women ≥25 years compared to younger women (aHR 3.88 and 2.20, respectively).

**Conclusions:**

In this cohort of women who initiated ART in pregnancy and were suppressed at delivery, report of antenatal missed ART doses was predictive of postnatal viraemia. Self-reported antenatal missed doses, together with other routinely collected antenatal risk factors like age, could be used to flag women at high risk of postnatal viral rebound. There is a need for further research to explore how reported antenatal adherence could be used in low-resource settings to target interventions to recently postpartum women most at risk.

# Sub article: WEPDE0201 Community leader engagement and peer group attendance improves selected MCH and PMTCT services uptake and retention: preliminary findings from project ACCLAIM

**Published:** 2016-07-22

**Authors:** G Woelk, MP Kieffer, D Mpofu, R Cathcart, ACCLAIM Study Group

**Affiliations:** 1 Elizabeth Glaser Paediatric AIDS Foundation, Washington, DC, United States; 2 Elizabeth Glaser Paediatric AIDS Foundation, Lilongwe, Malawi; 3 Elizabeth Glaser Paediatric AIDS Foundation, Mbabane, Swaziland

## Abstract

**Introduction:**

Project advancing community level action for improving maternal and child health (MCH)/prevention of mother-to-child HIV transmission (PMTCT) (Project ACCLAIM), a three-arm randomized trial with 45 PMTCT-implementing health facilities and their catchment areas across Swaziland, Uganda and Zimbabwe, aimed to improve access, uptake and retention in MCH and PMTCT services.

The study evaluates three interventions:

Arm 1) Community leader (CL) engagement (training in MCH/PMTCT, community action mentoring including dialogues;

Arm 2) CL plus community days (CDs), a community event with structured dialogues on MCH/PMTCT and provision of health services;

Arm 3) CL plus CDs and male and female MCH classes: four structured peer-led sessions. We report preliminary results on outcomes of increased proportions of HIV exposed infants (HEI) receiving HIV testing at 6–8 weeks, health facility deliveries and male partners tested.

**Methods:**

Routine health facility data were collected prior to implementation (July 2013, Swaziland and Zimbabwe, January 2014, Uganda) and for each quarter through June 2015. We compared changes in proportions pre-implementation and the last quarter after implementation in the three arms using chi square tests for linear proportions.

**Results:**

The interventions’ effects differed in the three countries. In Uganda, the proportion of HEI tested increased from 31% (56/182) to 48% (56/116), p<0.001 in Arm 1, and in Arm 3 from 19% (20/106) to 43% (22/51), p<0.001; male partners tested increased from 11% (224/2067) to 22% (533/2475) p<0.001 in Arm 1 and 10% (71/728) to 15% (119/797) in Arm 3, p<0.001. The proportion of women delivering in health facilities increased from 60% (1252/2083) to 94% (1694/1797) p<0.001, Arm 1. In Swaziland, the proportions of women delivering in a health facility increased in both Arm 1 and Arm 3 – 49% (160/325) to 81% (264/324) p<0.001 and 50% (100/199) to 78% (153/195), respectively, p<0.001. In Zimbabwe, the proportions of male partners tested increased in Arm 1 from 42% (66/159) to 73% (130/178), p<0.001.

**Conclusions:**

The CL and peer group interventions appeared to increase MCH/PMTCT services update and retention, with Uganda registering the most improvements. The CL plus CD intervention, Arm 2, appeared to have no effect on the outcomes.

# Sub article: WEPDE0202 The importance of involving young women living with HIV in sexual reproductive health research: International Community of Women Living with HIV Eastern Africa (ICWEA) experience

**Published:** 2016-07-22

**Authors:** B Azizuyo

**Affiliations:** International Community of Women Living with HIV (ICW), Sexual Reproductive and Health Rights, Kampala, Uganda

## Abstract

**Introduction:**

Almost 60% of new HIV infection among young people aged 15–24 occur among adolescent girls and young women (2013). Globally, 15% of the women living with HIV are aged 15–24, of whom 80% live in sub-Saharan Africa. Although young women living with HIV can have a role in ensuring that research in sexual and reproductive health and rights (SRHR) is relevant to their needs, they have historically not been targeted as research assistants and the benefits and opportunities of involving them are not well documented.

**Description:**

The young women were engaged in multi stakeholder dialogue on SRHR violations. Experiences and lessons learnt involving women living with HIV as research assistants have been documented in a toolkit to inform future research. In April 2014, ICWEA involved young women living with HIV (15–30 years) in a research “Violation of Sexual Reproductive Health Rights of women living with HIV in clinical and community settings in Uganda.” ICWEA called for application for research assistants on list-servers and HIV civil society including youth-focused organizations in nine research focus districts. Thirty-five young women living with HIV were selected and underwent training in research methods, data collection and understanding SRHR. They participated in identifying research respondents, pre-tested research tools, collected and transcribed data, participated in report writing, validation and dissemination of findings. They were represented on the Advisory Board to the research.

**Lessons learned:**

The process enhanced the capacity of the young women to engage in research and dialogue on SRHR violations. The process built confidence in the respondents, because research assistants were also women living with HIV and some spoke out for the first-time on sensitive issues such as forced and coerced sterilization. The young women got in-depth understanding of SRHR information and services to inform their own advocacy.

**Conclusions/Next steps:**

Involving young women living with HIV as research assistants is beneficial for respondents of SRHR research and empowering to them. Population-specific recruitment and capacity building strategies with greater gender consideration for young women living with HIV are important to ensure their involvement in research processes. Their experiences should be documented and shared to inform future researchers.

# Sub article: WEPDE0203 “When you don't have money, he controls you”: financial security, community savings groups, and HIV risk among female sex workers in Iringa, Tanzania

**Published:** 2016-07-22

**Authors:** A Mantsios, C Shembilu, J Mbwambo, S Likindikoki, S Beckham, A Mwampashi, A Leddy, N Galai, W Davis, D Kerrigan

**Affiliations:** 1 Health, Behavior and Society, Johns Hopkins Bloomberg School of Public Health, Baltimore, MD, United States; 2 Psychiatry and Mental Health, Muhimbili University of Health and Allied Sciences, Dar es Salaam, United Republic of Tanzania; 3 Epidemiology, Johns Hopkins Bloomberg School of Public Health, Baltimore, MD, United States

## Abstract

**Introduction:**

There is a growing literature indicating the importance of financial insecurity as a structural driver of HIV risk behaviours such as unprotected sex including among female sex workers (FSW), who are at heightened risk for HIV infection across geographic settings.

**Methods:**

A Phase II randomized controlled trial of a community-empowerment based combination HIV prevention intervention is being conducted in Iringa, Tanzania. Using baseline survey data from 254 FSW of an ongoing cohort, bivariate and multivariate logistic regression was conducted to examine the statistical association between community savings group participation and HIV protective behaviours. Iterative, semi-structured in-depth interviews were also conducted with 15 FSWs participating in community savings groups. Interviews were audiotaped, transcribed, translated from Swahili into English, coded and analyzed through thematic content analysis.

**Results:**

Participants qualitatively described that immediate financial need inhibited their ability to refuse high-risk sexual behaviours such as sex without a condom and anal sex with clients. With insufficient capital to participate in formal banking, community savings groups were described as a mechanism through which FSW can securely save their money, and create a safety net they can utilize when they have immediate financial need, safeguarding against HIV risks. Quantitative analysis confirmed the women's qualitative narratives. Approximately 25% of the cohort participates in community savings groups. In multivariate analysis controlling for age, education, marital status, number of children, and length of time in sex work, participating in a community savings group was significantly associated with always using a condom with new clients (AOR 2.06; 95% CI: 0.98–4.32) and refusal of unsafe sex (AOR: 2.94; 95% CI: 1.33–6.49), including when a client was unwilling to use a condom, requested anal sex, or was unwilling to pay the requested price. Savings group participation was also significantly associated with a lower odds of having reported an STI in the last 6 months (AOR: 0.37; 95% CI: 0.16–0.84).

**Conclusions:**

Findings demonstrate the promising role of community savings groups as a structural intervention to promote financial security and reduce HIV risk among FSW.

# Sub article: WEPDE0204 Chasing the possible: are we there yet? Innovations in testing to end the HIV epidemic in NSW, Australia

**Published:** 2016-07-22

**Authors:** T Duck, C Selvey, B Telfer, HMA Schmidt, K Price, P Keen, ME Cecilio, J Holden

**Affiliations:** 1 New South Wales Ministry of Health, North Sydney, Australia; 2 Health Protection NSW, North Sydney, Australia; 3 ACON, Surry Hills, Australia; 4 Kirby Institute, UNSW, Kensington, Australia; 5 Centre for Population Health, New South Wales Ministry of Health, North Sydney, Australia

## Abstract

**Introduction:**

To achieve the ambitious targets for reducing HIV transmission set in the *NSW HIV Strategy 2012–2015*, NSW implemented a suite of innovations to facilitate increased HIV testing and better detect undiagnosed HIV infections among priority populations.

**Description:**

In 2013–2015, NSW:

- Integrated rapid HIV testing (RHT) into the mix of testing options for gay men at public clinics across NSW.
- Delivered RHT at community sites and mobile locations through peer educators to increase accessibility.
- Facilitated service redesign in public clinics to increase targeted testing delivery.
- Realigned purchasing arrangements with non-government organizations to support Strategy targets.
- Established a “real-time” monitoring and reporting framework to drive performance among key stakeholders.
- Delivered targeted marketing and communications activities, including the award-winning *Ending HIV* campaign.

**Lessons learned:**

From January to September 2015, HIV testing in laboratories increased by 7, 11 and 18% compared with January to September 2014, 2013 and 2012. From July to September 2015, there was a 68% increase in HIV testing at sexual health services among gay men compared with the same period in 2014. Self-reported HIV testing rates among gay men were the highest on record since 1996, with 76% surveyed in 2014 and 75% surveyed in 2015 reporting an HIV test within the last 12 months. From January to September 2015, there were 247 new HIV diagnoses in NSW; 7% less than the 2009–2014 average during the same period. Of these, 41% had evidence of early stage infection; less than the January to September 2009–2014 average of 46%.

**Conclusions/Next steps:**

The combination of increases in HIV testing, a decrease in new diagnoses count and evidence for reductions in early stage diagnosis suggest a reduction in HIV transmission. The diversity and range of programme efforts have contributed towards *NSW HIV Strategy* targets. However, further innovations are needed to meet the Strategy's goal of virtual elimination of HIV transmission by 2020, including the introduction of self-sampling testing to reach high-risk populations testing infrequently for HIV.

# Sub article: WEPDE0205 A cohort study of community-based test and treat for men who have sex with men and transgender women: preliminary findings from Thailand

**Published:** 2016-07-22

**Authors:** R Vannakit, J Jantarapakde, S Pengnonyang, S Jitjang, R Janamnuaysook, T Pankam, D Trachunthong, K Pussadee, R Reankhomfu, D Lingjongrat, S Janyam, T Nakpor, P Leenasirimakul, T Jadwattanakul, S Noriega, S Charoenying, T Sattayapanich, A Arunmanakul, P Phanuphak, M Cassell, N Phanuphak

**Affiliations:** 1 Office of Public Health, United States Agency for International Development, Bangkok, Thailand; 2 The Thai Red Cross AIDS Research Centre, Bangkok, Thailand; 3 Caremat Organisation, Chiang Mai, Thailand; 4 Rainbow Sky Association of Thailand, Bangkok, Thailand; 5 Service Workers In Group Foundation, Bangkok, Thailand; 6 Sisters Foundation, Chonburi, Thailand; 7 Nakornping Hospital, Chiang Mai, Thailand; 8 Queen Savang Vadhana Memorial Hospital, Chonburi, Thailand; 9 RTI International, Bangkok, Thailand; 10 FHI 360, Bangkok, Thailand

## Abstract

**Introduction:**

HIV prevalence is high among men who have sex with men (MSM) and transgender women (TG) in Thailand. We explored the feasibility of the community-based Test and Treat strategy conducted by community-based organization (CBO) staff, to provide early case identification and immediate antiretroviral therapy (ART), for MSM and TG.

**Methods:**

MSM and TG were recruited into an operational research cohort through five CBOs in Bangkok, Pattaya and Chiang Mai. Trained CBO staff provided same-day result HIV testing and sexually transmitted infection (STI) screening at baseline. At diagnosis, HIV-positive individuals had point-of-care CD4 count measurements and ART immediately offered. Data on demographic, risk behaviour, knowledge and attitudes towards HIV and ART were collected using self-administered questionnaires.

**Results:**

From May to November 2015, 1029 participants were enrolled (71% MSM and 29% TG). HIV prevalence was 17% (20% in MSM: 8% in TG). HIV-positive participants were more likely to

- have never had HIV testing (72% vs. 42%, p<0.001);
- have been screened positive for STIs (58% vs. 29%, p<0.001);
- perceive themselves to be moderate to high risk for HIV (62% vs. 48%, p=0.001);
- had unprotected sex in the past 6 months (87% vs. 78%, p=0.01);
- have used amphetamine-type stimulants in the past 6 months (12% vs. 7%, p=0.02); and
- have low knowledge on HIV transmission routes (p=0.007).

Overall, 39% knew that ART could reduce HIV risk for their partners. Among 172 HIV-positive, 141 (82%) accepted immediate ART while 31 declined or did not start ART after more than 2 weeks of diagnosis. Binary logistic regression identified being sex workers or unemployed (OR: 0.40, 95% CI: 0.16–0.98, p=0.046) and having used illicit drugs in the past 6 months (OR: 0.26, 95% CI: 0.11–0.63, p=0.003) to be factors associated with unsuccessful ART initiation.

**Conclusions:**

Implementing the community-based test and treat strategy by trained CBO staff is feasible in Thailand. Overall acceptance and success of immediate ART initiation were high. Additional efforts are needed to more effectively target HIV-positive MSM and TG who are unemployed, sex workers or using drugs in order to strengthen linkages to care and treatment.

# Sub article: THPDA0101 Immuno-PET/CT imaging reveals differences in virus and CD4^+^ cell localization in SIV-infected rhesus macaques treated with an anti-α4β7 mab

**Published:** 2016-07-22

**Authors:** J Arthos, S Byrareddy, C Cicala, K Ortiz, D Little, S Gumber, JJ Hong, C Zurla, F Villinger, A Fauci, A Ansari, P Santangelo

**Affiliations:** 1 National Institutes of Health (NIH), Bethesda, MD, United States; 2 Department of Pharmacology and Experimental Neuroscience, University of Nebraska Medical Center, Omaha, NE, United States; 3 Department of Pathology, Emory University School of Medicine, Atlanta, GA, United States; 4 The Yerkes National Primate Research Center, Atlanta, GA, United States; 5 Georgia Institute of Technology, Atlanta, GA, United States; 6 New Iberia Research Center, University of Louisiana, Lafayette, LA, United States

## Abstract

**Introduction:**

Integrin α4β7 mediates the trafficking of leukocytes including CD4^+^ T cells to lymphoid tissues in the gut, which are principal sites of HIV and SIV replication during acute infection.

**Methods:**

We evaluated the impact of an anti-integrin α4β7 mAb, which acts as an α4β7 antagonist, on the distribution of both SIV-infected cells and CD4^+^ cells in rhesus macaques infected with highly pathogenic SIV on live animals using a PET/CT imaging technique. Probes for both the gp120 envelope protein and the CD4 receptor were employed.

**Results:**

We determined that the anti α4β7 mAb reduces viral replication in gut associated lymphoid tissues but also in other tissues including lung, spleen and axillary and inguinal lymph nodes that are not linked to α4β7-mediated homing. The reduction in viral replication in gut tissues occurred despite the fact that α4β7 mAb treatment did not deplete gut tissues of CD4^+^ cells. Treatment with anti α4β7 during the acute phase of infection appeared to facilitate CD4^+^ cell reconstitution at a later stage of infection.

**Conclusions:**

These results demonstrate that an α4β7 antagonist can reduce viral replication in gut tissues without depleting those tissues of CD4^+^ cells. Because damage to the gut is believed to play a central role HIV pathogenesis, these results support further evaluation of α4β7 antagonists in the study and treatment of HIV disease.

# Sub article: THPDA0102 HIV persists in colon and blood CCR6+CD4+ T cells during ART

**Published:** 2016-07-22

**Authors:** A Gosselin, TR Wiche Salinas, D Planas, VS Wacleche, Y Zhang, R Fromentin, V Mehraj, N Chomont, J-P Routy, P Ancuta

**Affiliations:** 1 CHUM Research Centre, Montreal, Canada; 2 Université de Montréal, Montreal, Canada; 3 McGill University Health Centre, Montreal, Canada

## Abstract

**Introduction:**

Peripheral blood CD4+ T cells expressing the Th17 marker CCR6 are highly permissive to HIV. Retinoic acid (RA), a gut-homing inducer, increases HIV integration/replication only in CCR6+ T cells; this further supports the role of the gut in HIV disease progression. Herein, we investigated the contribution of CCR6+ T cells from the colon and blood to viral persistence during antiretroviral therapy (ART) and tested the ability of RA to reactivate latent HIV in these cells.

**Methods:**

Experiments were performed on matched blood/colon biopsy samples (n=7) and PBMC collected by leukapheresis (n=14) from chronically HIV-infected subjects aviremics under ART. Cells were extracted from colon biopsies by enzymatic digestion; then, memory CCR6+/CCR6- CD4+ T cell subsets were sorted by flow cytometry (BD AriaIII). Blood total (T_M_, CD45RA-), effector (T_EM_, CD45RA-CCR7-) and central memory (T_CM_, CD45RA-CCR7+) CD4+ T cells expressing or not CCR6 and producing cytokines (IL-17A or IFN-g) were also sorted by flow cytometry. Integrated HIV genomes were quantified by real-time PCR. HIV reactivation was induced by stimulation with CD3/CD28 Abs in the presence or absence of RA (10 nM) and/or ART for 9 days. Levels of HIV-RNA in cell-culture supernatants were quantified by real-time RT-PCR.

**Results:**

Memory CCR6+ compared to CCR6- T cells isolated from the blood and colon biopsies were highly enriched in integrated HIV-DNA (median blood: 2298 vs. 830, p=0.0041; median colon: 2484 vs. 1463 HIV-DNA copies/10^6^ cells; p=0.01). Among the T_M_ pool in the blood, CCR6+ T_CM_ showed the highest levels of integrated HIV-DNA. Upon TCR triggering, HIV reactivation was preferentially observed in blood CCR6+ compared to CCR6- T_M_, T_CM_, and T_EM_; exposure to RA further induced HIV reactivation in CCR6+ T cells, including cells producing IL-17A.

**Conclusions:**

We identified CCR6 as a marker for CD4+ T cells enriched in HIV reservoirs in the blood and the colon of ART-treated subjects and demonstrated that HIV-DNA persists in IL-17-producing cells. The finding that RA promotes HIV latency reversal in a TCR-dependent manner indicates an important contribution of the intestinal environment to viral persistence and reactivation. Understanding molecular mechanisms of HIV persistence in Th17 cells will be critical for the design of therapeutic strategies aimed at viral eradication.

# Sub article: THPDA0103 CD8^+^ cytotoxic T lymphocytes exert a strong cytolytic effect on virally-infected cells prior to viral integration in SIVmac251-infected rhesus macaques

**Published:** 2016-07-22

**Authors:** B Policicchio, C Xu, D Ma, T He, K Raehtz, R Sivanandham, A Kleinman, N Krampe, G Haret-Richter, T Dunsmore, C Apetrei, I Pandrea, R Ribeiro

**Affiliations:** 1 Center for Vaccine Research, University of Pittsburgh, Pittsburgh, PA, United States; 2 Infectious Diseases and Microbiology, University of Pittsburgh, Pittsburgh, PA, United States; 3 Pathology, University of Pittsburgh, Pittsburgh, PA, United States; 4 Microbiology and Molecular Genetics, University of Pittsburgh, Pittsburgh, PA, United States; 5 Theoretical Biology and Biophysics, Los Alamos National Laboratory, Los Alamos, NM, United States

## Abstract

**Introduction:**

The mechanism of action of CD8^+^ T cells in controlling virus during HIV infection is not completely elucidated. Previous experiments using nucleoside reverse transcriptase inhibitors (NRTIs) in CD8-depleted SIV-infected macaques indicate that this mechanism is non-cytolytic. However, it is uncertain if CD8^+^ T cells have the ability to eliminate infected cells prior to virus integration. To answer this question, we analyzed the effects of integrase inhibitor raltegravir (RAL) monotherapy on infection outcome in SIV-infected rhesus macaques (RMs) either with or without CD8^+^ T cells.

**Methods:**

Eleven SIVmac251-infected RMs were given either RAL, the CD8-depleting antibody M-T807R1, or a combination of both and were followed for 23 days, at which point RAL was interrupted. Plasma viral loads (VLs) were monitored with conventional qRT-PCR. Changes in T-cell counts and immune activation were monitored by flow cytometry.

**Results:**

The CD8 depletion only group exhibited a plasma VL increase of ~1 log. RMs receiving RAL exhibited a decline in VL, with the CD8 depletion plus RAL group showing both a smaller decay in plasma virus compared to the RAL only group and a slower secondary VL decline. We fitted a heuristic model with double exponential decay to the first 10 days of VL decline, during complete CD8 depletion. The results showed that the very early phase of decay (<3 days) occurred at the same rate in the CD8-depleted and non-depleted groups (rate ~1/day). However, the next phase of decay was much slower in the CD8 depleted group (~0.015/day) than in the non-depleted group (~0.12/day). Moreover, this model fitted the data significantly better (p=0.045) than a model with the same second phase rate for both groups of RMs. These results are consistent with a dynamical model in which the first rate of decay corresponds to the death of productively infected cells and the second rate of decay corresponds to the loss rate of infected cells prior to provirus integration.

**Conclusions:**

Use of RAL monotherapy revealed a potential cytolytic effect of CD8^+^ T cells in SIV infection. Our results indicate that almost 90% of the death/loss rate of infected cells prior to proviral integration is due to CD8^+^ T cells.

# Sub article: THPDA0104 Understanding the effects of latency reversing agents on HIV RNA splicing: implications for latency reversal

**Published:** 2016-07-22

**Authors:** TM Mota, G Khoury, JC Jacobson, HK Lu, F Wightman, RM Van der Sluis, JL Anderson, PU Cameron, SR Lewin, DFJ Purcell

**Affiliations:** 1 Doherty Institute for Infection and Immunity, University of Melbourne, Melbourne, Australia; 2 Department of Infectious Diseases, Alfred Health and Monash University, Melbourne, Australia

## Abstract

**Introduction:**

Latency reversing agents (LRAs) are currently under investigation as part of a strategy for an HIV cure. Histone deacetylase inhibitors (HDACi) increase transcription of unspliced (US) HIV RNA but have limited potency *in vivo*. We investigated whether this may arise from impaired splicing of HIV RNA. We determined the effect of LRAs on HIV RNA splicing in the presence and absence of HIV Tat protein, which impacts splicing alongside transcription.

**Methods:**

We employed an LTR-driven splicing reporter construct where HIV Envelope gp140 is fused to eGFP and is expressed if the RNA transcribed from the reporter remains unspliced. If the RNA is spliced across the naturally occurring splice donor-4 and acceptor-7 within *env*, then a Rev mutant fused to dsRed is expressed instead. This system therefore allows the detection of unspliced and spliced RNA products through the measurement of eGFP or dsRed via flow cytometry. HEK293T cells were transfected with the reporter construct with or without a Tat expression plasmid and treated with a panel of LRAs. Fluorescence was assessed and paired T-tests were performed.

**Results:**

In cells transfected with the splicing reporter, treatment with romidepsin, panobinostat, JQ1 and PMA/ionomycin significantly increased the expression of unspliced product compared to DMSO in the absence of Tat (4.3, 1.6, 2.9, 6.8 fold change (FC) respectively, all p<0.05) and in the presence of Tat (1.5, 1.7, 1.5, 1.2, 1.7 FC respectively, all p<0.05). Interestingly, without Tat only JQ1 and PMA/ionomycin significantly increased the expression and proportion of spliced product (dsRed/ (dsRed+eGFP)) (4.9, 4.6 FC respectively, both p<0.05). Conversely, although HDACi had no effect on the level of spliced product without Tat, when Tat was added, a significant reduction in the proportion of spliced product was observed following treatment with vorinostat, romidepsin and panobinostat compared to DMSO (0.54, 0.62, 0.76 FC respectively, all p<0.05).

**Conclusions:**

HDACi reduce the efficiency of HIV RNA splicing in the absence or presence of Tat. This was not seen with other LRAs including JQ1 and mitogen. In addition to the effect of LRAs on HIV transcription, we propose that an assessment of RNA splicing should also be evaluated.

# Sub article: THPDA0105 Real time imaging of HIV uncoating in living cells

**Published:** 2016-07-22

**Authors:** TJ Hope, J Mamede

**Affiliations:** 1 Feinberg School of Medicine, Northwestern University, Chicago, IL, United States; 2 Department of Cell and Molecular Biology, Northwestern University, Chicago, IL, United States

## Abstract

**Introduction:**

Following the viral fusion of HIV with the cell membrane, the HIV genome is delivered into the cytoplasm within a fullerene cone-like structure known as the capsid. The capsid is composed of the viral proteins p24CA, and at some point, is separated from the reverse transcribing HIV genome in a process known as uncoating. The timing of the uncoating event remains controversial with some models proposing that the conical capsid structure is lost early while other models suggest that the intact capsid structure docks at the nuclear pore. The lack of data for kinetics and localization of uncoating has led to intense discussions over recent years.

**Methods:**

To develop a cell biology approach to visualize dynamic changes in capsid integrity and composition, we utilized GFP as a fluid phase marker intravirion marker. With this technique, the loss of the fluid phase GFP occurs in two steps, with fusion and upon the loss of the capsid core integrity. Live-cell microscopy of dual labelled virions allows for the moment of fusion and capsid integrity loss to be timed, thereby revealing the kinetics, localization and composition of HIV-1 early steps of infection.

**Results:**

Direct observation of individual virions reveals that the time between fusion and uncoating for both HIV envelope and VSVg-mediated fusion in tissue culture and primary cells (macrophages and T cells), is approximately 30 minutes. Uncoating occurs entirely in the cytoplasm. Inhibition of reverse transcription or RNAseH activity delays uncoating. Quantification of p24CA reveals that the majority of p24CA is lost when the capsid integrity is disrupted revealing uncoating happens on a minute time scale. Long-term imaging experiments at less than one particle per cell reveals that the early uncoating particles are associated with productive infection.

**Conclusions:**

Our observations demonstrate that uncoating occurs approximately 30 minutes after fusion in primary cells and transformed tissue culture cells. The newly developed ability to follow HIV at the single particle level and demonstrate that specific virion behaviour is associated with productive infection opens up many opportunities to define and characterize the earliest steps of the HIV lifecycle and how the virus interacts with innate host defences.

# Sub article: THPDB0101 Comparison of neurodevelopmental outcomes between HIV-exposed uninfected infants versus HIV-unexposed infants

**Published:** 2016-07-22

**Authors:** J Leidner, P Williams, G Mayondi, G Ajibola, P Holding, V Tepper, S Nichols, M Diseko, J Magetse, M Sakoi, K Moabi, J Makhema, C Mdluli, S Chaudhury, C Petlo, H Jibril, B Kammerer, S Lockman

**Affiliations:** 1 Goodtables Data Consulting, Norman, OK, United States; 2 Harvard AIDS Institute, Harvard TH Chan School of Public Health, Boston, MA, United States; 3 Department of Biostatistics, Harvard TH Chan School of Public Health, Boston, MA, United States; 4 Botswana Harvard AIDS Institute Partnership for Research and Education, Gaborone, Botswana; 5 Independent Consultant, Boston, MA, United States; 6 Department of Pediatrics, University of Maryland School of Medicine, Baltimore, MD, United States; 7 Department of Neurosciences, University of California, San Diego, CA, United States; 8 Department of Epidemiology, Harvard TH Chan School of Public Health, Boston, MA, United States; 9 Ministry of Health Botswana, Gaborone, Botswana; 10 Department of Psychiatry, Boston Children's Hospital, Boston, MA, United States; 11 Brigham and Women's Hospital, Boston, MA, United States

## Abstract

**Introduction:**

The effect of perinatal HIV- and antiretroviral (ARV)-exposure on neurodevelopment in HIV-exposed uninfected (HEU) children remains unclear. We prospectively compared neurodevelopmental outcomes in HEU versus HIV-unexposed (HU) children at 24 months of age.

**Methods:**

We enrolled HIV-infected and HIV-uninfected mothers (during pregnancy or 1 week post-partum) and their babies in the prospective observational Tshipidi study in two sites in Botswana (1 urban and 1 rural) from May 2010 to July 2012. Liveborn infants and their mothers were followed for 24 months postpartum, with socio-demographics and health data collected at entry and at 6 month intervals. Neurodevelopmental outcomes were assessed at 24 months of age with an adapted version of Bayley Scales of Infant and Toddler Development-Third Edition (Bayley-III), a comprehensive child assessment, and the Developmental Milestones Checklist (DMC), a parent-report of general development designed for the African context.

**Results:**

Among the 910 (453 HEU, 457 HU) infants, 670 (313 HEU, 357 HU) had at least one valid domain on the Bayley-III prior to 30 months of age (>90% completed all five domains), and 731 (342 HEU, 349 HU) children had a parent-completed DMC. Among HEU infants, 116 (37%) were exposed *in utero* to 3-drug antiretroviral combinations, and 196 (62.6%) to zidovudine; 355 (99%) of HU infants versus 27 (9%) of HEU infants ever breastfed. In adjusted analyses of Bayley-III scores, only mean cognitive scores differed significantly (slightly higher in HEU than HU, see Table 1). All Bayley-III outcomes were associated with at least one socioeconomic status (SES) factor, such as home electricity, food security, or toilet facilities. In adjusted models, there were no significant differences between HEU and HU in DMC scores across the four domains evaluated (Table 1).

**Conclusions:**

HEU children performed equally well to HIV unexposed children on two assessments of neurodevelopment at 24 months of age, easing the concern that HIV exposure and/or related neonatal ARV exposure negatively impact neurodevelopment.

**Abstract THPDB0101–Table 1:** Crude and Adjusted Differences in Mean Bayley-III Scores

	Mean (SD) scores		Unadjusted differences		Adjusted differences	
			
Bayley-III domain	HEU (N = 313)	HU (N = 357)	Mean difference	p	Mean difference	p
Cognitive	53.75 (3.37)	53.07 (3.25)	0.68	0.01	0.60	0.02
Gross motor	52.66 (2.77)	52.91 (2.67)	−0.25	0.25	−0.14	0.51
Fine motor	37.25 (1.80)	37.35 (1.80)	−0.10	0.46	−0.07	0.63
Expressive language	25.02 (4.36)	25.85 (3.98)	−0.83	0.01	−0.49	0.14
Receptive language	21.07 (3.54)	20.76 (3.21)	0.31	0.24	0.14	0.61

# Sub article: THPDB0102 Neurodevelopment of Ugandan and Malawian PROMISE HIV unexposed uninfected children

**Published:** 2016-07-22

**Authors:** M Boivin, A Sikorskii, J Aizire, L Maliwichi-Senganimalunje, L Wambuzi Ogwang, R Kawalazira, M Nyakato, I Familiar, H Ruisenor-Escudero, M Mallewa, T Taha, MG Fowler

**Affiliations:** 1 Psychiatry, Michigan State University, East Lansing, MI, United States; 2 Statistics and Probability, Michigan State University, East Lansing, MI, United States; 3 Infectious Disease, Johns Hopkins School of Public Health, Baltimore, MD, United States; 4 Global Mental Health, Johns Hopkins School of Public Health, Baltimore, MD, United States; 5 IMPAACT Studies, Makere University – Johns Hopkins University (MUJHU), Kampala, Uganda; 6 IMPAACT Studies, Malawi College Medicine – Johns Hopkins University, Blantyre, Malawi; 7 Family and Child Psychology, University of Chester, Chester, United Kingdom

## Abstract

**Introduction:**

HIV exposed uninfected (HEU) in Africa are developmentally at-risk both from the effects of HIV disease on the mother and foetus during gestation, and from pre-and postnatal (breast feeding) exposure to anti-retroviral treatments (ARTs). This NIH/NICHD-sponsored study compares neurodevelopmental outcomes of co-enrolled PROMISE Malawian and Ugandan children, to age- and gender-matched HIV uninfected unexposed (HUU) community controls.

**Methods:**

A total of 125 Malawian (Blantyre) and 125 Ugandan (Kampala) HEU infants completed neurodevelopmental assessment at both 12 and 24 months of age, along with 139 Malawian and 125 Ugandan age- and gender-matched HUU children. Children were tested with the Mullen Scales of Early Learning (MSEL). Data from 60% of total enrolled sample are presented here since 24-month MSEL testing is still going on. Overall loss to follow-up so far is 8%, mostly in the Malawian control cohort perhaps due to displacement from severe flooding in Blantyre January 2015. Least-squared means for standardized scores were compared by exposure group (HUU, HEU) and by country site (Uganda, Malawi) for 12 and 24 months using the linear mixed models with interaction effects of time, site and HIV exposure status.

**Results:**

In a repeated-measure (12 and 24 months) mixed models, HUU children had higher composite MSEL composite cognitive ability scores than the HEU cohort for the Malawi children at 12 months and for the Ugandan children at 24 months. This composite difference of ~1/2 SD (normative) was clinically meaningful in terms of developmental delay. Significant MSEL differences favouring HUU were obtained for Visual Reception, Fine Motor, and Expressive Language scales. Gross Motor and Receptive Language between-cohort differences were not significant. MSEL scores were significantly higher for the Malawian children differences for all scales (p<0.002) except for Fine Motor.

**Conclusions:**

HEU breastfed children on NVP prophylaxis or with maternal ART exposure are at greater overall neurodevelopmental risk than a matched cohort of HUU children, even though they receive monthly medical and nutritional monitoring and support through their enrolment in the IMPAACT PROMISE study. However, these preliminary findings suggest that most developmental domains are more at risk for the HEU children.

# Sub article: THPDB0103 Evaluation of the growth of young children born to HIV-infected mothers in western Kenya

**Published:** 2016-07-22

**Authors:** M McHenry, E Apondi, S Ayaya, W Tu, G Bi, E Sang, R Vreeman

**Affiliations:** 1 Children's Health Services Research, School of Medicine, Indiana University, Indianapolis, IN, United States; 2 Academic Model Providing Access to Healthcare (AMPATH), Eldoret, Kenya; 3 Department of Child Health and Paediatrics, School of Medicine, College of Health Sciences, Moi University, Eldoret, Kenya; 4 Biostatistics, School of Medicine, Indiana University, Indianapolis, IN, United States

## Abstract

**Introduction:**

Understanding growth patterns and associated factors for children born to HIV-infected mothers is critical for reducing morbidity and mortality. This study evaluated anthropometrics and factors associated with underweight status for children born to HIV-infected mother in western Kenya.

**Methods:**

Retrospective chart review was performed using data collected prospectively and stored in the electronic medical record system of Academic Model Providing Access to Healthcare (AMPATH). Data were obtained from children under the age of 5 years between January 2011 and September 2014. Descriptive statistics and logistic regression analysis were performed.

**Results:**

Data from 13,925 children born to HIV-infected mothers were included: 51.7% (n=7197) female, 2.67% (n=3731) double orphans, 69.2% (n=9639) HIV-exposed, uninfected (HEU), 14.75% (n=2054) HIV-infected, and 16.0% (n=2232) without confirmatory HIV testing during study period. Mean age at HIV diagnosis was 2.04±1.53 years. Mean weight-for-age Z score (WFAZ) was −0.68±1.45; 32.8% (n=4561) WFAZ −2 to −3 (moderately underweight) and 14.4% (n=2014) WFAZ <−3 (severely underweight) during the study period. Mean height-for-age Z score (HFAZ) was −1.38±1.92; 46.7% (n=6506) HFAZ −2 to −3 (moderately stunted) and 25.0% (n=3488) HFAZ <−3 (severely stunted). WFAZ changes over time differed between males/females and HIV/HEU (Figure 1). For those with a HIV-infected sibling, HIV-infected were more likely to have WFAZ<−2 (OR: 1.167; 95% CI: 1.042–1.307), while HEU were less likely (OR: 0.932; 95% CI: 0.970–0998). HEU were more likely to have WFAZ<−2 if they were orphaned (OR: 1.189; 95% CI: 1.001–1.413) and enrolled in clinic at a later age (OR: 3.212; 95% CI: 3.012–3.425), with each year of delayed enrolment increasing risk of WFAZ<−2 by 17% (OR: 1.167; p<0.001).

**Conclusions:**

Children born to HIV-infected mothers in western Kenya have greater degrees of underweight and stunting than the general Kenyan population. There appears to be a difference in the overall WFAZ trend over time between males and females, as well as between HIV-infected and HEU children.

**Figure 1:** Weight-for-age Z scores over time: comparisons.

# Sub article: THPDB0104 Drivers, barriers and consequences of HIV disclosure to HIV-infected children age 9–14 years: a qualitative study among children and their caregivers in Thika, Kenya

**Published:** 2016-07-22

**Authors:** N Njuguna, K Ngure, D Bukusi, D Wamalwa, N Mugo

**Affiliations:** 1 Partners in Health, Research and Development, Nairobi, Kenya; 2 Kenyatta National Hospital, Nairobi, Kenya; 3 University of Washington, Seattle, WA, United States; 4 Jomo Kenyatta University of Agriculture and Technology, Juja, Kenya; 5 University of Nairobi, Nairobi, Kenya; 6 Kenya Medical Research Institute, Nairobi, Kenya

## Abstract

**Introduction:**

HIV disclosure to adolescents is associated with increased adherence to antiretroviral drugs and reduced anxiety. However, disclosure rates remain low with recent studies estimating that only 3–37% of HIV-infected children under 15 years old in sub-Saharan Africa are aware of their HIV status. We sought to understand the motivation, process and impact of HIV disclosure to HIV-infected children in Thika, Kenya.

**Methods:**

We conducted semi-structured interviews between May and December 2014 through focus group discussions (FGDs) and in-depth interviews (IDIs) among HIV-infected children 9–14 years old enrolled in a human papilloma virus vaccine study and their caregivers (parents/guardians). IDIs were conducted among child-caregiver dyads while children’s FGDs were stratified by age and sex with caregivers interviewed separately. Transcripts were analyzed thematically to identify concepts related to HIV disclosure patterns and practices.

**Results:**

We conducted 20 IDIs with child-caregiver dyads (median age of children=12 years, adults=42 years) and 9 FGDs (3 with HIV-infected boys, 4 with HIV-infected girls and 2 with adult caregivers). Both children and caregivers reported disclosure as a one-time event with some HIV-infected caregivers choosing to disclose their own status at the same time. Disclosure was mainly as a consequence of persistent questioning by children concerning use of chronic medication or frequent hospital visits. Caregivers reported disclosing when they felt a child was “mature enough” to maintain confidentiality of HIV status. Caregivers typically described an older age group as ideal for disclosure when compared to children’s preference. Most children expressed emotional distress following the disclosure event. Many caregivers described support and encouragement by healthcare workers as a key enabler for disclosure. Stigma and feeling of inadequacy were identified as the main reasons for not disclosing their child’s status earlier. Negative community perceptions concerning HIV were identified as hindering disclosure by both children and their caregivers.

**Conclusions:**

Disclosure of HIV status continues to be a challenge for most caregivers resulting in abrupt disclosure with subsequent negative emotional outcomes for HIV-infected children. Structured disclosure interventional tools and techniques for parents and healthcare providers should be incorporated as part of healthcare management of HIV-infected children.

# Sub article: THPDB0105 Right heart abnormalities in HIV-infected children in Harare, Zimbabwe

**Published:** 2016-07-22

**Authors:** ED Majonga, J Rylance, JO Odland, K Kranzer, G Mchugh, J Metcalfe, T Bandason, H Mujuru, JP Kaski, RA Ferrand

**Affiliations:** 1 Clinical Research Department, London School of Hygiene & Tropical Medicine, London, United Kingdom; 2 Biomedical Research and Training Institute, Harare, Zimbabwe; 3 University of Liverpool, Liverpool, United Kingdom; 4 Liverpool School of Tropical Medicine, Liverpool, United Kingdom; 5 UiT, The Arctic University of Norway, Tromso, Norway; 6 UCSF, San Francisco, CA, United States; 7 Paediatrics, University of Zimbabwe, Harare, Zimbabwe; 8 Inherited Cardiovascular Diseases Unit, Great Ormond Street Hospital, London, United Kingdom; 9 Institute of Cardiovascular Science, University College London, London, United Kingdom

## Abstract

**Introduction:**

Cardiac abnormalities are a cause of chronic morbidity in HIV-infected children. Left ventricular (LV) hypertrophy and systolic and diastolic dysfunction are well described in HIV-infected children. In contrast, the right side of the heart is rarely studied. HIV-infected individuals are at an increased risk of pulmonary hypertension (PHT) and this may affect right ventricular (RV) function. This study aimed to determine the prevalence and risk factors of echocardiographically determined right heart abnormalities in HIV-infected children on ART.

**Methods:**

HIV-infected children aged 6–16 years on ART were enrolled from the Paediatric HIV clinic at Harare Central Hospital. Assessment included clinical history, New York Heart Association (NYHA) class, incremental shuttle walk testing, spirometry, viral load, CD4 count and transthoracic 2D, M-mode, pulsed wave and continuous wave Doppler echocardiography.

**Results:**

201 children, median age 11 (IQR: 9–12) and 48% females were enrolled. The median CD4 count was 726.5 (IQR: 473–935) and 68% had a viral load of <50 copies/ml. Chest pain on exertion was reported in 11% and chronic cough in 15%. Two children (1%) were hypoxic at rest and 11% post-exercise; 18% were symptomatic in NYHA score 2 and 3. Abnormal spirometry was found in 24%. The prevalence of right heart abnormalities was 30% (N=59). Right ventricular dilatation (RVD) was most common in 28% (N=56) and of these, 63% (N=37) had concomitant left heart abnormalities. There was biventricular dilatation in three patients and 45% (N=25) had RVD with LV systolic and/or diastolic dysfunction. PHT was found in 2% (N=3) and they had normal right ventricle size. Right heart abnormalities were not associated with any of the reported symptoms including chest pains, chronic cough or abnormal lung function.

**Conclusions:**

Our findings show a high prevalence of abnormalities of the right heart in HIV-infected older children and adolescents. Strikingly was the substantial proportion of patients with RV dilatation not associated with PHT. This could be secondary to left heart abnormalities or primary RV abnormality due to lack of association with lung function abnormality. Furthermore, inappropriate normative data may have over diagnosed RV dilatation.

# Sub article: THPDB0106 Treatment cascade of HIV-infected infants in the Thailand National Programme: how close are we to the 90-90-90 target?

**Published:** 2016-07-22

**Authors:** T Puthanakit, P Kosalaraksa, W Petdachai, R Hansudewechakul, T Borkird, R Lolekha, H Thaisri, T Samleerat, S Boonsuk, S Ongwandee, on behalf of the ACC Working Group

**Affiliations:** 1 Faculty of Medicine, Department of Pediatrics, Chulalongkorn University, Bangkok, Thailand; 2 HIV-NAT, Thai Red Cross AIDS Research Centre, Bangkok, Thailand; 3 Department of Pediatrics, Srinagarind Hospital, Khon Kaen, Thailand; 4 Department of Pediatrics, Phrachomklao Hospital, Petchaburi, Thailand; 5 Department of Pediatrics, Chiangrai Prachanukroh Hospital, Chiangrai, Thailand; 6 Department of Pediatrics, Hat Yai Hospital, Hat Yai, Thailand; 7 Global AIDS Program (GAP), Thailand Ministry of Public Health-U.S. CDC Collaboration (TUC), Nonthaburi, Thailand; 8 Department of Medical Sciences, Ministry of Public Health, Nonthaburi, Thailand; 9 Faculty of Associated Medical Sciences, Chiang Mai University, Chiang Mai, Thailand; 10 Department of Health, Ministry of Public Health, Nonthaburi, Thailand; 11 Bureau of AIDS, TB and STIs, Ministry of Public Health, Nonthaburi, Thailand

## Abstract

**Introduction:**

UNAIDS has set 90-90-90 targets for diagnosis, treatment and viral suppression in HIV-infected children by 2020. The Thailand Global AIDS Response Program estimated 4869 HIV-infected pregnant women and 102 new perinatal HIV-infected children in 2014. We describe the coverage of early infant diagnosis and treatment cascades of perinatally HIV-infected infants in the national program.

**Methods:**

The national AIDS program provides HIV DNA PCR testing for all HIV-exposed infants and antiretroviral therapy (ART) is provided, free of charge, regardless of CD4 count. Viral load testing is performed at 6 and 12 months after ART initiation. We analyzed national data collected on HIV-infected infants by the Active Case Management Network and the HIV DNA PCR database of 15 laboratories. The coverage of infant diagnosis will be calculated against estimated data from 2014.

**Results:**

From August 2014 to December 2015, 21,415 HIV DNA PCR tests were performed. Of these, 101 HIV-infected infants were identified, accounting for 70% of the estimated number of newly infected infants per year. ART was initiated in 83 infants (82%); 74 (89%) received the lopinavir/r-based regimen. The median age at ART initiation was 2.5 months (IQR: 1.2–4.2). In 55% of infants, ART was initiated the same day that blood was drawn for confirmatory HIV DNA PCR. The median (IQR) CD4 cell count was 2251 (1554–3057) cell/mm^3^ and the HIV-RNA prior to ART was 5.5 (3.6–6.4) log_10_ copies/ml. The overall mortality rate was 14% (9 infants died prior to and 5 infants died after ART initiation) and median age at death was 4.4 months (IQR: 2.4–6.2), with pneumonia being the commonest cause of death. Of these 15 deaths, 11 (73%) did not receive neonatal antiretroviral prophylaxis. The proportion of infants on ART with HIV RNA <400 copies/ml were (23/47) 49% (95% CI: 34–64) at 6 months and (11/18) 61% (95% CI: 36–83) at 12 months.

**Conclusions:**

Seventy percent of HIV-infected infants diagnosed, 82% began treatment, and 61% achieved virological suppression. A high mortality rate was noted, particularly among HIV-infected infants not included in the cascade care. Additional work is needed to prevent HIV-associated infant mortality and improve virological suppression among infants on ART.

# Sub article: THPDB0201 Point-of-care cryptococcal antigen screening – a case-control diagnostic accuracy study of the immuno-mycologics cryptococcal antigen lateral flow assay for screening finger-prick blood and urine among asymptomatic HIV-infected adults

**Published:** 2016-07-22

**Authors:** R Wake, J Jarvis, T Harrison, S Mashamaite, N Govender

**Affiliations:** 1 St George's University of London, London, United Kingdom; 2 National Institute for Communicable Diseases, Johannesburg, South Africa; 3 London School of Hygiene and Tropical Medicine, London, United Kingdom; 4 Botswana-UPenn Partnership, Gabarone, Botswana; 5 University of Pennsylvania, Philadelphia, United States; 6 Right to Care, Johannesburg, South Africa; 7 University of the Witwatersrand, Johannesburg, South Africa; 8 University of Cape Town, Cape Town, South Africa

## Abstract

**Introduction:**

Reflex laboratory screening of blood samples with CD4 counts of less than 100 cells/µl for cryptococcal antigen (CrAg) is being introduced nationally in South Africa. This enables identification of patients with sub-clinical cryptococcal infection and administration of pre-emptive fluconazole therapy to prevent life-threatening meningitis. However, access to laboratories may be limited in rural areas. The CrAg lateral flow assay (LFA) is ideally formatted for point-of-care (POC) use. Therefore, the accuracy of the CrAg LFA on finger-prick blood and urine samples performed in clinic settings by front-line health workers was determined.

**Methods:**

Patients with asymptomatic cryptococcal antigenaemia detected by reflex laboratory-based CrAg screening were identified from HIV clinics in Johannesburg, along with CrAg-negative controls. A CrAg LFA was performed on finger-prick blood and urine samples by a nurse and repeated in a laboratory. Results of POC and laboratory-performed LFA tests were compared to the reference laboratory-based CrAg LFA test performed on plasma during the previous month. Testing was repeated on positive urine samples following centrifugation at 2000 rpm for 10 minutes.

**Results:**

Fifty-three patients with known CrAg status (19 CrAg-positive: 34 CrAG-negative) were tested using POC and laboratory-based CrAg LFA. POC CrAg LFA on blood had a sensitivity of 89.5% (95% CI: 66.7–98.7%) and specificity of 100% (95% CI: 89.7–100%). Both CrAg positive patients who were tested as POC-LFA negative had very low CrAg titres. Sensitivity improved to 100% using laboratory-based testing. POC CrAg LFA on urine had a sensitivity of 84.2% (95% CI: 60.42–96.62%) and a specificity of 44.1% (95% CI: 27.2–62.1%), with no improvement using laboratory-based testing, or after centrifugation.

**Conclusions:**

CrAg LFA on finger-prick blood is an appropriate POC method for screening HIV-infected adults commencing ART. This could reduce turn-around time and loss to follow up, particularly where laboratory access is limited. Urine samples should not be used due to a high rate of false positive results.

# Sub article: THPDB0202 Utility of GeneXpert MTB/RIF assay in the diagnosis of extrapulmonary tuberculosis

**Published:** 2016-07-22

**Authors:** P Ramjathan, N Reddy, KP Mlisana

**Affiliations:** 1 National Health Laboratory Services, Microbiology, Durban, South Africa; 2 Department of Medical Microbiology, University of KwaZulu-Natal, Durban, South Africa

## Abstract

**Introduction:**

South Africa has high rates of both HIV and tuberculosis. HIV infection is linked with an increased risk of extrapulmonary tuberculosis (EPTB), and the risk increases as the CD4+ lymphocyte count declines. EPTB is difficult to diagnose due to its paucibacillary nature and culture takes up to 6 weeks. The GeneXpert MTB/RIF Assay (GXP) is a fully automated system developed by Cepheid that is easy to use with results available in 2 hours. There have been many studies on its use in pulmonary tuberculosis but few studies have focused on its utility with EPTB.

**Methods:**

The study was performed from June to October 2014 at King Edward VIII Hospital in Durban, Kwa-Zulu Natal. Fifty extrapulmonary specimens were processed at the National Health Laboratory Services facility. Specimens were incubated with sample reagent buffer and then GXP was performed according to manufacturer's instructions.

**Results:**

Specimens analyzed included 11 biopsy specimens, 15 pus specimens, 7 body fluids (1 pericardial fluid, 3 peritoneal fluids, and 3 ascitic fluids), 9 pleural fluids and 8 cerebrospinal fluids. Of the 50 extrapulmonary specimens processed, 12 (24%) were GXP positive and 8 (16%) were culture positive. Of a total of 8 culture positive specimens, 6 were GXP positive, and 2 were GXP negative giving a pooled sensitivity of 75% (using culture as the gold standard). Of a total of 42 culture negative specimens, 36 were GXP negative resulting in a specificity of 85.7%. There were 6 specimens that were GXP positive but culture negative. Ninety percent (45/50) of the extrapulmonary specimens were smear microscopy negative.

**Conclusions:**

The GXP assay provides rapid results that would aid in diagnosing EPTB earlier than culture. This study showed similar results to [1], who found that the sensitivity of the GXP was 77.3%. The 6 specimens that were GXP positive and culture negative could represent patients with tuberculosis that culture missed or they could be false positive results.

## Reference

1. Hillemann D., et al. 2011. Rapid Molecular Detection of Extrapulmonary Tuberculosis by the Automated GeneXpert MTB/RIF System. J Clin Microbiol. 49(4): 1202–1205.

# Sub article: THPDB0203 Stability of HIV serological markers collected by HemaSpot or dried blood spots

**Published:** 2016-07-22

**Authors:** M Manak, A Shutt, H Hack, L Jagodzinski, S Peel, B Danboise

**Affiliations:** 1 Henry Jackson Foundation, MHRP, Silver Spring, United States; 2 Waler Reed Army Institute for Research, MHRP, DLDM, Silver Spring, United States

## Abstract

**Introduction:**

Dried blood spot (DBS) technology is increasingly being used to acquire clinical specimens for biosurveillance, clinical trials, epidemiology, diagnosis and monitoring of vulnerable populations in remote areas. Problems with analyte recovery after prolonged storage at high temperature and humidity in addition to cumbersome processing procedures have hindered widespread acceptance of these methods. In this study, we compared the performance of the HemaSpot™ (Spot On Sciences) Blood Collection Device to DBS for collection and testing of HIV positive specimens.

**Methods:**

The HemaSpot™ Blood Collection Device and standard Whatman 903 DBS were evaluated on clinical specimens including HIV seroconversion panels known to be positive for HIV markers. Serum or plasma samples were reconstituted with an equal volume of whole blood from HIV negative donors; 100 µl was applied to the DBS or HemaSpot and placed into unsealed or sealed zip-lock bags containing desiccant. Specimens were stored at room temperature or in high humidity (95%) at 37 or 45°C for 30 days. Blood was eluted in 200 µl PBS-0.2% Tween, and tested by HIV-1/2/O, HIV Combo Ag/Ab EIA (EIA), HIV-1 Western Blot (WB), and MultiSpot HIV-1/HIV-2 Rapid Tests (MS), (Bio-Rad, Hercules, CA).

**Results:**

All HIV infected samples tested positive in the HIV-1/2/O, MultiSpot, and WB assays following up to 1 month storage at room temperature. A marked loss in reactivity was observed in samples stored at 37 or 45°C under high humidity conditions (Figure 1). Samples placed in a sealed bag with desiccant were protected from degradation. The HIV-1/2 Combo assay could not be used for screening with either device due to excessive background signal.

**Conclusions:**

Dried blood spot samples are suitable for collection, transport, and HIV testing by HIV 1/2/O Combo, MultiSpot, and WB, provided they are protected from humidity. The HemaSpot device was easier to use than standard DBS methods.

**Abstract THPDB0203–Figure 1:** Performance of HIV-1/2/O EIA on reconstituted seroconversion panel members (PRB947 and PRB910, SeraCare) eluted from HemaSpot (HS) or DBS.

# Sub article: THPDB0204 Evaluation of Abbott RealTi*m*e HIV-1 DBS assay

**Published:** 2016-07-22

**Authors:** N Tang, V Pahalawatta, A Frank, J Lampinen, G Leckie, R Bilkovski, Z Bagley, R Viana, CL Wallis, K Abravaya

**Affiliations:** 1 Abbott Molecular Inc., Des Plaines, IL, United States; 2 BARC-SA and Lancet Laboratories, Johannesburg, South Africa

## Abstract

**Introduction:**

HIV RNA suppression is a key indicator for monitoring of antiretroviral therapy. Viral load (VL) testing using dried blood spots (DBS) is a promising alternative to plasma based VL testing in resource-limited settings. The analytical and clinical performance of the Abbott RealTi*m*e HIV-1 assay using DBS from venous blood and finger prick blood was evaluated.

**Methods:**

Limit of detection (LOD) was determined using dilution of HIV-1 Virology Quality Assurance stock in venous blood. A total of 316 HIV-1 positive adult clinical samples collected from Ivory Coast, Uganda and South Africa were tested. For each participant sample, plasma, DBS-venous and DBS-finger were collected. Samples collected from Ivory Coast and Uganda were tested at Abbott Molecular and samples collected in South Africa were tested at Lancet Laboratories. For each HIV-1 participant, DBS-venous, DBS-finger and plasma sample results were compared. Correlation and mean bias values were obtained. The sensitivity and specificity were analyzed based on DBS misclassification at a threshold of 1000 HIV RNA copies/ml in plasma.

**Results:**

The LOD of the Abbott HIV VL assay on DBS was found to be 839 copies /ml. Participant samples included 195 (62.3%) “≥LOD”, 95 (30.35%) “Not detected”, 23 (7.35%) “<LOD detected” based on plasma results. Within the linear range of 839 copies/ml to 1×10^7^ copies/ml, the correlation coefficient for DBS-finger versus plasma (n=150) was 0.887, for DBS-venous versus plasma (n=150) was 0.902, for DBS-finger versus DBS-venous (n=146) was 0.947. The mean bias was −0.13 log copies for DBS-finger versus plasma, −0.10 log copies for DBS-venous versus plasma and −0.05 for DBS-finger versus DBS-venous. Using a misclassification threshold of 1000 copies/ml: for DBS-finger, 13/152 samples had an HIV VL ≥1000 with plasma and <1000 with DBS-finger giving a sensitivity of 91.4%; for DBS-venous, 9/152 samples had an HIV VL ≥1000 with plasma and <1000 with DBS-venous giving a sensitivity of 94.1%. The specificity was 96% (155/161) for both DBS-finger and DBS-venous (6/161 samples misclassified with a VL <1000 for plasma and ≥1000 for DBS).

**Conclusions:**

The HIV-1 VL quantitated between DBS-finger, DBS-venous and plasma correlates well. The mean bias between the sample types were all <0.2 log copies/ml.

# Sub article: THPDB0205 GeneXpert HIV-1 Quant: a tool for monitoring the success of ART programme in developing countries

**Published:** 2016-07-22

**Authors:** S Kulkarni, S Jadhav, P Khopkar, S Sane, V Chimanpure, V Dhilpe, R Londhe, M Ghate, R Yelgate, N Panchal, G Rahane, B Rewari, R Gangakhedkar

**Affiliations:** 1 Virology, National AIDS Research Institute, Pune, India; 2 WHO Country Officer for India, New Delhi, India; 3 National AIDS Research Institute, Pune, India

## Abstract

**Introduction:**

Recent guidelines re-identify virologic monitoring as the gold standard practice for diagnosing and confirming ART failure. In support to the national ART programme to scale up HIV-1 viral load testing with the point-of-care (POC) technologies, GeneXpert HIV-1 Quant assay was validated at NARI, Pune, India.

**Methods:**

A total of 314 HIV-positive individuals (pre ART n=151, on ART n=129, suspected ART failures n=34) were screened by the Abbott m2000rt RealTi*m*e HIV-1 VL assay. 219 plasma specimens falling in different viral load ranges (<40 to >5L copies/ml) were selected and tested by the GeneXpert HIV-1 Quant assay. Additionally, 20 seronegative, 16 stored specimens (1, 2, 3 months storage) and 10 spiked copy controls (40 to 40L copies/ml) were tested. Statistical analysis was carried out for determining the coefficient of variation (inter and intra assay), linear regression, Bland-Altman plots, sensitivity, specificity, NPV, PPV. The percent misclassifications were calculated for international and national cut-offs used for classifying the treatment failures (DHSs/AISs-200 copies/ml; WHO-400 copies/ml and NACO-1000 copies/ml).

**Results:**

Correlation between two assays (r=0.938) was statistically significant (p<0.01) and linear regression showed a good fit (R^2^=0.878). The GeneXpert HIV-1 Quant assay compared well with the gold standard with a higher sensitivity (91–95%), specificity (99–100%) and reproducibility on the spiked copy controls. The LLD and ULD of the GeneXpert HIV-1 Quant were 1.94 log_10_ and 6.98 log_10_ copies/ml. The misclassification rates for the three viral load cutoffs were not statistically different when compared by proportion test (p=0.830). Bland-Altman analysis showed almost all differences were within limits of agreement. All seronegative samples were negative and viral loads of the stored samples showed a good fit in the linear regression (R^2^=0.896 to 0.982).

**Conclusions:**

With the ease of performance and rapidity, the POC GeneXpert platform that is used for detection of *Mycobacterium tuberculosis* DNA can be used for HIV-1 quantification for better management of ART therapy. This will facilitate an integrated management of patients with HIV and TB and can be an important tool for scaling up the UNAIDS 90:90:90 initiative in resource limited settings.

# Sub article: THPDB0206 LYNX p24 antigen point-of-care test can improve infant HIV diagnosis in rural Zambia

**Published:** 2016-07-22

**Authors:** C Sutcliffe, P Thuma, K Sinywimaanzi, M Hamahuwa, W Moss

**Affiliations:** 1 Epidemiology, Johns Hopkins School of Public Health, Laurel, MD, United States; 2 Macha Research Trust, Choma, Zambia; 3 Epidemiology, Johns Hopkins Bloomberg School of Public Health, Baltimore, MD, United States

## Abstract

**Introduction:**

An affordable and effective point-of-care test would increase access to testing for HIV-1 exposed infants and linkage to care for HIV-infected infants. The LYNX p24 antigen point-of-care test, developed by CIGHT at Northwestern University and Northwestern Global Health Foundation, was shown to be valid in laboratory and clinical studies and has the potential to reduce the time to diagnosis and treatment in rural sub-Saharan Africa.

**Methods:**

From July 2014 to June 2015, LYNX testing machines were evaluated at the Macha Hospital HIV clinic in rural Southern Province, Zambia. HIV-exposed infants requiring early infant diagnosis were enrolled. At the study visit, a dried blood spot card was collected for HIV DNA testing in Lusaka and a blood sample was collected for the LYNX test. The LYNX test was performed immediately by clinic counsellors under observation by study staff. All steps in performing the LYNX test were assessed; HIV DNA test results were recorded.

**Results:**

A total of 156 infants (median age: 5.0 months; 48% male; 88% received PMTCT) were tested. Ninety-seven percent of tests were performed according to protocol. Among these, test results were available in a median of 55 minutes (IQR: 54, 58). Electricity was not available at the health care facility for 11% of tests (testing machines were operated using battery power). Eight infants tested positive and 137 tested negative by HIV DNA PCR (6 results yet to be returned). Of the 8 positive infants, 6 were also positive by the LYNX test (sensitivity: 75%; 95% CI: 35, 97). Of the 137 HIV DNA negative infants, 1 LYNX test was invalid and 136 were negative (specificity: 100%; 95% CI: 97, 100). The median time from sample collection to returning the HIV DNA PCR results to the mother was 90 days (IQR: 84, 141). Three mothers defaulted and 1 infant died while waiting for test results.

**Conclusions:**

The LYNX test was successfully performed by counsellors in rural health facilities and identified the majority of HIV-infected infants, without misdiagnosing any children. The LYNX test would significantly decrease the turnaround time for diagnosis in this setting, enabling HIV-infected infants to initiate antiretroviral therapy at an earlier age.

# Sub article: THPDC0101 Difficult decisions: individual and couple fertility desires and HIV acquisition among HIV serodiscordant couples in Zambia

**Published:** 2016-07-22

**Authors:** D Joseph Davey, K Wall, M Inambao, W Kilembe, I Brill, E Chomba, N Htee Khu, J Mulenga, A Tichacek, B Vwalika, S Allen, Rwanda-Zambia Hiv Research Group

**Affiliations:** 1 Epidemiology, University of California, Los Angeles (UCLA), Los Angeles, CA, United States; 2 Emory University, Atlanta, GA, United States; 3 Rwanda-Zambia HIV Research Group, Lusaka, Zambia; 4 University of Alabama, Birmingham, AL, United States

## Abstract

**Introduction:**

Previous research on the determinants of fertility desires in HIV-infected women, and serodiscordant couples has yielded mixed results. Despite the risk of HIV acquisition, transmission and mortality associated with conception, safer conception interventions for serodiscordant couples are not readily available in sub-Saharan Africa. This study evaluated determinants of fertility desires and effect of fertility desire on HIV acquisition among HIV serodiscordant couples in an open cohort in Lusaka, Zambia.

**Methods:**

We collected demographic, behavioural, clinical exposures, and data on fertility desires in a prospective cohort of HIV serodiscordant couples from 1995 to 2012. Factors associated with fertility desires by gender were evaluated using multivariable logistic regression. To estimate the overall effect of fertility desire on risk of HIV infection, we developed inverse-probability-of-treatment-weighted estimation of a marginal structural model to adjust for time-varying confounders affected by prior exposure.

**Results:**

Among 1029 serodiscordant couples, 311 agreed that they wanted a child in the future (30.4%), 368 agreed they did not want a child (36.0%), and 344 couples disagreed about having more children (33.6%). Women's fertility desire was associated with younger age (aOR=0.95; 95% CI=0.91–0.99), not being pregnant at baseline (aOR=0.21; 95% CI=0.12–0.37), fewer living children (aOR=0.75; 95% CI=0.62–0.90), fewer previous pregnancies (aOR=0.87; 95% CI=0.61–0.98), and partner wanting a child (aOR=2.79; 95% CI=1.97–3.95). Men's fertility desire was associated with younger age (aOR=0.88; 95% CI=0.80–0.97), fewer years cohabiting (aOR=0.95; 95% CI=0.90–1.00), fewer living children (aOR=0.84; 95% CI=0.70–1.01), and partner wanting a child (aOR=2.83; 95% CI=2.00–4.01). The adjusted risk ratio for woman's HIV acquisition was 2.06 (95% CI=1.40–3.03) among women wanting a child, 1.75 (95% CI=1.07–2.87) for men wanting a child, and highest at 2.55 (95% CI=1.32–4.93) when the couple agreed on wanting a child compared to couples who agreed they didn't want a child. Male seroconversion was not associated with fertility desire.

**Conclusions:**

Women had increased risk of seroconverting if they or their partner wanted a child. Safer conception interventions are needed to protect serodiscordant couples who want children.

# Sub article: THPDC0102 PrEP and ART reduce HIV transmission between members of HIV serodiscordant couples during pregnancy and pregnancy attempts

**Published:** 2016-07-22

**Authors:** R Heffron, N Mugo, K Ngure, E Bukusi, J Odoyo, E Katabira, N Bulya, S Asiimwe, E Tindimwebwa, J Haberer, M Marzinke, C Celum, JM Baeten

**Affiliations:** 1 University of Washington, Seattle, WA, United States; 2 Kenya Medical Research Institute, Nairobi, Kenya; 3 Makerere University, Kampala, Uganda; 4 Kabwohe Clinical Research Center, Kabwohe, Uganda; 5 Massachusetts General Hospital, Boston, MA, United States; 6 Johns Hopkins University, Baltimore, MD, United States

## Abstract

**Introduction:**

HIV-negative members of HIV serodiscordant couples are extremely vulnerable to HIV acquisition during pregnancy attempts because it is necessary to forgo condom use. Antiretroviral treatment (ART) and pre-exposure prophylaxis (PrEP) are powerful HIV prevention strategies and can be leveraged to reduce HIV transmission risk during pregnancy attempts.

**Methods:**

Among 1013 Kenyan and Ugandan high risk HIV serodiscordant couples, we implemented an integrated ART and PrEP strategy for HIV prevention and followed couples for 2 years. Following quarterly HIV testing, PrEP was provided to HIV-negative partners until HIV-positive partners used ART for ≥6 months. Contraception was available at all study sites. Pregnancy testing was conducted when clinically indicated and self-reported data on fertility desires and sexual behaviour were collected through standardized interviewer administered questionnaires on a quarterly basis.

**Results:**

In 67% of couples, the woman was the HIV-positive partner. At enrolment, 37% of HIV-positive and 39% of HIV-negative women reported intentions to have a child within 3 years and 77% of HIV-positive and 66% of HIV-negative women were not using effective contraception, proportions that remained fairly constant throughout follow up. Among HIV-positive and negative women, there were 154 incident pregnancies (incidence rate=18.6/100 person years) and 87 incident pregnancies (incidence=20.3/100 person years), respectively. There were no HIV transmissions to male partners during incident pregnancies or in the 6 months preceding those pregnancies. One woman seroconverted and became pregnant during the same 3-month interval; behavioural reports and biological testing confirm that she was not using PrEP prior to seroconversion. During the 3 months prior to pregnancy diagnosis, 62% of couples were using PrEP or ART; 30% were using both. Of 34 women who were using PrEP when they became pregnant, 88% elected to continue using PrEP during pregnancy.

**Conclusions:**

Fertility intentions were common and most couples chose to use PrEP and/or ART, which nearly eliminated HIV transmission during pregnancy and pre-conception periods in this large cohort with high pregnancy rates. ART and PrEP are important elements to promote within safer conception programmes for HIV serodiscordant couples.

# Sub article: THPDC0103 Undiagnosed HIV-infected partners are the major gap in the cascade for serodiscordant couples in two high prevalence settings

**Published:** 2016-07-22

**Authors:** D Maman, H Huerga, G Van Cutsem, I Mukui, B Chilima, W Hennequin, C Masiku, L Salumu, T Ellman, J-F Etard

**Affiliations:** 1 Epicentre – Médecins Sans Frontières (MSF), Cape Town, South Africa; 2 Epicentre – Médecins Sans Frontières (MSF), Paris, France; 3 Médecins Sans Frontières, Cape Town, South Africa; 4 National AIDS and STDs Control Program, Nairobi, Kenya; 5 Community Health Sciences Unit, Malawi Ministry of Health, Lilongwe, Malawi; 6 Médecins Sans Frontières, Nairobi, Kenya; 7 Médecins Sans Frontières, Lilongwe, Malawi; 8 Médecins Sans Frontières, Paris, France; 9 Southern Africa Medical Unit, Medecins Sans Frontières, Cape Town, South Africa

## Abstract

**Introduction:**

Discordant couples are a major source of HIV transmission. We quantified the prevalence of HIV discordant couples and, among HIV infected individuals, evaluated each step of the cascade of care in two high prevalence settings in sub-Saharan Africa.

**Methods:**

Two population-based surveys of persons aged 15–59 were conducted in Ndhiwa (Nyanza, Kenya) and Chiradzulu (Malawi) between September 2012 and May 2013, to assess HIV incidence and cascade of care. Each individual who agreed to participate was interviewed and tested for HIV at home. All HIV positive were tested for VL and CD4, regardless of their ART status. A couple consisted of two persons who were legally married or who were living together in a consensual union.

**Results:**

In total, 7425 houses were visited and among 15,104 individuals eligible, 13,345 (88.4%) were included and tested for HIV. Among 2970 identified as couples, HIV discordancy was found in 15.8% (95% CI: 13.9–17.9) in Kenya and 10.0% (95% CI: 7.9–12.7) in Malawi. Among couples with at least one HIV-infected partner, the proportion of HIV-discordancy was 45.8% in Kenya, 40.9% in Malawi. Men were the HIV-positive partner in 63.6% (95% CI: 56.7–70.0) of the discordant couples in Kenya, higher than in Malawi (47.9%; 95% CI: 40.4–55.5).

HIV status awareness among HIV-positive partner of discordant couples was 42.2% in Kenya and 64.4% in Malawi. VL suppression was 34.6% in Kenya and 54.5% in Malawi, lower than in the general population (40.0% in Kenya, 61.9% in Malawi). VL suppression was higher in women compared to men, in Kenya (39.5% vs. 26.8%, p=0.1) and in Malawi (61.2% vs. 46.5, p<0.01).

**Conclusions:**

Discordant couples were frequent and VL suppression ranged between 35 and 55% among HIV-positive partners. The high rate of unawareness of status among HIV-positive partners must be addressed in order to promote timely initiation of ART and/or PREP to reduce transmission within this high-risk group.

# Sub article: THPDC0104 Clinical outcomes and lessons learned from a safer conception clinic for HIV-affected couples trying to conceive

**Published:** 2016-07-22

**Authors:** S Schwartz, R Phofa, N Yende, J Bassett, I Sanne, A Van Rie

**Affiliations:** 1 Epidemiology, Johns Hopkins University, Baltimore, MD, United States; 2 Witkoppen Health and Welfare Centre, Johannesburg, South Africa; 3 Clinical HIV Research Unit, University of the Witwatersrand, Johannesburg, South Africa; 4 Epidemiology, University of North Carolina, Chapel Hill, NC, United States; 5 University of Antwerp, Antwerp, Belgium

## Abstract

**Introduction:**

Many couples affected by HIV express the desire to conceive despite the risk of horizontal and vertical HIV transmission. *Sakh'umndeni* is one of the first safer conception services integrated within a primary care clinic in sub-Saharan Africa.

**Methods:**

Since July 2013, Witkoppen Health and Welfare Centre in Johannesburg, South Africa, offers safer conception services to individuals in relationships who wish to conceive in the next 3 months if one or both partners are living with HIV. The safer conception clinic, *Sakh'umndeni*, offers patients a range of services and choices for HIV prevention including antiretroviral therapy (ART) initiation for HIV-positive partners independent of CD4 count, pre-exposure prophylaxis (PrEP) for HIV-negative partners, STI screening and treatment, viral load monitoring, counselling around peak fertility identification, self-insemination, and referral to on-site male medical circumcision. Participants are followed prospectively from enrolment until HIV testing at age 6 weeks of the infant.

**Results:**

Overall, 406 individuals participated in *Sakh'umndeni*, including 144 couples and 118 unaccompanied women. About half (46%) of all couples were serodiscordant (n=66/144). The majority (341/406, 84%) of participants were HIV positive. Most men (90%) and women (60%) already had one or more children. Median age was 34 years (IQR: 30–38) among women and 37 years (IQR: 34–43) among men. At enrolment, almost half (45%) of participants had engaged in condomless sex with their partner in the past 30 days. At first visit, 81% of HIV-positive women and 70% of HIV-positive men were already on ART. During follow-up, 51/52 HIV-positive ART-naïve individuals initiated ART. Only 14 of 66 (21%) HIV-negative participants chose PrEP: 13/30 HIV-negative women and 1/36 HIV-negative men. To-date, 57 pregnancies among 52 women (52/262=20%) have been documented, of which 9 (16%) resulted in a miscarriage. All 29 women failing to conceive with 6 months of attempted conception were referred to a fertility clinic with their partners. No horizontal transmissions have been observed; all 24 live-born babies ≥6 weeks tested HIV-negative.

**Conclusions:**

Results from over 2 years of *Sakh'umndeni* suggest that safer conception services can be effective at primary care in high burden settings, with low risks of horizontal and vertical HIV transmission.

# Sub article: THPDC0105 Uptake and clinical outcomes from a primary healthcare based safer conception service in Johannesburg, South Africa: findings at 7 months

**Published:** 2016-07-22

**Authors:** NECG Davies, S Mullick, S Schwartz

**Affiliations:** 1 Wits Reproductive Health and HIV Institute, University of the Witwatersrand, Johannesburg, South Africa; 2 Center for Public Health and Human Rights, Johns Hopkins School of Public Health, Baltimore, MD, United States

## Abstract

**Introduction:**

Safer-conception services (SCS) for HIV-seroconcordant, serodiscordant or sero-unknown couples support HIV counselling and testing (HCT), ART initiation, viral load (VL) suppression and HIV prevention. The South African Contraception and Fertility Planning Policy includes SCS provision yet primary healthcare (PHC)-based SCS remain rare. This study aimed to develop, implement and evaluate a PHC-based SCS, in Johannesburg, South Africa. Here, we report early uptake and clinical outcomes.

**Methods:**

Individuals and couples in HIV-seroconcordant, serodiscordant and sero-unknown relationships desiring pregnancy enrolled at the new SCS from existing PHC services. Safer-conception strategies offered included: HCT, ART initiation, VL monitoring, STI screening/management, and periovulatory condomless sex or self-insemination. Data were collected using standardized clinical record forms.

**Results:**

Of 218 participants enrolled, 141 (65%) were female (average 33.5 years; range 19–45) and 77 male (average 37.4 years, range 24–57). Overall, 134/141 (95%) women and 62/77 (81%) men were HIV-positive, including five newly diagnosed. No seroconversions have occurred amongst 22 HIV-negative clients.

Among SCS-users, 74 enrolled as couples (51 seroconcordant; 23 serodiscordant) and 70 as unaccompanied individuals (27 reporting seroconcordant relationships, 16 serodiscordant and 27 with unknown partner status). Among unaccompanied females, 7/18 disclosed to their partners post-enrolment, resulting in two more male HIV diagnoses and one ART initiation. Tailored safer-conception strategies were offered based on initial assessment. Twenty-one ARV-naïve clients initiated treatment. Out of 11 on ART for ≥3 months, 9 had VL <200 copies. Nine of 172 (5%) ART-experienced clients had VL >200 copies. All received adherence support. Four with confirmed virological failure switched to second-line therapy.

Fourteen pregnancies have been confirmed, two via self-insemination. Eight couples/individuals (57%) utilized optimal SC strategies, four attended only one consultation, two partners were not on ART. Thirteen (93%) of the women were virally suppressed with CD4 counts >350 cells at pregnancy confirmation. All attended antenatal care (ANC) ≤7 weeks’ gestation.

**Conclusions:**

Implementing a PHC-based safer-conception service is acceptable and feasible, impacting HCT, ART initiations, viral suppression and PMTCT targets including earlier ANC access. High demand for the service creates opportunities for HIV prevention and improved HIV treatment outcomes. However, challenges include partner non-disclosure, difficulties engaging men and pregnancies occurring before SC strategies are optimized.

# Sub article: THPDC0106 High planned partner pregnancy incidence among HIV-positive men in rural Uganda: implications for comprehensive safer conception services for men

**Published:** 2016-07-22

**Authors:** A Kaida, J Kabakyenga, M Bwana, F Bajunirwe, K Bennett, JE Haberer, Y Boum, JN Martin, P Hunt, DR Bangsberg, LT Matthews

**Affiliations:** 1 Faculty of Health Sciences, Simon Fraser University, Vancouver, BC, Canada; 2 Mbarara University of Science and Technology (MUST), Mbarara, Uganda; 3 Bennett Statistical Consulting, Ballston Lake, NY, United States; 4 Massachusetts General Hospital (MGH) Global Health and Department of General Medicine, Boston, MA, United States; 5 Epicentre Mbarara, Mbarara, Uganda; 6 Department of Medicine, University of California at San Francisco (UCSF), San Francisco, CA, United States; 7 Massachusetts General Hospital (MGH) Global Health and Division of Infectious Disease, Boston, MA, United States

## Abstract

**Introduction:**

In 2015, UNAIDS called for greater inclusion of men within sexual and reproductive health programming. An estimated 30 to 50% of HIV-positive men intend to have children, and approximately half have HIV-uninfected partners. Men often play a dominant role in reproductive decision-making, including uptake of strategies that support achievement of pregnancy while minimizing sexual HIV transmission. To inform implementation of male-inclusive safer conception services, we measured partner pregnancy incidence among HIV-positive men engaged in HIV care.

**Methods:**

Participants were enrolled in the Uganda AIDS Rural Treatment Outcomes cohort of HIV-positive individuals initiating antiretroviral therapy (ART) in Mbarara, Uganda. Bloodwork (CD4 cells/mm^3^, HIV-RNA) and questionnaires (including health status, behaviours, partner dynamics, and self-reported partner pregnancy) were completed at baseline and quarterly. Our analysis includes 189 HIV-positive men enrolled between 2011 and 2015. We measured partner pregnancy incidence overall and by reported partner HIV-serostatus and pregnancy intention.

**Results:**

At baseline, median age was 40 years (Q1, Q3: 35, 47) and median number of living children was 4 (Q1, Q3: 2, 5). Seventy-four percent were married with 66% reporting HIV-positive partners; 19% reported ≥2 sexual partners. Median years on ART was 3.9 (Q1, Q3: 0, 5.1), median CD4 was 318 cells/mm^3^ (Q1, Q3: 235, 424), and 51% were virally suppressed (<400 copies/ml). Over 480.7 person-years of follow-up, 63 men reported 85 partner pregnancies (incidence rate 17.7/100 person-years). By 3 years of follow-up, 33% reported at least one partner pregnancy (Figure 1). Of 85 pregnancies, 45% of partners were HIV-negative/unknown-serostatus and only 7% were unintended.

**Conclusions:**

One-third of HIV-positive men on ART reported at least one partner pregnancy within 3 years of follow-up. Nearly half of all pregnancy partners were at risk for HIV acquisition and over 90% were intended pregnancies. Findings highlight the need for reproductive health services that include men and support uptake of strategies to minimize sexual HIV transmission risk in the context of intended pregnancy.

**Figure 1:** Probability of reported partner pregnancy over time among HIV-positive men on ART in Mbarara, Uganda.

# Sub article: THPDD0101 Ethical and social implications of proposed HIV cure research: stakeholder perspectives from South Africa

**Published:** 2016-07-22

**Authors:** K Moodley, C Staunton, Z Duby, M Hendricks, T Roussow, M de Roubaix, G Nair, D Skinner

**Affiliations:** 1 Centre for Medical Ethics and Law, Stellenbosch University, Cape Town, South Africa; 2 Departments of Immunology and Family Medicine, University of Pretoria, Pretoria, South Africa; 3 CAPRISA, University of KwaZulu Natal, Durban, South Africa; 4 Community Health Division, Stellenbsoch University, Cape Town, South Africa

## Abstract

**Introduction:**

The ethical concerns associated with HIV prevention and treatment research have been widely explored in South Africa. However, HIV cure research is relatively new to the region and significant ethical and social challenges are anticipated as various scientific strategies including early treatment of acute infection, neutralizing antibodies, gene therapy and therapeutic vaccines are explored. Consequently, early stakeholder engagement is critical. While two studies in China and Australia have researched stakeholder perspectives, there has been no similar published empirical enquiry in Africa regarding HIV cure research. This study was conducted to gain preliminary data from South African HIV clinicians, patients, caregivers, medical students, researchers and activists.

**Methods:**

In-depth interviews were conducted on a purposive sample of thirty-five stakeholders in South Africa from October 2015 to February 2016. In addition five focus group discussions (FGDs) were conducted with adolescent patients, caregivers, adult patients, Community Advisory Board members and medical students. Audiotaped interviews and FGDs were transcribed verbatim with concurrent thematic analysis. Analyst triangulation occurred as the data were analyzed by three researchers independently and then integrated via discussion.

**Results:**

Common themes emerged from the interviews. The rapid evolution of HIV cure research agendas was prominent with participants expressing some concern that the global North was driving the cure agenda. Assessing and managing knowledge and expectations around HIV cure research emerged as a central theme related to challenges to constructing “cure”. Distinguishing between biomedical and emotional cure, remission and healing was highlighted as important. Avoiding curative misconception would be critical. Treatment interruption was regarded as a major risk if “cure” failed.

**Conclusions:**

A holistic approach integrating biomedical treatment, prevention and cure research is critical to achieve HIV eradication. The synergistic effect of such scientific research will be enhanced if the social and ethical dimensions of cure are taken into account. The findings of this study have important implications for community engagement, consent processes and possibly compensation for failed interventions in future cure trials. Undoubtedly, knowledge and resource sharing in the context of collaboration between research scientists working in cure and those working in treatment and prevention will accelerate progress towards cure.

# Sub article: THPDD0102 Community engagement in HIV cure-related research: applying good participatory practice (GPP) principles to community education efforts

**Published:** 2016-07-22

**Authors:** J Salzwedel, S Hannah, J Taylor, K Dube

**Affiliations:** 1 AVAC, New York, NY, United States; 2 Collaboratory of AIDS Researchers for Eradication (CARE) Community Advisory Board (CAB), Palm Springs, CA, United States; 3 Gillings School of Public Health, University of North Carolina at Chapel Hill, Chapel Hill, NC, United States

## Abstract

**Introduction:**

Meaningful community engagement demands basic scientific knowledge and principles of engagement. We investigated the attitudes and beliefs of people living with HIV toward HIV cure research. The Good Participatory Practice guidelines for HIV Biomedical Prevention (GPP) is a set of recommendations that outlines practices to engage a broad set of stakeholders in the research process.

**Methods:**

We completed a cross-sectional survey with 400 American adults living with HIV (22% females; 77% males; <1% transgender) connected to HIV cure research networks in 2015 to assess basic knowledge and attitudes around HIV cure research. The sample was ethnically diverse with broad geographical representation. We also conducted extensive key informant interviews with 36 people living with HIV, researchers, bioethicists and regulators to discuss the role of community in HIV cure research.

**Results:**

Of the survey respondents, 96% (95% CI: 91–100%; n=399) were generally interested in HIV cure research and 95% (95% CI: 90–100%; n=399) were concerned with medical issues. Nonetheless, a proportion of respondents (8% (3, 13%); n=350) thought a cure for HIV was already in existence. Study participants agreed that more information around HIV cure research was needed at a basic level of understanding given the complexity of the science, particularly about the various risks of HIV cure research modalities under investigation. Furthermore, the results revealed a disconnect between this stakeholder group and the research process, highlighting the need for a comprehensive and robust stakeholder engagement plan around HIV cure research in the United States.

**Conclusions:**

Given that a subset of HIV-positive survey respondents thought a cure for HIV existed but wasn't currently available to everyone, a comprehensive education and stakeholder engagement plan is needed. Engaging people living with HIV early in the research process using Good Participatory Principles can help lessen potential participants’ misconceptions and fears and lead to greater acceptance and support for HIV cure research. Additional formative research is needed with different stakeholder groups, including physicians, religious leaders and community educators.

# Sub article: THPDD0103 HIV cure research: a survey of Australian people living with HIV on perspectives, perceived benefits and willingness to participate in trials

**Published:** 2016-07-22

**Authors:** J Power, J Lucke, G Dowsett, G Brown, A Lyons

**Affiliations:** The Australian Research Centre in Sex, Health and Society, La Trobe University, Melbourne, Australia

## Abstract

**Introduction:**

In 2015, there were over 100 HIV Cure-related trials operating worldwide. Participation in current, and future, trials may pose health risks for people living with HIV (PLHIV), while being unlikely to deliver therapeutic benefit. As such, there is an ethical imperative for researchers to understand the motivations, expectations and understandings of potential trial participants. This paper reports on a survey of Australian PLHIV which aimed to identify:

- familiarity with HIV Cure research and optimism regarding achieving a cure;
- anticipated benefits of cure;
- socio-demographic and health-related characteristics of PLHIV who indicate willingness to participate in HIV Cure trials; and
- factors that moderate willingness to participate in a trial.

**Methods:**

Data for this study were derived from a cross-sectional survey of PLHIV in Australia conducted in 2015/2016. There were ~800 responses collected via a self-complete instrument that could be filled-in online or using a “pen and paper” survey. The study was advertised through HIV organizations, relevant email lists, social media and websites. Analysis involved (1) multivariate hierarchical regression to identify factors associated with greater willingness to participate in trials and (2) non-parametric tests to identify participants’ expectations regarding HIV cure.

**Results:**

Preliminary data analysis shows that not passing HIV to others and not being at risk of ill-health were the most desirable “HIV Cure” outcomes reported. Approximately 80% indicated willingness to participate in a trial. However, this was reduced if respondents thought participation could result in greater unpredictability of viral load, increased risk of resistance to current antiretroviral treatment, or increased susceptibility to illness. Belief that an HIV Cure would be achieved within the respondent's lifetime was associated with greater willingness to participate in a trial.

**Conclusions:**

These findings suggest that many Australian PLHIV are open to participating in HIV Cure trials. However, concern about possible effects on treatment efficacy and viral suppression clearly influences willingness, pointing to the importance of high quality informed consent processes. Optimism for achieving an HIV Cure may also influence willingness to participate. This is a further ethical concern as unrealistic optimism may be associated with misunderstanding of the therapeutic benefits of trial participation.

# Sub article: THPDD0104 Treatment interruptions in HIV cure studies in the United States: perceptions, motivations and ethical considerations from potential HIV-positive volunteers

**Published:** 2016-07-22

**Authors:** D Evans, J Taylor, L Sylla, S Garner, B Weiner, A Skinner, S Greene, S Rennie, K Dubé

**Affiliations:** 1 Project Inform, Research Advocacy, South Pasadena, CA, United States; 2 Collaboratory of AIDS Researchers for Eradication (CARE), Palm Springs, CA, United States; 3 defeatHIV Community Advisory Board, Seattle, WA, United States; 4 Division of AIDS (DAIDS), National Institute of Allergy and Infectious Diseases (NIAID), Bethesda, MD, United States; 5 UNC Gillings School of Global Public Health, Chapel Hill, NC, United States; 6 Center for Bioethics, Department of Social Medicine, University of North Carolina at Chapel Hill, Chapel Hill, NC, United States

## Abstract

**Introduction:**

We investigated perceptions of and willingness to undergo analytical treatment interruptions (ATIs) as part of HIV cure studies in the United States.

**Methods:**

We completed a cross-sectional survey with 400 American adults living with HIV (22% females; 77% males; <1% transgenders) in 2015. The sample was ethnically diverse and 38 U.S. states were represented. We also conducted extensive key informant interviews with 36 people living with HIV, researchers, bioethicists and regulators to assess motivations, perceptions of and concerns around ATIs.

**Results:**

In the sample of potential HIV-positive volunteers, 98% ((95% CI: 93–100%); n=400) were currently taking antiretrovirals. Almost half 44% (39–49%; n=399) reported they had ever participated in an HIV treatment study and 7% (2–12%; n=400) said they had ever been part of a HIV cure-related study. Of the survey respondents, 26% (21–31%) were very willing to interrupt treatment, 42% (37–47%) were somewhat willing, 12% (7–17%) were not very willing, 9% (4–14%) were not at all willing and 11% (6–16%; n=359) were unsure. Close to two thirds (65% (60–70%); n=350) reported that no more HIV treatment ever would be the definition of cure to them. Motivations for undergoing ATIs included: desire to help find a cure, past experiences with treatment interruptions and compensation. A subset of potential volunteers considered ATIs to be “too much risk” and expressed concerns about the possibility of viral rebound and development of resistance to ARVs. Clinicians-researchers and regulators were divided on the topic of ATIs. Some believed ATIs should not be attempted unless proof of concept is established for experimental modalities. Others agreed that there are criteria for proceeding with ATIs, including strong scientific justification, close monitoring and demonstrated substantial reduction in reservoir size and/or significant augmentation of the immune clearance function.

**Conclusions:**

As a functional cure may be defined as ART-free remission, ATIs could become a clinically meaningful measure for cure. To ensure ethical utilization, it will be important to continue understanding stakeholders’ perspectives while minimizing risks.

# Sub article: THPDD0105 Interrupting HIV treatment in cure research: scientific and ethical considerations

**Published:** 2016-07-22

**Authors:** S Garner, S Rennie, J Ananworanich, K Dube, D Margolis, J Sugarman, R Tressler, A Gilbertson, L Dawson

**Affiliations:** 1 Henry M. Jackson Foundation, Division of AIDS, NIAID, NIH, Bethesda, MD, United States; 2 University of North Carolina at Chapel Hill (UNC), Chapel Hill, NC, United States; 3 U.S. Military HIV Research Program, Bethesda, MD, United States; 4 Johns Hopkins University, Baltimore, MD, United States; 5 Henry M. Jackson Foundation, Bethesda, United States, MD; 6 Division of AIDS, NIAID, NIH, Bethesda, MD, United States

## Abstract

**Introduction:**

Intense activity is being directed at strategies for remission or eradication of HIV infection. However, current laboratory assays for HIV reservoir measurement are insufficient to demonstrate clearance of HIV and the best available test is to interrupt antiretroviral therapy (ART) – known as analytic treatment interruption (ATI) – for a defined period of time or until viral rebound occurs. Although ATIs are currently used in some HIV cure research, they raise important scientific and ethical questions.

**Methods:**

A multidisciplinary group conducted an ethical analysis of the use of ATI in HIV cure research. The analysis examined the rationale for ATI and its potential scientific utility as well as the risks and burdens to study participants. Criteria for use of ATI in HIV cure studies were developed.

**Results:**

Despite the ethical obligation to minimize research risks, there are limited data to directly inform risk assessment for ATIs in HIV cure trials. Experts have extrapolated from information from trials using longer repeated treatment interruptions, and from biological assays measuring effects of viral replication and inflammation. Despite these best efforts, it is difficult to ascertain the probability and magnitude of harm from a single ATI. There is also disagreement about the scientific utility of ATI in different types of studies. In spite of these uncertainties, studies involving ATIs must meet three basic ethical criteria:

1. a strong scientific justification for the ATI;
2. minimization of risks to study participants; and
3. a robust informed consent process.

**Conclusions:**

Based on the ethical criteria identified in this analysis, investigators should carefully consider the acceptability of ATIs by providing a strong justification of an ATI and carefully select participants. Further work should be undertaken by clinical researchers in partnership with social scientists, behavioural researchers and ethicists to enhance the ethical conduct of studies employing ATIs.

# Sub article: THPDD0106LB An innovation contest as community engagement for HIV cure research in North Carolina: a mixed methods evaluation

**Published:** 2016-07-22

**Authors:** A Mathews, A Anderson, S Farley, L Hightow-Weidman, K Muessig, S Rennie, J Tucker

**Affiliations:** 1 Institute for Global Health and Infectious Disease, University of North Carolina at Chapel Hill, Chapel Hill, NC, United States; 2 University of North Carolina at Greensboro, Greensboro, NC, United States; 3 Department of Global Health and Infectious Disease, University of North Carolina at Chapel Hill, Chapel Hill, NC, United States; 4 Social Medicine, University of North Carolina at Chapel Hill, Chapel Hill, NC, United States

## Abstract

**Introduction:**

Optimal ways to engage communities about HIV cure research remain unclear. The early stages of HIV cure research require an examination of alternative community engagement strategies that adopt a bottom-up approach. Innovation contests solicit contributions from the community, then evaluate and celebrate them. This pilot study evaluates community engagement in an innovation contest on HIV cure using social media analytics and qualitative methods.

**Methods:**

The innovation contest solicited images and videos of what HIV cure meant to people. Participants submitted entries to an Ideascale site, an encrypted online platform specifically designed for innovation contests. We primarily engaged Black young adults aged between 18 and 35 in North Carolina, because they are at the highest risk for acquiring HIV in the state. Recruitment included radio interviews and in-person and online engagement. Google, Twitter and Facebook analytics assessed social media contest engagement. Online engagement included page follows (unique users who subscribe to page update alerts), page visits, video views, reach (unique users who saw any contest-related material) and contest submissions. Qualitative research included focus groups and community forums that were transcribed, coded and analyzed using MaxQDA.

**Results:**

The innovation contest resulted in substantial in-person and online engagement, including in-person events (n=258 participants), focus groups and community forums (n=172 participants) and a reach of 168,364 unique users online. We have 31 contest submissions. Facebook analytics showed 277 page followers, 1297 page visits, 1409 video views and a combined 112,041 unique users who viewed any contest-related media. Similar trends were observed on Twitter, YouTube and Google analytics. Qualitative findings reveal the innovation contest was perceived as feasible and inclusive. In-person and online engagement about HIV cure research facilitated contest participation. Low HIV cure literacy and HIV-related stigma were barriers to contest participation.

**Conclusions:**

Innovation contests may be useful for HIV cure community engagement. Findings suggest that combining recruitment through in-person events and multimedia platforms reach a broad range of individuals for potential participation in innovation contests. Community contributions to innovation contests may provide useful content for culturally relevant and locally responsive social marketing campaigns.

# Sub article: THPDE0101 Project START intervention increases HIV testing uptake and decreases HIV risk behaviour among men released from prison: a randomized study in Ukraine

**Published:** 2016-07-22

**Authors:** T Kiriazova, Y Sereda, O Neduzhko, O Postnov, R Yorick, I Shvab, S Dvoriak

**Affiliations:** 1 Ukrainian Institute on Public Health Policy, Kyiv, Ukraine; 2 USAID RESPOND, Pact Inc, Kyiv, Ukraine

## Abstract

**Introduction:**

In Ukraine, the prevalence of infectious diseases and substance use disorders is high among incarcerated population. This study assessed whether the effective case-management intervention Project START decreases risk of HIV in men who transfer from prison to community in four regions of Ukraine.

**Methods:**

Male prisoners were randomized either to standard services or to an intervention group that included two sessions before release and up to four sessions within three months after release focused on decreasing risk of HIV, STIs and hepatitis transmission. Participants were assessed at baseline before the intervention, and at 3- and 6-month follow-ups. Primary outcome variables were: being at risk of contracting HIV due to their behaviour in the past 3 months, and having had an HIV test in the past 12 months. The effects for group, time and group by time interactions were analyzed using mixed effects logistic regression with random intercepts.

**Results:**

In total, 394 male prisoners (mean age 35.2±9.4 years, mean duration of imprisonment 41.0±24.7 months, 56.9% reported history of injection drug use) were included in the study. Follow-up rates at 3- and 6-month assessments were 86% and 85% for the intervention group and 82% and 82% for the control group, correspondingly. Compared to controls, participants in the intervention group were significantly less likely to report HIV-related risk behaviour, including irregular condom use or/and sharing injection needles in the past three months (AOR 0.51, 95% CI: 0.39–0.67, identical for the 3- and 6-month post-release assessments). Intervention was associated with higher odds of testing for HIV at 3-month (AOR 4.56, 95% CI: 1.61–12.94) and 6-month (AOR 1.70, 95% CI: 1.08–8.88) follow-ups.

**Conclusions:**

In this first implementation study of the Project START in Ukraine, the intervention was shown effective in increasing HIV testing uptake and decreasing HIV risk behaviour in men released from prison, and can be implemented in other countries of the Eastern Europe and Central Asia.

# Sub article: THPDE0102 Expanding HIV and STI care to prisoners: the experience from Zomba Central Prison, Malawi

**Published:** 2016-07-22

**Authors:** DB Garone, G Mateyu, J van Oosterhout, V Singano, V van Schoor, M Chigayo, H Ndindi, A Kwekwesa, S Gaven, K Harawa

**Affiliations:** 1 Medical, Dignitas International, Zomba, Malawi; 2 Medical, Malawi Prison Health Services, Zomba, Malawi

## Abstract

**Introduction:**

Globally, prevalence rates of HIV, sexually transmitted infections (STI's) and hepatitis B (HBV) in prison populations are 2 to 50 times higher than in general populations. Risks affect prisoners, prison staff, their families and the entire community. In 2013, the Malawi Prison Services established a steering committee to scale up HIV care and treatment in Malawian Prisons. We used routinely collected programme data to evaluate the HIV cascade and prevalence of syphilis and HBV in Zomba Central Prison, a high-security facility in the south of Malawi.

**Description:**

Since 2014 Dignitas International and the Malawi Prison Health Services have been implementing a comprehensive package of care and treatment for HIV-TB and STI's. Prisoners are routinely screened for HIV, TB and STI at entry and when they are released. In addition, HIV, TB, syphilis and HBV screening campaigns are conducted every six months.

**Lessons learned:**

During a June 2015 screening campaign 1052/1745 (60%) prisoners with unknown HIV status accepted to be tested for HIV, HBV and syphilis. 68/1052 (6.5%) tested HIV positive, resulting in an overall prison HIV prevalence of 35%. 52/1052 (4.9%) tested positive for syphilis and 59/1052 (5.7%) tested positive for HBV. 1.1% were co-infected with HIV/HBV. By October 2015, 482/539 (89%) HIV positive patients were on ART, 52% were initiated due WHO 3/4, 48% due to low CD4 (<350 or <500 cells/µl, depending on calendar episode). 98% were on the standardized 1st line ART regimen. All ART patients who were eligible for routine viral load (VL) monitoring according to National Guidelines received VL testing. 277/319 (86%) were virologically suppressed (<1000 copies/ml). All patients with VL≥1,000 accessed enhanced adherence counselling.

**Conclusions/Next steps:**

Malawian Prisoners attained acceptable HIV testing coverage, high ART uptake, and good adherence demonstrated by high virological suppression. Incarceration provides an opportunity to address HIV care in hard-to-reach individuals. Prison health care programmes needs to carefully plan for the special needs of prisoners such as confidentiality and continuity of care within and outside prisons.

# Sub article: THPDE0103 Institutionalizing health education in prisons: the adoption of peer education as the national approach for HIV prevention among inmates in Mozambique

**Published:** 2016-07-22

**Authors:** A Zandamela, MR Mobaracaly, M Benedetti, D Solomon, V Bernardo

**Affiliations:** 1 Pathfinder International, Maputo City, Mozambique; 2 Pathfinder International, Watertown, NY, United States; 3 Consultant, Pathfinder International, Watertown, NY, United States

## Abstract

**Introduction:**

Incarcerated populations in Mozambique are particularly vulnerable to HIV infection. A recent study found the HIV prevalence among male prisoners to be 24%, nearly three times higher than the general adult male population (9.2%). Transmission and acquisition of HIV is affected by many factors, with a large proportion of incarcerated men reporting consensual sex, commercial sex and coerced sex involving both inmates and staff. Overcrowding, poor sanitation, lack of medical assistance, malnutrition and violence also increase prisoners’ vulnerability to HIV exposure, making them a priority group for HIV prevention and care interventions. Despite this, health facilities are often scarce within the prison system.

**Description:**

To respond to the health needs of the prison population, Pathfinder International, in partnership with Ministry of Justice (MOJ) and supported by CDC/PEPFAR, developed a multi-faceted HIV prevention programme implemented in 10 prisons across Mozambique that incorporated behavioural, biomedical and structural interventions. In order to generate demand and stimulate behaviour change, a 48-hour training curriculum on sexual and reproductive health and rights was used to train 100 peer educators. These peer educators conducted one-on-one sessions with other inmates, and also led groups of 5–8 people in a series of five sessions that provided information regarding HIV, STIs and TB. Additionally 26 senior prison staff were trained to facilitate linkages between prison and health facilities to improve access to health services.

**Lessons learned:**

Between April and December 2015, 3988 inmates took part in the peer education programme, resulting in 2265 referrals to health services and 991 people receiving HTC (HIV testing and counselling). 87 people (8.8%) were found to be HIV positive and 58 (66.7%) were enrolled into care and treatment. The use of a peer-led model allowed for the integration of health services into the existing prison model. This promotes both scalability and sustainability. In addition, although the project focused on HIV prevention and treatment, the training curriculum also provided information on STIs, TB, nutrition, hygiene and sanitation, allowing for adaption of this model to address a range health needs.

**Conclusions/Next steps:**

Peer support mechanisms are an effective method of generating demand and reinforcing healthy behaviour among incarcerated populations.

# Sub article: THPDE0104 Female prisoners in Zambia: resourcing and relational risk factors for health and healthcare access

**Published:** 2016-07-22

**Authors:** SM Topp, CN Moonga, C Mudenda, C Chileshe, G Magwende, SJ Heymann, G Henostroza

**Affiliations:** 1 College of Public Health, Medical and Veterinary Sciences, James Cook University, Belgian Gardens, Australia; 2 Centre for Infectious Disease Research, Lusaka, Zambia; 3 Zambian Prison Service, Kabwe, Zambia; 4 School of Public Health, University of California Los Angeles, Los Angeles, CA, United States; 5 School of Medicine, University of Alabama at Birmingham, Birmingham, AL, United States; 6 Centre for Infectious Disease Research in Zambia, Lusaka, Zambia

## Abstract

**Introduction:**

In sub-Saharan Africa, empirical research focussing on the experiences and issues of women prisoners is almost non-existent. Forming part of a larger programme to strengthen Zambian prison health systems this study examined factors driving health and access to healthcare among Zambia's female inmates.

**Methods:**

A total of 44 interviews were conducted with a cluster random sample of 23 women (50% HIV-positive) prisoners and 21 officers and healthcare workers. Four Zambian prisons were purposively selected based on geographic spread (one facility in each of four provinces), and a range of security levels (two medium-, one maximum- and one low-security District facility). Interviews were translated and transcribed and analyzed using an inductive approach that drew on the principles of health systems analysis.

**Results:**

Poor environmental conditions - including massive overcrowding and lack of adequate sanitation - and poor quality or insufficient quantity of food affected all female prisoners’ health. Access to health services was shaped by a combination of prison resourcing, administrative bias and inmate-officer relationships. For example, basic service availability was weak due to the absence of internal health services in any of the female prisons. However, in some sites, access was further limited when male prisoners (in adjacent holding facilities) were given priority access to already limited transport for travel to external health centres. A further compounding factor was the varied and *ad hoc* female officers “responsiveness to requests for healthcare access, with sympathetic responses often dependent on inmates” wealth or long-term relationship with the officer. Women prisoners with no visible physical symptoms of ill-health, as well as those looking after children, reported particular difficulties in persuading officers to commit resources to helping them access services.

**Conclusions:**

This study highlights the compounding effect that resource deficits (e.g. weak infrastructure, lack of health services and poor nutrition) and organizational culture (e.g. female officers’ lack of responsiveness to women's health needs and implicit prioritization of male inmates’ service access) are having on the health status and healthcare access of Zambian female prisoners. Findings suggest that vulnerabilities and inequities experienced by Zambian women in society are being exacerbated and deepened in prison.

# Sub article: THPDE0105 Promoting human rights and access to health services in prisons in Southern Africa: VSO, UNODC and SDC working together to reduce HIV and improve the health of incarcerated populations

**Published:** 2016-07-22

**Authors:** C Ingleby, M Ayalew, T Ponde

**Affiliations:** 1 Programme Development and Policy Team, Voluntary Service Overseas, London, United Kingdom; 2 Voluntary Service Overseas Rhaisa, Pretoria, South Africa

## Abstract

**Introduction:**

Across Southern Africa HIV infection rates in the regions overcrowded prisons are double that of the general population. Health service provision is poor and rates of disease such as TB, STIs and hepatitis are unacceptably high. Female prisoners, adolescent and juvenile males are at particular risk of sexual coercion and violence.

Governments are reluctant to invest money in prisoner health or rehabilitation despite the fact that up to 33% of prisoners are on remand and most prisoners are eventually released back into society.

**Description:**

The project works across 7 countries (Malawi, Zimbabwe, Swaziland, Zambia, Mozambique, Lesotho and Tanzania) following a holistic programming approach including:

- Base line research to establish conditions in relation to prisoner health across the region
- Building capacity of civil society organizations by placing VSO volunteers with organizational development and social work skills to improve health services delivered in prison settings
- Regional advocacy by the VSO supported Southern African Network on Prisons and working with and training Southern African parliamentarians to lobby for implementation of regional minimum prison health standards
- Engaging with ex-offenders and raising their voice at national level Prisons Technical Working Groups.

**Lessons learned:**

Evaluation conducted to date has highlighted the following:

- Working with prisoner populations requires time and commitment. A long process of sensitization was undertaken with ministries of justice, parliamentarians, donors and ex-offenders to build support.
- The importance of an evidence base. VSO conducted research and small scale prison projects over a number of years prior to this project.
- The joint approach of capacity building of civil society organizations and prison settings whilst undertaking high level advocacy is most effective
- Working regionally, across 7 countries, maximizes opportunities to share learning
- Long term funding support from SDC provides essential project stability.

**Conclusions/Next steps:**

- Use learning from the first 3 focus countries (Malawi, Swaziland and Zimbabwe) to expand project activity across the remaining four.
- Increase focus on youth and juvenile prisoners as the most vulnerable sub-group scaling up the provision of psycho-social support
- Promote prison health at global level in line with SDG commitments to leave no-one behind.

# Sub article: THPDE0201 Modelling the cost-per-HIV infection averted by couples’ voluntary HIV counselling and testing in six African countries

**Published:** 2016-07-22

**Authors:** K Wall, M Inamba, W Kilembe, E Karita, B Vwalika, J Mulenga, R Parker, T Sharkey, A Tichacek, E Hunter, R Yohnka, J Abdallah, S Allen

**Affiliations:** 1 Epidemiology, Emory University, Atlanta, GA, United States; 2 Emory University Rwanda Zambia HIV Research Group, Ndola, Zambia; 3 Emory University Rwanda Zambia HIV Research Group, Lusaka, Zambia; 4 Emory University Rwanda Zambia HIV Research Group, Kigali, Rwanda; 5 Emory University Rwanda Zambia HIV Research Group, Atlanta, GA, United States

## Abstract

**Introduction:**

Though couples’ voluntary HIV counselling and testing (CVCT) has been shown to be effective in multiple countries and financially cost-effective in Zambia, it is not yet widely implemented, funded, or systematically measured in sub-Saharan Africa.

**Methods:**

We recently demonstrated the cost effectiveness of CVCT in Zambia using the actual financial expenditures and observed HIV prevention impact of implementing CVCT in Lusaka, Copperbelt, and Southern Provinces among 172,981 couples. Here, we estimate the cost-per-HIV infection averted (CHIA) by CVCT in six sub-Saharan African countries with very different HIV epidemics. We used the prevention impact of CVCT observed in Zambia (63–84% reduction in HIV incidence) as well as conservative estimates of effect (50% reduction). Based on experience implementing CVCT in two countries, models assume a 4-phase CVCT implementation: in the initiation phase, we assume 10% of couples are tested at $75/couple; in the expansion phase an additional 10% of couples tested at $50/couple; in the mature phase an additional 60% of couples tested at $25/couple; and in the maintenance phase 20% of residual and new couples tested at $10/couple.

**Results:**

CVCT CHIA ranged from extremes of $35 (in Lesotho, assuming 10-years of impact and observed estimates of CVCT effectiveness) to $3076 (in Sierra Leone, assuming 5-years of impact and conservative estimates of CVCT effectiveness) (Figure 1). Our model is most sensitive to HIV prevalence and couple serodiscordance. CHIAs were lowest in areas with high prevalence of HIV and HIV discordance (as in Southern Africa) and highest in areas with lower prevalence of HIV and HIV discordance (as in Western Africa).

**Conclusions:**

Estimates of CVCT CHIA were cost-effective under a range of real-world implementation scenarios. These findings provide further support for inclusion of CVCT as a required indicator and for funding priority setting.

**Abstract THPDE0201–Figure 1:** Cost-per-HIV infection averted by CVCT in countries with a range of HIV.

# Sub article: THPDE0202 HIV prevention costs and its determinants: evidence from the ORPHEA project in Kenya

**Published:** 2016-07-22

**Authors:** O Galarraga, RG Wamai, SG Sosa-Rubi, M Mugo, D Contreras, S Bautista-Arredondo, H Nyakundi, J Wang'ombe

**Affiliations:** 1 Health Services, Policy & Practice, Brown University, Providence, RI, United States; 2 Global Health Initiative, Northeastern University, Boston, MA, United States; 3 National Institute of Public Health (INSP), Cuernavaca, Morelos, Mexico; 4 School of Economics, University of Nairobi, Nairobi, Kenya; 5 School of Public Health, University of Nairobi, Nairobi, Kenya

## Abstract

**Introduction:**

As part of the “Optimizing the Response of Prevention: HIV Efficiency in Africa” (ORPHEA) project, we analyzed determinants of economic efficiency for two HIV prevention interventions in Kenya: HIV testing & counselling (HTC) and prevention of mother-to-child transmission (PMTCT).

**Methods:**

We collected retrospective data from key informants, administrative registers and time-motion observations for 2011–12 from 78 multi-stage sampled health facilities in 33 districts across Kenya. We stratified analyses by health facility type, ownership, size and intervention type. We computed total costs of production using both quantities and unit prices for each input. We estimated average costs by dividing total cost per intervention by number of clients accessing the intervention. We used forward-selection stepwise regression methods to identify and analyze significant determinants of log-transformed average costs (p<0.05).

**Results:**

For HTC, the cost per client tested was $7.4 and cost per client tested and positive was $146. For PMTCT, cost per client tested was $57.4 and cost per client tested and positive was $677 (Table 1). We found evidence of economies of scale for the two interventions: doubling the number of clients per year was associated with average cost reductions of 25% for HTC and 48% for PMTCT (Figure 1). Task shifting was associated with reduced costs for PMTCT (59–63%), but not for HTC. On the other hand, costs in facilities that target testing (for persons most at risk or for those with symptoms) tend to have higher costs for HTC (63–75%) but not for PMTCT.

**Conclusions:**

Aside from increasing production scale, HIV prevention costs may be further contained by using task shifting for PMTCT; targeted testing for HTC may require more resources.

**Abstract THPDE0202–Figure 1:** Economies of scale in HIV prevention services: Kenyan health facilities, 2011–12.

**Abstract THPDE0202–Table 1:** Average cost (US$) per client across HIV prevention service cascade in Kenyan health facilities, 2011–12

		N (facilities)	Mean	Patient-volume weighted mean	Median	Standard deviation	IQR
**HTC:**	Cost per client tested	56	7.4	6.5	4.8	7.1	5.9
	Cost per client tested and positive	56	145.9	80.2	54.9	318.0	74.7
**PMTCT:**	Cost per client tested	57	57.4	47.3	32.7	84.8	60.5
	Cost per client tested and positive	49	677.2	753.9	254.2	1,056.8	571.6
	Cost per client on ART	14	2,472.6	1,752.6	364.6	5,464.7	1,689.9

# Sub article: THPDE0203 Answering the financial question with country programmes: what is the cost and impact of adopting the 2015 WHO paediatric HIV treatment guidelines?

**Published:** 2016-07-22

**Authors:** A Wilhelm, C Amole, C Middlecote, J Harwell, E Mccarthy

**Affiliations:** 1 HIV Access Team, Clinton Health Access Initiative, London, United Kingdom; 2 HIV Access Team, Clinton Health Access Initiative, Boston, MA, United States; 3 Clinical Support Team, Clinton Health Access Initiative, Boston, MA, United States; 4 Applied Analytics Team, Clinton Health Access Initiative, Lusaka, Zambia

## Abstract

**Introduction:**

The 2015 revisions to the WHO paediatric HIV treatment guidelines align with the UNAIDS 90-90-90 strategy by recommending countries treat all HIV+ patients <15 years. In moving from the 2013 guidelines, which recommended treating all <5 years and treating those >5 years if CD4<500, Governments want to understand the costs in adopting both this treat-all strategy and latest optimized Inter-Agency Task Team (IATT) products, such as heat-stable LPV/r oral pellets to replace cold-chain LPV/r syrup to improve treatment outcomes for new initiates<3 years.

**Methods:**

We conducted a macro-level, government-perspective, 5-year (2016–2020) cost analysis of a hypothetical country (defined as 150,000 HIV+ children <15 years). We included the following variable costs: antiretroviral (ARV) drugs, CD4 tests, viral load tests, early infant diagnosis tests and health worker salaries, with fixed and programmatic costs excluded. Three scenarios – comparing WHO 2013 versus 2015 eligibility guidelines, and also a LPV/r pellet and ABC/3TC 120/60 mg regimen choice for ART initiates – were then analyzed and compared in an Excel-based mathematical model.

**Results:**

A per-patient-per-year costing using WHO 2013 versus 2015 guidelines yielded estimated ARV costs of US$119 versus $US122 in 2016, increasing to US$152 versus $US156 in 2020 to reach the “90% coverage by 2020” UNAIDS target. Total ARV+Lab+HR programme costs for 2013 versus 2015 guidelines were estimated at US$11.5M versus US$13.6M in 2016, with an annual average of US$22M versus US$26M through 2020. Notably, adopting LPV/r oral pellets only added $US4M to total 5-year costs for 2015 guideline adoption.

**Conclusions:**

This evidence indicates a 23% increase in overall costs to adopt a treat-all paediatric strategy featuring LPV/r pellets. This should encourage countries to develop a budgetary roadmap to expand HIV treatment to all infected children. The model and process can be applied in new contexts for informed HIV treatment scale-up policy and implementation decisions.

**Figure 1:** Comparing Annual Pediatric Costs and Patient Volumes: WHO 2013 vs. 2015 Guidelines.

# Sub article: THPDE0204 Average costs of voluntary medical male circumcision and their determinants in Kenya, Rwanda, South Africa and Zambia

**Published:** 2016-07-22

**Authors:** SG Sosa-Rubí, S Bautista-Arredondo, M Opuni, D Contreras-Loya, G La Hera-Fuentes, A Salas-Ortiz, A Kwan, J Condo, K Dzekedzeke, O Galárraga, N Martinson, F Masiye, S Nsanzimana, R Wamai, J Wang'ombe

**Affiliations:** 1 Health Economics, Instituto Nacional de Salud Pública, Cuernavaca, Mexico; 2 UNAIDS, Geneva, Switzerland; 3 World Bank, San Francisco, CA, United States; 4 School of Public Health, National University of Rwanda, Kigali, Rwanda; 5 Dzekedzeke Research & Consultancy, Lusaka, Zambia; 6 Brown University, Providence, RI, United States; 7 Perinatal HIV Research Unit, University of the Witwatersrand, Johannesburg, South Africa; 8 Division of Economics, University of Zambia, Lusaka, Zambia; 9 Rwanda Biomedical Center, Kigali, Rwanda; 10 Northeastern University, Boston, United States; 11 School of Public Health, University of Nairobi, Nairobi, Kenya

## Abstract

**Introduction:**

Voluntary medical male circumcision (VMMC) is recommended by WHO as a key component of HIV prevention. VMMC programmes have substantially contributed to avert new infections, but while coverage scales up, critical challenges arise such as technology adoption and availability of resources hindering the achievement of their goals. The objective of this study was to estimate average costs and to analyze the determinants of efficiency of VMMC in Kenya, Rwanda, South Africa and Zambia for 2013, as part of the ORPHEA project.

**Methods:**

ORPHEA is a cross-sectional observational study with data collected in Kenya, Rwanda, South Africa and Zambia between 2011 and 2013. The analytical sample comprise of 82 facilities: 25 facilities in Kenya, 20 facilities in Rwanda, 23 facilities in South Africa and 14 facilities in Zambia. Micro-costing methods were applied to determine relevant costs (personnel, supplies, utilities, equipment and property) and output data. Information on self-reported time allocation was used to estimate staff costs and national prices of surgical kits and HIV tests were used in the calculations.

**Results:**

Average cost per VMMC was US$ 29 in Rwanda, US$ 51 in Zambia, US$ 32 in Kenya and US$ 106 in South Africa. Considerable variation in the average cost within countries was found; staff costs dominated in South Africa and Zambia (55 and 59%, respectively), and circumcision kits costs in Kenya and Rwanda (46%). In all countries, except for Rwanda, we found that unit cost per VMMC decreased with the number of MC clients: from 31 to 48% of average reduction by doubling the number of clients. In general, there were also variations by type of facility, with lower average cost per MC clients found in health centres (both public and private) as compared to hospitals.

**Conclusions:**

There were important differences in the average cost per VMMC across facilities in the four countries studied that provide opportunities for increasing the levels of efficiency in the provision of VMMC. Additionally, it is important to expand the volume of VMMC services by means of sustained demand generation, while acceptable levels of quality are maintained.

# Sub article: THPDE0205 Spending more to spend less: the unit costs of a tailored demand creation intervention to increase uptake of voluntary medical male circumcision

**Published:** 2016-07-22

**Authors:** S Torres-Rueda, HA Weiss, M Wambura, H Mahler, J Chilongani, E Kuringe, R Hayes, M Plotkin, M Makokha, A Hellar, C Schutte, G Mshana, N Larke, G Lija, J Changalucha, JM Grund, F Terris-Prestholt

**Affiliations:** 1 London School of Hygiene & Tropical Medicine, London, United Kingdom; 2 National Institute for Medical Research (NIMR), Mwanza, United Republic of Tanzania; 3 Jhpiego Tanzania, Dar es Salaam, United Republic of Tanzania; 4 Strategic Development Consultants, Durban, South Africa; 5 National AIDS Control Program, Ministry of Health and Social Welfare, Dar es Salaam, United Republic of Tanzania; 6 Division of Global HIV/AIDS, Centers for Disease Control and Prevention, Atlanta, GA, United States

## Abstract

**Introduction:**

HIV prevalence is higher for men aged 20–34 years than for younger males (aged 15–19 years) in Tanzania. Voluntary medical male circumcision (VMMC) is a proven HIV prevention intervention, but uptake in Tanzania is highest among younger males. A cluster randomized controlled trial was conducted to assess the effectiveness of a locally-adapted demand creation intervention in increasing uptake of VMMC among men aged 20–34 years in Tabora and Njombe regions of Tanzania. The intervention evaluated included: demand-creation messages; use of peer promoters; separate waiting areas for older clients and information sessions for female partners. This study presents the total, incremental and unit costs of this VMMC intervention.

**Methods:**

Cost data were collected from a provider's perspective on surgical, demand-creation and supervisory activities in all clusters across both trial arms. Costs per circumcision were calculated taking into account staff, supplies, start-up and capital costs. Univariate sensitivity analyses were conducted to understand drivers of unit costs.

**Results:**

The total mean costs per cluster were higher in the intervention arms ($48,820 and $46,222, in Tabora and Njombe, respectively) than the control arms ($36,088 and $37,344). Cluster-level client load varied widely across clusters and was higher in the intervention arms (480 to 1187 in Tabora, and 218 to 500 in Njombe) than in the control arms (272 to 951, and 102 to 268, respectively). Demand increased more than proportionately, resulting in lower unit costs: the costs per male circumcised in the intervention arms were $62 ($42–$99) in Tabora and $139 ($93–$195) in Njombe, while in the control arms they were $72 ($39–$123) and $202 ($132–$313), respectively. The sensitivity analysis showed that client volume was a greater determinant of unit costs than input prices or other variables.

**Conclusions:**

The higher unit cost of VMMC in Njombe compared to Tabora may be due to greater VMMC saturation: the number of clients in Tabora was 2.5 times higher than in Njombe. Despite added costs of delivering the intervention, mean unit costs per circumcision were lower. Developing a tailored demand creation package for older VMMC clients is likely an effective approach to increase uptake and ultimately reduce unit costs.

# Sub article: THPDE0206 Rapidly falling costs for new hepatitis C direct-acting antivirals (DAAs): potential for universal access

**Published:** 2016-07-22

**Authors:** D Gotham, M Barber, J Fortunak, A Pozniak, A Hill

**Affiliations:** 1 Faculty of Medicine, Imperial College London, London, United Kingdom; 2 Centre of Development Studies, Cambridge University, Cambridge, United Kingdom; 3 Department of Chemistry and Pharmaceutical Sciences, Howard University, Washington, DC, United States; 4 Chelsea and Westminster Hospital, St Stephens AIDS Trust, London, United Kingdom

## Abstract

**Introduction:**

Novel direct-acting antivirals (DAAs) achieve high sustained viral response rates of >90% in chronic hepatitis C (HCV). But access to DAAs is low: “access” prices are available in countries covering only 50% of the worldwide epidemic. Production costs depend largely on prices of active pharmaceutical ingredient (API).

**Methods:**

Data on the per-kilogram prices of exported API, and export volumes, were extracted from an online database (www.infodriveindia.com) for Jan–Dec 2015. Mean end-2015 API costs were calculated using linear regression models weighted by shipment size (figures). For velpatasvir, API cost was calculated by analyzing chemical synthesis, costs of raw materials, processes and yields. Per-pill API costs were calculated based on daily dosage. Estimated costs for formulation and excipients ($0.04/ pill), packaging ($0.35/month) and a profit margin (50%) were added.

**Results:**

Total exports from India in 2015 were, sofosbuvir: 8.4 tons, (equivalent to 250,000 12-week treatment courses), daclatasvir: 523 kg (104,000 courses), ledipasvir: 56 kg (7300 courses). API prices decreased throughout 2015 (Figure 1). End-2015 API prices were sofosbuvir $1758/kg, daclatasvir $1432/kg, ledipasvir $11,432/kg. API cost for velpatasvir was estimated at $8900–11,700/kg. US price was 884 times higher than the target price for sofosbuvir, 3706 times for daclatasvir and 409 times higher for sofosbuvir+ledipasvir.

**Conclusions:**

HCV DAAs production costs are falling rapidly. We estimate that 12-week treatments of sofosbuvir can be manufactured for $95, sofosbuvir+ledipasvir $231, daclatasvir $17, velpatasvir $119–154, all including a 50% profit margin. These low production prices show the potential for Universal Access programmes for HCV, similar to those already established for HIV/AIDS.

**Abstract THPDE0206–Figure 1:** Decreasing prices of exported sofosbuvir/daclatasvir through 2015. 1 bubble = 1 shipment, bubble area scaled to size of shipment in kg.

**Abstract THPDE0206–Table 1:** Calculated target prices and current prices for 12-week DAA treatment courses

Drug	End-2015 API cost/kg	Target price per 12-week treatment	Current global lowest price per 12-week treatment	Current US price per 12-week treatment
Sofosbuvir (SOF)	$1,758	$95	$483	$84,000
Daclatasvir	$1,432	$17	$183	$63,000
Ledipasvir (LDV)	$11,432	$136	unknown	unknown
SOF + LDV	N/A	$231	$615	$94,500
Velpatasvir	$8,900–11,700	$119–154	unknown	unknown

# Sub article: AUTHOR INDEX

**Published:** 2016-07-22

**Table:** 

**A**	
Abach, J	FRAB0102LB
Abdallah, J	THPDE0201
Abdallah, S	WEAC0403, WEPDD0103^*^
Abdool Karim, Q	FRAE0102, THAX0104, TUAC0201, TUPDA0101, TUPDA0105, WEAA0102, WEAB0101
Abdool Karim, S	THAX0104, TUPDA0101, WEAA0102, WEAB0101
Abdool Karim, SS	FRAE0102, TUPDA0105
Abena Messomo Mbida, P	THAB0104
Aberle-Grasse, J	WEAE0201
Abimiku, A	WEAB0103
Abishev, A	TUPDB0103
Abongomera, G	TUAB0204^*^
Aboud, M	THAB0203^*^, THAB0205LB
Abramovitz, D	WEAC0405
Abrams, E	WEPDB0103
Abrams, EJ	TUAB0101, WEAB0105, WEPDE0106LB
Abravaya, K	THPDB0204
Abreu, L	TUPDD0206
Achra, AC	WEPDB0101
Adera, F	WEPDC0107^*^
Adetokunboh, O	TUPDE0102
Adeyemi, O	WEAB0301, WEPDE0104
Adipo, T	WEPDC0107
Affolabi, D	WEAB0205LB
Agegnehu, D	THAE0302
Agius, P	TUPDD0301
Agolory, S	TUPDC0104
Agot, K	FRAC0104^*^, WEPDC0104
Agovi, AM-A	TUPDC0104
Aguiar, P	TUPDD0201
Aguilar-Martinez, JM	THAC0103
Agutu, C	FRAB0101LB
Ahmed, M	THAX0105
Ahmed, R	THAA0206
Ahmed, S	THAX0102, WEAB0204, WEAE0204
Aizire, J	THPDB0102
Ajibola, G	THPDB0101
Ajok, S	WEAD0202
Albert-Hope, C	WEAD0304^*^
Alejos, B	WEPDB0105^*^
Alexander, G	WEAE0205
Alicea, C	THAA0201
Allen, H	WEAD0203^*^
Allen, M	TUAX0102LB
Allen, S	THPDC0101, THPDE0201, WEAD0101
Alloui, C	TUPDB0104
Altice, F	WEAC0402
Altice, FL	WEAC0404
Amara, RR	THAA0206^*^
Ambani, A	THAD0101
Ambrose, K	THAE0203
Amico, R	TUAC0102
Amin, J	FRAE0105
Amin, T	FRAD0204
Amole, C	THPDE0203
Amoros, I	TUPDB0104
Amoros Quiles, I	FRAE0201
Anahtar, MN	TUPDA0104
Ananworanich, J	THPDD0105, TUAX0101LB^*^
Ancuta, P	THPDA0102
Andama, A	WEAB0202
Anderegg, N	FRAE0202
Anderson, A	THPDD0106LB, TUPDD0303
Anderson, E	THAA0104LB
Anderson, J	WEAE0306LB
Anderson, JL	THPDA0104
Anderson, S	TUAD0201
Andersson, M	WEPDB0102
Andrade, A	TUAC0102
Andrew, E	FRAE0101
Andrew, P	THAD0106LB, TUAC0102
Andrews, L	FRAC0103
Angel, J	THAB0206LB
Angelidou, K	THAB0106LB
Anglaret, X	WEAB0303
Anoma, C	THAC0102
Ansari, A	THPDA0101
Apetrei, C	THAA0205, THPDA0103, TUAA0102, TUAC0101, WEAA0103
Apollon, A	WEAE0202
Apondi, E	THPDB0103
Aptekar, S	TUPDB0101
Ardiet, DL	TUPDB0104
Arellano, G	THAB0101
Arenas-Pinto, A	THAB0202^*^
Arhel, N	WEPDA0104
Armstrong, D	WEAB0202
Arthos, J	THPDA0101^*^
Artz, L	FRAD0102
Arunmanakul, A	WEPDE0205
Asari, V	WEAB0101
Asege, L	WEAB0202
Asiimwe, S	THPDC0102, WEAC0105, WEAE0304
Asokan, M	WEPDA0101
Aspin, C	WEPDD0106
Athiambo, M	WEPDC0102
Atuhumuza, E	WEAB0202
Atujuna, M	TUAD0104
Audet, C	WEPDD0102^*^
Auerbach, J	FRAE0104
Auld, A	TUAC0204
Aung, PP	TUPDD0301
Aurpibul, L	THAB0103LB, THAB0106LB
Avalos, A	THAE0302
Avihingsanon, A	FRAB0103LB, THAB0104, WEPDB0101
Awori, QD	WEPDC0105^*^
Awotwi, E	FRAD0104
Ayala, V	THAA0101
Ayalew, M	THPDE0105
Ayaya, S	THPDB0103, FRAE0203, FRAE0205, WEAC0106LB
Aylott, A	THAB0205LB
Azar, M	WEAC0402
Azbel, L	WEAC0404
Azizuyo, B	WEPDE0202^*^
**B**	
Babiker, A	WEAA0105LB
Babikhina, K	FRAD0205^*^
Back, D	TUAC0103
Badjé, A	WEAB0303
Baeten, J	FRAE0106LB^*^, TUAC0105LB, WEAC0105^*^, WEAE0304, WEPDC0203
Baeten, JM	THPDC0102
Baggaley, R	TUAC0104
Baghazal, AA	WEAC0403^*^
Bagley, Z	THPDB0204
Bah-sow, O	WEAB0205LB
Baijnath, P	TUAA0103
Bailey, RC	WEPDC0102, WEPDC0107
Bajos, N	TUAD0103
Bajracharya, A	TUPDD0301
Bajunirwe, F	THPDC0106
Bakowska, E	WEPDB0101
Bakwalufu, J	WEPDE0105
Balestre, E	FRAC0105LB
Balkan, S	TUPDB0104
Balzer, L	WEAC0106LB
Bamba, A	TUAD0202
Banda, G	TUAE0104
Banda, KM	TUPDC0104
Bandason, T	THPDB0105
Bangsberg, DR	THPDC0106
Bañón, S	TUPDB0106
Baral, S	THAE0105, TUAD0202, TUAD0302, TUPDD0106, WEAD0306LB
Barasa, M	WEPDC0105, THPDE0206
Barbosa de Souza, M	TUPDC0105
Barbour, R	WEAC0402
Barde, A	WEPDE0104
Bardeguez, A	WEAB0105
Barihuta, T	THAE0103
Barker, C	THAE0104^*^
Barnabas, R	WEAE0304^*^
Barnes, P	THAB0104
Barnighausen, T	FRAC0105LB
Baron, D	WEPDC0206
Barone, MA	WEPDC0105
Barr, D	WEAB0203
Barrenas, C	WEAA0103
Barrington, C	TUAD0401
Barron, P	WEPDE0103^*^
Baruwa, E	THAE0203
Basar, M	THAB0106LB
Bassett, I	THAE0204*
Bassett, J	THPDC0104, TUAB0203
Bastos, F	FRAD0206
Batrouney, C	FRAC0102
Bauer, GR	WEAC0205
Baum, M	TUPDA0106
Bautista-Arredondo, S	THPDE0202, THPDE0204^*^, WEAE0105
Bavinton, BR	FRAC0102, THAC0101^*^
Baxter, C	FRAE0102
Baya, J	WEPDD0103
Bazin, B	FRAC0105LB
Bärnighausen, T	THAB0102, WEAE0204
Beauchamp, G	THAC0105LB, WEAC0104
Beck, G	WEAA0106LB
Beckham, S	WEPDE0203
Bedi, K	WEPDA0103
Bekker, L-G	TUAX0102LB^*^
Beletsky, L	WEAC0405
Belkind, U	WEAC0202
Bell, J	THAA0104LB
Belloso, W	WEPDB0101
Belonosova, E	THAB0205LB
Belzer, M	TUPDD0203
Benedetti, M	THPDE0103
Benfield, T	THAB0201
Bennett, K	THPDC0106, TUPDC0103, WEAE0305
Benson, S	WEPDB0103
Bere, A	TUAC0201
Berkhout, B	TUAA0104
Bernard, EJ	FRAD0101^*^
Bernardo, V	THPDE0103
Bernaud, C	WEAC0102
Bertrand, S	WEAC0403
Bhagani, SR	WEAB0304LB
Bhardwaj, K	FRAD0202^*^
Bhardwaj, S	TUAE0105, TUAE0106
Bhayani, L	WEAD0301
Bi, G	THPDB0103
Biello, K	TUPDD0203, WEAC0203
Bii, S	WEAE0203
Bilkovski, R	THPDB0204
Binda, K	WEAB0104
Binley, J	WEPDA0101
Bisht, M	FRAC0101
Bisignano, A	TUAD0403
Bivol, S	THAD0105^*^
Black, D	WEAC0106LB
Blantari, J	FRAD0104
Blick, G	TUPDC0106
Blumenthal, S	THAE0105
Bock, N	THAE0303
Boivin, M	THPDB0102^*^
Boleo, C	TUPDC0103, WEAE0305
Bolton-Moore, C	TUAB0104
Bond, V	WEPDD0104
Bonnecwe, C	WEPDC0106
Bonnington, O	TUAD0405, WEPDD0101
Bonzela, J	TUAC0204
Boonsuk, S	THPDB0106
Bor, J	THAB0102^*^, TUAB0102, TUAB0205, TUAC0205^*^, TUPDC0102, WEAE0204^*^
Borkird, T	THPDB0106
Borquez, A	WEAC0405^*^
Bose, M	TUPDA0102
Bosomprah, S	WEAE0101
Bouchaud, O	TUPDB0104
Boulle, A	WEAB0203
Boum, Y	THPDC0106
Bouzas, MB	TUPDB0102
Bowman, BA	TUPDA0104
Boyd, A	WEAB0303
Boyd, R	THAE0302
Boyer, S	FRAC0105LB, THAD0101
Boyes, ME	THAD0204
Bozinoff, N	TUAD0102^*^
Bozzi, G	THAA0104LB^*^
Bradley, J	FRAC0102
Brahmbhatt, H	THAD0203
Brainard, DM	WEAB0301
Brand, RM	TUAC0103
Bräu, N	WEAB0301^*^
Bredeek, UF	THAB0203
Brenchley, J	TUAA0101
Brennan, A	TUAB0205, TUAC0205, WEAB0102
Brennan, C	THAB0203
Brennan, DJ	TUPDD0204
Brewster-Lee, D	WEAD0103
Brezak, A	THAX0102^*^
Brigham, F	WEAC0202
Brigstock-Barron, O	WEAE0306LB^*^
Brill, I	THPDC0101, WEAD0101
Brittain, K	WEPDE0106LB
Britto, P	THAB0103LB
Brocca-Cofano, E	THAA0205, TUAA0102, TUAC0101^*^, WEAA0103
Brooks, R	FRAC0103
Brooks-Pollock, E	WEAC0404
Brothers, J	TUAX0104LB
Brotto, LA	TUPDD0306
Brouwers, P	THAB0202
Brown, E	TUAC0105LB^*^
Brown, G	THPDD0103
Brown, H	WEAA0105LB
Brown, L	TUAD0104
Browning, R	THAB0103LB, THAB0106LB
Brummel, S	THAB0103LB, THAB0106LB
Bryant, H	THAE0206
Bryant, K	WEAC0401
Bucek, A	TUAB0101, WEPDB0103
Buchanan, A	THAB0205LB
Buehler, S	TUAD0205
Bukenya, D	TUAD0405, WEPDD0101, WEPDE0102
Bukowski, L	TUAD0205^*^, WEAC0204^*^
Bukusi, D	THPDB0104
Bukusi, E	FRAE0205, THPDC0102, WEAC0105, WEAC0106LB1
Bukusi, EA	FRAE0203
Bulterys, M	WEAB0305LB
Bulya, N	THPDC0102, WEAC0105
Bunu, A	TUPDE0105^*^
Burchett, S	WEAB0105
Burgener, A	TUAA0106LB
Burke, S	WEAE0206LB
Burman, W	TUPDC0105
Burmeister, S	WEPDB0102
Burton, R	WEAB0203
Burwitz, B	TUAA0101
Busch, MP	THAX0105, WEAA0106LB
Busza, J	TUAX0103LB
Bwakura-Dangarembizi, M	FRAB0101LB, FRAB0102LB
Bwana, M	THPDC0106
Bygrave, H	WEAE0301
Byrareddy, S	THPDA0101
Byrd, J	WEAE0205
Byrne, EH	TUPDA0104
**C**	
Cadigan, J	TUAD0301
Cahn, P	FRAB0103LB^*^, FRAB0104LB^*^
Cai, F	THAX0105
Cai, Y	THAA0201
Cale, E	WEPDA0101^*^
Cambiano, V	TUAX0103LB
Cameron, DW	WEAB0103
Cameron, PU	THPDA0104
Campa, A	TUPDA0106
Campbell, JR	THAE0301^*^
Campbell, T	TUPDA0103
Candrinho, B	TUAB0202
Capitant, C	THAE0304, WEAC0102
Caraulan, L	THAD0105
Cardenas-Ochoa, A	TUAA0105
Cardoso, J	TUAC0204
Carias, AM	WEAA0102
Carlson, J	TUPDA0101
Carmona, S	TUAB0102, TUAB0205, TUAC0205, TUPDC0102
Carneiro, P	WEAC0202
Carnimeo, V	TUPDB0104
Carolus, G	WEPDD0104
Carrasco, MA	TUAD0401^*^
Carrington, M	WEPDA0102
Carroll, RW	TUPDC0106
Carter, A	TUPDD0306^*^
Casado, JL	TUPDB0106
Casavant, I	TUAC0204
Cassell, M	WEPDE0205
Castellanos, E	WEAD0304
Castelnuovo, B	THAB0105
Cathcart, R	WEPDE0201
Catranji, V	THAD0105
Cattamanchi, A	WEAB0202
Cawley, C	FRAE0201, THAD0104
Cawood, C	THAX0104, TUAC0201, TUPDC0101
Cazein, F	TUAC0203
Cecchini, DM	TUPDB0102^*^
Cecilio, ME	WEPDE0204
Celum, C	FRAE0106LB, THPDC0102, WEAC0105, WEAE0304
Ceranto, A	TUPDD0204
Chabala, C	TUAB0204
Chabata, S	TUAX0103LB
Chabeda, S	TUAD0303
Chakhtoura, N	THAB0103LB, THAB0106LB, WEAB0105
Chamanga, R	THAB0106LB
Chamie, G	WEAC0106LB
Chanaiwa, V	THAB0106LB
Chandawale, A	THAB0106LB
Chandra, C	THAE0105
Chandra, S	WEAB0201
Chang, E	TUPDE0104
Chang, M	WEAE0304
Chang, W	FRAE0203
Changalucha, J	THPDE0205
Chapman, S	TUAE0101^*^
Chappell, E	TUAB0103
Charlebois, E	FRAE0205, TUAD0105, WEAC0106LB
Charlebois, ED	FRAE0203
Charoenying, S	WEPDE0205
Charreau, I	WEAC0102, THAE0304
Chas, J	WEAC0102, THAE0304
Chasanov, WM	THAB0202
Chaturvedi, S	TUPDC0104
Chaudhury, S	THPDB0101
Chege, W	TUAC0102
Chelbi-Alix, M	WEPDA0104
Chemhuru, M	TUAX0103LB
Chen, C-H	THAA0106LB
Chen, L	WEAB0305LB
Chen, M	FRAC0102
Chen, S	WEPDA0105^*^
Chen, Y	TUAC0102, THAX0105, WEAC0104
Chen, Z	WEPDA0105
Cheng, H	WEPDC0203
Cheng, Z	TUAD0305
Cheu, R	TUAA0106LB
Chidiac, C	THAE0304
Chigayo, M	THPDE0102
Chikandiwa, A	THAD0203, WEAD0204
Chikonda, J	TUAE0104
Chileshe, C	THPDE0104
Chilima, B	THPDC0103, TUPDB0104
Chilongani, J	THPDE0205
Chimanpure, V	THPDB0205
Chimbetete, C	THAD0102, TUAB0104
Chime, C	WEPDE0104
Chinkonde, J	TUAE0104
Chinsinga, B	TUPDD0103
Chipadze, MR	TUPDC0104
Chipeta, Z	TUAC0201
Chipungu, J	WEAE0101
Chirchir, B	WEPDC0105
Chirowodza, A	TUPDE0102^*^
Chirwa, E	TUAD0203, TUPDD0304
Chirwa, Z	TUPDB0104
Chiyaka, T	TUAX0103LB
Chomba, E	THPDC0101, WEAD0101
Chomchey, N	TUAX0101LB
Chomont, N	THPDA0102, TUAX0101LB
Chopera, D	TUPDA0101^*^
Chung, AW	THAA0203
Church, K	THAD0104, WEPDE0102
Cianci, GC	WEAA0102
Cicala, C	THPDA0101
Clark, T	FRAE0205, WEAC0106LB
Clark, TD	FRAE0203
Clarke, A	WEPDB0101
Clarke, K	WEAE0106LB
Clotet, B	THAB0206LB
Cluver, L	TUAB0201^*^
Cluver, LD	THAD0204
Coffey-Esquivel, J	TUPDD0203
Cofield, SS	WEAC0305LB
Cohen, C	FRAE0205, WEAC0106LB
Cohen, CR	FRAE0203
Cohen, KE	TUPDA0104
Cohen, MS	THAX0105
Cole, M	WEPDA0102
Colebunders, R	TUAB0204
Coletti, A	THAB0103LB, THAB0106LB
Collins, IJ	TUAB0105LB^*^
Collins, S	THAB0202
Colua, E	THAE0206^*^
Come, J	TUAC0204
Comstock, L	WEAC0202
Condo, J	THPDE0204
Contreras, D	THPDE0202
Contreras-Loya, D	THPDE0204
Conway, DP	FRAC0102
Cook, A	TUAB0204
Coombs, J-A	WEAB0103
Coombs, R	WEAE0304
Cooper, D	FRAE0105, THAB0202
Cooper, K	TUAX0101LB
Copas, A	WEAE0105
Corbelli, G	TUPDC0105
Corey, L	TUAX0102LB
Coris	WEPDB0105
Corrigan, B	THAD0103
Cossa, L	TUPDB0104
Costagliola, D	TUAC0203
Costiniuk, C	TUAA0103
Cotte, L	WEAC0102
Couderc, C	THAC0102^*^
Coulibaly, A	THAC0102
Coulter, R	TUAD0205
Cowan, F	TUAX0103LB^*^
Cowan, FM	WEAE0103, WEAE0105
Crampin, AC	WEAD0104
Crandall, B	THAE0205
Cranston, RD	TUAC0103
Crauwels, H	THAB0206LB
Crooks, E	WEPDA0101
Crosno, K	TUAA0101
Crouch, P-C	FRAE0104^*^
Cua, E	WEAC0102
Cuembelo, F	TUAE0103
Cummins, N	THAB0202
Curran, K	WEAE0203
Currier, J	THAB0103LB^*^, THAB0106LB
Curtis, P	WEAD0305
Custer, B	WEAA0106LB
Czaicki, N	THAD0201
**D**	
D’Angelo, L	WEAD0203
D’Aquila, R	THAX0103
Dabis, F	FRAC0105LB^*^
Daho, S	WEAE0301
Dalal, S	THAE0303
Dam, K	WEPDC0106^*^
Danboise, B	THPDB0203
Danel, C	WEAB0303
Dange, A	FRAC0101
Daniel, C	TUAD0101
Daniels, B	TUAX0102LB
Darbes, L	WEAD0102^*^
Darong, G	WEPDD0101
Darr, E	WEAB0301
Das Dores, C	TUAB0202
Daskalakis, D	FRAD0106LB
Daskilewicz, K	FRAD0102
Datong, P	WEAB0103^*^
Davenport, M	THAA0101^*^
Davey, C	TUAX0103LB
Davies, M-A	TUAB0104^*^
Davies, NECG	THPDC0105^*^
Davis, SLM	TUPDD0107LB^*^
Davis, W	WEPDE0203
Dawood, H	FRAE0102, WEPDB0104
Dawson, L	THPDD0105
Dayton, F	THAA0201
Ddaaki, W	TUAD0405, WEPDD0101, WEPDE0102
De Castro, D	TUPDD0201
De Gruttola, V	THAB0102
De Jong, BC	WEAB0205LB
de la Grecca, R	TUAD0404
de Roubaix, M	THPDD0101
De Oliveira, T	FRAC0105LB
de Oliveira, T	THAX0104^*^
de Pedro, AM	TUPDB0104
de Pokomandy, A	TUPDD0306
de Schacht, C	TUAE0103^*^
de Souza, M	TUAX0101LB
de Vries, H	WEAC0302
Decker, M	WEPDC0202^*^
Decroo, T	TUAB0202^*^
del Amo, J	WEPDB0105
Del Prete, G	THAA0101
Delany-Moretlwe, S	THAD0106LB, THAD0203, WEAD0204, WEPDC0206
Delate, R	TUPDD0202
Delaugerre, C	WEAC0102
Delfraissy, J-F	WEAC0102
Dellar, R	THAX0104
Dembé lé Keita, B	THAC0102
Demeri, D	FRAD0105
Denny, TN	THAX0105
der Sluis	THPDA0104
Desbiens, M	TUPDD0306
Desgress du Lou, A	TUAD0103
Deuba, K	TUAD0201^*^
Devarajulu Reddy, S	WEAB0201
Devieux, J	WEAE0202
Dewar, R	THAA0104LB
Deyde, V	TUAC0201
Dezembro, S	TUAB0202
Dezutti, C	TUAC0105LB
Dhilpe, V	THPDB0205
Dhodho, M	WEAE0301^*^
Di Giano, L	FRAD0204
Diaz Granados, C	TUAX0102LB
Diergaardt, C	TUPDE0102
Diez-Martin, JL	THAA0105
Dikgale, F	TUPDE0103
Dikobe, W	THAE0302
Dimba, A	WEAB0204
Dinapoli, S	TUAA0101
Dindi, P	THAE0202, WEAD0201^*^
Ding, E	TUPDD0306
Diop, AK	THAC0102
Diouf, D	TUAD0202
DiPaola, A	WEAC0402
Dirawo, J	TUAX0103LB
Diseko, M	THPDB0101
Ditekemena Dinanga, J	WEPDE0105^*^
Díaz, A	TUPDB0106
Dlamini, D	TUPDD0102^*^
Doehle, B	WEAB0301
Doerholt, K	TUAB0103
Doherty, T	TUAE0106
Dolezal, C	TUAB0101, WEPDB0103
Donaldson, E	THAE0106^*^
Donastorg, Y	TUAD0401
Doncel, GF	FRAE0102
Dong, K	WEAA0104
Dong, KL	TUPDA0104
Donnell, D	WEAC0105
Donnelly, A	WEAD0305
Dorey, D	THAB0206LB
Doré, V	WEAC0102
Doria-Rose, N	WEPDA0101
Dorvil, N	WEAE0202
Dos Santos, N	TUAB0202
Dougherty, G	WEAE0106LB^*^
Dow, W	THAD0201
Dowdy, D	WEAB0202
Dowsett, G	THPDD0103
Drain, P	THAE0204
Drame, F	TUAD0202
Dray-Spira, R	TUAD0103
Dreyer, J	FRAC0105LB
Dronda, F	TUPDB0106
Dube, F	WEPDD0105^*^
Dube, K	THPDD0102, THPDD0105^*^
Dube, M	WEPDD0105
Dubé, K	THPDD0104
Dubuc, D	TUPDD0306
Duby, Z	THPDD0101
Duck, T	WEPDE0204
Duerr, A	THAX0102
Duffill, K	TUAC0103
Dumont, E	WEAE0202
Dunkle, K	TUAD0203
Dunn, DT	TUPDB0105
Dunsmore, T	THPDA0103, TUAC0101
Durand-Zaleski, I	THAE0304^*^
Dutrieux, J	WEPDA0104^*^
Dutta, A	THAE0104
Duverger, L	WEAE0202
Dvoriak, S	THPDE0101
Dzekedzeke, K	THPDE0204
Dzissyuk, N	TUPDB0103^*^
Dzoro, S	WEPDA0103
**E**	
Eba, P	FRAD0101
Ebagua, I	WEPDE0104
Eckard, M	TUPDE0102
Ecochard, R	TUAC0202
Edelstein, H	THAB0203
Edick, S	TUAC0103
Egan, D	TUAC0103
Egan, J	WEAC0204
Ehmer, J	FRAE0202, WEAE0303
Eholié, SP	WEAB0303
Ehouman, S	THAC0102
Ehrenkranz, P	WEAE0206LB
Ekong, E	THAB0201
Ekström, AM	TUAD0201
El-Sadr, W	TUPDC0105, WEAE0206LB
Eley, B	TUAB0104
Elizabeth de Lima Pereira, M	TUPDD0201
Elkington, K	TUAB0101
Elkington, KS	WEPDB0103
Ellman, T	THAB0101, THPDC0103, TUAB0202, WEAE0302
Else, L	TUAC0103
Emel, L	THAC0105LB, WEAC0104
Emery, S	TUPDC0105
Emusu, D	WEPDC0104
Engstrom, J	TUAC0103
Erekaha, S	WEPDE0104
Ericsen, A	TUAA0101^*^
Ernest, O	WEAE0205
Eshleman, S	TUAC0102
Esiru, G	TUAE0102
Essex, M	TUPDC0103, WEAB0104, WEAE0305
Estes, J	THAA0101, WEAA0103
Etard, J-F	THPDC0103, TUPDB0104
Etima, J	WEPDC0203
Etyang, T	FRAB0102LB
Eubanks, K	TUAX0101LB
Euzébio de Lima, C	TUPDD0201^*^
Evans, D	THPDD0104^*^, WEAD0305
Evans, M	WEPDC0204
Evans-Frantz, I	WEAC0202
Ezouatchi, R	TUAD0202
**F**	
Faden, R	TUAD0301
Fagan, T	THAE0102^*^
Fahey, C	THAD0201
Fair, C	WEAD0203
Fairley, CK	FRAC0102
Falcæ, V	THAB0205LB
Familiar, I	THPDB0102
Fantus, S	TUPDD0204
Farley, S	THPDD0106LB
Farley, T	THAE0303
Farquhar, C	TUAE0103
Fast, D	TUAD0102
Fatti, G	TUAB0104, WEAE0104
Fauci, A	THPDA0101
Faustin, M	WEAE0202
Faye, S	THAE0203^*^
Fayorsey, R	WEAE0106LB
Fearon, E	TUAX0103LB
Felber, BK	THAA0201^*^
Fennessey, C	THAA0101
Fernandes, A	WEPDE0103
Fernandez Giuliano, S	TUPDB0102
Ferrand, RA	THPDB0105
Ferreira Santana, D	TUPDD0201
Ferrusi, C	FRAD0106LB
Fidler, S	WEAA0105LB
Fiebig, L	THAE0302
Fiedler, TL	WEPDC0201
Fields, S	THAC0105LB, WEAC0104
Fields, SD	WEAC0103
Fiellin, D	WEAC0401
Figueroa, ME	WEPDC0106
Figueroa, MI	FRAB0104LB
Finlay-Vickers, A	THAE0302
Finlayson, T	THAC0104
Fish, R	FRAE0101
Fisher, K	THAE0106
Fisher, M	WEAA0105LB
Flick, R	WEAB0204
Floyd, S	WEAB0205LB
Flynn, PM	THAB0202
Fonner, V	TUAC0104
Fonseca, E	FRAD0206^*^
Footer, K	TUPDD0106
Ford, S	THAB0206LB
Forster, N	THAE0103
Fortenberry, A	WEAC0202
Fortunak, J	THPDE0206
Fought, AJ	WEAA0101
Fowler, MG	THAB0106LB, THPDB0102
Fox, A	WEAD0305, WEPDC0103
Fox, J	THAB0202, WEAA0105LB
Fox, M	TUAB0205^*^
Fox, MP	TUAB0102, TUAC0205, WEAB0102, WEAE0204
Franck, D	TUAA0102
Frank, A	THPDB0204
Frank, S	THAE0204
Fraser, N	TUPDC0102
Frater, J	WEAA0105LB
Fredrick, LM	WEAB0304LB
Fredricks, DN	WEPDC0201
Freedberg, KA	THAE0305
Freeman, G	THAA0206
Frieden, M	WEAE0301
Friedland, G	FRAC0103, TUPDC0105
Friedrich, T	TUAA0101
Fritz, K	WEAD0102
Frohlich, J	TUAC0201
Fromentin, R	THPDA0102
Fung, K	TUAD0403
Furco, A	WEAB0205LB
Furrer, H	WEPDB0101
Fwamba, F	WEPDE0105
**G**	
Gabillard, D	WEAB0303
Gachuhi, A	WEAE0206LB
Gagliano, J	FRAE0104
Galai, N	WEPDE0203
Galarraga, O	THPDE0202^*^
Galárraga, O	THPDE0204
Galindo-Arandi, C	THAC0103
Galli, L	TUAB0103
Galperine, T	WEAB0205LB
Gane, E	WEAB0304LB
Gangakhedkar, R	THPDB0205
Gao, F	THAX0105
Gaolathe, T	TUPDC0103, WEAE0305
Gaoshan, J	TUAD0305
Garcia-Diaz, J	THAB0203
Garner, S	THPDD0104, THPDD0105
Garofalo, R	WEAC0203^*^
Garone, DB	THPDE0102
Garrett, N	TUPDA0101, TUPDA0105, WEAB0101^*^
Garrett, NJ	WEAA0102
Gaseitsiwe, S	TUPDC0103, WEAE0305
Gatei, J	WEAB0103
Gatell, J	TUPDC0105
Gathii, P	WEAE0203
Gauer Bermudez, L	THAD0205^*^
Gaufin, T	THAA0205
Gautam, R	THAA0201, THAA0205
Gaven, S	THPDE0102
Gede, S	TUPDE0102
Geffner, M	WEAB0105
Gehr-Seloover, A	TUAD0205
Gelman, M	FRAE0101
Gengiah, TN	FRAE0102
George, G	TUAC0201, WEPDC0204
Gerstoft, J	TUPDC0105
Getahun, M	FRAD0103
Ghate, M	THPDB0205
Ghebremichael, MS	TUPDA0104
Giaquinto, C	TUAB0103
Gibb, D	TUAB0103
Gibb, DM	FRAB0101LB, FRAB0102LB, TUAB0204
Gibbs, A	TUAD0203^*^, TUPDD0304^*^
Gibson, S	FRAE0104
Gichangi, P	TUAD0303
Giddy, J	TUAB0104
Gikaro, J	WEAE0205
Gilbert, P	TUAX0102LB
Gilbert, S	TUAD0301^*^
Gilbertson, A	THPDD0105
Gill, MM	WEPDE0105
Gimbel, S	TUAE0103
Giovenco, D	TUAD0104
Girard, P-M	WEAB0304LB
Giuliani, R	WEAE0302^*^
Glass, N	WEPDC0202
Glenshaw, M	TUAC0201
Glidden, D	TUAC0104
Gloyd, S	TUAE0103
Glynn, J	WEAB0205LB
Gobodo, N	TUPDE0102
Goetghebuer, T	TUAB0103
Goga, A	TUAE0106^*^
Goldstein, M	WEPDC0105
Golovin, S	FRAD0205
Golub, S	WEAC0202
Golub, SA	FRAC0101
Gomathi, NS	WEAB0201
Gomez-Olive, FX	WEPDC0205
Gonzales Dias, P	TUPDB0104
Gonzales-Zuñiga, P	WEAC0405
Goodall, R	TUAB0103
Goodier, S	TUPDA0101
Gordin, F	THAB0201
Gorelick, R	THAA0104LB
Gorgens, M	TUPDC0102
Gorman, J	WEPDA0101
Gosselin, A	THPDA0102
Gotham, D	THPDE0206^*^
Goulder, PJR	THAA0202
Gouse, H	TUPDD0205
Govender, K	TUAC0201
Govender, N	THPDB0201
Govere, S	THAE0204
Goverwa-Sibanda, TP	TUPDC0106^*^
Gómez-Ayerbe, C	TUPDB0106
Granich, R	WEAE0201^*^
Grant, C	FRAD0103
Grant, R	FRAE0104, TUAC0104^*^
Gray, C	WEAB0103
Gray, G	THAD0106LB, TUAX0102LB, WEPDC0206
Green, B	TUPDE0102
Greene, E	THAD0106LB
Greene, J	TUAA0101
Greene, S	THPDD0104, TUPDD0306
Greiger-Zanlungo, P	TUPDC0106
Griffith, S	THAB0206LB
Griffiths, A	FRAB0101LB, FRAB0102LB
Grinsztejn, B	THAC0101, THAE0305
Grobler, A	FRAE0102, THAX0104, TUAC0201, TUPDA0105, TUPDC0101^*^
Gross, J	TUAE0102
Gross, R	WEPDB0106
Grosso, A	THAE0105
Groves, AK	TUAD0204^*^
Gruhlich, A	TUPDC0105
Grulich, AE	FRAC0102, FRAE0105, THAC0101
Grund, B	THAB0201, THAB0202
Grund, J	WEPDC0104^*^
Grund, JM	THPDE0205
Grunenberg, N	TUAX0102LB
Guddera, V	FRAC0103
Gudukeya, S	WEAE0105
Guion, M	WEAB0301
Guise, A	TUAD0106LB
Gulick, R	TUAC0102^*^
Gulzar, N	THAX0105
Gumber, S	THPDA0101
Gun, A	FRAB0104LB
Gupta, S	WEAE0201
Gustafson, K	THAE0301
Gustav, R	WEAD0304
Gutierrez, E	TUPDD0206
Gutierrez, F	THAB0206LB, WEPDB0105
Guy, R	FRAE0105
Guy, RJ	FRAC0102
**H**	
Haacker, M	THAE0103
Haberer, J	THPDC0102, WEAC0105
Haberer, JE	THPDC0106
Hachiya, A	THAX0101
Hack, H	THPDB0203
Haddad, L	WEAD0101
Hader, S	WEAE0201
Hagins, D	THAB0205LB
Hagos, K	FRAD0106LB^*^
Hajiyiannis, H	TUPDD0202
Hakim, J	FRAB0101LB^*^, FRAB0102LB, TUPDB0105
Hall, C	FRAE0104
Hall, I	WEAE0201
Hamahuwa, M	THPDB0206
Hammond, R	WEAC0205
Hamunime, N	TUPDB0101, TUPDC0104
Hanisch, D	TUAX0103LB
Hankins, C	THAE0303, TUPDD0306
Hanley, S	THAB0106LB, THPDD0102
Hanrahan, C	TUAB0203
Hansudewechakul, R	THPDB0106
Harawa, K	THPDE0102
Haret-Richter, G	THPDA0103, TUAA0102, WEAA0103
Haret-Richter, GS	TUAC0101
Hargreaves, J	TUAX0103LB
Hariharan, N	TUAE0102
Harmon, T	THAE0106
Harrington, M	FRAD0106LB
Harrison, P	THAE0106
Harrison, T	THPDB0201
Hart, M	TUPDD0107LB
Hartson, K	TUAD0402^*^
Harwell, J	THPDE0203
Hattori, J	THAX0101
Hatzold, K	TUAX0103LB, WEAE0103, WEAE0105, WEPDC0106
Havens, PL	WEAC0305LB
Havlir, D	FRAE0205, WEAC0106LB
Havlir, DV	FRAE0203
Hayes, R	THPDE0205
Haynes, BF	THAX0105
Hazra, R	TUAB0104
He, T	THAA0205^*^, THPDA0103, TUAA0102
Hecht, J	FRAE0104, TUAD0105^*^
Hector, J	FRAE0202
Hedt-Gauthier, B	WEAE0202
Heffron, R	FRAE0106LB, THPDC0102^*^, WEAC0105
Hellar, A	THPDE0205
Hemanth Kumar, AK	WEAB0201
Hembling, J	WEAD0103
Hendricks, M	THPDD0101
Hendriks, S	THAD0202
Hendrix, C	TUAC0102, TUAC0105LB
Hennequin, W	THPDC0103, TUAC0202
Hennessey, K	WEAE0202
Henostroza, G	THPDE0104
Henry, E	THAC0102
Henry, M	TUPDD0205
Hensley-McBain, T	TUAA0106LB
Herbeck, J	THAX0102
Herbst, K	FRAC0105LB
Herce, M	TUAE0104
Hernando, V	WEPDB0105
Hernández-Quero, J	WEPDB0105
Heymann, SJ	THPDE0104
Hick, C	WEAB0104
Hickling, S	WEAA0105LB
Hickman, M	WEAC0405
Hicks, S	THAA0206
Hightow-Weidman, L	THAC0105LB^*^, THPDD0106LB, WEAC0104
Hill, A	THPDE0206
Hinson, K	THAD0106LB
Hoare, J	TUAD0104
Hobbins, M	WEAE0303
Hobbins, MA	FRAE0202
Hodder, S	TUAC0102
Hoddinott, G	WEPDD0104
Hodes, R	THAD0204, TUAB0201
Hoffman, R	THAB0103LB
Hoffmann, C	TUAD0205
Hoffmann, M	WEAA0105LB
Hogg, RS	TUPDD0306
Holden, J	WEPDE0204^*^
Holding, P	THPDB0101
Holele, P	TUAE0105
Holland, C	TUAD0302
Holloway, IW	WEAD0305^*^
Holme, MP	TUPDC0103, WEAE0305
Holmes, C	WEAE0101
Holmes, MC	THAA0103
Holt, M	FRAC0102
Honermann, B	THAE0105
Hong, JJ	THPDA0101
Hong, P	TUAD0305
Hong, S	TUPDB0101^*^
Hong, T	THAE0204
Hoosegood, V	WEPDD0101
Hoots, B	THAC0104
Hope, TJ	THPDA0105^*^, WEAA0101^*^, WEAA0102
Hopking, J	THAB0203
Hora, B	THAX0105^*^
Hosegood, V	WEAD0102
Hosek, S	TUAX0104LB^*^, TUPDD0203, WEAC0305LB
Hossain, S	TUPDD0305
Hossain, T	TUPDD0305
Hosseinipour, M	WEAB0204
Htee Khu, N	THPDC0101, WEAD0101
Htun, S	TUPDD0301
Hu, S-L	THAA0103
Hu, W	THAA0102
Hu, X	THAA0201
Hu, Y	FRAD0203
Hu, YB	WEAB0304LB
Huang, KC	WEAB0301
Huang, L	THAA0106LB^*^
Huang, M	THAE0204
Hucks-Ortiz, C	WEAC0103^*^
Hudson, PF	WEPDB0106
Huerga, H	THPDC0103
Huerta, L	TUAD0404
Huetter, G	THAA0105
Huffman, F	TUPDA0106
Hughes, J	WEAC0303, WEPDC0205
Hui, C	TUAD0403^*^
Hunt, G	THAX0104
Hunt, P	THPDC0106
Hunter, E	THPDE0201
Hurst, J	WEAA0105LB
Hurt, C	THAC0105LB
Hyle, E	THAE0204
**I**	
Ibrahim, F	WEPDD0103
Igonya, E	TUAD0106LB^*^
Ilunga, V	WEPDE0105
Imakit, R	WEAD0202^*^
Imrie, J	WEAD0204
Inamba, M	THPDE0201, THPDC0101
Ingleby, C	THPDE0105^*^, WEAA0106LB
Innes, C	TUAX0102LB
Intasan, J	TUAX0101LB
Iovita, A	TUPDD0106
Irani, L	THAE0202, WEAD0201
Irwin, R	WEAD0304
Isaacsohn, M	THAD0204
Ishimwe, A	TUPDE0104
Ismail, N	THAA0202, TUPDA0104, WEAA0104
Iwuji, C	FRAC0105LB
Izazola-Licea, JA	THAE0106
**J**	
Jackson, D	TUAE0106
Jacobs, D	TUPDE0103
Jacobson, JC	THPDA0104
Jadhav, S	THPDB0205
Jadwattanakul, T	WEPDE0205
Jagessar, N	THAD0202^*^
Jagodzinski, L	THPDB0203
Jain, S	THAA0101
Jain, V	FRAE0203, WEAC0106LB
Jakubowski, A	FRAE0205
Jalan, P	THAE0301
Jama-Shai, N	TUAD0203, TUPDD0304
Jamil, MS	FRAC0102^*^
Janamnuaysook, R	WEPDE0205
Jansen, M	THAB0104
Jantarapakde, J	WEPDE0205
Janyam, S	WEPDE0205
Jao, J	WEAB0105^*^
Jaoko, W	WEPDC0201
Jarvis, J	THPDB0201
Jaspan, H	WEAB0103
Jefferys, LF	FRAE0202^*^
Jennings, L	THAD0205
Jewell, N	THAD0201
Jewkes, R	TUAD0203, TUPDD0304
Ji, GP	WEAD0105
Jibril, H	THPDB0101
Jin, F	THAC0101
Jitjang, S	WEPDE0205
Joao, E	THAB0103LB, THAB0106LB
Jofrisse, M	TUAB0202
John-Stewart, G	WEPDC0201
Johns, B	THAE0203
Johnson, MO	WEAD0102
Jones, B	WEPDA0102
Jordan, M	TUPDB0101
Joseph Davey, D	THPDC0101^*^
Joseph, J	TUAE0102^*^
Josiah, R	WEAE0205
Joska, J	TUPDD0205
Ju, S	WEPDC0206
Judd, A	TUAB0103^*^, WEAC0301
Justice, A	WEAC0401
Justman, J	WEAE0205
**K**	
Kabahenda, S	FRAB0102LB
Kabakyenga, J	THPDC0106
Kabami, J	FRAE0205, WEAC0106LB
Kabanga, JD	TUPDD0105
Kabore, SM	FRAE0204
Kabunga, E	TUAD0304
Kacanek, D	WEAB0105
Kadede, K	WEAC0106LB
Kadiyala, S	THAD0201
Kadzandira, J	TUPDD0103
Kahabuka, C	WEPDC0106
Kahn, JG	FRAE0203
Kahn, K	TUPDD0303, WEAC0303, WEPDC0205
Kaida, A	THPDC0106^*^, TUPDD0306
Kaimal, A	THAB0105
Kaizer, S	WEPDA0102
Kaldor, JM	FRAC0102
Kalombo, C	FRAE0206LB
Kamanga, E	TUAE0104^*^
Kamateeka, M	THAB0106LB
Kamath, C	FRAC0101
Kambugu, A	TUPDB0105
Kamenova, K	WEAE0303
Kaminski, R	THAA0102
Kammerer, B	THPDB0101
Kamonga, M	WEAE0106LB
Kamya, M	FRAE0205, WEAB0202, WEAC0106LB
Kamya, MR	FRAE0203
Kanesa-Thasan, N	TUAX0102LB
Kann, L	WEAC0304^*^
Kaplan, R	FRAB0103LB
Kapogiannis, B	TUAX0104LB
Kapogiannis, BG	WEAC0305LB
Kapologwe, N	THAD0201
Kappler, J	WEPDA0102
Karagiannis, K	THAX0105
Karim, F	TUAA0103
Karita, E	THPDE0201
Karki, DK	TUAD0201
Karn, J	THAA0102
Karoney, M	FRAB0101LB
Karuna, S	THAD0106LB
Kashuba, ADM	TUAC0101
Kaski, JP	THPDB0105
Katabira, E	FRAE0103, FRAE0106LB, THPDC0102, WEAC0105
Katende, J	WEAB0202
Katlama, C	THAB0202
Katlholo, T	THAE0302
Katz, I	WEAE0204
Katze, M	WEAA0103
Kaufman, M	WEPDC0106
Kaufmann, D	WEPDA0102
Kaunda, S	FRAB0101LB
Kaunda-Khangamwa, B	TUPDD0103
Kavanagh, M	FRAD0201^*^
Kawalazira, R	THPDB0102
Kayuni Chihana, N	TUAE0101
Kazatchkine, C	FRAD0101
Kazaura, K	WEAE0205
Kazembe, P	WEAB0204
Kedem, E	THAB0202
Keele, B	THAA0101, WEAA0103
Keele, BF	TUAC0101
Keen, P	FRAC0102, WEPDE0204
Kegoli, S	TUPDE0106
Keiser, O	TUAB0104
Kelleher, AD	THAA0203
Kennedy, C	TUAD0401
Kent, SJ	THAA0203
Kerr, T	TUAD0102
Kerrigan, D	THAB0204^*^, TUAD0401, WEPDE0203
Kerschberger, B	FRAE0204
Kestler, M	TUPDD0306
Ketende, S	TUAD0202, TUAD0302
Kew, M	WEPDB0102
Keyser, V	TUAB0203
Kgwaadira, B	THAE0302^*^
Khalili, K	THAA0102^*^
Khan, N	TUPDC0103
Khanyile, D	TUAC0201, TUPDC0101
Kharsany, A	THAX0104, TUAC0201^*^, TUPDA0105, TUPDC0101
Khasoane, M	TUAD0402
Khoo, S	TUAC0103
Khopkar, P	THPDB0205
Khoury, G	THPDA0104
Khoza, N	THAD0203^*^, TUPDD0303
Khumalo, P	THAX0104
Khumalo, T	TUAD0203, TUPDD0304
Kiarie, J	FRAE0106LB
Kidoguchi, L	WEAC0105
Kieffer, MP	WEPDE0201
Kiem, H-P	THAA0103
Kiesling, A	TUPDB0101
Kijak, G	TUPDA0102
Kilembe, W	THPDC0101, THPDE0201, WEAD0101
Kim, J	TUPDA0102
Kim, M	WEAB0204
Kimambo, S	WEAE0106LB
King, A	WEPDD0106
King, AJ	WEAD0305
King, C	FRAD0106LB
King, D	THAA0204
Kinloch, S	WEAA0105LB
Kinuthia, J	WEPDC0201
Kiragga, A	THAB0105
Kiriazova, T	THPDE0101^*^
Kirimo, M	WEPDD0103
Kirui, M	WEPDC0105
Kiswi, N	WEPDC0105
Kitheka, M	WEAE0203^*^
Kituku, A	WEPDE0101
Kityo, C	FRAB0101LB, FRAB0102LB^*^, TUAB0204 TUPDB0105
Klatt, N	TUAA0106LB^*^
Kleinman, A	THPDA0103
Klingman, K	TUAC0102
Klingman, KL	THAB0202
Klinker, HHF	WEAB0304LB
Koenig, S	WEAE0202^*^
Kolstee, J	FRAC0102
Kone, A	TUAE0103
Koofhethile, CK	THAA0202^*^
Koole, O	WEAD0104
Korenromp, E	THAE0103
Kosalaraksa, P	THPDB0106
Kose, J	THAD0103
Kose, Z	TUAD0302
Koteff, J	THAB0203
Kotokwe, KP	TUPDC0103
Kottilil, S	WEAB0301
Kouamé, A	TUAD0202
Kouamé, MG	WEAB0303^*^
Kourtis, A	WEAB0305LB^*^
Krampe, N	THPDA0103
Kranzer, K	THPDB0105
Kreh, L	TUPDD0203
Kriek, J-M	WEAA0102
Kripke, K	THAE0303
Krishnan, A	WEAC0402
Kroon, E	TUAX0101LB
Krows, M	THAE0204
Krubiner, C	TUAD0301
Kuate, L	THAE0302
Kuball, J	THAA0105
Kufa-Chakeza, T	TUAC0201
Kuhns, L	WEAC0203
Kulkarni, S	THPDB0205^*^
Kumar, P	THAB0203
Kumar, S	WEAB0201
Kundi, G	WEAE0205
Kuo, C	TUAD0104^*^
Kurani, S	WEAD0306LB
Kurauone, W	TUPDC0106
Kuringe, E	THPDE0205
Kwan, A	THPDE0204
Kwarisiima, D	FRAE0203
Kwarsiima, D	WEAC0106LB
Kwekwesa, A	THPDE0102^*^
Kwon, DS	TUPDA0104^*^
Kwon, M	THAA0105
Kwong, P	WEPDA0101
Kyriakides, T	FRAC0103
**L**	
La Eller	TUPDA0102
La Hera-Fuentes, G	THPDE0204
Lachowsky, N	TUPDD0204
Lafort, Y	TUAD0303
Laher, F	TUAX0102LB
Lai, W	THAA0106LB
Lake, JE	THAB0203
Laker Agnes Odongpiny, E	THAB0105^*^
Lakhani, I	TUPDD0102
Lakshmi, M	WEAB0201
Lalloo, U	TUAA0103
Lama, JR	TUAD0404
Lamb, M	WEAE0206LB
Lambert, A	TUAD0302
Lamorde, M	TUAB0204
Lamothe-Molina, P	WEPDA0102
Lampe, F	TUPDC0105
Lampinen, J	THPDB0204
Landay, A	TUAA0102
Landovitz, R	TUAC0102, TUAX0104LB
Landovitz, RJ	WEAC0305LB
Larke, N	THPDE0205
Larmarange, J	FRAC0105LB
Latt, NZ	TUPDD0301
Lauck, M	TUAA0101
Laurent, C	THAD0101, THAC0102
Lavanya, J	WEAB0201
Lavoy, G	WEAC0106LB
Law, M	FRAC0102, THAB0201
Lawson, B	THAA0206
Lazar, L	THAE0105^*^
Lazzarin, A	WEAB0304LB
Le Coeur, S	TUAB0103
Le Guen, M	TUAD0103
Le Mestre, S	WEAC0102
Leask, K	TUPDA0105, WEAB0101
Leavitt, R	FRAB0103LB
Leckie, G	THPDB0204
Leddy, A	WEPDE0203
Lee, E	TUAA0106LB, WEPDC0105
Leenasirimakul, P	WEPDE0205
Lehrer-Brey, G	TUAA0101
Leibowitz, A	WEAD0305
Leidner, J	THPDB0101^*^, WEAB0104
Lelutiu-Weinberger, C	FRAC0101
Lemp, G	WEAD0305
Leon-Fuentes, L	TUAA0105
Leonard, W	TUPDE0104^*^
Leroy, V	WEAC0301
Lert, F	TUAD0103
Leslie, A	TUPDA0104
Letsatsi, V	WEPDB0106
Leu, C-S	WEPDB0103
Leu, CS	TUAB0101, TUPDD0205
Lewin, SR	THPDA0104, TUAX0101LB
Lewinsohn, D	TUAA0103
Li, AT-W	TUAD0403
Li, C	TUAD0305^*^
Li, L	TUAD0305, WEAD0105^*^
Li, P	WEPDC0105
Li, S	TUPDA0103
Liang, L-J	WEAD0105
Liang, S	WEAB0305LB
Liang, X	TUAD0305
Licata, M	TUPDD0104
Liebenberg, L	TUPDA0105^*^
Liebenberg, LJ	WEAA0102
Liegler, T	TUAC0104, WEAC0106LB
Liestman, B	TUAD0202
Lifson, J	THAA0101
Lija, G	THPDE0205, WEPDC0106
Likindikoki, S	WEPDE0203
Lin, CQ	WEAD0105
Lin, J	TUAD0105
Lin, SY	TUPDD0306
Lindsey, K	THAE0105
Ling Xu, C	WEAA0103
Lingappa, JR	WEPDC0201
Lingjongrat, D	WEPDE0205
Lipkey, L	THAA0101
Lippman, S	TUPDD0303
Little, D	THPDA0101
Little, M	TUAD0301, WEPDC0103^*^
Liu, A	FRAE0101
Liu, C	WEAE0102
Liu, F	WEAE0102
Liu, N	TUAX0104LB, WEAC0305LB
Liu, W	WEAB0305LB
Livingston, EG	WEAB0105
Loando, A	WEPDE0105
Lockman, S	THPDB0101, TUPDC0103, WEAB0104, WEAE0305
Logan, L	WEAE0306LB
Logie, C	TUAD0101^*^
Lolekha, R	THPDB0106
Lombard, C	TUAE0106
Londhe, R	THPDB0205
Long, C	TUAD0102
Losina, E	THAE0305
Losso, M	THAB0103LB, THAB0201, THAB0203
Loutfy, MR	TUPDD0306
Lowrance, DW	TUPDC0104
Lozupone, C	TUPDA0103
Lu, HK	THPDA0104
Lucas, G	WEPDB0101
Lucas, J	THAD0106LB^*^
Lucas, JP	WEAC0103
Luchters, S	TUPDD0301
Lucke, J	THPDD0103
Luetkemeyer, A	WEAB0301, WEAB0302^*^, WEAB0304LB
Lugemwa, A	FRAB0101LB, FRAB0102LB
Lukhele, N	FRAE0204
Lukoda, N	THAE0205
Lundgren, J	THAB0201
Luphala, P	TUPDC0104
Luthuli, N	THAD0106LB
Lutkam, D	WEAE0106LB
Lutz, T	THAB0206LB
Luyombya, H	TUAD0403
Luz, PM	THAE0305
Lydié, N	TUAD0103
Lyerly, A	TUAD0301
Lyon, W	FRAE0104
Lyons, A	THPDD0103
Lyons, C	THAE0105, TUAD0202^*^, WEAD0306LB
**M**	
Ma, D	THPDA0103, TUAA0102, TUAC0101, WEAA0103
Maarifi, G	WEPDA0104
Maartens, G	WEAB0203
Maartens, T	TUPDE0103
Mabhele, S	TUPDD0104
Mabhula, A	TUPDA0104
Mabirizi, D	TUPDB0101
Mabuku, I	TUPDC0104
Mabuse, R	TUPDE0103^*^
MacAllister, J	THAE0105, WEAD0306LB^*^
Macharia, P	WEPDC0105
Machekano, R	WEPDE0105
MacKellar, D	TUAC0204, WEAE0205^*^
MacLean, RL	THAE0305
MacLeod, W	TUAB0102, TUAB0205, TUAC0205
MacLeod, WB	TUPDC0102^*^
MacPhail, C	THAD0203, TUPDD0303, WEAC0303, WEPDC0205
Maculuve, B	TUAC0204
Madanhire, C	WEAE0103^*^
Madevu-Matson, C	WEAE0106LB
Madi, J	FRAC0103
Madondo, T	FRAC0103
Madurai, L	THAX0104, TUAC0201, TUPDC0101
Mafwenko, M	WEAE0101
Magaia, A	WEAE0302
Maganga, L	TUPDA0102
Magasana, V	TUAE0106
Magetse, J	THPDB0101
Magnus, M	THAC0105LB, WEAC0104
Magnuson, D	TUAX0105LB
Magure, T	WEPDD0105
Magwende, G	THPDE0104
Maharaj, P	TUAA0103
Maher, AD	TUPDC0104
Mahilmaran, A	WEAB0201
Mahlasela, L	TUPDD0202, WEPDC0106
Mahler, H	THPDE0205
Maina, M	WEPDC0105
Maitland, D	TUAD0403
Maitland, K	FRAB0101LB
Majola, N	WEAB0101
Majonga, ED	THPDB0105^*^
Makeleni, N	TUPDE0102
Makhema, J	THPDB0101, TUPDA0106, WEAB0104
Makhema, JM	TUPDC0103, WEAE0305
Makokha, M	THPDE0205
Makori, J	TUPDE0105
Makowa, T	FRAC0105LB
Malahleha, M	TUAX0102LB
Maldarelli, F	THAA0104LB
Maliwichi-Senganimalunje, L	THPDB0102
Mallewa, J	FRAB0101LB, FRAB0102LB
Mallewa, M	THPDB0102
Mallick, R	WEAB0103
Maluwa, M	THAB0106LB
Maman, D	THPDC0103^*^, TUAC0202^*^
Maman, S	FRAC0104, TUAD0204
Mamba, S	FRAE0204
Mamede, J	THPDA0105
Man, C	THAB0205LB
Manak, M	THPDB0203^*^
Mandaliya, KN	WEPDC0201
Manjezi, N	WEAE0104
Manson, K	THAD0103
Mansoor, LE	FRAE0102^*^
Mantell, J	TUAD0303
Mantsios, A	THAB0204, WEPDE0203^*^
Manuel, R	TUPDB0104
Manuzak, J	TUAA0106LB
Mao, J	WEAE0102
Maphorisa, CN	WEAE0305
Maphosa, T	TUPDC0106
Maponga, T	WEPDB0102^*^
Maradan, G	THAD0101
Marcell, A	WEPDC0106
Margolis, D	THAB0204, THAB0206LB^*^, THPDD0105
Marinda, E	THAE0206
Marlink, R	TUPDA0106
Marrone, G	TUAD0201
Marshall, B	WEAC0401^*^
Martin, GE	WEAA0105LB^*^
Martin, JN	THPDC0106
Martin, MA	THAA0201
Martin, N	WEAC0405
Martin, NK	WEAC0404
Martinez, E	THAB0202
Martinez, M	TUPDB0102
Martinez, S	TUPDA0106^*^
Martinez-Picado, J	THAA0105
Martinson, N	THPDE0204
Marty, L	TUAC0203^*^
Maruyama, H	WEAE0205
Marx, PA	WEAA0101
Marzinke, M	THPDC0102, TUAC0102, TUAC0105LB, WEAC0105
Masching, R	WEPDD0106
Mascola, J	WEPDA0101
Mashamaite, S	THPDB0201
Masheto, G	THAB0103LB, THAB0106LB
Masiku, C	FRAE0201, THPDC0103
Masiye, F	THPDE0204, TUPDD0103
Maskew, M	TUAB0102^*^, TUAB0205, TUAC0205
Masters, S	FRAC0104
Mastroianni, A	TUAD0301
Masuka, N	TUAX0103LB, TUPDC0106
Masungo, T	TUPDC0106
Masvawure, TB	TUAD0303^*^
Mateyu, G	THPDE0102
Mathews, A	THPDD0106LB^*^
Mathews, C	TUAD0104
Mathias, A	TUPDD0206^*^, WEAB0302
Matias, E	WEAA0101
Matthews, D	WEAC0204
Matthews, LT	THPDC0106
Mattur, D	THAE0106
Maughan-Brown, B	WEPDC0204^*^
Mavedzenge, SN	WEAE0103
Mavhu, W	WEPDC0106
Mayaud, P	WEAD0204
Mayer, G	TUAX0105LB
Mayer, K	FRAE0101, THAB0102, THAC0105LB, TUAC0102, TUPDD0203, WEAC0104
Mayer, KH	TUAD0404
Maylin, S	WEAB0303
Mayondi, G	THPDB0101
Mazibuko, G	TUPDB0101
Mazibuko, S	FRAE0204, WEAE0206LB
Mazumder, R	THAX0105
Má Rodríguez	TUPDB0106
Mbatha, N	TUAD0203, TUPDD0304
Mbilinyi, D	WEAE0205
Mbonze, N	WEPDE0105
Mboya, E	WEPDC0104
Mbwambo, J	WEPDE0203
Mc Grath, N	FRAC0105LB
McCallister, S	TUAX0105LB
Mccarthy, E	THPDE0203
McCarthy, K	THAB0106LB
McCauley, M	TUAC0102
McClair, T	TUPDD0305^*^
McClelland, RS	WEPDC0201^*^
McClure, C	WEAA0106LB
McCoy, S	WEAE0105
McCoy, SI	THAD0201^*^
McCracken, J	FRAD0105
McElrath, J	TUAX0102LB
McFarland, W	TUPDC0104
McGinn, E	THAE0202, WEAD0201
McGinnis, K	WEAC0401
McGowan, I	TUAC0102, TUAC0103^*^
McGrath, NM	WEAD0102
McHenry, M	THPDB0103
Mchugh, G	THPDB0105
McHutchison, JG	WEAB0301
McIlleron, H	WEAB0205LB
Mcingana, M	TUAD0302
McKay, M	THAD0205
McKinnon, LR	TUPDA0105, WEAA0102
McLean, E	TUAD0405, WEAD0104, WEPDE0102^*^
McLean, S	TUAD0404
McLean, T	WEAB0301
McManus, T	WEAC0304
McNairy, M	WEAE0206LB^*^
McNally, J	WEAB0301, WEAB0302
McNaughton Reyes, HL	TUAD0204
McNulty, AM	FRAC0102
McRaven, M	WEAA0102
Mdluli, C	THPDB0101
Mduluza, T	WEPDA0103^*^
Meacher, P	WEAC0202
Meanley, S	WEAC0204
Meer, T	FRAD0102
Mehraj, V	THPDA0102
Mehta, S	WEPDC0107
Meintjes, G	WEAB0203
Mekonen, T	TUPDB0101
Mellins, C	THAD0205
Mellins, CA	TUAB0101^*^, TUPDD0205, WEPDB0103^*^, WEPDE0106LB
Mellish, M	THAE0202, WEAD0201
Mellouk, O	FRAD0204
Mendiharat, P	TUAC0202
Mendizabal-Burastero, R	THAC0103^*^
Menon, PA	WEAB0201
Menon, V	THAE0102
Mera, R	TUAX0105LB
Merle, CS	WEAB0205LB^*^
Mermin, J	WEAE0201
Mesquita, F	THAE0305^*^
Metcalf, C	TUAB0202
Metcalfe, J	THPDB0105
Mewalal, N	WEAA0104
Meyer, L	THAE0304, WEAC0102
Meyerowitz, J	WEAA0105LB
Mgodi, N	THAD0106LB
Mhembere, T	THAB0106LB
Mhlane, Z	TUAA0103
Mhlongo, B	THAE0204
Michael, N	TUAX0102LB, TUPDA0102
Middlecote, C	THAE0301, THPDE0203
Milanga, M	WEAD0302
Miller, C	TUAA0106LB
Millett, G	THAE0105, WEAD0306LB
Mimiaga, M	THAB0102, TUPDD0203, WEAC0203
Mirembe, B	TUAE0102
Mitchell, C	TUAC0103
Mitha, M	TUAA0103
Mkhize, L	THAA0202
Mkwamba, A	TUAB0202
Mlisana, KP	THPDB0202
Mlongo, R	TUPDE0105
Mmalane, M	TUPDC0103, WEAB0104
Mncube, Z	THAA0202
Mngadi, K	TUAX0102LB
Mngadi, KT	FRAE0102
Moabi, K	THPDB0101
Mobaracaly, MR	THPDE0103
Mocroft, A	WEPDB0101^*^
Mofelehetsi, S	TUAD0402
Mofolo, I	TUAE0104
Mogalian, E	WEAB0302
Mogambery, JC	WEPDB0104^*^
Mogashoa, M	TUAE0105
Moh, R	WEAB0303
Mohammed, P	THAB0104
Mohammed, T	TUPDC0103, WEAE0305
Mohapi, L	THAB0104
Mohns, M	TUAA0101
Mokganyetji, T	TUPDD0303
Mokgatlhe, L	WEPDB0106
Molfino, L	TUPDB0104, WEAE0302
Molina, J-M	FRAB0103LB, WEAC0102^*^, THAB0201*
Molina, JM	THAE0304, TUPDC0105
Moll, A	FRAC0103
Money, D	TUPDD0306
Mongi, A	WEPDD0103
Montague, C	FRAE0102
Montalvo Pacahuala, CDP	WEPDD0106^*^
Montero, M	WEPDB0105
Montgomery, ET	WEPDC0203
Moodeley, A	TUPDA0104
Moodie, Z	TUAX0102LB
Moodley, A	WEAA0104, WEPDB0104
Moodley, D	TUAD0204
Moodley, K	THPDD0101^*^
Moodley, V	TUAA0103
Moonga, CN	THPDE0104^*^
Moosa, M-Y	THAE0204
Moraka, NO	WEAE0305
Morales, F	WEAE0205
Moreno, A	TUPDB0106
Moreno, S	WEPDB0105, TUPDB0106^*^
Morgan, E	THAX0103^*^
Morin, S	WEAD0305
Mortimer, J	WEAD0305
Morton, J	WEAC0105
Mosepele, M	WEPDB0106^*^
Moshabela, M	TUAD0405, WEPDD0101^*^
Mosher, S	WEAC0202
Moss, W	THPDB0206
Mota, TM	THPDA0104^*^
Mothibi, E	WEAE0104^*^
Motoku, J	TUPDE0106
Motuba, T	TUPDD0202
Moyo, S	TUPDA0106, TUPDC0103^*^, WEAE0305^*^
Mpofu, A	WEPDD0105
Mpofu, D	WEPDE0201
Mpoloka, WS	WEPDA0103
Mrus, J	THAB0206LB
Mshana, G	THPDE0205
Msuka, S	WEAE0106LB
Mtema, O	THAE0202^*^, WEAD0201
Mtetwa, S	TUAX0103LB
Mtileni, T	TUAE0105
Mtiro, H	WEAE0106LB
Mubiana-Mbewe, M	THAB0106LB
Mudany, M	WEAE0203
Mudenda, C	THPDE0104
Mudzviti, T	THAD0102
Muessig, K	THPDD0106LB
Mufuka, J	WEAE0105
Muga, R	WEPDB0105
Mugabe, D	TUAC0204
Mugisha, V	WEAE0106LB
Mugo, M	THPDE0202
Mugo, N	FRAE0106LB, THPDB0104, THPDC0102, WEAC0105
Mugurungi, O	TUAX0103LB
Mugwanya, K	FRAE0106LB
Mugyenyi, P	FRAB0101LB
Mujuru, H	THPDB0105
Mukasa, S	TUPDD0106
Mukui, I	THPDC0103, TUAC0202, TUPDB0104
Mukuye, A	THAE0205^*^
Muldoon, KA	WEAB0103
Mulenga, J	THPDC0101, THPDE0201
Mulenga, V	TUAB0204
Mulhern-Pearson, C	WEAD0305
Mullick, S	THPDC0105
Mulligan, K	WEAC0305LB^*^
Mullins, J	THAX0102
Mullins, JI	THAA0201
Mumba, O	WEAD0301
Munch, MM	WEPDC0201
Munderi, P	THAB0104^*^
Munroe, D	THAB0201
Munthali, A	TUPDD0103^*^, WEAB0204
Mupfumi, L	TUPDC0103, WEAE0305
Muriithi, C	TUPDE0106
Murphy, EL	WEAA0106LB
Murray, M	THAB0204
Murungu, J	WEAE0303
Musara, P	WEPDC0203
Mushati, P	TUAX0103LB
Musiime, V	FRAB0101LB, TUAB0204
Musingila, P	WEPDC0104
Musonda, RM	TUPDC0103, WEAE0305
Musoro, G	FRAB0102LB
Musyoki, H	WEAC0403
Mutaganzwa, A	TUPDE0104
Mutambanengwe, M	THAB0106LB
Mutenda, N	TUPDB0101
Mutevedzi, T	THAB0102
Mutisya, I	TUPDE0106^*^
Mutschler, J	TUAA0101
Mutunga, L	TUAB0203^*^
Mutuon, P	THAE0304
Muwonge, TR	FRAE0103
Müller, A	FRAD0102^*^
Mwai, D	FRAE0203, FRAE0205
Mwakangalu, D	TUPDE0105
Mwale, G	TUAE0104
Mwale, M	TUAE0104
Mwamkita, D	WEPDC0105
Mwampashi, A	WEPDE0203
Mwamzandi, Y	TUPDE0105
Mwangwa, F	FRAE0203, FRAE0205, WEAC0106LB
Mwanza, F	WEAD0302
Mwaringa, S	FRAB0102LB
Mwebe, S	WEAB0202
Mweemba, A	TUPDB0105
Mwelase, N	THAB0201
Mwero, BS	TUPDE0105
Mwinga, S	TUPDB0101
Myer, L	FRAE0206LB, TUPDD0205
Myers, L	TUPDD0202^*^
Mylvaganam, G	THAA0206
**N**	
N’takpé, JB	WEAB0303
Nabaggala, SM	THAB0105
Nabunya, P	THAD0205
Nadella, P	THAA0101
Naeveke, A	THAE0106
Nagashbekova, G	TUPDB0103
Nagashima, M	THAX0101
Naggie, S	WEAB0301
Naicker, N	WEAB0101
Naidoo, S	WEAC0302^*^
Naik, S	WEAB0302
Nair, G	THPDD0101
Nakanyala, T	TUPDB0101, TUPDC0104^*^
Nakku-Joloba, E	FRAE0103
Nakpor, T	WEPDE0205
Nalwanga, D	THAB0105
Namadingo, H	WEAD0104
Namakula, A	WEAD0202
Namey, E	TUAD0301
Namutamba, D	WEAD0303^*^
Nandelenga, R	WEAD0303
Napierala Mavedzenge, S	FRAC0104, TUAX0103LB, WEAE0105
Nardone, A	WEAE0306LB
Narendran, G	WEAB0201^*^
Natanael, S	TUPDB0101
Natha, M	WEAB0301, WEAB0302
Nathoo, K	FRAB0102LB
Navas, E	TUPDB0106
Ncheke, T	TUAD0402
Ncube, G	WEPDC0106
Ndabambi, N	TUPDA0101
Ndayizeye, N	TUPDD0105
Ndeogo, TS	FRAD0104^*^
Ndhlovu, Z	THAA0202, WEAA0104^*^
Ndiaye, A	WEAB0205LB
Ndimbii, J	TUAD0106LB
Ndindi, H	THPDE0102
Ndirangu, J	TUPDE0103
Ndlovu, S	FRAE0204
Nduati, R	TUAE0103
Ndun’gu, T	WEAA0104
Ndunda, E	THAD0202
Ndung’u, T	THAA0202, TUAA0103, TUPDA0104
Neaton, J	TUPDC0105
Neduzhko, O	THPDE0101
Neff, C	TUPDA0103
Nega, M	THAA0206
Nelson, L	THAC0105LB, WEAC0104
Nelson, M	WEAB0304LB
Nelson, R	TUAC0204^*^
Newell, M-L	FRAC0105LB
Newman, L	THAA0101
Ng’oma, K	TUAE0105^*^
Ngandu, N	TUAE0106, TUPDA0101
Ngarivume, K	TUPDC0106
Ngauv, B	THAE0301
Ngcapu, S	WEAA0102^*^
Ngirabega, JD	TUPDE0104
Ngobeni, S	WEPDD0102
Ngubane, T	WEAD0102
Ngure, K	THPDB0104, THPDC0102, WEAC0105
Nguyen, B-Y	FRAB0103LB
Ngwira, B	TUPDD0103
Nicholas, S	FRAE0201, TUPDB0104
Nichols, S	THPDB0101
Nicholson, V	TUPDD0306
Nielsen, M	TUAA0103
Nijhuis, M	THAA0105
Nikiforov, A	TUAC0103
Nininahazwe, C	TUPDD0105
Nisole, S	WEPDA0104
Nitayaphan, S	TUPDA0102
Nitschke, A-M	THAE0103
Njage, M	THAD0104
Njamwea, B	THAD0104
Njau, P	THAD0201
Njeuhmeli, E	THAE0303^*^, WEPDC0106
Njoroge, A	TUPDE0106
Njuguna, N	THPDB0104^*^
Nkomo, B	THAE0302
Nkosi, T	WEAA0104
Nofemela, A	FRAE0206LB^*^
Noguera Julian, A	TUAB0103
Nomsenge, S	WEPDD0104^*^
Nordstrom, S	WEPDC0107
Noriega, S	WEPDE0205
Norman, E	WEAB0101
Noveve, N	TUAE0106
Novitsky, V	TUPDC0103, WEAE0305
Nowacki, A	WEAE0102
Nsanzimana, S	THPDE0204
Ntema, C	TUPDC0104
Ntini, N	TUAD0203, TUPDD0304
Ntombela, F	FRAE0102, THAD0106LB
Nuwagaba-Biribonwoha, H	WEAE0206LB
Nwokolo, N	WEAA0105LB
Nyaboke, I	WEPDC0102^*^
Nyaguara, A	THAD0104
Nyakato, M	THPDB0102
Nyakerario Omare, J	WEPDE0101^*^
Nyaku, A	THAX0103
Nyakundi, H	THPDE0202
Nyamukapa, C	TUAD0405, WEPDD0101
Nyanchoka, J	WEPDC0105
Nyangweso, N	WEPDC0105
Nyathi, M	THAB0106LB
Nyirenda, C	WEAD0104
Nyombe, C	WEPDE0105
Nyondo-Mipando, L	FRAB0102LB
Nzaro, M	TUPDE0105
**O**	
O’Brien, N	TUPDD0306
O’Connor, D	TUAA0101
O’rie, T	TUPDE0102
Oberth, G	WEAD0301^*^
Obonyo, B	FRAC0104
Obura, N	WEPDC0105
Odhiambo, F	THAD0104
Odland, JO	THPDB0105
Odongo, F	TUAD0405
Odongo, I	TUAE0105
Odoyo, J	THPDC0102, WEAC0105
Odoyo-June, E	WEPDC0104
Ogum, E	WEPDE0104
Ohaga, S	WEPDC0104
Ohlen, C	THAA0101
Ojuok, S	WEPDC0102
Okal, J	TUPDD0302
Okala, SG	THAA0204^*^
Okello, E	WEPDC0102
Okello, V	WEAE0206LB
Okesola, N	FRAC0105LB
Oketch, J	WEPDC0105
Oldenburg, C	THAB0102
Oleson, S	TUPDD0203
Oliveira, R	THAB0106LB
Oliveras, E	THAE0206
Olsen, E	WEAC0304
Olson, GS	TUPDA0104
Oluwatimilehin, I	TUPDE0102
Omanga, E	FRAC0104, WEPDC0104
Omar, A	WEPDD0103
Omar, B	WEAC0403
Omwoyo, W	TUPDB0104
Ongwandee, S	THPDB0106
Onoya, D	WEAB0102^*^
Oo, SM	TUPDD0301
Oo, WL	TUPDB0104
Oosterhout	TUPDB0105
Operario, D	TUAD0104
Opolo, V	TUPDB0104
Opuni, M	THPDE0204
Orkin, C	TUPDC0105, WEAB0304LB
Orkin, M	TUAB0201
Ormaasen, V	THAB0202
Orne-Gliemann, J	FRAC0105LB
Orrell, C	FRAE0206LB, THAB0205LB^*^
Ortiz, K	THPDA0101
Osawa, K	WEPDA0101
Osawe, S	WEAB0103
Osher, B	THAE0305
Osinusi, A	WEAB0301, WEAB0302
Osman, M	WEAE0306LB
Ossanga, O	THAD0101
Oti, S	THAD0104
Otiende, P	WEPDC0105
Otieno, F	WEPDC0102, WEPDC0107
Otieno, FO	THAD0104^*^
Otieno-Nyunya, B	WEPDC0104
Ott, D	THAA0101
Ouédraogo, A	THAC0102
Ouma, D	WEPDC0105
Oundo, M	WEPDC0105
Outlaw, S	FRAD0105
Overbaugh, J	WEPDC0201
Owaraganise, A	FRAE0203, FRAE0205, WEAC0106LB
Owen, C	TUPDA0102
Owiti, F	TUAD0106LB
Oyebanji, O	WEAE0104
Ozorowski, G	WEPDA0101
**P**	
Padavattan, N	TUPDA0104
Padian, N	THAD0201, WEAE0105
Padmapriyadarsini, C	WEAB0201
Pahalawatta, V	THPDB0204
Paing, AK	TUPDD0301
Palanee-Philips, T	TUAC0105LB
Palanee-Phillips, T	WEPDC0203
Palmer, B	TUAX0105LB, TUPDA0103^*^
Palmer, S	TUAX0101LB
Pals, S	TUAC0204
Paltiel, AD	THAE0305
Panchal, N	THPDB0205
Pandey, SR	TUAD0201
Pandrea, I	THAA0205, THPDA0103, TUAA0102, TUAC0101, WEAA0103
Pankam, T	WEPDE0205
Pannetier, J	TUAD0103^*^
Pantazis, N	WEAA0105LB
Panya, M	WEAE0106LB
Pape, JW	WEAE0202
Papua, L	THAD0202
Parker, R	THAE0204, THPDE0201
Parker, RA	THAE0305
Parkes-Ratanshi, R	THAB0105
Pasipamire, L	FRAE0204^*^
Pasquet, A	WEAC0102
Pasricha, N	TUPDD0301
Passmore, J-A	TUPDA0105
Passmore, J-AS	WEAA0102
Pasternak, A	TUAA0104^*^
Patel, K	WEAB0105
Patel, P	FRAD0103
Patel, SV	TUPDC0104
Patel, VV	FRAC0101^*^
Paton, NI	TUPDB0105
Patriarca, T	FRAE0104
Patta, S	WEAC0403
Patterson, P	FRAB0104LB
Patterson, S	TUPDD0306
Paul, C	WEAE0202
Pavlakis, GN	THAA0201
Paz-Bailey, G	THAC0104
Peacock, D	TUPDD0303
Peel, S	THPDB0203
Peeters, M	TUPDB0104
Peitzmeier, S	WEPDC0202
Pena, S	WEAC0202
Pengnonyang, S	WEPDE0205
Pengo, V	WEPDC0102
Perez, M	TUAD0401
Perez-Brumer, A	TUAD0404^*^
Perez-Patrigeon, S	TUAA0105^*^
Perodin, C	WEAE0202
Perry, S	FRAE0201
Perumean-Chaney, SE	WEAC0305LB
Petdachai, W	THPDB0106
Petersen, M	FRAE0205, WEAC0106LB^*^
Petersen, ML	FRAE0203
Peterson, CW	THAA0103^*^
Peterson, E	TUAA0101
Petlo, C	THAE0302, THPDB0101
Pett, SL	FRAB0101LB, FRAB0102LB
Pettifor, A	TUPDD0303, WEAC0303^*^, WEPDC0205
Pé rez-Elías, MJ	TUPDB0106
Pfeiffer, K	WEAE0303
Phanuphak, N	THAC0101, TUAX0101LB, WEPDE0205
Phanuphak, P	TUAX0101LB, WEPDE0205
Phaswana-Mafuya, N	TUAD0302
Phillip, R	THAD0102^*^
Phillips, A	TUAX0103LB, TUPDC0105
Phillips, H	THAE0302
Phillips, R	WEAA0105LB
Phillips, T	WEPDE0106LB^*^
Phiri, S	TUAB0104
Phofa, R	THPDC0104
Phogat, S	TUAX0102LB
Picker, L	WEPDA0102
Pierre, MF	THAB0103LB
Pillay, D	FRAC0105LB, THAB0102, WEAE0204
Pillay, Y	TUAE0105, TUAE0106, TUPDC0102, WEPDE0103
Pillonel, J	TUAC0203
Pilotto, J	THAB0103LB, THAB0106LB
Pinkevych, M	THAA0101
Pinyakorn, S	TUAX0101LB
Pisarski, EE	FRAE0103
Piwowar-Manning, E	THAC0105LB, WEAC0104
Planas, D	THPDA0102
Plank, R	WEPDC0102
Plenty, A	TUAD0105, WEAC0106LB
Plotkin, M	THPDE0205
Podzamczer, D	THAB0206LB
Poku, N	THAE0103^*^
Polacino, P	THAA0103
Policicchio, B	THAA0205, THPDA0103^*^, TUAA0102, WEAA0103
Policicchio, BB	TUAC0101
Ponde, T	THPDE0105
Poojary, R	FRAC0101
Poon, K	TUAD0403
Porteiro, N	THAB0205LB
Porter, S	WEAE0205
Portilho, D	WEPDA0104
Postnov, O	THPDE0101
Power, J	THPDD0103^*^
Powis, K	WEAB0104
Powis, KM	WEAE0305
Pozniak, A	THPDE0206
Prado, J	THAA0202
Prakadan, S	TUAA0103
Preiser, W	WEPDB0102
Prejean, J	THAC0104^*^
Prendergast, AJ	FRAB0101LB
Prestage, G	FRAC0102, THAC0101
Price, K	FRAE0105, WEPDE0204
Prins, J	TUAA0104
Prybylski, D	TUPDC0104
Pujades Rodríguez, M	TUPDB0104
Pulerwitz, J	TUPDD0302
Pulsipher, C	WEAD0305
Purcell, D	THAC0104
Purcell, DFJ	THPDA0104
Puren, A	TUAC0201, TUAE0106, TUPDC0101
Pussadee, K	WEPDE0205
Puthanakit, T	THPDB0106^*^
**Q**	
Qin, Y	WEAE0102^*^
Quereda, C	TUPDB0106
**R**	
Rabie, H	TUAB0104
Rabkin, M	WEAE0106LB
Radakovich, N	WEPDA0101
Radhakrishnan, P	FRAD0204^*^
Radix, A	WEAC0202^*^
Raehtz, K	THAA0205, THPDA0103, WEAA0103^*^
Raehtz, KD	TUAC0101
Rahane, G	THPDB0205
Raja, K	WEAB0201
Rajapakse, C	FRAB0102LB
Rakesh, A	TUPDB0104
Rakhmanina, N	THAD0103^*^
Ramadhani, A	WEAE0106LB
Ramesh Kumar, S	WEAB0201
Ramjathan, P	THPDB0202^*^
Ramokolo, V	TUAE0106
Ramraj, T	TUAE0106
Ranasinghe, S	WEPDA0102^*^
Rangel, G	WEAC0405
Rao, A	TUAD0302^*^
Rassool, M	FRAB0103LB
Rassool, MS	WEPDB0101
Ratanasuwan, W	FRAB0103LB
Ravalihasy, A	TUAD0103
Ravichandran, N	WEAB0201
Raw, A	TUPDD0101^*^
Rawat, S	FRAC0101
Rawlings, K	TUAX0105LB^*^
Rawlins, S	FRAB0103LB
Reankhomfu, R	WEPDE0205
Rebombo, D	TUPDD0303^*^
Reddy, K	WEPDC0203
Reddy, N	THPDB0202
Reed, J	THAE0303
Rees, H	WEAD0204, WEPDC0206
Rehm, C	THAA0104LB
Reid, A	FRAB0102LB
Reid, C	THAA0101
Reisner, S	WEAC0203
Reisner, SL	TUAD0404
Rekacewicz, C	FRAC0105LB
Remien, RH	TUPDD0205^*^, WEPDE0106LB
Renju, J	WEAD0104^*^, WEPDD0101, WEPDE0102
Rennie, S	THPDD0104, THPDD0105, THPDD0106LB
Restar, A	TUAD0303
Rewari, B	THPDB0205
Reynaldi, A	THAA0101
Reynolds, M	TUAA0101
Rhodes, T	TUAD0106LB
Rhoe Davis, V	WEAD0103
Ribeiro, R	THPDA0103
Richardson, BA	WEPDC0201
Richardson, P	TUAC0102
Riche, B	TUAC0202
Richmond, G	THAB0206LB
Riley, N	TUAD0205
Rinehart, A	TUAC0102
Ristola, M	WEPDB0101
Rivera, V	WEAE0202
Rivero, M	WEPDB0105
Riviere, C	WEAE0202
Rizzardini, G	WEAB0304LB
Robb, M	TUPDA0102
Robbins, R	TUPDD0205
Robertson, B	WEPDB0102
Robinson, N	WEAA0105LB
Robinson, P	TUAE0105
Rocha, V	THAA0105
Rockstroh, JK	WEAB0304LB^*^
Rodger, A	TUPDC0105^*^
Rodgers, A	FRAB0103LB
Rodolph, M	TUAC0104
Rodriguez, CG	TUPDB0102
Rodriguez-Castañón, M	TUAA0105
Rojas, D	THAE0304
Rojas, E	FRAB0103LB
Rojas-Castro, D	WEAC0102
Rolæn, MJ	FRAB0104LB
Rolland, M	TUPDA0102^*^
Ronald, A	FRAE0106LB
Ronan, A	WEPDE0106LB
Rono, K	TUPDA0102
Rooney, J	TUAC0102, TUAX0104LB
Rosati, M	THAA0201
Rosen, S	WEAE0204
Rosenberg, M	WEPDC0205^*^
Rosenthal, K	WEAB0103
Rositch, A	TUPDA0105
Ross, J	WEPDB0101
Ross, M	WEPDB0101
Rotich, K	THAE0103
Roussow, T	THPDD0101
Routy, J-P	THPDA0102^*^
Rowe, J	TUPDD0205
Rozenbaum, W	WEAC0102
Ruane, P	WEAB0301
Ruark, A	WEAD0103^*^
Rubio, R	WEPDB0105
Rudy, B	TUAX0104LB
Ruhode, N	WEAE0103
Ruisenor-Escudero, H	THPDB0102
Rusch, B	FRAE0204
Russell, A	WEAD0302^*^
Russell, D	FRAC0102
Russell, S	TUAD0304
Rutledge, B	TUAX0104LB, WEAC0305LB
Rutter, L	FRAD0203
Ruxrungtham, K	TUPDC0105
Ryan, C	TUPDD0301
Rylance, J	THPDB0105
Ryom, L	WEPDB0101
**S**	
Sacha, J	TUAA0101
Sadamasu, K	THAX0101
Sadiq, N	TUPDD0305
Saez-Cirion, A	THAA0105
Sagaon-Teyssier, L	THAD0101
Sagwa, E	TUPDB0101
Saha, A	FRAD0103^*^
Sahabo, R	WEAE0206LB
Saintil, G	WEAE0202
Sajwani, K	WEAB0302
Sakoi, M	THPDB0101
Salas-Ortiz, A	THPDE0204
Salgado, M	THAA0105
Salters, K	TUPDD0306
Salumu, L	THPDC0103, TUPDB0104
Salzwedel, J	THPDD0102^*^
Sam-Agudu, NA	WEPDE0104^*^
Samba, BM	THAB0104
Samleerat, T	THPDB0106
Samsunder, N	FRAE0102, TUAC0201, TUPDA0101, TUPDA0105
Samuelson, J	THAE0303
Sanchez, AM	THAX0105
Sanchez, J	TUAD0404
Sanchez, M	TUPDD0306
Sanders-Buell, E	TUPDA0102
Sandfort, T	TUAD0303
Sane, S	THPDB0205
Sang, E	THPDB0103
Sang, N	WEAC0106LB
Sangrujee, N	THAE0302
Sanne, I	THPDC0104
Santangelo, P	THPDA0101
Santos, B	THAB0103LB, THAB0106LB
Sardesai, NY	THAA0201
Sarr, M	WEAB0205LB
Sartorius, B	WEAC0302
Sattayapanich, T	WEPDE0205
Saunders, P	FRAD0105^*^
Sawadogo, S	TUPDC0104
Sawry, S	TUAB0104
Sawyer, A	WEPDC0202
Sawyerr, G	THAB0104
Sax, P	FRAB0103LB
Schaafsma, T	WEAE0304
Schechter, M	THAB0201
Scheepers, E	TUAE0101
Scheim, AI	WEAC0205
Schmidt, H-M	FRAE0105
Schmidt, HMA	WEPDE0204
Schmitz, K	TUAE0101
Schneider, J	THAX0103, TUPDA0103
Schnure, M	THAE0303
Schoor	THPDE0102
Schramm, B	TUPDB0104^*^
Schulze zur Wiesch, J	THAA0105
Schutte, C	THPDE0205
Schutz, C	WEAB0203
Schwartz, JL	FRAE0102
Schwartz, S	THPDC0104, THPDC0105, TUAB0203, TUAD0302
Scott, GB	WEAB0105
Seage, G	THAB0102
Sebidi, J	WEPDE0103
Sebogodi, P	WEAE0305
Sedlacek, D	THAB0202
Seeley, J	TUAD0304, WEPDD0101
Seifert-Ahanda, K	WEPDC0106
Sein, TT	TUPDD0301
Sekar, L	WEAB0201
Sekar, S	WEAB0201
Sekiziyivu, A	TUPDA0102
Selin, A	TUPDD0303, WEAC0303, WEPDC0205
Sellers, T	FRAD0103
Selvey, C	FRAE0105, WEPDE0204
Semini, I	THAE0103
Semitala, FC	WEAB0202^*^
Seraise, B	WEAE0305
Sereda, Y	THPDE0101
Serrano, L	TUPDB0104
Serrano, S	TUPDB0106
Seto, E	THAX0102
Severe, P	WEAE0202
Shade, SB	FRAE0203^*^
Shah, SA	THAX0105
Shahmanesh, M	THAB0102
Shaikh, N	WEAE0104
Shalek, A	WEPDA0102
Shalek, AK	TUAA0103
Shamu, A	WEAE0303
Shamu, T	THAD0102
Shao, W	THAA0104LB
Shapiro, D	THAB0103LB
Shapiro, R	WEAB0104
Shapley-Quinn, MK	WEPDC0203
Sharkey, T	THPDE0201
Sharma, S	THAB0202
Shattock, R	THAA0204
Shembilu, C	WEPDE0203
Shen, G	WEAB0302
Shenoi, S	FRAC0103^*^
Shepard, C	WEAB0305LB
Sherman, D	TUAD0402
Sherman, G	TUAB0102, TUAE0106
Sherman, J	TUAE0104
Sherman, S	WEPDC0202
Sherr, K	TUAE0103
Sherr, L	THAD0204, TUAB0201
Sherwood, J	THAE0105, WEAD0306LB
Shiino, T	THAX0101^*^
Shikely, K	WEAC0403
Shingwenyana, N	TUPDE0102
Shodell, D	TUAC0204
Shoham, T	THAD0104
Shokoohi, M	WEAC0205^*^
Shoptaw, S	THAC0105LB, WEAC0104
Shossi, M	WEPDD0103
Shrestha, R	TUAD0201
Shroufi, A	THAB0101, WEAE0301
Shubber, Z	TUPDC0102
Shubert, V	FRAD0106LB
Shulman, NS	WEAB0304LB
Shutt, A	THPDB0203
Shvab, I	THPDE0101
Sibanda, E	WEAE0103
Sibanda, EL	WEAE0105^*^
Sibanda, G	FRAE0204
Sibeko, S	WEAA0102
Siberry, G	TUAX0104LB
Siegel, A	TUAC0103
Siegler, A	FRAE0101^*^
Sierra-Madero, J	THAB0202, TUAA0105
Sievwright, K	TUPDD0106
Siika, A	FRAB0101LB, FRAB0102LB, TUPDB0105
Sikorskii, A	THPDB0102
Sikweyiya, Y	TUAD0203, TUPDD0304
Silva, C	WEAE0302
Silva-Santisteban, A	TUAD0404
Silvis Rustagi, A	TUAE0103
Simon, K	WEAB0204^*^
Simon, M-C	WEAC0102
Simon, Y	WEAD0304
Simonetti, FR	THAA0104LB
Simons, E	WEAE0301
Simonyan, V	THAX0105
Sineke, T	WEAB0102
Singano, V	THPDE0102
Singh, A	TUPDA0105
Singh, D	TUPDD0106
Singh, Y	TUAE0106
Sinikithemba Cohort	THAA0202
Sinywimaanzi, K	THPDB0206
Sipemba, J	WEAE0106LB
Sista, N	THAD0106LB
Sithole, J	TUPDE0103
Sivanandham, R	THPDA0103
Skiles, M	THAE0302
Skinner, A	THPDD0104
Skinner, D	THPDD0101
Skoutelis, A	THAB0201
Skovdal, M	TUAD0405
Sloan, L	THAB0206LB
Slogrove, A	WEAC0301^*^
Small, W	TUAD0102
Smith, K	FRAD0106LB, THAB0205LB, THAB0206LB, THAX0105
Smith, KS	FRAC0102
Smyrnov, P	WEAC0404
Snyman, K	FRAE0205
Soghoian, D	WEPDA0102
Solomon, D	THPDE0103
Somboonwit, C	WEPDB0101
Somda, M	THAC0102
Sopheap, S	THAE0301
Sosa-Rubi, SG	THPDE0202
Sosa-Rubí, SG	THPDE0204
Soto-Malave, R	WEAB0304LB
Soto-Ramirez, L	TUAA0105
Soto-Torres, L	TUAC0105LB
Souda, S	WEAB0104
Souleymanov, R	TUPDD0204^*^
Sovannarith, S	THAE0301
Sowerbutts, H	WEAE0306LB
Soyizwaphi, P	TUAB0203
Spencer, S	FRAD0102
Sperling, RS	WEAB0105
Spiassi, A	TUPDD0206
Spire, B	THAC0102, THAD0101, THAE0304, WEAC0102
Spreen, W	THAB0206LB
Springer, S	WEAC0402^*^
Squires, K	FRAB0103LB
Sridhar, R	WEAB0201
Srinivasan, S	WEPDC0201
Ssewamala, F	THAD0205
St Clair, M	THAB0206LB
Stadler, J	THAD0203, WEAD0204^*^, WEPDC0206*
Stall, R	WEAC0204
Stam, A	THAA0105
Stamm, L	WEAB0302
Stangl, A	TUPDD0106^*^
Staunton, C	THPDD0101
Steele, SJ	THAB0101^*^
Stegman, P	THAE0303
Stein, D	TUAD0104, TUPDD0205
Steingo, J	TUAB0203
Stellbrink, H-J	THAB0206LB
Stephanos, S	WEAC0202
Stern, E	TUPDD0303
Stevens, W	TUAB0205, TUAC0205
Steward, WT	WEAD0305
Stieh, DJ	WEAA0101
Stinson, K	TUAB0104
Stock, J	THAA0205, TUAA0102, WEAA0103
Stone, J	WEAC0404^*^
Stone, M	THAX0105, WEAA0106LB
Stover, J	THAE0103
Stranix-Chibanda, L	THAB0106LB^*^
Strathde, S	TUAD0106LB
Strathdee, SA	WEAC0405
Stratton, T	WEPDD0106
Strauss, M	THAE0103
Streeck, H	WEPDA0102
Struchiner, CJ	THAE0305
Su, C	THAX0105
Subtil, F	TUAC0202
Sued, O	FRAB0104LB
Sugarman, J	THPDD0105
Suggu, K	TUAE0102
Sugiura, W	THAX0101
Suleman, M	TUAA0103
Sulkowski, M	WEAB0301
Sullivan, E	WEAD0306LB
Sullivan, K	TUAD0301
Sullivan, P	FRAE0101, TUPDD0203^*^
Sultana, N	TUPDD0305
Sun, LP	THAE0301
Supervie, V	TUAC0203
Sutcliffe, C	THPDB0206
Suzan-Monti, M	THAD0101^*^
Swaminathan, S	WEAB0201
Swomen, H	WEPDE0104
Sykes, C	TUAC0101
Sylla, L	THPDD0104
Szubert, AJ	FRAB0101LB, FRAB0102LB
Szumilin, E	FRAE0201, TUPDB0104
**T**	
Taege, A	WEAE0102
Taha, T	THPDB0102
Talima, D	WEAD0202
Tam, A	TUAD0304^*^
Tam, M	FRAE0103
Tang, J	WEPDA0105
Tang, K	TUAD0305
Tang, N	THPDB0204
Tang, S	WEAE0102
Tang, W	WEAE0102
Tanser, F	FRAC0105LB, THAB0102, THAX0104, TUAB0104, WEAE0204
Taramusi, I	WEPDD0105
Tariko, L	WEAC0403
Tate, J	WEAC0401
Taylor, J	THPDD0102, THPDD0104
Taylor, M	WEAC0302
Taylor, S	TUPDD0203
Taylor, T	WEAD0205^*^
Tazhibayeva, G	TUPDB0103
Tchetgen Tchetgen, EJ	TUPDC0103, WEAE0305
Technau, K-G	TUAB0104
Telfer, B	TUAB0202, WEPDE0204
Tello-Mercado, A	TUAA0105
Telnov, A	TUPDB0104
Temblay, C	THAE0304
Tepper, V	THPDB0101
Teppler, H	FRAB0103LB
Terris-Prestholt, F	THPDE0205
Thaisri, H	THPDB0106
Thambinayagam, A	TUPDE0103
Thein, ZW	TUPDD0301
Theron, G	THAB0106LB
Thiam, M	TUAD0202
Thiebaut, R	FRAC0105LB
Thirumurthy, H	FRAC0104, FRAE0205^*^, WEAE0101, WEAE0105
Thobakgale, C	THAA0202
Thomas, A	THAE0303, WEPDD0104
Thomas, K	WEAE0304
Thomason, M	TUAB0204
Thompson, JA	TUPDB0105^*^
Thompson, R	TUAC0204
Thulare, H	THAE0204
Thuma, P	THPDB0206^*^
Thure, K	FRAE0101
Tichacek, A	THPDC0101, THPDE0201, WEAD0101
Tierney, C	THAB0106LB
Tietz, D	FRAD0106LB
Tindimwebwa, E	THPDC0102, WEAC0105
Tjituka, F	TUPDB0101
Tobian, A	WEPDC0106
Tocco, J	TUAD0303
Todd, J	THAD0104
Toledo, C	TUAC0201
Tomaras, G	TUAX0102LB
Tomko, C	WEPDC0202
Tomlinson, C	FRAD0203^*^
Tong, C	THAD0101
Topp, SM	THPDE0104
Torrens, A	WEAE0302
Torres-Rueda, S	THPDE0205^*^
Torriente, A	TUPDD0104^*^
Toska, E	THAD0204^*^, TUAB0201
Tovanabutra, S	TUPDA0102
Trachunthong, D	WEPDE0205
Tracy, R	TUAA0102
Trautmann, L	TUAX0101LB
Tremblay, C	WEAC0102
Tressler, R	THPDD0105
Treves-Kagan, S	TUPDD0303
Trexler, C	WEAD0203
Trichel, A	WEAA0103
Trinh, R	WEAB0304LB
Trottier, B	THAB0203
Tshuma, ES	TUPDC0106
Tu, W	THPDB0103
Tucker, J	THPDD0106LB, WEAE0102
Tumushime, M	WEAE0105
Tumwekwase, G	TUAD0304
Tun, W	TUPDD0301^*^
Turay, M	THAE0103
Turkova, A	TUAB0103
Turner, D	FRAC0103
Tuswa, N	TUPDE0102
Twine, R	TUPDD0303, WEPDC0205
**U**	
Ujamaa, D	TUAC0204
Uldrick, T	THAA0104LB
Ulrich, A	THAX0102
Underhill, K	TUAD0104
Urasa, P	WEAE0106LB
Uwacu, T	TUPDE0104
Uwamahoro, D	TUPDE0104
**V**	
Vail, R	WEAC0202
Valentin, A	THAA0201
Van Cutsem, G	THAB0101, THPDC0103
van Delft, Y	THAB0104
van den Berg, K	WEAA0106LB^*^
Van den Eede, P	TUPDB0105
van der Stok, M	THAA0202
van der Straten, A	WEPDC0203^*^
van Dijk, J	WEAE0303^*^
Van Dyke, R	WEAB0105
Van Gorder, D	WEAD0305
van, JJ	TUPDB0105
Van Lith, L	WEPDC0106
van Oosterhout, J	THPDE0102
Van Rie, A	THPDC0104, TUAB0203
Van, RM	THPDA0104
van Rooyen, H	WEAD0102
van, V	THPDE0102
van Widenfelt, E	TUPDC0103, WEAE0305
Vandenbulcke, A	TUAC0202, TUPDB0104
Vanderkerckhove, L	THAB0201
Vannakit, R	WEPDE0205^*^
Vavro, C	THAB0205LB
Veazey, RS	WEAA0101
Vega, C	TUAA0105
Vela, I	THAC0103
Veloso, VG	THAE0305
Velu, V	THAA0206
Vergara-Mendoza, M	TUAA0105
Vermeulen, H	WEPDB0102
Vermeulen, M	WEAA0106LB
Viana, R	THPDB0204
Viani, RM	WEAB0304LB
Vichea, C	THAE0301
Vickerman, P	WEAC0404, WEAC0405
Vidal, L	THAD0101
Villaran, M	THAX0102
Villinger, F	THPDA0101
Virga, A	TUPDD0105^*^
Vivancos, MJ	TUPDB0106
Viveros-Rogel, M	TUAA0105
Vreeman, R	THPDB0103^*^
Vu, L	TUPDD0302^*^
Vubil, A	TUPDB0104
Vwalika, B	THPDC0101, THPDE0201, WEAD0101
Vwalika, C	WEAD0101
**W**	
Wachira, S	FRAB0102LB
Wacleche, VS	THPDA0102
Wade, AS	THAC0102
Wagner, R	WEAC0303, WEPDD0102
Wagner, RG	WEPDC0205
Wake, R	THPDB0201^*^
Wakefield, S	THAD0106LB
Walensky, RP	THAE0305
Walker, AS	FRAB0101LB, FRAB0102LB, TUPDB0105
Walker, BD	THAA0202, TUPDA0104, WEAA0104, WEPDA0102
Walker, S	TUAB0204
Walkowiak, H	TUPDB0101
Wall, K	THPDC0101, THPDE0201^*^, WEAD0101^*^
Wallis, CL	THPDB0204^*^
Walmsley, S	THAB0205LB
Wamai, R	THPDE0204
Wamai, RG	THPDE0202
Wamalwa, D	THPDB0104
Wambura, M	THPDE0205
Wambuzi Ogwang, L	THPDB0102
Wame, M	THAE0302
Wamoyi, J	TUAD0405^*^, WEPDD0101, WEPDE0102
Wand, H	FRAC0102
Wang, A	WEAB0305LB
Wang, J	THAA0103, WEAC0303
Wang, L	WEAB0305LB
Wang, R	TUPDC0103
Wang, Y	TUAD0101
Wang’ombe, J	THPDE0202, THPDE0204
Wanjala, S	TUAC0202
Wanjiru, E	WEAE0301
Ward, A	WEAB0203^*^, WEPDA0101
Ware, NC	FRAE0103
Warne, P	TUAB0101, WEPDB0103
Warren, M	THAE0106
Washington, L	TUAD0203, TUPDD0304
Wasserheit, J	TUAE0103
Waterhouse, C	FRAD0203
Watkins, P	THAC0105LB, WEAC0104
Watters, S	THAA0104LB
Wavamunno, P	FRAB0102LB
Weber, J	WEAA0105LB
Weber, R	WEAE0205
Webster, K	TUPDD0306
Wei, C	WEAE0102
Wei, S	TUAC0204
Wei, X	WEAB0305LB
Weinberg, A	THAB0103LB
Weiner, B	THPDD0104
Weinfurter, J	TUAA0101
Weiss, HA	THPDE0205
Weiss, S	WEAC0202
Wensing, AM	THAA0105^*^
Were, E	THAB0104
Werner, L	WEAA0102
West, N	TUAB0203
Wexler, D	THAE0204
Wheeler, D	THAC0105LB, WEAC0104^*^
Wheeler, DP	WEAC0103
White, R	THAD0106LB
Wiche Salinas, TR	THPDA0102
Wiener, J	WEAB0305LB
Wightman, F	THPDA0104
Wijewardana, V	WEAA0103
Wijne, M-L	THAD0202
Wilhelm, A	THAE0301, THPDE0203^*^
Wilkin, T	TUAC0102
Wilkinson, RJ	WEAB0203
Willan, S	TUAD0203, TUPDD0304
Willberg, CB	WEAA0105LB
Willenberg, L	TUPDD0301
Williams, D	TUPDE0102
Williams, I	THAB0201
Williams, OD	TUPDA0106
Williams, P	THAB0206LB, THPDB0101, TUAC0103, WEAB0105
Williamson, C	TUPDA0101
Wilson, C	TUAA0102, TUAA0106LB
Wilson, CM	TUAX0104LB, WEAC0305LB
Wilson, D	WEPDB0104
Wilton, L	THAC0105LB
Windle, M	TUPDD0106
Wingo, E	WEPDC0202
Wirth, K	TUPDC0103, WEAE0305
Wiselka, MJ	THAB0201
Wiseman, R	TUAA0101
Wiznia, A	WEPDB0103
Woeber, K	WEPDC0203
Woelk, G	WEPDE0201^*^
Wolkon, A	TUPDC0104
Womack, V	WEAE0306LB
Wong, EB	TUAA0103^*^
Wong, JP-H	TUAD0403
Wood, R	TUAB0104
Workowski, K	WEAB0301
Worley, MJ	THAA0203^*^
Wringe, A	FRAE0201^*^, THAD0104, TUAD0405, WEAD0104, WEPDD0101, WEPDE0102
Wuhib, T	THAE0302
Wyatt, C	FRAE0106LB, WEPDB0101
Wyatt, MA	FRAE0103^*^
Wyles, D	WEAB0301, WEAB0304LB
Wynne, B	THAB0203, THAB0205LB
**X**	
XGó mez-Olivé	WEAC0303
Xia, M	THAC0104
Xiao, Y	WEAD0105
Xu, C	THAA0205, THPDA0103, TUAA0102^*^, TUAC0101
Xu, X	FRAB0103LB
Xulu, B	TUAA0103
Xulu, T	WEAA0106LB
**Y**	
Yakubovich, A	TUAB0201
Yam, E	TUPDD0301, TUPDD0302, TUPDD0305
Yancor, MP	THAC0103
Yang, H	TUAX0101LB
Yarchoan, R	THAA0104LB
Yasmin, R	TUPDD0305
Yavuz, E	TUAE0102
Yelgate, R	THPDB0205
Yende, N	THAA0202, THPDC0104^*^
Yende-Zuma, N	FRAE0102
Yohnka, R	THPDE0201
Yola, N	THAD0106LB
Yomb, Y	THAC0102
Yoon, C	WEAB0202
Yorick, R	THPDE0101
Yosef, N	WEPDA0102
Yoshimura, K	THAX0101
Young, A	TUAC0102
Young, M	WEPDC0107
Yuhas, K	WEPDC0201
Yunusa, F	WEPDE0104
**Z**	
Zaba, B	THAD0104
Zablotska, I	FRAE0105^*^, THAC0101, FRAE0204
Zampieri, T	TUPDD0206
Zandamela, A	THPDE0103^*^
Zanolini, A	WEAE0101^*^
Zapiola, I	TUPDB0102
Zash, R	WEAB0104^*^
Zaza, S	WEAC0304
Zeh, C	TUPDB0104
Zembe, L	TUAC0201
Zeng, W	THAE0102
Zerbe, A	WEPDE0106LB
Zevin, A	TUAA0106LB
Zhang, F	WEAB0305LB
Zhang, H	TUAD0305
Zhang, W	WEAE0102
Zhang, Y	THPDA0102
Zhanpeisova, A	TUPDB0103
Zhao, Y	TUAD0305
Zhu, L	THAA0106LB
Zieman, B	TUPDD0302, TUPDD0305
Zulu, C	THAE0102
Zuma, T	WEPDD0101
Zuñiga	WEAC0405
Zurla, C	THPDA0101
